# Supplementary material for: Asymmetric formal C–C bond insertion into aldehydes via copper-catalyzed diyne cyclization
Source: Nat Commun. 2023 Nov 3;14:7058. doi: 10.1038/s41467-023-42805-2 (PMC10624849; doi:10.1038/s41467-023-42805-2)
Supplement: Supplementary file 1 — Supplementary Information [file 41467_2023_42805_MOESM1_ESM.pdf]

Supplementary Information

Asymmetric formal C–C bond insertion into aldehydes via copper-catalyzed diyne cyclization

Cui-Ting Li,[1] Lin-Jun Qi,[1] Li-Gao Liu,[1] Chang Ge,[1] Xin Lu,[*,1]

Long-Wu Ye,[1,2] and Bo Zhou[*,1]

[1]State Key Laboratory of Physical Chemistry of Solid Surfaces, Key Laboratory of Chemical Biology of Fujian Province, and College of Chemistry and Chemical Engineering, Xiamen University, Xiamen 361005, China.

[2]State Key Laboratory of Organometallic Chemistry, Shanghai Institute of Organic Chemistry, Chinese Academy of Sciences, Shanghai 200032, China.

*Email: zhoubo@xmu.edu.cn; xinlu@xmu.edu.cn

Table of Contents

I. Supplementary Methods 2

1.1 General Information 2

1.2 More Reaction Condition and Substrate Scope Studies 3

1.3 Preparation of Starting Materials 9

1.4 General Procedure for the Formal C–C Bond Insertion 34

1.5 Synthetic Utility Study 71

1.6 Crystal Data 82

1.7 DFT calculations 84

1.8 NMR Spectra 88

1.9 HPLC Chromatograms 198

II. Supplementary References 235

I. Supplementary Methods

1.1 General Information

Acetonitrile (ACS grade), toluene (ACS grade), trifluorotoluene (ACS grade), ethyl acetate (ACS grade), 1,4-dioxane (ACS grade), methanol (ACS grade), 1,2-dichloroethane (ACS grade), dichloromethane (ACS grade), and hexanes (ACS grade) were obtained commercially and used without further purification. Tetrahydrofuran (THF) was purified according to standard methods unless otherwise noted. Commercially available reagents were used without further purification. Reactions were monitored by thin layer chromatography (TLC) using silicycle pre-coated silica gel plates. Flash column chromatography was performed over silica gel (300-400 mesh). Infrared spectra were recorded on a Nicolet AVATER FTIR330 spectrometer as thin film and are reported in reciprocal centimeter (cm^{-1}). Mass spectra were recorded with Micromass QTOF2 Quadrupole/Time-of-Flight Tandem mass spectrometer using electron spray ionization.

^{1}H NMR spectra, ^{13}C NMR and ^{31}P NMR spectra were recorded on a Bruker AV-400 spectrometer and a Bruker AV-500 spectrometer in chloroform-d. For ^{1}H NMR spectra, chemical shifts are reported in ppm with the internal TMS signal at 0.0 ppm as a standard. For ^{13}C NMR spectra, chemical shifts are reported in ppm with the internal chloroform signal at 77.0 ppm as a standard.

1.2 More Reaction Condition and Substrate Scope Studies

Supplementary Table 1. Screening of more reaction conditions for the formal C–C bond insertion[a]

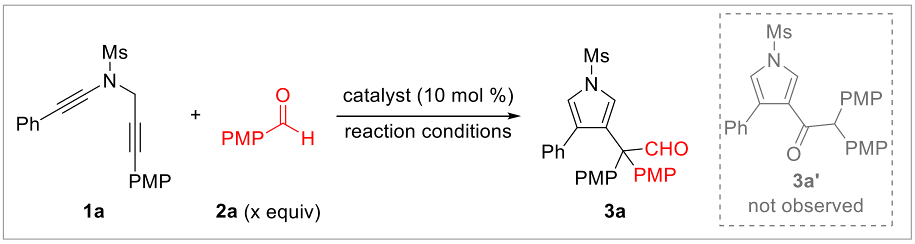

Entry	Catalyst	Reaction conditions	Yield(%)[b]
1	$Cu(OAc)_2$	DCE, 30 °C, x = 2, 12 h	<1
2	$Cu(acac)_2$	DCE, 30 °C, x = 2, 12 h	<1
3	CuBr	DCE, 30 °C, x = 2, 12 h	<1
4	$Cu(CH_3CN)_4OTf$	DCE, 30 °C, x = 2, 12 h	<1
5	$Cu(CH_3CN)_4PF_6$	$CHCl_3$, 60 °C, x = 4, 1 h	<1
6	$Cu(CH_3CN)_4PF_6$	THF, 60 °C, x = 4, 2 h	<1
7	$Cu(CH_3CN)_4PF_6$	1,4-dioxane, 60 °C, x = 4, 3 h	<1
8	$Cu(CH_3CN)_4PF_6$	PhCl, 60 °C, x = 4, 3 h	48
9	$Cu(CH_3CN)_4PF_6$	DCE, 60 °C, x = 4, 3Å MS, 0.5 h	<1
10	$Cu(CH_3CN)_4PF_6$	DCE, 60 °C, x = 4, 4Å MS, 0.5 h	<1
11	$Cu(CH_3CN)_4PF_6$	DCE, 60 °C, x = 4, 5Å MS, 0.5 h	56
12	$Cu(CH_3CN)_4PF_6$	DCE, 60 °C, x = 4, $NaBAr^F_4$, 0.5 h	46

[a]Reaction conditions: **1a** (0.05 mmol), **2a** (0.1–0.2 mmol), catalyst (0.005 mmol), solvent (1 mL), 30 °C to 60 °C, 0.5–12 h, in vials. [b]Determined by ^{1}H NMR spectroscopy using 1,3,5-trimethoxybenzene as the internal standard. Ms = methanesulfonyl, PMP = 4-methoxyphenyl, DCE = 1,2-dichloroethane.

As shown in Supplementary Table 1, the reaction gave almost no conversion using other catalysts, including $Cu(OAc)_2$, $Cu(acac)_2$ and CuBr (entries 1–3). Further screening of $Cu(CH_3CN)_4OTf$ catalyst and solvents produced messy results (entries 4–7) or low yield (entry 8). The reaction demonstrated no conversion after adding 3Å MS and 4Å MS (entries 9–10). Other additives such as 5Å MS and $NaBAr^F_4$ failed to improve the reaction. Importantly, the competitive formal C–H bond insertion into aldehyde (ketone byproduct **3a′**) was not observed in above conditions.

Supplementary Table 2. Screening of more reaction conditions for the atroposelective formal C–C bond insertion[a]

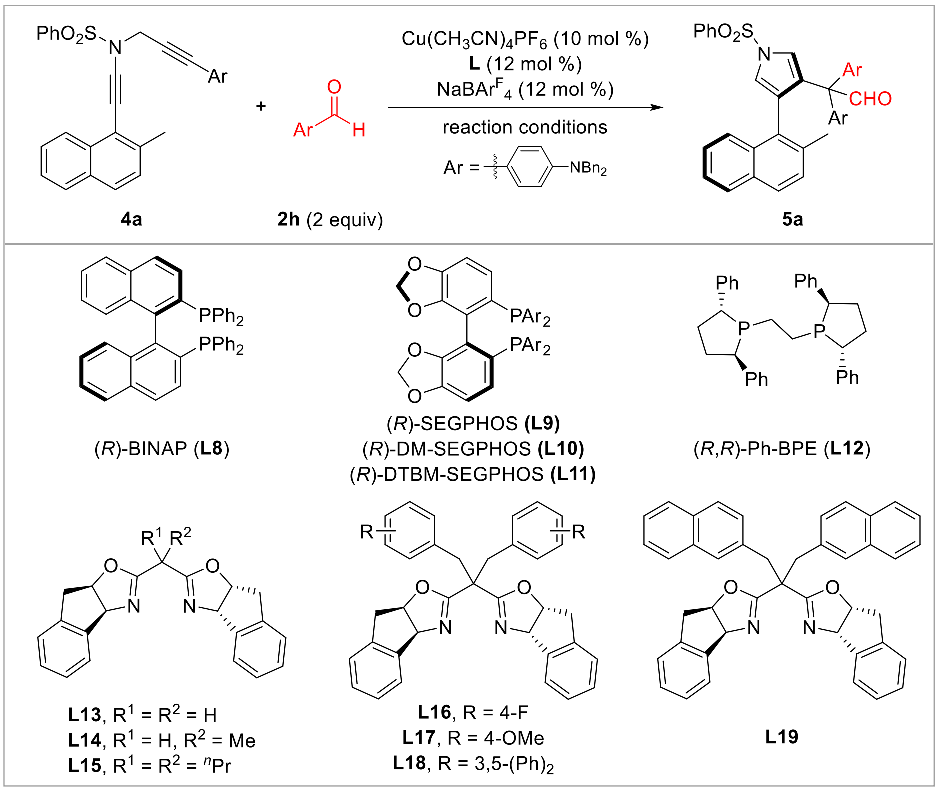

Entry	L	Reaction conditions	Yield (%)[b]	ee (%)[c]
1	**L8**	DCE, 30 °C, 1 h	74	25
2	**L9**	DCE, 30 °C, 1 h	79	56
3	**L10**	DCE, 30 °C, 1 h	80	33
4	**L11**	DCE, 30 °C, 1 h	76	77
5	**L12**	DCE, 30 °C, 12 h	<1	--
6	**L13**	DCE, 30 °C, 1 h	70	29
7	**L14**	DCE, 30 °C, 1 h	73	10
8	**L15**	DCE, 30 °C, 1 h	81	7
9	**L16**	DCE, 30 °C, 1 h	83	68
10	**L17**	DCE, 30 °C, 4 h	75	73
11	**L18**	DCE, 30 °C, 1 h	74	70
12	**L19**	DCE, 30 °C, 1 h	76	70

[a]Reaction conditions: **4a** (0.05 mmol), **2h** (0.1 mmol), $Cu(CH_3CN)_4PF_6$ (0.005 mmol), **L** (0.006 mmol), $NaBAr^F_4$ (0.006 mmol), solvent (1 mL), 30 °C, N_2, 1–12 h, in Schlenk tubes. [b]Measured by ^1H NMR using 1,3,5-trimethoxybenzene as the internal standard. [c]Determined by HPLC analysis.

As shown in Supplementary Table 2, the optimization of more chiral ligands was

carried out in the presence of $Cu(CH_3CN)_4PF_6$ catalyst. Bisphosphine ligands, including BINAP and SEGPHOS type ligands (**L8**–**L11**), gave moderate to good yields with 25–77% ees (entries 1–4). However, the reaction demonstrated no conversion when using (*R*,*R*)-Ph-BPE as ligand (entry 5). Further investigation into different SaBOX ligands by adjusting the sidearms showed the critical role of sidearm-effect in this atroposelective formal C–C bond insertion (entries 6–12).

Supplementary Figure 1. Screening of other diynes **1** and aldehydes **2** for the formal C–C bond insertion[a]

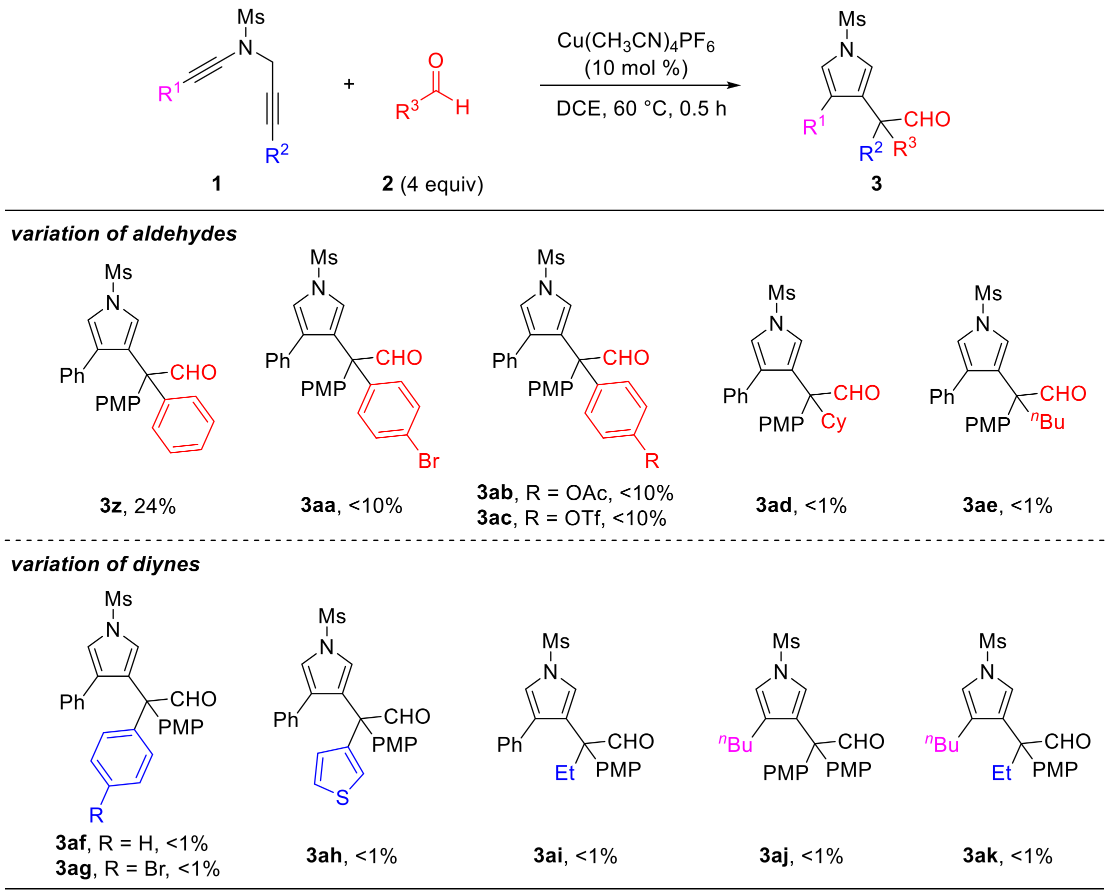

[a]Reaction conditions: **1** (0.2 mmol), **2** (0.8 mmol), $Cu(CH_3CN)_4PF_6$ (0.02 mmol), DCE (4 mL), 60 °C, 0.5 h, in vials; yields were determined by ^{1}H NMR using 1,3,5-trimethoxybenzene as the internal standard.

As shown in Supplementary Figure 1, the scope of aryl aldehydes **2** was further investigated under optimized conditions. The simple benzaldehyde (**3z**) and aryl aldehydes with substituents including Br (**3aa**), OAc (**3ab**), OTf (**3ac**), resulted in low yields. The reaction of alkyl aldehydes (**3ad** and **3ae**) also failed to deliver any intermolecular trapping product. It’s clear that the electron-rich aryl aldehydes gave better results compared with electron-poor aldehydes, due to the higher nucleophilicity. The reaction of non-substituted diyne (**3af**) as well as bromide (**3ag**) and thiophene (**3ah**) containing diynes only led to the hydrolysis of ynamide. Moreover, the reaction of alkyl diynes (R^1 or R^2 is alkyl, **3ai** and **3aj**) or dialkyl

aldehyde diynes (R^1 and R^2 are alkyl, **3ak**) only gave inseparable mixtures. Therefore, the suitable nucleophilicity of aldehyde and alkyne is important for this reaction.

Supplementary Figure 2. Screening of other diynes **4** and aldehydes **2** for the atroposelective formal C–C bond insertion[a]

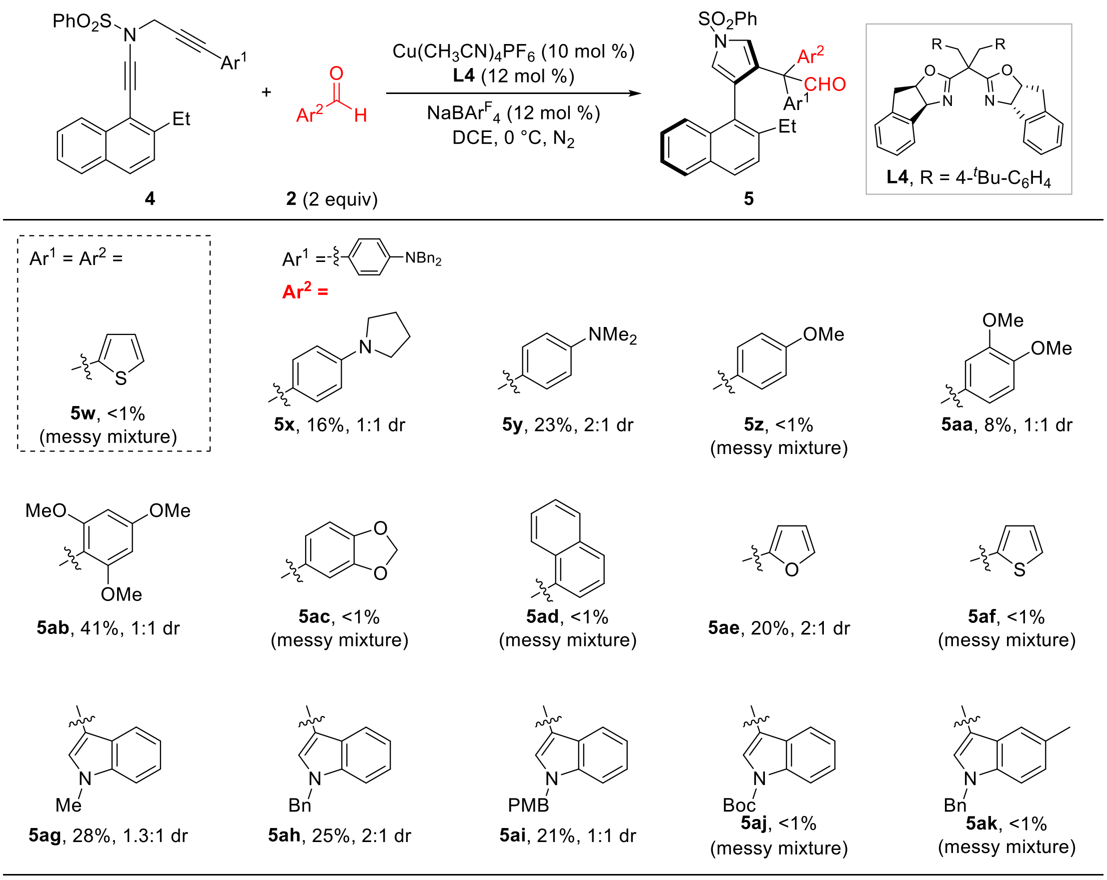

[a]Reaction conditions: **4** (0.1 mmol), **2** (0.2 mmol), $Cu(CH_3CN)_4PF_6$ (0.01 mmol), **L4** (0.012 mmol), $NaBAr^F{}_4$ (0.012 mmol), DCE (2 mL), 0 °C, 10–20 h, N_2, in Schlenk tubes; yields were determined by ^1H NMR using 1,3,5-trimethoxybenzene as the internal standard.

As depicted in Supplementary Figure 2, our attempts to extend the atroposelective formal C–C bond insertion to the other electron-rich aryl aldehydes led to poor results (**5w**–**5ak**), which might due to the direct intermolecular attack of these nucleophiles onto ynamides. Among these, several aryl aldehydes ($Ar^1 \neq Ar^2$) demonstrated low yields with 1:1 to 2:1 dr values. Thus, the appropriate nucleophilicity of aldehyde is also necessary for the atroposelective transformation.

1.3 Preparation of Starting Materials

Supplementary Figure 3. List of known aldehydes

The aryl aldehydes **2a**, **2b**, **2d–2g**, **2i**, **2j**, **2n**, **2o** and **2r-2v** are commercially available. Aryl aldehydes **2c**[1], **2h**[2], **2k**[3], **2l**[3], **2m**[4], **2p**[5], **2q**[5] and **2w-2z**[4] are known compounds, which were prepared according to known procedures.

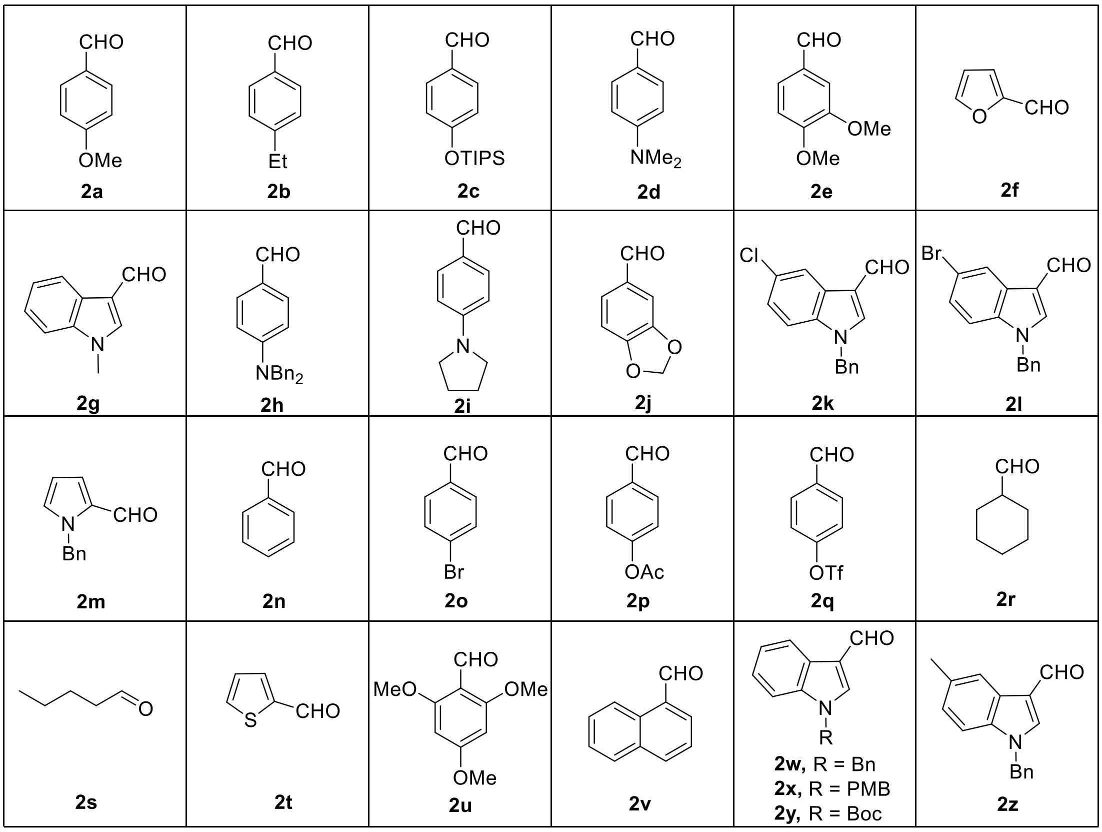

Supplementary Figure 4. List of known diynes

The diynes **1b–1e**[6], **1s**[7], **1t**[6], **1u-1y**[6] and **4t**[6] are known compounds, which were prepared according to known procedures.

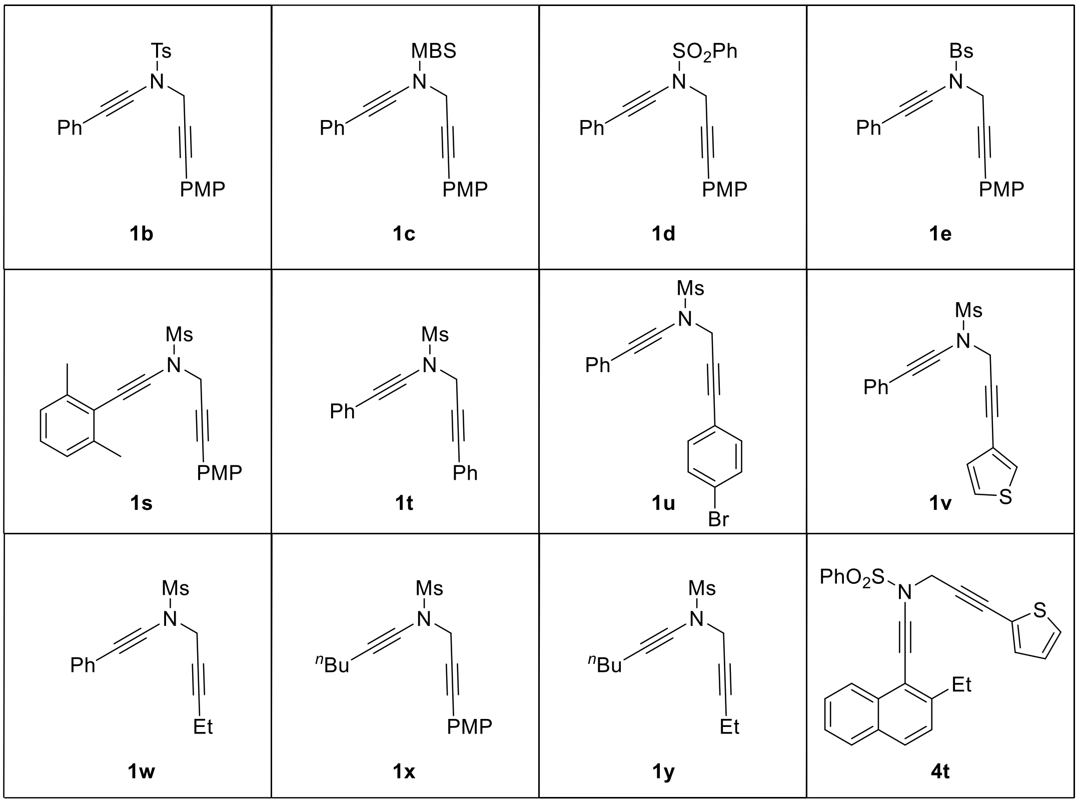

Synthetic procedures for the preparation of diynes 1 and 4:

Synthetic procedure A (**1**, **4q–4s**)[8]:

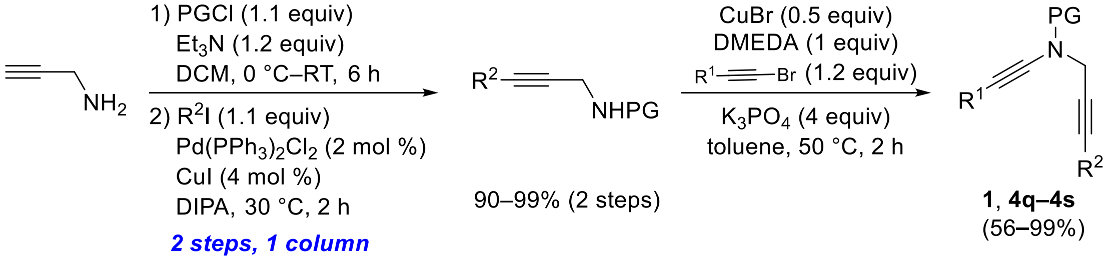

To a solution of propargylamine (0.3 mL, 5.0 mmol) and Et_3N (0.8 mL, 6.0 mmol) in DCM (25 mL) was slowly added the corresponding sulfonyl chloride (5.5 mmol) at 0 °C. Then the resulting mixture was stirred at room temperature for 6 h and the progress of the reaction was monitored by TLC. Upon completion, the reaction was quenched with HCl (1.0 M), extracted with DCM for 3 times, dried over Na_2SO_4 and filtered. The filtrate was concentrated under reduced pressure to afford the crude propargylamide without further purification. To the solution of above propargylamide in diisopropylamine (15 mL) were added corresponding aryl iodide (5.5 mmol), $Pd(PPh_3)_2Cl_2$ (70.2 mg, 0.1 mmol) and CuI (38.1 mg, 0.2 mmol) under N_2 atmosphere. The reaction was stirred at 30 °C for 2 h and the progress of the reaction was

monitored by TLC. Upon completion, the reaction mixture was filtered and concentrated under reduced pressure. The residue was purified by column chromatography on silica gel (eluent: PE/EtOAc) to afford the desired propargyl sulfonamide (90–99% yields, 2 steps).

To a solution of above propargyl sulfonamide (1.0 mmol) in toluene (5 mL) were added the corresponding alkynyl bromide (1.2 mmol), CuBr (71.7 mg, 0.5 mmol), DMEDA (0.1 mL, 1.0 mmol) and K_3PO_4 (849.2 mg, 4.0 mmol). The resulting mixture was stirred at 50 °C for 2 h, and the progress of the reaction was monitored by TLC. Upon completion, the reaction mixture was filtered and concentrated under reduced pressure. The residue was purified by column chromatography on silica gel (eluent: PE/EtOAc) to afford the desired diyne **1** or **4q-4s** in 56–99% yields.

Synthetic procedure B (**4a**–**4p**):

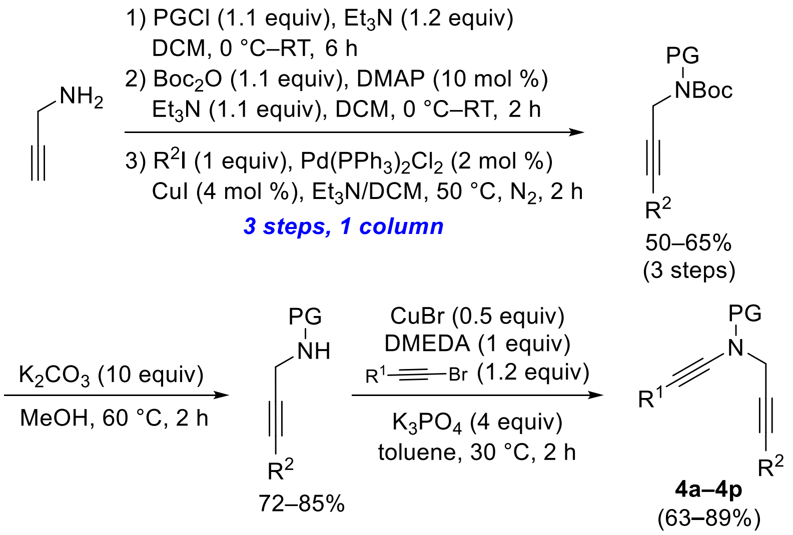

To a solution of propargylamine (0.3 mL, 5.0 mmol) and Et_3N (0.8 mL, 6.0 mmol) in DCM (25 mL) was slowly added the corresponding sulfonyl chloride (5.5 mmol) at 0 °C. Then the resulting mixture was stirred at room temperature for 6 h and the progress of the reaction was monitored by TLC. Upon completion, the reaction was quenched with HCl (1.0 M), extracted with DCM for 3 times, dried over Na_2SO_4 and filtered. The filtrate was concentrated under reduced pressure to afford the crude protected propargylamide without further purification. To the solution of above protected propargylamide in DCM (20 mL) were added DMAP (61.1 mg, 0.5 mmol),

Et_3N (0.8 mL, 5.5 mmol) and Boc_2O (1.3 mL, 5.5 mmol) at 0 °C. Then the resulting mixture was stirred at room temperature for 2 h and the progress of the reaction was monitored by TLC. Upon completion, the reaction was quenched with HCl (1.0 M), extracted with DCM for 3 times, dried over Na_2SO_4 and filtered. The filtrate was concentrated under reduced pressure to afford the crude Boc-protected propargylamide without further purification. To the solution of above Boc-protected propargylamide in Et_3N (15 mL) and DCM (3 mL) were added corresponding aryl iodide (5 mmol), $Pd(PPh_3)_2Cl_2$ (70.2 mg, 0.1 mmol) and CuI (38.1 mg, 0.2 mmol) under N_2 atmosphere. The reaction was stirred at 50 °C for 2 h and the progress of the reaction was monitored by TLC. Upon completion, the reaction mixture was filtered and concentrated under reduced pressure. The residue was purified by column chromatography on silica gel (eluent: PE/EtOAc) to afford the desired propargyl sulfonamide (50–65% yields, 3 steps).

To a solution of above propargyl sulfonamide (2.0 mmol) in MeOH (20 mL) was added K_2CO_3 (2.76 g, 20 mmol). The reaction was stirred at 60 °C for 2 h and the progress of the reaction was monitored by TLC. Upon completion, the reaction mixture was concentrated under reduced pressure. The resulting mixture was then diluted with water, extracted with EtOAc for 3 times, dried over $MgSO_4$ and concentrated under reduced pressure. The residue was purified by column chromatography on silica gel (eluent: PE/EtOAc) to afford the desired propargyl sulfonamide in 72–85% yields.

To a solution of above propargyl sulfonamide (1.0 mmol) in toluene (5 mL) were added the corresponding alkynyl bromide (1.2 mmol), CuBr (71.7 mg, 0.5 mmol), DMEDA (0.1 mL, 1.0 mmol) and K_3PO_4 (849.2 mg, 4.0 mmol). The resulting mixture was stirred at 30 °C for 2 h, and the progress of the reaction was monitored by TLC. Upon completion, the reaction mixture was filtered and concentrated under reduced pressure. The residue was purified by column chromatography on silica gel (eluent: PE/EtOAc) to afford the desired diyne **4a-4p** in 63–89% yields.

***N*-(3-(4-methoxyphenyl)prop-2-yn-1-yl)-*N*-(phenylethynyl)methanesulfonamide**

(1a)

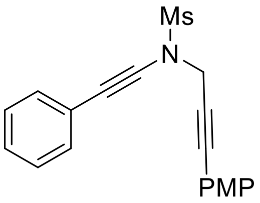

1a

Compound **1a** was prepared in 86% yield (291.9 mg) according to procedure A as a colorless oil (eluent: PE/EtOAc = 5/1). ^{1}H NMR (400 MHz, $CDCl_3$) δ 7.46 – 7.42 (m, 2H), 7.38 (d, *J* = 8.8 Hz, 2H), 7.30 – 7.26 (m, 3H), 6.83 (d, *J* = 9.0 Hz, 2H), 4.56 (s, 2H), 3.76 (s, 3H), 3.23 (s, 3H); ^{13}C NMR (100 MHz, $CDCl_3$) δ 160.0, 133.2, 131.5, 128.2, 128.0, 122.1, 114.0, 113.5, 86.9, 81.3, 80.0, 71.1, 55.1, 42.9, 38.5; IR (neat): 2927(bs), 2236(s), 1605, 1509, 1361, 1249, 1166, 1110, 1031, 756, 516 cm^{-1}; HRESIMS Calcd for $[C_{19}H_{17}NNaO_3S]^+$ (M + Na^+) 362.0821, found 362.0808.

***N*-((4-chlorophenyl)ethynyl)-*N*-(3-(4-methoxyphenyl)prop-2-yn-1-yl)methanesulfonamide (1f)**

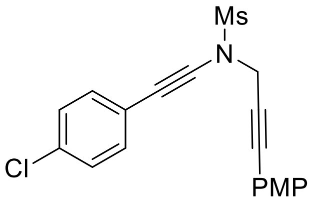

1f

Compound **1f** was prepared in 73% yield (273.0 mg) according to procedure A as a colorless oil (eluent: PE/EtOAc = 5/1). ^{1}H NMR (500 MHz, $CDCl_3$) δ 7.40 – 7.34 (m, 4H), 7.26 (d, *J* = 9.0 Hz, 2H), 6.84 (d, *J* = 8.5 Hz, 2H), 4.57 (s, 2H), 3.78 (s, 3H), 3.25 (s, 3H); ^{13}C NMR (125 MHz, $CDCl_3$) δ 160.1, 134.0, 133.2, 132.7, 128.5, 120.7, 114.0, 113.5, 87.0, 82.2, 79.9, 70.1, 55.2, 42.9, 38.7; IR (neat): 2927(bs), 2236(s), 1606, 1509, 1361, 1251, 1165, 1108, 1033, 765, 517 cm^{-1}; HRESIMS Calcd for $[C_{19}H_{16}ClNNaO_3S]^+$ (M + Na^+) 396.0432, found 396.0438.

***N*-((4-bromophenyl)ethynyl)-*N*-(3-(4-methoxyphenyl)prop-2-yn-1-yl)methanesulfonamide (1g)**

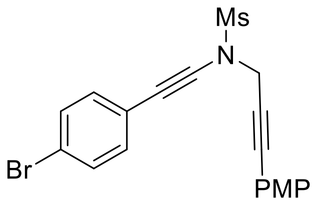

1g

Compound **1g** was prepared in 57% yield (238.4 mg) according to procedure A as a colorless solid (mp 85–86 °C, eluent: PE/EtOAc = 5/1). ^{1}H NMR (400 MHz, $CDCl_3$) δ 7.43 – 7.36 (m, 4H), 7.29 (d, *J* = 8.8 Hz, 2H), 6.84 (d, *J* = 8.8 Hz, 2H), 4.57 (s, 2H), 3.79 (s, 3H), 3.25 (s, 3H); ^{13}C NMR (100 MHz, $CDCl_3$) δ 160.1, 133.2, 132.9, 131.4, 122.2, 121.2, 114.0, 113.4, 87.0, 82.4, 79.9, 70.2, 55.2, 42.9, 38.7; IR (neat): 2930(bs), 2236(s), 1586, 1485, 1362, 1263, 1165, 1114, 1070, 755, 518 cm^{-1}; HRESIMS Calcd for $[C_{19}H_{16}BrNNaO_3S]^+$ (M + Na$^+$) 439.9926, found 439.9935.

methyl 4-((*N*-(3-(4-methoxyphenyl)prop-2-yn-1-yl)methylsulfonamido)ethynyl)benzoate (1h)

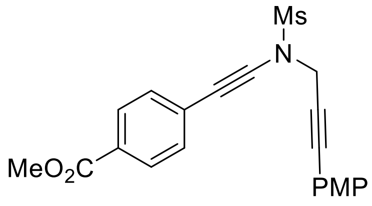

1h

Compound **1h** was prepared in 62% yield (246.4 mg) according to procedure A as a colorless solid (mp 113–114 °C, eluent: PE/EtOAc = 5/1). ^{1}H NMR (400 MHz, $CDCl_3$) δ 7.96 (d, *J* = 8.4 Hz, 2H), 7.47 (d, *J* = 8.4 Hz, 2H), 7.39 (d, *J* = 8.8 Hz, 2H), 6.85 (d, *J* = 8.8 Hz, 2H), 4.61 (s, 2H), 3.89 (s, 3H), 3.79 (s, 3H), 3.28 (s, 3H); ^{13}C NMR (100 MHz, $CDCl_3$) δ 160.0, 159.7, 133.6, 133.2, 114.0, 113.9, 113.7, 86.8, 80.2, 79.9, 70.8, 55.2, 55.1, 43.1, 38.4; IR (neat): 2932(bs), 2233(s), 1721, 1605, 1509, 1365, 1250, 1165, 1118, 1035, 768, 513 cm^{-1}; HRESIMS Calcd for $[C_{21}H_{19}NNaO_5S]^+$ (M + Na$^+$) 420.0876, found 420.0886.

***N*-(3-(4-methoxyphenyl)prop-2-yn-1-yl)-4-methyl-*N*-(*p*-tolylethynyl)benzenesulfo**

namide (1i)

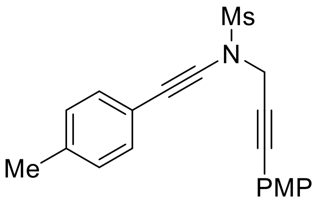

1i

Compound **1i** was prepared in 89% yield (314.6 mg) according to procedure A as a colorless oil (eluent: PE/EtOAc = 5/1). ^{1}H NMR (500 MHz, $CDCl_3$) δ 7.38 (d, *J* = 8.5 Hz, 2H), 7.34 (d, *J* = 8.0 Hz, 2H), 7.09 (d, *J* = 8.0 Hz, 2H), 6.83 (d, *J* = 9.0 Hz, 2H), 4.55 (s, 2H), 3.77 (s, 3H), 3.23 (s, 3H), 2.31 (s, 3H); ^{13}C NMR (125 MHz, $CDCl_3$) δ 160.0, 138.2, 133.2, 131.6, 128.9, 119.0, 113.9, 113.6, 86.8, 80.6, 80.1, 71.0, 55.1, 42.9, 38.3, 21.2; IR (neat): 2927(bs), 2236(s), 1606, 1509, 1361, 1251, 1165, 1108, 1033, 765, 517 cm^{-1}; HRESIMS Calcd for $[C_{20}H_{19}NNaO_3S]^+$ (M + Na$^+$) 376.0978, found 376.0975.

***N*-((4-methoxyphenyl)ethynyl)-*N*-(3-(4-methoxyphenyl)prop-2-yn-1-yl)methanesulfonamide (1j)**

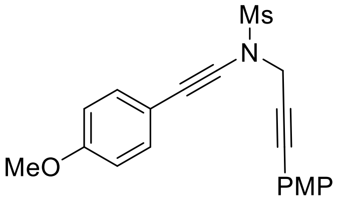

1j

Compound **1j** was prepared in 66% yield (243.8 mg) according to procedure A as a colorless oil (eluent: PE/EtOAc = 5/1). ^{1}H NMR (400 MHz, $CDCl_3$) δ 7.41 – 7.38 (m, 4H), 6.87 – 6.82 (m, 4H), 4.57 (s, 2H), 3.81 (s, 3H), 3.80 (s, 3H), 3.25 (s, 3H); ^{13}C NMR (100 MHz, $CDCl_3$) δ 160.1, 159.8, 133.7, 133.3, 114.2, 114.1, 113.9, 113.8, 86.9, 80.3, 80.0, 70.9, 55.3, 55.2, 43.2, 38.5; IR (neat): 2930(bs), 2239(s), 1605, 1508, 1362, 1248, 1165, 1107, 1037, 778, 514 cm^{-1}; HRESIMS Calcd for $[C_{20}H_{19}NNaO_4S]^+$ (M + Na$^+$) 392.0927, found 392.0917.

***N*-((3-chlorophenyl)ethynyl)-*N*-(3-(4-methoxyphenyl)prop-2-yn-1-yl)methanesulf**

onamide (1k)

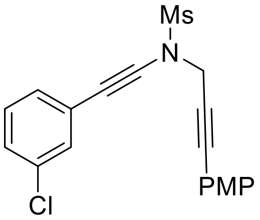

1k

Compound **1k** was prepared in 78% yield (291.6 mg) according to procedure A as a colorless oil (eluent: PE/EtOAc = 5/1). ^{1}H NMR (500 MHz, $CDCl_3$) δ 7.43 – 7.37 (m, 3H), 7.32 – 7.28 (m, 1H), 7.25 – 7.19 (m, 2H), 6.84 (d, *J* = 8.5 Hz, 2H), 4.58 (s, 2H), 3.78 (s, 3H), 3.25 (s, 3H); ^{13}C NMR (125 MHz, $CDCl_3$) δ 160.1, 133.9, 133.2, 131.1, 129.4, 128.2, 124.0, 114.0, 113.4, 87.1, 82.5, 79.8, 70.0, 55.1, 42.9, 38.7; IR (neat): 2928(bs), 2237(s), 1605, 1508, 1361, 1256, 1166, 1123, 1035, 764, 513 cm^{-1}; HRESIMS Calcd for $[C_{19}H_{16}ClNNaO_3S]^+$ (M + Na^+) 396.0432, found 396.0438.

***N*-(3-(4-methoxyphenyl)prop-2-yn-1-yl)-*N*-(*m*-tolylethynyl)methanesulfonamide (1l)**

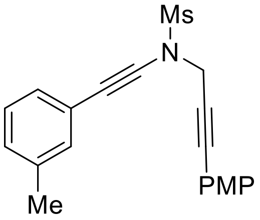

1l

Compound **1l** was prepared in 56% yield (197.9 mg) according to procedure A as a colorless oil (eluent: PE/EtOAc = 5/1). ^{1}H NMR (400 MHz, $CDCl_3$) δ 7.39 (d, *J* = 8.8 Hz, 2H), 7.28 – 7.23 (m, 2H), 7.20 – 7.15 (m, 1H), 7.11 – 7.07 (m, 1H), 6.83 (d, *J* = 8.8 Hz, 2H), 4.55 (s, 2H), 3.77 (s, 3H), 3.23 (s, 3H), 2.29 (s, 3H); ^{13}C NMR (100 MHz, $CDCl_3$) δ 160.0, 137.8, 133.2, 132.1, 128.9, 128.5, 128.1, 121.9, 113.9, 113.6, 86.9, 81.0, 80.1, 71.2, 55.1, 42.9, 38.4, 21.0; IR (neat): 2929(bs), 2238(s), 1605, 1509, 1362, 1249, 1165, 1109, 1034, 781, 515 cm^{-1}; HRESIMS Calcd for $[C_{20}H_{19}NNaO_3S]^+$ (M + Na^+) 376.0978, found 376.0975.

N-(cyclopropylethynyl)-N-(3-(4-methoxyphenyl)prop-2-yn-1-yl)methanesulfonamide (1m)

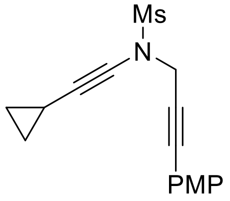

1m

Compound **1m** was prepared in 72% yield (218.4 mg) according to procedure A[9] as a pale yellow solid (mp 70–71 °C, eluent: PE/EtOAc = 5/1). ^{1}H NMR (400 MHz, $CDCl_3$) δ 7.38 (d, J = 8.8 Hz, 2H), 6.85 (d, J = 8.8 Hz, 2H), 4.43 (s, 2H), 3.81 (s, 3H), 3.16 (s, 3H), 1.39 – 1.33 (m, 1H), 0.87 – 0.78 (m, 2H), 0.75 – 0.67 (m, 2H); ^{13}C NMR (100 MHz, $CDCl_3$) δ 160.0, 133.2, 114.0, 113.7, 86.5, 80.3, 75.4, 67.9, 55.2, 42.9, 38.0, 8.9, -0.9; IR (neat): 2931(bs), 2246(s), 1606, 1509, 1360, 1349, 1164, 1093, 1030, 776, 519 cm^{-1}; HRESIMS Calcd for $[C_{16}H_{17}NNaO_3S]^+$ (M + Na$^+$) 326.0821, found 326.0822.

N-(phenylethynyl)-N-(3-(p-tolyl)prop-2-yn-1-yl)methanesulfonamide (1n)

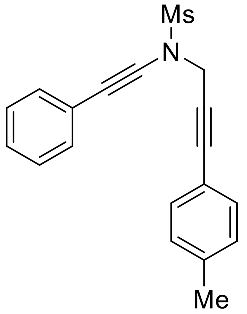

1n

Compound **1n** was prepared in 99% yield (320.2 mg) according to procedure A as a colorless oil (eluent: PE/EtOAc = 5/1). ^{1}H NMR (400 MHz, $CDCl_3$) δ 7.45 – 7.42 (m, 2H), 7.34 (d, J = 8.4 Hz, 2H), 7.28 – 7.24 (m, 3H), 7.10 (d, J = 8.0 Hz, 2H), 4.54 (s, 2H), 3.22 (s, 3H), 2.31 (s, 3H); ^{13}C NMR (100 MHz, $CDCl_3$) δ 139.1, 131.5, 131.4, 129.0, 128.1, 128.0, 122.0, 118.4, 87.0, 81.3, 80.7, 71.0, 42.7, 38.3, 21.2; IR (neat):

2928(bs), 2235(s), 1598, 1485, 1360, 1260, 1164, 1115, 1037, 757, 515 cm^{-1}; HRESIMS Calcd for [$C_{19}H_{17}NNaO_2S$]$^+$ (M + Na$^+$) 346.0872, found 346.0869.

***N*-(3-(4-(benzyloxy)phenyl)prop-2-yn-1-yl)-*N*-(phenylethynyl)methanesulfonamide (1o)**

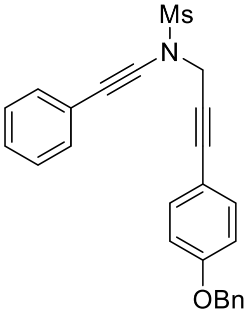

1o

Compound **1o** was prepared in 90% yield (373.9 mg) according to procedure A as a colorless oil (eluent: PE/EtOAc = 5/1). ^{1}H NMR (400 MHz, $CDCl_3$) δ 7.45 – 7.42 (m, 2H), 7.41 – 7.32 (m, 6H), 7.32 – 7.26 (m, 4H), 6.90 (d, *J* = 8.8 Hz, 2H), 5.02 (s, 2H), 4.54 (s, 2H), 3.21 (s, 3H); ^{13}C NMR (100 MHz, $CDCl_3$) δ 159.2, 136.3, 133.3, 131.5, 128.5, 128.2, 128.1, 128.0, 127.3, 122.1, 114.9, 113.9, 86.9, 81.3, 80.1, 71.1, 69.9, 42.9, 38.5; IR (neat): 2927(bs), 2237(s), 1604, 1508, 1362, 1244, 1166, 1111, 1038, 750, 516 cm^{-1}; HRESIMS Calcd for [$C_{25}H_{21}NNaO_3S$]$^+$ (M + Na$^+$) 438.1134, found 438.1114.

***N*-(3-(2-methoxyphenyl)prop-2-yn-1-yl)-*N*-(phenylethynyl)methanesulfonamide (1p)**

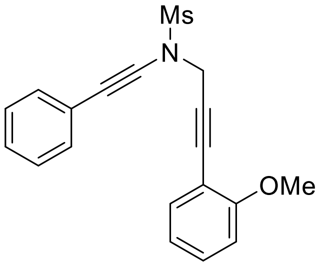

1p

Compound **1p** was prepared in 95% yield (322.4 mg) according to procedure A as a colorless oil (eluent: PE/EtOAc = 5/1). ^{1}H NMR (400 MHz, $CDCl_3$) δ 7.45 – 7.37 (m,

3H), 7.33 – 7.24 (m, 4H), 6.92 – 6.82 (m, 2H), 4.60 (s, 2H), 3.78 (s, 3H), 3.31 (s, 3H); ^{13}C NMR (100 MHz, $CDCl_3$) δ 160.1, 133.3, 131.5, 130.3, 128.1, 128.0, 122.1, 120.3, 110.7, 110.6, 85.2, 83.4, 81.5, 70.9, 55.3, 43.1, 38.3; IR (neat): 2931(bs), 2236(s), 1596, 1493, 1435, 1360, 1166, 1115, 1024, 751, 518 cm^{-1}; HRESIMS Calcd for $[C_{19}H_{17}NNaO_3S]^+$ (M + Na^+) 362.0821, found 362.0808.

***N*-(3-(benzo[*d*][1,3]dioxol-5-yl)prop-2-yn-1-yl)-*N*-(phenylethynyl)methanesulfonamide (1q)**

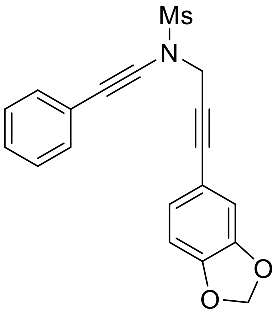

1q

Compound **1q** was prepared in 73% yield (258.0 mg) according to procedure A as a colorless solid (mp 74–75 °C, eluent: PE/EtOAc = 5/1). ^{1}H NMR (400 MHz, $CDCl_3$) δ 7.47 – 7.42 (m, 2H), 7.33 – 7.28 (m, 3H), 6.99 (dd, *J* = 8.0, 1.6 Hz, 1H), 6.89 (d, *J* = 1.2 Hz, 1H), 6.76 (d, *J* = 8.0 Hz, 1H), 5.97 (s, 2H), 4.57 (s, 2H), 3.25 (s, 3H); ^{13}C NMR (100 MHz, $CDCl_3$) δ 148.5, 147.5, 131.6, 128.3, 128.2, 126.7, 122.2, 114.8, 111.7, 108.5, 101.4, 86.9, 81.3, 79.8, 71.2, 43.0, 38.6; IR (neat): 2927(bs), 2232(s), 1602, 1488, 1361, 1249, 1166, 1113, 1037, 780, 518 cm^{-1}; HRESIMS Calcd for $[C_{19}H_{15}NNaO_4S]^+$ (M + Na^+) 376.0614, found 376.3602.

***N*-(3-(naphthalen-1-yl)prop-2-yn-1-yl)-*N*-(phenylethynyl)methanesulfonamide (1r)**

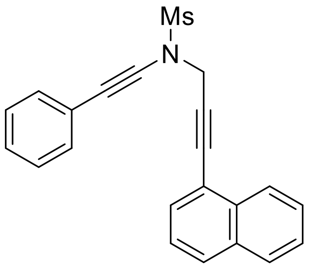

1r

Compound **1r** was prepared in 95% yield (341.5 mg) according to procedure A as a colorless oil (eluent: PE/EtOAc = 5/1). ^{1}H NMR (400 MHz, $CDCl_3$) δ 8.38 (d, *J* = 8.4 Hz, 1H), 7.81 – 7.78 (m, 2H), 7.68 – 7.54 (m, 1H), 7.51 – 7.43 (m, 3H), 7.42 – 7.34 (m, 2H), 7.31 – 7.24 (m, 3H), 4.71 (s, 2H), 3.22 (s, 3H); ^{13}C NMR (100 MHz, $CDCl_3$) δ 133.1, 132.9, 131.6, 130.7, 129.4, 128.2(4), 128.2(0), 128.1, 127.0, 126.4, 125.6, 125.0, 122.0, 119.1, 86.2, 85.0, 81.4, 71.4, 43.0, 38.4; IR (neat): 2928(bs), 2236(s), 1585, 1505, 1442, 1362, 1166, 1113, 1033, 775, 515 cm^{-1}; HRESIMS Calcd for $[C_{22}H_{17}NNaO_2S]^+$ (M + Na^+) 382.0872, found 382.0865.

***N*-(3-(4-(dibenzylamino)phenyl)prop-2-yn-1-yl)-*N*-((2-methylnaphthalen-1-yl)ethynyl)benzenesulfonamide (4a)**

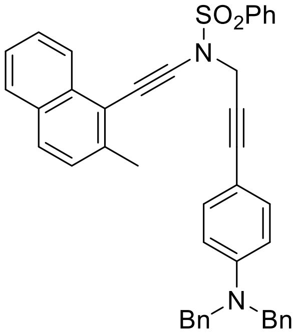

4a

Compound **4a** was prepared in 78% yield (492.0 mg) according to procedure B as a colorless solid (mp 79–80 °C, eluent: PE/EtOAc = 5/1). ^{1}H NMR (500 MHz, $CDCl_3$) δ 8.25 (d, *J* = 8.5 Hz, 1H), 8.02 (d, *J* = 7.5 Hz, 2H), 7.67 (d, *J* = 8.0 Hz, 1H), 7.58 (d, *J* = 8.5 Hz, 1H), 7.48 – 7.44 (m, 1H), 7.38 – 7.34 (m, 2H), 7.30 – 7.27 (m, 3H), 7.24 – 7.18 (m, 5H), 7.14 (d, *J* = 7.5 Hz, 4H), 7.08 (s, 1H), 7.00 (d, *J* = 9.0 Hz, 2H), 6.54 (d, *J* = 9.0 Hz, 2H), 4.61 (s, 2H), 4.56 (s, 4H), 2.53 (s, 3H); ^{13}C NMR (125 MHz, $CDCl_3$)

δ 149.0, 138.7, 137.6, 137.1, 133.6, 133.4, 133.0, 131.3, 128.9, 128.6, 128.0, 127.7(4), 127.7(3), 127.5, 127.0, 126.6, 126.3, 125.9, 125.2, 118.6, 111.7, 109.0, 90.6, 87.7, 78.9, 68.5, 53.9, 43.2, 21.2; IR (neat): 2921(bs), 2228(s), 1606, 1519, 1362, 1261, 1171, 1089, 1028, 748, 594 cm^{-1}; HRESIMS Calcd for $[C_{42}H_{34}N_2NaO_2S]^+$ (M + Na^+) 653.2233, found 653.2210.

***N*-(3-(4-(dibenzylamino)phenyl)prop-2-yn-1-yl)-4-methyl-*N*-((2-methylnaphthalen-1-yl)ethynyl)benzenesulfonamide (4b)**

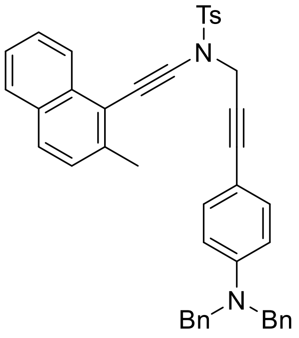

4b

Compound **4b** was prepared in 82% yield (528.7 mg) according to procedure B as a colorless solid (mp 110–111 °C, eluent: PE/EtOAc = 5/1). ^{1}H NMR (400 MHz, $CDCl_3$) δ 8.24 (d, *J* = 8.4 Hz, 1H), 7.93 (d, *J* = 8.4 Hz, 2H), 7.74 (d, *J* = 8.0 Hz, 1H), 7.65 (d, *J* = 8.4 Hz, 1H), 7.37 – 7.27 (m, 8H), 7.23 – 7.18 (m, 7H), 6.99 (d, *J* = 8.8 Hz, 2H), 6.57 (d, *J* = 8.8 Hz, 2H), 4.65 – 4.62 (m, 6H), 2.58 (s, 3H), 2.27 (s, 3H); ^{13}C NMR (100 MHz, $CDCl_3$) δ 149.1, 144.7, 138.7, 137.8, 134.4, 133.5, 133.1, 131.4, 129.6, 128.7, 128.2, 127.9, 127.8, 127.5, 127.1, 126.6, 126.5, 126.1, 125.3, 118.9, 111.8, 109.4, 91.0, 87.6, 79.0, 68.5, 54.1, 43.4, 21.5, 21.4; IR (neat): 2921(bs), 2226(s), 1607, 1519, 1362, 1263, 1169, 1089, 1028, 747, 590 cm^{-1}; HRESIMS Calcd for $[C_{43}H_{36}N_2NaO_2S]^+$ (M + Na^+) 667.2390, found 667.2374.

***N*-(3-(4-(dibenzylamino)phenyl)prop-2-yn-1-yl)-4-methoxy-*N*-((2-methylnaphthalen-1-yl)ethynyl)benzenesulfonamide (4c)**

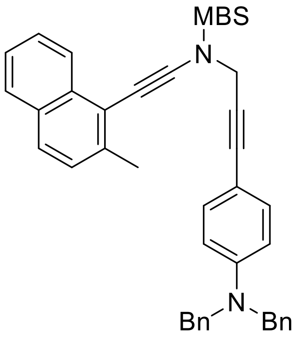

4c

Compound **4c** was prepared in 72% yield (475.8 mg) according to procedure B as a colorless solid (mp 66–67 °C, eluent: PE/EtOAc = 3/1). ^{1}H NMR (500 MHz, $CDCl_3$) δ 8.25 (d, *J* = 8.5 Hz, 1H), 7.97 – 7.93 (m, 2H), 7.71 (d, *J* = 8.0 Hz, 1H), 7.62 (d, *J* = 8.5 Hz, 1H), 7.36 – 7.27 (m, 6H), 7.25 – 7.21 (m, 3H), 7.17 (d, *J* = 8.0 Hz, 4H), 7.00 (d, *J* = 9.0 Hz, 2H), 6.85 – 6.82 (m, 2H), 6.55 (d, *J* = 9.0 Hz, 2H), 4.61 (s, 4H), 4.60 (m, 2H), 3.59 (s, 3H), 2.57 (s, 3H); ^{13}C NMR (125 MHz, $CDCl_3$) δ 163.7, 149.0, 138.7, 137.7, 133.5, 133.0, 131.4, 130.4, 128.7, 128.6, 127.8, 127.7, 127.5, 127.0, 126.6, 126.4, 126.0, 125.2, 118.8, 114.1, 111.7, 109.3, 91.1, 87.6, 79.1, 68.4, 55.4, 54.0, 43.2, 21.3; IR (neat): 2917(bs), 2229(s), 1604, 1518, 1362, 1262, 1163, 1091, 1027, 733, 553 cm^{-1}; HRESIMS Calcd for $[C_{43}H_{36}N_2NaO_3S]^+$ (M + Na$^+$) 683.2339, found 683.2325.

4-bromo-*N*-(3-(4-(dibenzylamino)phenyl)prop-2-yn-1-yl)-*N*-((2-methylnaphthalen-1-yl)ethynyl)benzenesulfonamide (4d)

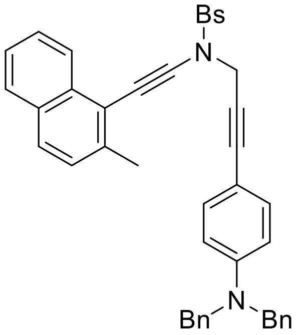

4d

Compound **4d** was prepared in 75% yield (532.3 mg) according to procedure B as a colorless solid (mp 58–59 °C, eluent: PE/EtOAc = 10/1). ^{1}H NMR (500 MHz, $CDCl_3$)

δ 8.21 (d, *J* = 8.5 Hz, 1H), 7.88 (d, *J* = 8.5 Hz, 2H), 7.73 (d, *J* = 8.0 Hz, 1H), 7.65 (d, *J* = 8.5 Hz, 1H), 7.53 – 7.50 (m, 2H), 7.35 – 7.27 (m, 8H), 7.24 – 7.21 (m, 1H), 7.19 (d, *J* = 8.0 Hz, 4H), 6.97 (d, *J* = 8.5 Hz, 2H), 6.59 (d, *J* = 8.5 Hz, 2H), 4.64 (s, 2H), 4.63 (s, 4H), 2.57 (s, 3H); ^{13}C NMR (125 MHz, $CDCl_3$) δ 149.2, 139.1, 137.8, 136.3, 133.6, 133.0, 132.2, 131.4, 129.7, 129.1, 128.7, 127.9, 127.8, 127.1, 126.8, 126.5, 125.9, 125.4, 118.4, 112.0, 108.9, 90.4, 88.1, 78.7, 68.5, 54.0, 43.6, 21.3; IR (neat): 2918(bs), 2231(s), 1607, 1519, 1371, 1235, 1172, 1087, 1028, 749, 558 cm^{-1}; HRESIMS Calcd for $[C_{42}H_{33}BrN_2NaO_2S]^+$ (M + Na$^+$) 731.1338, found 731.1305.

_N_-(3-(4-(dibenzylamino)phenyl)prop-2-yn-1-yl)-_N_-((2-methylnaphthalen-1-yl)ethynyl)methanesulfonamide (4e)

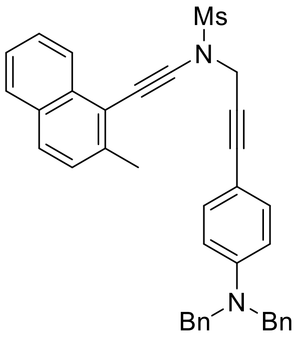

4e

Compound **4e** was prepared in 79% yield (449.3 mg) according to procedure B as a colorless oil (eluent: PE/EtOAc = 3/1). ^{1}H NMR (400 MHz, $CDCl_3$) δ 8.36 – 8.29 (m, 1H), 7.77 – 7.72 (m, 1H), 7.66 (d, *J* = 8.4 Hz, 1H), 7.39 – 7.36 (m, 2H), 7.33 – 7.24 (m, 9H), 7.19 (d, *J* = 6.8 Hz, 4H), 6.64 (d, *J* = 8.8 Hz, 2H), 4.65 (s, 2H), 4.64 (s, 4H), 3.27 (s, 3H), 2.63 (s, 3H); ^{13}C NMR (100 MHz, $CDCl_3$) δ 149.4, 138.8, 137.6, 133.4, 133.3, 131.4, 128.7, 127.9(0), 127.8(5), 127.8, 127.1, 126.8, 126.4, 125.8, 125.4, 118.4, 112.0, 108.8, 90.4, 88.2, 79.4, 68.6, 54.0, 43.5, 38.5, 21.4; IR (neat): 2919(bs), 2226(s), 1602, 1516, 1360, 1261, 1166, 1089, 1026, 750, 559 cm^{-1}; HRESIMS Calcd for $[C_{37}H_{32}N_2NaO_2S]^+$ (M + Na$^+$) 591.2077, found 591.2087.

_N_-(3-(4-(dibenzylamino)phenyl)prop-2-yn-1-yl)-_N_-((2-ethylnaphthalen-1-yl)ethynyl)benzenesulfonamide (4f)

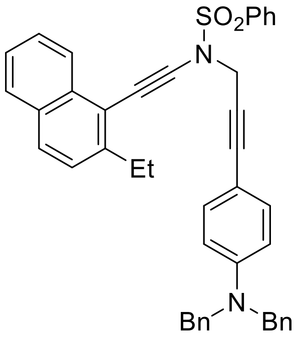

4f

Compound **4f** was prepared in 87% yield (560.9 mg) according to procedure B as a colorless solid (mp 87–88 °C, eluent: PE/EtOAc = 5/1). ^{1}H NMR (500 MHz, $CDCl_3$) δ 8.27 (d, *J* = 8.0 Hz, 1H), 8.07 – 8.00 (m, 2H), 7.71 (d, *J* = 8.0 Hz, 1H), 7.66 (d, *J* = 8.0 Hz, 1H), 7.54 – 7.46 (m, 1H), 7.45 – 7.38 (m, 2H), 7.34 – 7.26 (m, 6H), 7.26 – 7.21 (m, 3H), 7.18 – 7.14 (m, 4H), 7.00 (d, *J* = 9.0 Hz, 2H), 6.56 (d, *J* = 8.5 Hz, 2H), 4.64 (s, 2H), 4.60 (s, 4H), 2.93 (q, *J* = 7.5 Hz, 2H), 1.20 (t, *J* = 7.5 Hz, 3H); ^{13}C NMR (125 MHz, $CDCl_3$) δ 149.1, 144.9, 137.7, 137.3, 133.6, 133.1, 131.5, 128.9, 128.6, 128.1, 128.0, 127.8, 127.0, 126.6, 126.4, 126.3, 126.1, 125.3, 117.8, 111.8, 109.2, 90.0, 87.7, 79.0, 68.1, 53.9, 43.3, 28.4, 15.1; IR (neat): 2925(bs), 2227(s), 1605, 1518, 1362, 1261, 1171, 1089, 1027, 749, 596 cm^{-1}; HRESIMS Calcd for $[C_{43}H_{36}N_2NaO_2S]^+$ (M + Na$^+$) 667.2390, found 667.2363.

_N_-(3-(4-(dibenzylamino)phenyl)prop-2-yn-1-yl)-_N_-((2-propylnaphthalen-1-yl)ethynyl)benzenesulfonamide (4g)

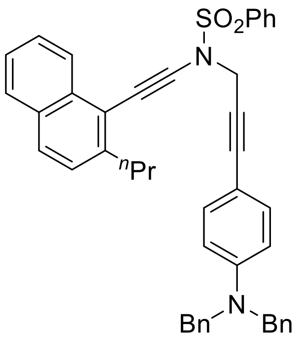

4g

Compound **4g** was prepared in 65% yield (428.3 mg) according to procedure B as a colorless oil (eluent: PE/EtOAc = 5/1). ^{1}H NMR (500 MHz, $CDCl_3$) δ 8.27 (d, *J* = 8.5

Hz, 1H), 8.08 – 8.02 (m, 2H), 7.73 (d, *J* = 8.0 Hz, 1H), 7.67 (d, *J* = 8.5 Hz, 1H), 7.56 – 7.50 (m, 1H), 7.46 – 7.40 (m, 2H), 7.36 – 7.22 (m, 9H), 7.20 – 7.15 (m, 4H), 7.01 (d, *J* = 8.5 Hz, 2H), 6.56 (d, *J* = 9.0 Hz, 2H), 4.66 (s, 2H), 4.62 (s, 4H), 2.90 (t, *J* = 7.5 Hz, 2H), 1.72 – 1.61 (m, 2H), 0.89 (t, *J* = 7.5 Hz, 3H); ^{13}C NMR (125 MHz, $CDCl_3$) δ 149.2, 143.4, 137.7, 137.5, 133.6, 133.1, 131.5, 129.0, 128.7, 128.1, 127.8, 127.7, 127.2, 127.1, 126.6, 126.4, 126.2, 125.3, 118.4, 111.8, 109.2, 89.9, 87.7, 79.0, 68.3, 54.0, 43.3, 37.1, 24.0, 13.8; IR (neat): 2926(bs), 2230(s), 1607, 1519, 1366, 1235, 1171, 1089, 1028, 733, 599 cm^{-1}; HRESIMS Calcd for $[C_{44}H_{38}N_2NaO_2S]^+$ (M + Na$^+$) 681.2546, found 681.2524.

***N*-((2-cyclopentylnaphthalen-1-yl)ethynyl)-*N*-(3-(4-(dibenzylamino)phenyl)prop-2-yn-1-yl)benzenesulfonamide (4h)**

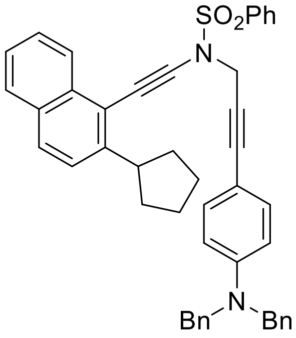

4h

Compound **4h** was prepared in 72% yield (493.1 mg) according to procedure B as a colorless solid (mp 66–67 °C, eluent: PE/EtOAc = 5/1). ^{1}H NMR (500 MHz, $CDCl_3$) δ 8.27 (d, *J* = 8.5 Hz, 1H), 8.08 – 8.01 (m, 2H), 7.75 – 7.67 (m, 2H), 7.57 – 7.52 (m, 1H), 7.49 – 7.41 (m, 2H), 7.39 – 7.28 (m, 6H), 7.27 – 7.22 (m, 3H), 7.20 – 7.15 (m, 4H), 7.02 – 6.97 (m, 2H), 6.56 (d, *J* = 8.5 Hz, 2H), 4.66 (s, 2H), 4.62 (s, 4H), 3.82 – 3.69 (m, 1H), 2.10 – 1.97 (m, 2H), 1.80 – 1.71 (m, 2H), 1.62 – 1.55 (m, 4H); ^{13}C NMR (125 MHz, $CDCl_3$) δ 149.1, 147.2, 137.7, 137.5, 133.6, 133.5, 133.1, 131.5, 129.0, 128.7, 128.2, 128.1, 127.7, 127.1, 126.7, 126.4, 126.3, 125.3, 123.8, 118.1, 111.8, 109.2, 90.1, 87.7, 79.0, 68.3, 54.0, 43.9, 43.3, 34.2, 26.0; IR (neat): 2949(bs), 2229(s), 1607, 1518, 1362, 1234, 1171, 1089, 1028, 734, 596 cm^{-1}; HRESIMS Calcd for $[C_{46}H_{40}N_2NaO_2S]^+$ (M + Na$^+$) 707.2703, found 707.2688.

N-(3-(4-(dibenzylamino)phenyl)prop-2-yn-1-yl)-N-((2-ethyl-7-methylnaphthalen-1-yl)ethynyl)benzenesulfonamide (4i)

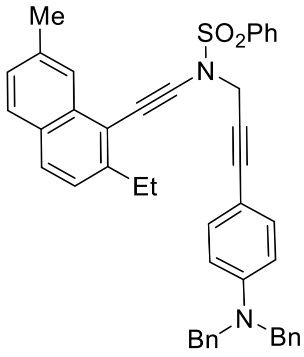

4i

Compound **4i** was prepared in 74% yield (487.6 mg) according to procedure B as a colorless oil (eluent: PE/EtOAc = 5/1). ^{1}H NMR (400 MHz, $CDCl_3$) δ 8.06 – 8.00 (m, 3H), 7.60 (d, J = 8.0 Hz, 2H), 7.50 – 7.45 (m, 1H), 7.40 – 7.35 (m, 2H), 7.30 – 7.27 (m, 3H), 7.23 – 7.18 (m, 4H), 7.17 – 7.10 (m, 5H), 6.97 (d, J = 8.8 Hz, 2H), 6.52 (d, J = 8.8 Hz, 2H), 4.63 (s, 2H), 4.56 (s, 4H), 2.90 (q, J = 7.6 Hz, 2H), 2.27 (s, 3H), 1.18 (t, J = 7.6 Hz, 3H); ^{13}C NMR (100 MHz, $CDCl_3$) δ 149.0, 145.1, 137.6, 137.3, 136.4, 133.9, 133.5, 132.9, 129.7, 128.9, 128.6, 128.0, 127.8, 127.6, 127.5, 127.0, 126.4, 125.5, 125.1, 117.0, 111.7, 109.1, 89.8, 87.5, 78.9, 68.0, 53.9, 43.3, 28.3, 21.7, 15.1; IR (neat): 2927(bs), 2228(s), 1607, 1518, 1363, 1234, 1170, 1086, 1041, 734, 598 cm^{-1}; HRESIMS Calcd for $[C_{44}H_{38}N_2NaO_2S]^+$ (M + Na$^+$) 681.2546, found 681.2524.

N-(3-(4-(dibenzylamino)phenyl)prop-2-yn-1-yl)-N-((2,7-diethylnaphthalen-1-yl)ethynyl)benzenesulfonamide (4j)

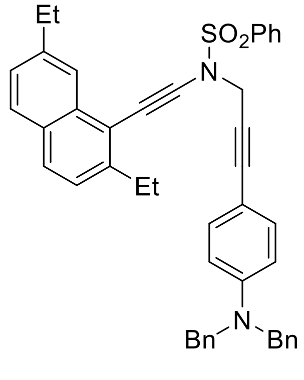

4j

Compound **4j** was prepared in 78% yield (524.8 mg) according to procedure B as a colorless oil (eluent: PE/EtOAc = 5/1). ^{1}H NMR (400 MHz, $CDCl_3$) δ 8.11 (s, 1H), 8.06 – 8.00 (m, 2H), 7.68 – 7.58 (m, 2H), 7.50 – 7.45 (m, 1H), 7.41 – 7.35 (m, 2H), 7.30 – 7.19 (m, 8H), 7.16 – 7.10 (m, 4H), 6.97 (d, *J* = 8.8 Hz, 2H), 6.53 (d, *J* = 8.8 Hz, 2H), 4.64 (s, 2H), 4.57 (s, 4H), 2.90 (q, *J* = 7.6 Hz, 2H), 2.63 (q, *J* = 7.6 Hz, 2H), 1.23 – 1.13 (m, 6H); ^{13}C NMR (100 MHz, $CDCl_3$) δ 149.0, 144.9, 142.8, 137.7, 137.4, 133.8, 133.5, 132.9, 129.9, 128.9, 128.6, 128.0, 127.8, 127.7, 127.0, 126.4, 126.3, 125.6, 123.9, 117.3, 111.7, 109.2, 89.9, 87.5, 78.9, 68.1, 53.9, 43.3, 29.1, 28.4, 15.5, 15.1; IR (neat): 2926(bs), 2230(s), 1607, 1519, 1362, 1234, 1170, 1089, 1028, 734, 597 cm^{-1}; HRESIMS Calcd for $[C_{45}H_{40}N_2NaO_2S]^+$ (M + Na^+) 695.2703, found 695.2675.

***N*-(3-(4-(dibenzylamino)phenyl)prop-2-yn-1-yl)-*N*-((2-ethyl-7-isopropylnaphthalen-1-yl)ethynyl)benzenesulfonamide (4k)**

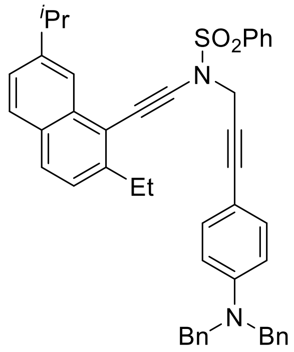

4k

Compound **4k** was prepared in 67% yield (460.2 mg) according to procedure B as a colorless oil (eluent: PE/EtOAc = 5/1). ^{1}H NMR (400 MHz, $CDCl_3$) δ 8.17 (s, 1H), 8.05 (d, *J* = 8.0 Hz, 2H), 7.66 (d, *J* = 8.4 Hz, 1H), 7.61 (d, *J* = 8.4 Hz, 1H), 7.51 – 7.43 (m, 1H), 7.42 – 7.33 (m, 2H), 7.32 – 7.25 (m, 5H), 7.24 – 7.18 (m, 3H), 7.17 – 7.08 (m, 4H), 6.96 (d, *J* = 8.4 Hz, 2H), 6.53 (d, *J* = 8.4 Hz, 2H), 4.64 (s, 2H), 4.57 (s, 4H), 3.01 – 2.83 (m, 3H), 1.23 (d, *J* = 6.8 Hz, 6H), 1.18 (t, *J* = 7.6 Hz, 3H); ^{13}C NMR (100 MHz, $CDCl_3$) δ 149.1, 147.4, 144.6, 137.7, 137.5, 133.7, 133.5, 133.0, 130.1, 128.9, 128.6, 128.0, 127.9, 127.6, 127.0, 126.4, 125.6, 124.9, 122.6, 117.5, 111.7, 109.2, 90.0, 87.5, 78.9, 68.2, 53.9, 43.3, 34.4, 28.4, 23.9, 15.1; IR (neat): 2926(bs),

2231(s), 1606, 1519, 1362, 1233, 1170, 1089, 1043, 733, 596 cm^{-1}; HRESIMS Calcd for $[C_{46}H_{42}N_2NaO_2S]^+$ (M + Na^+) 709.2859, found 709.2853.

***N*-(3-(4-(dibenzylamino)phenyl)prop-2-yn-1-yl)-*N*-((2-ethyl-7-methoxynaphthalen-1-yl)ethynyl)benzenesulfonamide (4l)**

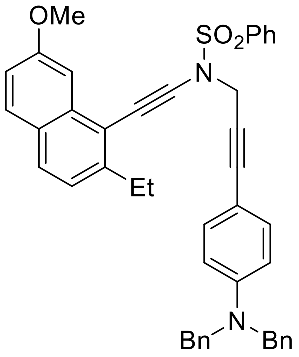

4l

Compound **4l** was prepared in 78% yield (526.4 mg) according to procedure B as a colorless solid (mp 68–69 °C, eluent: PE/EtOAc = 3/1). ^{1}H NMR (400 MHz, $CDCl_3$) δ 7.94 – 7.88 (m, 2H), 7.63 (d, *J* = 2.4 Hz, 1H), 7.51 – 7.43 (m, 2H), 7.38 – 7.33 (m, 1H), 7.29 – 7.23 (m, 2H), 7.19 – 7.13 (m, 4H), 7.13 – 7.06 (m, 2H), 7.05 – 6.99 (m, 5H), 6.94 (dd, *J* = 8.8, 2.4 Hz, 1H), 6.83 (d, *J* = 9.2 Hz, 2H), 6.41 (d, *J* = 8.8 Hz, 2H), 4.50 (s, 2H), 4.46 (s, 4H), 3.68 (s, 3H), 2.75 (q, *J* = 7.6 Hz, 2H), 1.04 (t, *J* = 7.6 Hz, 3H); ^{13}C NMR (100 MHz, $CDCl_3$) δ 158.6, 149.1, 144.8, 137.6, 137.4, 135.0, 133.6, 132.9, 129.3, 128.9, 128.6, 127.9, 127.6, 127.0, 126.8, 126.4, 124.0, 118.3, 116.7, 111.8, 109.1, 104.3, 90.2, 87.4, 78.8, 68.4, 55.3, 53.9, 43.1, 28.4, 15.0; IR (neat): 2926(bs), 2231(s), 1621, 1518, 1362, 1232, 1170, 1089, 1025, 734, 597 cm^{-1}; HRESIMS Calcd for $[C_{44}H_{38}N_2NaO_3S]^+$ (M + Na^+) 697.2495, found 697.2470.

***N*-(3-(4-(dibenzylamino)phenyl)prop-2-yn-1-yl)-*N*-((2-ethyl-7-(trimethylsilyl)naphthalen-1-yl)ethynyl)benzenesulfonamide (4m)**

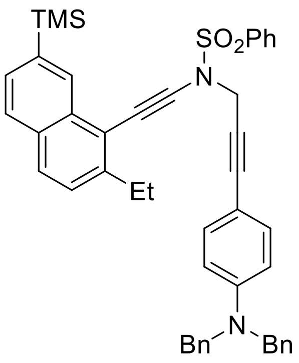

4m

Compound **4m** was prepared in 65% yield (466.1 mg) according to procedure B as a colorless solid (mp 84–85 °C, eluent: PE/EtOAc = 5/1). ^{1}H NMR (400 MHz, $CDCl_3$) δ 8.58 (s, 1H), 8.08 – 8.03 (m, 2H), 7.73 (d, *J* = 8.0 Hz, 1H), 7.64 (d, *J* = 8.4 Hz, 1H), 7.55 (dd, *J* = 8.0, 0.8 Hz, 1H), 7.51 – 7.45 (m, 1H), 7.44 – 7.37 (m, 2H), 7.32 – 7.26 (m, 5H), 7.25 – 7.20 (m, 2H), 7.18 – 7.12 (m, 4H), 6.95 (d, *J* = 8.8 Hz, 2H), 6.53 (d, *J* = 8.8 Hz, 2H), 4.66 (s, 2H), 4.59 (s, 4H), 2.90 (q, *J* = 7.6 Hz, 2H), 1.17 (t, *J* = 7.6 Hz, 3H), 0.32 (s, 9H); ^{13}C NMR (100 MHz, $CDCl_3$) δ 149.1, 144.3, 139.0, 137.7, 137.6, 133.6, 133.0, 132.6, 131.7, 131.6, 129.4, 128.9, 128.7, 128.0, 127.5, 127.0, 126.9, 126.8, 126.4, 118.1, 111.8, 109.2, 90.5, 87.5, 78.8, 68.4, 53.9, 43.3, 28.4, 15.0, -1.1; IR (neat): 2958(bs), 2229(s), 1607, 1519, 1367, 1248, 1171, 1090, 1027, 736, 594 cm^{-1}; HRESIMS Calcd for $[C_{46}H_{44}N_2NaO_2SSi]^+$ (M + Na^+) 739.2785, found 739.2767.

***N*-(3-(4-(dibenzylamino)phenyl)prop-2-yn-1-yl)-*N*-((2-ethyl-6-methylnaphthalen-1-yl)ethynyl)benzenesulfonamide (4n)**

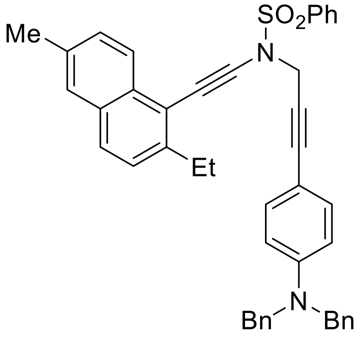

4n

Compound **4n** was prepared in 72% yield (474.4 mg) according to procedure B as a colorless solid (mp 66–67 °C, eluent: PE/EtOAc = 5/1). ^{1}H NMR (400 MHz, $CDCl_3$) δ 8.05 (d, *J* = 8.8 Hz, 1H), 7.92 – 7.86 (m, 2H), 7.43 (d, *J* = 8.4 Hz, 1H), 7.39 – 7.31 (m, 2H), 7.28 – 7.21 (m, 2H), 7.18 – 7.12 (m, 4H), 7.11 – 7.07 (m, 3H), 7.05 – 7.00 (m, 4H), 6.99 – 6.93 (m, 1H), 6.87 (d, *J* = 8.8 Hz, 2H), 6.43 (d, *J* = 9.2 Hz, 2H), 4.49 (s, 2H), 4.45 (s, 4H), 2.77 (q, *J* = 7.6 Hz, 2H), 2.25 (s, 3H), 1.06 (t, *J* = 7.6 Hz, 3H); ^{13}C NMR (100 MHz, $CDCl_3$) δ 149.1, 143.9, 137.6, 137.3, 134.8, 133.5, 133.0, 131.8, 131.6, 128.9, 128.6, 128.0, 127.4, 127.0, 126.8, 126.4, 126.3, 125.9, 117.5, 111.7, 109.1, 89.7, 87.6, 78.9, 68.2, 53.9, 43.3, 28.2, 21.4, 15.1; IR (neat): 2926(bs), 2231(s), 1606, 1518, 1363, 1234, 1170, 1139, 1028, 734, 597 cm^{-1}; HRESIMS Calcd for $[C_{44}H_{38}N_2NaO_2S]^+$ (M + Na^+) 681.2546, found 681.2524.

***N*-(3-(4-(dimethylamino)phenyl)prop-2-yn-1-yl)-*N*-((2-ethylnaphthalen-1-yl)ethynyl)benzenesulfonamide (4o)**

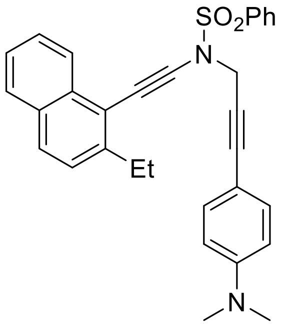

4o

Compound **4o** was prepared in 85% yield (418.7 mg) according to procedure B as a colorless solid (mp 105–106 °C, eluent: PE/EtOAc = 5/1). ^{1}H NMR (500 MHz, $CDCl_3$) δ 8.30 (d, *J* = 8.5 Hz, 1H), 8.12 – 8.01 (m, 2H), 7.72 (d, *J* = 8.0 Hz, 1H), 7.67 (d, *J* = 8.0 Hz, 1H), 7.56 – 7.51 (m, 1H), 7.46 – 7.41 (m, 2H), 7.38 – 7.28 (m, 3H), 7.07 (d, *J* = 9.0 Hz, 2H), 6.50 (d, *J* = 9.0 Hz, 2H), 4.68 (s, 2H), 2.94 (q, *J* = 7.5 Hz, 2H), 2.89 (s, 6H), 1.21 (t, *J* = 7.5 Hz, 3H); ^{13}C NMR (125 MHz, $CDCl_3$) δ 150.2, 144.9, 137.3, 133.6(1), 133.6(0), 132.8, 131.5, 128.9, 128.1, 128.0, 127.8, 126.6, 126.5, 126.1, 125.3, 117.8, 111.4, 108.4, 90.0, 87.9, 78.7, 68.0, 43.4, 39.9, 28.4, 15.1;

IR (neat): 2924(bs), 2229(s), 1607, 1521, 1366, 1227, 1170, 1136, 1042, 737, 590 cm^{-1}; HRESIMS Calcd for $[C_{31}H_{28}N_2NaO_2S]^+$ (M + Na^+) 515.1764, found 515.1752.

_N_-((2-ethylnaphthalen-1-yl)ethynyl)-_N_-(3-(4-(pyrrolidin-1-yl)phenyl)prop-2-yn-1-yl)benzenesulfonamide (4p)

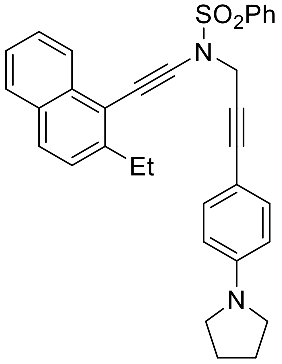

4p

Compound **4p** was prepared in 79% yield (409.8 mg) according to procedure B as a colorless solid (mp 98–99 °C, eluent: PE/EtOAc = 5/1). ^{1}H NMR (500 MHz, $CDCl_3$) δ 8.29 (d, *J* = 8.5 Hz, 1H), 8.09 – 8.02 (m, 2H), 7.75 – 7.71 (m, 1H), 7.67 (d, *J* = 8.5 Hz, 1H), 7.57 – 7.52 (m, 1H), 7.46 – 7.41 (m, 2H), 7.39 – 7.31 (m, 2H), 7.29 (d, *J* = 8.5 Hz, 1H), 7.06 (d, *J* = 8.5 Hz, 2H), 6.35 (d, *J* = 9.0 Hz, 2H), 4.69 (s, 2H), 3.23 – 3.17 (m, 4H), 2.94 (q, *J* = 7.5 Hz, 2H), 1.97 – 1.89 (m, 4H), 1.22 (t, *J* = 7.5 Hz, 3H); ^{13}C NMR (125 MHz, $CDCl_3$) δ 147.7, 145.0, 137.3, 133.6, 133.0, 131.5, 129.0, 128.9, 128.1, 128.0, 127.8, 126.7, 126.5, 126.1, 125.3, 117.9, 111.0, 107.4, 90.1, 88.3, 78.5, 68.0, 47.3, 43.5, 28.4, 25.3, 15.1; IR (neat): 2963(bs), 2233(s), 1607, 1519, 1372, 1264, 1171, 1089, 1044, 737, 603 cm^{-1}; HRESIMS Calcd for $[C_{33}H_{30}N_2NaO_2S]^+$ (M + Na^+) 541.1920, found 541.1909.

_N_-((2-ethylnaphthalen-1-yl)ethynyl)-_N_-(3-(4-methoxyphenyl)prop-2-yn-1-yl)benzenesulfonamide (4q)

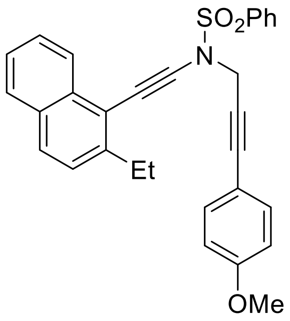

4q

Compound **4q** was prepared in 89% yield (426.8 mg) according to procedure A as a colorless oil (eluent: PE/EtOAc = 5/1). ^{1}H NMR (500 MHz, $CDCl_3$) δ 8.28 (d, *J* = 8.5 Hz, 1H), 8.11 – 8.01 (m, 2H), 7.73 (d, *J* = 8.0 Hz, 1H), 7.69 (d, *J* = 8.5 Hz, 1H), 7.58 – 7.53 (m, 1H), 7.48 – 7.42 (m, 2H), 7.40 – 7.34 (m, 1H), 7.33 – 7.27 (m, 2H), 7.12 (d, *J* = 8.5 Hz, 2H), 6.75 (d, *J* = 9.0 Hz, 2H), 4.68 (s, 2H), 3.74 (s, 3H), 2.94 (q, *J* = 7.5 Hz, 2H), 1.21 (t, *J* = 7.5 Hz, 3H); ^{13}C NMR (125 MHz, $CDCl_3$) δ 159.8, 145.0, 137.3, 133.7, 133.6, 133.2, 131.5, 129.0, 128.1, 127.8, 126.6, 126.5, 126.0, 125.4, 117.7, 113.8, 113.7, 89.9, 86.7, 79.7, 68.0, 55.2, 43.1, 28.4, 15.1; IR (neat): 2923(bs), 2232(s), 1633, 1509, 1367, 1250, 1171, 1088, 1031, 750, 599 cm^{-1}; HRESIMS Calcd for $[C_{30}H_{25}NNaO_3S]^+$ (M + Na^+) 502.1447, found 502.1434.

***N*-((2-ethylnaphthalen-1-yl)ethynyl)-*N*-(3-(4-((triisopropylsilyl)oxy)phenyl)prop-2-yn-1-yl)benzenesulfonamide (4r)**

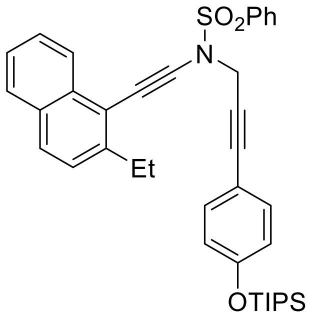

4r

Compound **4r** was prepared in 63% yield (391.8 mg) according to procedure A as a colorless solid (mp 76–77 °C, eluent: PE/EtOAc = 5/1). ^{1}H NMR (400 MHz, $CDCl_3$) δ 8.29 (d, *J* = 8.4 Hz, 1H), 8.09 – 8.03 (m, 2H), 7.72 (d, *J* = 8.0 Hz, 1H), 7.68 (d, *J* = 8.4 Hz, 1H), 7.56 – 7.50 (m, 1H), 7.46 – 7.41 (m, 2H), 7.37 – 7.24 (m, 3H), 7.08 (d, *J*

= 8.8 Hz, 2H), 6.75 (d, *J* = 8.4 Hz, 2H), 4.67 (s, 2H), 2.94 (q, *J* = 7.2 Hz, 2H), 1.25 – 1.17 (m, 6H), 1.08 (d, *J* = 7.2 Hz, 18H); ^{13}C NMR (100 MHz, $CDCl_3$) δ 156.7, 145.0, 137.3, 133.6, 133.2, 131.5, 128.9, 128.1, 127.8, 126.6, 126.5, 126.1, 125.3, 119.8, 117.7, 114.3, 89.9, 86.9, 79.9, 68.1, 43.1, 28.4, 17.8, 15.1, 12.5; IR (neat): 2944(bs), 2232(s), 1601, 1507, 1371, 1279, 1172, 1090, 1027, 745, 589 cm^{-1}; HRESIMS Calcd for $[C_{38}H_{43}NNaO_3SSi]^+$ (M + Na$^+$) 644.2625, found 644.2606.

***N*-(3-(benzo[*d*][1,3]dioxol-5-yl)prop-2-yn-1-yl)-*N*-((2-ethylnaphthalen-1-yl)ethynyl)benzenesulfonamide (4s)**

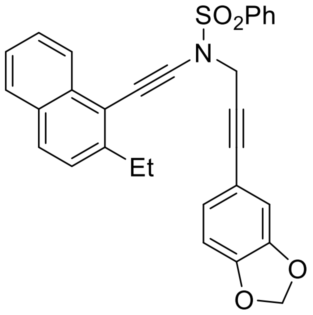

4s

Compound **4s** was prepared in 71% yield (350.4 mg) according to procedure A as a colorless solid (mp 85–86 °C, eluent: PE/EtOAc = 5/1). ^{1}H NMR (400 MHz, $CDCl_3$) δ 8.27 (d, *J* = 8.4 Hz, 1H), 8.10 – 8.04 (m, 2H), 7.75 (d, *J* = 7.6 Hz, 1H), 7.71 (d, *J* = 8.4 Hz, 1H), 7.62 – 7.56 (m, 1H), 7.51 – 7.46 (m, 2H), 7.41 – 7.31 (m, 3H), 6.72 (dd, *J* = 8.0, 1.6 Hz, 1H), 6.66 (d, *J* = 8.0 Hz, 1H), 6.61 (d, *J* = 1.6 Hz, 1H), 5.93 (s, 2H), 4.67 (s, 2H), 2.94 (q, *J* = 7.6 Hz, 2H), 1.22 (t, *J* = 7.6 Hz, 3H); ^{13}C NMR (100 MHz, $CDCl_3$) δ 148.2, 147.2, 145.1, 137.4, 133.8, 133.7, 131.6, 129.0, 128.2, 127.9, 126.7, 126.6, 126.5, 126.0, 125.4, 117.7, 115.0, 111.6, 108.3, 101.3, 99.9, 89.8, 86.6, 79.6, 68.1, 43.1, 28.4, 15.2; IR (neat): 2927(bs), 2231(s), 1504, 1488, 1368, 1248, 1172, 1102, 1038, 739, 588 cm^{-1}; HRESIMS Calcd for $[C_{30}H_{23}NNaO_4S]^+$ (M + Na$^+$) 516.1240, found 516.1260.

1.4 General Procedure for the Formal C–C Bond Insertion

1.4.1 General procedure for the synthesis of pyrrylaldehydes 3:

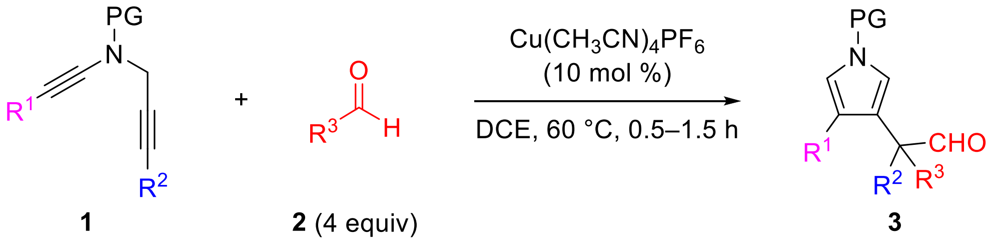

To the solution of *N*-propargyl ynamide **1** (0.2 mmol) and arylaldehyde **2** (0.8 mmol) in DCE (4 mL) was added $Cu(CH_3CN)_4PF_6$ (7.6 mg, 0.02 mmol). The resulting mixture was then stirred at 60 °C for 0.5–1.5 h and the progress of the reaction was monitored by TLC. Upon completion, the resulting mixture was concentrated under reduced pressure and purified by column chromatography on silica gel (eluent: PE/EtOAc) to afford the desired pyrrylaldehyde **3**.

2,2-bis(4-methoxyphenyl)-2-(1-(methylsulfonyl)-4-phenyl-1*H*-pyrrol-3-yl)acetaldehyde (3a)

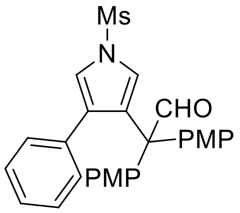

3a

Compound **3a** was prepared in 90% yield (85.6 mg) according to the general procedure as a colorless solid (mp 262–263 °C, eluent: PE/EtOAc = 5/1). ^{1}H NMR (400 MHz, $CDCl_3$) δ 9.89 (s, 1H), 7.15 (d, J = 2.4 Hz, 1H), 7.13 – 7.09 (m, 1H), 7.08 – 7.03 (m, 2H), 7.02 – 6.98 (m, 4H), 6.85 – 6.80 (m, 6H), 6.67 (d, J = 2.8 Hz, 1H), 3.80 (s, 6H), 3.19 (s, 3H); ^{13}C NMR (100 MHz, $CDCl_3$) δ 197.9, 158.8, 134.1, 131.9, 130.7, 130.1, 129.9, 129.4, 127.8, 127.1, 121.6, 120.1, 113.7, 63.2, 55.2, 42.8; IR (neat): 2934(bs), 1723(s), 1600, 1510, 1367, 1259, 1160, 1082, 770, 597 cm^{-1}; HRESIMS Calcd for $[C_{27}H_{25}NNaO_5S]^+$ (M + Na^+) 498.1346, found 498.1317.

2,2-bis(4-methoxyphenyl)-2-(4-phenyl-1-tosyl-1*H*-pyrrol-3-yl)acetaldehyde (3b)

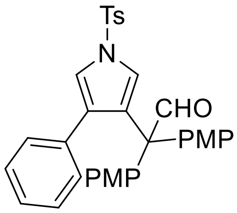

3b

Compound **3b** was prepared in 85% yield (93.8 mg) according to the general procedure as a colorless solid (mp 161–162 °C, eluent: PE/EtOAc = 5/1). ^{1}H NMR (400 MHz, $CDCl_3$) δ 9.82 (s, 1H), 7.73 (d, *J* = 8.0 Hz, 2H), 7.33 (d, *J* = 8.0 Hz, 2H), 7.15 (d, *J* = 2.4 Hz, 1H), 7.11 – 7.06 (m, 1H), 7.05 – 6.99 (m, 2H), 6.94 – 6.89 (m, 4H), 6.81 – 6.77 (m, 4H), 6.76 – 6.72 (m, 2H), 6.70 (d, *J* = 2.4 Hz, 1H), 3.80 (s, 6H), 2.46 (s, 3H); ^{13}C NMR (100 MHz, $CDCl_3$) δ 197.9, 158.8, 145.2, 135.8, 134.2, 132.0, 130.8, 130.2, 130.1, 130.0, 129.4, 127.8, 127.0, 126.9, 122.3, 120.5, 113.6, 63.3, 55.2, 21.7; IR (neat): 2927(bs), 1726(s), 1604, 1509, 1372, 1254, 1173, 1079, 749, 597 cm^{-1}; HRESIMS Calcd for $[C_{33}H_{29}NNaO_5S]^+$ (M + Na^+) 574.1659, found 574.1641.

2,2-bis(4-methoxyphenyl)-2-(1-((4-methoxyphenyl)sulfonyl)-4-phenyl-1*H*-pyrrol-3-yl)acetaldehyde (3c)

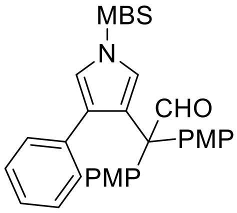

3c

Compound **3c** was prepared in 70% yield (79.5 mg) according to the general procedure as a colorless solid (mp 162–163 °C, eluent: PE/EtOAc = 3/1). ^{1}H NMR (400 MHz, $CDCl_3$) δ 9.82 (s, 1H), 7.83 – 7.75 (m, 2H), 7.15 (d, *J* = 2.4 Hz, 1H), 7.12 – 7.06 (m, 1H), 7.03 (d, *J* = 7.6 Hz, 2H), 6.99 (d, *J* = 9.2 Hz, 2H), 6.95 – 6.89 (m, 4H), 6.81 – 6.78 (m, 4H), 6.76 – 6.73 (m, 2H), 6.70 (d, *J* = 2.4 Hz, 1H), 3.88 (s, 3H), 3.80 (s, 6H); ^{13}C NMR (100 MHz, $CDCl_3$) δ 197.9, 163.9, 158.7, 134.3, 132.0, 130.8,

130.1, 130.0, 129.9, 129.3, 129.2, 127.7, 127.0, 122.1, 120.4, 114.6, 113.6, 63.3, 55.7, 55.2; IR (neat): 2932(bs), 1725(s), 1595, 1509, 1372, 1253, 1166, 1079, 736, 599 cm^{-1}; HRESIMS Calcd for $[C_{33}H_{29}NNaO_6S]^+$ (M + Na^+) 590.1608, found 590.1592.

2,2-bis(4-methoxyphenyl)-2-(4-phenyl-1-(phenylsulfonyl)-1*H*-pyrrol-3-yl)acetaldehyde (3d)

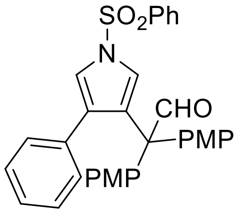

3d

Compound **3d** was prepared in 71% yield (76.3 mg) according to the general procedure as a colorless solid (mp 147–148 °C, eluent: PE/EtOAc = 5/1). ^1H NMR (500 MHz, $CDCl_3$) δ 9.82 (s, 1H), 7.88 – 7.83 (m, 2H), 7.68 – 7.63 (m, 1H), 7.57 – 7.51 (m, 2H), 7.17 (d, *J* = 2.5 Hz, 1H), 7.11 – 7.07 (m, 1H), 7.05 – 7.00 (m, 2H), 6.93 – 6.89 (m, 4H), 6.81 – 6.77 (m, 4H), 6.76 – 6.73 (m, 2H), 6.72 (d, *J* = 2.5 Hz, 1H), 3.80 (s, 6H); ^{13}C NMR (125 MHz, $CDCl_3$) δ 197.8, 158.8, 138.7, 134.1, 134.0, 131.9, 130.8, 130.4, 130.3, 129.4, 129.3, 127.8, 127.0, 126.8, 122.3, 120.5, 113.6, 63.2, 55.2; IR (neat): 2932(bs), 1725(s), 1606, 1508, 1373, 1252, 1119, 1078, 727, 597 cm^{-1}; HRESIMS Calcd for $[C_{32}H_{27}NNaO_5S]^+$ (M + Na^+) 560.1502, found 560.1481.

2-(1-((4-bromophenyl)sulfonyl)-4-phenyl-1*H*-pyrrol-3-yl)-2,2-bis(4-methoxyphenyl)acetaldehyde (3e)

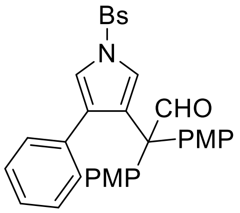

3e

Compound **3e** was prepared in 79% yield (97.4 mg) according to the general procedure as a colorless solid (mp 161–162 °C, eluent: PE/EtOAc = 10/1). ^1H NMR

(400 MHz, $CDCl_3$) δ 9.82 (s, 1H), 7.70 (d, *J* = 2.8 Hz, 4H), 7.14 (d, *J* = 2.8 Hz, 1H), 7.11 – 7.08 (m, 1H), 7.05 – 7.00 (m, 2H), 6.92 – 6.88 (m, 4H), 6.82 – 6.78 (m, 4H), 6.75 – 6.72 (m, 2H), 6.70 (d, *J* = 2.8 Hz, 1H), 3.81 (s, 6H); ^{13}C NMR (100 MHz, $CDCl_3$) δ 197.6, 158.8, 137.6, 133.9, 132.8, 131.7, 130.9, 130.8, 130.7, 129.3, 128.3, 127.8, 127.1, 122.2, 120.5, 113.7, 63.2, 55.2; IR (neat): 2931(bs), 1725(s), 1605, 1509, 1377, 1252, 1183, 1068, 744, 597 cm^{-1}; HRESIMS Calcd for $[C_{32}H_{26}BrNNaO_5S]^+$ (M + Na$^+$) 638.0607, found 638.0587.

2-(4-(4-chlorophenyl)-1-(methylsulfonyl)-1*H*-pyrrol-3-yl)-2,2-bis(4-methoxyphenyl)acetaldehyde (3f)

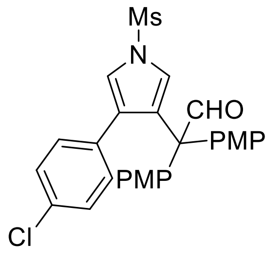

3f

Compound **3f** was prepared in 77% yield (78.5 mg) according to the general procedure as a colorless solid (mp 112–113 °C, eluent: PE/EtOAc = 5/1). ^{1}H NMR (400 MHz, $CDCl_3$) δ 9.89 (s, 1H), 7.13 (d, *J* = 2.8 Hz, 1H), 7.04 – 6.97 (m, 6H), 6.85 – 6.81 (m, 4H), 6.74 – 6.69 (m, 3H), 3.81 (s, 6H), 3.21 (s, 3H); ^{13}C NMR (100 MHz, $CDCl_3$) δ 197.4, 158.9, 133.0, 132.7, 131.5, 130.8, 130.7, 129.8, 128.9, 127.9, 121.6, 120.1, 113.8, 63.1, 55.2, 42.9; IR (neat): 2930(bs), 1724(s), 1606, 1509, 1368, 1252, 1172, 1080, 770, 599 cm^{-1}; HRESIMS Calcd for $[C_{27}H_{24}ClNNaO_5S]^+$ (M + Na$^+$) 532.0956, found 532.0956.

2-(4-(4-bromophenyl)-1-(methylsulfonyl)-1*H*-pyrrol-3-yl)-2,2-bis(4-methoxyphenyl)acetaldehyde (3g)

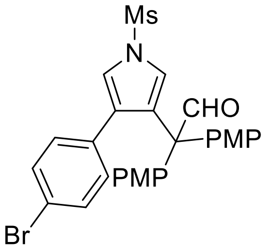

3g

Compound **3g** was prepared in 71% yield (78.7 mg) according to the general procedure as a colorless solid (mp 139–140 °C, eluent: PE/EtOAc = 5/1). ^{1}H NMR (400 MHz, $CDCl_3$) δ 9.89 (s, 1H), 7.17 (d, *J* = 8.4 Hz, 2H), 7.13 (d, *J* = 2.8 Hz, 1H), 6.99 (d, *J* = 8.8 Hz, 4H), 6.83 (d, *J* = 8.8 Hz, 4H), 6.72 (d, *J* = 2.8 Hz, 1H), 6.65 (d, *J* = 8.4 Hz, 2H), 3.81 (s, 6H), 3.21 (s, 3H); ^{13}C NMR (100 MHz, $CDCl_3$) δ 197.4, 158.9, 133.1, 131.4, 131.1, 130.8, 130.7, 129.7, 128.8, 121.6, 121.2, 120.0, 113.8, 63.1, 55.2, 42.9; IR (neat): 2924(bs), 1724(s), 1605, 1508, 1367, 1252, 1173, 1080, 770, 603 cm^{-1}; HRESIMS Calcd for $[C_{27}H_{24}BrNNaO_5S]^+$ (M + Na^+) 576.0451, found 576.0466.

methyl 4-(4-(1,1-bis(4-methoxyphenyl)-2-oxoethyl)-1-(methylsulfonyl)-1*H*-pyrrol-3-yl)benzoate (3h)

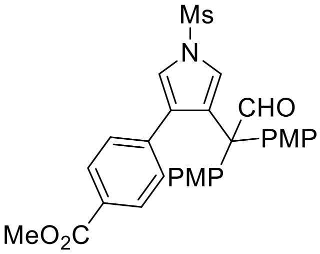

3h

Compound **3h** was prepared in 71% yield (75.8 mg) according to the general procedure as a colorless solid (mp 107–108 °C, eluent: PE/EtOAc = 3/1). ^{1}H NMR (400 MHz, $CDCl_3$) δ 9.90 (s, 1H), 7.11 (d, *J* = 2.4 Hz, 1H), 7.02 – 6.98 (m, 4H), 6.86 – 6.82 (m, 4H), 6.75 – 6.71 (m, 2H), 6.63 – 6.59 (m, 3H), 3.81 (s, 6H), 3.72 (s, 3H), 3.20 (s, 3H); ^{13}C NMR (100 MHz, $CDCl_3$) δ 198.3, 158.8, 158.7, 132.0, 130.8, 130.6, 130.4, 129.5, 126.3, 121.6, 119.9, 113.7, 113.3, 63.2, 55.2, 55.1, 42.8; IR (neat):

2932(bs), 1724(s), 1608, 1510, 1367, 1255, 1171, 1080, 765, 563 cm^{-1}; HRESIMS Calcd for $[C_{29}H_{27}NNaO_7S]^+$ (M + Na^+) 556.1400, found 556.1394.

2,2-bis(4-methoxyphenyl)-2-(1-(methylsulfonyl)-4-(*p*-tolyl)-1*H*-pyrrol-3-yl)acetaldehyde (3i)

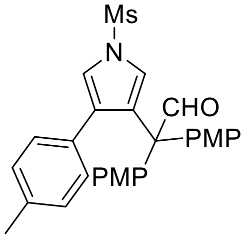

3i

Compound **3i** was prepared in 70% yield (68.5 mg) according to the general procedure as a colorless solid (mp 101–102 °C, eluent: PE/EtOAc = 5/1). ^{1}H NMR (500 MHz, $CDCl_3$) δ 9.90 (s, 1H), 7.13 (d, *J* = 2.5 Hz, 1H), 7.02 – 6.99 (m, 4H), 6.89 (d, *J* = 8.0 Hz, 2H), 6.85 – 6.82 (m, 4H), 6.71 (d, *J* = 8.0 Hz, 2H), 6.60 (d, *J* = 3.0 Hz, 1H), 3.81 (s, 6H), 3.19 (s, 3H), 2.24 (s, 3H); ^{13}C NMR (125 MHz, $CDCl_3$) δ 198.3, 158.8, 136.8, 132.1, 131.1, 130.8, 130.4, 129.8, 129.2, 128.6, 121.7, 120.0, 113.7, 63.2, 55.2, 42.8, 21.0; IR (neat): 2921(bs), 1725(s), 1605, 1508, 1367, 1252, 1172, 1080, 770, 563 cm^{-1}; HRESIMS Calcd for $[C_{28}H_{27}NNaO_5S]^+$ (M + Na^+) 512.1502, found 512.1484.

2,2-bis(4-methoxyphenyl)-2-(4-(4-methoxyphenyl)-1-(methylsulfonyl)-1*H*-pyrrol-3-yl)acetaldehyde (3j)

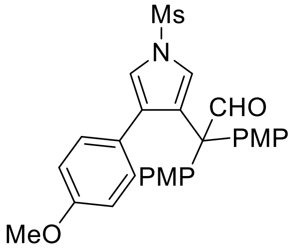

3j

Compound **3j** was prepared in 68% yield (68.8 mg) according to the general procedure as a colorless solid (mp 109–110 °C, eluent: PE/EtOAc = 3/1). ^{1}H NMR

(400 MHz, $CDCl_3$) δ 9.90 (s, 1H), 7.11 (d, J = 2.8 Hz, 1H), 7.02 – 6.98 (m, 4H), 6.86 – 6.82 (m, 4H), 6.75 – 6.71 (m, 2H), 6.63 – 6.59 (m, 3H), 3.81 (s, 6H), 3.72 (s, 3H), 3.19 (s, 3H); ^{13}C NMR (100 MHz, $CDCl_3$) δ 198.3, 158.8, 158.7, 132.0, 130.8, 130.6, 130.4, 129.5, 126.3, 121.6, 119.9, 113.7, 113.3, 63.2, 55.2, 55.1, 42.8; IR (neat): 2920(bs), 1723(s), 1607, 1509, 1367, 1251, 1170, 1081, 769, 562 cm^{-1}; HRESIMS Calcd for $[C_{28}H_{27}NNaO_6S]^+$ (M + Na$^+$) 528.1451, found 528.1433.

2-(4-(3-chlorophenyl)-1-(methylsulfonyl)-1*H*-pyrrol-3-yl)-2,2-bis(4-methoxyphenyl)acetaldehyde (3k)

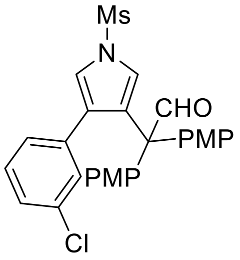

3k

Compound **3k** was prepared in 64% yield (65.3 mg) according to the general procedure as a colorless solid (mp 142–143 °C, eluent: PE/EtOAc = 5/1). ^{1}H NMR (400 MHz, $CDCl_3$) δ 9.89 (s, 1H), 7.14 (d, J = 2.8 Hz, 1H), 7.10 – 7.07 (m, 1H), 7.01 – 6.97 (m, 5H), 6.86 – 6.82 (m, 4H), 6.77 (d, J = 2.4 Hz, 1H), 6.74 – 6.70 (m, 1H), 6.67 – 6.64 (m, 1H), 3.81 (s, 6H), 3.22 (s, 3H); ^{13}C NMR (100 MHz, $CDCl_3$) δ 197.2, 159.0, 136.0, 133.4, 131.2, 130.7, 129.7, 129.6, 128.8, 128.7, 127.7, 127.1, 121.4, 120.2, 113.9, 63.2, 55.3, 42.9; IR (neat): 2930(bs), 1725(s), 1605, 1509, 1368, 1253, 1173, 1079, 770, 600 cm^{-1}; HRESIMS Calcd for $[C_{27}H_{24}ClNNaO_5S]^+$ (M + Na$^+$) 532.0956, found 532.0956.

2,2-bis(4-methoxyphenyl)-2-(1-(methylsulfonyl)-4-(*m*-tolyl)-1*H*-pyrrol-3-yl)acetaldehyde (3l)

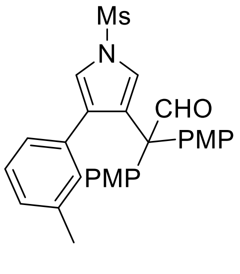

3l

Compound **3l** was prepared in 69% yield (67.6 mg) according to the general procedure as a colorless solid (mp 156–157 °C, eluent: PE/EtOAc = 5/1). ^{1}H NMR (400 MHz, $CDCl_3$) δ 9.88 (s, 1H), 7.14 (d, *J* = 2.8 Hz, 1H), 7.00 (d, *J* = 8.8 Hz, 4H), 6.97 – 6.94 (m, 2H), 6.84 (d, *J* = 9.2 Hz, 4H), 6.68 – 6.64 (m, 2H), 6.54 (s, 1H), 3.81 (s, 6H), 3.20 (s, 3H), 2.11 (s, 3H); ^{13}C NMR (100 MHz, $CDCl_3$) δ 198.1, 158.8, 137.3, 133.9, 132.0, 130.8, 130.3, 130.2, 130.0, 127.8, 127.7, 126.4, 121.5, 120.0, 113.7, 63.3, 55.2, 42.8, 21.1; IR (neat): 2919(bs), 1726(s), 1606, 1509, 1366, 1252, 1173, 1079, 769, 603 cm^{-1}; HRESIMS Calcd for $[C_{28}H_{27}NNaO_5S]^+$ (M + Na$^+$) 512.1502, found 512.1484.

2-(4-cyclopropyl-1-(methylsulfonyl)-1*H*-pyrrol-3-yl)-2,2-bis(4-methoxyphenyl)acetaldehyde (3m)

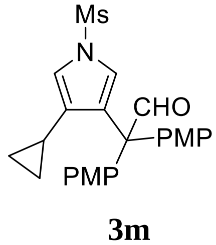

3m

Compound **3m** was prepared in 77% yield (67.7 mg) according to the general procedure as a colorless solid (mp 144–145 °C, eluent: PE/EtOAc = 10/1). ^{1}H NMR (400 MHz, $CDCl_3$) δ 10.27 (s, 1H), 7.06 (d, *J* = 8.8 Hz, 4H), 6.88 (d, *J* = 8.8 Hz, 4H), 6.76 (d, *J* = 2.0 Hz, 1H), 6.45 (d, *J* = 2.4 Hz, 1H), 3.81 (s, 6H), 3.10 (s, 3H), 1.03 – 0.95 (m, 1H), 0.62 – 0.53 (m, 2H), 0.44 – 0.35 (m, 2H); ^{13}C NMR (100 MHz, $CDCl_3$) δ 198.0, 158.8, 131.7, 131.3, 130.7, 121.7, 116.4, 113.8, 63.4, 55.2, 42.5, 8.6, 8.0; IR (neat): 2931(bs), 1724(s), 1606, 1509, 1364, 1252, 1170, 1081, 770, 591 cm^{-1}; HRESIMS Calcd for $[C_{24}H_{25}NNaO_5S]^+$ (M + Na$^+$) 462.1346, found 462.1331.

2-(4-methoxyphenyl)-2-(1-(methylsulfonyl)-4-phenyl-1*H*-pyrrol-3-yl)-2-(*p*-tolyl)acetaldehyde (3n)

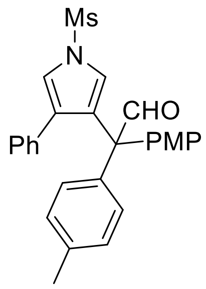

3n

Compound **3n** was prepared in 44% yield (40.4 mg) according to the general procedure as a colorless solid (mp 129–130 °C, eluent: PE/EtOAc = 5/1). ^{1}H NMR (400 MHz, $CDCl_3$) δ 9.91 (s, 1H), 7.15 (d, *J* = 2.8 Hz, 1H), 7.13 – 7.08 (m, 3H), 7.08 – 7.03 (m, 2H), 7.02 – 6.95 (m, 4H), 6.85 – 6.80 (m, 4H), 6.65 (d, *J* = 2.4 Hz, 1H), 3.81 (s, 3H), 3.20 (s, 3H), 2.35 (s, 3H); ^{13}C NMR (100 MHz, $CDCl_3$) δ 198.1, 158.8, 137.3, 137.1, 134.1, 131.8, 130.8, 130.0, 129.5, 129.4, 129.1, 127.8, 127.1, 121.8, 120.1, 113.7, 63.6, 55.3, 42.9, 21.0; IR (neat): 2921(bs), 1726(s), 1605, 1509, 1367, 1252, 1172, 1082, 769, 597 cm^{-1}; HRESIMS Calcd for $[C_{27}H_{25}NNaO_4S]^+$ (M + Na^+) 482.1397, found 482.1377.

2-(4-(benzyloxy)phenyl)-2-(4-methoxyphenyl)-2-(1-(methylsulfonyl)-4-phenyl-1*H*-pyrrol-3-yl)acetaldehyde (3o)

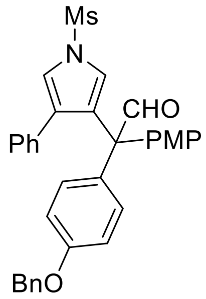

3o

Compound **3o** was prepared in 64% yield (70.6 mg) according to the general procedure as a colorless oil (eluent: PE/EtOAc = 3/1). ^{1}H NMR (400 MHz, $CDCl_3$) δ 9.89 (s, 1H), 7.45 – 7.32 (m, 5H), 7.15 (d, *J* = 2.4 Hz, 1H), 7.12 – 6.98 (m, 7H), 6.90

(d, J = 9.2 Hz, 2H), 6.85 – 6.79 (m, 4H), 6.67 (d, J = 2.8 Hz, 1H), 5.06 (s, 2H), 3.80 (s, 3H), 3.19 (s, 3H); ^{13}C NMR (100 MHz, $CDCl_3$) δ 197.9, 158.8, 158.0, 136.7, 134.1, 132.2, 131.8, 130.8, 130.7, 130.0, 129.9, 129.4, 128.6, 128.0, 127.8, 127.5, 127.1, 121.6, 120.1, 114.6, 113.7, 70.0, 63.2, 55.2, 42.8; IR (neat): 2926(bs), 1723(s), 1605, 1508, 1368, 1251, 1173, 1080, 769, 563 cm^{-1}; HRESIMS Calcd for $[C_{33}H_{29}NNaO_5S]^+$ (M + Na$^+$) 574.1659, found 574.1637.

2-(2-methoxyphenyl)-2-(4-methoxyphenyl)-2-(1-(methylsulfonyl)-4-phenyl-1*H*-pyrrol-3-yl)acetaldehyde (3p)

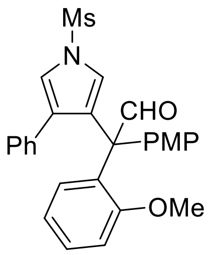

3p

Compound **3p** was prepared in 45% yield (42.8 mg) according to the general procedure as a colorless solid (mp 179–180 °C, eluent: PE/EtOAc = 5/1). ^{1}H NMR (400 MHz, $CDCl_3$) δ 9.75 (s, 1H), 7.31 – 7.26 (m, 1H), 7.14 – 7.10 (m, 4H), 7.08 – 7.03 (m, 2H), 6.89 – 6.80 (m, 6H), 6.75 (dd, J = 7.6, 1.6 Hz, 1H), 6.70 (d, J = 2.8 Hz, 1H), 3.80 (s, 3H), 3.58 (s, 3H), 3.19 (s, 3H); ^{13}C NMR (100 MHz, $CDCl_3$) δ 199.7, 158.7, 156.3, 134.3, 131.7, 131.3, 131.2, 130.7, 129.7, 129.6, 129.5, 129.3, 127.9, 127.1, 121.0, 120.8, 120.2, 113.4, 111.9, 61.5, 55.5, 55.2, 42.8; IR (neat): 2930(bs), 1724(s), 1605, 1509, 1366, 1247, 1172, 1079, 768, 571 cm^{-1}; HRESIMS Calcd for $[C_{27}H_{25}NNaO_5S]^+$ (M + Na$^+$) 498.1346, found 498.1327.

2-(benzo[*d*][1,3]dioxol-5-yl)-2-(4-methoxyphenyl)-2-(1-(methylsulfonyl)-4-phenyl-1*H*-pyrrol-3-yl)acetaldehyde (3q)

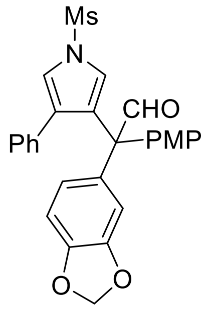

3q

Compound **3q** was prepared in 79% yield (77.3 mg) according to the general procedure as a colorless oil (eluent: PE/EtOAc = 3/1). ^{1}H NMR (400 MHz, CDCl$_3$) δ 9.87 (s, 1H), 7.16 – 7.06 (m, 4H), 7.01 (d, *J* = 8.4 Hz, 2H), 6.88 – 6.80 (m, 4H), 6.74 – 6.67 (m, 2H), 6.58 (s, 1H), 6.53 (d, *J* = 8.0 Hz, 1H), 5.95 (d, *J* = 2.0 Hz, 2H), 3.81 (s, 3H), 3.21 (s, 3H); ^{13}C NMR (100 MHz, CDCl$_3$) δ 197.7, 158.9, 147.7, 146.9, 134.1, 133.7, 131.7, 130.8, 129.9, 129.8, 129.4, 127.8, 127.1, 123.2, 121.6, 120.1, 113.8, 110.3, 107.9, 101.2, 63.5, 55.2, 42.9; IR (neat): 2925(bs), 1725(s), 1607, 1509, 1367, 1245, 1172, 1081, 769, 566 cm^{-1}; HRESIMS Calcd for [$C_{27}H_{23}NNaO_6S$]$^+$ (M + Na$^+$) 512.1138, found 512.1121.

2-(4-methoxyphenyl)-2-(1-(methylsulfonyl)-4-phenyl-1*H*-pyrrol-3-yl)-2-(naphthalen-1-yl)acetaldehyde (3r)

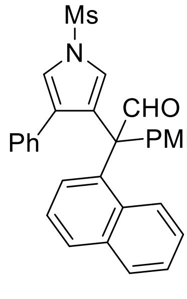

3r

Compound **3r** was prepared in 33% yield (32.7 mg) according to the general procedure as a colorless solid (mp 100–101 °C, eluent: PE/EtOAc = 5/1). ^{1}H NMR (400 MHz, CDCl$_3$) δ 10.04 (s, 1H), 7.83 (d, *J* = 8.0 Hz, 1H), 7.77 (d, *J* = 8.0 Hz, 1H), 7.39 – 7.35 (m, 1H), 7.33 (d, *J* = 8.4 Hz, 1H), 7.29 – 7.25 (m, 1H), 7.24 – 7.19 (m, 1H), 7.14 – 6.96 (m, 7H), 6.83 (d, *J* = 8.8 Hz, 2H), 6.77 – 6.70 (m, 3H), 3.80 (s, 3H), 3.12 (s, 3H); ^{13}C NMR (100 MHz, CDCl$_3$) δ 199.8, 158.9, 137.2, 134.6, 134.2, 131.1,

131.0, 129.9, 129.7, 129.6, 129.6, 129.3, 129.1, 127.7, 127.1, 126.9, 125.4, 125.2, 124.8, 121.4, 120.4, 113.8, 64.2, 55.2, 42.8; IR (neat): 2930(bs), 1723(s), 1605, 1508, 1368, 1252, 1173, 1081, 775, 600 cm^{-1}; HRESIMS Calcd for $[C_{30}H_{25}NNaO_4S]^+$ (M + Na^+) 518.1397, found 518.1379.

2-(4-ethylphenyl)-2-(4-methoxyphenyl)-2-(1-(methylsulfonyl)-4-phenyl-1*H*-pyrrol-3-yl)acetaldehyde (3s)

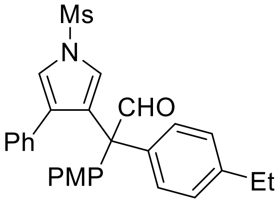

3s

Compound **3s** was prepared in 42% yield (39.9 mg) according to the general procedure using **2b** (10 equiv) at 50 °C as a colorless oil (eluent: PE/EtOAc = 5/1). ^1H NMR (400 MHz, $CDCl_3$) δ 9.91 (s, 1H), 7.15 (d, *J* = 2.4 Hz, 1H), 7.14 – 7.09 (m, 3H), 7.07 – 6.97 (m, 6H), 6.86 – 6.78 (m, 4H), 6.68 (d, *J* = 2.4 Hz, 1H), 3.81 (s, 3H), 3.21 (s, 3H), 2.65 (q, *J* = 7.6 Hz, 2H), 1.24 (t, *J* = 7.6 Hz, 3H); ^{13}C NMR (100 MHz, $CDCl_3$) δ 198.0, 158.8, 143.6, 137.1, 134.1, 131.7, 130.9, 130.1, 130.0, 129.6, 129.5, 127.9, 127.8, 127.1, 121.7, 120.1, 113.7, 63.6, 55.3, 42.9, 28.3, 15.4; IR (neat): 2928(bs), 1725(s), 1606, 1508, 1367, 1253, 1172, 1081, 769, 602 cm^{-1}; HRESIMS Calcd for $[C_{28}H_{27}NNaO_4S]^+$ (M + Na^+) 496.1553, found 496.1539.

2-(4-methoxyphenyl)-2-(1-(methylsulfonyl)-4-phenyl-1*H*-pyrrol-3-yl)-2-(4-((triisopropylsilyl)oxy)phenyl)acetaldehyde (3t)

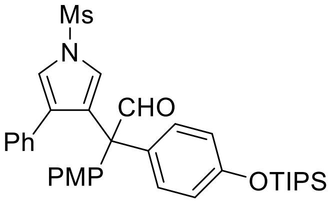

3t

Compound **3t** was prepared in 49% yield (60.6 mg) according to the general procedure as a colorless solid (mp 89–90 °C, eluent: PE/EtOAc = 5/1). ^1H NMR (400

MHz, $CDCl_3$) δ 9.89 (s, 1H), 7.15 (d, *J* = 2.8 Hz, 1H), 7.14 – 7.09 (m, 1H), 7.08 – 7.03 (m, 2H), 7.00 – 6.94 (m, 4H), 6.87 – 6.80 (m, 6H), 6.63 (d, *J* = 2.8 Hz, 1H), 3.80 (s, 3H), 3.20 (s, 3H), 1.28 – 1.25 (m, 3H), 1.11 (d, *J* = 7.2 Hz, 18H); ^{13}C NMR (100 MHz, $CDCl_3$) δ 198.1, 158.8, 155.5, 134.2, 132.3, 132.0, 130.9, 130.6, 130.3, 129.9, 129.4, 127.8, 127.1, 121.8, 120.1, 120.0, 113.6, 63.3, 55.2, 42.9, 17.9, 12.6; IR (neat): 2945(bs), 1726(s), 1604, 1507, 1370, 1254, 1174, 1081, 770, 570 cm^{-1}; HRESIMS Calcd for $[C_{35}H_{43}NNaO_5SSi]^+$ (M + Na^+) 640.2523, found 640.2493.

2-(4-(dimethylamino)phenyl)-2-(4-methoxyphenyl)-2-(1-(methylsulfonyl)-4-phenyl-1*H*-pyrrol-3-yl)acetaldehyde (3u)

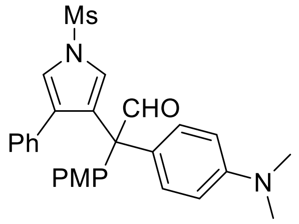

3u

Compound **3u** was prepared in 60% yield (58.6 mg) according to the general procedure as a colorless solid (mp 170–171 °C, eluent: PE/EtOAc = 3/1). ^1H NMR (400 MHz, $CDCl_3$) δ 9.88 (s, 1H), 7.15 (d, *J* = 2.8 Hz, 1H), 7.12 – 7.04 (m, 3H), 7.02 (d, *J* = 8.8 Hz, 2H), 6.93 (d, *J* = 8.8 Hz, 2H), 6.87 – 6.79 (m, 4H), 6.69 – 6.61 (m, 3H), 3.79 (s, 3H), 3.18 (s, 3H), 2.95 (s, 6H); ^{13}C NMR (100 MHz, $CDCl_3$) δ 198.0, 158.6, 149.6, 134.3, 132.3, 130.7, 130.5, 130.3, 130.1, 129.4, 127.8, 126.9, 121.7, 119.9, 113.6, 112.1, 63.1, 55.2, 42.8, 40.3; IR (neat): 2922(bs), 1722(s), 1607, 1510, 1365, 1252, 1172, 1082, 770, 594 cm^{-1}; HRESIMS Calcd for $[C_{28}H_{28}N_2NaO_4S]^+$ (M + Na^+) 511.1662, found 511.1637.

2-(3,4-dimethoxyphenyl)-2-(4-methoxyphenyl)-2-(1-(methylsulfonyl)-4-phenyl-1*H*-pyrrol-3-yl)acetaldehyde (3v)

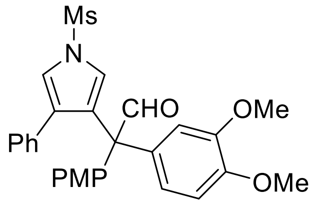

3v

Compound **3v** was prepared in 80% yield (80.9 mg) according to the general procedure as a colorless solid (mp 88–89 °C, eluent: PE/EtOAc = 3/1). ^{1}H NMR (400 MHz, $CDCl_3$) δ 9.91 (s, 1H), 7.16 (d, *J* = 2.4 Hz, 1H), 7.14 – 7.01 (m, 5H), 6.87 – 6.78 (m, 5H), 6.70 (d, *J* = 2.8 Hz, 1H), 6.64 – 6.59 (m, 2H), 3.88 (s, 3H), 3.81 (s, 3H), 3.70 (s, 3H), 3.20 (s, 3H); ^{13}C NMR (100 MHz, $CDCl_3$) δ 197.8, 158.8, 148.5, 148.3, 134.1, 132.2, 131.8, 130.8, 129.9, 129.8, 129.3, 127.8, 127.0, 122.3, 121.6, 120.0, 113.6, 112.9, 110.7, 63.4, 55.8, 55.2, 42.8; IR (neat): 2932(bs), 1724(s), 1604, 1510, 1367, 1254, 1172, 1083, 769, 562 cm^{-1}; HRESIMS Calcd for $[C_{28}H_{27}NNaO_6S]^+$ (M + Na^+) 528.1451, found 528.1428.

2-(furan-2-yl)-2-(4-methoxyphenyl)-2-(1-(methylsulfonyl)-4-phenyl-1*H*-pyrrol-3-yl)acetaldehyde (3w)

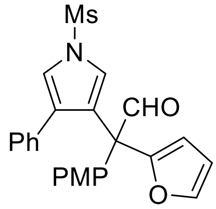

3w

Compound **3w** was prepared in 54% yield (47.0 mg) according to the general procedure as a colorless oil (eluent: PE/EtOAc = 10/1). ^{1}H NMR (400 MHz, $CDCl_3$) δ 9.82 (s, 1H), 7.43 (d, *J* = 0.8 Hz, 1H), 7.18 – 7.08 (m, 4H), 7.00 (d, *J* = 8.8 Hz, 2H), 6.92 – 6.81 (m, 4H), 6.74 (d, *J* = 2.4 Hz, 1H), 6.37 – 6.26 (m, 1H), 6.02 (d, *J* = 3.2 Hz, 1H), 3.80 (s, 3H), 3.21 (s, 3H); ^{13}C NMR (100 MHz, $CDCl_3$) δ 195.0, 159.2, 152.6, 142.6, 133.7, 130.1, 129.8, 129.6, 129.3, 127.8, 127.2, 121.4, 119.8, 114.0, 110.8, 110.4, 60.4, 55.3, 42.9; IR (neat): 2922(bs), 1730(s), 1606, 1509, 1366, 1253, 1172,

1080, 770, 558 cm^{-1}; HRESIMS Calcd for $[C_{24}H_{21}NNaO_5S]^+$ (M + Na^+) 458.1033, found 458.1045.

2-(4-methoxyphenyl)-2-(1-methyl-1*H*-indol-3-yl)-2-(1-(methylsulfonyl)-4-phenyl-1*H*-pyrrol-3-yl)acetaldehyde (3x)

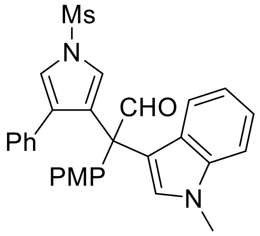

3x

Compound **3x** was prepared in 44% yield (43.9 mg) according to the general procedure as a colorless solid (mp 226–227 °C, eluent: PE/EtOAc = 10/1). ^{1}H NMR (400 MHz, $CDCl_3$) δ 9.92 (s, 1H), 7.25 (s, 1H), 7.23 – 7.17 (m, 3H), 7.12 – 7.06 (m, 3H), 7.03 – 6.95 (m, 3H), 6.90 (d, *J* = 2.8 Hz, 1H), 6.86 (d, *J* = 8.8 Hz, 2H), 6.71 (d, *J* = 7.2 Hz, 2H), 6.39 (s, 1H), 3.83 (s, 3H), 3.62 (s, 3H), 3.15 (s, 3H); ^{13}C NMR (100 MHz, $CDCl_3$) δ 196.1, 158.8, 137.6, 134.2, 131.3, 130.3, 130.2, 129.5, 128.5, 127.4, 126.8, 126.4, 121.8, 121.5, 121.3, 119.9, 119.3, 113.8, 113.4, 109.9, 109.6, 59.2, 55.3, 42.7, 32.7; IR (neat): 2926(bs), 1723(s), 1605, 1508, 1366, 1250, 1172, 1082, 769, 567 cm^{-1}; HRESIMS Calcd for $[C_{29}H_{26}N_2NaO_4S]^+$ (M + Na^+) 521.1505, found 521.1483.

2-(3,4-dimethoxyphenyl)-2-(4-(2,6-dimethylphenyl)-1-(methylsulfonyl)-1*H*-pyrrol -3-yl)-2-(4-methoxyphenyl)acetaldehyde (3y)

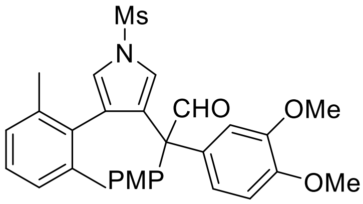

3y

Compound **3y** was prepared in 13% yield (13.9 mg) according to the general procedure at 30 °C for 10 min as a colorless solid (mp 92–93 °C, eluent: PE/EtOAc =

3/1). ^{1}H NMR (400 MHz, $CDCl_3$) δ 9.45 (s, 1H), 7.04 (d, *J* = 2.4 Hz, 1H), 7.02 (d, *J* = 7.6 Hz, 1H), 6.94 – 6.89 (m, 3H), 6.89 – 6.84 (m, 2H), 6.79 – 6.75 (m, 2H), 6.73 (d, *J* = 8.4 Hz, 1H), 6.53 (dd, *J* = 8.4, 2.0 Hz, 1H), 6.49 (d, *J* = 2.4 Hz, 1H), 3.86 (s, 3H), 3.79 (s, 3H), 3.67 (s, 3H), 3.20 (s, 3H), 1.83 (s, 3H), 1.79 (s, 3H); ^{13}C NMR (100 MHz, $CDCl_3$) δ 196.0, 158.8, 148.3, 148.2, 137.6, 137.5, 133.4, 131.3, 131.1(2), 131.1(0), 129.0, 127.8, 127.2, 127.1, 127.0, 122.5, 119.0, 113.3, 113.2, 110.5, 64.5, 55.8, 55.6, 55.2, 42.7, 21.0, 20.9; IR (neat): 2935(bs), 1720(s), 1610, 1505, 1366, 1250, 1171, 1085, 774, 565 cm^{-1}; HRESIMS Calcd for $[C_{30}H_{31}NNaO_6S]^+$ (M + Na^+) 556.1764, found 556.1775.

3-(2,5-bis(3,4-dimethoxyphenyl)-4-(4-methoxyphenyl)-1,3-dioxolan-4-yl)-4-(2,6-dimethylphenyl)-1-(methylsulfonyl)-1*H*-pyrrole (3y′)

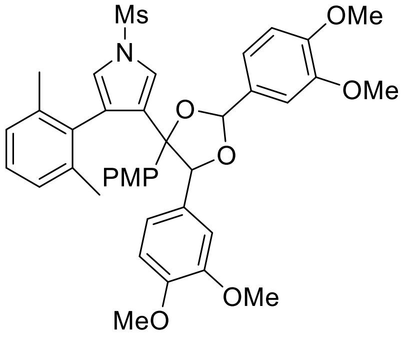

3y′

Compound **3y′** was prepared in 41% yield (57.4 mg) according to the general procedure at 30 °C for 10 min as a colorless solid (mp 82–83 °C, eluent: PE/EtOAc = 1/1). ^{1}H NMR (400 MHz, $CDCl_3$) δ 7.17 (d, *J* = 8.8 Hz, 2H), 7.07 – 7.02 (m, 2H), 6.95 – 6.92 (m, 2H), 6.90 – 6.78 (m, 5H), 6.72 (d, *J* = 8.8 Hz, 2H), 6.54 (d, *J* = 2.4 Hz, 1H), 6.50 (d, *J* = 2.4 Hz, 1H), 5.93 (s, 1H), 5.55 (s, 1H), 3.91 (s, 3H), 3.88 (s, 3H), 3.87 (s, 3H), 3.77 (s, 3H), 3.61 (s, 3H), 2.79 (s, 3H), 1.78 (s, 3H), 1.36 (s, 3H); ^{13}C NMR (100 MHz, $CDCl_3$) δ 158.5, 149.3, 149.2, 148.6(1), 148.6(0), 138.1, 137.5, 134.7, 133.6, 131.5, 129.6, 128.6, 127.3, 127.0, 126.8, 126.3(1), 126.3(0), 121.7, 119.5, 118.5, 117.7, 113.4, 112.5, 110.5, 110.3, 109.3, 101.5, 89.7, 86.6, 56.0, 55.9, 55.8, 55.5, 55.2, 42.1, 21.3, 20.3; IR (neat): 2921(bs), 1607, 1511, 1371, 1172, 1085,

766, 526 cm^{-1}; HRESIMS Calcd for $[C_{39}H_{41}NNaO_9S]^+$ (M + Na^+) 722.2394, found 722.2380.

1.4.2 General procedure for the synthesis of axially chiral naphthylpyrroles 5

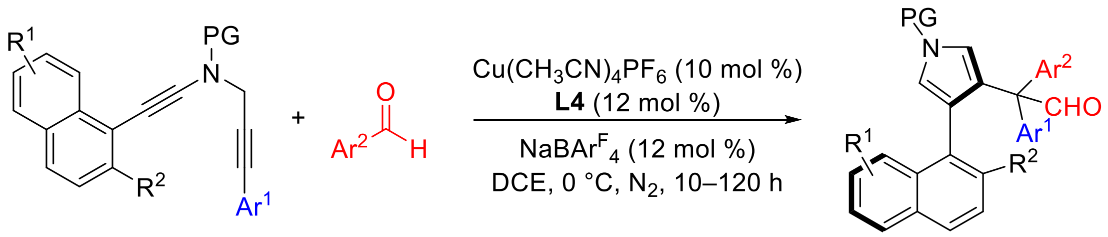

4 **2** (2 equiv) **5**

To a dry 10 mL Schlenk tube charged with a stir bar were added $Cu(CH_3CN)_4PF_6$ (3.8 mg, 0.01 mmol), **L4** (7.5 mg, 0.012 mmol), $NaBAr^F{}_4$ (10.6 mg, 0.012 mmol) and DCE (1 mL) sequentially under N_2 atmosphere. The solution was stirred at room temperature for 2 h. After cooling to 0 °C, the solution of *N*-propargyl ynamide **4** (0.1 mmol) and arylaldehyde **2** (0.2 mmol) in DCE (1 mL) was added into the reaction dropwise. The resulting mixture was stirred at 0 °C for 10–120 h and the progress of the reaction was monitored by TLC. Upon completion, the resulting mixture was concentrated under reduced pressure and purified by column chromatography on silica gel (eluent: PE/EtOAc) to afford the desired axially chiral naphthylpyrrole **5**.

(*R*)-2,2-bis(4-(dibenzylamino)phenyl)-2-(4-(2-methylnaphthalen-1-yl)-1-(phenylsulfonyl)-1*H*-pyrrol-3-yl)acetaldehyde (5a)

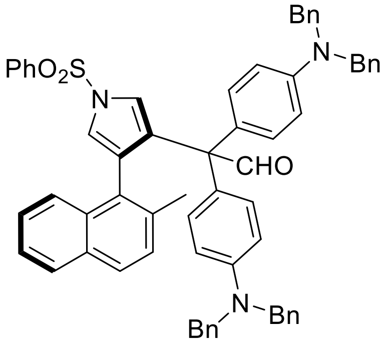

5a

Compound **5a** was prepared in 88% yield (82.0 mg) according to the general procedure as a colorless solid (mp 98–99 °C, eluent: PE/EtOAc = 10/1). $[\alpha]_D^{20}$ =

-29.2° (c = 1.0, $CHCl_3$). 92% ee (determined by HPLC: Chiralpak IE Column, 50/50 *i*-PrOH/hexane, 1.0 mL/min, 254 nm; TR = 16.71 min (major), 42.59 min (minor)). ^{1}H NMR (400 MHz, $CDCl_3$) δ 9.18 (s, 1H), 7.84 – 7.80 (m, 2H), 7.63 (d, *J* = 8.4 Hz, 1H), 7.54 (d, *J* = 8.4 Hz, 1H), 7.51 – 7.46 (m, 1H), 7.38 – 7.31 (m, 10H), 7.29 – 7.27 (m, 1H), 7.25 – 7.19 (m, 13H), 7.05 (d, *J* = 8.4 Hz, 1H), 6.95 (d, *J* = 2.4 Hz, 1H), 6.92 – 6.86 (m, 2H), 6.57 (d, *J* = 8.8 Hz, 2H), 6.47 (d, *J* = 8.8 Hz, 2H), 6.39 – 6.33 (m, 4H), 4.57 (s, 4H), 4.54 (s, 4H), 1.72 (s, 3H); ^{13}C NMR (100 MHz, $CDCl_3$) δ 195.7, 147.9, 147.8, 138.8, 138.5, 135.3, 133.7, 133.4, 131.5, 131.4, 130.7, 130.5, 129.8, 129.1, 128.6, 128.5, 128.0, 127.7, 127.5, 127.2, 126.9, 126.8, 126.7, 126.6, 126.5, 126.4, 126.3, 125.1, 124.4, 123.6, 121.1, 111.6, 63.5, 54.2, 20.7; IR (neat): 2922(bs), 1723(s), 1608, 1515, 1373, 1235, 1183, 1069, 728, 598 cm^{-1}; HRESIMS Calcd for $[C_{63}H_{53}N_3NaO_3S]^+$ (M + Na$^+$) 954.3700, found 954.3677.

(*R*)-2,2-bis(4-(dibenzylamino)phenyl)-2-(4-(2-methylnaphthalen-1-yl)-1-tosyl-1*H*-pyrrol-3-yl)acetaldehyde (5b)

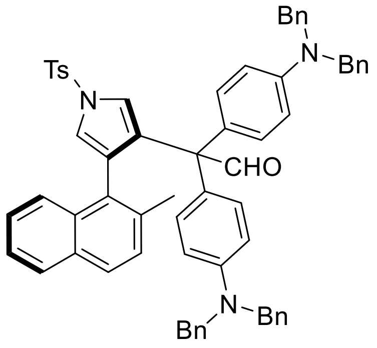

5b

Compound **5b** was prepared in 79% yield (74.8 mg) according to the general procedure as a colorless solid (mp 95–96 °C, eluent: PE/EtOAc = 10/1). $[\alpha]_D^{20}$ = -23.4° (c = 1.0, $CHCl_3$). 92% ee (determined by HPLC: Chiralpak IE Column, 50/50 *i*-PrOH/hexane, 1.0 mL/min, 254 nm; TR = 19.00 min (major), 45.92 min (minor)). ^{1}H NMR (400 MHz, $CDCl_3$) δ 9.18 (s, 1H), 7.70 (d, *J* = 8.4 Hz, 2H), 7.63 (d, *J* = 8.0 Hz, 1H), 7.54 (d, *J* = 8.4 Hz, 1H), 7.35 – 7.30 (m, 8H), 7.25 – 7.15 (m, 16H), 7.05 (d, *J* = 8.4 Hz, 1H), 6.95 – 6.90 (m, 3H), 6.59 (d, *J* = 8.8 Hz, 2H), 6.50 (d, *J* = 8.4 Hz, 2H), 6.39 – 6.33 (m, 4H), 4.57 (s, 4H), 4.53 (s, 4H), 2.32 (s, 3H), 1.75 (s, 3H); ^{13}C

NMR (125 MHz, $CDCl_3$) δ 195.9, 148.0, 147.9, 144.8, 138.5, 135.9, 135.4, 133.4, 131.4, 131.2, 130.8, 130.6, 129.9, 129.7, 128.6, 128.5, 128.0, 127.7, 127.2, 127.1, 126.9, 126.8, 126.7, 126.6, 126.5(8), 126.5(3), 126.4, 126.3, 125.1, 124.4, 123.5, 121.0, 111.5, 111.4, 63.6 54.1, 54.0, 21.5, 20.8; IR (neat): 2923(bs), 1723(s), 1608, 1515, 1373, 1235, 1173, 1071, 732, 595 cm^{-1}; HRESIMS Calcd for $[C_{64}H_{55}N_3NaO_3S]^+$ (M + Na^+) 968.3856, found 968.3821.

(*R*)-2,2-bis(4-(dibenzylamino)phenyl)-2-(1-((4-methoxyphenyl)sulfonyl)-4-(2-methylnaphthalen-1-yl)-1*H*-pyrrol-3-yl)acetaldehyde (5c)

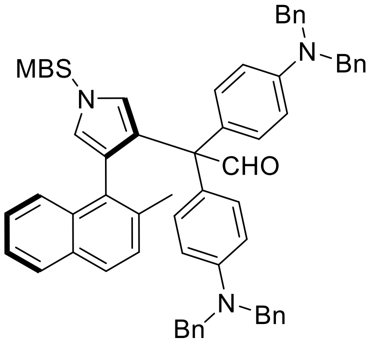

5c

Compound **5c** was prepared in 80% yield (77.0 mg) according to the general procedure as a colorless solid (mp 108–109 °C, eluent: PE/EtOAc = 5/1). $[\alpha]_D^{20}$ = -41.4° (c = 1.0, $CHCl_3$). 93% ee (determined by HPLC: Chiralpak IG Column, 50/50 *i*-PrOH/hexane, 1.0 mL/min, 254 nm; TR = 25.93 min (major), 35.21 min (minor)). ^{1}H NMR (500 MHz, $CDCl_3$) δ 9.18 (s, 1H), 7.77 – 7.74 (m, 2H), 7.63 (d, *J* = 8.0 Hz, 1H), 7.54 (d, *J* = 8.0 Hz, 1H), 7.35 – 7.31 (m, 9H), 7.26 – 7.23 (m, 3H), 7.22 – 7.19 (m, 10H), 7.06 (d, *J* = 8.5 Hz, 1H), 6.94 – 6.92 (m, 3H), 6.84 (d, *J* = 9.0 Hz, 2H), 6.60 (d, *J* = 9.0 Hz, 2H), 6.50 (d, *J* = 9.0 Hz, 2H), 6.39 – 6.36 (m, 4H), 4.57 (s, 2H), 4.56 (s, 2H), 4.54 (s, 2H), 4.53 (s, 2H), 3.73 (s, 3H), 1.75 (s, 3H); ^{13}C NMR (125 MHz, $CDCl_3$) δ 196.0, 163.7, 147.9(4), 147.9(3), 138.5, 135.4, 133.4, 131.4, 131.2, 130.8, 130.6, 130.3, 130.0, 129.1, 128.6, 128.5, 128.0, 127.7, 127.2, 127.1, 126.9, 126.8, 126.6(3), 126.6(0), 126.5, 126.4, 125.1, 124.4, 123.5, 121.0, 114.3, 111.5, 111.4, 63.6, 55.7, 54.1, 20.8; IR (neat): 2921(bs), 1722(s), 1607, 1514, 1361, 1263, 1166, 1070,

730, 595 cm^{-1}; HRESIMS Calcd for $[C_{64}H_{55}N_3NaO_4S]^+$ (M + Na^+) 984.3805, found 984.3843.

(*R*)-2-(1-((4-bromophenyl)sulfonyl)-4-(2-methylnaphthalen-1-yl)-1*H*-pyrrol-3-yl)-2,2-bis(4-(dibenzylamino)phenyl)acetaldehyde (5d)

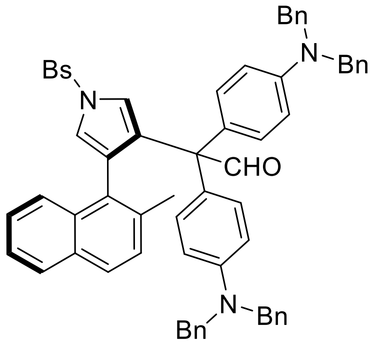

5d

Compound **5d** was prepared in 70% yield (70.8 mg) according to the general procedure as a colorless solid (mp 88–89 °C, eluent: PE/EtOAc = 10/1). $[\alpha]_D^{20}$ = -29.1° (c = 1.0, $CHCl_3$). 92% ee (determined by HPLC: Chiralpak IE Column, 50/50 *i*-PrOH/hexane, 1.0 mL/min, 254 nm; TR = 15.41 min (major), 31.94 min (minor)). ^1H NMR (500 MHz, $CDCl_3$) δ 9.18 (s, 1H), 7.68 – 7.65 (m, 2H), 7.63 (d, *J* = 8.5 Hz, 1H), 7.55 – 7.50 (m, 3H), 7.36 – 7.31 (m, 9H), 7.28 – 7.26 (m, 2H), 7.24 – 7.18 (m, 11H), 7.04 (d, *J* = 8.5 Hz, 1H), 6.96 – 6.91 (m, 2H), 6.84 (d, *J* = 8.0 Hz, 1H), 6.57 (d, *J* = 8.5 Hz, 2H), 6.47 (d, *J* = 8.5 Hz, 2H), 6.39 – 6.34 (m, 4H), 4.58 (s, 2H), 4.57 (s, 2H), 4.54 (s, 2H), 4.53 (s, 2H), 1.74 (s, 3H); ^{13}C NMR (125 MHz, $CDCl_3$) δ 195.5, 147.9(7), 147.9(6), 138.5, 137.7, 135.3, 133.3, 132.5, 131.7, 131.4, 130.7, 130.5, 129.6, 128.9, 128.6, 128.5, 128.2, 128.0(3), 128.0(1), 127.8, 127.2, 127.0, 126.9, 126.7, 126.6, 126.2, 126.1, 125.2, 124.4, 123.6, 121.0, 111.6, 111.5, 63.5, 54.2, 54.1, 20.8; IR (neat): 2920(bs), 1722(s), 1608, 1515, 1390, 1234, 1184, 1067, 744, 597 cm^{-1}; HRESIMS Calcd for $[C_{63}H_{52}BrN_3NaO_3S]^+$ (M + Na^+) 1032.2805, found 1032.2847.

(*R*)-2,2-bis(4-(dibenzylamino)phenyl)-2-(4-(2-methylnaphthalen-1-yl)-1-(methylsulfonyl)-1*H*-pyrrol-3-yl)acetaldehyde (5e)

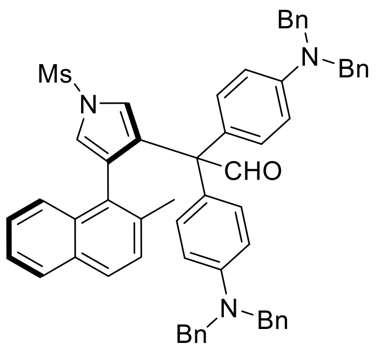

5e

Compound **5e** was prepared in 89% yield (77.0 mg) according to the general procedure as a colorless oil (eluent: PE/EtOAc = 5/1). $[\alpha]_D^{20}$ = -53.2° (c = 1.0, $CHCl_3$). 86% ee (determined by HPLC: Chiralpak IE Column, 50/50 *i*-PrOH/hexane, 1.0 mL/min, 254 nm; TR = 9.29 min (major), 14.62 min (minor)). ^{1}H NMR (400 MHz, $CDCl_3$) δ 9.29 (s, 1H), 7.64 (d, *J* = 8.0 Hz, 1H), 7.55 (d, *J* = 8.4 Hz, 1H), 7.38 (d, *J* = 2.4 Hz, 1H), 7.35 – 7.29 (m, 8H), 7.26 – 7.17 (m, 14H), 7.07 (d, *J* = 8.4 Hz, 1H), 7.03 – 6.98 (m, 1H), 6.93 (d, *J* = 2.8 Hz, 1H), 6.73 (d, *J* = 8.8 Hz, 2H), 6.60 (d, *J* = 9.2 Hz, 2H), 6.40 (d, *J* = 8.8 Hz, 4H), 4.56 (s, 4H), 4.52 (s, 4H), 3.21 (s, 3H), 1.87 (s, 3H); ^{13}C NMR (100 MHz, $CDCl_3$) δ 195.5, 148.0, 138.5, 135.5, 133.6, 131.4, 130.8, 130.6, 129.9, 129.5, 128.5(8), 128.5(5), 128.1, 127.7, 127.2, 126.8(9), 126.8(6), 126.6(3), 126.6(0), 126.5(1), 126.4(6), 126.2, 126.1, 125.1, 124.4, 122.2, 119.9, 111.6, 63.5, 54.1, 42.8, 21.1; IR (neat): 2923(bs), 1724(s), 1610, 1516, 1384, 1236, 1176, 1070, 739, 599 cm^{-1}; HRESIMS Calcd for $[C_{58}H_{51}N_3NaO_3S]^+$ (M + Na^+) 892.3543, found 892.3553.

(*R*)-2,2-bis(4-(dibenzylamino)phenyl)-2-(4-(2-ethylnaphthalen-1-yl)-1-(phenylsulfonyl)-1*H*-pyrrol-3-yl)acetaldehyde (5f)

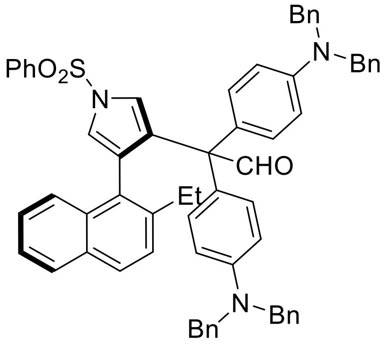

5f

Compound **5f** was prepared in 81% yield (76.6 mg) according to the general procedure as a colorless solid (mp 106–107 °C, eluent: PE/EtOAc = 10/1). $[\alpha]_D^{20}$ = -47.0° (c = 1.0, $CHCl_3$). 94% ee (determined by HPLC: Chiralpak IE Column, 50/50 *i*-PrOH/hexane, 1.0 mL/min, 254 nm; TR = 12.22 min (major), 29.87 min (minor)). ^{1}H NMR (500 MHz, $CDCl_3$) δ 9.15 (s, 1H), 7.83 – 7.77 (m, 2H), 7.62 (d, *J* = 8.0 Hz, 1H), 7.59 (d, *J* = 8.5 Hz, 1H), 7.49 – 7.45 (m, 1H), 7.36 – 7.31 (m, 10H), 7.25 – 7.19 (m, 14H), 7.10 (d, *J* = 8.5 Hz, 1H), 6.97 (d, *J* = 2.5 Hz, 1H), 6.94 – 6.88 (m, 2H), 6.57 (d, *J* = 9.0 Hz, 2H), 6.46 (d, *J* = 9.0 Hz, 2H), 6.38 – 6.33 (m, 4H), 4.57 (s, 4H), 4.54 (s, 4H), 2.02 – 1.91 (m, 2H), 0.81 (t, *J* = 7.5 Hz, 3H); ^{13}C NMR (125 MHz, $CDCl_3$) δ 195.7, 147.8(4), 147.8(3), 141.2, 138.8, 138.5, 133.7, 133.4, 131.6, 131.4, 130.7, 130.5, 129.1, 128.8, 128.6, 128.5, 128.1, 127.2, 127.0, 126.9, 126.8, 126.6, 126.5, 126.4(9), 126.4(3), 126.3, 126.2, 125.1, 124.5, 123.5, 121.3, 111.6, 111.5, 63.5, 54.2(3), 54.2(0), 27.0, 15.6; IR (neat): 2922(bs), 1722(s), 1608, 1515, 1374, 1235, 1182, 1065, 727, 596 cm^{-1}; HRESIMS Calcd for $[C_{64}H_{55}N_3NaO_3S]^+$ (M + Na^+) 968.3856, found 968.3813.

(*R*)-2,2-bis(4-(dibenzylamino)phenyl)-2-(1-(phenylsulfonyl)-4-(2-propylnaphthalen-1-yl)-1*H*-pyrrol-3-yl)acetaldehyde (5g)

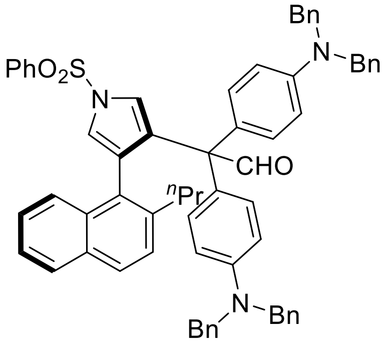

5g

Compound **5g** was prepared in 70% yield (67.2 mg) according to the general procedure at 20 °C as a colorless solid (mp 103–104 °C, eluent: PE/EtOAc = 10/1). $[\alpha]_D^{20}$ = -28.8° (c = 1.0, $CHCl_3$). 80% ee (determined by HPLC: Chiralpak IE Column, 50/50 *i*-PrOH/hexane, 1.0 mL/min, 254 nm; TR = 10.26 min (major), 33.56 min (minor)). ^{1}H NMR (400 MHz, $CDCl_3$) δ 9.17 (s, 1H), 7.86 – 7.79 (m, 2H), 7.63 (d, *J* = 8.4 Hz, 1H), 7.57 (d, *J* = 8.4 Hz, 1H), 7.52 – 7.44 (m, 1H), 7.38 – 7.30 (m, 10H), 7.27 – 7.19 (m, 14H), 7.08 (d, *J* = 8.4 Hz, 1H), 6.95 (d, *J* = 2.4 Hz, 1H), 6.94 – 6.89 (m, 2H), 6.58 (d, *J* = 9.2 Hz, 2H), 6.47 (d, *J* = 8.8 Hz, 2H), 6.37 – 6.33 (m, 4H), 4.61 – 4.49 (m, 8H), 2.02 – 1.85 (m, 2H), 1.27 – 1.25 (m, 2H), 0.61 (t, *J* = 7.6 Hz, 3H); ^{13}C NMR (100 MHz, $CDCl_3$) δ 195.7, 147.9, 147.8, 139.7, 138.8, 138.5, 133.7, 133.5, 131.4, 131.3, 130.7, 130.5, 129.2, 129.1, 128.6, 128.5, 127.9, 127.1, 126.9(4), 126.9(1), 126.8(8), 126.8, 126.7, 126.6, 126.5(7), 126.5, 126.3, 125.1, 124.5, 123.3, 121.2, 111.6, 111.5, 63.5, 54.2, 54.1, 36.0, 24.3, 14.2; IR (neat): 2925(bs), 1723(s), 1608, 1515, 1374, 1233, 1183, 1067, 727, 599 cm^{-1}; HRESIMS Calcd for $[C_{65}H_{57}N_3NaO_3S]^+$ (M + Na^+) 982.4013, found 982.3981.

(*R*)-2-(4-(2-cyclopentylnaphthalen-1-yl)-1-(phenylsulfonyl)-1*H*-pyrrol-3-yl)-2,2-bis(4-(dibenzylamino)phenyl)acetaldehyde (5h)

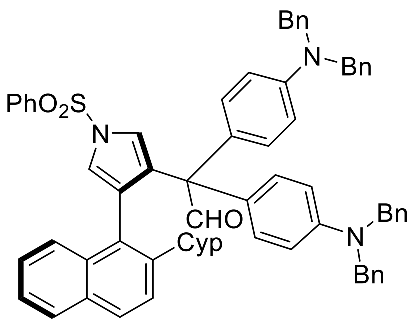

5h

Compound **5h** was prepared in 41% yield (40.4 mg) according to the general procedure using **L3** as ligand, and isolated as a colorless solid (mp 119–120 °C, eluent: PE/EtOAc = 10/1). $[\alpha]_D^{20}$ = -5.5° (c = 1.0, $CHCl_3$). 85% ee (determined by HPLC: Chiralpak IE Column, 50/50 *i*-PrOH/hexane, 1.0 mL/min, 254 nm; TR = 11.12 min (major), 43.30 min (minor)). ^{1}H NMR (400 MHz, $CDCl_3$) δ 9.15 (s, 1H), 7.85 – 7.77 (m, 2H), 7.66 – 7.59 (m, 2H), 7.50 – 7.44 (m, 1H), 7.36 – 7.31 (m, 9H), 7.29 – 7.20 (m, 12H), 7.17 (d, *J* = 8.0 Hz, 4H), 7.01 – 6.93 (m, 2H), 6.79 (d, *J* = 8.4 Hz, 1H), 6.57 (d, *J* = 8.8 Hz, 2H), 6.48 – 6.44 (m, 4H), 6.24 (d, *J* = 9.2 Hz, 2H), 4.61 (s, 4H), 4.52 – 4.39 (m, 4H), 2.61 – 2.50 (m, 1H), 1.70 – 1.51 (m, 4H), 1.32 – 1.21 (m, 4H); ^{13}C NMR (100 MHz, $CDCl_3$) δ 195.7, 148.0, 147.9, 143.3, 138.9, 138.6, 138.5, 133.6, 133.4, 131.7, 131.4, 130.7, 130.6, 129.2, 129.1, 128.7, 128.6, 127.4, 127.2, 127.1, 126.9(4), 126.8(9), 126.7(3), 126.6(5), 126.5(8), 126.5(5), 125.7, 125.3, 124.6, 123.9, 123.8, 121.5, 111.6, 111.5, 63.9, 54.3, 54.1, 43.1, 36.6, 32.6, 26.6, 25.9; IR (neat): 2923(bs), 1722(s), 1604, 1515, 1374, 1231, 1183, 1063, 727, 544 cm^{-1}; HRESIMS Calcd for $[C_{67}H_{59}N_3NaO_3S]^+$ (M + Na^+) 1008.4169, found 1008.4137.

(*R*)-2,2-bis(4-(dibenzylamino)phenyl)-2-(4-(2-ethyl-7-methylnaphthalen-1-yl)-1-(phenylsulfonyl)-1*H*-pyrrol-3-yl)acetaldehyde (5i)

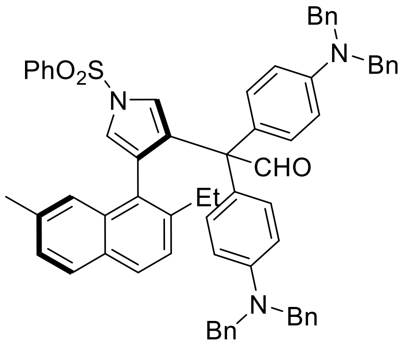

5i

Compound **5i** was prepared in 98% yield (94.1 mg) according to the general procedure as a colorless solid (mp 109–110 °C, eluent: PE/EtOAc = 10/1). $[\alpha]_D^{20}$ = -45.1° (c = 1.0, $CHCl_3$). 95% ee (determined by HPLC: Chiralpak IE Column, 50/50 *i*-PrOH/hexane, 1.0 mL/min, 254 nm; TR = 10.37 min (major), 9.25 min (minor)). ^{1}H NMR (400 MHz, $CDCl_3$) δ 9.16 (s, 1H), 7.82 (d, *J* = 8.0 Hz, 2H), 7.56 – 7.50 (m, 2H), 7.49 – 7.45 (m, 1H), 7.38 – 7.30 (m, 10H), 7.28 – 7.27 (m, 1H), 7.26 – 7.19 (m, 12H), 7.07 (d, *J* = 8.0 Hz, 1H), 7.03 (d, *J* = 8.4 Hz, 1H), 6.96 (d, *J* = 2.8 Hz, 1H), 6.73 (s, 1H), 6.59 (d, *J* = 8.8 Hz, 2H), 6.47 – 6.36 (m, 4H), 6.31 (d, *J* = 8.8 Hz, 2H), 4.62 – 4.48 (m, 8H), 2.07 (s, 3H), 2.00 – 1.90 (m, 2H), 0.82 (t, *J* = 7.6 Hz, 3H); ^{13}C NMR (100 MHz, $CDCl_3$) δ 195.7, 147.9, 147.8, 141.1, 138.9, 138.6, 134.7, 133.6(4), 133.6(0), 131.1, 130.7, 130.5, 129.7, 129.1, 128.6(2), 128.6(1), 128.2, 127.8, 127.3, 127.1, 126.9, 126.7, 126.6(6), 126.6(1), 126.5, 126.3, 125.7, 125.5, 123.6, 121.0, 111.5(4), 111.5(0), 63.7, 54.3, 54.2, 27.1, 21.8, 15.7; IR (neat): 2924(bs), 1722(s), 1608, 1515, 1374, 1234, 1183, 1062, 727, 553 cm^{-1}; HRESIMS Calcd for $[C_{65}H_{57}N_3NaO_3S]^+$ (M + Na$^+$) 982.4013, found 982.3978.

(*R*)-2,2-bis(4-(dibenzylamino)phenyl)-2-(4-(2,7-diethylnaphthalen-1-yl)-1-(phenyl sulfonyl)-1*H*-pyrrol-3-yl)acetaldehyde (5j)

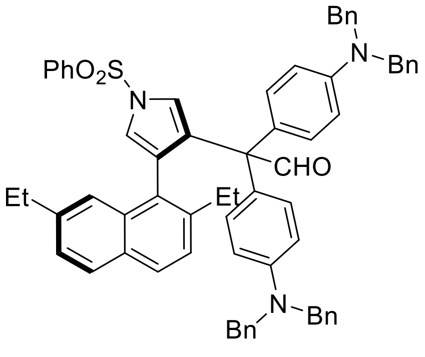

5j

Compound **5j** was prepared in 55% yield (53.6 mg) according to the general procedure as a colorless solid (mp 92–93 °C, eluent: PE/EtOAc = 10/1). $[\alpha]_D^{20}$ = -49.1° (c = 1.0, $CHCl_3$). 98% ee (determined by HPLC: Chiralpak IE Column, 50/50 *i*-PrOH/hexane, 1.0 mL/min, 254 nm; TR = 10.17 min (major), 8.75 min (minor)). ^{1}H

NMR (400 MHz, $CDCl_3$) δ 9.17 (s, 1H), 7.88 – 7.80 (m, 2H), 7.59 – 7.51 (m, 2H), 7.49 – 7.44 (m, 1H), 7.36 – 7.31 (m, 9H), 7.27 – 7.19 (m, 14H), 7.11 (dd, *J* = 8.4, 1.6 Hz, 1H), 7.04 (d, *J* = 8.8 Hz, 1H), 6.96 (d, *J* = 2.8 Hz, 1H), 6.78 (s, 1H), 6.59 (d, *J* = 8.8 Hz, 2H), 6.45 (d, *J* = 8.8 Hz, 2H), 6.39 (d, *J* = 9.2 Hz, 2H), 6.31 (d, *J* = 9.2 Hz, 2H), 4.64 – 4.46 (m, 8H), 2.37 (q, *J* = 7.2 Hz, 2H), 2.02 – 1.89 (m, 2H), 0.99 (t, *J* = 7.6 Hz, 3H), 0.82 (t, *J* = 7.2 Hz, 3H); ^{13}C NMR (100 MHz, $CDCl_3$) δ 195.5, 147.9, 147.8, 141.1, 138.9, 138.5, 133.7, 133.6, 130.8, 130.6, 130.5, 129.9, 129.5, 129.1, 128.5(7), 128.5(5), 128.5(0), 128.4(6), 128.4, 127.8, 127.3, 127.2, 126.9, 126.7, 126.5(9), 126.5(7), 126.5, 126.4, 125.6, 125.5, 124.4, 123.6, 120.9, 111.6, 111.4, 63.7, 54.2, 54.1, 29.1, 27.0, 15.8, 15.6; IR (neat): 2924(bs), 1723(s), 1608, 1515, 1375, 1233, 1183, 1063, 728, 624 cm^{-1}; HRESIMS Calcd for $[C_{66}H_{59}N_3NaO_3S]^+$ (M + Na$^+$) 996.4169, found 996.4130.

(*R*)-2,2-bis(4-(dibenzylamino)phenyl)-2-(4-(2-ethyl-7-isopropylnaphthalen-1-yl)-1-(phenylsulfonyl)-1*H*-pyrrol-3-yl)acetaldehyde (5k)

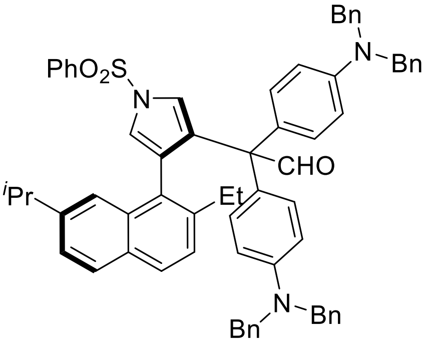

5k

Compound **5k** was prepared in 83% yield (82.0 mg) according to the general procedure as a colorless solid (mp 120–121 °C, eluent: PE/EtOAc = 10/1). $[\alpha]_D^{20}$ = -57.9° (c = 1.0, $CHCl_3$). 99% ee (determined by HPLC: Chiralpak IE Column, 50/50 *i*-PrOH/hexane, 1.0 mL/min, 254 nm; TR = 9.62 min (major), 7.86 min (minor)). ^{1}H NMR (400 MHz, $CDCl_3$) δ 9.17 (s, 1H), 7.85 – 7.80 (m, 2H), 7.57 (d, *J* = 8.4 Hz, 1H), 7.54 (d, *J* = 8.4 Hz, 1H), 7.47 – 7.43 (m, 1H), 7.36 – 7.30 (m, 10H), 7.26 – 7.19 (m, 14H), 7.04 (d, *J* = 8.8 Hz, 1H), 6.96 (d, *J* = 2.4 Hz, 1H), 6.85 (s, 1H), 6.58 (d, *J* = 8.8 Hz, 2H), 6.48 (d, *J* = 8.8 Hz, 2H), 6.38 (d, *J* = 8.8 Hz, 2H), 6.32 (d, *J* = 8.8 Hz, 2H),

4.61 – 4.49 (m, 8H), 2.69 – 2.60 (m, 1H), 2.02 – 1.90 (m, 2H), 1.04 (d, *J* = 7.2 Hz, 3H), 1.00 (d, *J* = 6.8 Hz, 3H), 0.81 (t, *J* = 7.6 Hz, 3H); ^{13}C NMR (100 MHz, $CDCl_3$) δ 195.5, 148.0, 147.9, 145.6, 141.1, 138.9, 138.5(4), 138.5(2), 133.7, 133.6, 130.6, 130.5, 130.1, 129.1, 128.5(8), 128.5(7), 128.5, 127.7, 127.4, 127.1, 126.9, 126.6(3), 126.5(9), 126.5(7), 125.6, 124.0, 123.6, 123.0, 120.7, 111.7, 111.5, 63.8, 54.2, 54.1, 34.2, 27.0, 24.0, 23.8, 15.6; IR (neat): 2923(bs), 1724(s), 1608, 1515, 1375, 1232, 1183, 1066, 727, 599 cm^{-1}; HRESIMS Calcd for $[C_{67}H_{61}N_3NaO_3S]^+$ (M + Na^+) 1010.4326, found 1010.4296.

(*R*)-2,2-bis(4-(dibenzylamino)phenyl)-2-(4-(2-ethyl-7-methoxynaphthalen-1-yl)-1-(phenylsulfonyl)-1*H*-pyrrol-3-yl)acetaldehyde (5l)

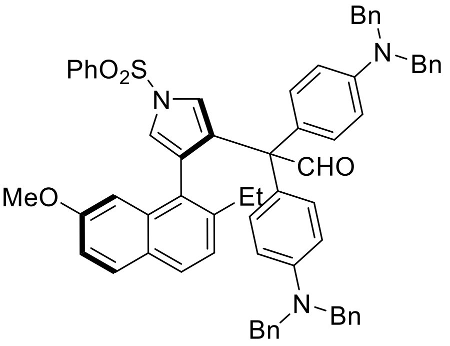

5l

Compound **5l** was prepared in 78% yield (76.1 mg) according to the general procedure as a colorless solid (mp 108–109 °C, eluent: PE/EtOAc = 5/1). $[\alpha]_D^{20}$ = -17.6° (c = 1.0, $CHCl_3$). 99% ee (determined by HPLC: Chiralpak IE Column, 50/50 *i*-PrOH/hexane, 1.0 mL/min, 254 nm; TR = 11.37 min (major), 12.62 min (minor)). ^{1}H NMR (400 MHz, $CDCl_3$) δ 9.21 (s, 1H), 7.86 – 7.81 (m, 2H), 7.52 (d, *J* = 8.8 Hz, 2H), 7.50 – 7.45 (m, 1H), 7.36 – 7.30 (m, 11H), 7.29 – 7.26 (m, 1H), 7.25 – 7.18 (m, 11H), 7.00 – 6.95 (m, 2H), 6.91 (dd, *J* = 8.8, 2.4 Hz, 1H), 6.57 (d, *J* = 8.8 Hz, 2H), 6.54 (d, *J* = 8.8 Hz, 2H), 6.35 (d, *J* = 7.6 Hz, 4H), 6.25 (d, *J* = 2.4 Hz, 1H), 4.59 – 4.51 (m, 8H), 3.26 (s, 3H), 2.02 – 1.92 (m, 2H), 0.81 (t, *J* = 7.6 Hz, 3H); ^{13}C NMR (100 MHz, $CDCl_3$) δ 195.5, 157.1, 148.0, 147.9, 141.8, 139.0, 138.5, 136.8, 134.8, 133.6, 130.7, 130.6, 130.5, 129.1, 128.9, 128.7, 128.6, 127.8, 127.7, 127.4, 127.1, 126.9, 126.7, 126.6, 126.5, 126.3, 124.0, 123.4, 120.7, 117.3, 111.6(0), 111.5(9),

104.7, 63.6, 54.7, 54.2(1), 54.2(0), 27.1, 15.5; IR (neat): 2923(bs), 1722(s), 1607, 1514, 1375, 1227, 1183, 1065, 727, 598 cm^{-1}; HRESIMS Calcd for $[C_{65}H_{57}N_3NaO_4S]^+$ (M + Na^+) 998.3962, found 998.3936.

(*R*)-2,2-bis(4-(dibenzylamino)phenyl)-2-(4-(2-ethyl-7-(trimethylsilyl)naphthalen-1-yl)-1-(phenylsulfonyl)-1*H*-pyrrol-3-yl)acetaldehyde (5m)

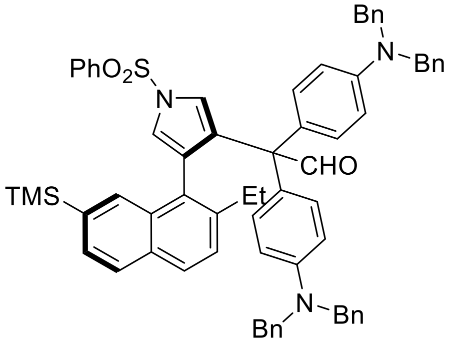

5m

Compound **5m** was prepared in 40% yield (40.7 mg) according to the general procedure as a colorless solid (mp 103–104 °C, eluent: PE/EtOAc = 10/1). $[\alpha]_D^{20}$ = -38.4° (c = 1.0, $CHCl_3$). 89% ee (determined by HPLC: Chiralpak IE Column, 50/50 *i*-PrOH/hexane, 1.0 mL/min, 254 nm; TR = 8.06 min (major), 7.47 min (minor)). ^1H NMR (400 MHz, $CDCl_3$) δ 9.22 (s, 1H), 7.85 (d, *J* = 8.0 Hz, 2H), 7.64 (d, *J* = 8.0 Hz, 1H), 7.58 (d, *J* = 8.8 Hz, 1H), 7.50 – 7.44 (m, 1H), 7.41 (d, *J* = 8.0 Hz, 1H), 7.37 – 7.31 (m, 11H), 7.28 – 7.21 (m, 11H), 7.21 – 7.17 (m, 2H), 7.11 (d, *J* = 8.4 Hz, 1H), 6.98 (d, *J* = 2.4 Hz, 1H), 6.62 – 6.54 (m, 4H), 6.39 (d, *J* = 8.4 Hz, 2H), 6.32 (d, *J* = 8.8 Hz, 2H), 4.63 – 4.47 (m, 8H), 2.09 – 1.90 (m, 2H), 0.82 (t, *J* = 7.6 Hz, 3H), 0.06 (s, 9H); ^{13}C NMR (100 MHz, $CDCl_3$) δ 195.5, 148.0, 147.8, 141.3, 138.9, 138.6, 138.5, 137.1, 133.8, 133.0, 132.0, 131.7, 130.7, 130.4, 130.2, 129.3, 129.0, 128.5(8), 128.5(7), 128.5, 127.9, 127.0, 126.8(9), 126.8(7), 126.7(0), 126.6(7), 126.6(2), 126.6(1), 126.5(3), 126.4(7), 123.3, 120.4, 111.7, 111.5, 63.8, 54.2, 54.1, 26.9, 15.5, -1.1; IR (neat): 2926(bs), 1723(s), 1608, 1515, 1361, 1247, 1183, 1072, 728, 598 cm^{-1}; HRESIMS Calcd for $[C_{67}H_{63}N_3NaO_3SSi]^+$ (M + Na^+) 1040.4252, found 1040.4212.

(*R*)-2,2-bis(4-(dibenzylamino)phenyl)-2-(4-(2-ethyl-6-methylnaphthalen-1-yl)-1-(

phenylsulfonyl)-1*H*-pyrrol-3-yl)acetaldehyde (5n)

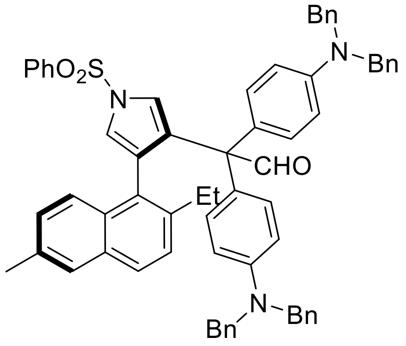

5n

Compound **5n** was prepared in 63% yield (60.5 mg) according to the general procedure as a colorless solid (mp 100–101 °C, eluent: PE/EtOAc = 10/1). $[\alpha]_D^{20}$ = -31.2° (c = 1.0, $CHCl_3$). 97% ee (determined by HPLC: Chiralpak IE Column, 50/50 *i*-PrOH/hexane, 1.0 mL/min, 254 nm; TR = 12.21 min (major), 37.39 min (minor)). ^{1}H NMR (400 MHz, $CDCl_3$) δ 9.14 (s, 1H), 7.79 (d, *J* = 7.2 Hz, 2H), 7.52 (d, *J* = 8.4 Hz, 1H), 7.48 – 7.44 (m, 1H), 7.41 (s, 1H), 7.37 – 7.30 (m, 10H), 7.25 – 7.17 (m, 13H), 7.07 (d, *J* = 8.4 Hz, 1H), 6.95 (d, *J* = 2.4 Hz, 1H), 6.82 (s, 2H), 6.57 (d, *J* = 8.8 Hz, 2H), 6.48 (d, *J* = 8.8 Hz, 2H), 6.41 – 6.34 (m, 4H), 4.58 (s, 4H), 4.55 (s, 4H), 2.41 (s, 3H), 2.00 – 1.88 (m, 2H), 0.80 (t, *J* = 7.6 Hz, 3H); ^{13}C NMR (100 MHz, $CDCl_3$) δ 195.9, 147.9, 147.8, 140.3, 138.8, 138.6, 133.9, 133.7, 132.0, 131.7, 131.6, 130.7, 130.6, 129.1, 128.6, 127.6, 127.4, 127.2, 126.9(2), 126.9(0), 126.8, 126.6(4), 126.5(9), 126.4(3), 126.3(7), 126.3, 123.6, 121.3, 111.6, 111.5, 63.6, 54.2, 26.9, 21.4, 15.6; IR (neat): 2924(bs), 1723(s), 1607, 1515, 1361, 1235, 1183, 1061, 727, 591 cm^{-1}; HRESIMS Calcd for $[C_{65}H_{57}N_3NaO_3S]^+$ (M + Na^+) 982.4013, found 982.3998.

(*R*)-2,2-bis(4-(dimethylamino)phenyl)-2-(4-(2-ethylnaphthalen-1-yl)-1-(phenylsulfonyl)-1*H*-pyrrol-3-yl)acetaldehyde (5o)

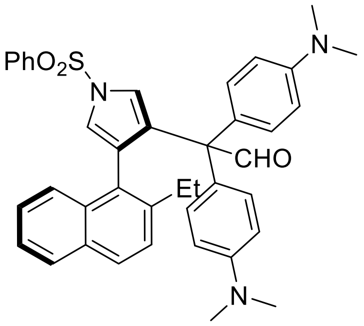

5o

Compound **5o** was prepared in 80% yield (51.3 mg) according to the general procedure as a colorless solid (mp 90–91 °C, eluent: PE/EtOAc = 10/1). $[\alpha]_D^{20}$ = -28.1° (c = 1.0, $CHCl_3$). 93% ee (determined by HPLC: Chiralpak IE Column, 50/50 *i*-PrOH/hexane, 1.0 mL/min, 254 nm; TR = 16.83 min (major), 24.43 min (minor)). ^{1}H NMR (400 MHz, $CDCl_3$) δ 9.28 (s, 1H), 7.95 – 7.89 (m, 2H), 7.74 – 7.68 (m, 1H), 7.62 – 7.54 (m, 4H), 7.32 (d, *J* = 2.4 Hz, 1H), 7.23 – 7.18 (m, 1H), 7.10 (d, *J* = 8.4 Hz, 1H), 7.01 (d, *J* = 2.4 Hz, 1H), 6.99 – 6.94 (m, 1H), 6.87 (d, *J* = 8.4 Hz, 1H), 6.67 – 6.59 (m, 4H), 6.39 (d, *J* = 9.2 Hz, 2H), 6.28 (d, *J* = 8.8 Hz, 2H), 2.89 (s, 6H), 2.83 (s, 6H), 2.20 – 2.03 (m, 2H), 0.86 (t, *J* = 7.6 Hz, 3H); ^{13}C NMR (100 MHz, $CDCl_3$) δ 195.7, 149.4, 149.1, 141.1, 139.0, 133.7, 133.4, 131.4, 130.7, 130.6, 130.4, 129.2, 128.9, 128.1, 127.1, 127.0, 126.8, 126.4(2), 126.3(8), 126.3, 125.9, 125.1, 124.4, 123.4, 121.2, 111.8, 111.5, 63.6, 40.4, 40.3, 27.2, 15.6; IR (neat): 2923(bs), 1722(s), 1609, 1518, 1372, 1264, 1183, 1065, 727, 563 cm^{-1}; HRESIMS Calcd for $[C_{40}H_{39}N_3NaO_3S]^+$ (M + Na^+) 664.2604, found 664.2638.

(*R*)-2-(4-(2-ethylnaphthalen-1-yl)-1-(phenylsulfonyl)-1*H*-pyrrol-3-yl)-2,2-bis(4-(pyrrolidin-1-yl)phenyl)acetaldehyde (5p)

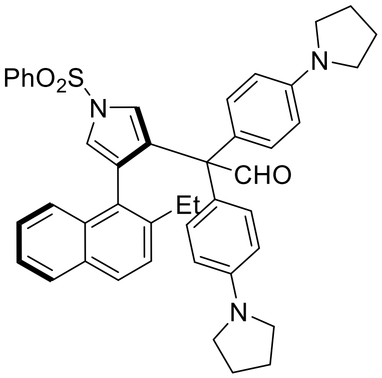

5p

Compound **5p** was prepared in 58% yield (40.2 mg) according to the general procedure as a colorless solid (mp 90–91 °C, eluent: PE/EtOAc = 10/1). $[\alpha]_D^{20}$ = -17.7° (c = 1.0, $CHCl_3$). 94% ee (determined by HPLC: Chiralpak IE Column, 50/50 *i*-PrOH/hexane, 1.0 mL/min, 254 nm; TR = 28.08 min (major), 53.61 min (minor)). ^{1}H NMR (400 MHz, $CDCl_3$) δ 9.28 (s, 1H), 7.92 (d, *J* = 7.2 Hz, 2H), 7.73 – 7.67 (m, 1H), 7.60 – 7.51 (m, 4H), 7.35 (d, *J* = 2.4 Hz, 1H), 7.20 – 7.16 (m, 1H), 7.10 (d, *J* = 8.4 Hz, 1H), 7.00 (d, *J* = 2.4 Hz, 1H), 6.98 – 6.93 (m, 1H), 6.86 (d, *J* = 8.8 Hz, 1H), 6.68 – 6.56 (m, 4H), 6.23 (d, *J* = 8.8 Hz, 2H), 6.07 (d, *J* = 8.8 Hz, 2H), 3.25 – 3.18 (m, 4H), 3.16 – 3.08 (m, 4H), 2.23 – 2.07 (m, 2H), 2.02 – 1.93 (m, 8H), 0.87 (t, *J* = 7.6 Hz, 3H); ^{13}C NMR (100 MHz, $CDCl_3$) δ 195.6, 146.7, 146.4, 141.1, 139.1, 133.7, 133.5, 131.5, 130.9, 130.6, 130.5, 129.2, 129.1, 128.0, 127.3, 127.0, 126.8, 126.5, 126.3, 125.3, 125.1, 124.8, 124.3, 123.4, 121.2, 110.9, 110.7, 63.7, 47.5, 47.3, 27.2, 25.4, 15.6; IR (neat): 2928(bs), 1721(s), 1608, 1516, 1373, 1263, 1183, 1064, 727, 596 cm^{-1}; HRESIMS Calcd for $[C_{44}H_{43}N_3NaO_3S]^+$ (M + Na^+) 716.2917, found 716.2911.

(*R*)-2-(4-(2-ethylnaphthalen-1-yl)-1-(phenylsulfonyl)-1*H*-pyrrol-3-yl)-2,2-bis(4-methoxyphenyl)acetaldehyde (5q)

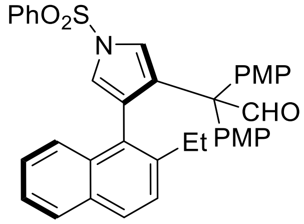

5q

Compound **5q** was prepared in 72% yield (44.3 mg) according to the general procedure at 25 °C as a colorless solid (mp 153–154 °C, eluent: PE/EtOAc = 10/1). $[\alpha]_D^{20}$ = -29.3° (c = 1.0, $CHCl_3$). 87% ee (determined by HPLC: Chiralpak IE Column, 50/50 *i*-PrOH/hexane, 1.0 mL/min, 254 nm; TR = 9.97 min (major), 14.59 min (minor)). ^{1}H NMR (400 MHz, $CDCl_3$) δ 9.19 (s, 1H), 7.82 (d, *J* = 7.6 Hz, 2H), 7.64 – 7.60 (m, 1H), 7.54 – 7.46 (m, 4H), 7.18 – 7.13 (m, 2H), 7.03 (d, *J* = 8.4 Hz, 1H), 6.97 (d, *J* = 2.0 Hz, 1H), 6.94 – 6.88 (m, 1H), 6.78 (d, *J* = 8.4 Hz, 1H), 6.64 – 6.53 (m, 4H),

6.48 (d, *J* = 8.8 Hz, 2H), 6.41 (d, *J* = 8.8 Hz, 2H), 3.63 (s, 3H), 3.58 (s, 3H), 2.08 – 1.92 (m, 2H), 0.78 (t, *J* = 7.6 Hz, 3H); ^{13}C NMR (100 MHz, $CDCl_3$) δ 195.5, 158.5, 158.3, 141.1, 138.8, 133.9, 133.2, 131.4, 130.9, 130.7(2), 130.6(8), 130.4(2), 130.3(6), 129.3, 128.5, 128.3, 127.2, 126.7, 126.3, 126.1, 125.3, 124.6, 123.3, 121.4, 113.2, 112.9, 63.8, 55.1, 55.0, 27.1, 15.6; IR (neat): 2930(bs), 1726(s), 1606, 1508, 1374, 1252, 1182, 1068, 727, 597 cm^{-1}; HRESIMS Calcd for $[C_{38}H_{33}NNaO_5S]^+$ (M + Na^+) 638.1972, found 638.1951.

(*R*)-2-(4-(2-ethylnaphthalen-1-yl)-1-(phenylsulfonyl)-1*H*-pyrrol-3-yl)-2,2-bis(4-((triisopropylsilyl)oxy)phenyl)acetaldehyde (5r)

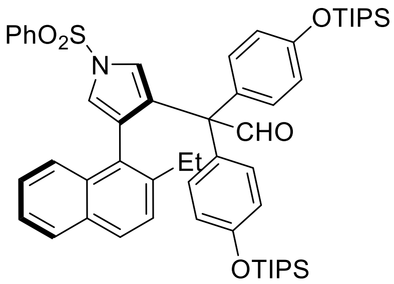

5r

Compound **5r** was prepared in 49% yield (44.1 mg) according to the general procedure as a colorless oil (eluent: PE/EtOAc = 10/1). $[\alpha]_D^{20}$ = -30.3° (c = 1.0, $CHCl_3$). 90% ee (determined by HPLC: Chiralpak IE Column, 50/50 *i*-PrOH/hexane, 1.0 mL/min, 254 nm; TR = 4.27 min (major), 5.48 min (minor)). ^{1}H NMR (400 MHz, $CDCl_3$) δ 9.11 (s, 1H), 7.90 – 7.82 (m, 2H), 7.75 – 7.62 (m, 3H), 7.61 – 7.53 (m, 2H), 7.26 – 7.23 (m, 1H), 7.16 (d, *J* = 8.8 Hz, 1H), 7.10 – 7.01 (m, 3H), 6.99 (d, *J* = 8.4 Hz, 1H), 6.67 (d, *J* = 8.8 Hz, 4H), 6.62 (d, *J* = 9.2 Hz, 4H), 2.11 – 1.99 (m, 2H), 1.26 – 1.20 (m, 6H), 1.10 (d, *J* = 2.0 Hz, 18H), 1.09 (d, *J* = 1.6 Hz, 18H), 0.87 (t, *J* = 7.6 Hz, 3H); ^{13}C NMR (100 MHz, $CDCl_3$) δ 195.8, 155.1(4), 155.0(9), 141.4, 138.9, 133.9, 133.3, 132.1, 131.5(3), 131.4(6), 131.3, 131.0, 130.8, 129.2, 128.6, 127.5, 126.8, 126.5, 126.1, 125.6, 124.8, 123.7, 121.4, 119.2, 119.1, 64.1, 27.1, 17.9, 15.7, 12.6; IR (neat): 2944(bs), 1728(s), 1603, 1506, 1382, 1367, 1176, 1067, 727, 595 cm^{-1}; HRESIMS Calcd for $[C_{54}H_{69}NNaO_5SSi_2]^+$ (M + Na^+) 922.4327, found 922.4295.

(*R*)-2,2-bis(benzo[*d*][1,3]dioxol-5-yl)-2-(4-(2-ethylnaphthalen-1-yl)-1-(phenylsulfo

nyl)-1*H*-pyrrol-3-yl)acetaldehyde (5s)

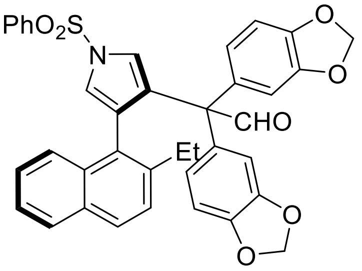

5s

Compound **5s** was prepared in 43% yield (27.7 mg) according to the general procedure at 25 °C as a colorless oil (eluent: PE/EtOAc = 5/1). $[\alpha]_D^{20}$ = -20.2° (c = 1.0, $CHCl_3$). 87% ee (determined by HPLC: Chiralpak IE Column, 50/50 *i*-PrOH/hexane, 1.0 mL/min, 254 nm; TR = 17.24 min (major), 21.10 min (minor)). ^{1}H NMR (400 MHz, $CDCl_3$) δ 9.27 (s, 1H), 7.96 – 7.88 (m, 2H), 7.76 – 7.70 (m, 1H), 7.66 – 7.57 (m, 4H), 7.31 (d, *J* = 2.8 Hz, 1H), 7.28 – 7.25 (m, 1H), 7.15 (d, *J* = 8.4 Hz, 1H), 7.07 – 7.01 (m, 2H), 6.87 (d, *J* = 8.4 Hz, 1H), 6.50 – 6.44 (m, 1H), 6.37 (d, *J* = 8.4 Hz, 1H), 6.27 (d, *J* = 1.6 Hz, 1H), 6.26 – 6.19 (m, 3H), 5.89 (d, *J* = 1.6 Hz, 1H), 5.88 (d, *J* = 1.2 Hz, 1H), 5.84 (d, *J* = 1.6 Hz, 1H), 5.82 (d, *J* = 1.2 Hz, 1H), 2.18 – 2.08 (m, 2H), 0.89 (t, *J* = 7.2 Hz, 3H); ^{13}C NMR (100 MHz, $CDCl_3$) δ 194.9, 147.3, 147.1, 146.8, 146.6, 141.1, 138.7, 134.0, 133.2, 132.3, 132.1, 131.4, 129.7, 129.4, 128.5, 128.3, 127.3, 126.7(1), 126.6(9), 126.3, 126.1, 125.2, 124.6, 123.6, 123.5, 123.3, 121.4, 110.4, 110.3, 107.4, 107.2, 101.1, 101.0, 64.5, 27.2, 15.6; IR (neat): 2924(bs), 1728(s), 1503, 1484, 1375, 1241, 1184, 1069, 727, 596 cm^{-1}; HRESIMS Calcd for $[C_{38}H_{29}NNaO_7S]^+$ (M + Na^+) 666.1557, found 666.1569.

(*R*)-2-(1-benzyl-5-chloro-1*H*-indol-3-yl)-2-(4-(dibenzylamino)phenyl)-2-(4-(2-ethylnaphthalen-1-yl)-1-(phenylsulfonyl)-1*H*-pyrrol-3-yl)acetaldehyde (5t)

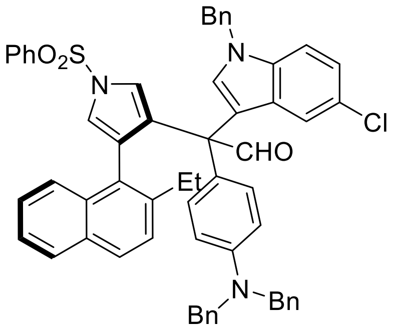

5t

Compound **5t** was prepared in 48% yield (87.8 mg, 0.2 mmol scale) according to the general procedure at -10 °C as a colorless oil (eluent: PE/EtOAc = 20/1). dr = 1.2:1 (determined by ^{1}H NMR analysis of the reaction mixture). $[\alpha]_D^{20}$ = -29.5° (c = 1.0, $CHCl_3$, major). 90% ee, (major, determined by HPLC: Chiralpak IE Column, 30/70 *i*-PrOH/hexane, 1.0 mL/min, 254 nm; TR = 11.37 min (major), 20.19 min (minor)). 95% ee, (minor, determined by HPLC: Chiralpak IE Column, 30/70 *i*-PrOH/hexane, 1.0 mL/min, 254 nm; TR = 11.58 min (major), 16.91 min (minor)). ^{1}H NMR (400 MHz, $CDCl_3$, major) δ 9.26 (s, 1H), 7.80 (d, *J* = 7.6 Hz, 2H), 7.65 – 7.56 (m, 3H), 7.49 (d, *J* = 2.4 Hz, 1H), 7.45 – 7.40 (m, 2H), 7.37 – 7.31 (m, 4H), 7.28 – 7.26 (m, 2H), 7.25 – 7.19 (m, 8H), 7.10 (s, 1H), 7.05 (d, *J* = 8.4 Hz, 1H), 6.99 (d, *J* = 2.4 Hz, 1H), 6.94 (d, *J* = 6.8 Hz, 2H), 6.85 (dd, *J* = 1.6, 8.8 Hz, 1H), 6.84 – 6.68 (m, 5H), 6.45 (d, *J* = 8.8 Hz, 2H), 6.34 (s, 1H), 4.82 (s, 2H), 4.60 (s, 4H), 1.93 – 1.79 (m, 2H), 0.74 (t, *J* = 7.2 Hz, 3H); ^{13}C NMR (100 MHz, $CDCl_3$, major) δ 194.2, 148.3, 141.4, 138.9, 138.4, 137.4, 136.7, 133.8, 133.2, 131.2, 130.4, 130.0, 129.9, 129.2, 128.8, 128.7, 128.4, 127.9, 127.7, 127.0(3), 127.0(0), 126.8, 126.6, 126.4, 126.2, 126.1, 125.9, 124.9, 124.6(6), 124.6(5), 124.5, 122.8, 122.5(4), 122.4(5), 121.3, 115.2, 113.4, 112.5, 112.1, 58.9, 54.3, 49.7, 27.1, 15.7; IR (neat): 2921(bs), 1723(s), 1605, 1516, 1374, 1175, 1063, 728, 598 cm^{-1}; HRESIMS Calcd for $[C_{59}H_{48}ClN_3NaO_3S]^+$ (M + Na$^+$) 936.2997, found 936.3021.

(*R*)-2-(1-benzyl-5-bromo-1*H*-indol-3-yl)-2-(4-(dibenzylamino)phenyl)-2-(4-(2-ethylnaphthalen-1-yl)-1-(phenylsulfonyl)-1*H*-pyrrol-3-yl)acetaldehyde (5u)

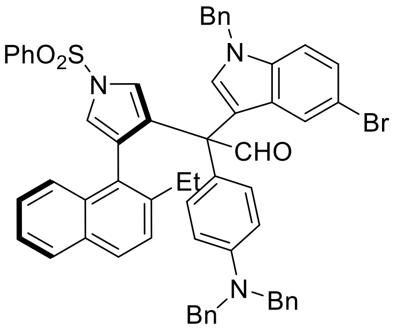

5u

Compound **5u** was prepared in 54% yield (103.6 mg, 0.2 mmol scale) according to the general procedure at -10 °C as a colorless oil (eluent: PE/EtOAc = 20/1). dr = 1.6:1 (determined by ^{1}H NMR analysis of the reaction mixture). $[\alpha]_D^{20}$ = -42.6° (c = 1.0, $CHCl_3$, major). $[\alpha]_D^{20}$ = -72.8° (c = 1.0, $CHCl_3$, minor). 95% ee (major, determined by HPLC: Chiralpak IE Column, 30/70 *i*-PrOH/hexane, 1.0 mL/min, 254 nm; TR = 11.97 min (major), 20.33 min (minor)). 95% ee (minor, determined by HPLC: Chiralpak IE Column, 30/70 *i*-PrOH/hexane, 1.0 mL/min, 254 nm; TR = 15.53 min (major), 6.84 min (minor)). ^{1}H NMR (400 MHz, $CDCl_3$, major) δ 9.33 (s, 1H), 7.83 (d, *J* = 7.6 Hz, 2H), 7.62 – 7.52 (m, 4H), 7.49 – 7.44 (m, 2H), 7.37 – 7.33 (m, 4H), 7.25 – 7.17 (m, 10H), 7.05 – 6.99 (m, 2H), 6.96 (d, *J* = 2.4 Hz, 1H), 6.93 – 6.87 (m, 3H), 6.83 – 6.69 (m, 5H), 6.48 (d, *J* = 8.8 Hz, 2H), 6.33 (s, 1H), 4.73 (d, *J* = 2.0 Hz, 2H), 4.62 (s, 4H), 1.98 – 1.82 (m, 2H), 0.73 (t, *J* = 7.6 Hz, 3H); ^{1}H NMR (500 MHz, $CDCl_3$, minor) δ 9.34 (s, 1H), 7.82 – 7.77 (m, 2H), 7.66 (d, *J* = 8.5 Hz, 2H), 7.59 – 7.54 (m, 1H), 7.48 – 7.43 (m, 3H), 7.37 – 7.33 (m, 4H), 7.28 – 7.25 (m, 2H), 7.25 – 7.19 (m, 8H), 7.17 – 7.10 (m, 3H), 6.96 (d, *J* = 2.5 Hz, 1H), 6.91 – 6.84 (m, 5H), 6.70 – 6.64 (m, 1H), 6.55 (d, *J* = 9.0 Hz, 2H), 6.39 (d, *J* = 8.5 Hz, 1H), 6.14 (s, 1H), 4.74 (d, *J* = 16.0 Hz, 1H), 4.64 – 4.57 (m, 5H), 2.24 – 2.17 (m, 1H), 2.11 – 2.04 (m, 1H), 0.90 (t, *J* = 7.5 Hz, 3H); ^{13}C NMR (100 MHz, $CDCl_3$, major) δ 194.3, 148.3, 141.3, 138.8, 138.4, 136.8, 135.1, 133.8, 133.1, 131.2, 130.9, 130.1, 129.4, 129.1, 128.8, 128.7, 128.5, 127.8, 127.7, 127.0, 126.9, 126.6, 126.5, 126.4, 126.2, 126.1, 124.7, 124.6, 124.5, 124.3, 123.7, 122.9, 121.3, 112.8, 112.6, 112.1, 111.1, 58.9, 54.3, 49.7, 27.2, 15.6; ^{13}C NMR (125 MHz, $CDCl_3$, minor) δ 193.9, 148.5, 141.4, 138.7, 138.3, 136.7, 135.2, 133.8, 133.6, 131.1, 130.4(2), 130.3(9), 129.7, 129.4, 128.7(2),

128.6(8), 128.1(0), 128.0(8), 127.6, 127.1, 127.0, 126.9, 126.6, 126.5, 126.4, 126.3, 126.0, 124.9, 124.5, 124.3, 123.7, 122.9, 121.3, 112.5(1), 112.4(5), 112.2, 111.2, 58.8, 54.2, 49.8, 27.1, 16.0; IR (neat): 2922(bs), 1723(s), 1606, 1515, 1372, 1174, 1068, 727, 593 cm^{-1}; HRESIMS Calcd for $[C_{59}H_{48}BrN_3NaO_3S]^+$ (M + Na^+) 980.2492, found 980.2469.

(*R*)-2-(1-benzyl-1*H*-pyrrol-2-yl)-2-(4-(dibenzylamino)phenyl)-2-(4-(2-ethylnaphthalen-1-yl)-1-(phenylsulfonyl)-1H-pyrrol-3-yl)acetaldehyde (5v)

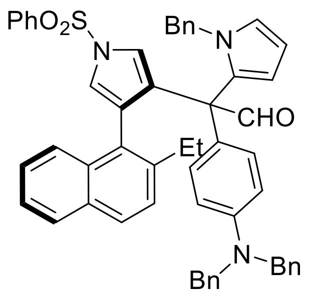

5v

Compound **5v** was prepared in 47% yield (78.0 mg, 0.2 mmol scale) according to the general procedure at -10 °C as a colorless oil (eluent: PE/EtOAc = 20/1). dr = 1.4:1 (determined by ^{1}H NMR analysis of the reaction mixture). 98% ee (major, determined by HPLC: Chiralpak IE Column, 10/90 *i*-PrOH/hexane, 1.0 mL/min, 254 nm; TR = 18.59 min (major), 23.39 min (minor)). 97% ee (minor, determined by HPLC: Chiralpak IE Column, 10/90 *i*-PrOH/hexane, 1.0 mL/min, 254 nm; TR = 21.65 min (major), 52.74 min (minor)). ^{1}H NMR (400 MHz, $CDCl_3$) δ 9.10 (s, 1H, major), 8.99 (s, 0.7H, minor), 7.87 – 7.83 (m, 4H, major + minor), 7.69 – 7.65 (m, 3H, major + minor), 7.59 – 7.52 (m, 3H, major + minor), 7.47 – 7.42 (m, 4H, major + minor), 7.35 – 7.32 (m, 8H, major + minor), 7.29 – 7.25 (m, 6H, major + minor), 7.21 – 7.18 (m, 7H, major + minor), 7.12 – 7.05 (m, 6H, major + minor), 6.96 – 6.87 (m, 6H, major + minor), 6.58 – 6.53 (m, 4H, major + minor), 6.41 – 6.38 (m, 4H, major + minor), 6.34 – 6.32 (m, 1H, major + minor), 6.28 – 6.26 (m, 1H, major + minor), 5.91 – 5.87 (m, 2H, major + minor), 5.39 – 5.34 (m, 2H, major + minor), 4.59 (s, 7H, major + minor), 4.53 – 4.46 (m, 2H, major), 4.41 – 4.36 (m, 1.4H, minor), 2.24 (q, *J* = 7.6 Hz, 2H, major), 2.07 – 1.98 (m, 1.5H, minor), 0.95 (t, *J* = 7.6 Hz, 2.3H, minor), 0.81 (t, *J* = 7.6

Hz, 3H, major); ^{13}C NMR (100 MHz, $CDCl_3$) δ 196.2, 195.7, 148.1(7), 148.1(5), 141.4(2), 141.3(6), 138.8, 138.4, 137.2, 133.9, 133.7, 133.5, 133.1, 132.0, 131.5(7), 131.5(5), 131.5, 130.9, 130.5, 129.9, 129.8, 129.3(0), 129.2(9), 129.1, 128.6, 128.5, 128.4, 128.1, 127.8, 127.7, 127.5, 127.4, 127.3, 127.0, 126.8, 126.7, 126.6, 126.4, 126.2, 126.0, 125.8, 125.7, 125.2, 125.1, 124.9, 124.7, 123.1, 123.0, 121.7, 112.4, 111.8, 109.7, 106.8(2), 106.7(6), 60.8, 60.6, 54.3, 51.9, 51.8, 27.1, 26.8, 15.6, 15.5; IR (neat): 2924(bs), 1722(s), 1609, 1517, 1378, 1183, 1070, 727, 595 cm^{-1}; HRESIMS Calcd for $[C_{55}H_{47}N_3NaO_3S]^+$ (M + Na^+) 852.3230, found 852.3252.

1.5 Synthetic Utility Study

(*R*)-2,2-bis(4-(dibenzylamino)phenyl)-2-(4-(2-methylnaphthalen-1-yl)-1*H*-pyrrol-3-yl)acetaldehyde (6)

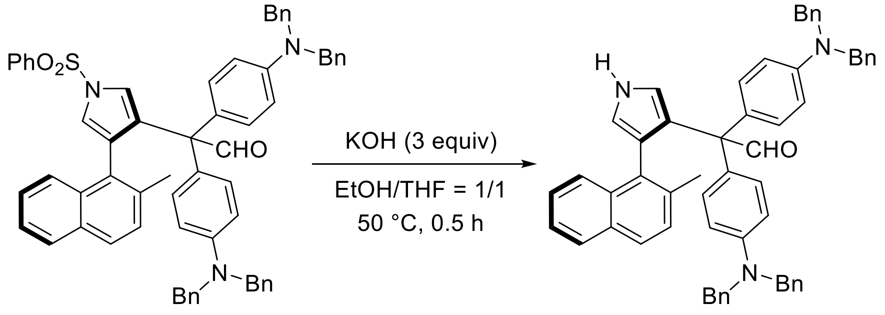

5a, 92% ee **6**, 99%, 92% ee

Compound **6** was prepared in 99% yield (235.2 mg, 0.3 mmol scale) according to the known procedure[10] as a brown solid (mp 92–93 °C, eluent: PE/EtOAc = 5/1). $[\alpha]_D^{20}$ = -657.5° (c = 1.0, $CHCl_3$). 92% ee (determined by HPLC: Chiralpak IE Column, 10/90 *i*-PrOH/hexane, 1.0 mL/min, 254 nm; TR = 20.29 min (major), 22.21 min (minor)). ^{1}H NMR (400 MHz, $CDCl_3$) δ 9.38 (s, 1H), 8.35 (s, 1H), 7.64 (d, *J* = 8.0 Hz, 1H), 7.54 (d, *J* = 8.4 Hz, 1H), 7.36 (d, *J* = 8.4 Hz, 1H), 7.32 – 7.27 (m, 8H), 7.24 – 7.17 (m, 13H), 7.12 (d, *J* = 8.4 Hz, 1H), 7.07 – 6.98 (m, 1H), 6.79 (d, *J* = 8.8 Hz, 2H), 6.71 (d, *J* = 8.8 Hz, 2H), 6.69 – 6.63 (m, 1H), 6.53 – 6.46 (m, 1H), 6.45 – 6.37 (m, 4H), 4.54 (s, 4H), 4.50 (s, 4H), 1.96 (s, 3H); ^{13}C NMR (100 MHz, $CDCl_3$) δ 197.7, 147.7, 147.6, 138.7(3), 138.7(1), 135.6, 134.5, 132.8, 131.5, 131.0, 130.8, 128.7(3), 128.6(5), 128.5(2), 128.4(9), 128.1, 127.2, 127.1, 126.8(4), 126.7(9), 126.7(6), 126.7(0), 126.6(6), 124.8, 124.1, 124.0, 120.3, 120.1, 117.8, 111.4, 111.3, 63.9, 54.1, 21.3; IR (neat): 3022(bs), 1717(s), 1608, 1515, 1233, 1164, 1027, 732, 696 cm^{-1}; HRESIMS Calcd for $[C_{57}H_{49}BrN_3NaO]^+$ (M + Na^+) 814.3768, found 814.3747.

(*R*)-2,2-bis(4-(dibenzylamino)phenyl)-2-(2,5-dibromo-4-(2-methylnaphthalen-1-yl)-1*H*-pyrrol-3-yl)acetaldehyde (7)

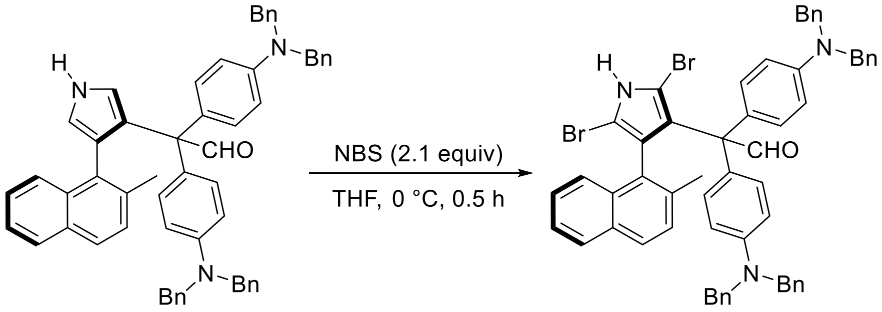

6, 92% ee **7**, 99%, 93% ee

Compound **7** was prepared in 99% yield (94.0 mg, 0.1 mmol scale) according to the known procedure[11] as a brown solid (mp 143–144 °C, eluent: PE/EtOAc = 5/1). $[\alpha]_D^{20}$ = +113.4° (c = 1.0, $CHCl_3$). 93% ee (determined by HPLC: Chiralpak IA Column, 10/90 *i*-PrOH/hexane, 1.0 mL/min, 254 nm; TR = 19.91 min (major), 26.09 min (minor)). ^{1}H NMR (400 MHz, $CDCl_3$) δ 9.43 (s, 1H), 8.63 (s, 1H), 7.66 (d, *J* = 8.0 Hz, 1H), 7.58 (d, *J* = 8.4 Hz, 1H), 7.36 (d, *J* = 8.4 Hz, 1H), 7.32 – 7.27 (m, 9H), 7.23 – 7.17 (m, 12H), 7.14 – 7.09 (m, 2H), 6.82 – 6.73 (m, 4H), 6.42 (d, *J* = 8.8 Hz, 2H), 6.36 (d, *J* = 8.8 Hz, 2H), 4.53 (s, 4H), 4.49 (s, 4H), 2.03 (s, 3H); ^{13}C NMR (100 MHz, $CDCl_3$) δ 196.3, 147.9, 147.8, 138.6(4), 138.6(1), 136.2, 133.7, 131.6, 131.1, 130.8, 129.9, 128.5(2), 128.4(8), 128.2, 127.8, 127.5, 126.8(3), 126.7(9), 126.7(5), 126.7, 126.2, 126.0, 125.8, 125.5, 124.4, 124.0, 123.0, 111.6, 111.5, 101.1, 99.6, 63.8, 54.0, 21.1; IR (neat): 3168(bs), 1721(s), 1607, 1515, 1360, 1199, 1028, 730, 696 cm^{-1}; HRESIMS Calcd for $[C_{57}H_{47}Br_2N_3NaO]^+$ (M + Na^+) 970.1978, found 970.1949.

(*R*)-*tert*-butyl 3-(1,1-bis(4-(dibenzylamino)phenyl)-2-oxoethyl)-4-(2-methylnaphthalen-1-yl)-1*H*-pyrrole-1-carboxylate (8)

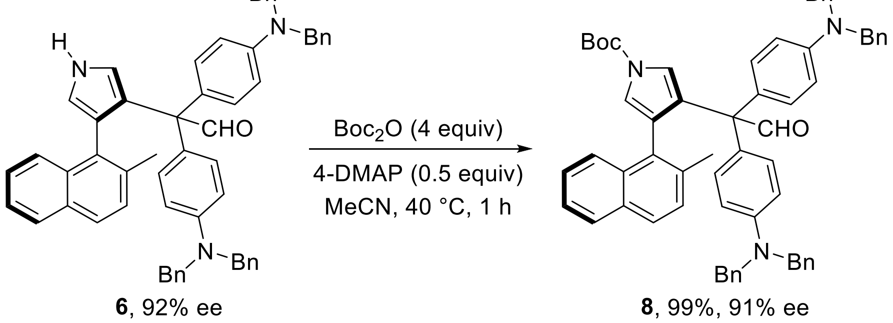

6, 92% ee **8**, 99%, 91% ee

Compound **8** was prepared in 99% yield (88.3 mg, 0.1 mmol scale) according to the known procedure[12] as a brown solid (mp 104–105 °C, eluent: PE/EtOAc = 10/1). $[\alpha]_D^{20}$ = -62.1° (c = 1.0, $CHCl_3$). 91% ee (determined by HPLC: Chiralpak IE Column, 10/90 *i*-PrOH/hexane, 1.0 mL/min, 254 nm; TR = 10.23 min (major), 11.42 min (minor)). ^{1}H NMR (400 MHz, $CDCl_3$) δ 9.34 (s, 1H), 7.65 (d, *J* = 8.0 Hz, 1H), 7.56 (d, *J* = 8.4 Hz, 1H), 7.35 – 7.30 (m, 11H), 7.25 – 7.18 (m, 12H), 7.10 (d, *J* = 8.4 Hz, 1H), 7.05 – 7.00 (m, 2H), 6.78 (d, *J* = 8.8 Hz, 2H), 6.67 (d, *J* = 8.8 Hz, 2H), 6.42 (d, *J* = 7.6 Hz, 4H), 4.57 (s, 4H), 4.53 (s, 4H), 1.93 (s, 3H), 1.59 (s, 9H); ^{13}C NMR (100 MHz, $CDCl_3$) δ 196.5, 148.7, 147.9, 147.8, 138.6, 135.5, 133.9, 131.4, 131.1, 131.0, 130.8, 128.9, 128.6, 128.5, 128.1, 127.4, 127.1(3), 127.0(6), 126.9(1), 126.8(6), 126.8, 126.6(8), 126.6(5), 126.3, 125.0, 124.5, 124.3, 121.6, 119.6, 111.5, 83.8, 63.7, 54.1, 28.0, 21.2; IR (neat): 2923(bs), 1741(s), 1608, 1515, 1362, 1251, 1154, 1028, 731, 696 cm^{-1}; HRESIMS Calcd for $[C_{62}H_{57}N_3NaO_3]^+$ (M + Na^+) 914.4292, found 914.4259.

(*R*)-2,2-bis(4-(dibenzylamino)phenyl)-2-(4-(2-methylnaphthalen-1-yl)-1-(phenylsulfonyl)-1*H*-pyrrol-3-yl)ethan-1-ol (9)

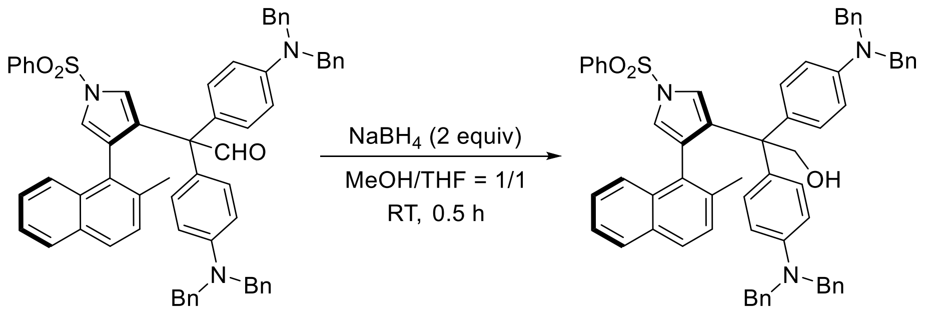

5a, 92% ee **9**, 97%, 92% ee

To a stirred solution of aldehyde **5a** (93.2 mg, 0.1 mmol) in MeOH (1 mL) and THF (1 mL) was added $NaBH_4$ (7.6 mg, 0.2 mmol) slowly at 0 °C. After stirring at room temperature for 0.5 h, the reaction was quenched with aqueous NH_4Cl and filtered. The mixture was then extracted with EtOAc, washed with H_2O and brine, dried over anhydrous $MgSO_4$, and concentrated under reduced pressure. The residue was purified by column chromatography on silica gel (PE/EtOAc = 5/1) to afford the desired alcohol **9** in 97% yield (90.5 mg) as a colorless solid (mp 108–109 °C). $[\alpha]_D^{20}$

= -39.2° (c = 1.0, $CHCl_3$). 92% ee (determined by HPLC: Chiralpak IE Column, 50/50 *i*-PrOH/hexane, 1.0 mL/min, 254 nm; TR = 13.26 min (major), 25.09 min (minor)). ^{1}H NMR (400 MHz, $CDCl_3$) δ 7.78 – 7.71 (m, 2H), 7.67 (d, *J* = 8.0 Hz, 1H), 7.61 (d, *J* = 8.4 Hz, 1H), 7.42 – 7.38 (m, 1H), 7.37 – 7.28 (m, 10H), 7.27 – 7.21 (m, 13H), 7.10 (d, *J* = 8.4 Hz, 1H), 7.02 (d, *J* = 2.4 Hz, 1H), 7.01 – 6.95 (m, 1H), 6.87 (d, *J* = 2.0 Hz, 1H), 6.85 (s, 1H), 6.75 (d, *J* = 8.8 Hz, 2H), 6.72 (d, *J* = 9.2 Hz, 2H), 6.50 – 6.39 (m, 4H), 4.66 – 4.51 (m, 8H), 3.59 (d, *J* = 2.0 Hz, 2H), 1.53 (s, 3H); ^{13}C NMR (100 MHz, $CDCl_3$) δ 147.6, 147.2, 138.9, 138.7(3), 138.7(0), 137.4, 135.5, 133.7, 133.6, 131.4, 130.7, 130.5, 130.4, 130.3, 130.0, 129.0, 128.5(9), 128.5(6), 128.1, 127.8, 127.4, 127.3, 126.9, 126.8, 126.6, 126.4, 125.9, 125.3, 124.4, 122.2, 121.5, 111.7, 111.6, 67.8, 54.3(1), 54.2(8), 53.7, 19.9; IR (neat): 3059(bs), 1609, 1515, 1369, 1234, 1175, 1062, 728, 597 cm^{-1}; HRESIMS Calcd for $[C_{63}H_{55}N_3NaO_3S]^+$ (M + Na^+) 956.3856, found 956.3845.

(*R*)-4,4'-(2-(benzylamino)-1-(4-(2-methylnaphthalen-1-yl)-1-(phenylsulfonyl)-1*H*-pyrrol-3-yl)ethane-1,1-diyl)bis(*N*,*N*-dibenzylaniline) (10)

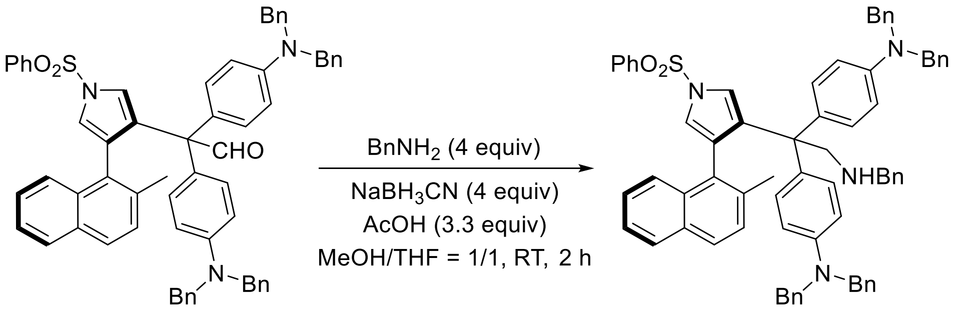

5a, 92% ee

10, 53%, 93% ee

To a stirred solution of aldehyde **5a** (93.2 mg, 0.1 mmol) in MeOH (1 mL) and THF (1 mL) were added benzylamine (44 μL, 0.4 mmol), sodium cyanoborohydride (25.1 mg, 0.4 mmol) and acetic acid (19 μL, 0.33 mmol) sequentially at room temperature. The reaction was stirred at room temperature for 2 h and the progress of the reaction was monitored by TLC. Upon completion, the mixture was diluted with water, extracted EtOAc, washed with aqueous $NaHCO_3$ and brine, dried over anhydrous $MgSO_4$ and concentrated. The residue was purified by column chromatography on

silica gel (PE/EtOAc = 5/1) to afford the desired product **10** in 53% yield (54.2 mg) as a colorless solid (mp 99–100 °C). $[\alpha]_D^{20}$ = -23.6° (c = 1.0, $CHCl_3$). 93% ee (determined by HPLC: Chiralpak IE Column, 10/90 *i*-PrOH/hexane, 1.0 mL/min, 254 nm; TR = 19.31 min (major), 21.49 min (minor)). ^{1}H NMR (400 MHz, $CDCl_3$) δ 7.72 – 7.69 (m, 3H), 7.61 (d, *J* = 8.4 Hz, 1H), 7.37 – 7.31 (m, 10H), 7.29 – 7.26 (m, 8H), 7.24 – 7.20 (m, 6H), 7.10 – 7.03 (m, 4H), 6.98 – 6.94 (m, 2H), 6.86 – 6.81 (m, 2H), 6.73 (d, *J* = 8.4 Hz, 2H), 6.69 (d, *J* = 8.4 Hz, 2H), 6.58 (d, *J* = 6.8 Hz, 2H), 6.46 – 6.39 (m, 4H), 4.63 – 4.55 (m, 8H), 3.06 – 2.99 (m, 2H), 2.66 – 2.54 (m, 2H), 1.40 (s, 3H); ^{13}C NMR (100 MHz, $CDCl_3$) δ 147.2, 146.9, 140.1, 139.4, 139.0, 138.9(4), 138.8(9), 135.6, 133.8, 133.5, 132.2, 131.7, 131.3, 130.5, 130.3, 130.2, 129.1, 128.9, 128.6, 128.5, 128.0, 127.9, 127.5, 127.4, 127.2, 126.8(4), 126.8(0), 126.7, 126.4, 126.2(3), 126.2(1), 125.2, 124.3, 121.9, 121.4, 111.6, 55.7, 54.3(2), 54.2(8), 53.5, 51.7, 19.8; IR (neat): 3028(bs), 1609, 1515, 1362, 1233, 1182, 1091, 728, 595 cm^{-1}; HRESIMS Calcd for $[C_{70}H_{62}N_4NaO_2S]^+$ (M + Na^+) 1045.4486, found 1045.4458.

(*R*)-(*E*)-4,4'-(2-(2-(2,4-dinitrophenyl)hydrazono)-1-(4-(2-methylnaphthalen-1-yl)-1-(phenylsulfonyl)-1*H*-pyrrol-3-yl)ethane-1,1-diyl)bis(*N*,*N*-dibenzylaniline) (11)

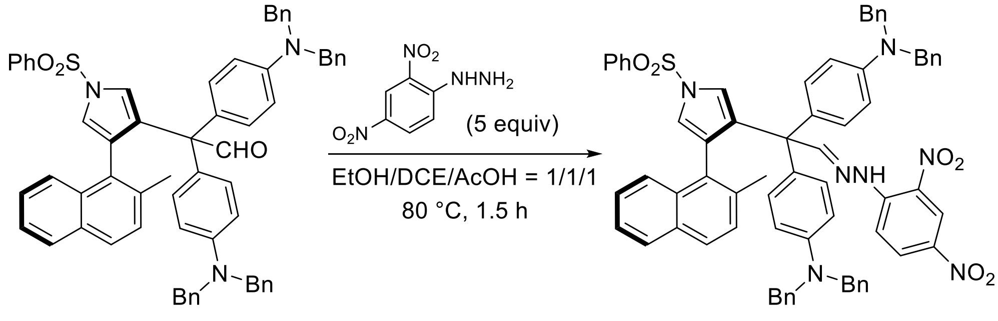

5a, 92% ee

11, 88%, 92% ee

To a solution of compound **5a** (93.2 mg, 0.1 mmol) in EtOH (0.5 mL), DCE (0.5 mL) and AcOH (0.5 mL) was added (2,4-dinitrophenyl)hydrazine (99.1 mg, 0.5 mmol) at room temperature. The mixture was stirred at 80 °C for 1.5 h and the progress of the reaction was monitored by TLC. Upon completion, the mixture was diluted with water, extracted EtOAc, washed with aqueous $NaHCO_3$ and brine, dried over anhydrous $MgSO_4$ and concentrated. The residue was purified by column

chromatography on silica gel (PE/EtOAc = 5/1) to afford the desired product **11** in 88% yield (97.9 mg) as a colorless solid (mp 228–229 °C). $[\alpha]_D^{20}$ = +53.9° (c = 1.0, $CHCl_3$). 92% ee (determined by HPLC: Chiralpak IC Column, 30/70 *i*-PrOH/hexane, 1.0 mL/min, 254 nm; TR = 43.41 min (major), 35.71 min (minor)). ^1H NMR (400 MHz, $CDCl_3$) δ 9.16 (s, 1H), 8.93 (d, *J* = 2.4 Hz, 1H), 7.93 (dd, *J* = 9.6, 2.4 Hz, 1H), 7.81 – 7.73 (m, 2H), 7.65 (d, *J* = 8.4 Hz, 1H), 7.55 (d, *J* = 8.0 Hz, 1H), 7.49 – 7.44 (m, 1H), 7.40 – 7.31 (m, 10H), 7.31 – 7.23 (m, 11H), 7.17 (d, *J* = 8.4 Hz, 1H), 7.04 (d, *J* = 9.6 Hz, 1H), 7.02 – 6.95 (m, 2H), 6.92 – 6.82 (m, 3H), 6.68 (d, *J* = 8.8 Hz, 2H), 6.63 (d, *J* = 8.8 Hz, 2H), 6.60 – 6.47 (m, 5H), 4.69 (s, 2H), 4.68 (s, 2H), 4.66 (s, 2H), 4.65 (s, 2H), 1.71 (s, 3H); ^{13}C NMR (100 MHz, $CDCl_3$) δ 154.7, 147.8, 147.7, 144.6, 138.7, 138.6, 138.4, 137.4, 136.7, 135.3, 133.8, 133.2, 131.5, 131.0, 130.8, 130.2(9), 130.2(5), 129.3(4), 129.2(7), 129.1, 128.7, 128.6, 128.5, 128.4, 127.6, 127.0, 126.7, 126.6, 126.5, 125.8, 125.6, 124.3, 123.5, 122.9, 120.9, 116.5, 111.8, 111.0, 55.8, 54.5, 54.4, 20.4; IR (neat): 3040(bs), 1614(s), 1515, 1451, 1332, 1182, 1058, 732, 565 cm^{-1}; HRESIMS Calcd for $[C_{69}H_{57}N_7NaO_6S]^+$ (M + Na^+) 1134.3983, found 1134.3955.

(*R*)-4,4'-(1-(4-(2-methylnaphthalen-1-yl)-1-(phenylsulfonyl)-1*H*-pyrrol-3-yl)prop-2-ene-1,1-diyl)bis(*N*,*N*-dibenzylaniline) (12)

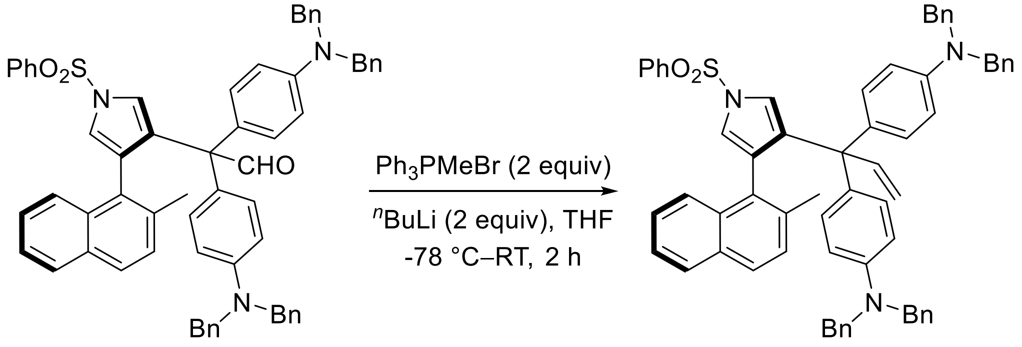

5a, 92% ee **12**, 99%, 92% ee

To a dry Schlenk bottle equipped with a stir bar were added Ph_3PMeBr (357.2 mg, 1 mmol) and THF (8 mL). After cooling to -78 °C, *ⁿ*BuLi (2.5 M in hexane, 0.4 mL, 1 mmol) was added to the solution at -78 °C and stirred at 0 °C for 1 h. Then the solution of **5a** (466.1 mg, 0.5 mmol) in THF (3 mL) was added to the reaction dropwise at -78 °C, and stirred at room temperature for another 1 h. The progress of

the reaction was monitored by TLC. Upon completion, the reaction was quenched with H_2O, extracted with EtOAc, dried over $MgSO_4$, and concentrated under reduced pressure. The residue was purified by column chromatography on silica gel (PE/EtOAc = 10/1) to afford the desired product **12** in 99% yield (460.4 mg) as a colorless solid (mp 140–141 °C). $[\alpha]_D^{20}$ = -26.1° (c = 1.0, $CHCl_3$). 92% ee (determined by HPLC: Chiralpak IE Column, 10/90 *i*-PrOH/hexane, 1.0 mL/min, 254 nm; TR = 10.29 min (major), 12.79 min (minor)). ^{1}H NMR (400 MHz, $CDCl_3$) δ 7.74 (d, *J* = 7.6 Hz, 2H), 7.66 (d, *J* = 8.0 Hz, 1H), 7.62 (d, *J* = 8.4 Hz, 1H), 7.42 – 7.37 (m, 1H), 7.36 – 7.29 (m, 9H), 7.28 – 7.21 (m, 14H), 7.15 (d, *J* = 8.4 Hz, 1H), 6.94 – 6.87 (m, 3H), 6.83 (d, *J* = 8.4 Hz, 1H), 6.61 (d, *J* = 8.8 Hz, 2H), 6.57 (d, *J* = 8.4 Hz, 2H), 6.45 (d, *J* = 8.4 Hz, 2H), 6.39 (d, *J* = 8.4 Hz, 2H), 5.71 (dd, *J* = 17.2, 10.8 Hz, 1H), 4.61 – 4.52 (m, 9H), 4.28 (d, *J* = 16.0 Hz, 1H), 1.73 (s, 3H); ^{13}C NMR (100 MHz, $CDCl_3$) δ 147.3, 147.2, 142.7, 138.9, 138.8, 137.7, 135.3, 133.6, 133.5, 132.6, 132.4, 131.4, 131.1, 130.6, 130.4, 129.0, 128.6, 128.1, 127.8, 127.6, 127.0, 126.8, 126.7, 126.6(3), 126.5(9), 125.0, 124.3, 122.8, 121.4, 115.5, 111.3, 111.2, 55.9, 54.4, 20.5; IR (neat): 3030(bs), 1608, 1514, 1361, 1233, 1182, 1065, 728, 594 cm^{-1}; HRESIMS Calcd for $[C_{64}H_{55}N_3NaO_2S]^+$ (M + Na$^+$) 952.3907, found 952.3888.

(*R*)-4,4'-(1-(5-bromo-4-(2-methylnaphthalen-1-yl)-1*H*-pyrrol-3-yl)prop-2-ene-1,1-diyl)bis(*N*,*N*-dibenzylaniline) (13)

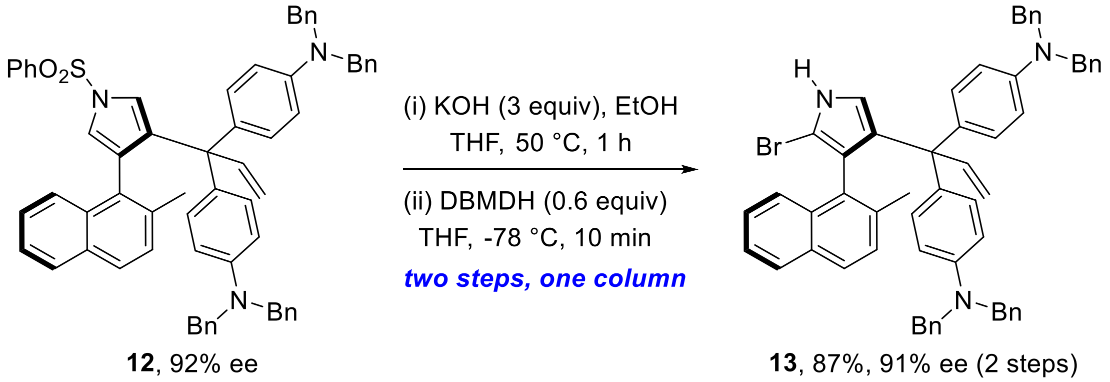

To a solution of compound **12** (460.4 mg, 0.5 mmol) in THF (4 mL) and EtOH (4 mL) was added KOH (84.2 mg, 1.5 mmol), and the mixture was stirred at 50 °C for 1 h. The progress of the reaction was monitored by TLC. Upon completion, the reaction

was diluted with water, extracted with EtOAc, washed with brine, dried over $MgSO_4$, and filtered. The filtrate was concentrated under reduced pressure to afford the crude free pyrrole without further purification. To the solution of above pyrrole product in THF (5 mL) was added a solution of 1,3-dibromo-5,5-dimethylhydantoin (85.8 mg, 0.3 mmol) in THF (3 mL) at -78 °C and stirred for 10 min. The progress of the reaction was monitored by TLC. Upon completion, the mixture was filtered and concentrated under reduced pressure. The residue was purified by column chromatography on silica gel (PE/EtOAc = 5/1) to afford brominated pyrrole **13** in 87% yield (2 steps, 378.0 mg) as a brown solid (mp 94–95 °C)[13]. $[\alpha]_D^{20}$ = +11.3° (c = 1.0, $CHCl_3$). 91% ee (determined by HPLC: Chiralpak ADH Column, 5/95 *i*-PrOH/hexane, 1.0 mL/min, 254 nm; TR = 19.24 min (major), 14.77 min (minor)). ^1H NMR (400 MHz, $CDCl_3$) δ 8.12 (d, *J* = 1.6 Hz, 1H), 7.72 – 7.65 (m, 2H), 7.33 – 7.27 (m, 10H), 7.25 – 7.20 (m, 13H), 7.05 – 7.00 (m, 1H), 6.82 – 6.76 (m, 4H), 6.53 – 6.45 (m, 5H), 5.91 (dd, *J* = 16.8, 10.4 Hz, 1H), 4.62 (d, *J* = 10.8 Hz, 1H), 4.57 (s, 8H), 4.35 (d, *J* = 17.2 Hz, 1H), 1.91 (s, 3H); ^{13}C NMR (100 MHz, $CDCl_3$) δ 147.2, 147.1, 144.0, 139.0, 138.9, 136.1, 133.9, 133.8, 132.2, 131.7, 131.5, 130.9, 130.6, 128.5(0), 128.5(0), 128.2, 127.3, 127.1, 126.9, 126.8(0), 126.7(9), 126.7(6), 124.9, 124.2, 120.3, 120.1, 114.4, 111.2, 111.1, 99.6, 56.0, 54.2(8), 54.2(6), 20.6; IR (neat): 3205(bs), 1608, 1515, 1360, 1264, 1174, 1027, 742, 696 cm^{-1}; HRESIMS Calcd for $[C_{58}H_{50}BrN_3Na]^+$ (M + Na^+) 890.3080, found 890.3043.

(*R*)-4,4'-(1-(5-(diphenylphosphino)-1-methyl-4-(2-methylnaphthalen-1-yl)-1*H*-pyrrol-3-yl)prop-2-ene-1,1-diyl)bis(*N*,*N*-dibenzylaniline) (14)

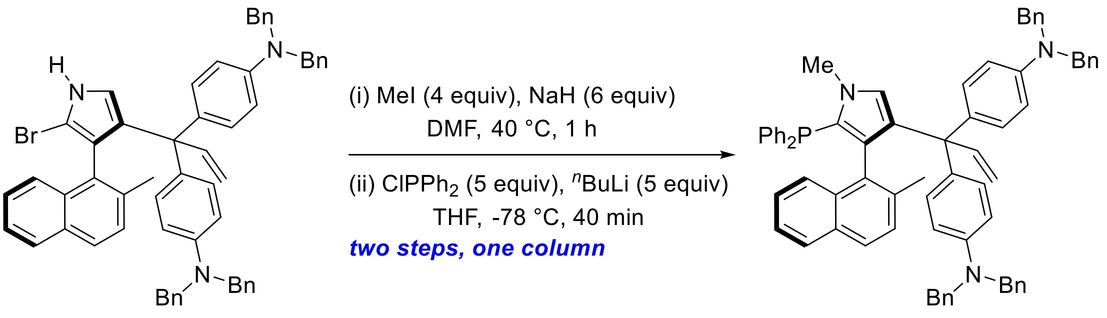

13, 91% ee

14, 56%, 92% ee, 2 steps
(98% ee after recrystalization)

To a dry flask equipped with a stir bar were added compound **13** (378.0 mg, 0.44 mmol) and DMF (8 mL). After cooling to 0 °C, NaH (104.4 mg, 2.61 mmol) was added to the solution and stirred at room temperature for 0.5 h. Then methyl iodide (0.11 mL, 1.74 mmol) was added and stirred at 40 °C for another 0.5 h. The progress of the reaction was monitored by TLC. Upon completion, the reaction was quenched with H_2O, extracted with EtOAc, dried over $MgSO_4$, and filtered. The filtrate was concentrated under reduced pressure to afford the crude methylated pyrrole without further purification[14]. To a dry Schlenk tube equipped with a stir bar were added above crude product and THF (8 mL) under N_2 atmosphere. After cooling to -78 °C, ^{n}BuLi (2.5 M in hexane, 0.87 mL, 2.175 mmol) was added to the solution and stirred at this temperature for 30 min. Then chlorodiphenylphosphine (0.39 mL, 2.175 mmol) was added dropwise at -78 °C and stirred for 10 min. The progress of the reaction was monitored by TLC. Upon completion, the reaction was quenched with H_2O, extracted with EtOAc, dried over $MgSO_4$ and concentrated under reduced pressure. The residue was purified by column chromatography on silica gel (PE/EtOAc = 20/1) to afford brominated pyrrole **14** in 56% yield (2 steps, 229.7 mg) as a colorless solid (mp 91–92 °C)[15]. $[\alpha]_D^{20}$ = -31.9° (c = 1.0, $CHCl_3$). 92% ee (determined by HPLC: Chiralpak ADH-3 Column, 1/99 *i*-PrOH/hexane, 1.0 mL/min, 230 nm; TR = 10.38 min (major), 7.30 min (minor)). ^{1}H NMR (400 MHz, $CDCl_3$) δ 7.66 (d, *J* = 8.0 Hz, 1H), 7.61 (d, *J* = 8.4 Hz, 1H), 7.32 – 7.27 (m, 9H), 7.25 – 7.20 (m, 16H), 7.18 – 7.11 (m, 8H), 7.05 – 7.00 (m, 1H), 6.85 (d, *J* = 8.8 Hz, 2H), 6.81 (d, *J* = 8.8 Hz, 2H), 6.60 (s, 1H), 6.52 (d, *J* = 8.8 Hz, 2H), 6.47 (d, *J* = 8.8 Hz, 2H), 5.96 (dd, *J* = 16.8, 10.4 Hz, 1H), 4.58 – 4.52 (m, 9H), 4.31 (dd, *J* = 17.2, 2.0 Hz, 1H), 3.10 (s, 3H), 1.88 (s, 3H); ^{13}C NMR (125 MHz, $CDCl_3$) δ 147.1, 147.0, 144.5, 139.0, 138.9, 137.1, 136.5, 136.0, 134.8, 134.5(9), 134.5(6), 134.4, 134.2, 131.7(4), 131.6(7), 131.4, 130.8, 130.6, 130.1, 129.5, 128.5, 128.2, 128.0, 127.9, 127.8, 127.4, 127.0, 126.9, 126.7(8), 126.7(7), 126.7, 124.4, 123.9, 121.9, 113.8, 111.2, 111.1, 55.6, 54.2, 54.1, 36.9, 21.3; ^{31}P NMR (200 MHz, $CDCl_3$) δ -33.4; IR (neat): 3021(bs), 1608, 1514, 1360, 1364, 1184, 1027, 734, 696 cm^{-1}; HRESIMS Calcd for $[C_{71}H_{62}N_3NaP]^+$ (M + Na^+) 1010.4574, found 1010.4539.

methyl (1*R*,3*S*,3a*R*,6a*S*)-3-(4-bromophenyl)-4,6-dioxo-5-phenyloctahydropyrrolo[3,4-*c*]pyrrole-1-carboxylate (17)

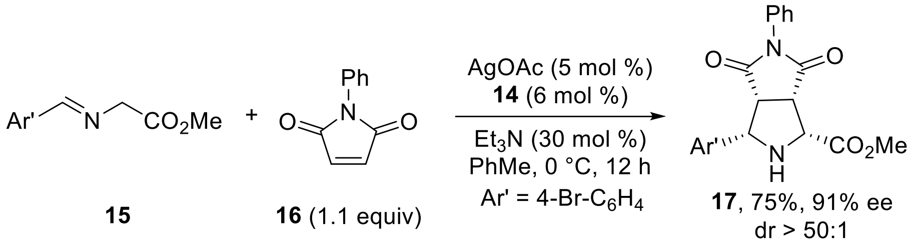

Compound **17** was prepared in 75% yield (48.3 mg, 0.15 mmol scale) according to the known procedure by replacing ligand with compound **14** (98% ee). This compound is known and the spectroscopic data match those reported.[16] $[\alpha]_D^{20}$ = +33.9° (c = 1.0, $CHCl_3$). 91% ee (determined by HPLC: Chiralpak ASH Column, 50/50 *i*-PrOH/hexane, 1.0 mL/min, 254 nm; TR = 11.35 min (major), 27.82 min (minor)). ^{1}H NMR (400 MHz, $CDCl_3$) δ 7.50 – 7.44 (m, 2H), 7.43 – 7.37 (m, 2H), 7.36 – 7.30 (m, 3H), 7.16 – 7.10 (m, 2H), 4.55 (d, *J* = 8.8 Hz, 1H), 4.13 (d, *J* = 6.4 Hz, 1H), 3.86 (s, 3H), 3.75 – 3.68 (m, 1H), 3.58 – 3.51 (m, 1H), 2.48 (s, 1H); ^{13}C NMR (100 MHz, $CDCl_3$) δ 174.9, 173.4, 169.9, 135.7, 131.6, 131.5, 129.1, 128.8, 128.6, 126.0, 122.3, 63.5, 61.8, 52.4, 49.0, 48.0.

dimethyl (*R*,*E*)-2-(1,3-diphenylallyl)malonate (20)

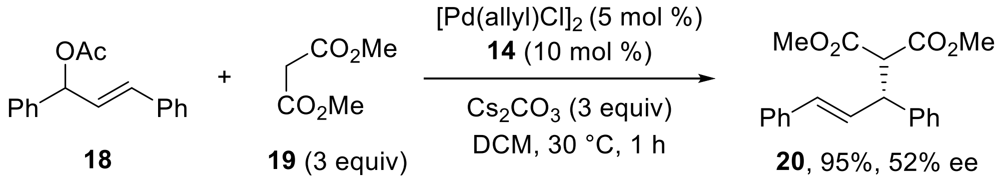

Compound **20** was prepared in 95% yield (61.6 mg, 0.2 mmol scale) according to the known procedure by replacing ligand with compound **14** (98% ee). This compound is known and the spectroscopic data match those reported.[17] $[\alpha]_D^{20}$ = -193.5° (c = 1.0, $CHCl_3$). 52% ee (determined by HPLC: Chiralpak IA Column, 10/90 *i*-PrOH/hexane, 1.0 mL/min, 254 nm; TR = 8.79 min (major), 11.12 min (minor)). ^{1}H NMR (400 MHz, $CDCl_3$) δ 7.34 – 7.27 (m, 7H), 7.25 – 7.17 (m, 3H), 6.51 – 6.44 (m, 1H), 6.33 (dd, *J* =

15.6, 8.4 Hz, 1H), 4.30 – 4.22 (m, 1H), 3.95 (d, *J* = 10.8 Hz, 1H), 3.70 (s, 3H), 3.51 (s, 3H); ^{13}C NMR (100 MHz, $CDCl_3$) δ 168.2, 167.8, 140.2, 136.8, 131.8, 129.1, 128.7, 128.4, 127.8, 127.5, 127.1, 126.4, 57.6, 52.6, 52.4, 49.2.

1.6 Crystal Data

Supplementary Table 3. X-ray structure of compound **3b**. CCDC Number = 2235694.

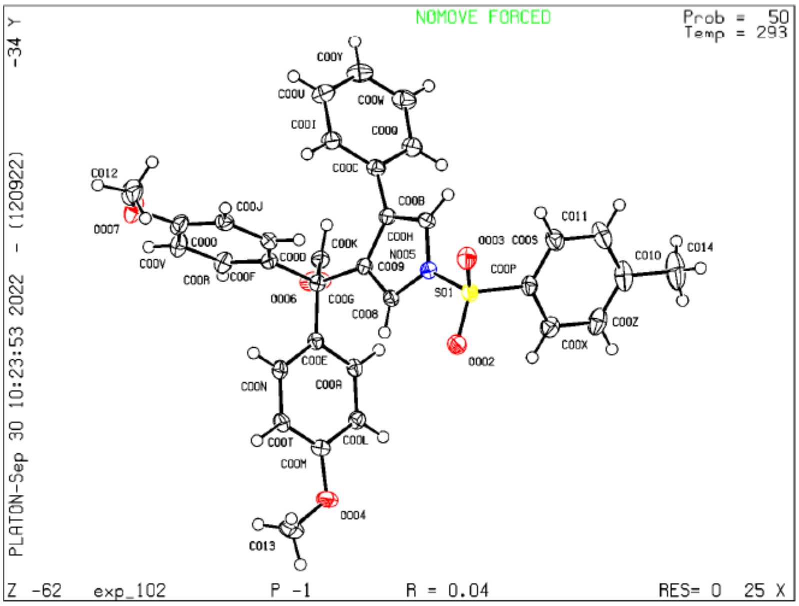

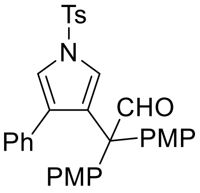

3b

Bond precision: C-C = 0.0024 A Wavelength=1.54184

Cell:	a=9.5278(2)	b=11.8083(3)	c=13.7109(4)
	alpha=69.583(2)	beta=78.003(2)	gamma=84.297(2)
Temperature:	293 K		

	Calculated	Reported
Volume	1413.48(7)	1413.49(7)
Space group	P -1	P -1
Hall group	-P 1	-P 1
Moiety formula	C33 H29 N O5 S	C33 H29 N O5 S
Sum formula	C33 H29 N O5 S	C33 H29 N O5 S
Mr	551.63	551.63
Dx,g cm-3	1.296	1.296
Z	2	2
Mu (mm-1)	1.365	1.365
F000	580.0	580.0
F000'	582.35	
h,k,lmax	11,14,16	11,14,16
Nref	5330	5059
Tmin,Tmax	0.872,0.872	0.835,1.000
Tmin'	0.872	

Correction method= # Reported T Limits: Tmin=0.835 Tmax=1.000
AbsCorr = MULTI-SCAN

Data completeness= 0.949 Theta(max)= 69.523

R(reflections)= 0.0426(4530) wR2(reflections)= 0.1232(5059)

S = 1.066 Npar= 364

Supplementary Table 4. X-ray structure of compound **11**. CCDC Number = 2235698.

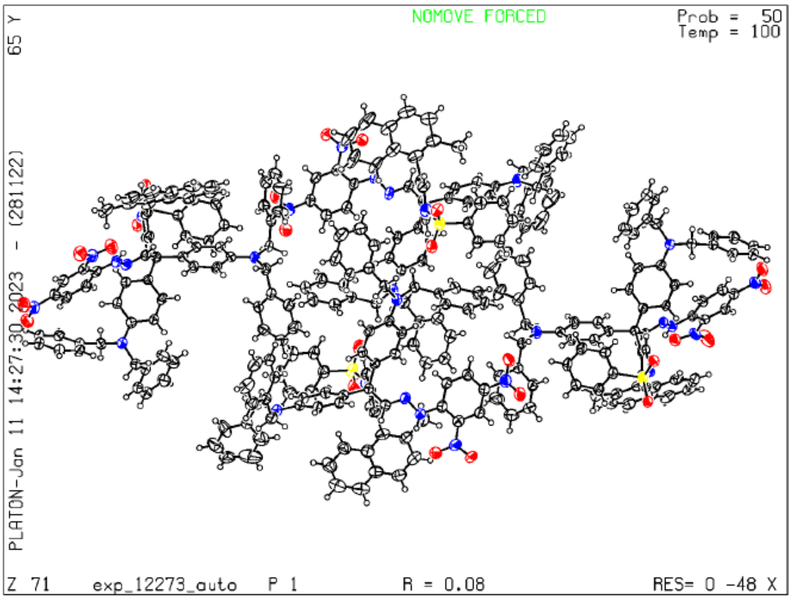

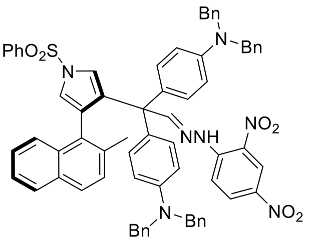

11

Bond precision: C-C = 0.0100 A Wavelength=1.54184

Cell:	a=10.0142(1)	b=21.0096(3)	c=27.1568(3)
	alpha=83.652(1)	beta=89.060(1)	gamma=88.254(1)
Temperature:	100 K		

	Calculated	Reported
Volume	5675.48(12)	5675.48(12)
Space group	P 1	P 1
Hall group	P 1	P 1
Moiety formula	C69 H57 N7 O6 S	C69 H57 N7 O6 S
Sum formula	C69 H57 N7 O6 S	C69 H57 N7 O6 S
Mr	1112.28	1112.27
Dx,g cm-3	1.302	1.302
Z	4	4
Mu (mm-1)	1.004	1.004
F000	2336.0	2336.0
F000'	2343.99	
h,k,lmax	12,25,33	12,25,33
Nref	43534[21767]	39275
Tmin,Tmax		0.224,1.000
Tmin'		

Correction method= # Reported T Limits: Tmin=0.224 Tmax=1.000
AbsCorr = MULTI-SCAN

Data completeness= 1.80/0.90 Theta(max)= 70.577

R(reflections)= 0.0780(33814) wR2(reflections)= 0.2417(39275)

S = 1.047 Npar= 2825

1.7 DFT calculations

All calculations were performed using Gaussian 16 package[18]. Geometry optimizations and vibrational analysis were conducted under the B3LYP-D3 level of theory[19,20] with the LANL2DZ basis set[21,22] for Cu atom, and the 6-31G(d) basis set[23] for C, H, O, N and S atom. Electronic energy of all the intermediates and transition states were recomputed at the B3LYP-D3/def2TZVP[24, 25] level of theory. All local minimums were confirmed with no imaginary frequency and all transition states had only one imaginary frequency. And every transition state was checked by intrinsic reaction coordinate (IRC) analysis. The SMD solvation model[26] with dichloroethane was used for all calculations. The ball stick models of molecules were drawn by CYLview 2.0[27].

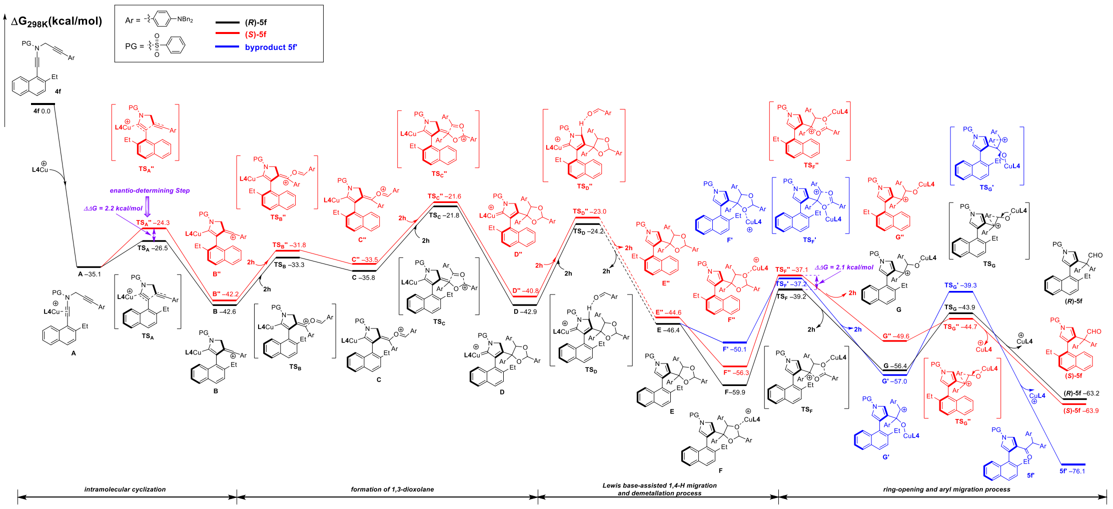

Supplementary Figure 5. Plausible reaction mechanism. Relative free energies (ΔG, in kcal/mol) of all the transition states and intermediates were computed at the SMD(dichloroethane)-B3LYP-D3/def2TZVP//SMD(dichloroethane)-B3LYP-D3/6-31 G(d)/LANL2DZ level of theory.

To probe the reaction mechanism, density functional theory (DFT) calculations were performed with diyne **4f**, aldehyde **2h** and **L4**-ligated copper(I) complex as the model. As shown in Supplementary Figure 5, the reaction begins with the coordination of BOX-ligated copper species to diyne **4f** to form intermediate **A**, followed by intramolecular cyclization to give the vinyl cation intermediate **B** via

transition state **TS$_A$** with a free energy barrier of 8.6 kcal/mol. Nucleophilic addition of aldehyde onto vinyl cation intermediate **B** affords vinyl copper intermediate **C** which is considered as the resonance form of carbonyl ylide. Then the second nucleophilic addition of aldehyde onto the oxonium ion of intermediate **C** delivers copper carbene intermediate **D** bearing a 1,3-dioxolane moiety. Subsequently, Lewis base-assisted 1,4-proton transfer and demetallation take place to give 1,3-dioxolane intermediate **E**, which has been isolated in the control experiments.

Due to the coordination of copper with two different oxygen atoms on 1,3-dioxolane unit, intermediate **F** and **F′** undergo two different pathways in subsequent steps. Starting from intermediate **F**, ring opening of 1,3-dioxolane leads to carbocation intermediate **F** with a free energy barrier of 20.7 kcal/mol, which finally generates the desired naphthylpyrrole (*R*)-**5f** with the exclusion of aldehyde **2h**. Alternatively, inspection into the ring-opening step of intermediate **F′** indicates that copper(I) catalyst undergoes a more thermally unfavorable process to generate intermediate **F′** (Cu-O distance: 3.24 Å, which shows this coordination is difficult due to large steric hindrance) than intermediate **F** (Cu-O distance: 2.00 Å). Also, the copper coordinated intermediate **F′** (-50.1 kcal/mol) has a much higher free energy compared with intermediate **F** (-59.9 kcal/mol). Therefore, the generation of ketone product **5f′** is less likely and the regioselective formation of homologated aldehyde (*R*)-**5f** is thermodynamically preferred. In addition, the detailed reaction mechanism for the formation of (*S*)-**5f** was also calculated and shown in Supplementary Figure 5 (red line).

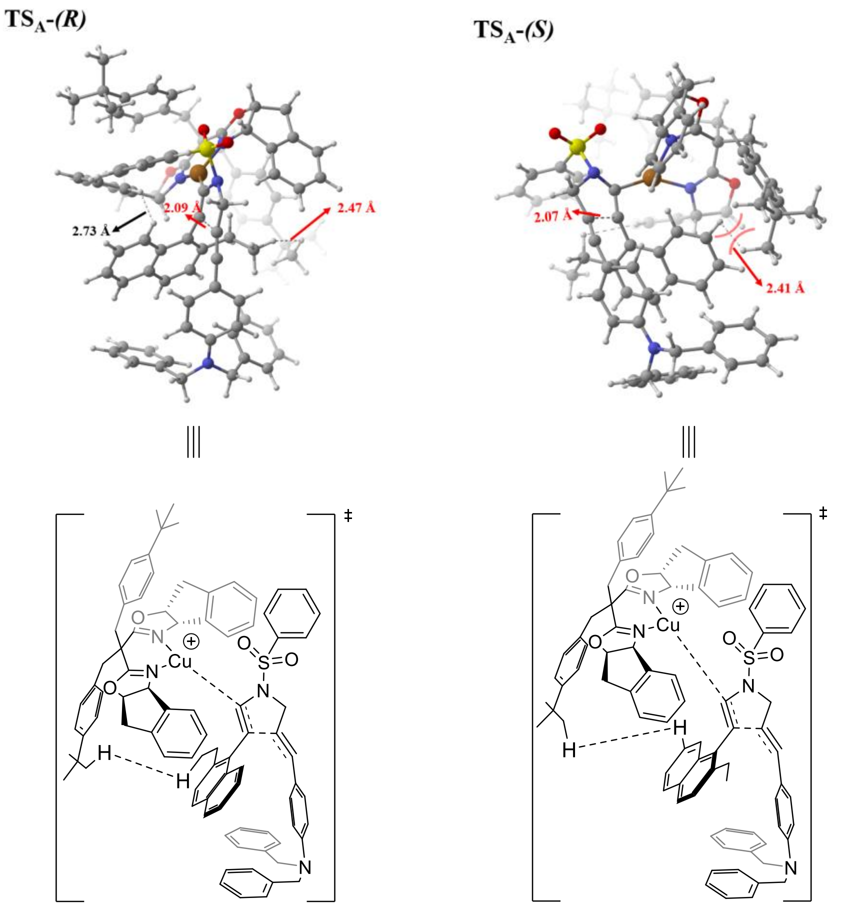

ΔΔG = 0.0 kcal/mol **ΔΔG = 2.2 kcal/mol**

theoretically predicted *ee* value: 96.6%

experimental *ee* value: 94.0%

Supplementary Figure 6. Geometries and relative free energies (ΔΔG, in kcal/mol) of enantioselectivity-determining transition states **TS$_A$-(*R*)** and **TS$_A$-(*S*)** in the cyclization step (intermediate **A** to **B**) computed at SMD(dichloroethane)-B3LYP-D3/def2TZVP//SMD(dichloroethane)-B3LYP-D3/6-31 G(d)/LANL2DZ level of theory.

In order to illustrate the origin of enantioselectivity, SaBOX coordinated Cu(I) complex was used in the enantioselectivity-determining intramolecular cyclization step (intermediate **A** to intermediate **B**). As shown in Supplementary Figure 6, the free energy of transition state **TS$_A$-(*R*)** (leading to the major enantiomer) is predicted to be 2.2 kcal/mol lower than **TS$_A$-(*S*)** (leading to the minor enantiomer), which leads to the theoretically predicted enantioselectivity (96.6% ee). Thus, the experimental enantioselectivity (94% ee) matches well with theoretical results. This result is

consistent with the free energy difference (from intermediate **F** to **G**) for *R* and *S* configurations in the rate-determining step (2.1 kcal/mol, shown in Supplementary Figure 5). Mechanistically, **TS$_{A}$-(*S*)** has stronger steric repulsion and lower stability than **TS$_{A}$-(*R*)**, including the shorter C···C distance for the bond-forming position (2.07 Å for **TS$_{A}$-(*S*)** *versus* 2.09 Å for **TS$_{A}$-(*R*)**), as well as shorter distance of ligand **L4** with naphthyl group on **TS$_{A}$-(*S*)** (closest distance: 2.41 Å for **TS$_{A}$-(*S*)** *versus* 2.47 Å for **TS$_{A}$-(*R*)**). Thus, the observed enantioselectivity originates from steric effects.

1.8 NMR Spectra

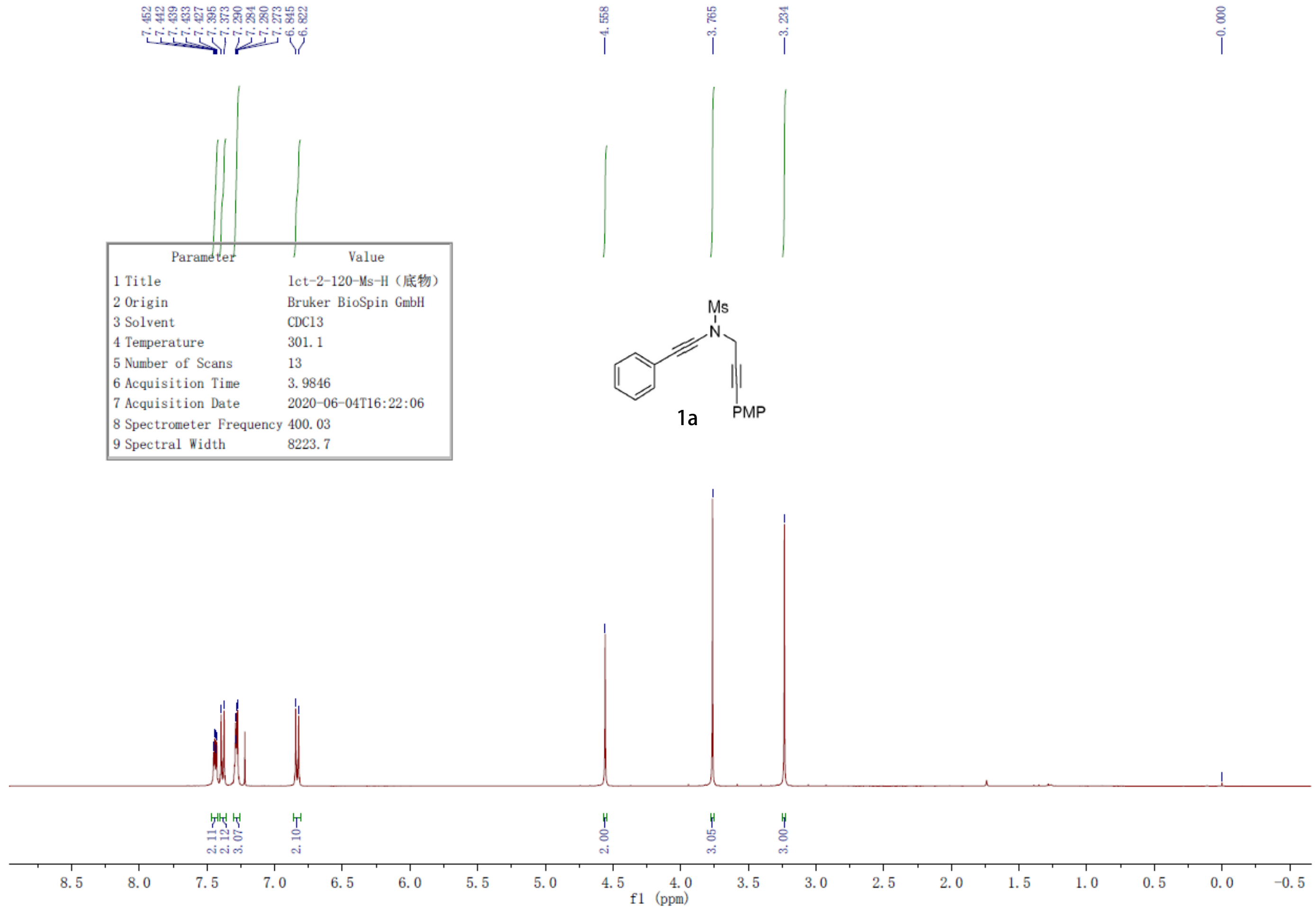

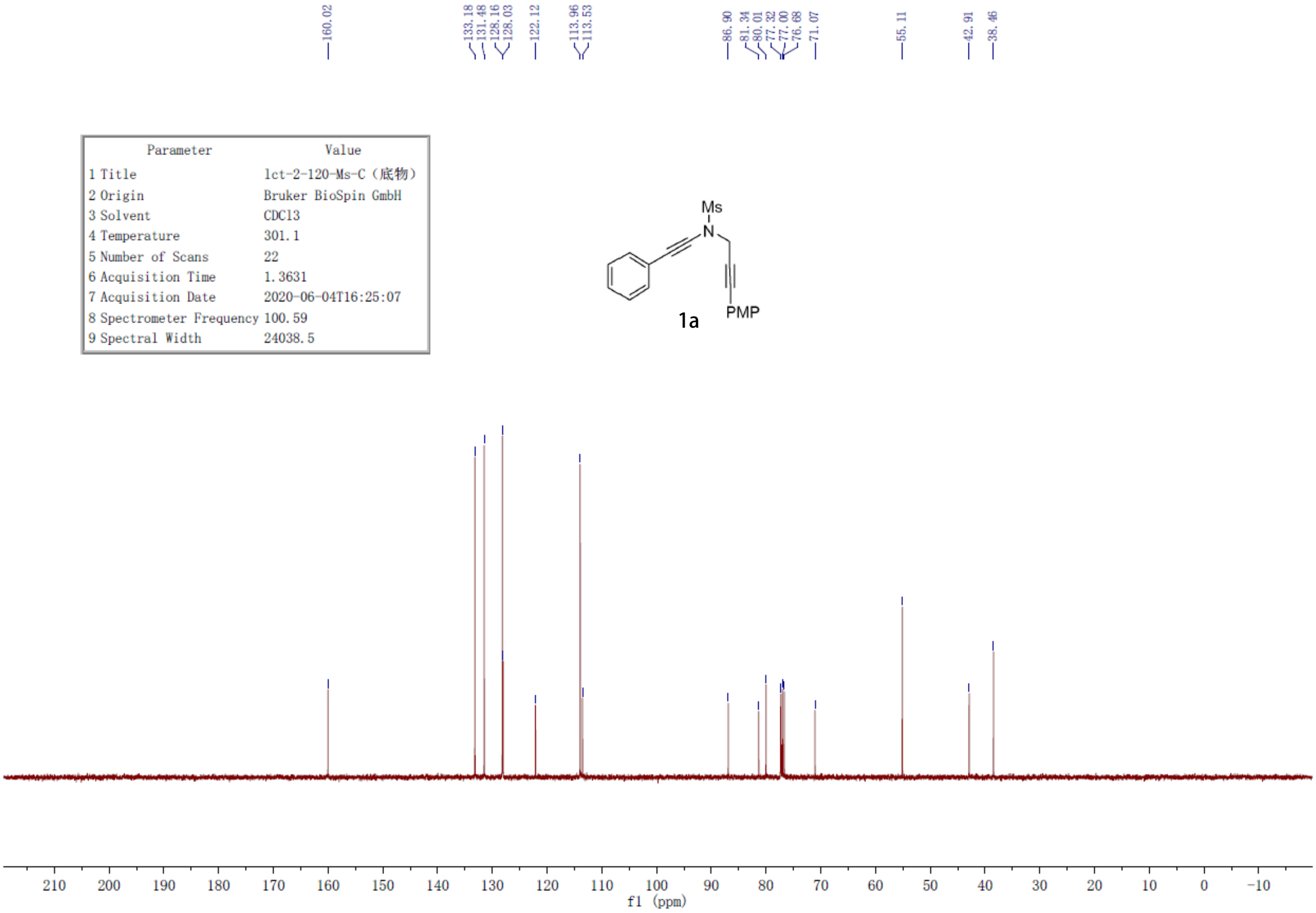

Supplementary Figure 7. ^{1}H and ^{13}C NMR spectra for **1a**

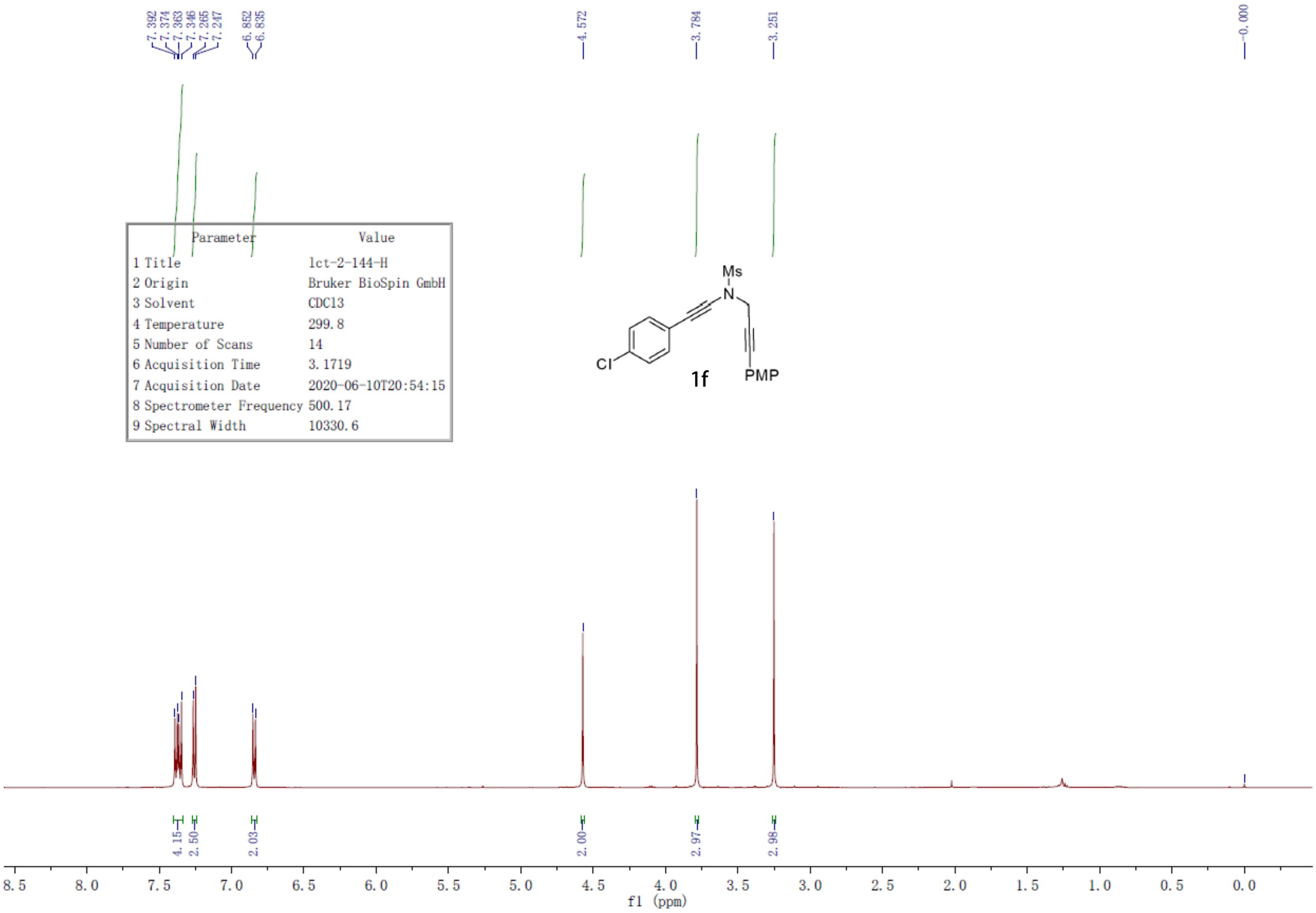

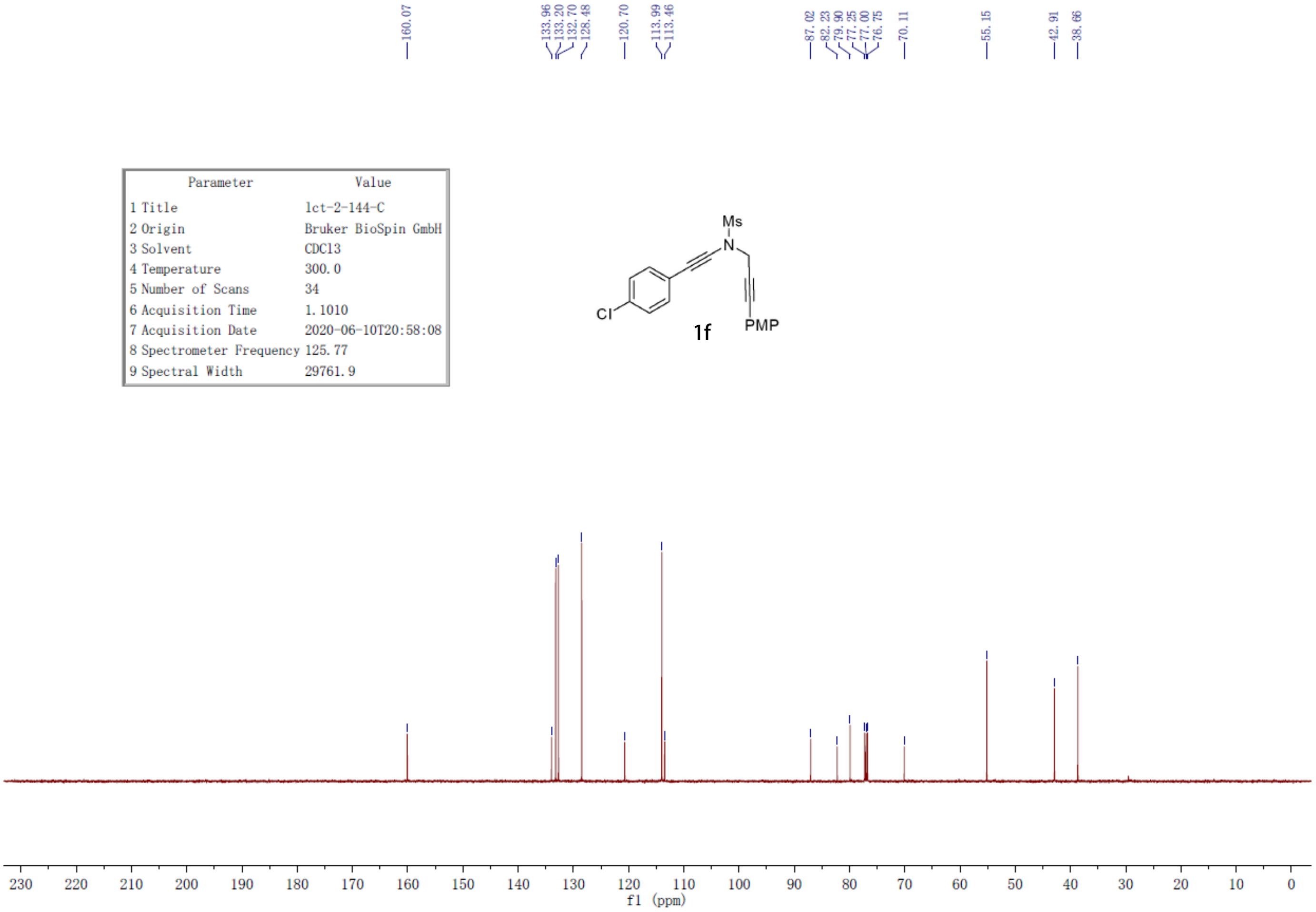

Supplementary Figure 8. ^{1}H and ^{13}C NMR spectra for **1f**

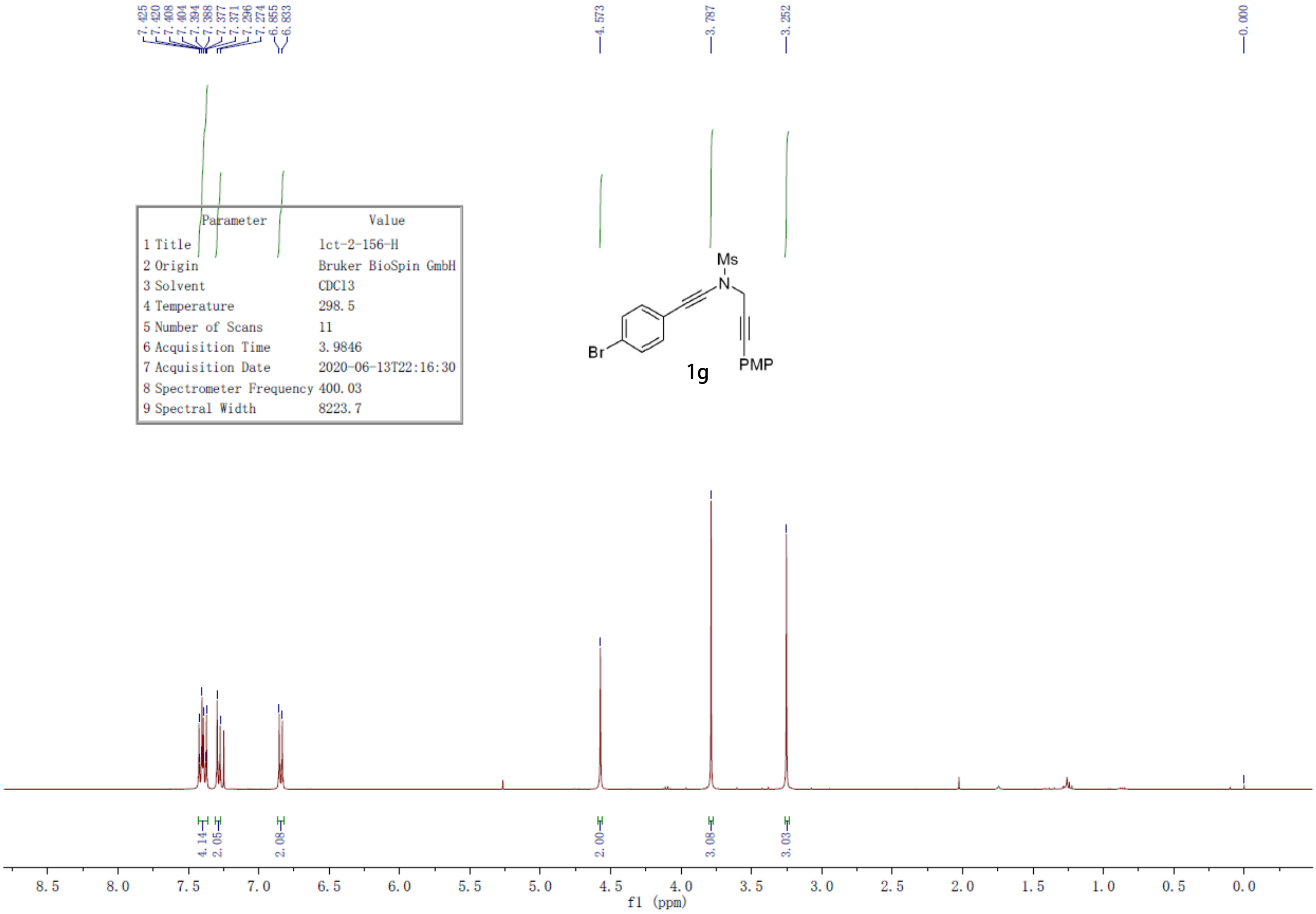

Parameter	Value
1 Title	lct-2-156-H
2 Origin	Bruker BioSpin GmbH
3 Solvent	CDCl3
4 Temperature	298.5
5 Number of Scans	11
6 Acquisition Time	3.9846
7 Acquisition Date	2020-06-13T22:16:30
8 Spectrometer Frequency	400.03
9 Spectral Width	8223.7

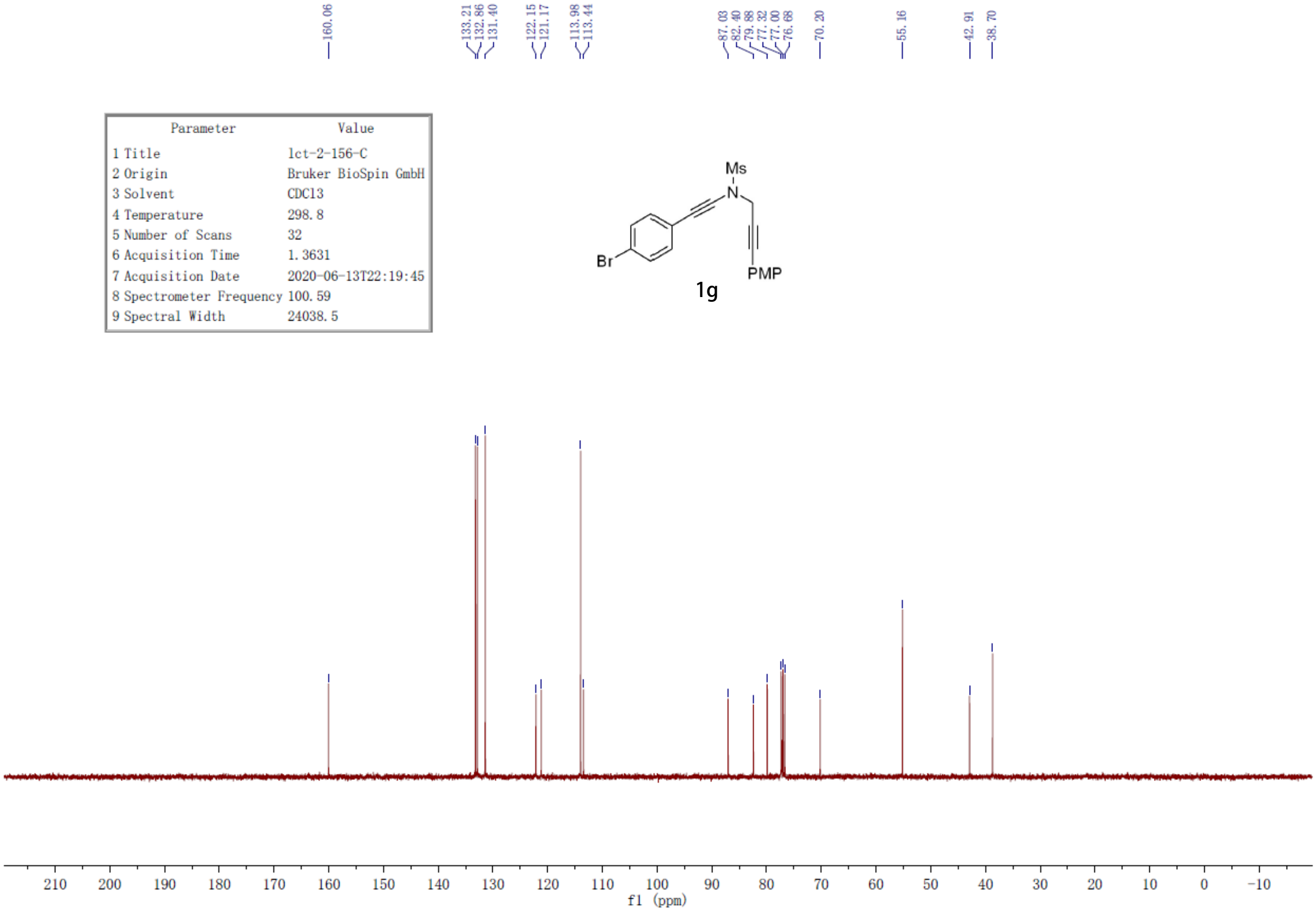

Parameter	Value
1 Title	lct-2-156-C
2 Origin	Bruker BioSpin GmbH
3 Solvent	CDCl3
4 Temperature	298.8
5 Number of Scans	32
6 Acquisition Time	1.3631
7 Acquisition Date	2020-06-13T22:19:45
8 Spectrometer Frequency	100.59
9 Spectral Width	24038.5

Supplementary Figure 9. ^{1}H and ^{13}C NMR spectra for **1g**

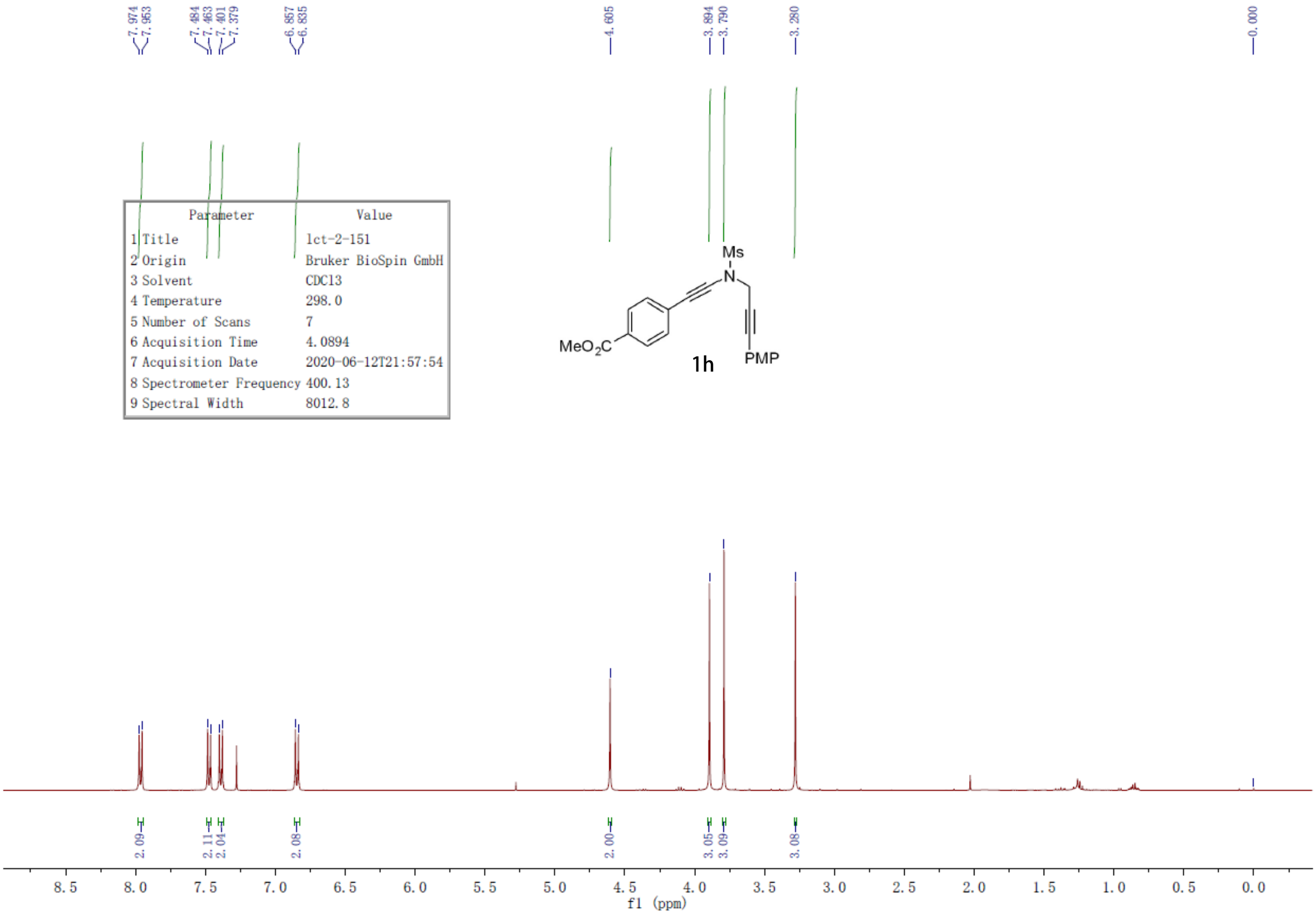

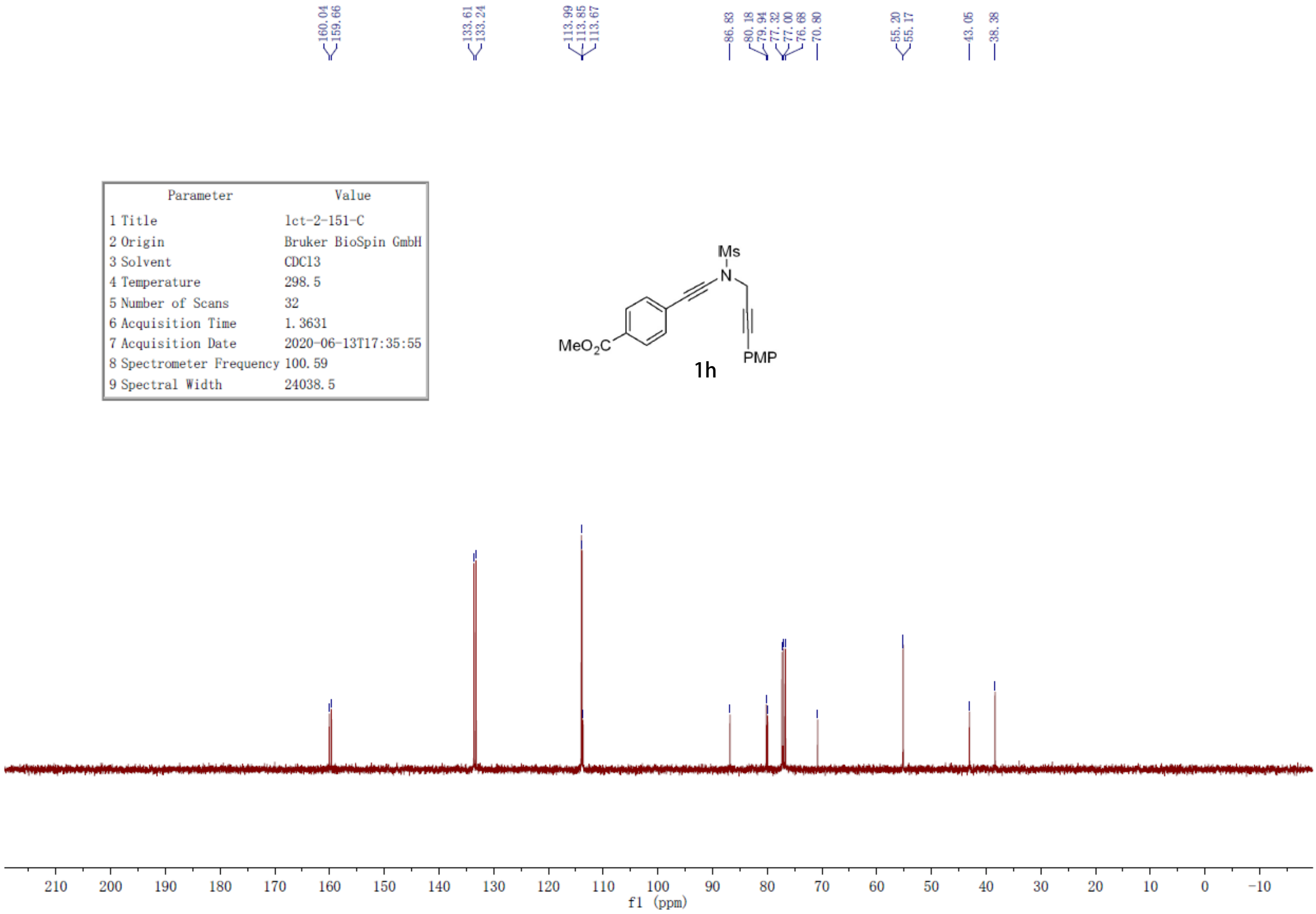

Supplementary Figure 10. ^{1}H and ^{13}C NMR spectra for **1h**

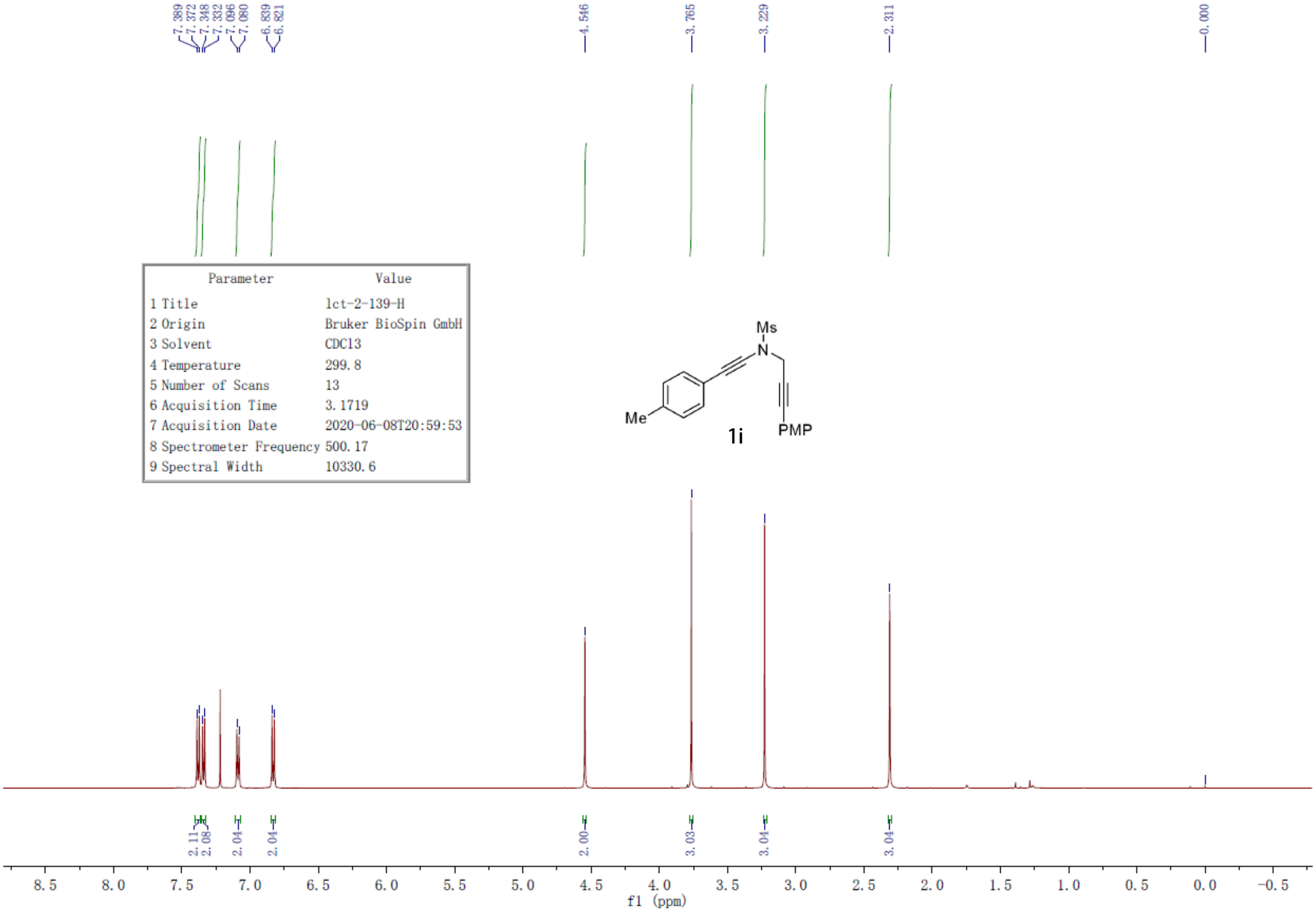

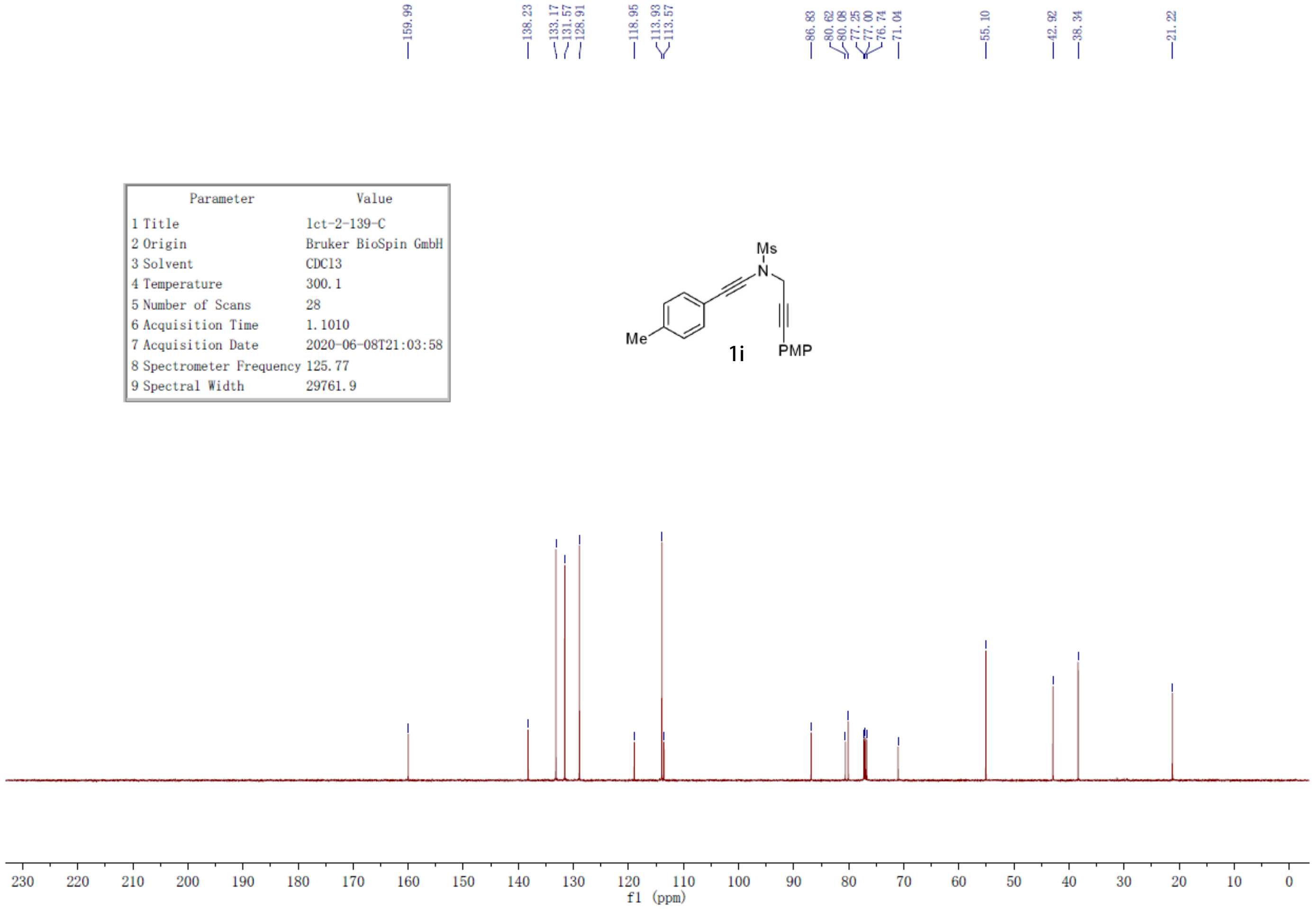

Supplementary Figure 11. ^{1}H and ^{13}C NMR spectra for **1i**

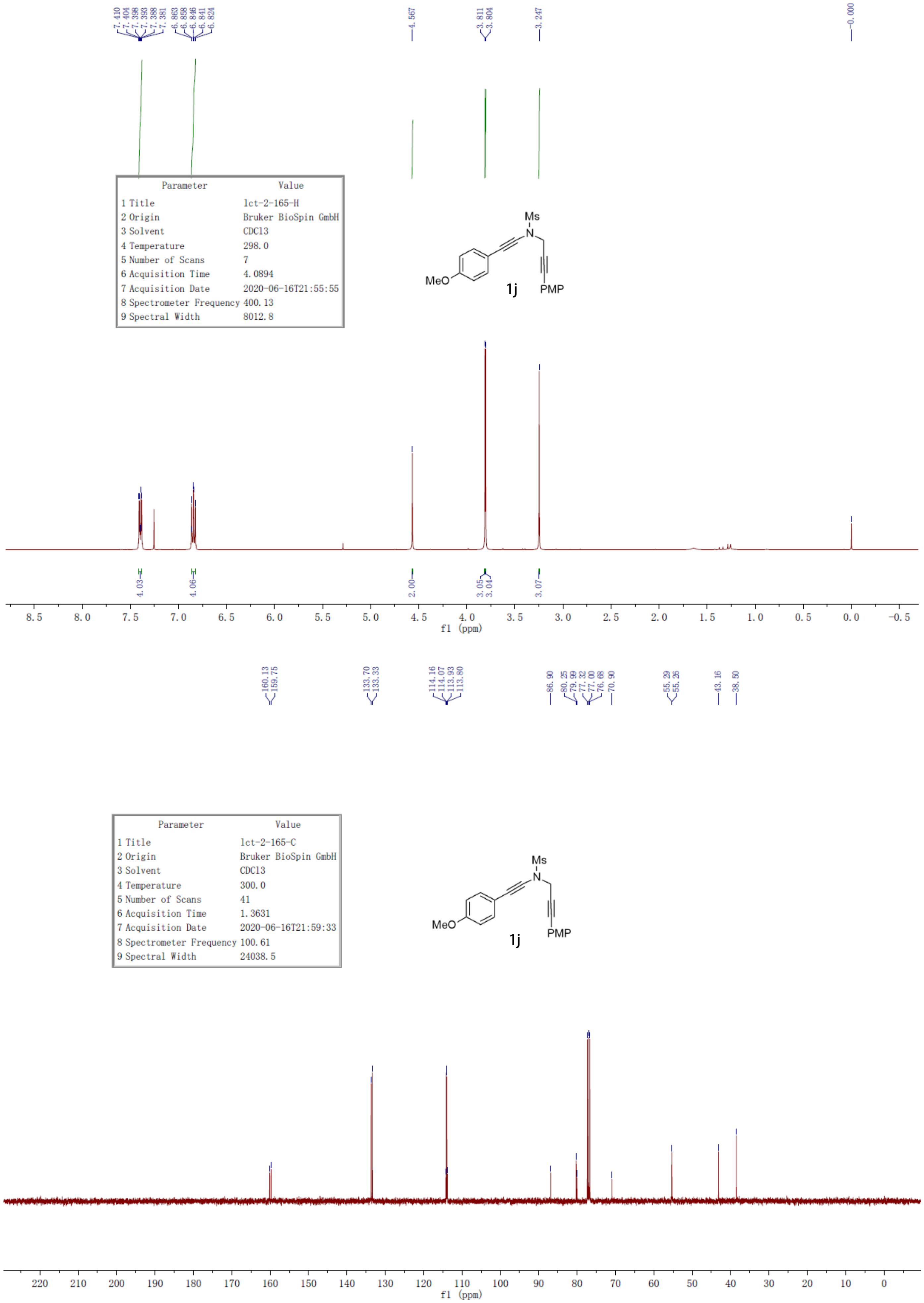

Supplementary Figure 12. ^{1}H and ^{13}C NMR spectra for **1j**

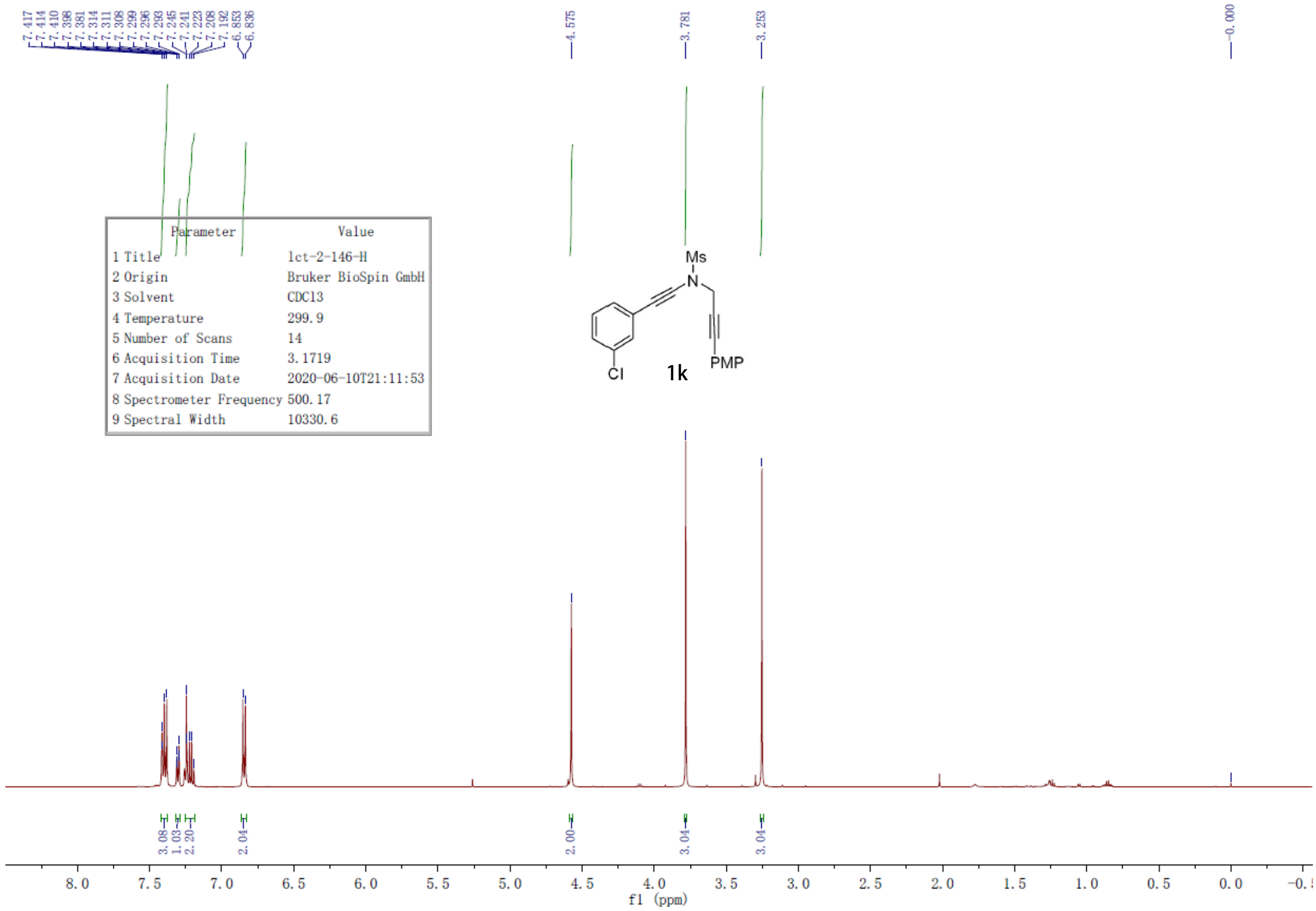

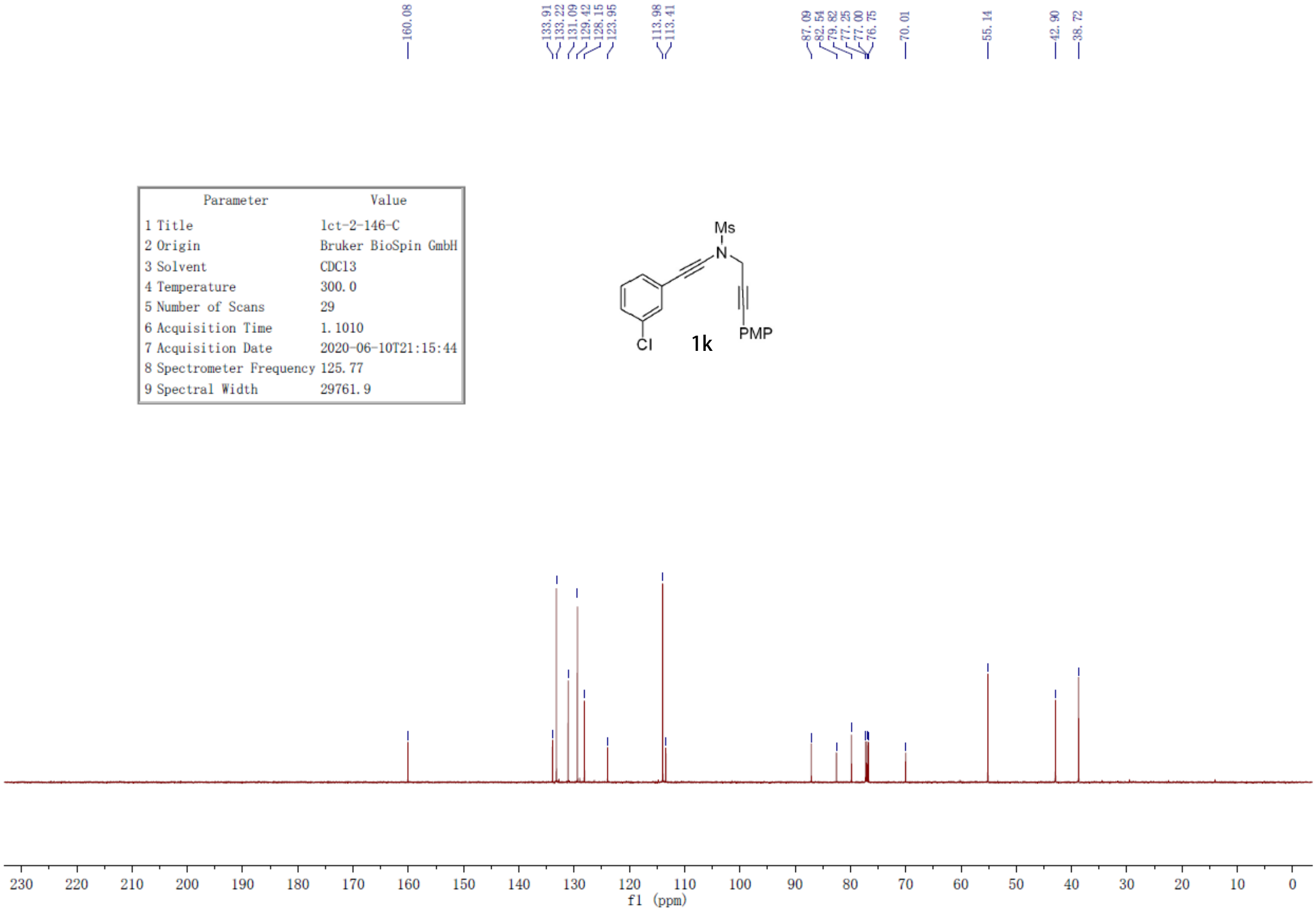

Supplementary Figure 13. ^{1}H and ^{13}C NMR spectra for **1k**

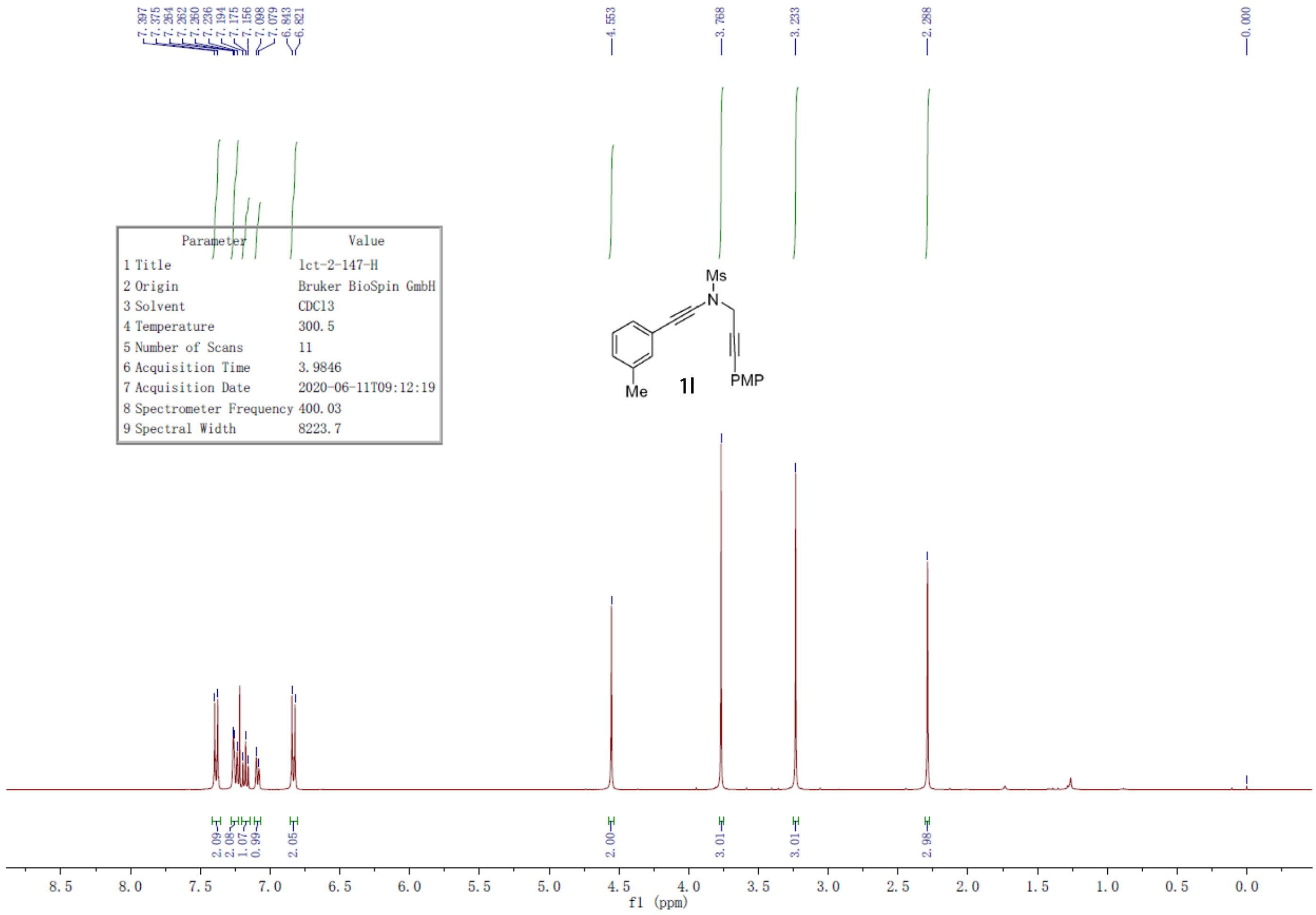

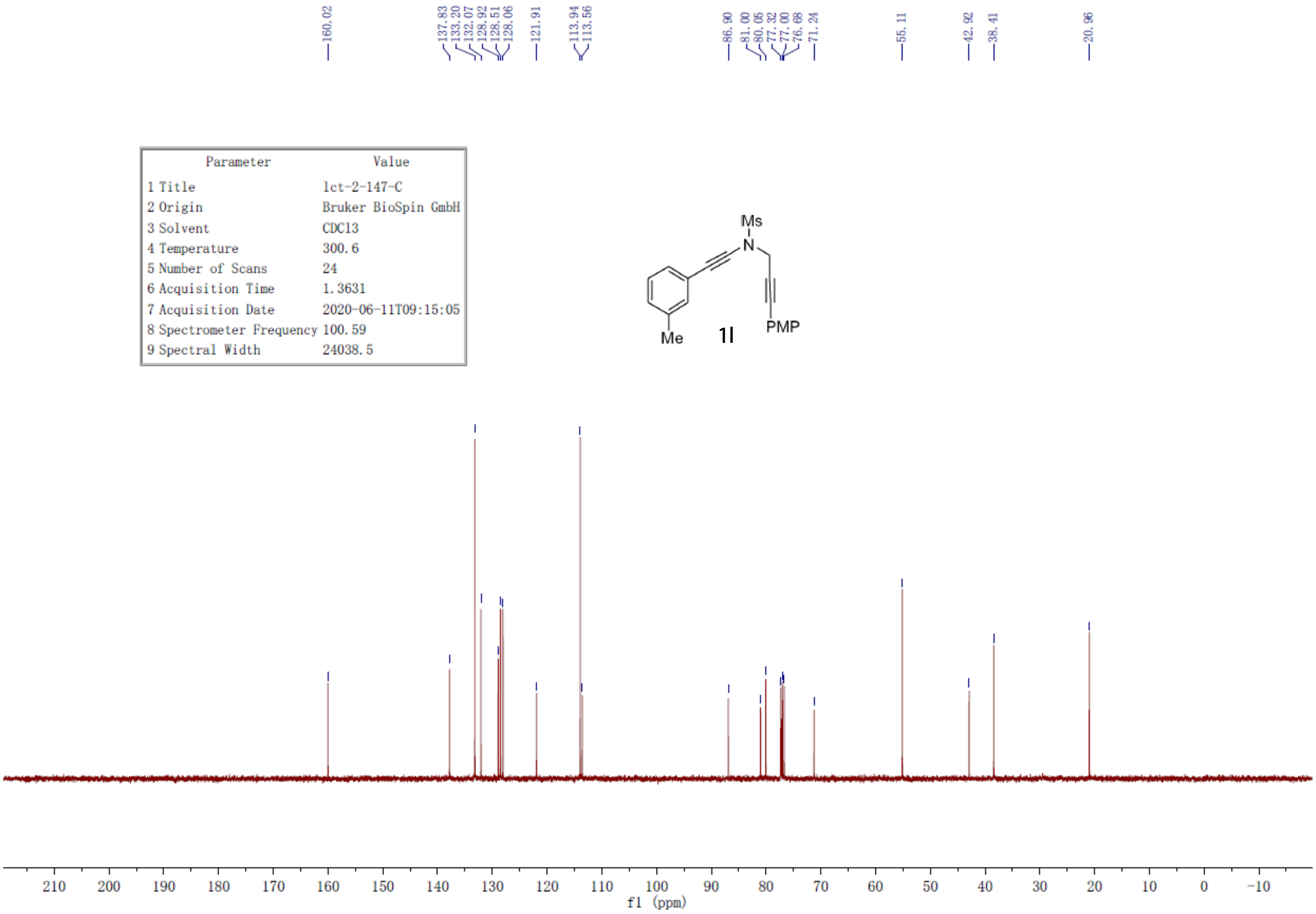

Supplementary Figure 14. ^{1}H and ^{13}C NMR spectra for **1l**

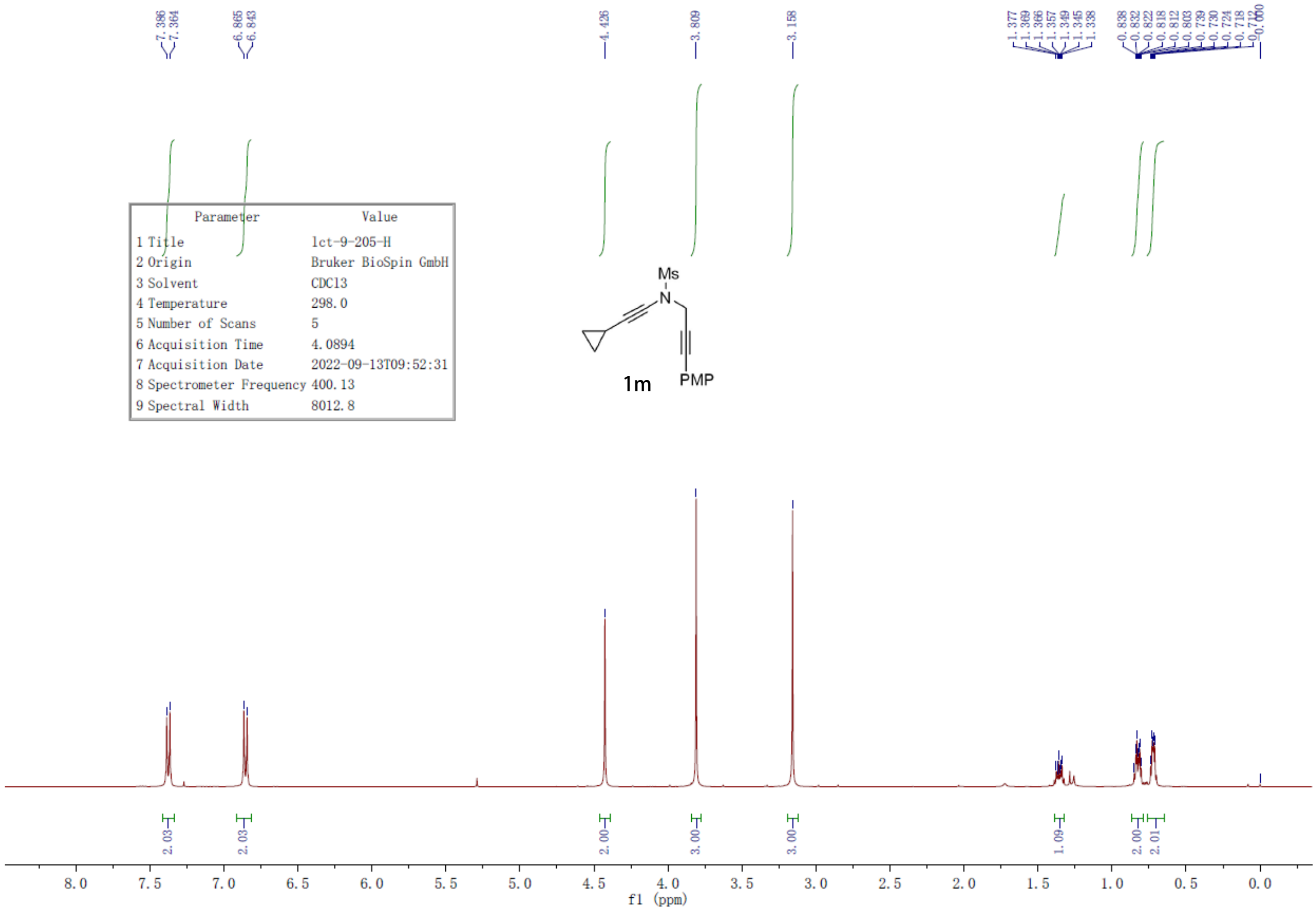

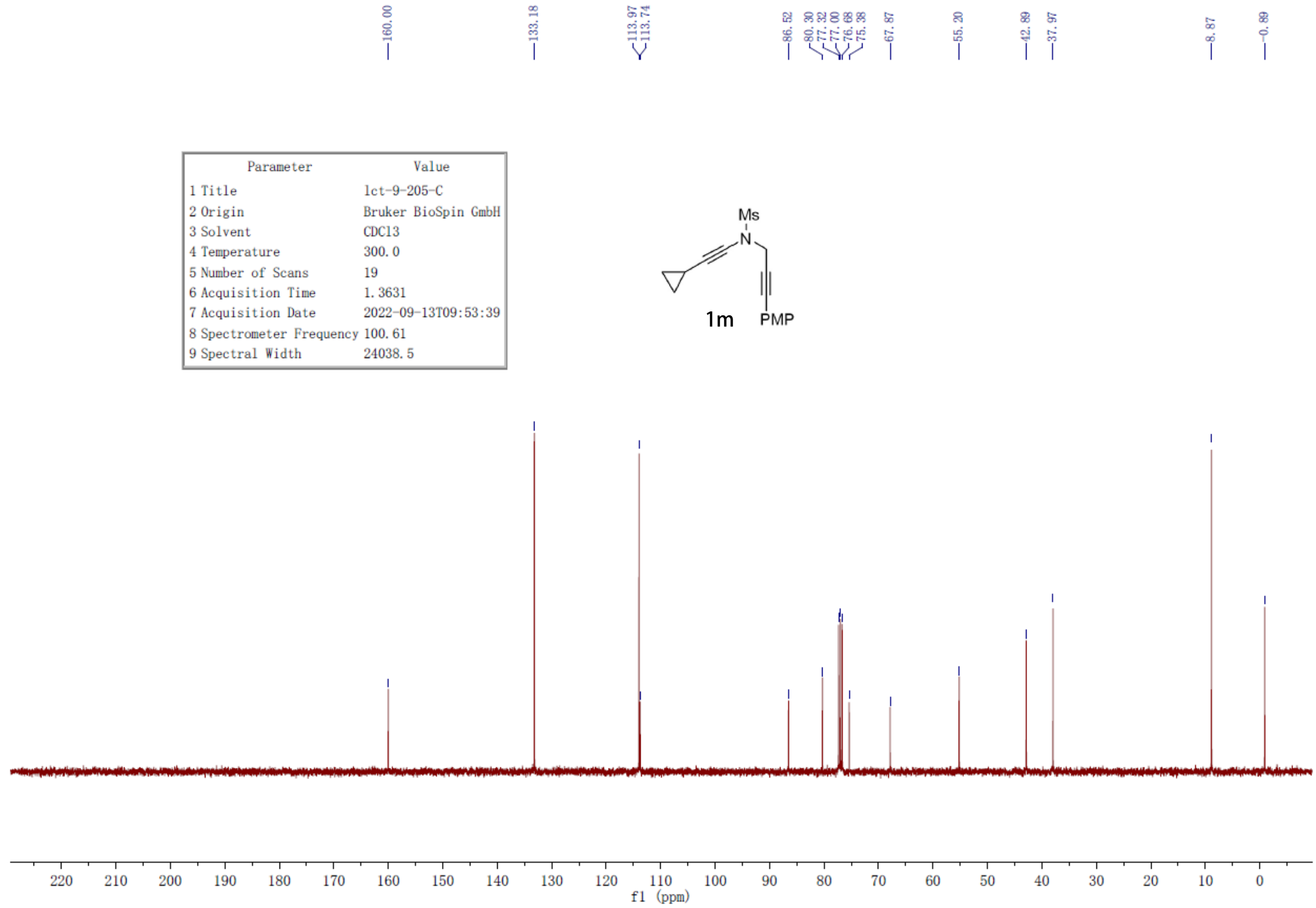

Supplementary Figure 15. ^{1}H and ^{13}C NMR spectra for **1m**

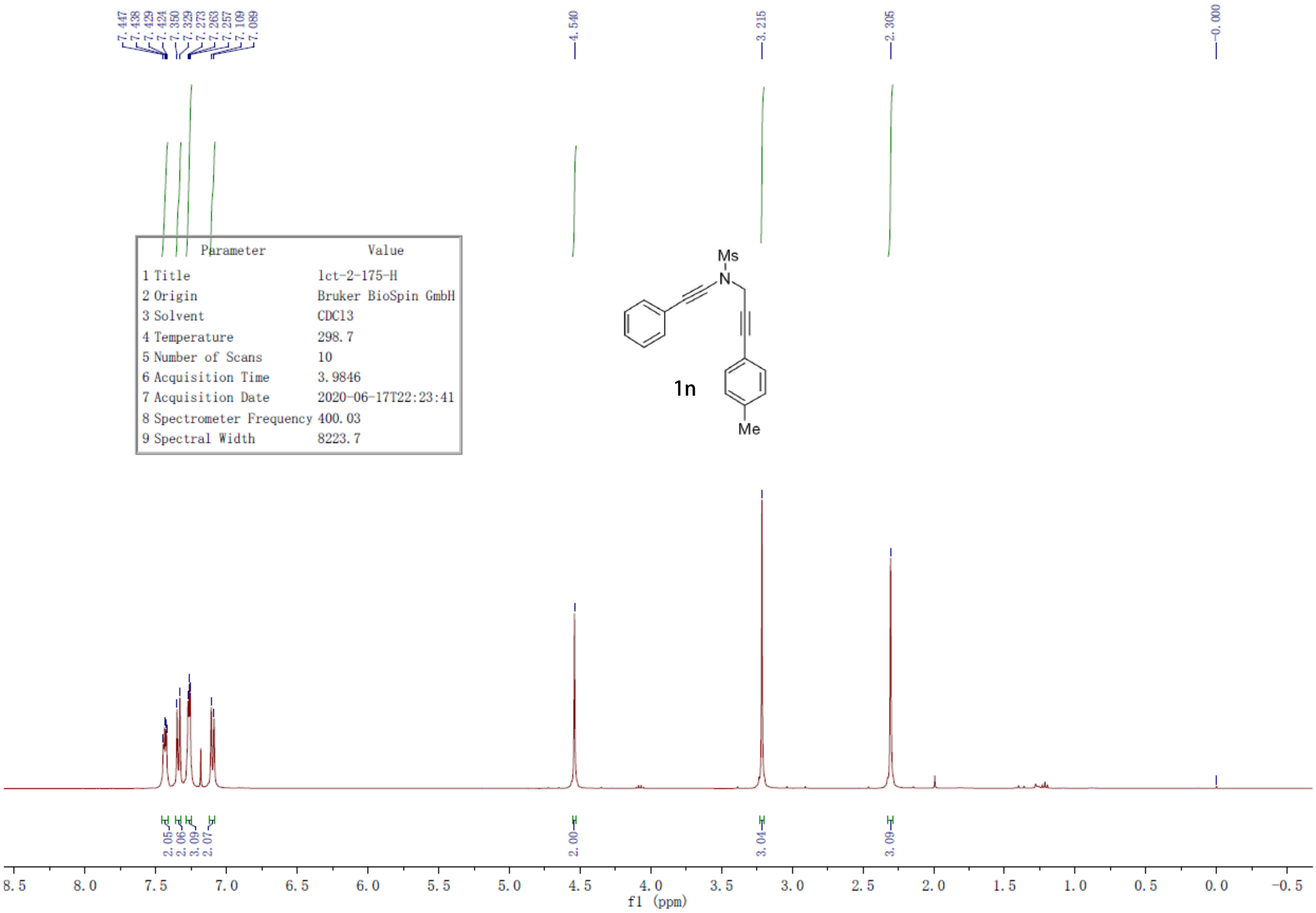

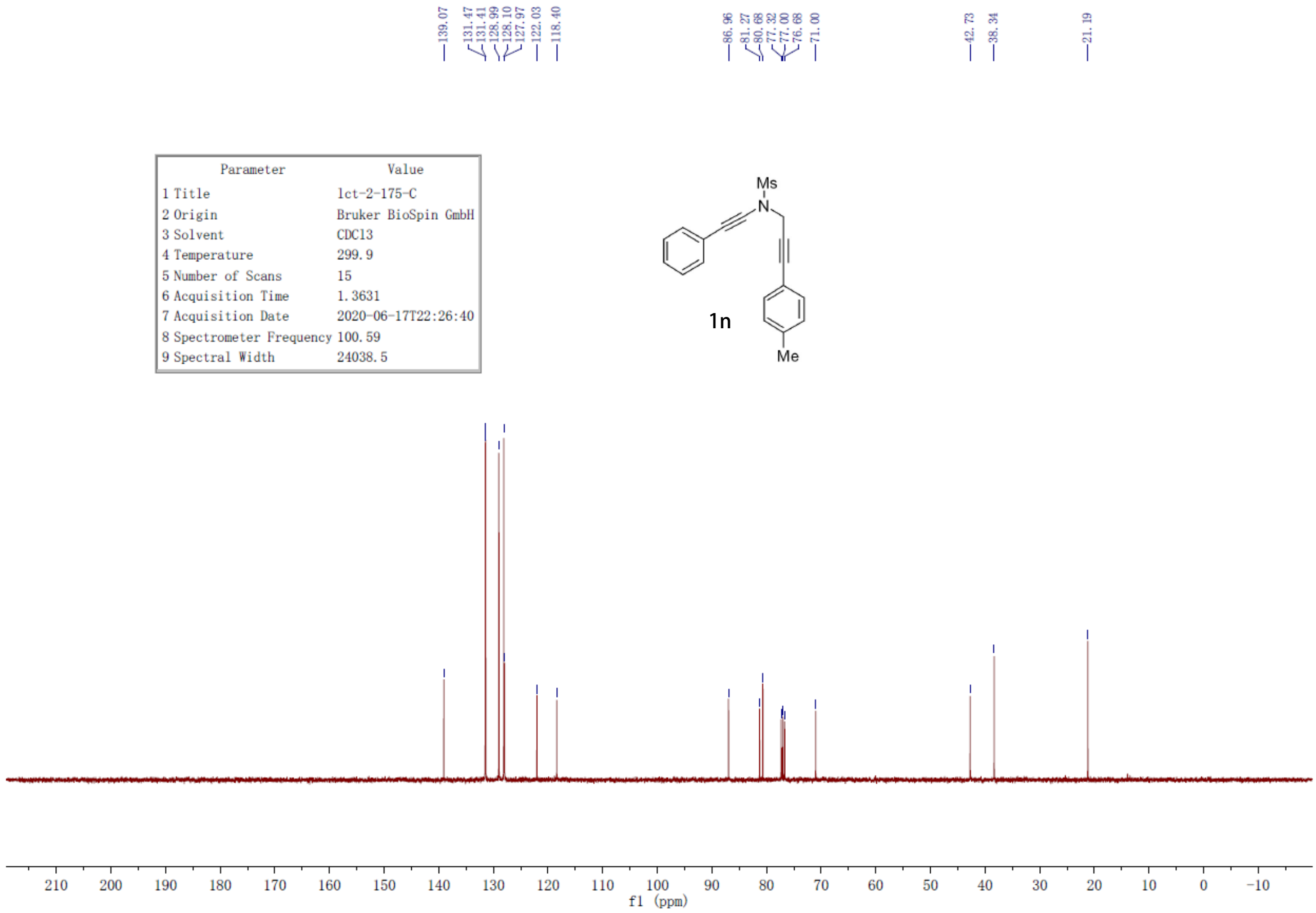

Supplementary Figure 16. ^{1}H and ^{13}C NMR spectra for **1n**

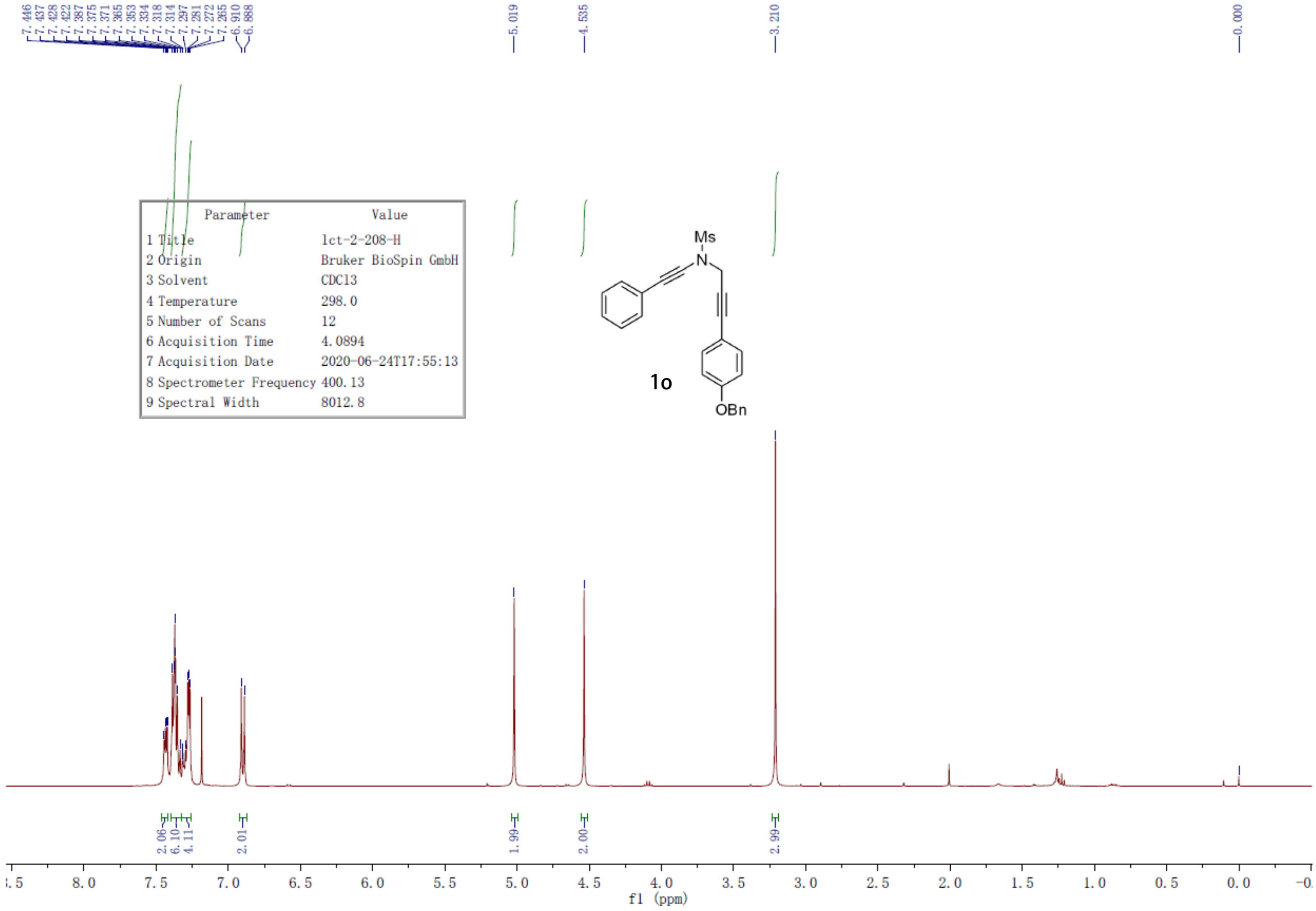

Parameter	Value
1 Title	lct-2-208-H
2 Origin	Bruker BioSpin GmbH
3 Solvent	CDCl3
4 Temperature	298.0
5 Number of Scans	12
6 Acquisition Time	4.0894
7 Acquisition Date	2020-06-24T17:55:13
8 Spectrometer Frequency	400.13
9 Spectral Width	8012.8

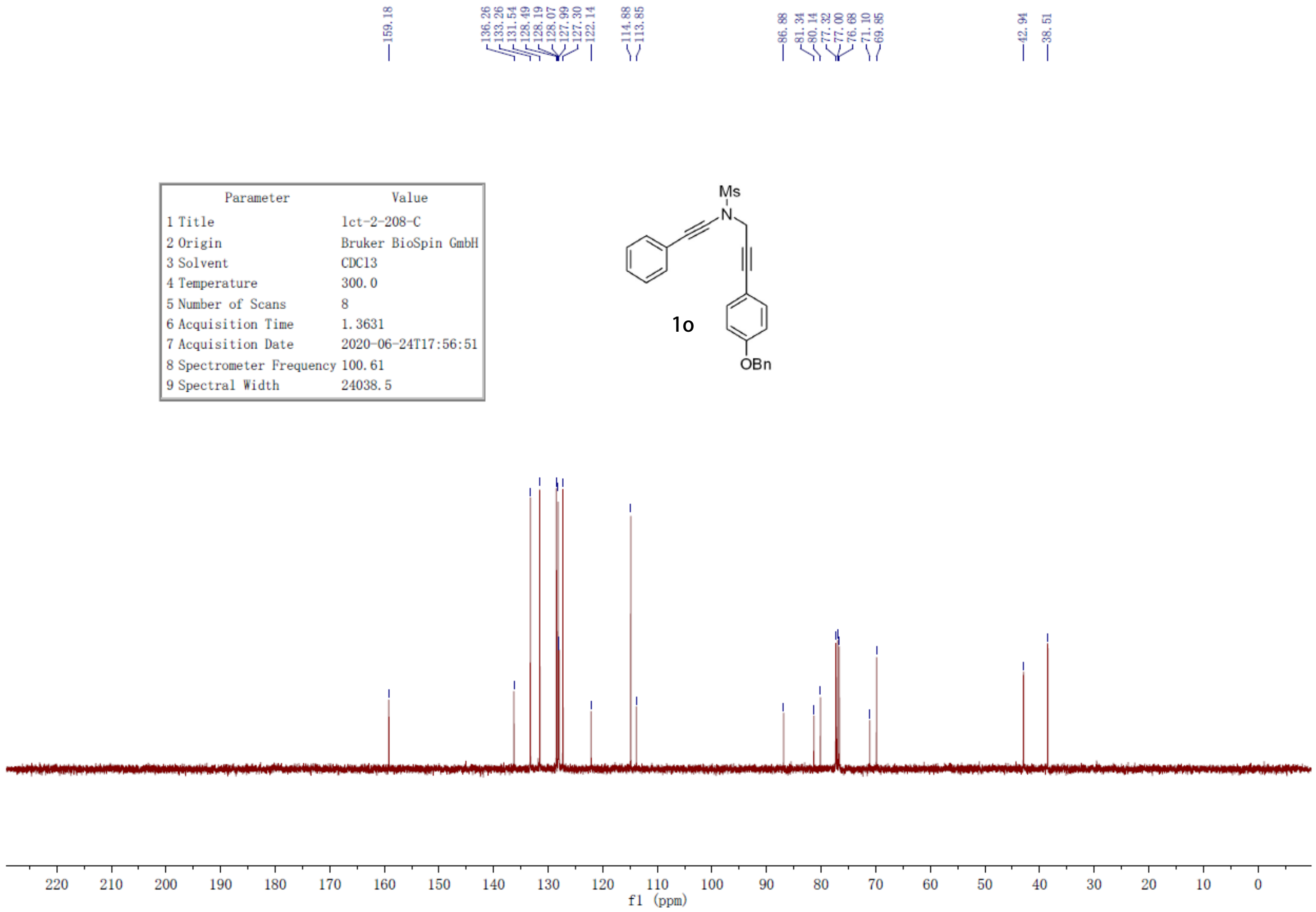

Parameter	Value
1 Title	lct-2-208-C
2 Origin	Bruker BioSpin GmbH
3 Solvent	CDCl3
4 Temperature	300.0
5 Number of Scans	8
6 Acquisition Time	1.3631
7 Acquisition Date	2020-06-24T17:56:51
8 Spectrometer Frequency	100.61
9 Spectral Width	24038.5

Supplementary Figure 17. ^{1}H and ^{13}C NMR spectra for **1o**

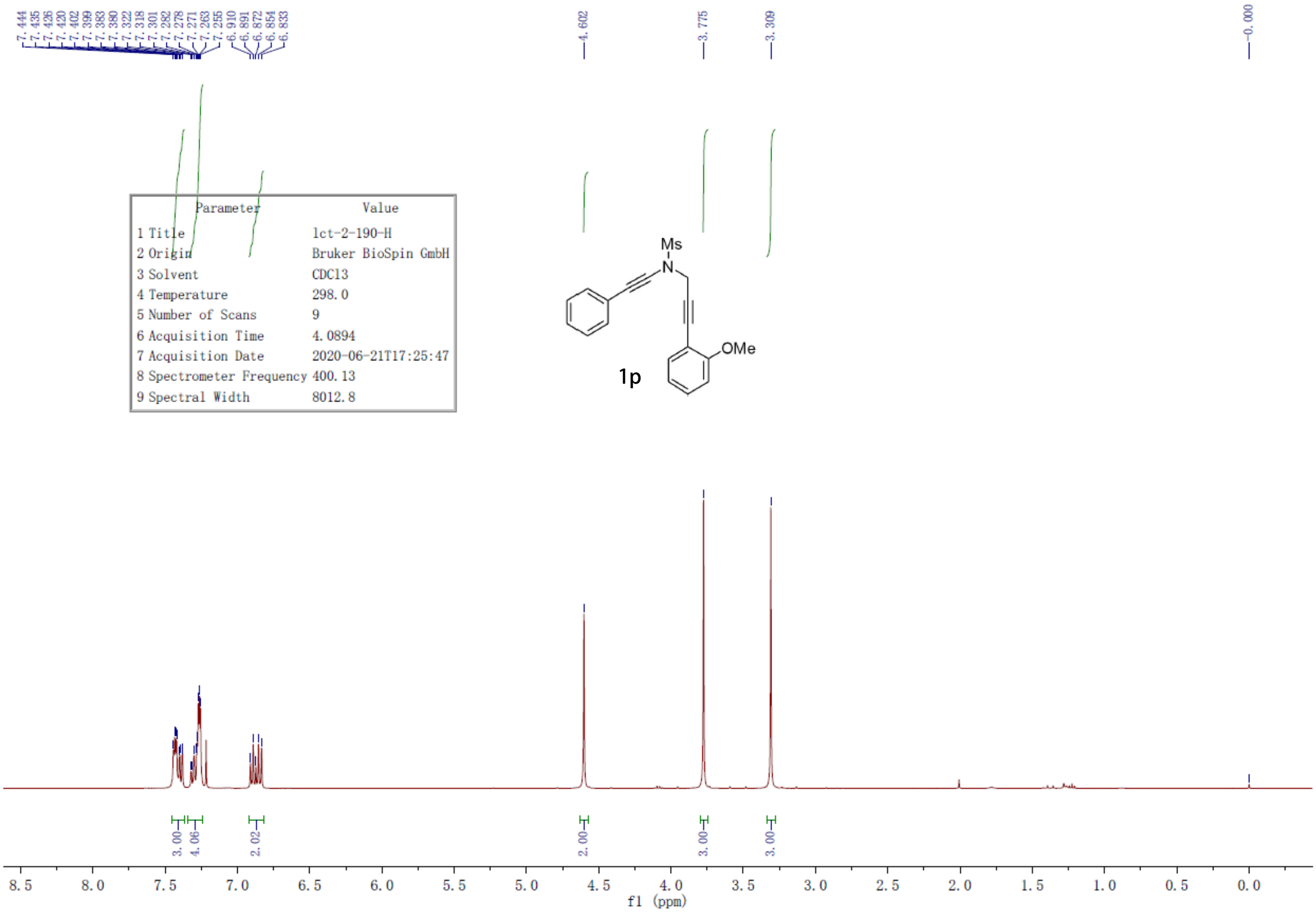

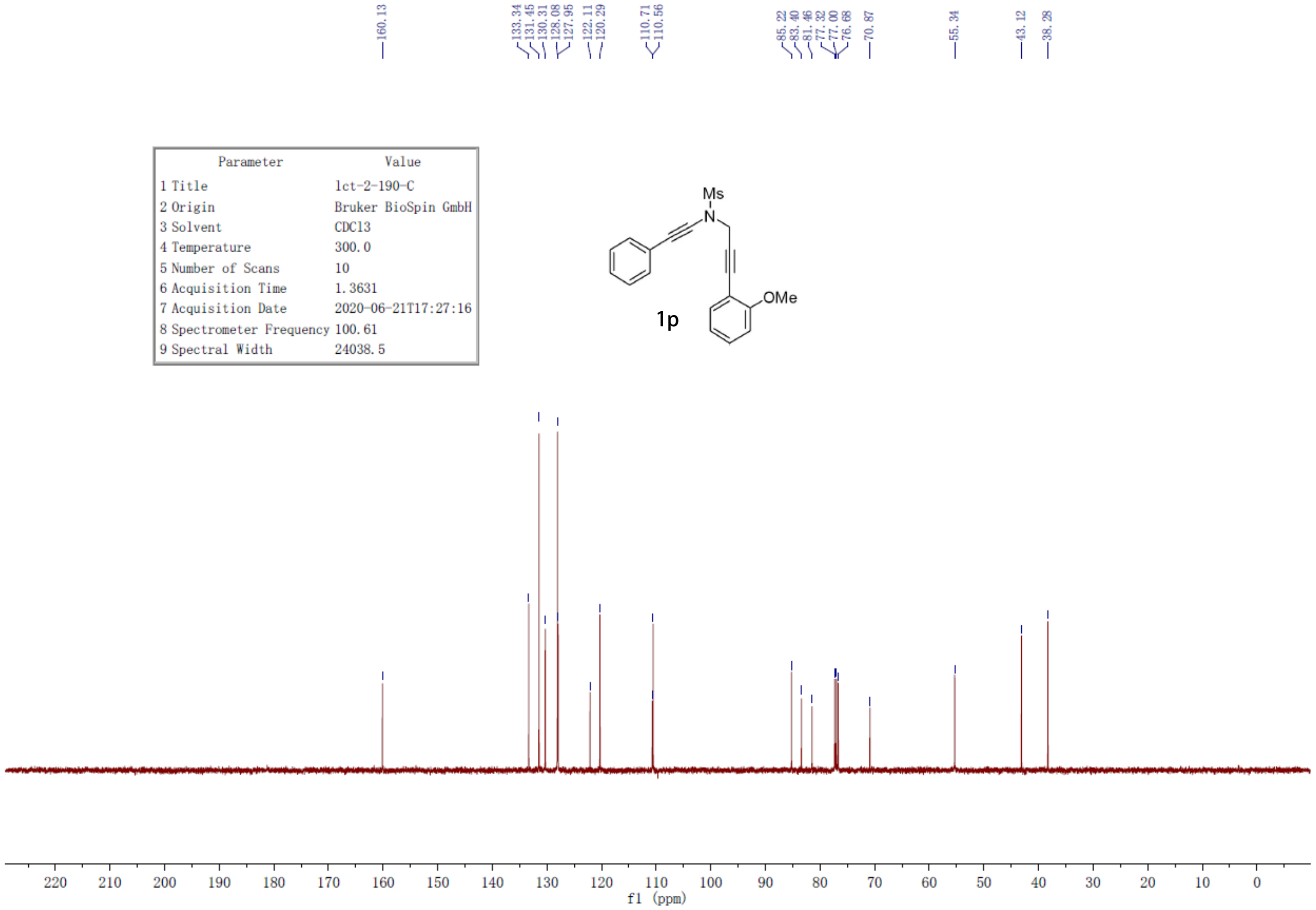

Supplementary Figure 18. ^{1}H and ^{13}C NMR spectra for **1p**

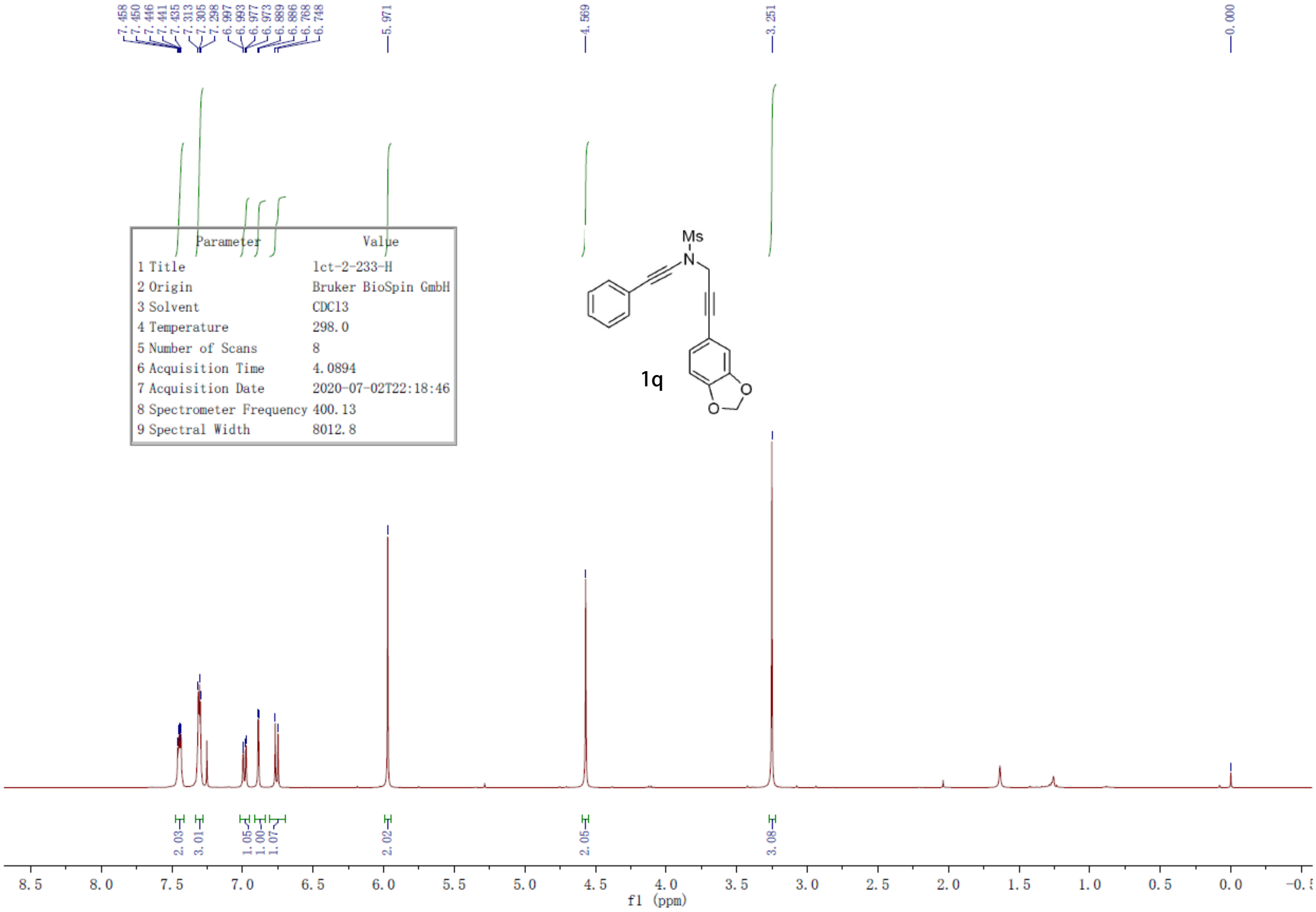

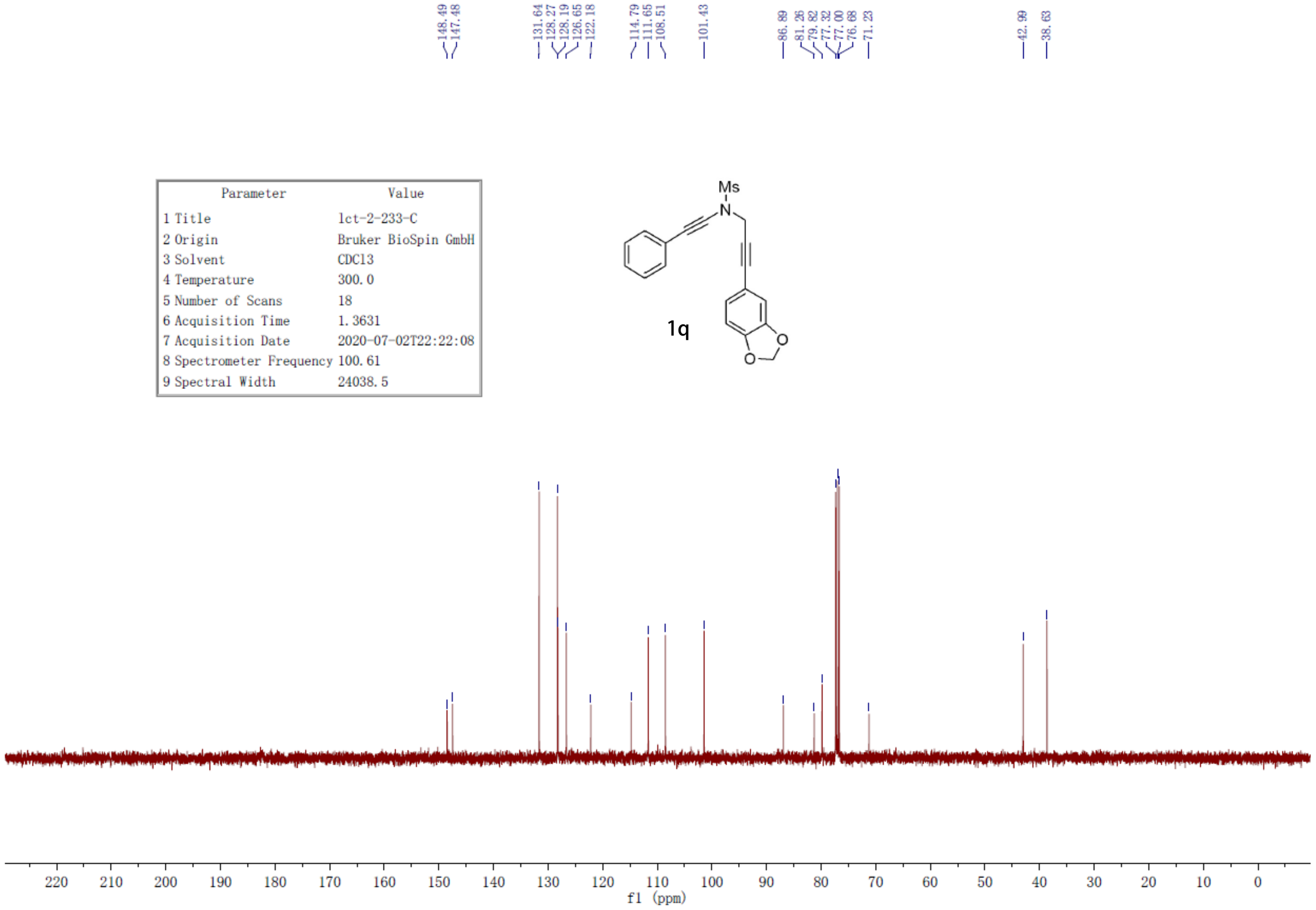

Supplementary Figure 19. ^{1}H and ^{13}C NMR spectra for **1q**

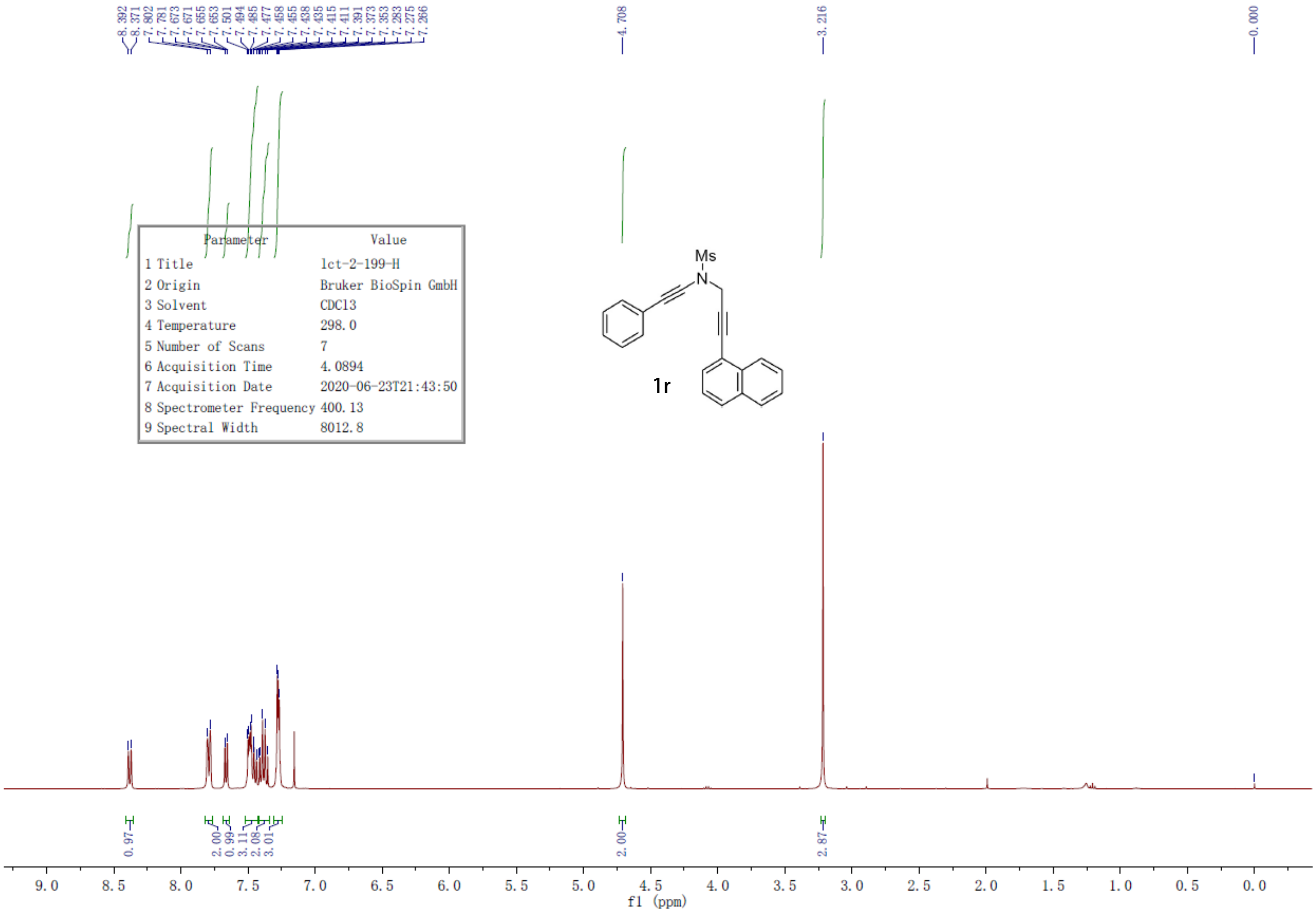

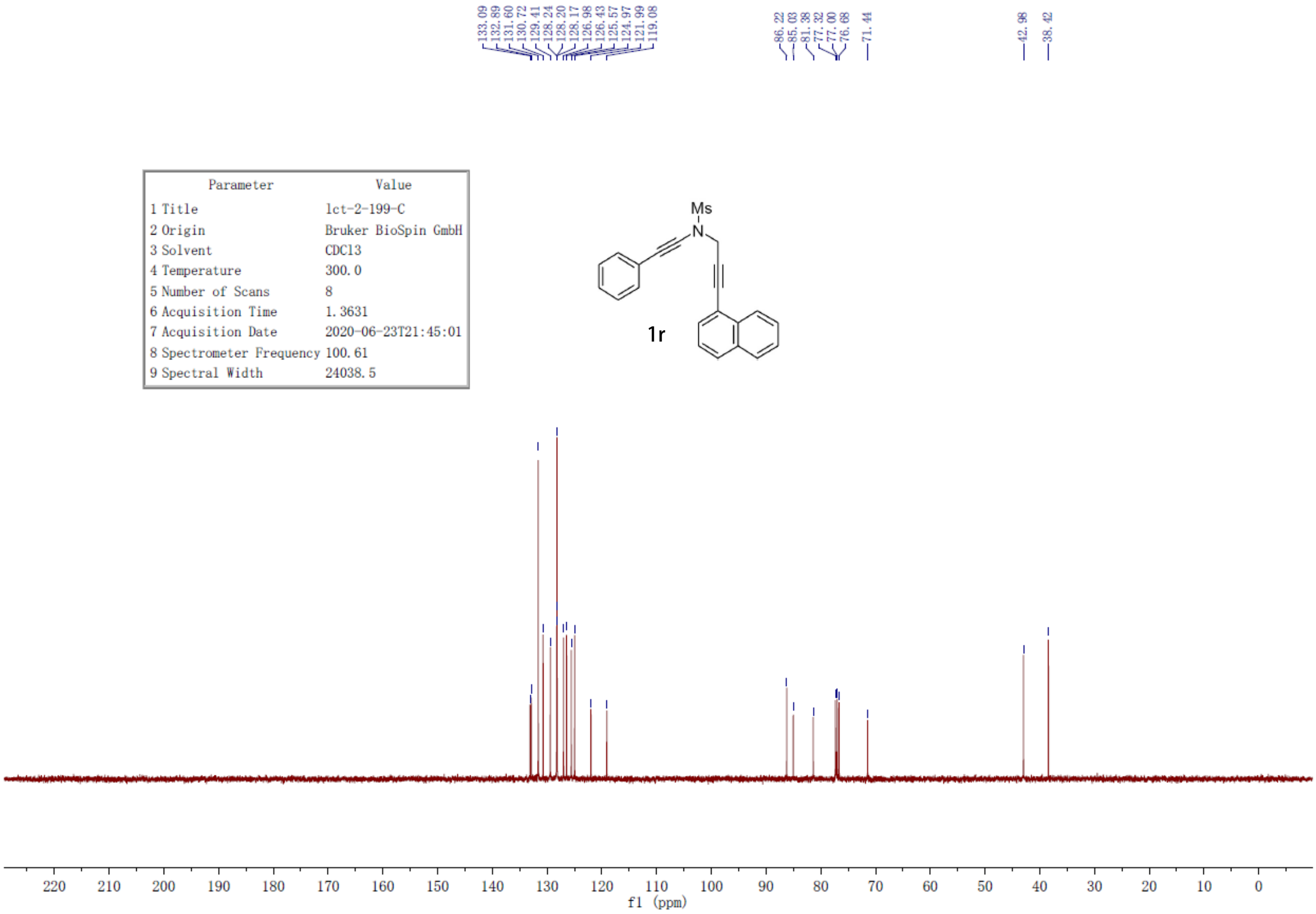

Supplementary Figure 20. ^{1}H and ^{13}C NMR spectra for **1r**

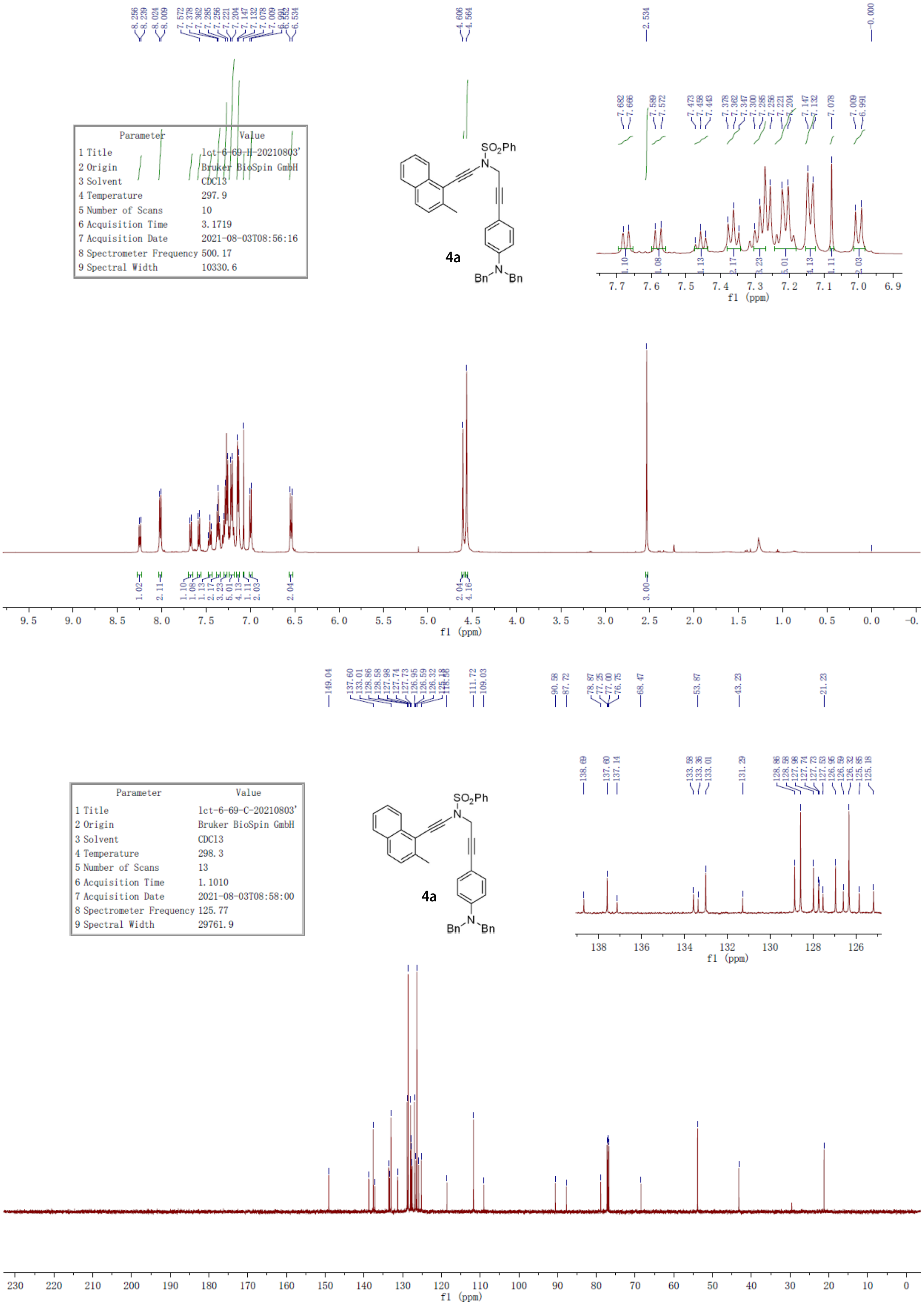

Supplementary Figure 21. ^{1}H and ^{13}C NMR spectra for **4a**

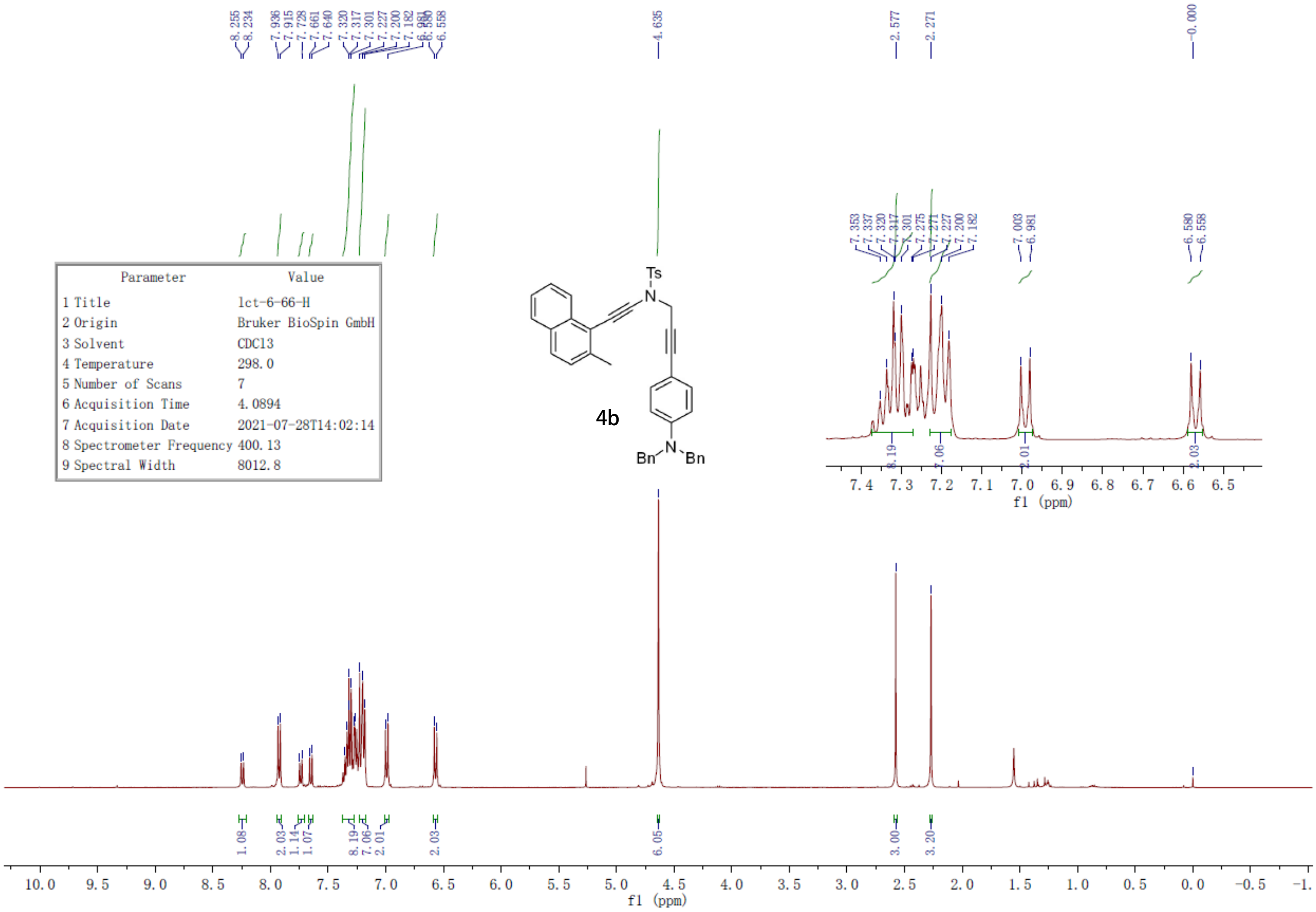

Parameter	Value
1 Title	lct-6-66-H
2 Origin	Bruker BioSpin GmbH
3 Solvent	CDCl3
4 Temperature	298.0
5 Number of Scans	7
6 Acquisition Time	4.0894
7 Acquisition Date	2021-07-28T14:02:14
8 Spectrometer Frequency	400.13
9 Spectral Width	8012.8

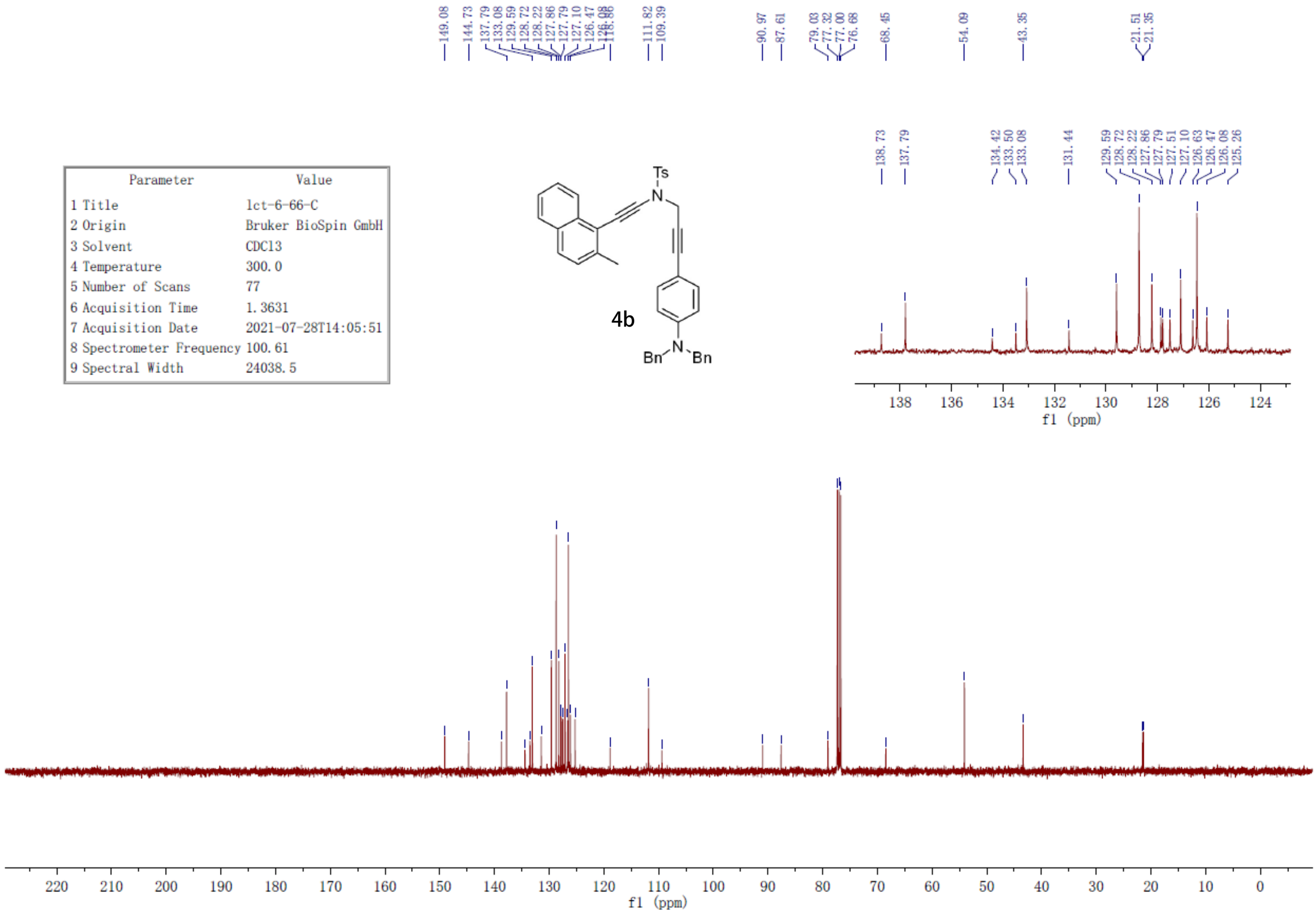

Parameter	Value
1 Title	lct-6-66-C
2 Origin	Bruker BioSpin GmbH
3 Solvent	CDCl3
4 Temperature	300.0
5 Number of Scans	77
6 Acquisition Time	1.3631
7 Acquisition Date	2021-07-28T14:05:51
8 Spectrometer Frequency	100.61
9 Spectral Width	24038.5

Supplementary Figure 22. ^{1}H and ^{13}C NMR spectra for **4b**

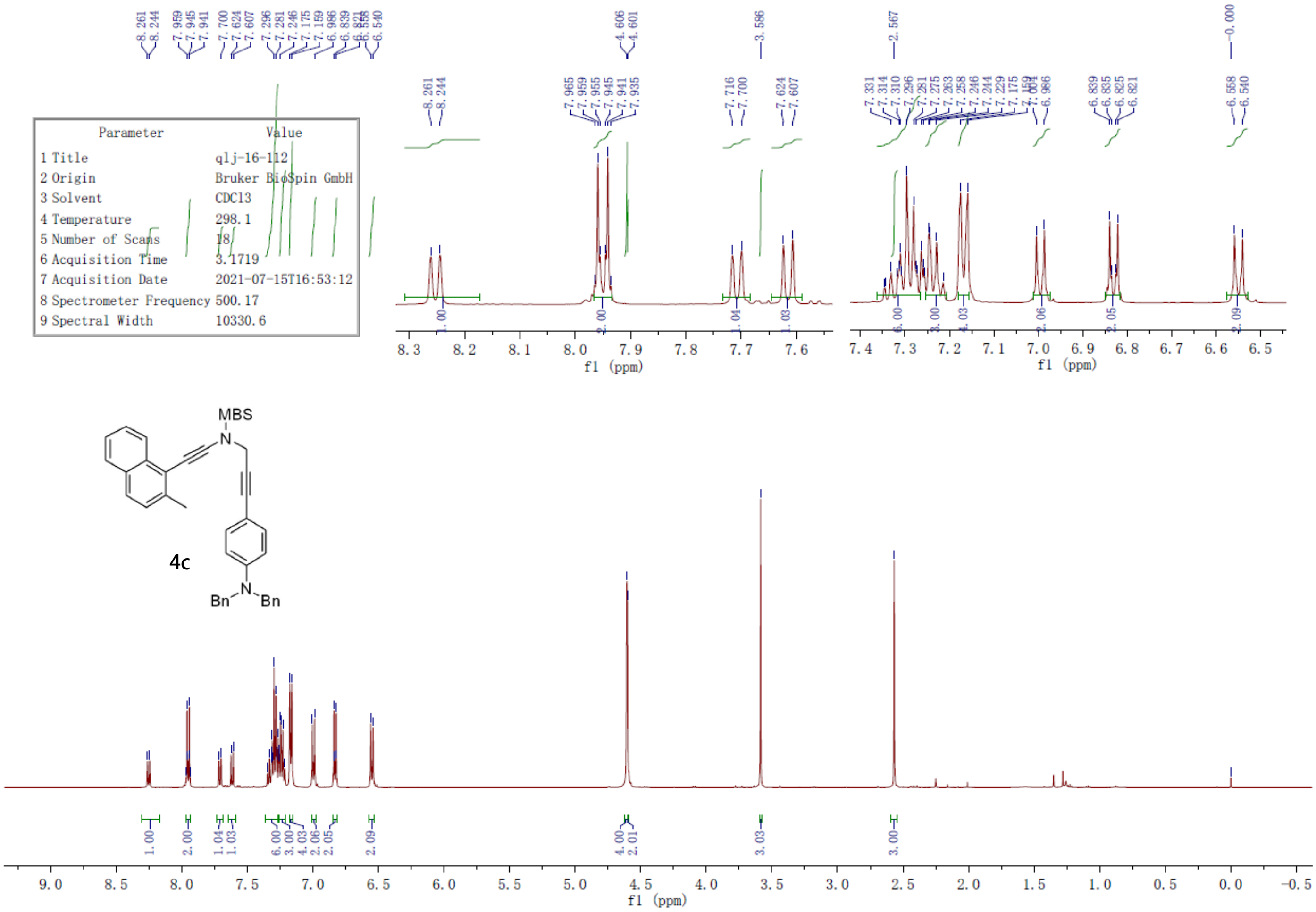

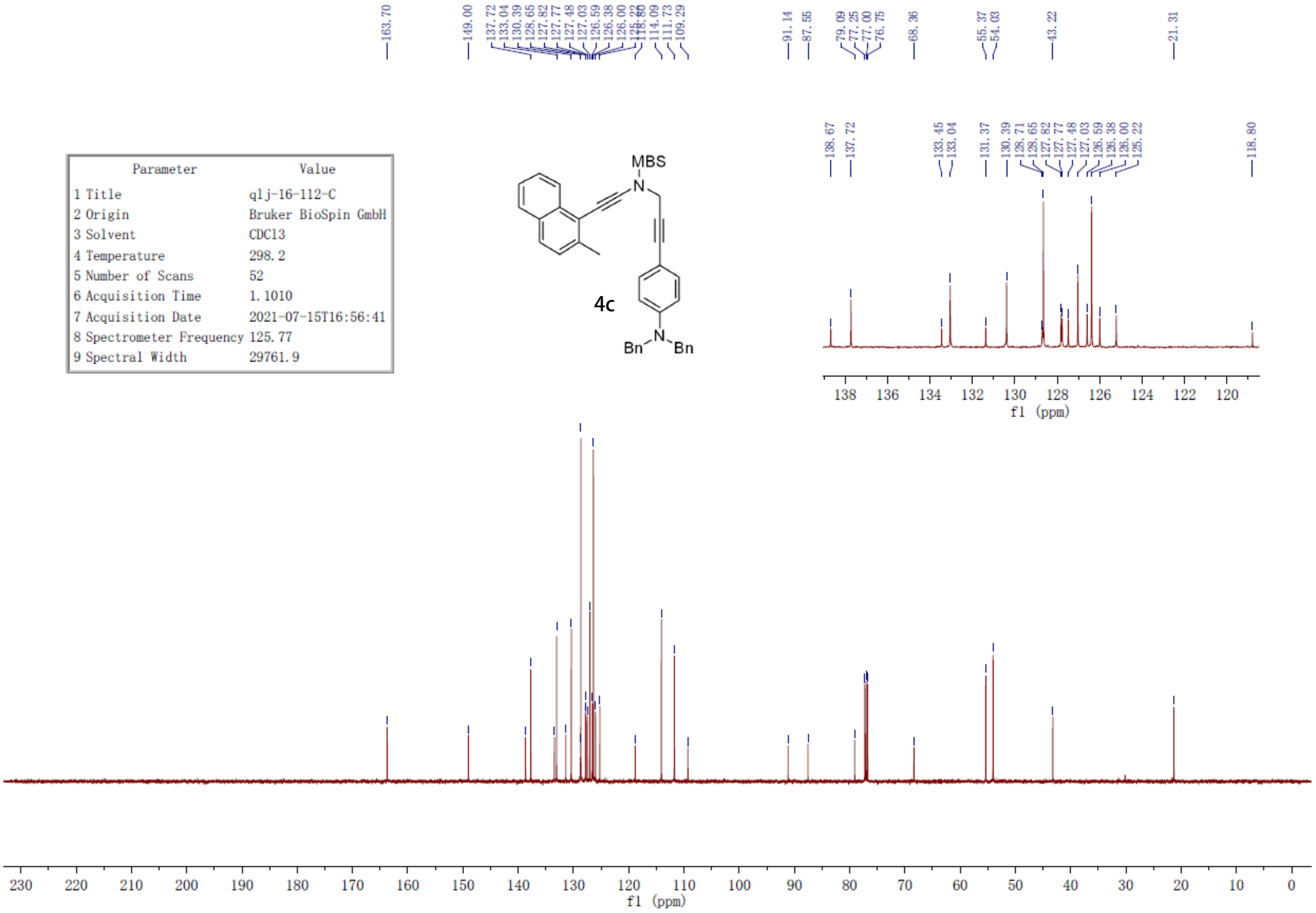

Supplementary Figure 23. ^{1}H and ^{13}C NMR spectra for **4c**

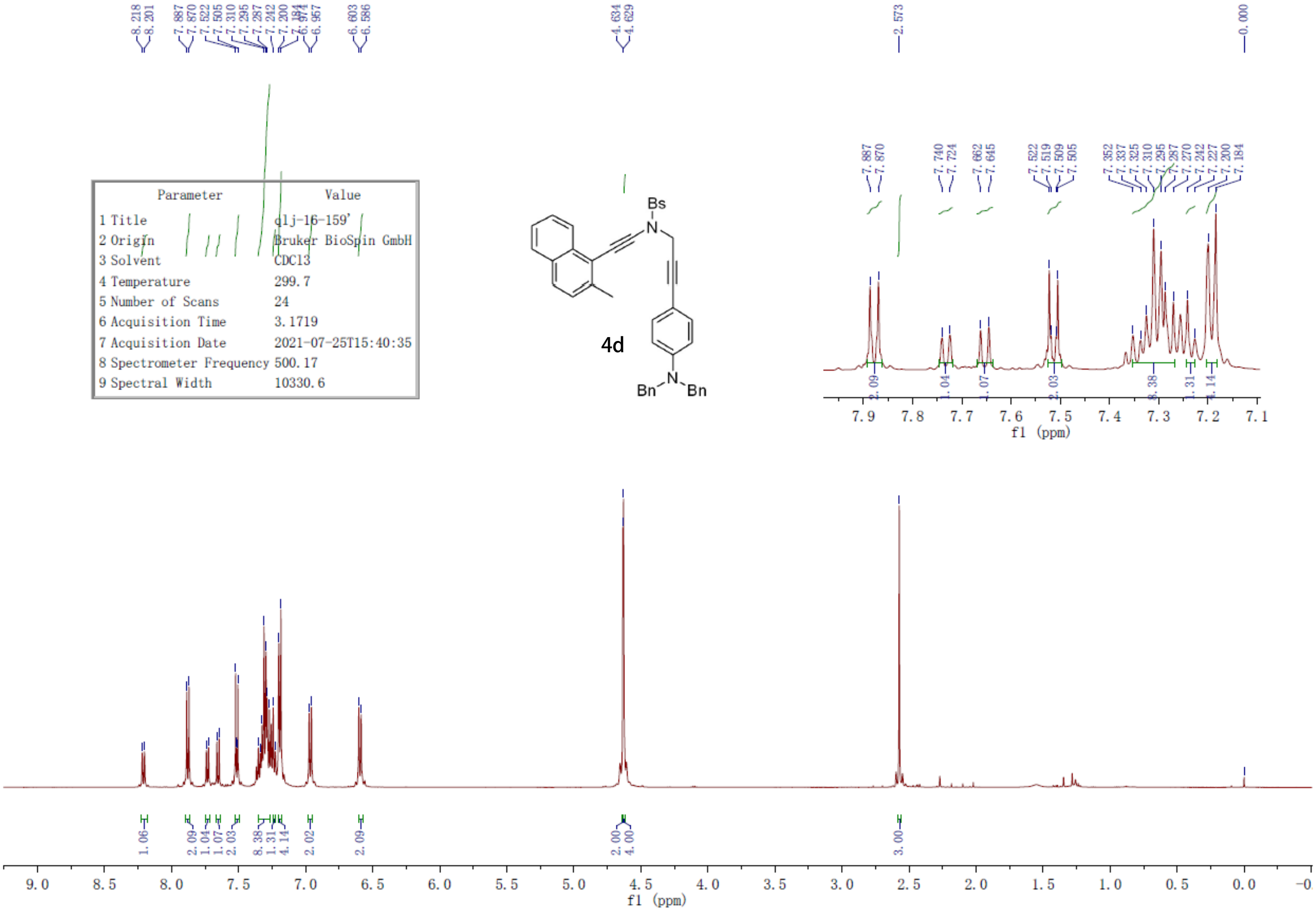

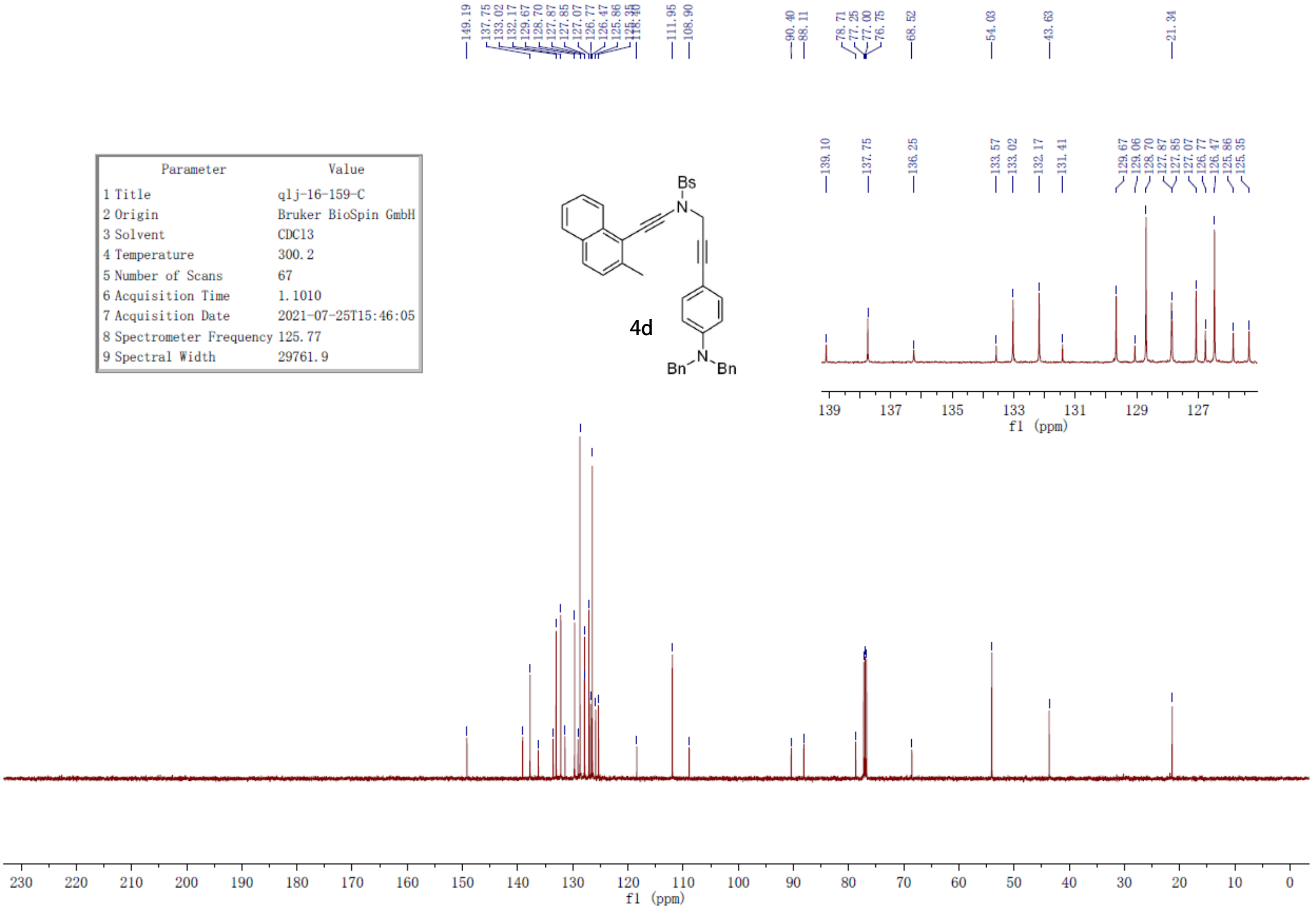

Supplementary Figure 24. ^{1}H and ^{13}C NMR spectra for **4d**

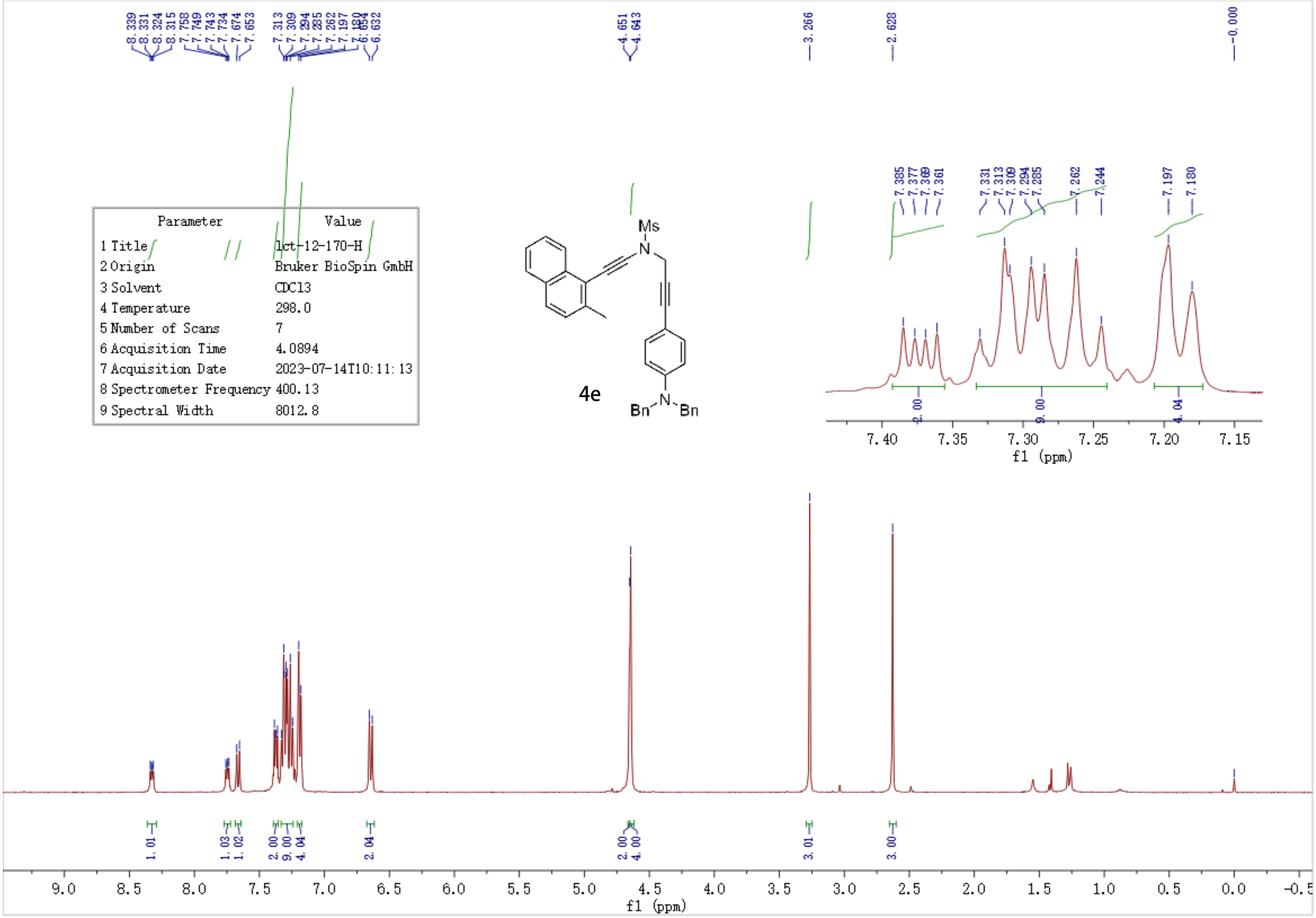

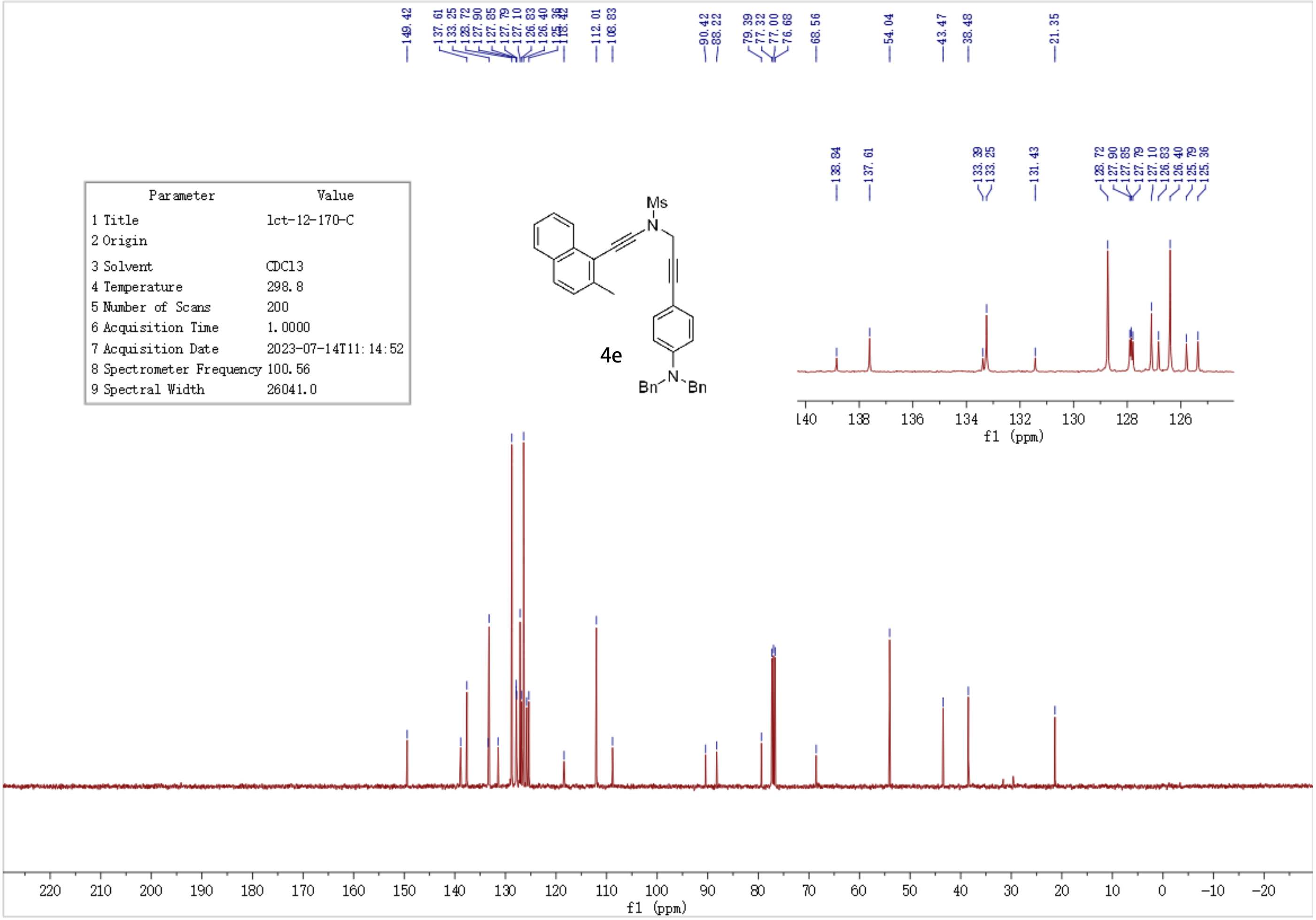

Supplementary Figure 25. ^{1}H and ^{13}C NMR spectra for **4e**

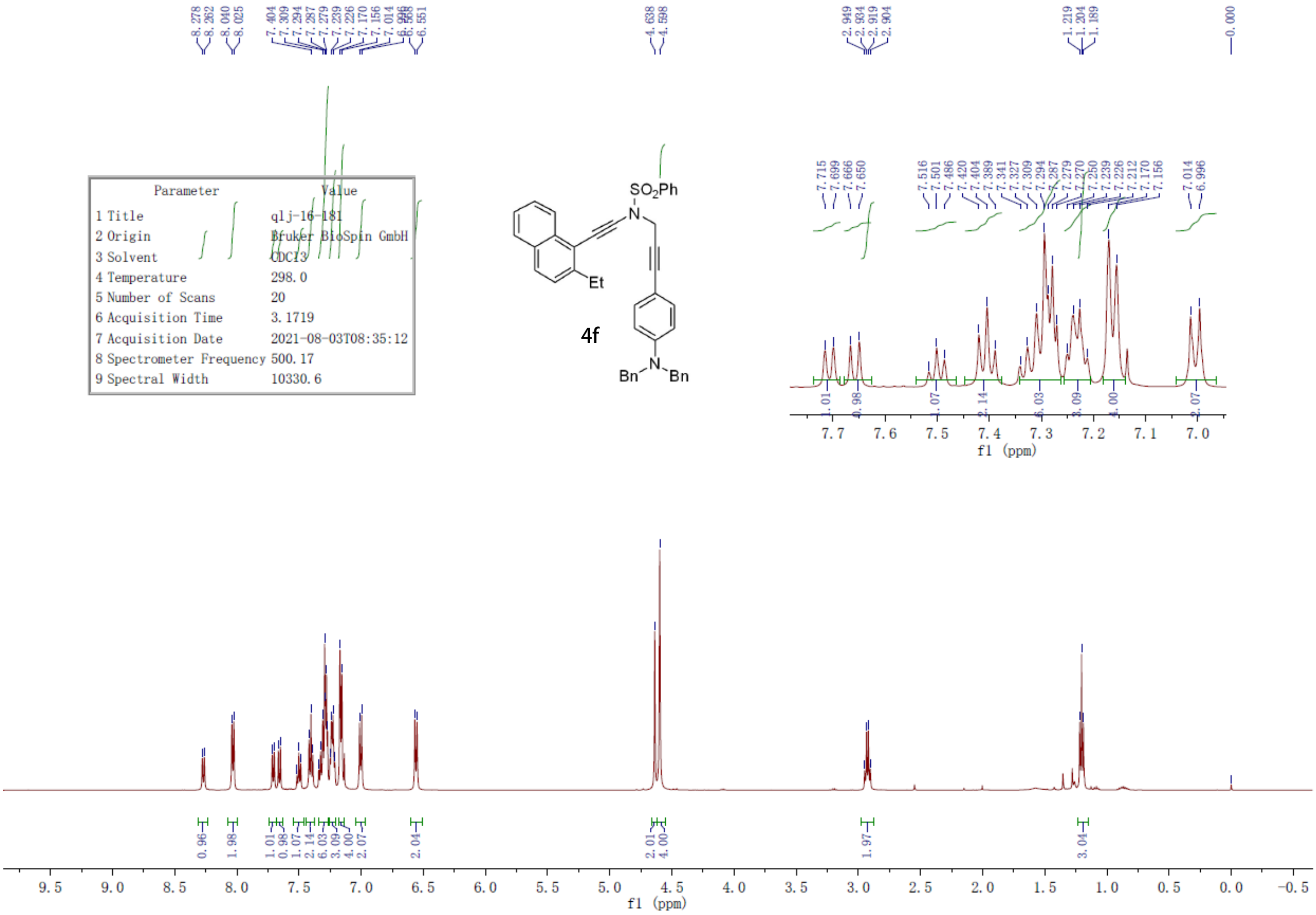

Parameter	Value
1 Title	qlj-16-181
2 Origin	Bruker BioSpin GmbH
3 Solvent	CDCl3
4 Temperature	298.0
5 Number of Scans	20
6 Acquisition Time	3.1719
7 Acquisition Date	2021-08-03T08:35:12
8 Spectrometer Frequency	500.17
9 Spectral Width	10330.6

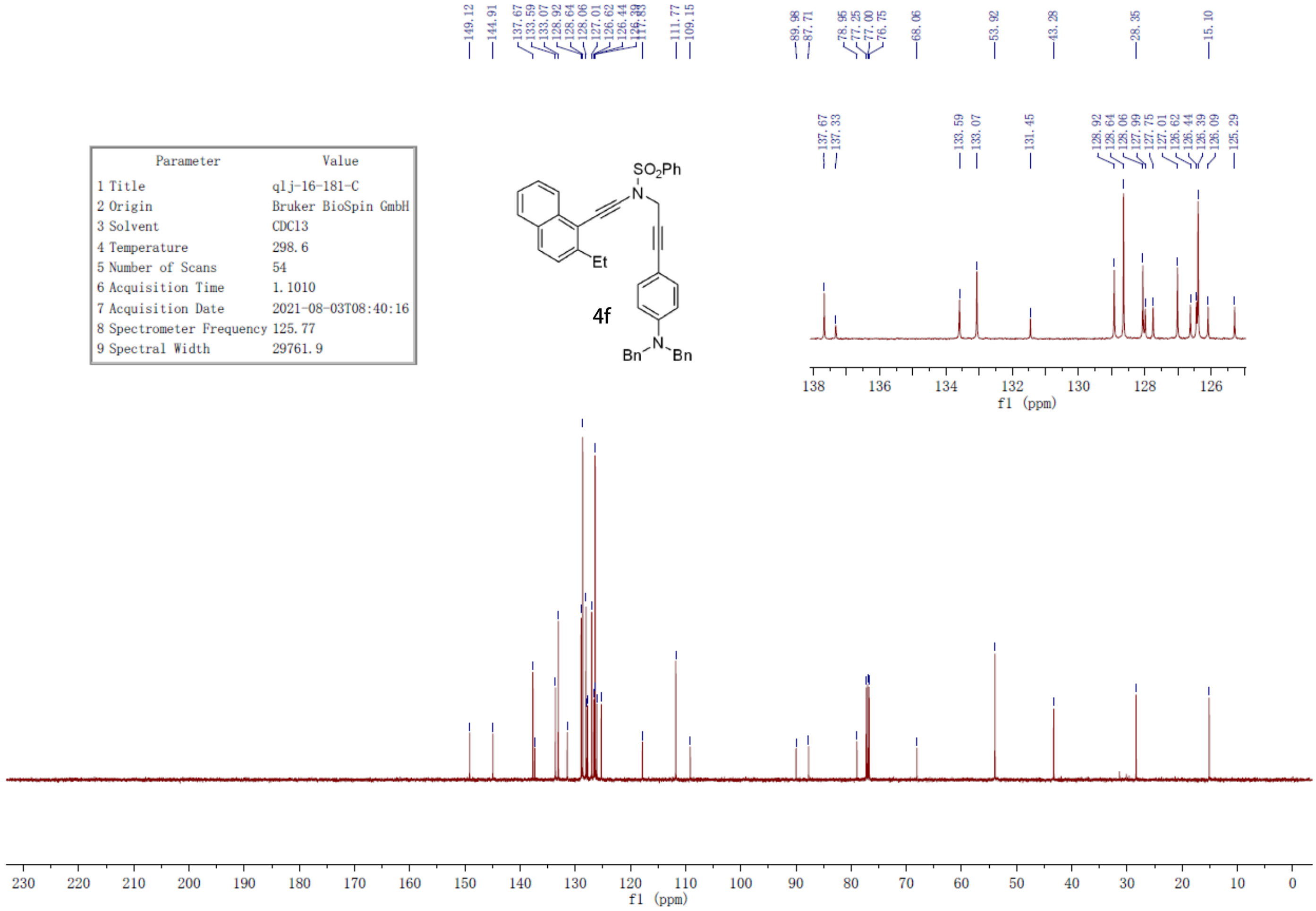

Parameter	Value
1 Title	qlj-16-181-C
2 Origin	Bruker BioSpin GmbH
3 Solvent	CDCl3
4 Temperature	298.6
5 Number of Scans	54
6 Acquisition Time	1.1010
7 Acquisition Date	2021-08-03T08:40:16
8 Spectrometer Frequency	125.77
9 Spectral Width	29761.9

Supplementary Figure 26. ^{1}H and ^{13}C NMR spectra for **4f**

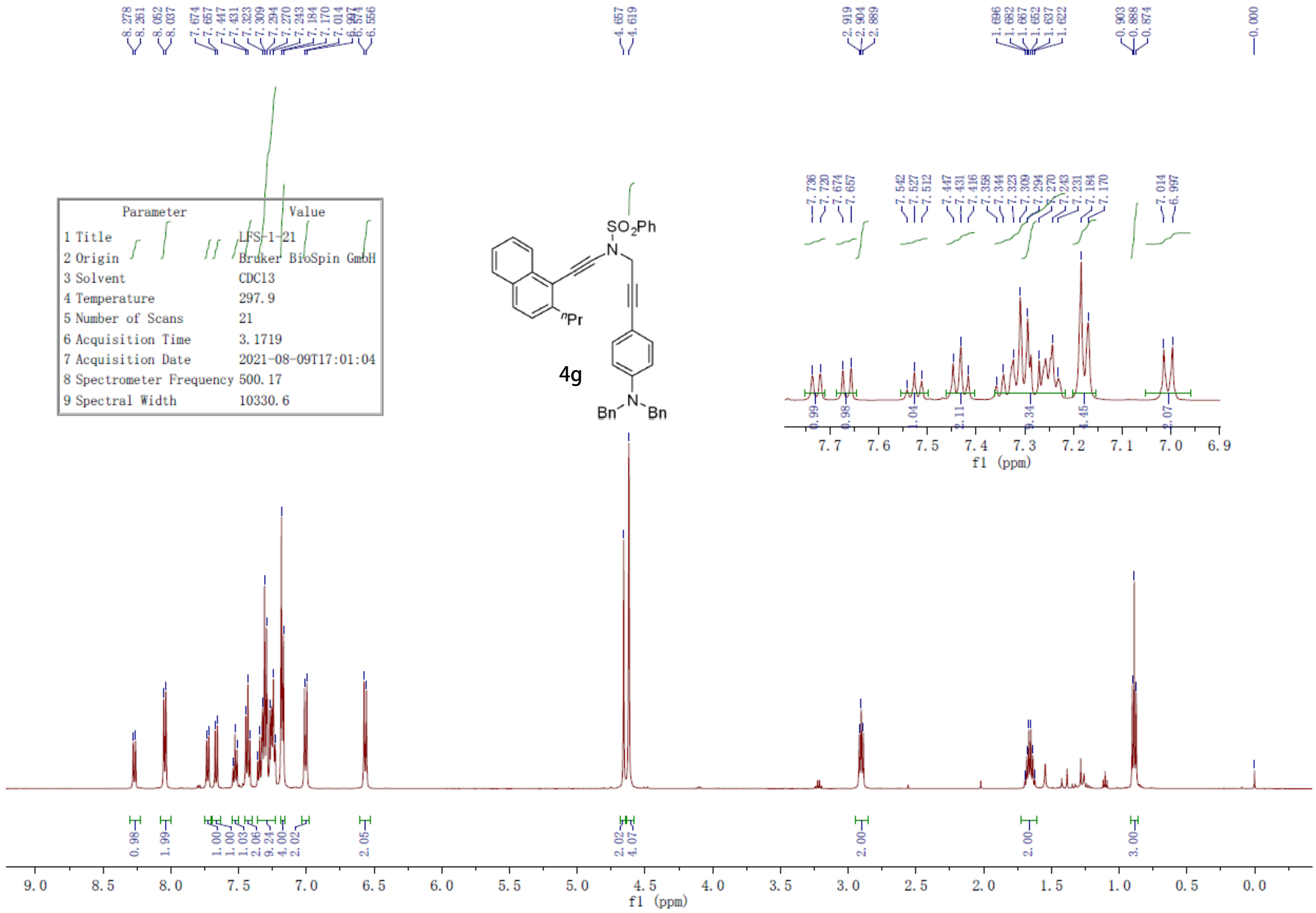

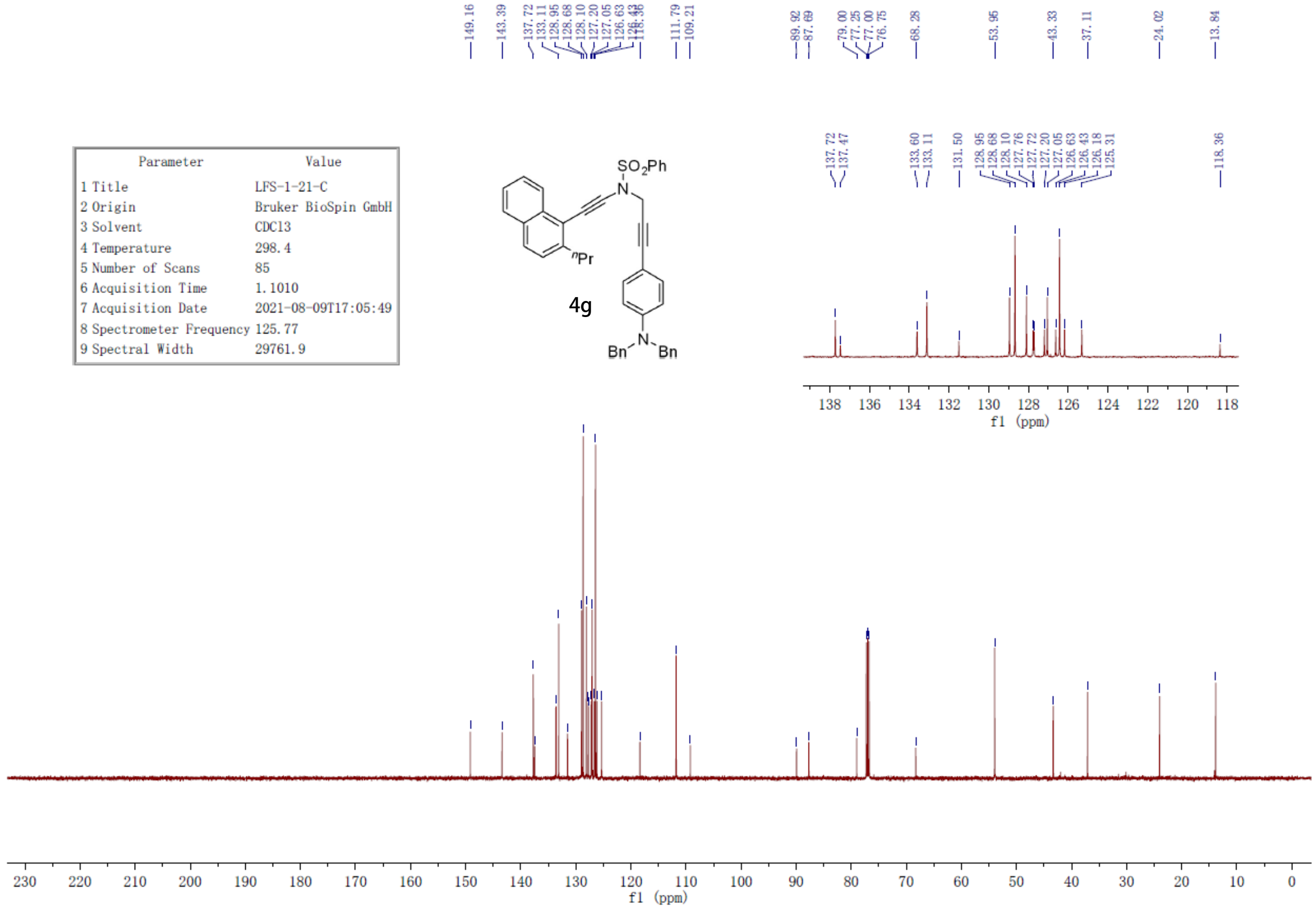

Supplementary Figure 27. ^{1}H and ^{13}C NMR spectra for **4g**

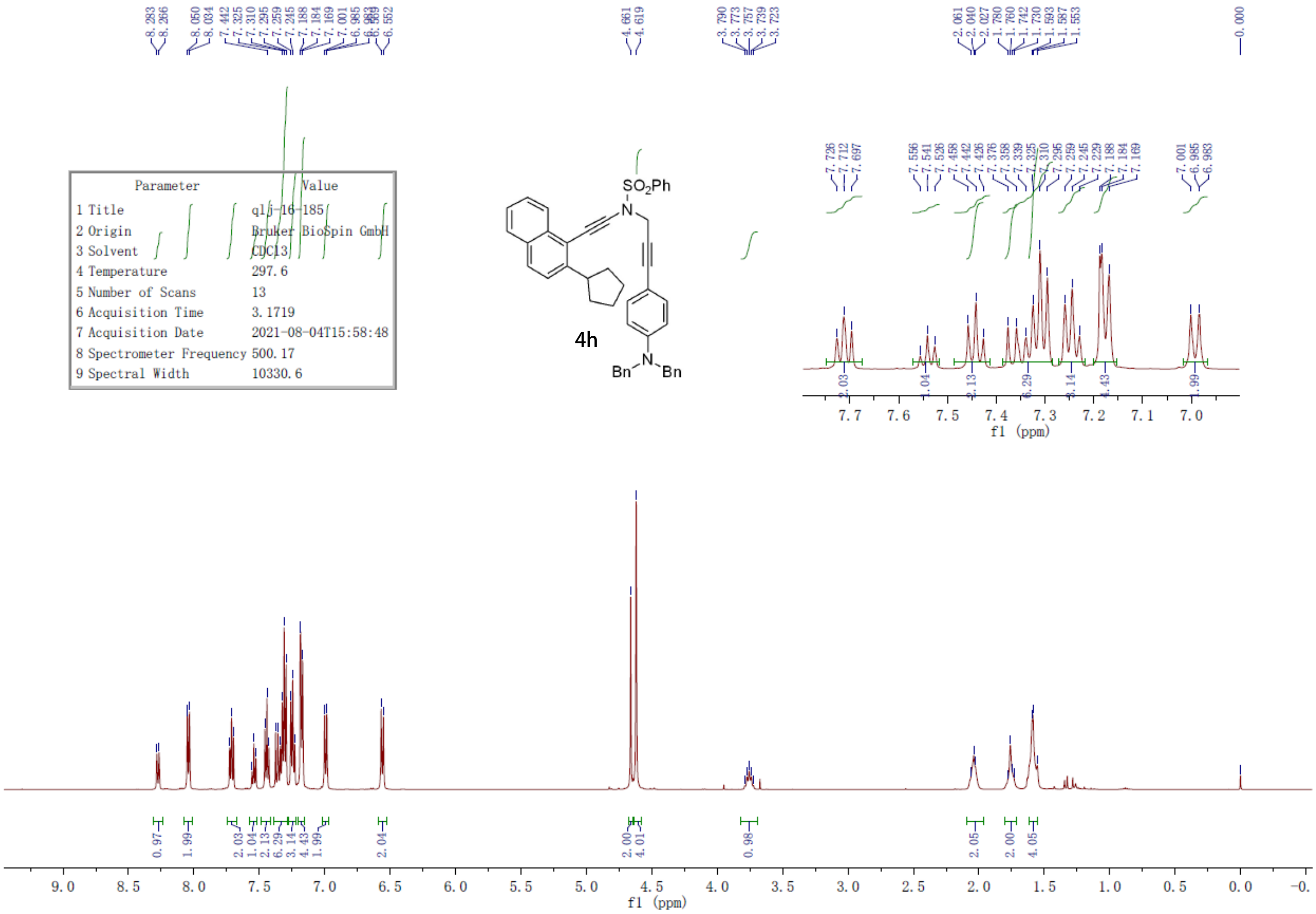

Parameter	Value
1 Title	qlj-16-185
2 Origin	Bruker BioSpin GmbH
3 Solvent	CDCl3
4 Temperature	297.6
5 Number of Scans	13
6 Acquisition Time	3.1719
7 Acquisition Date	2021-08-04T15:58:48
8 Spectrometer Frequency	500.17
9 Spectral Width	10330.6

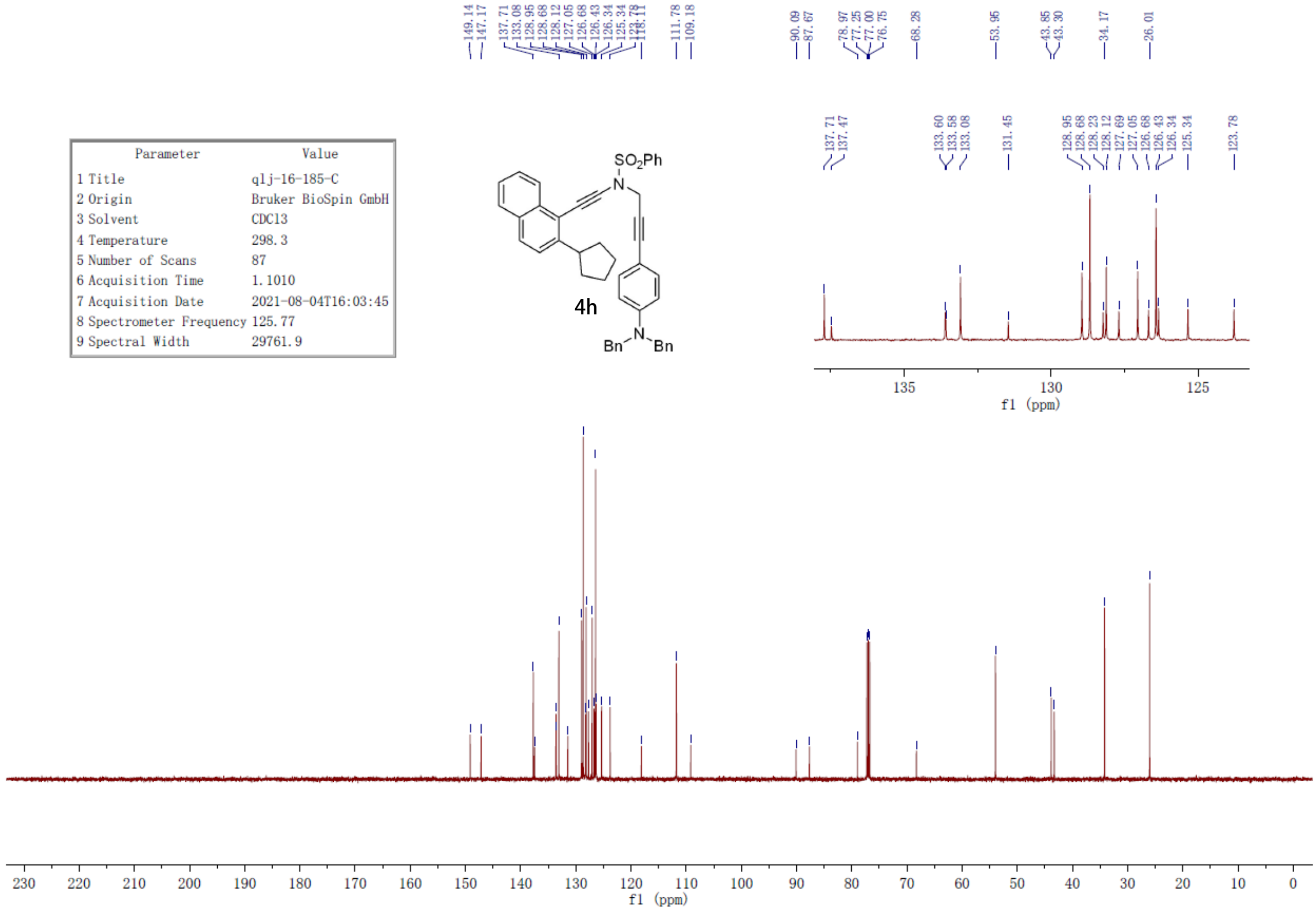

Parameter	Value
1 Title	qlj-16-185-C
2 Origin	Bruker BioSpin GmbH
3 Solvent	CDCl3
4 Temperature	298.3
5 Number of Scans	87
6 Acquisition Time	1.1010
7 Acquisition Date	2021-08-04T16:03:45
8 Spectrometer Frequency	125.77
9 Spectral Width	29761.9

Supplementary Figure 28. ^{1}H and ^{13}C NMR spectra for **4h**

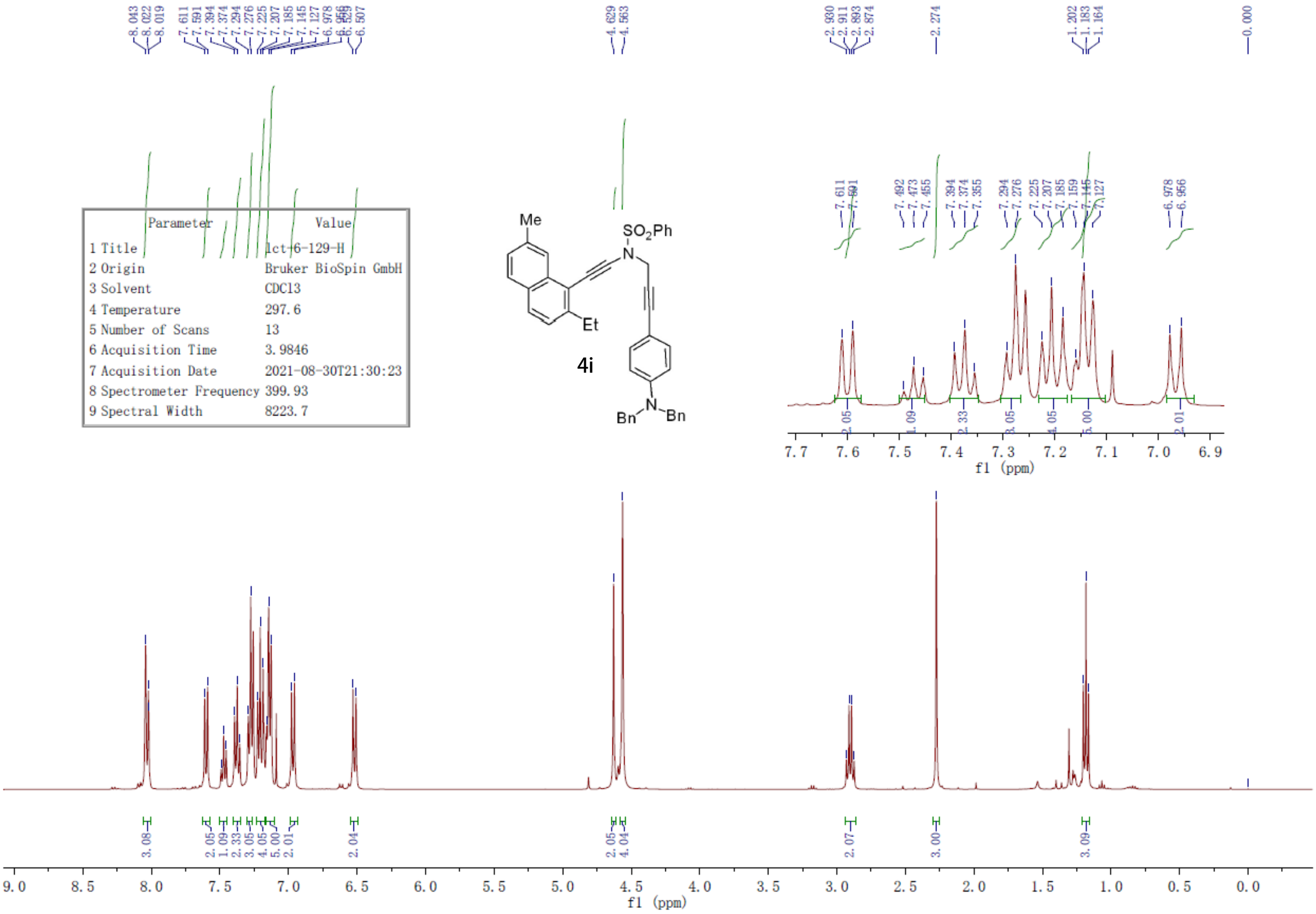

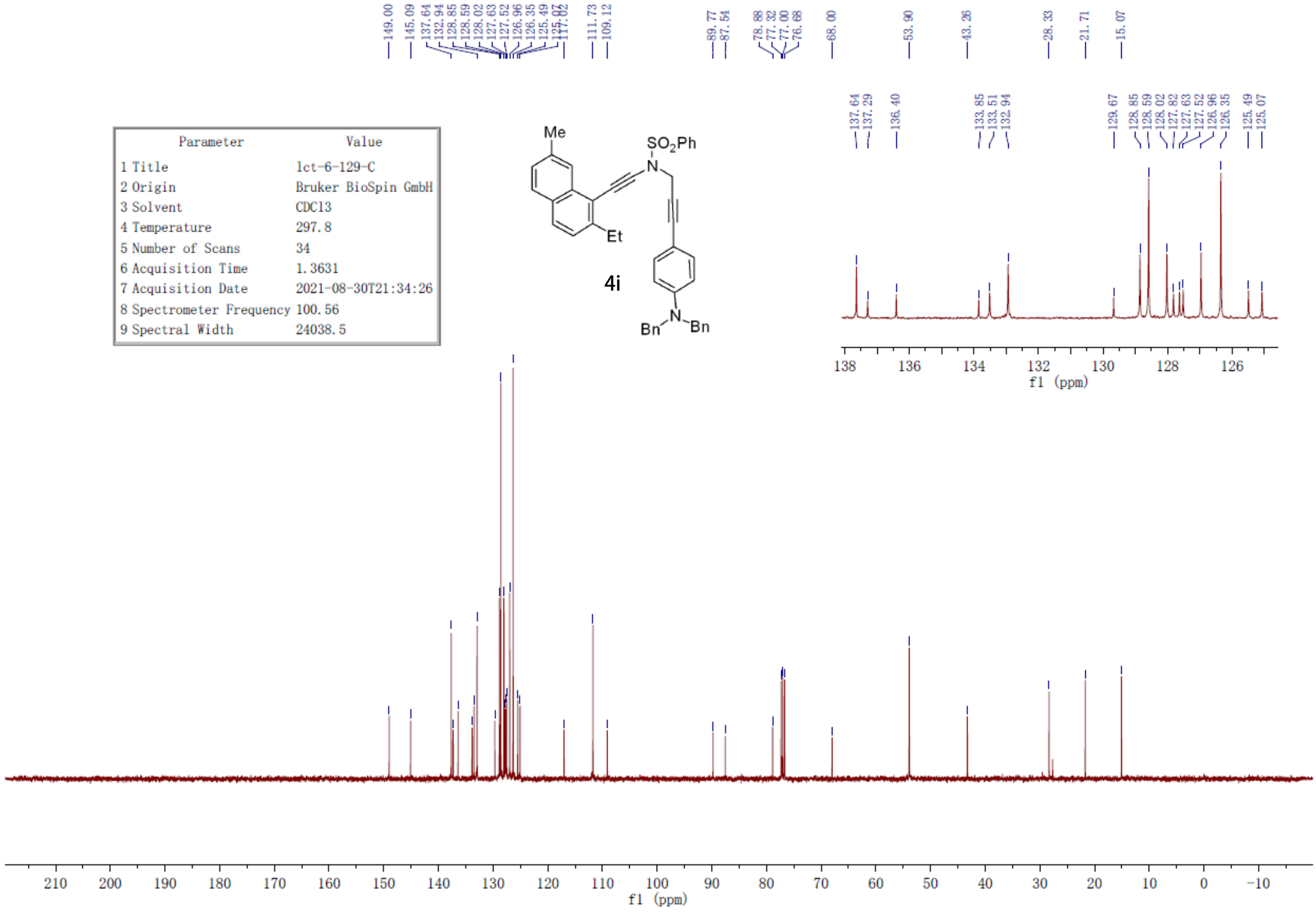

Supplementary Figure 29. ^{1}H and ^{13}C NMR spectra for **4i**

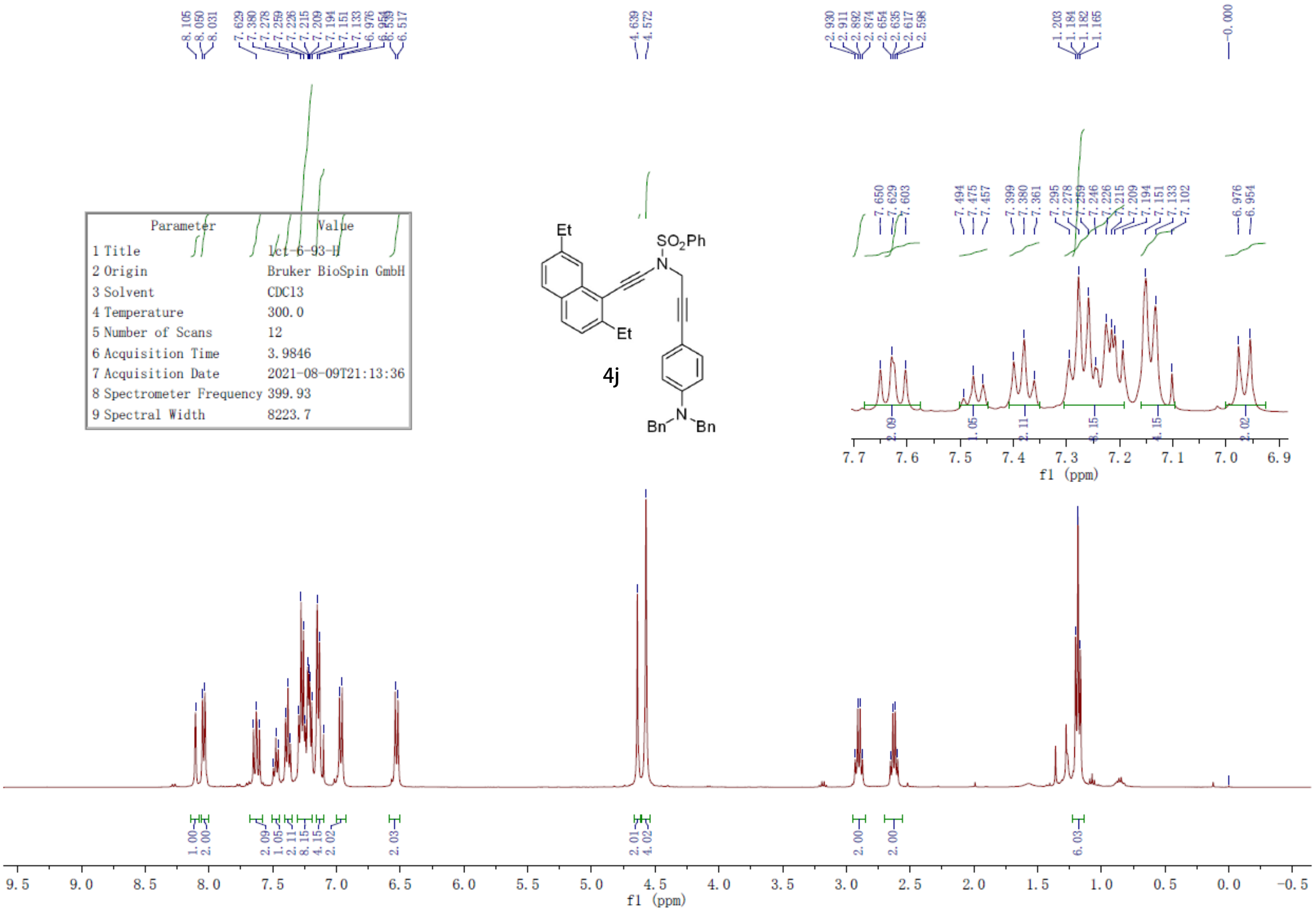

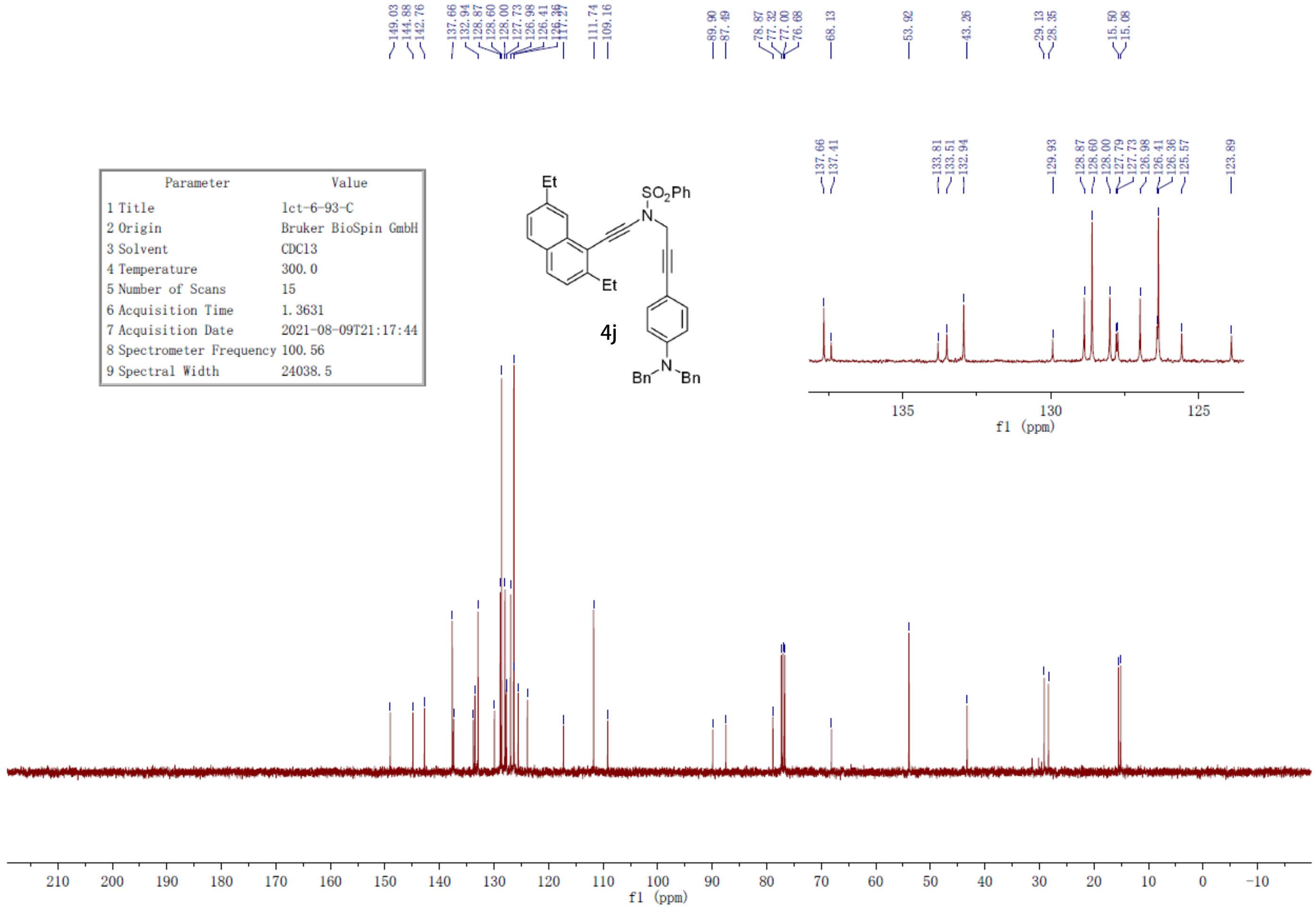

Supplementary Figure 30. ^{1}H and ^{13}C NMR spectra for **4j**

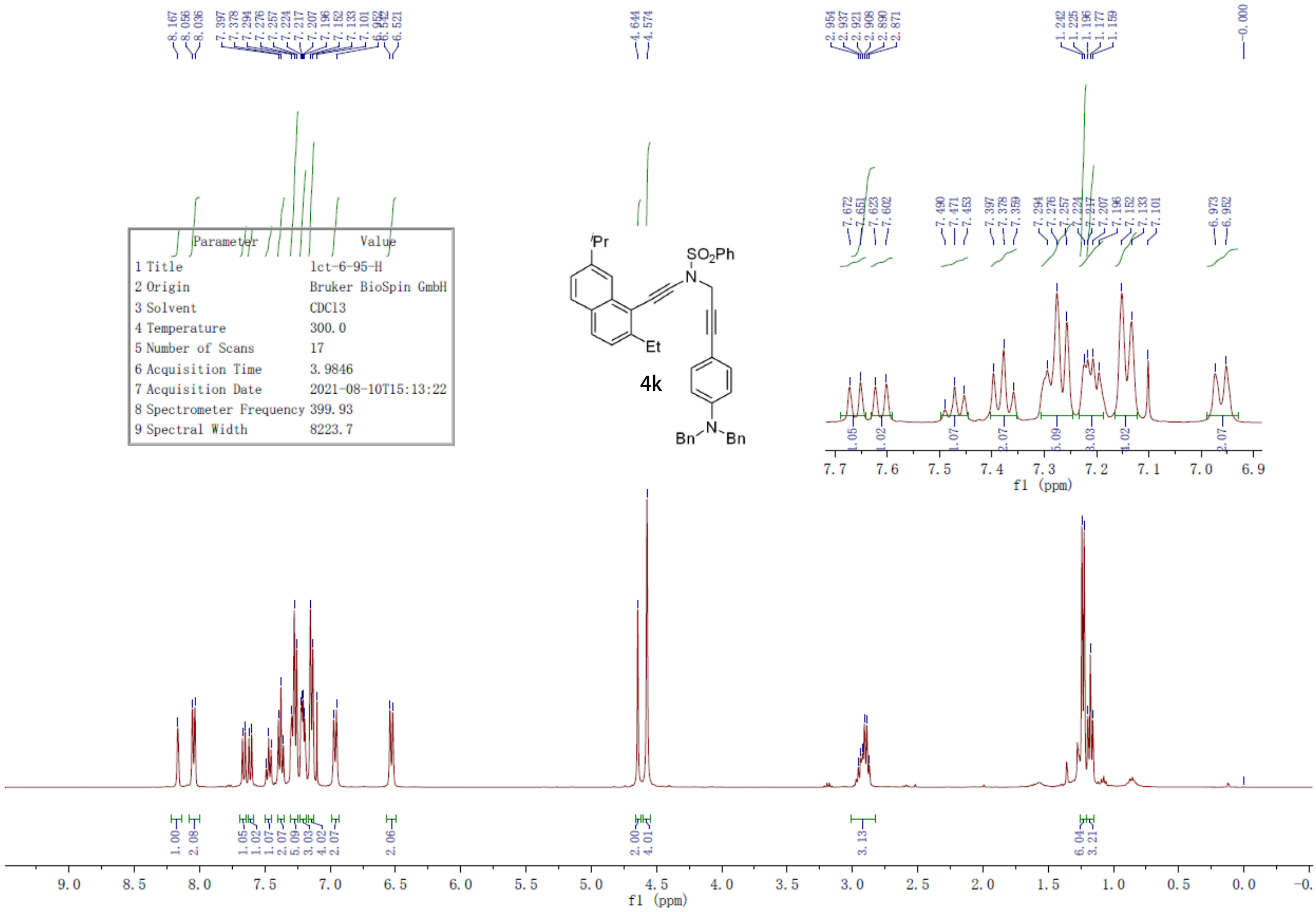

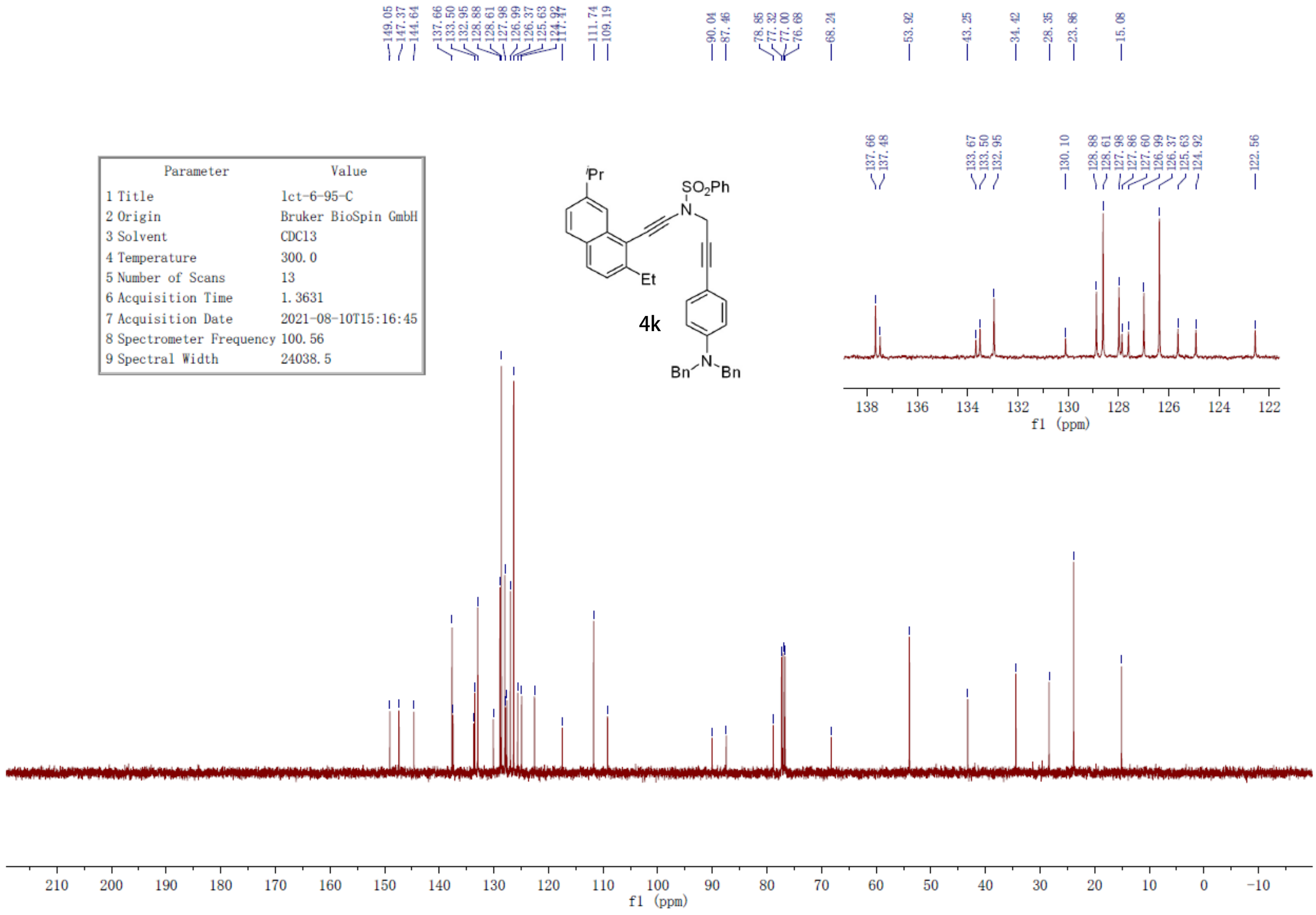

Supplementary Figure 31. ^{1}H and ^{13}C NMR spectra for **4k**

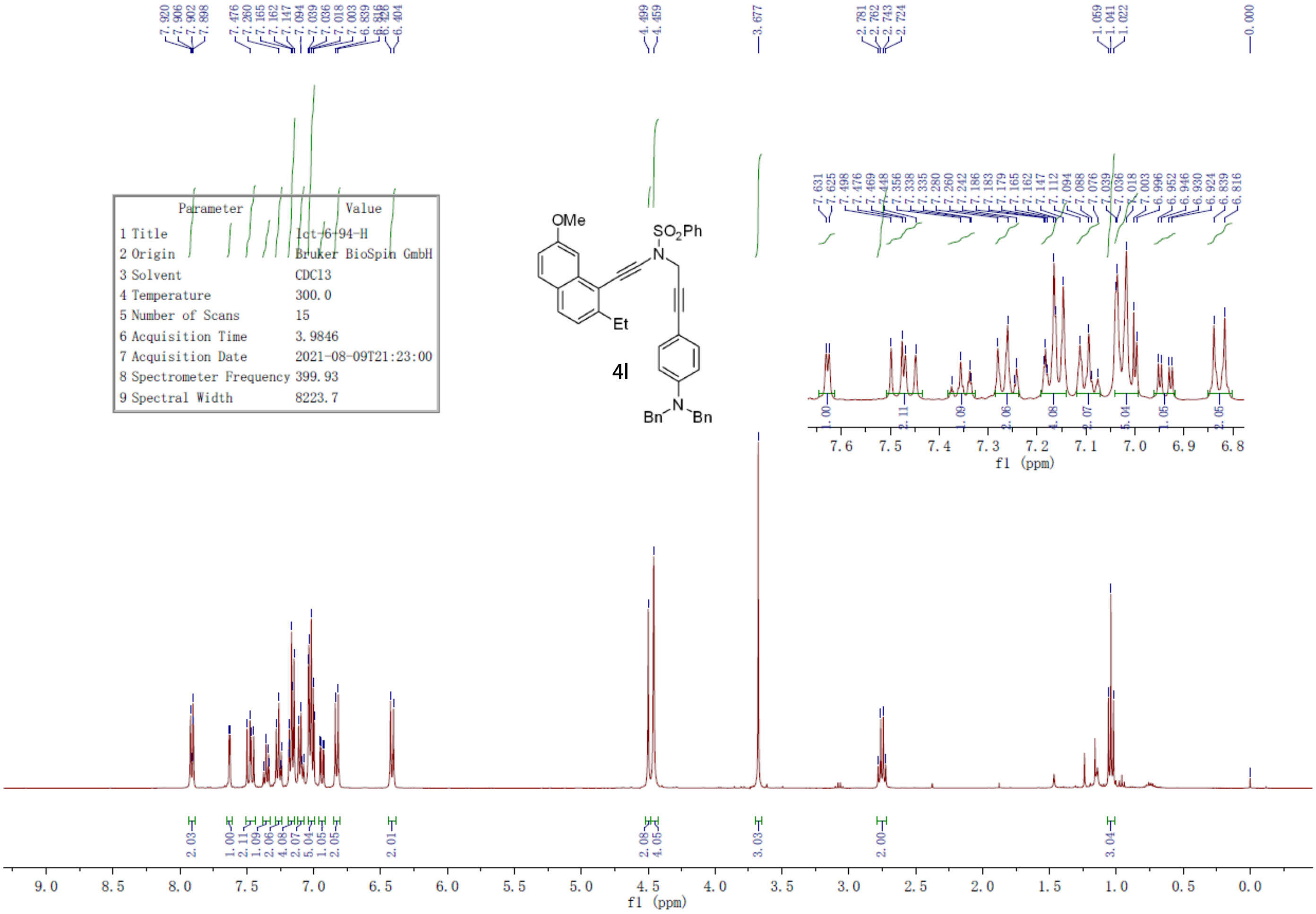

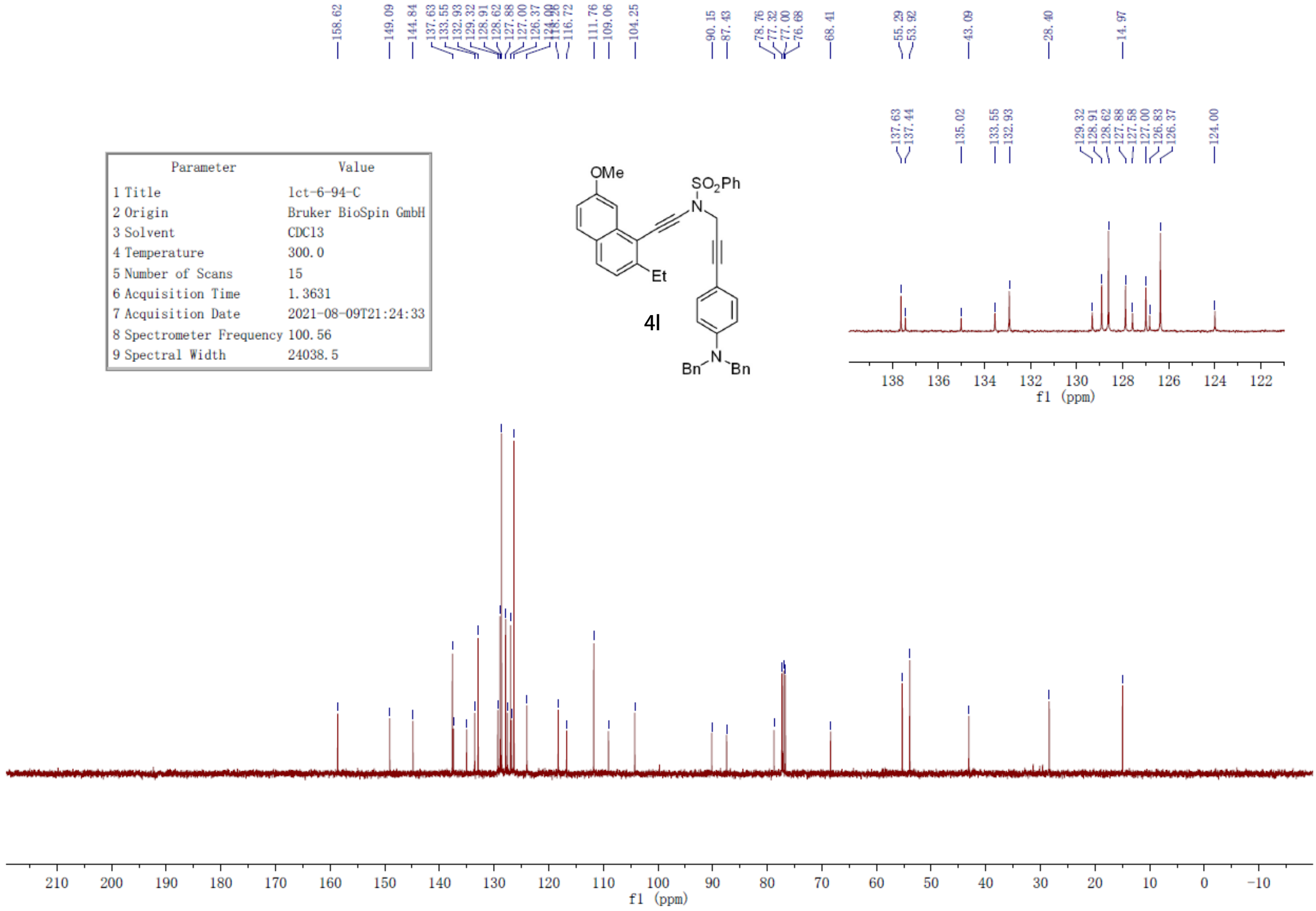

Supplementary Figure 32. ^{1}H and ^{13}C NMR spectra for **4l**

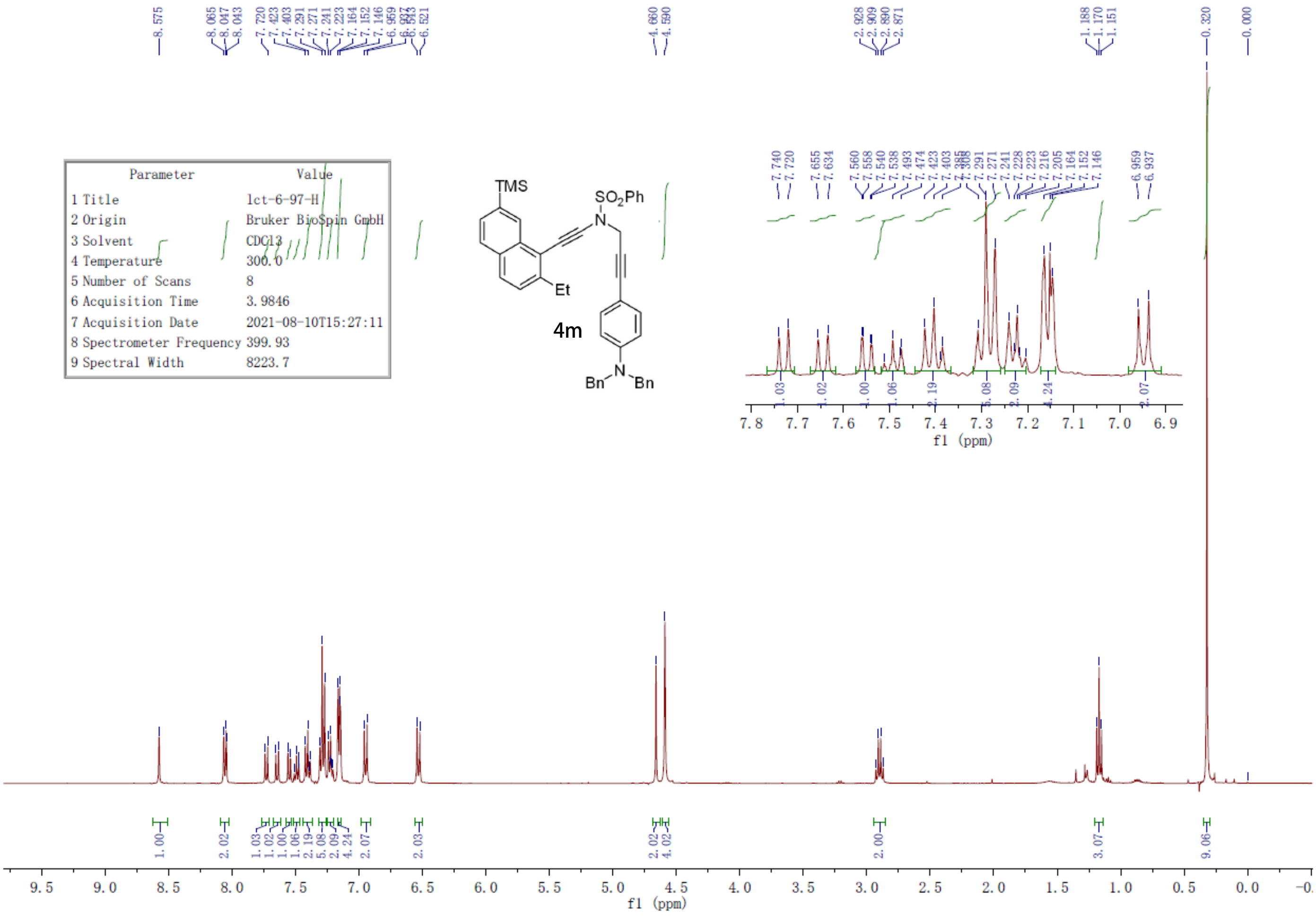

Parameter	Value
1 Title	lct-6-97-H
2 Origin	Bruker BioSpin GmbH
3 Solvent	CDCl3
4 Temperature	300.0
5 Number of Scans	8
6 Acquisition Time	3.9846
7 Acquisition Date	2021-08-10T15:27:11
8 Spectrometer Frequency	399.93
9 Spectral Width	8223.7

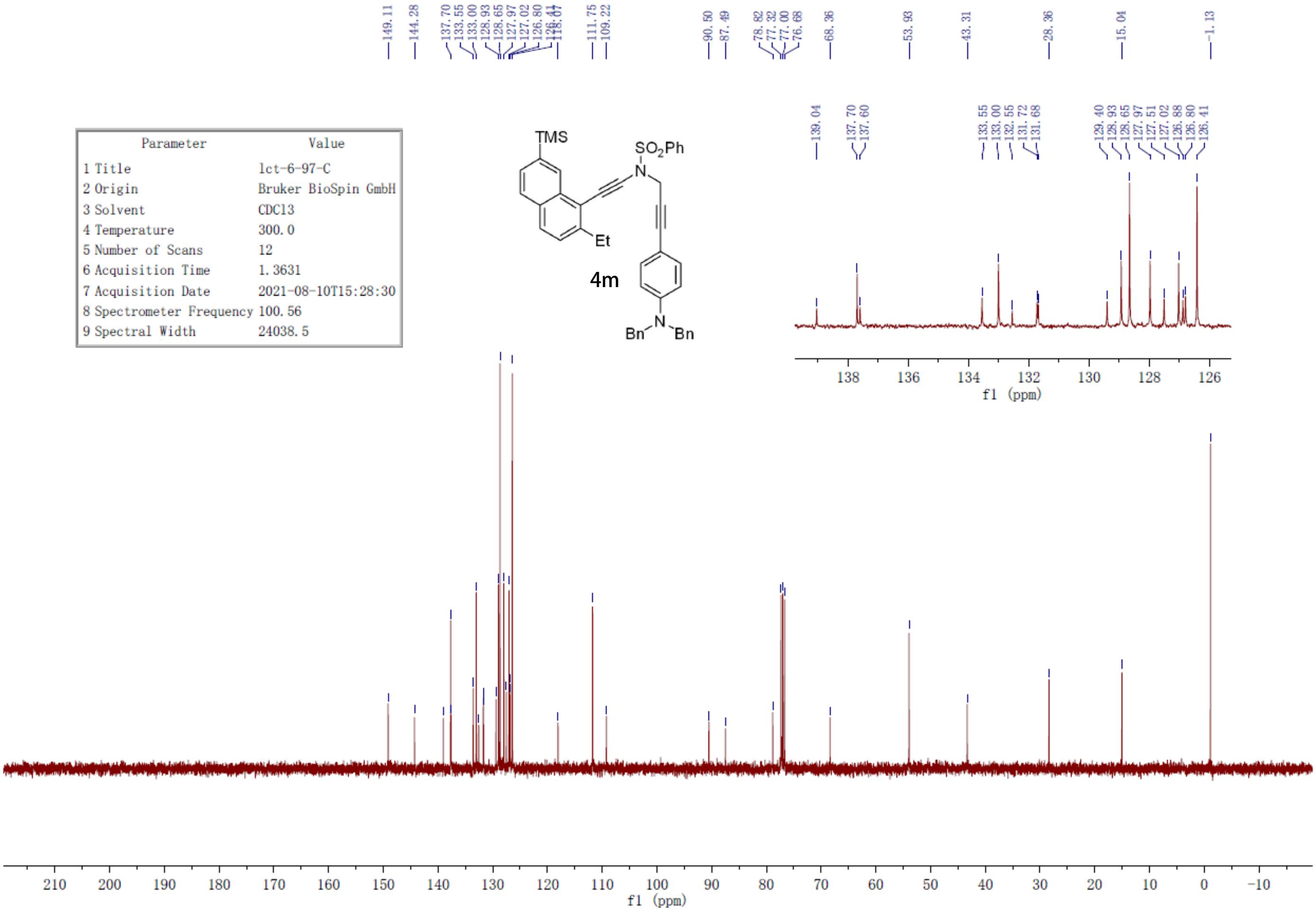

Parameter	Value
1 Title	lct-6-97-C
2 Origin	Bruker BioSpin GmbH
3 Solvent	CDCl3
4 Temperature	300.0
5 Number of Scans	12
6 Acquisition Time	1.3631
7 Acquisition Date	2021-08-10T15:28:30
8 Spectrometer Frequency	100.56
9 Spectral Width	24038.5

Supplementary Figure 33. ^{1}H and ^{13}C NMR spectra for **4m**

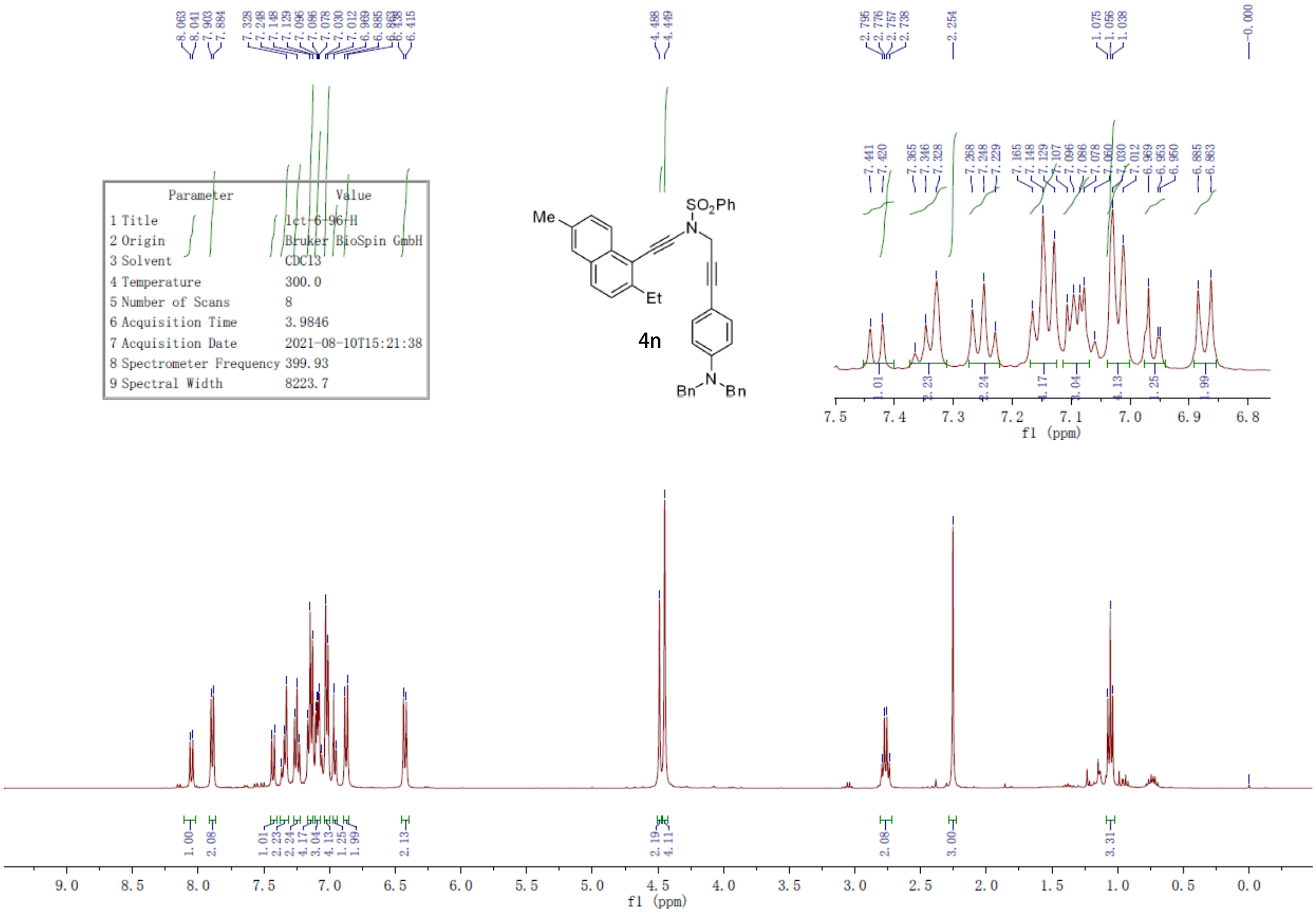

Parameter	Value
1 Title	lct-6-96-H
2 Origin	Bruker BioSpin GmbH
3 Solvent	CDCl3
4 Temperature	300.0
5 Number of Scans	8
6 Acquisition Time	3.9846
7 Acquisition Date	2021-08-10T15:21:38
8 Spectrometer Frequency	399.93
9 Spectral Width	8223.7

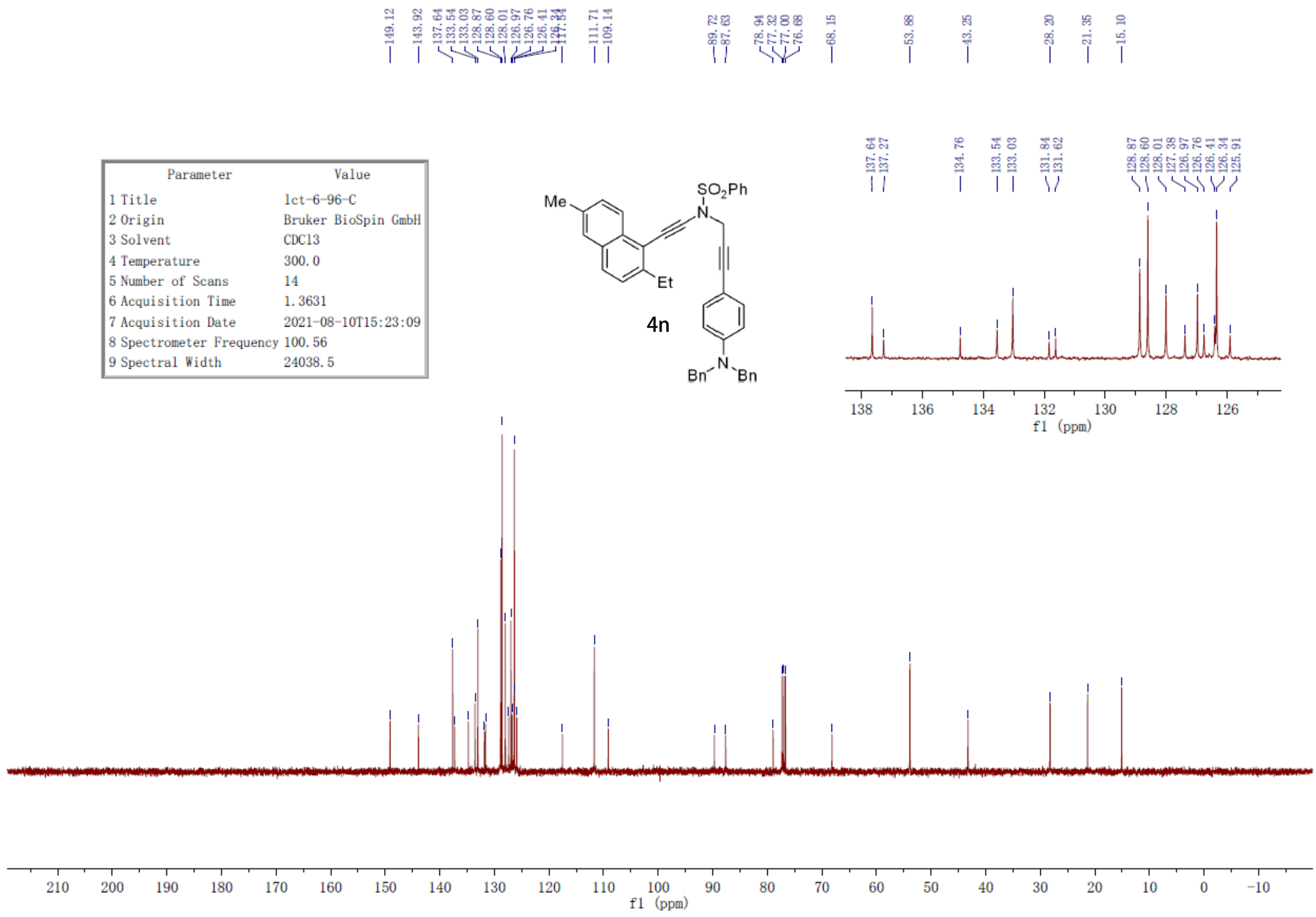

Parameter	Value
1 Title	lct-6-96-C
2 Origin	Bruker BioSpin GmbH
3 Solvent	CDCl3
4 Temperature	300.0
5 Number of Scans	14
6 Acquisition Time	1.3631
7 Acquisition Date	2021-08-10T15:23:09
8 Spectrometer Frequency	100.56
9 Spectral Width	24038.5

Supplementary Figure 34. ^{1}H and ^{13}C NMR spectra for **4n**

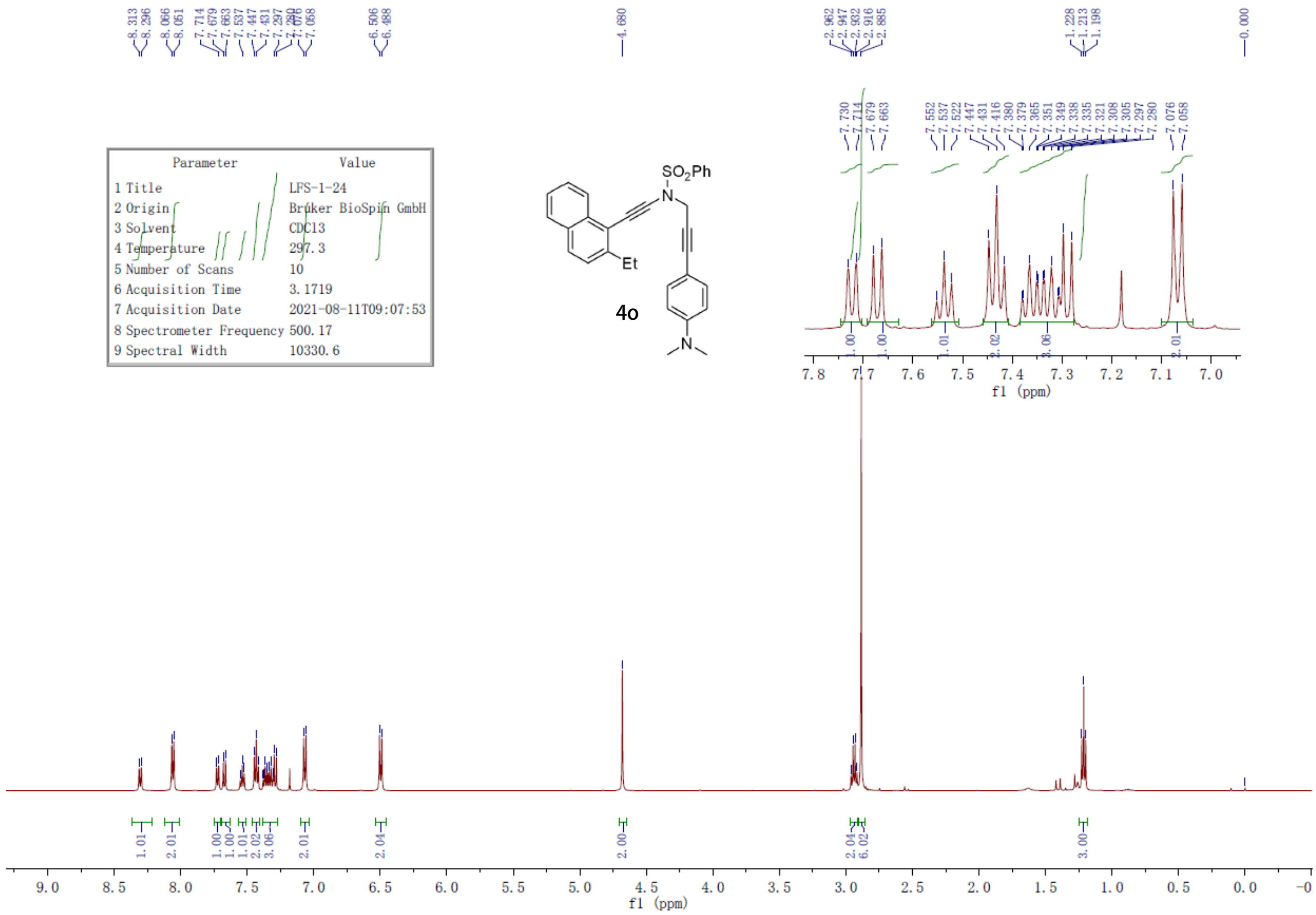

Parameter	Value
1 Title	LFS-1-24
2 Origin	Bruker BioSpin GmbH
3 Solvent	CDCl3
4 Temperature	297.3
5 Number of Scans	10
6 Acquisition Time	3.1719
7 Acquisition Date	2021-08-11T09:07:53
8 Spectrometer Frequency	500.17
9 Spectral Width	10330.6

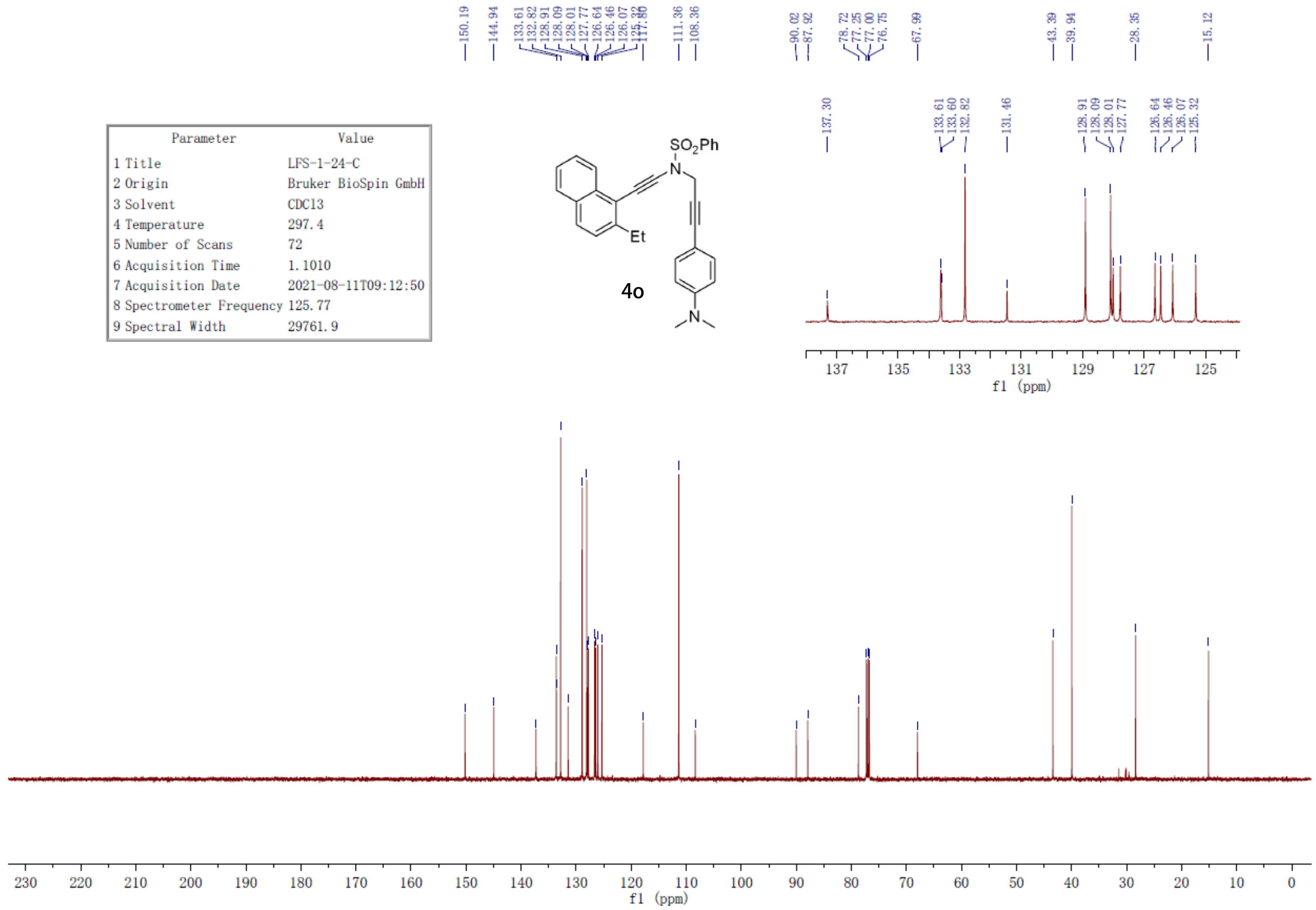

Parameter	Value
1 Title	LFS-1-24-C
2 Origin	Bruker BioSpin GmbH
3 Solvent	CDCl3
4 Temperature	297.4
5 Number of Scans	72
6 Acquisition Time	1.1010
7 Acquisition Date	2021-08-11T09:12:50
8 Spectrometer Frequency	125.77
9 Spectral Width	29761.9

Supplementary Figure 35. ^{1}H and ^{13}C NMR spectra for **4o**

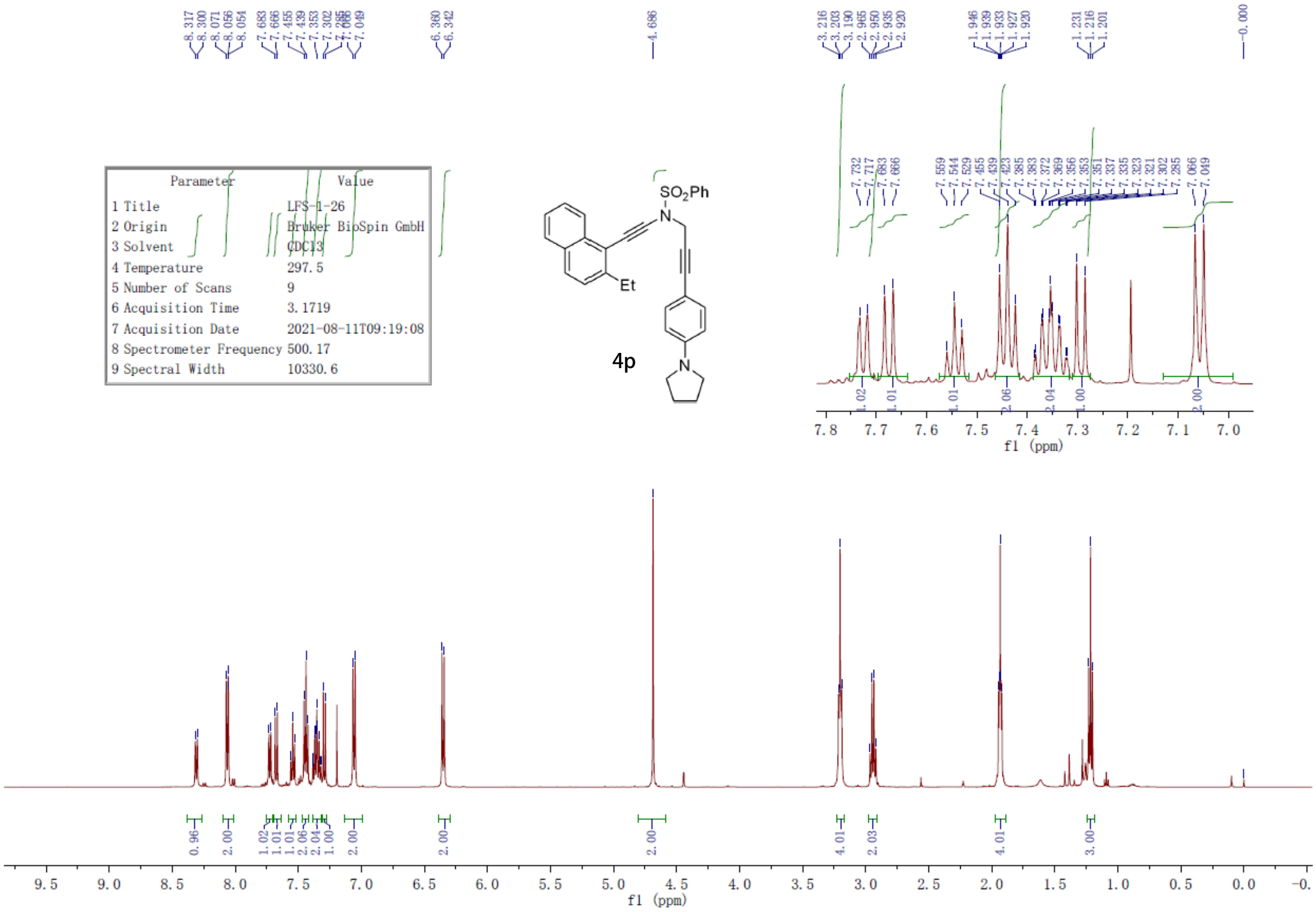

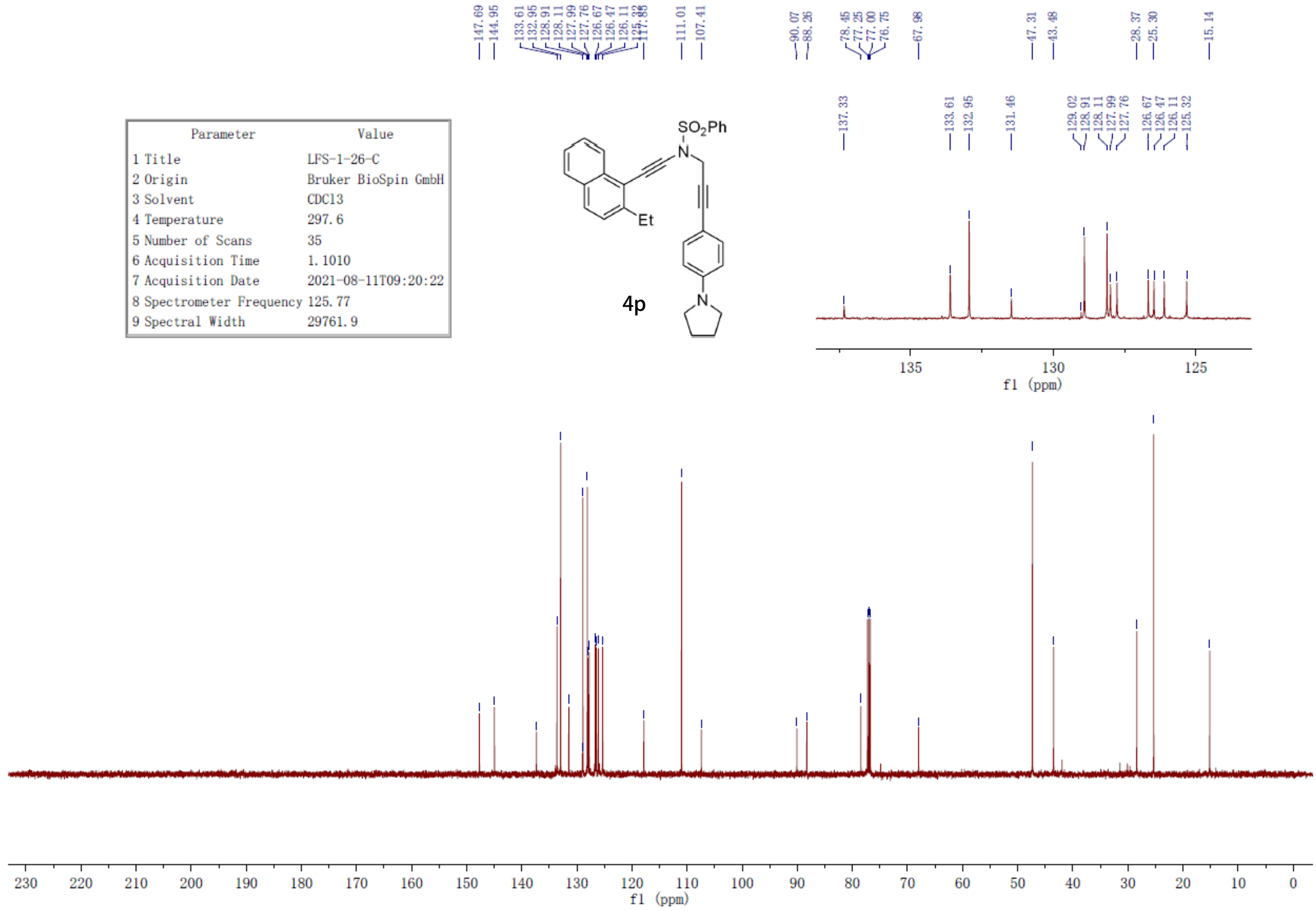

Supplementary Figure 36. ^{1}H and ^{13}C NMR spectra for **4p**

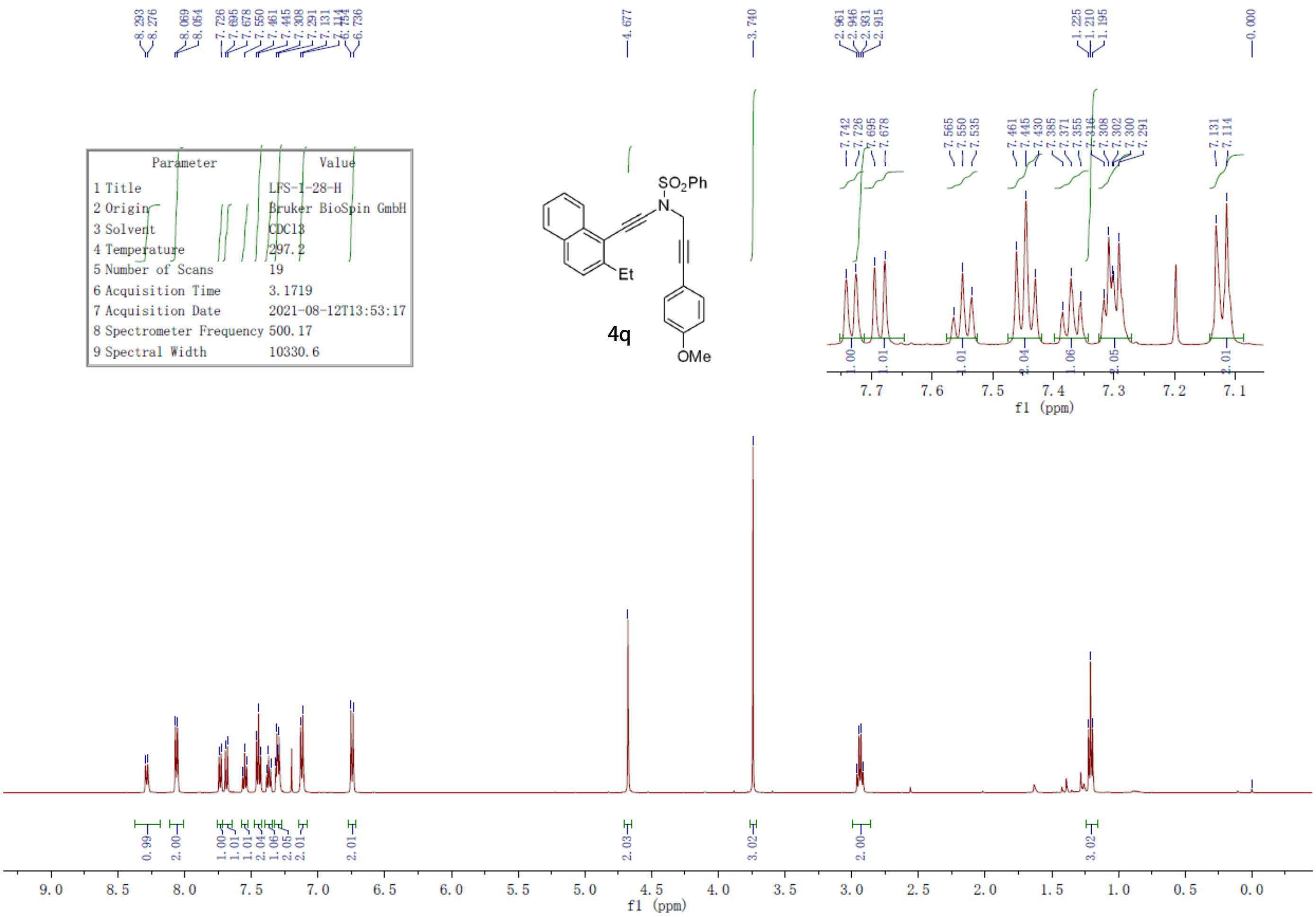

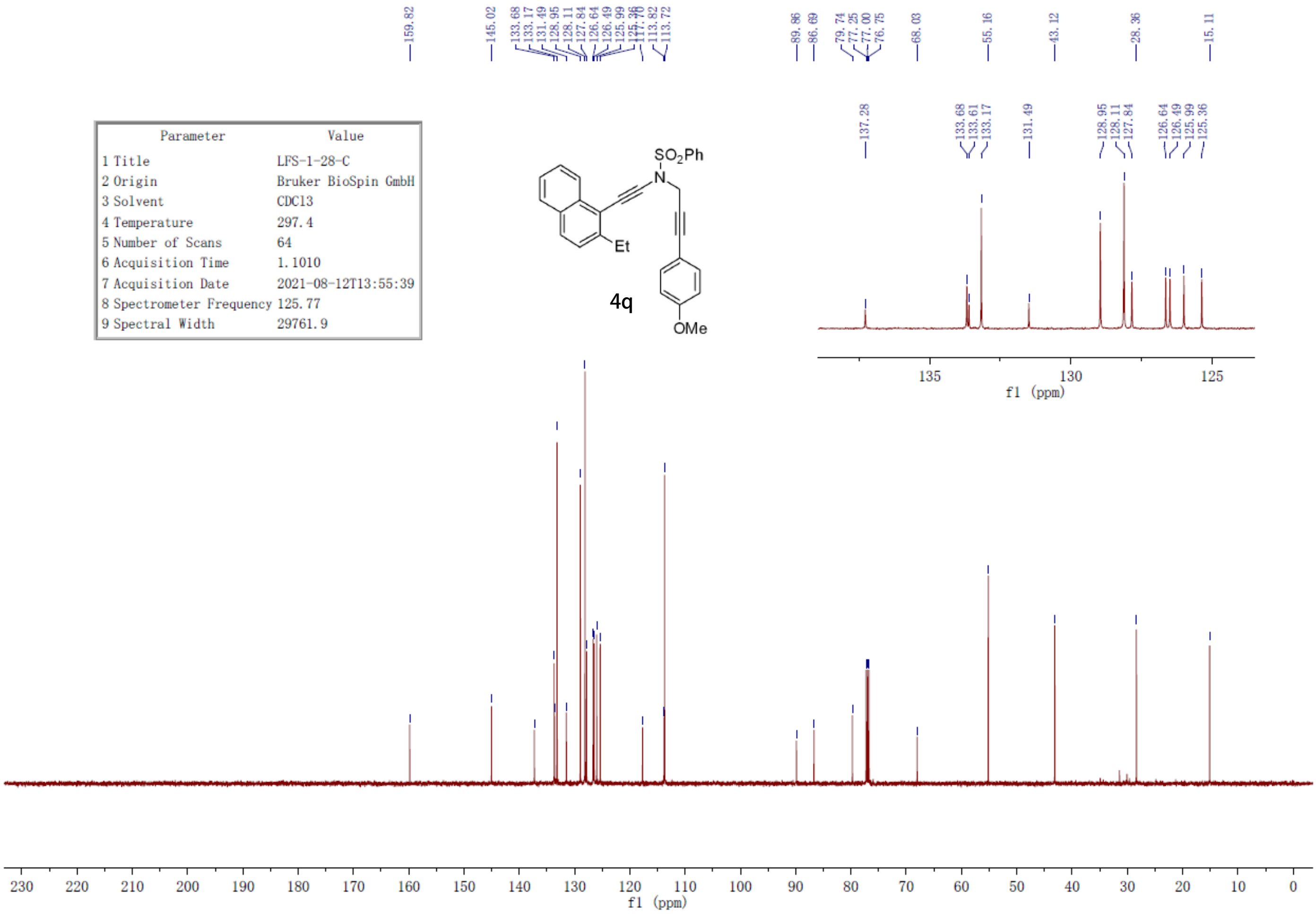

Supplementary Figure 37. ^{1}H and ^{13}C NMR spectra for **4q**

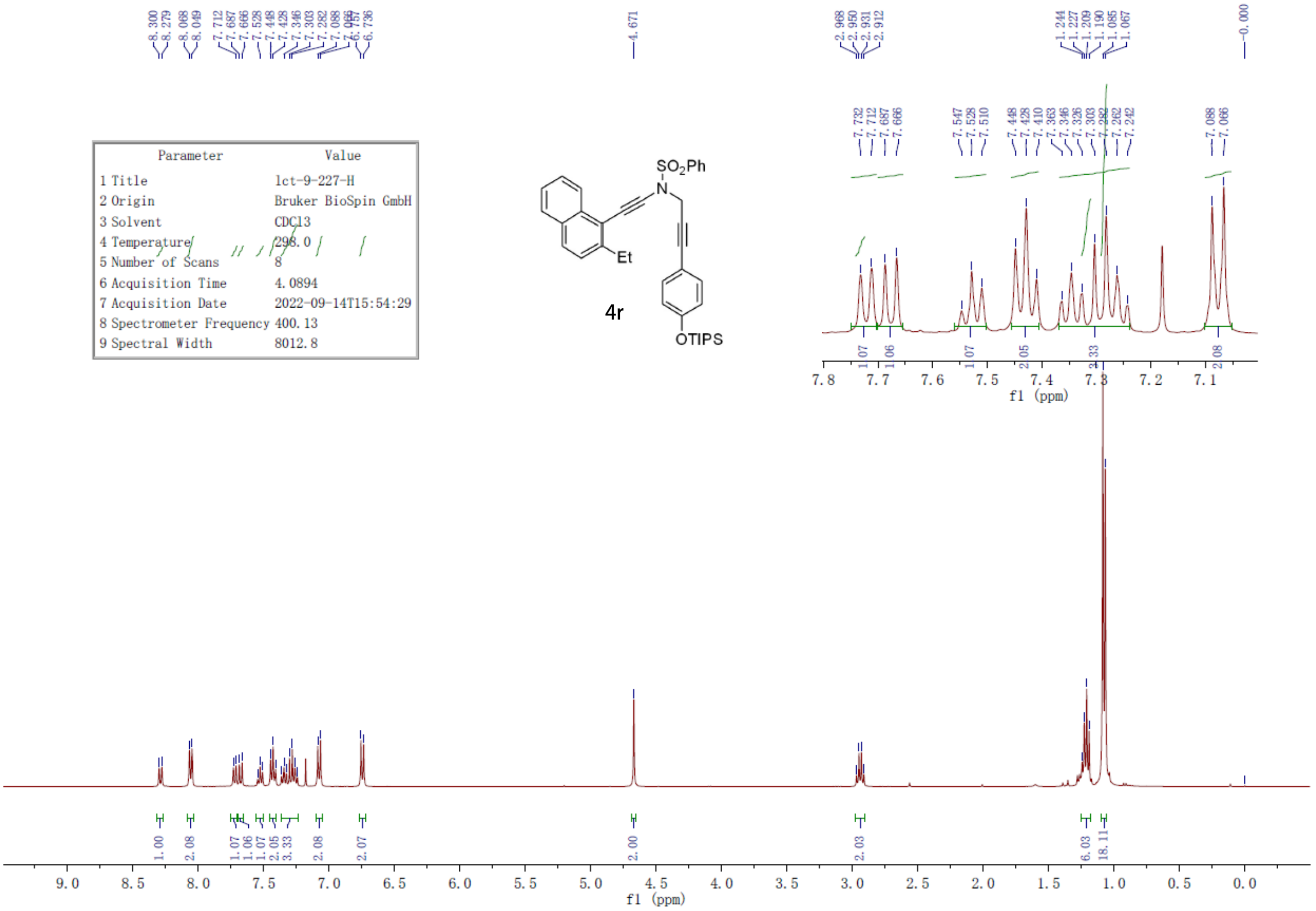

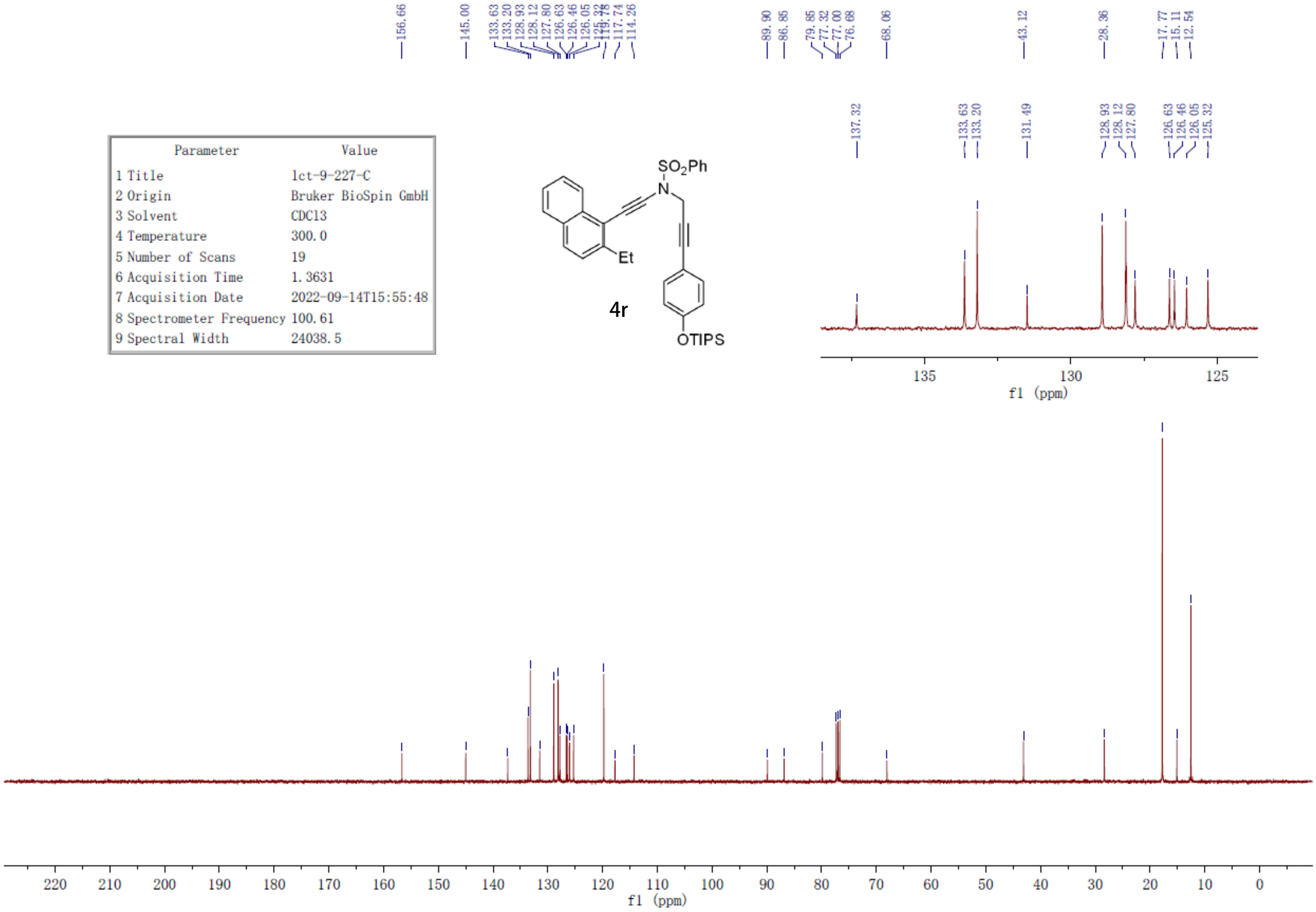

Supplementary Figure 38. ^{1}H and ^{13}C NMR spectra for **4r**

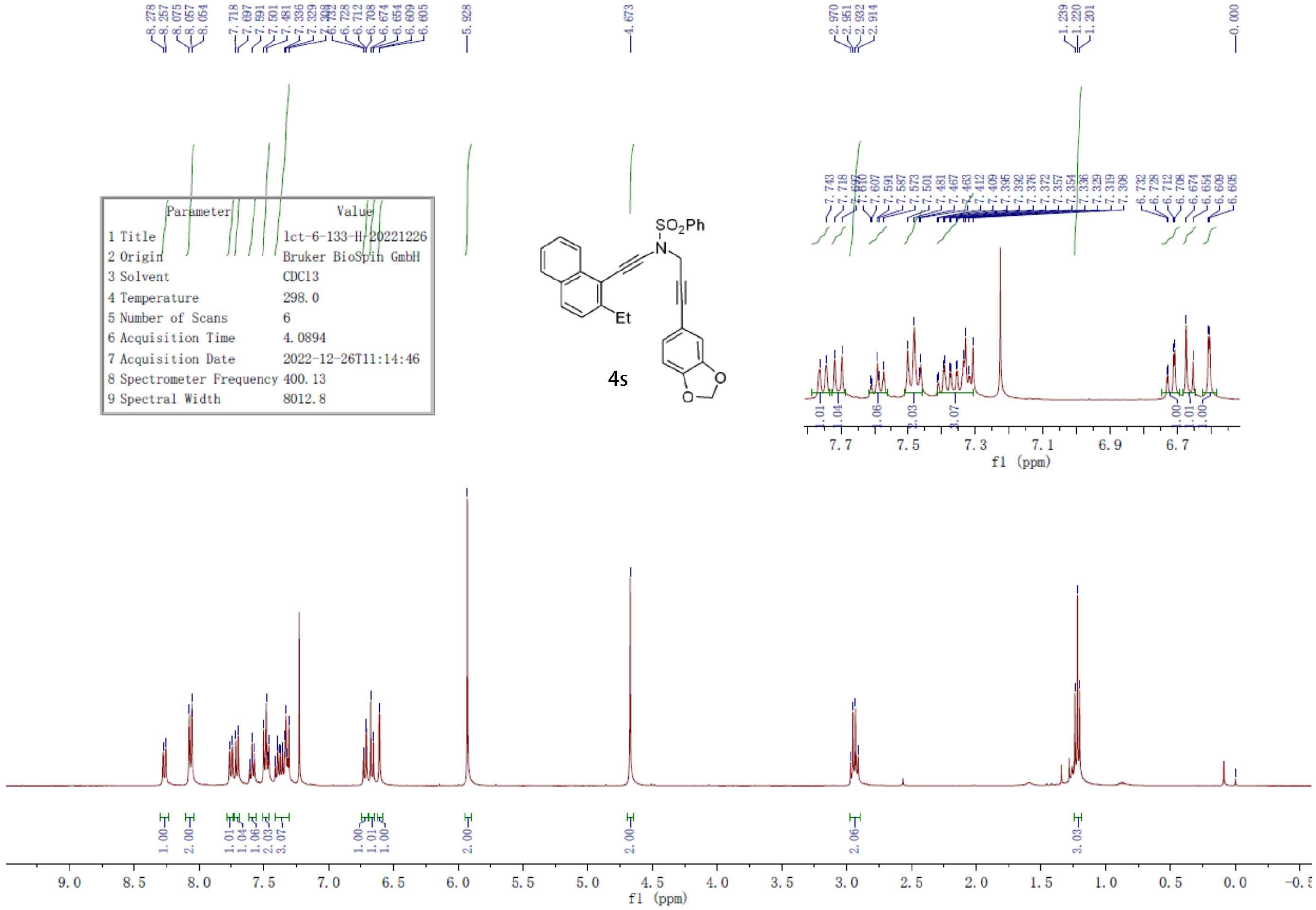

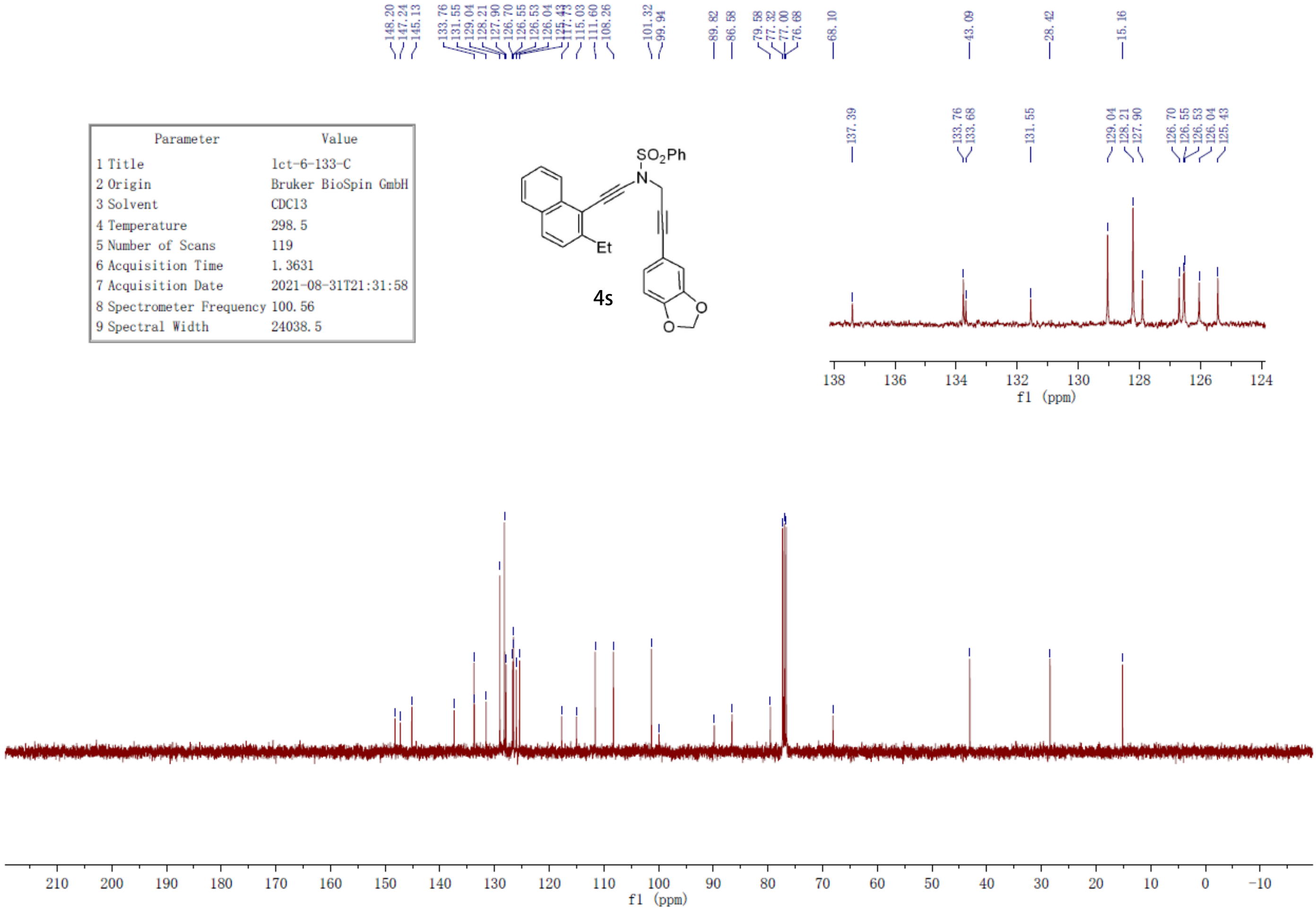

Supplementary Figure 39. ^{1}H and ^{13}C NMR spectra for **4s**

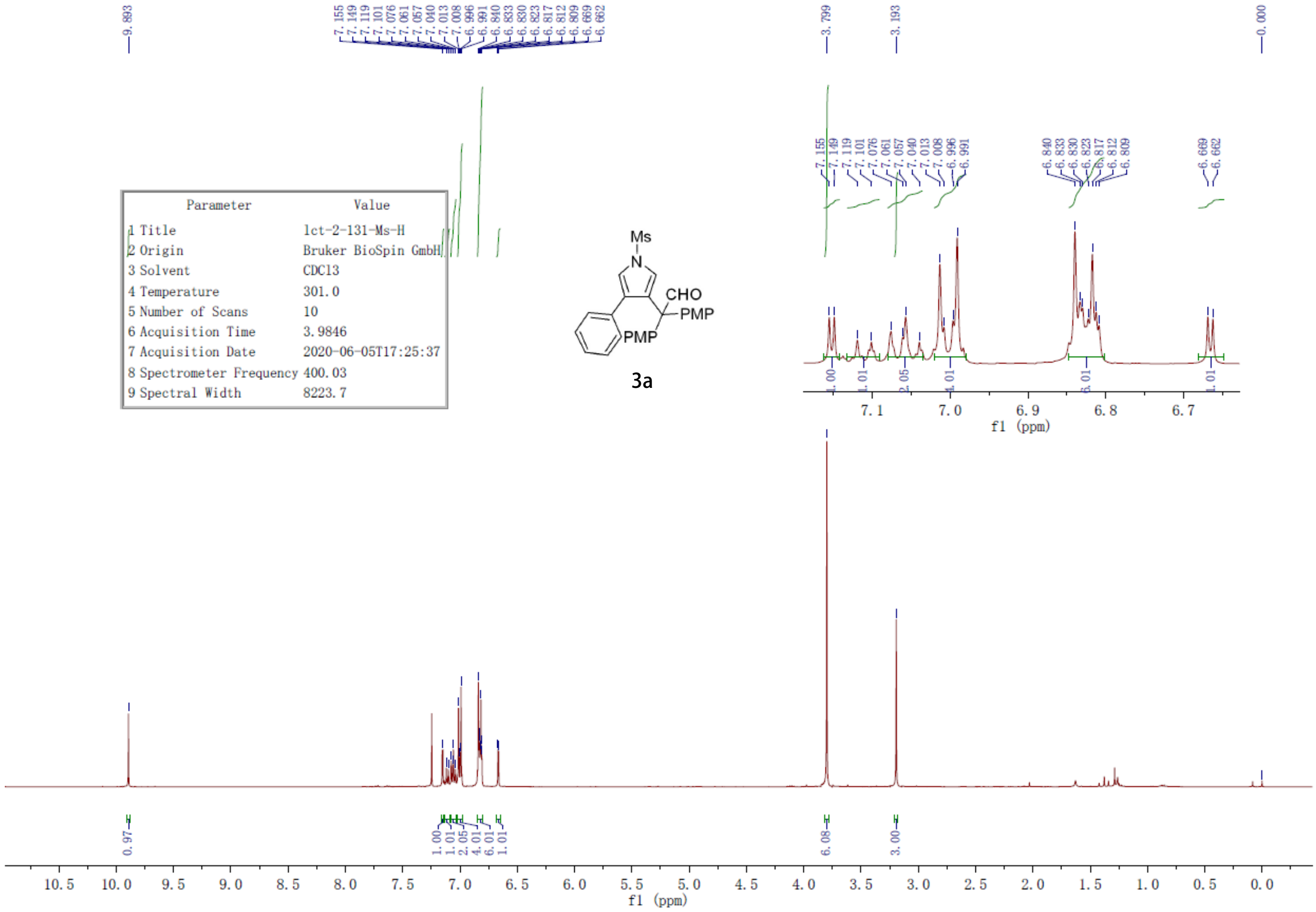

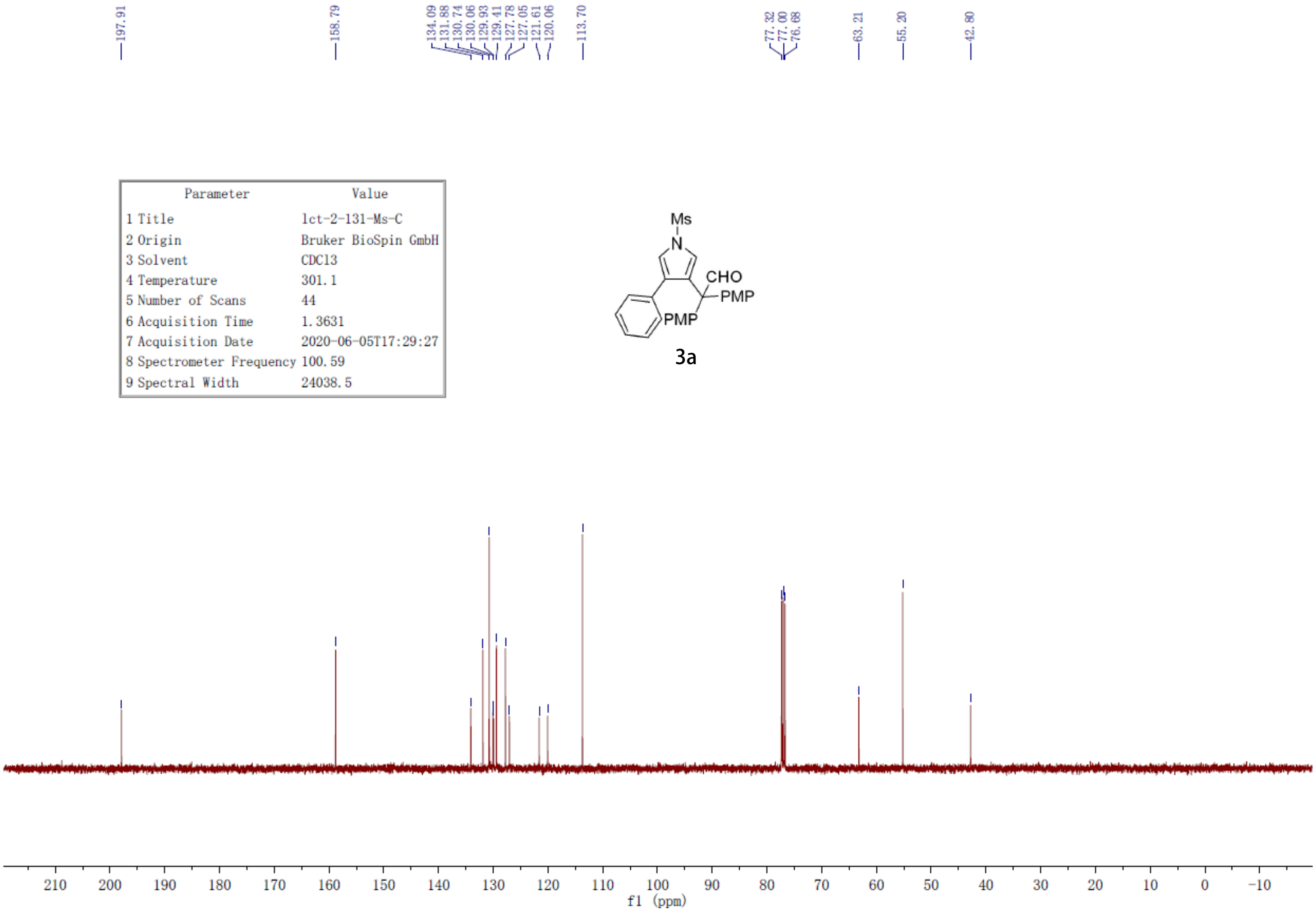

Supplementary Figure 40. ^{1}H and ^{13}C NMR spectra for **3a**

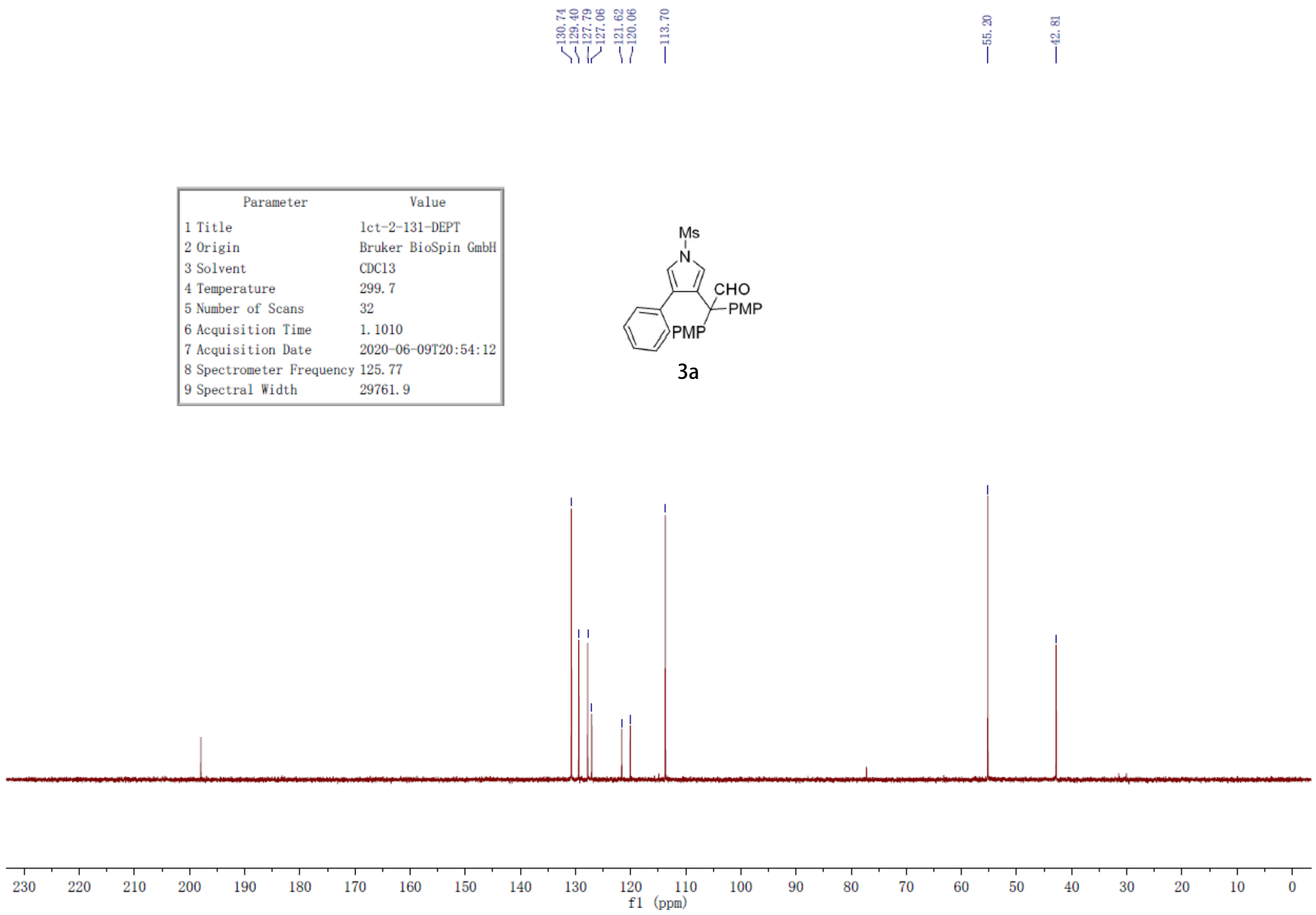

Supplementary Figure 41. DEPT 135 spectra for **3a**

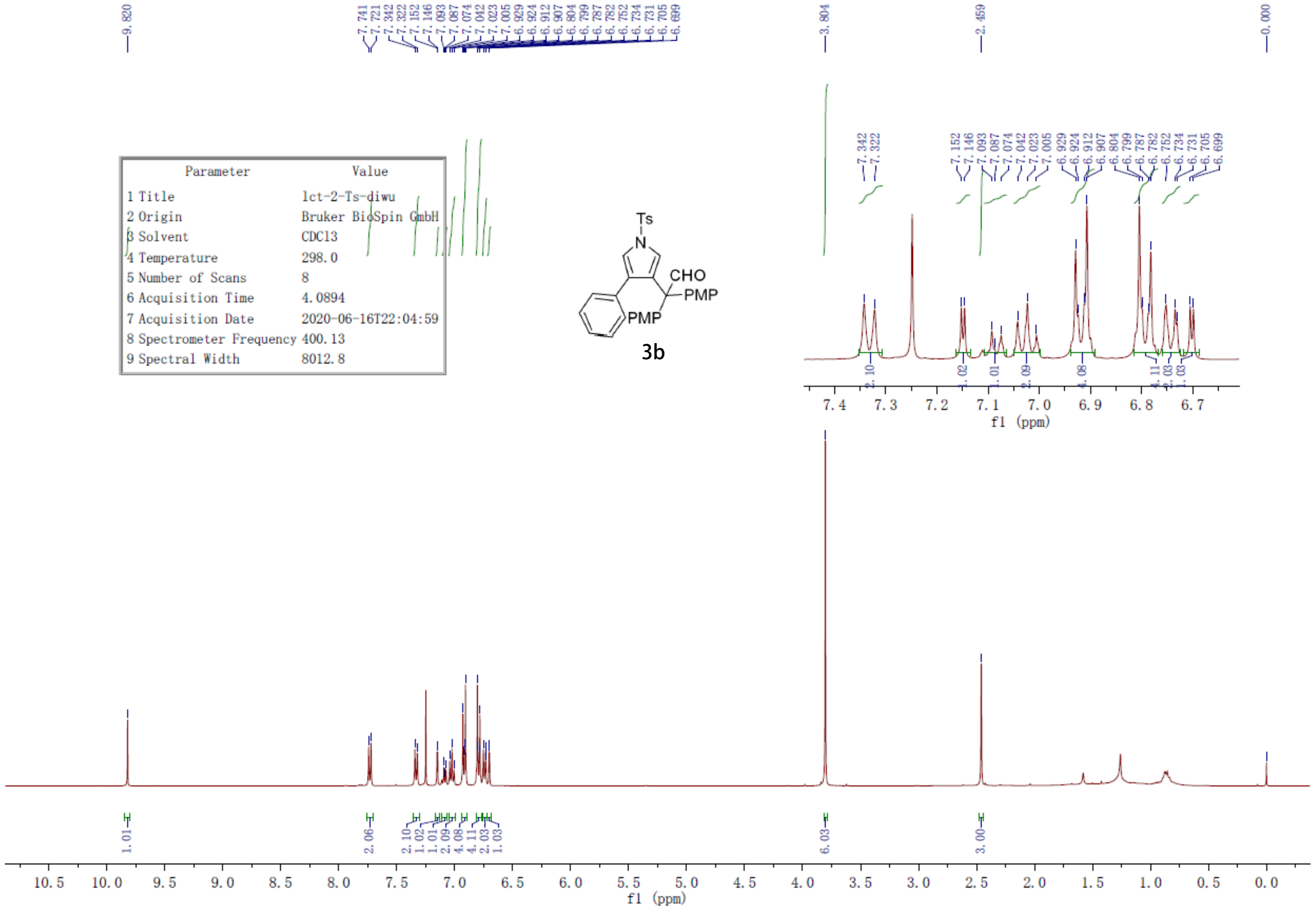

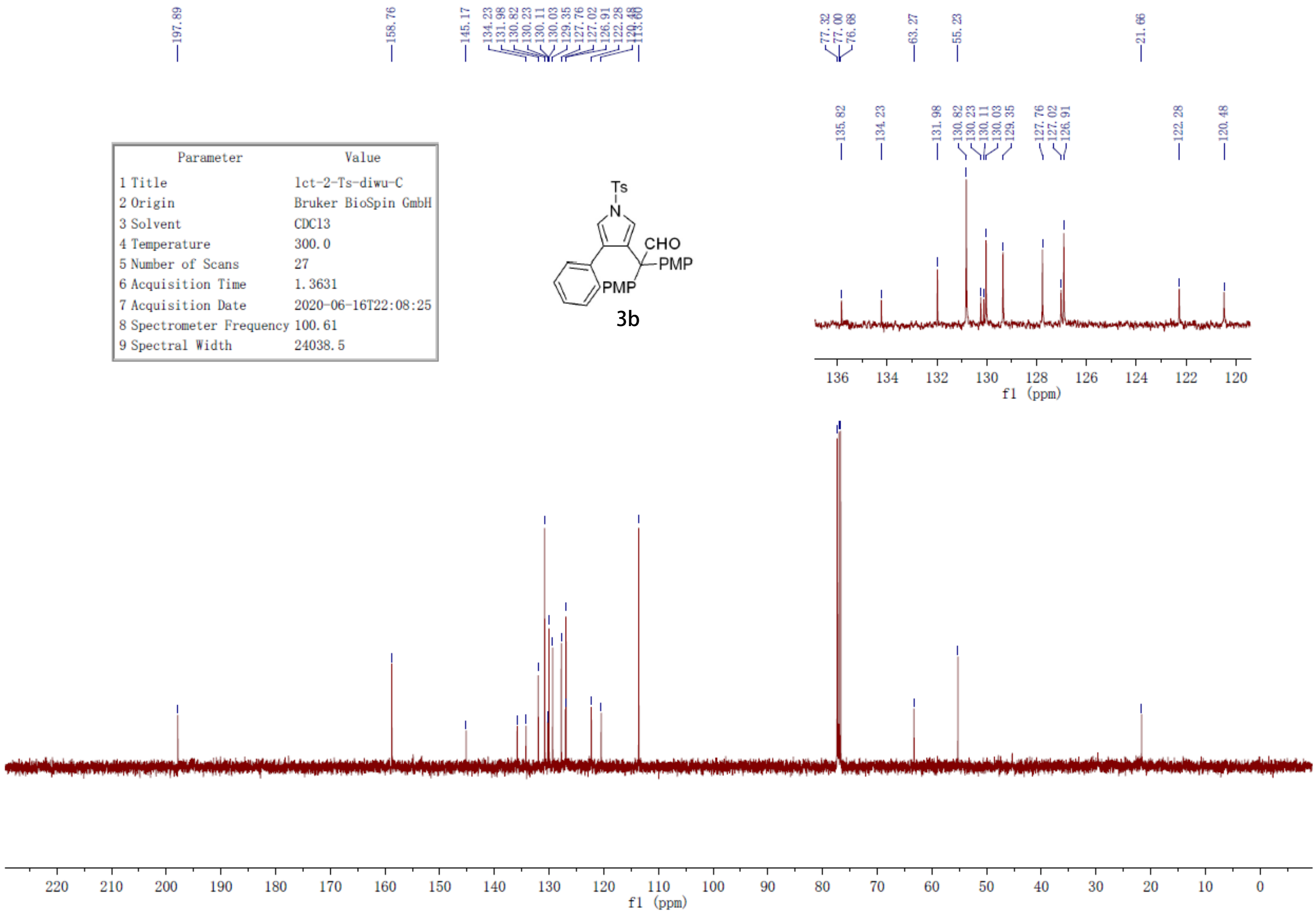

Supplementary Figure 42. ^{1}H and ^{13}C NMR spectra for **3b**

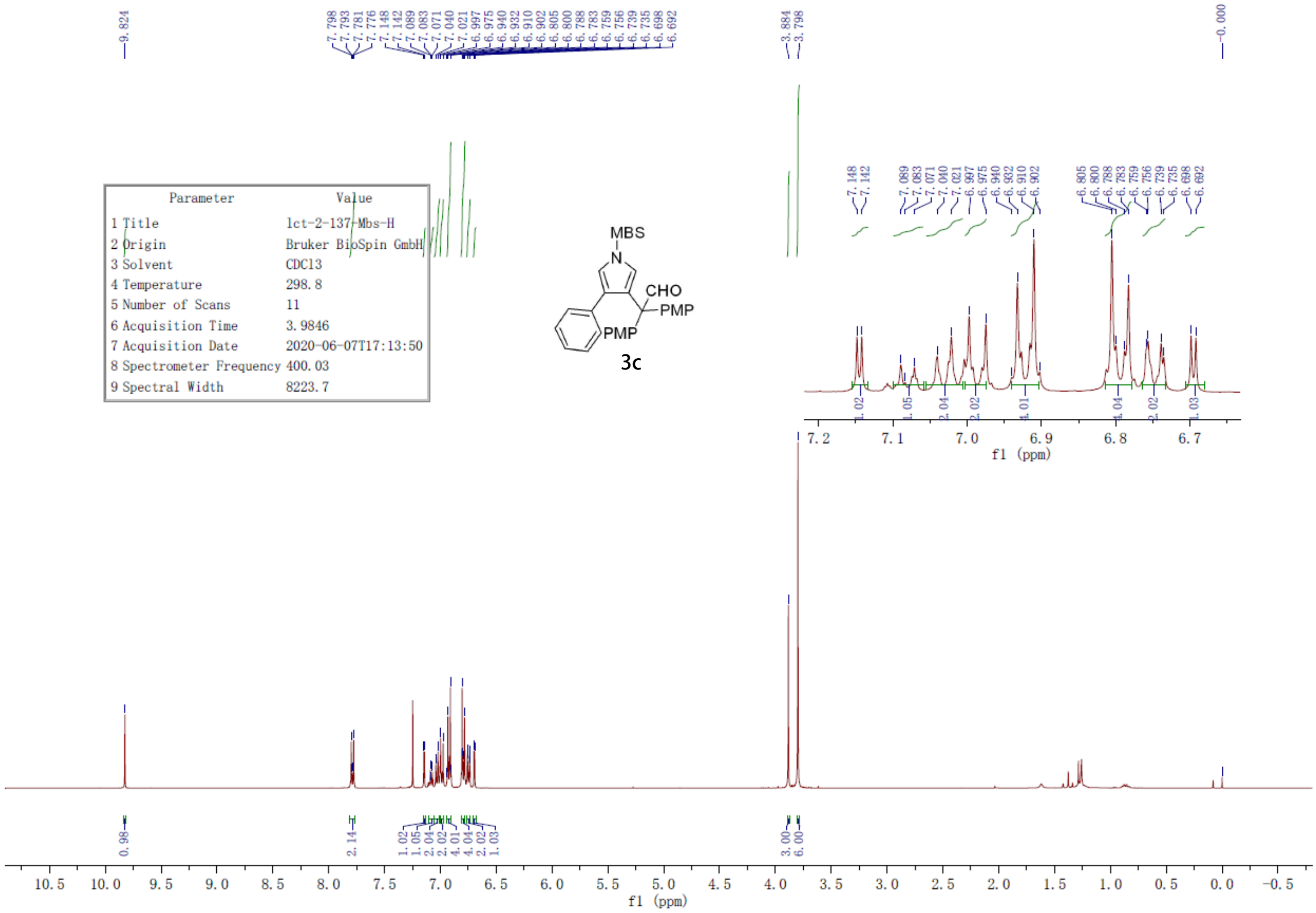

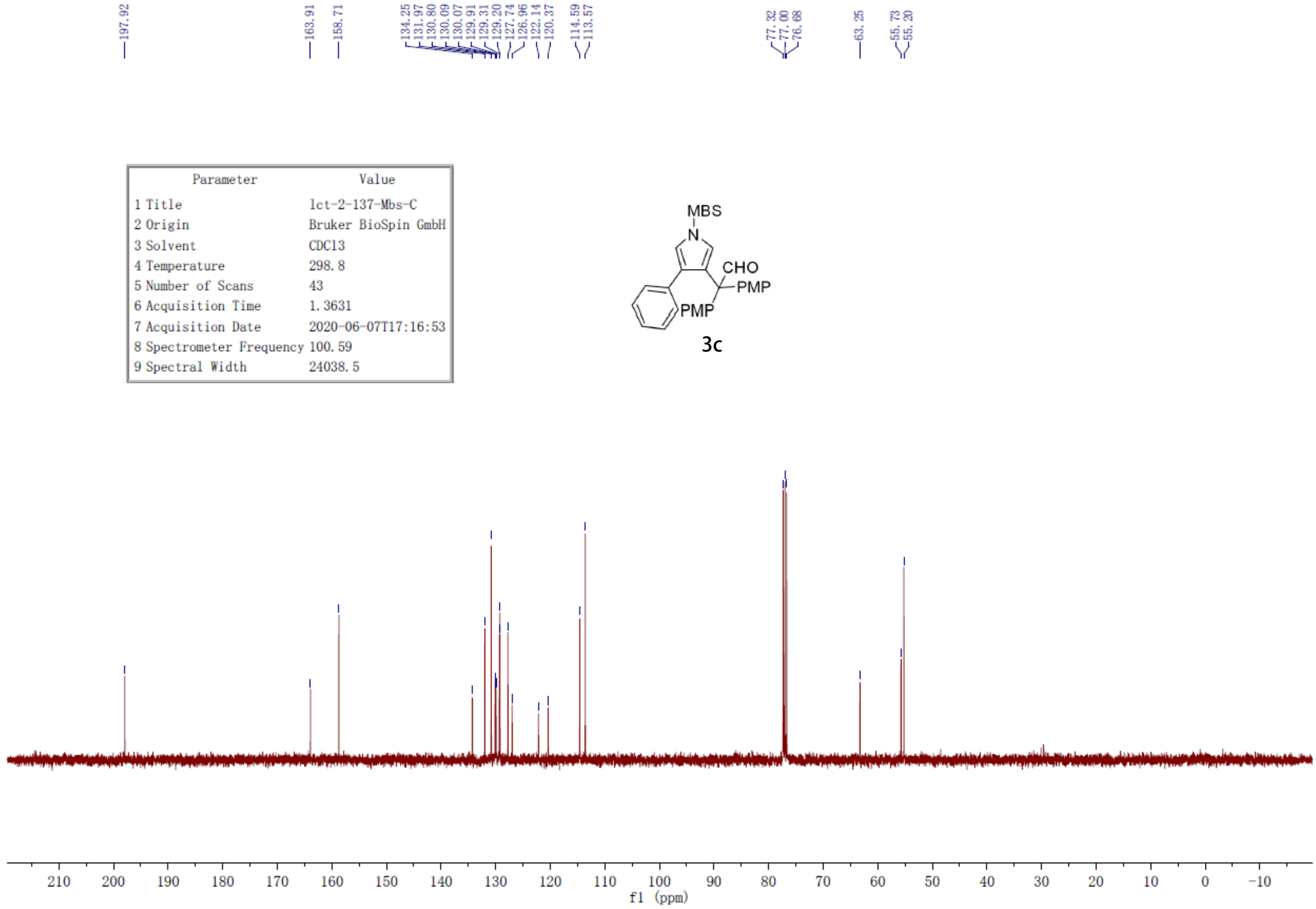

Supplementary Figure 43. ^{1}H and ^{13}C NMR spectra for **3c**

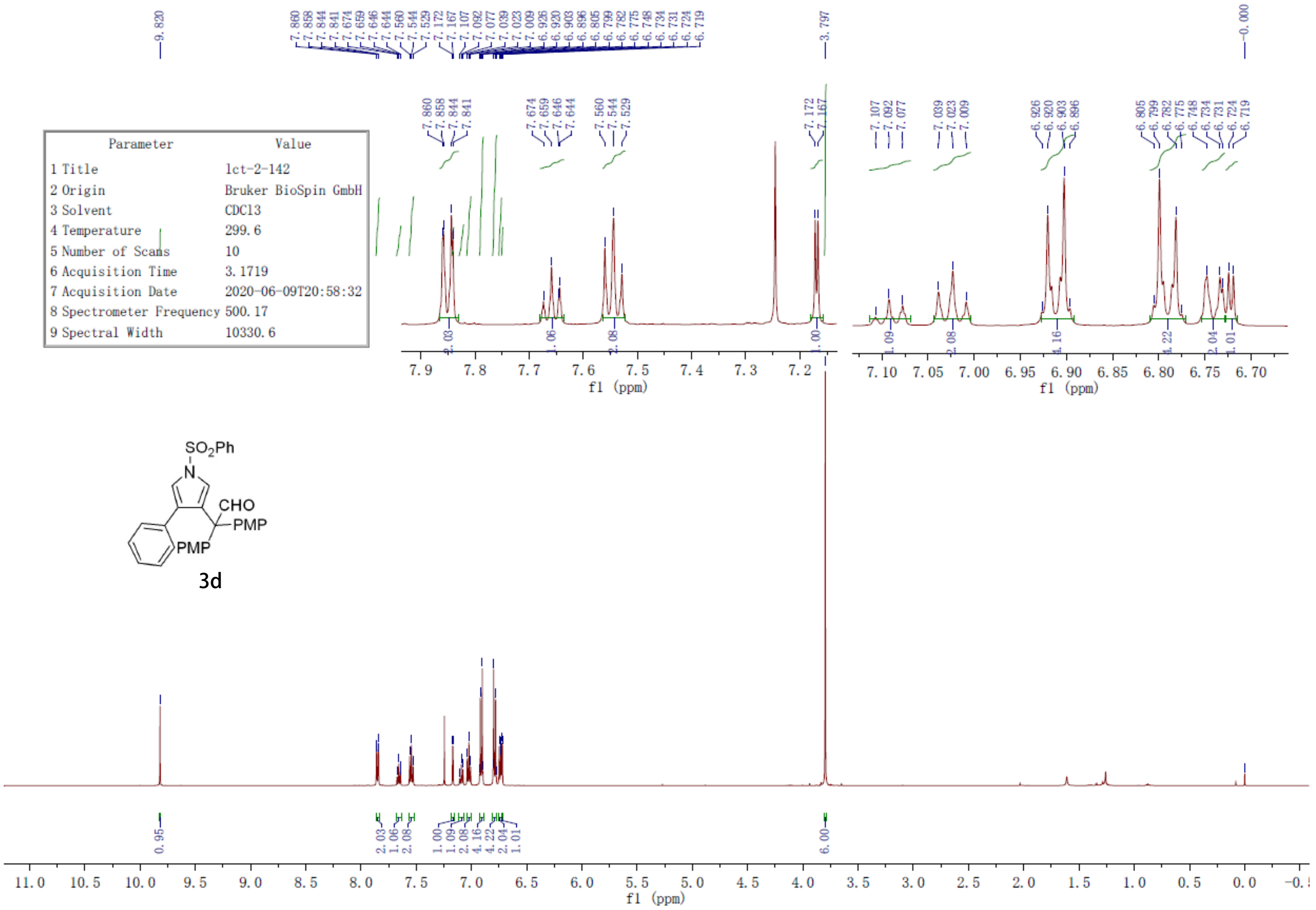

Parameter	Value
1 Title	lct-2-142
2 Origin	Bruker BioSpin GmbH
3 Solvent	CDCl3
4 Temperature	299.6
5 Number of Scans	10
6 Acquisition Time	3.1719
7 Acquisition Date	2020-06-09T20:58:32
8 Spectrometer Frequency	500.17
9 Spectral Width	10330.6

3d

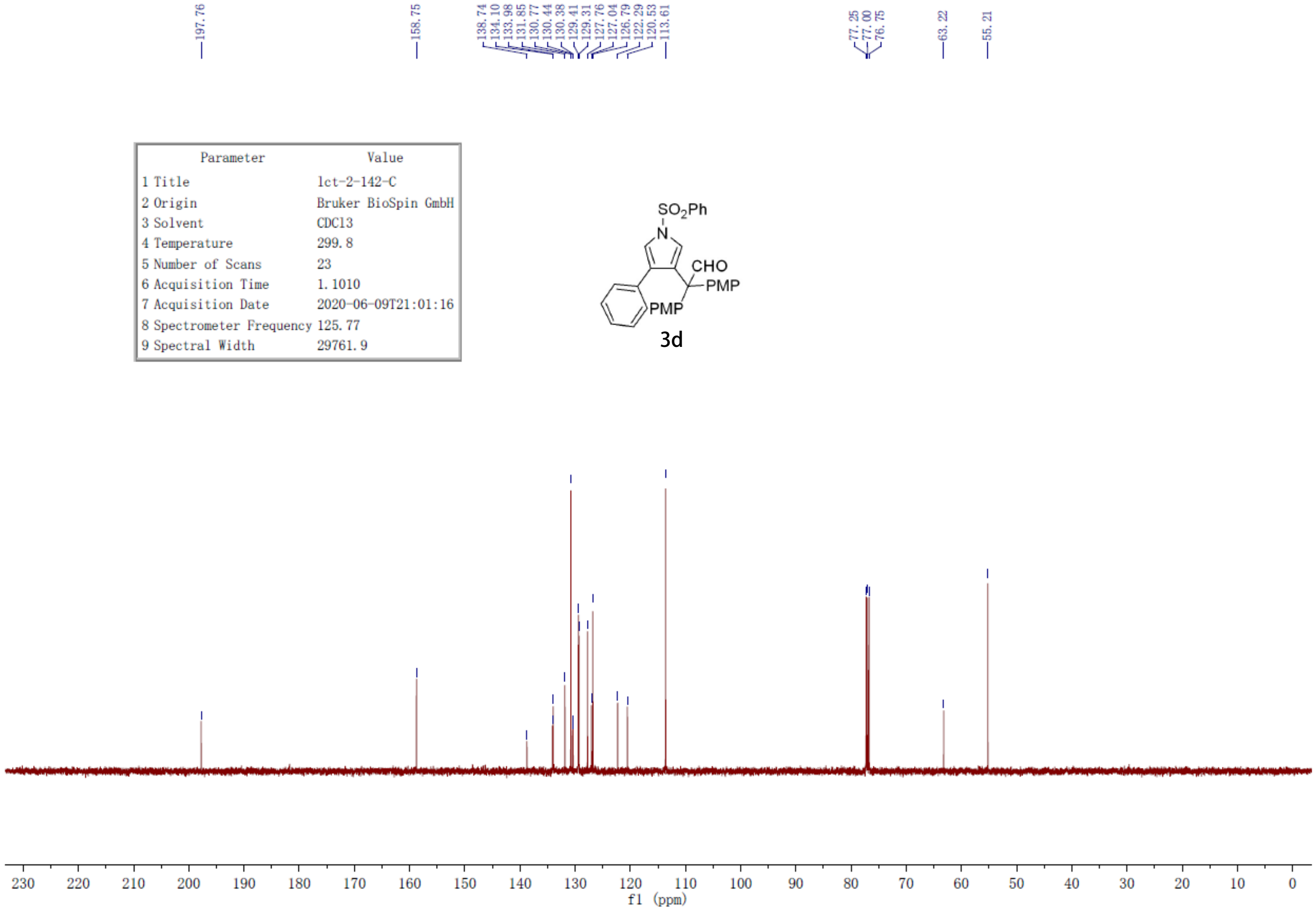

Parameter	Value
1 Title	lct-2-142-C
2 Origin	Bruker BioSpin GmbH
3 Solvent	CDCl3
4 Temperature	299.8
5 Number of Scans	23
6 Acquisition Time	1.1010
7 Acquisition Date	2020-06-09T21:01:16
8 Spectrometer Frequency	125.77
9 Spectral Width	29761.9

3d

Supplementary Figure 44. ^{1}H and ^{13}C NMR spectra for **3d**

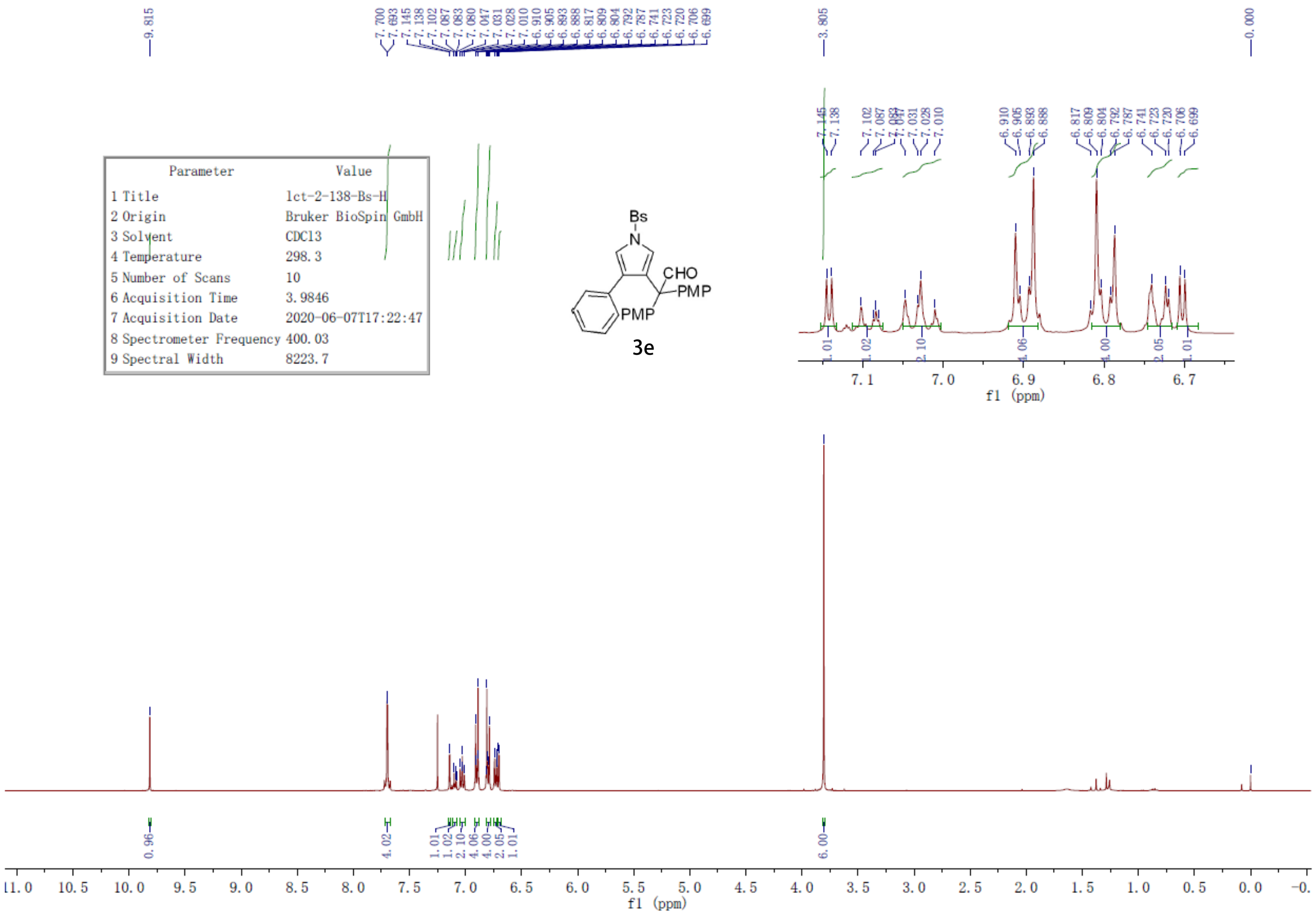

Parameter	Value
1 Title	lct-2-138-Bs-H
2 Origin	Bruker BioSpin GmbH
3 Solvent	CDCl3
4 Temperature	298.3
5 Number of Scans	10
6 Acquisition Time	3.9846
7 Acquisition Date	2020-06-07T17:22:47
8 Spectrometer Frequency	400.03
9 Spectral Width	8223.7

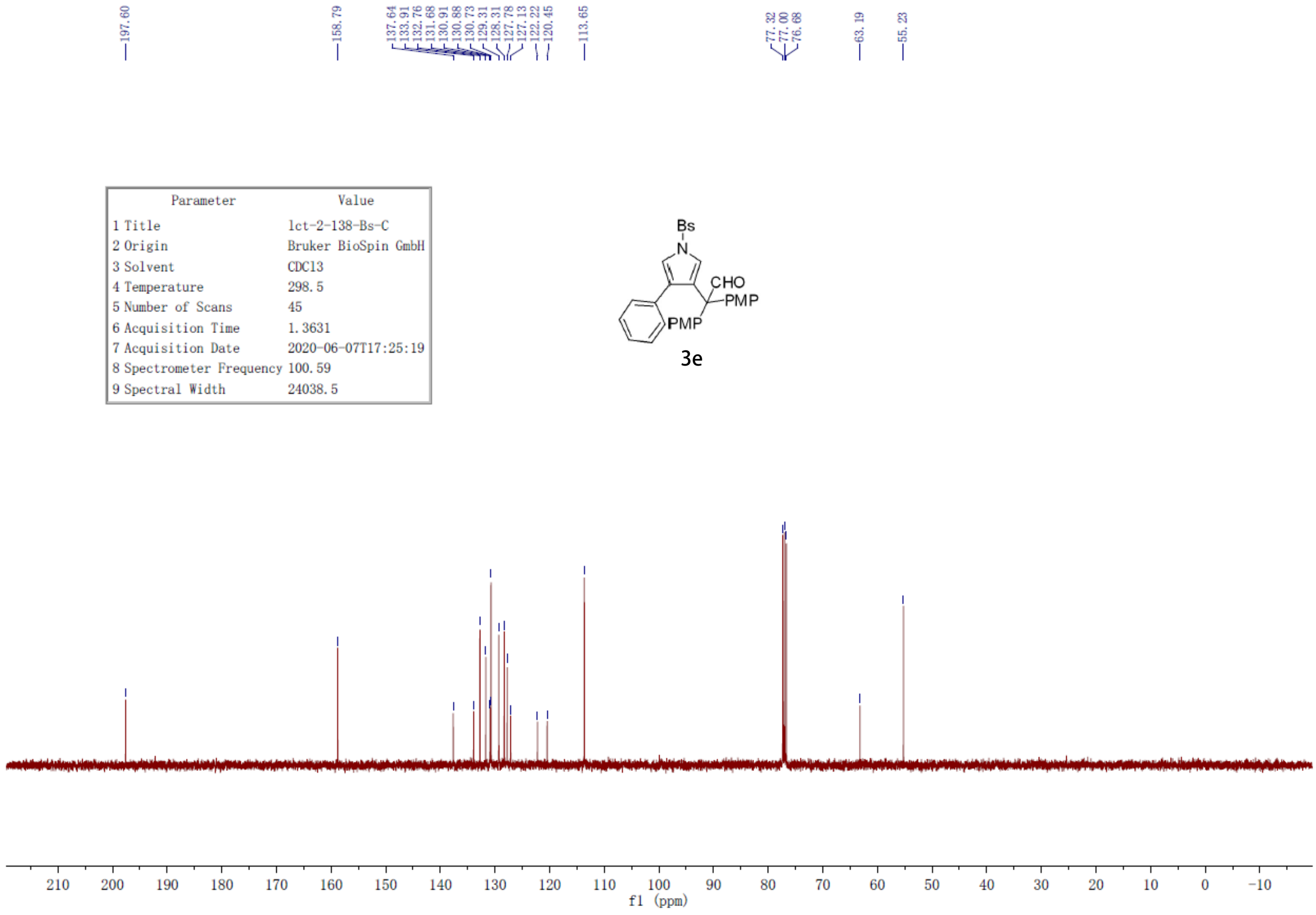

Parameter	Value
1 Title	lct-2-138-Bs-C
2 Origin	Bruker BioSpin GmbH
3 Solvent	CDCl3
4 Temperature	298.5
5 Number of Scans	45
6 Acquisition Time	1.3631
7 Acquisition Date	2020-06-07T17:25:19
8 Spectrometer Frequency	100.59
9 Spectral Width	24038.5

Supplementary Figure 45. ^{1}H and ^{13}C NMR spectra for **3e**

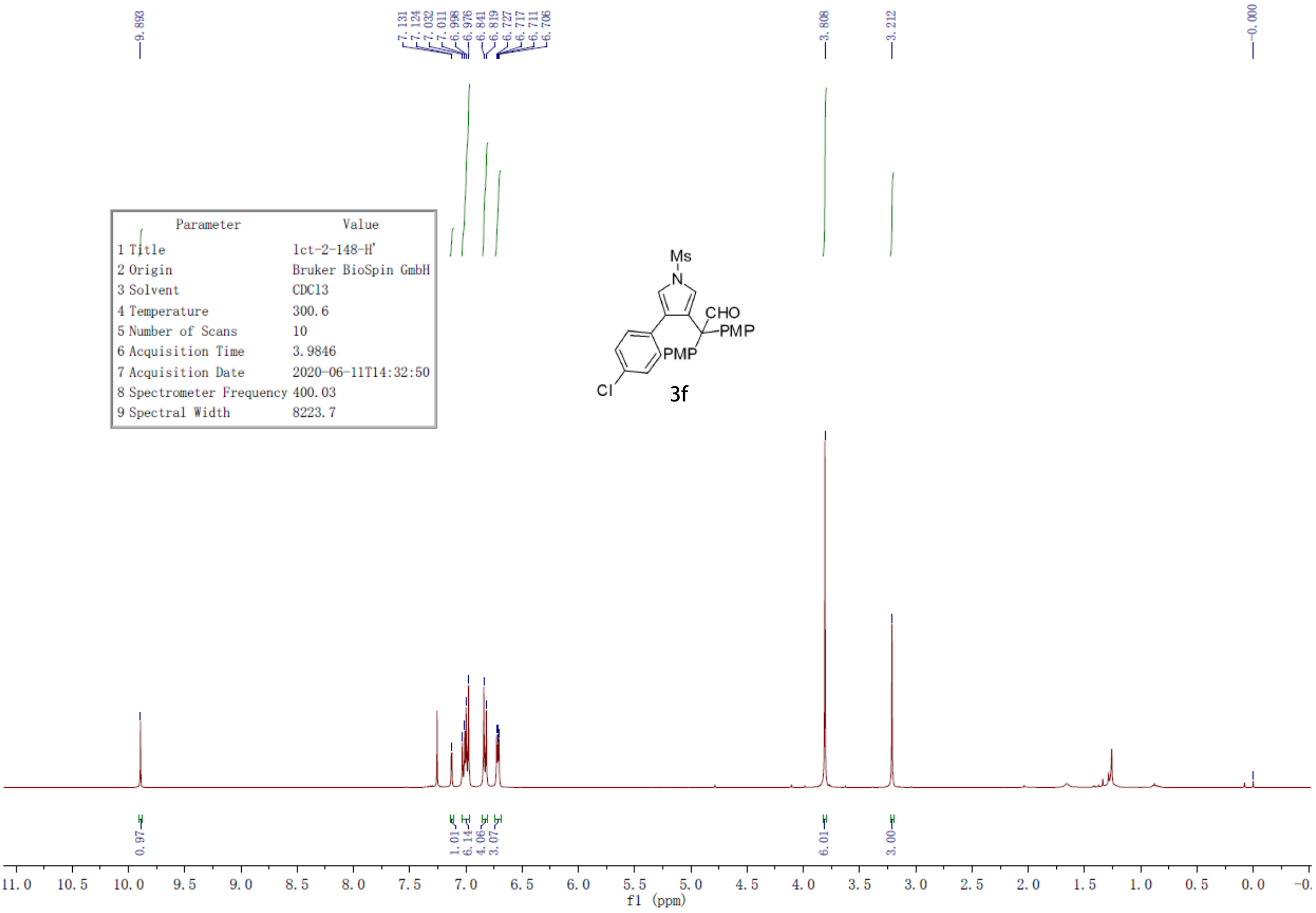

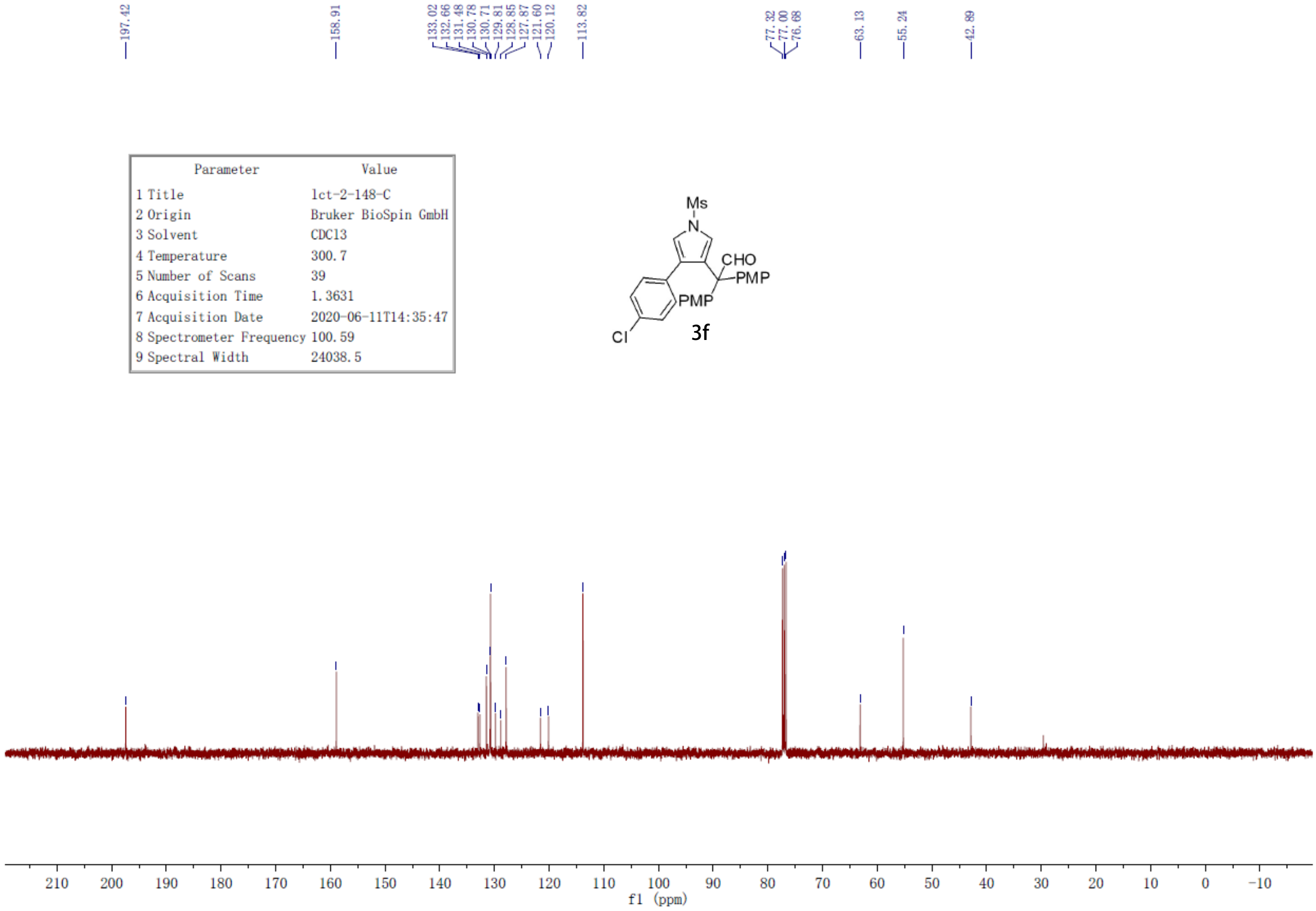

Supplementary Figure 46. ^{1}H and ^{13}C NMR spectra for **3f**

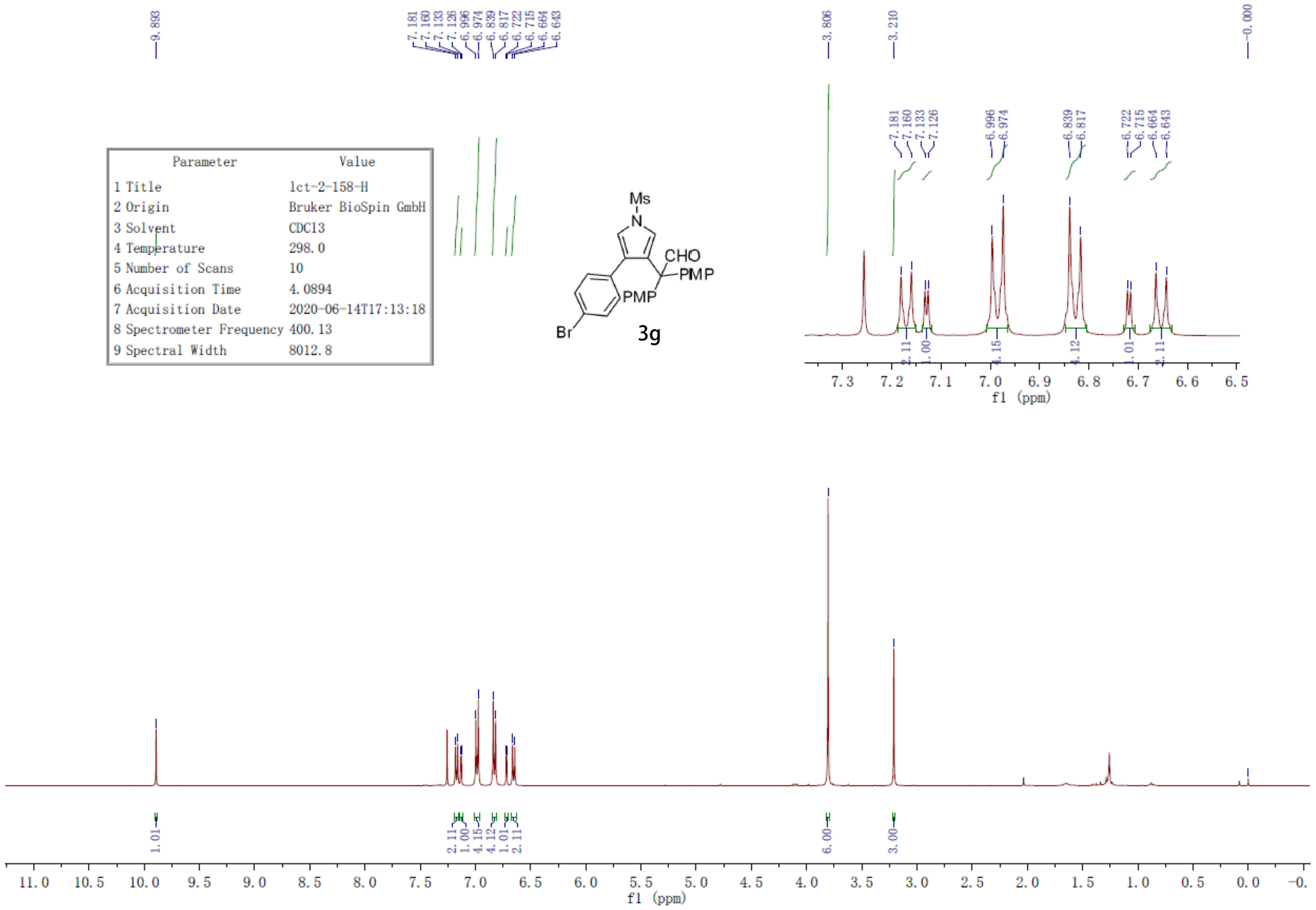

Parameter	Value
1 Title	lct-2-158-H
2 Origin	Bruker BioSpin GmbH
3 Solvent	CDCl3
4 Temperature	298.0
5 Number of Scans	10
6 Acquisition Time	4.0894
7 Acquisition Date	2020-06-14T17:13:18
8 Spectrometer Frequency	400.13
9 Spectral Width	8012.8

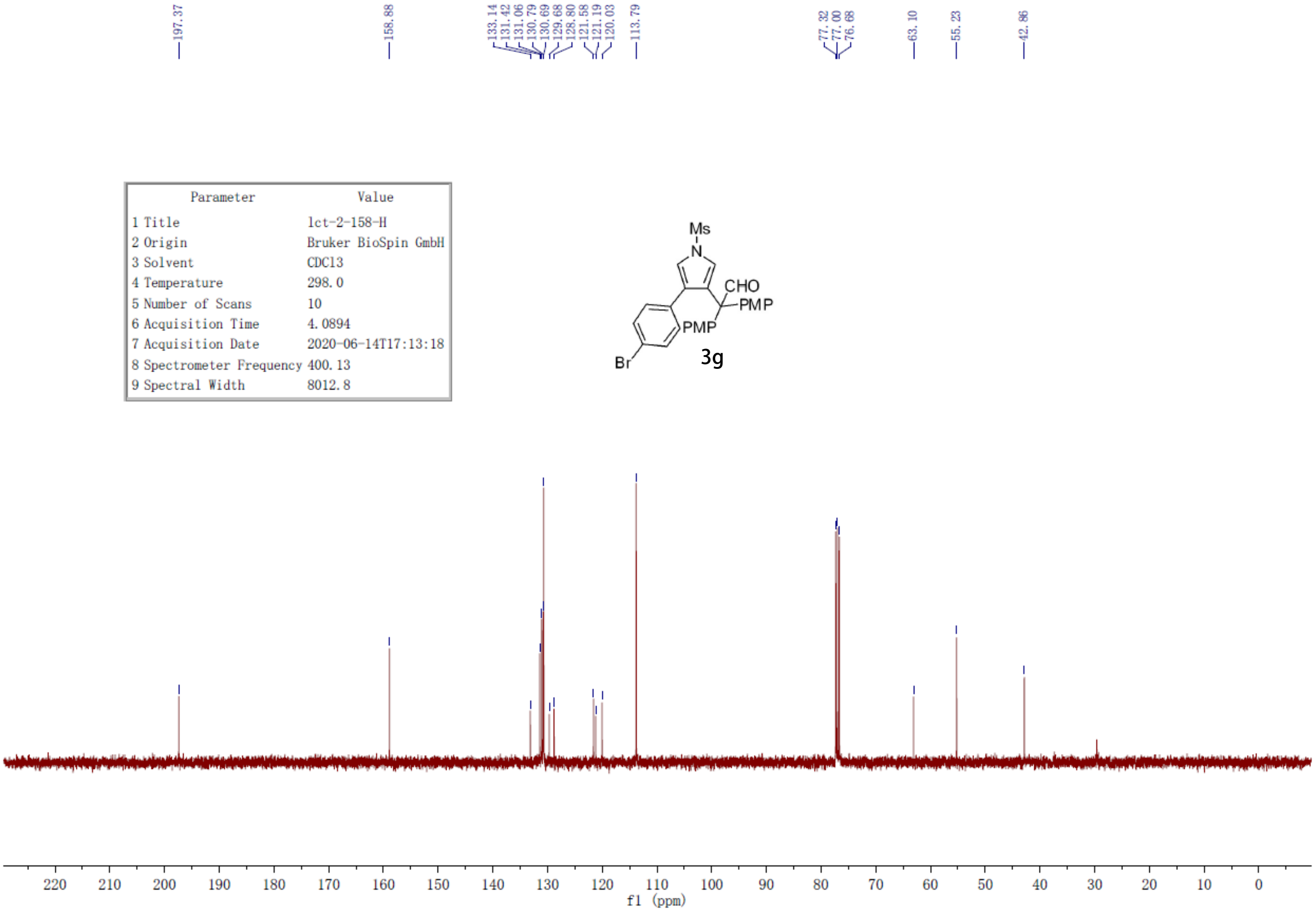

Parameter	Value
1 Title	lct-2-158-H
2 Origin	Bruker BioSpin GmbH
3 Solvent	CDCl3
4 Temperature	298.0
5 Number of Scans	10
6 Acquisition Time	4.0894
7 Acquisition Date	2020-06-14T17:13:18
8 Spectrometer Frequency	400.13
9 Spectral Width	8012.8

Supplementary Figure 47. ^{1}H and ^{13}C NMR spectra for **3g**

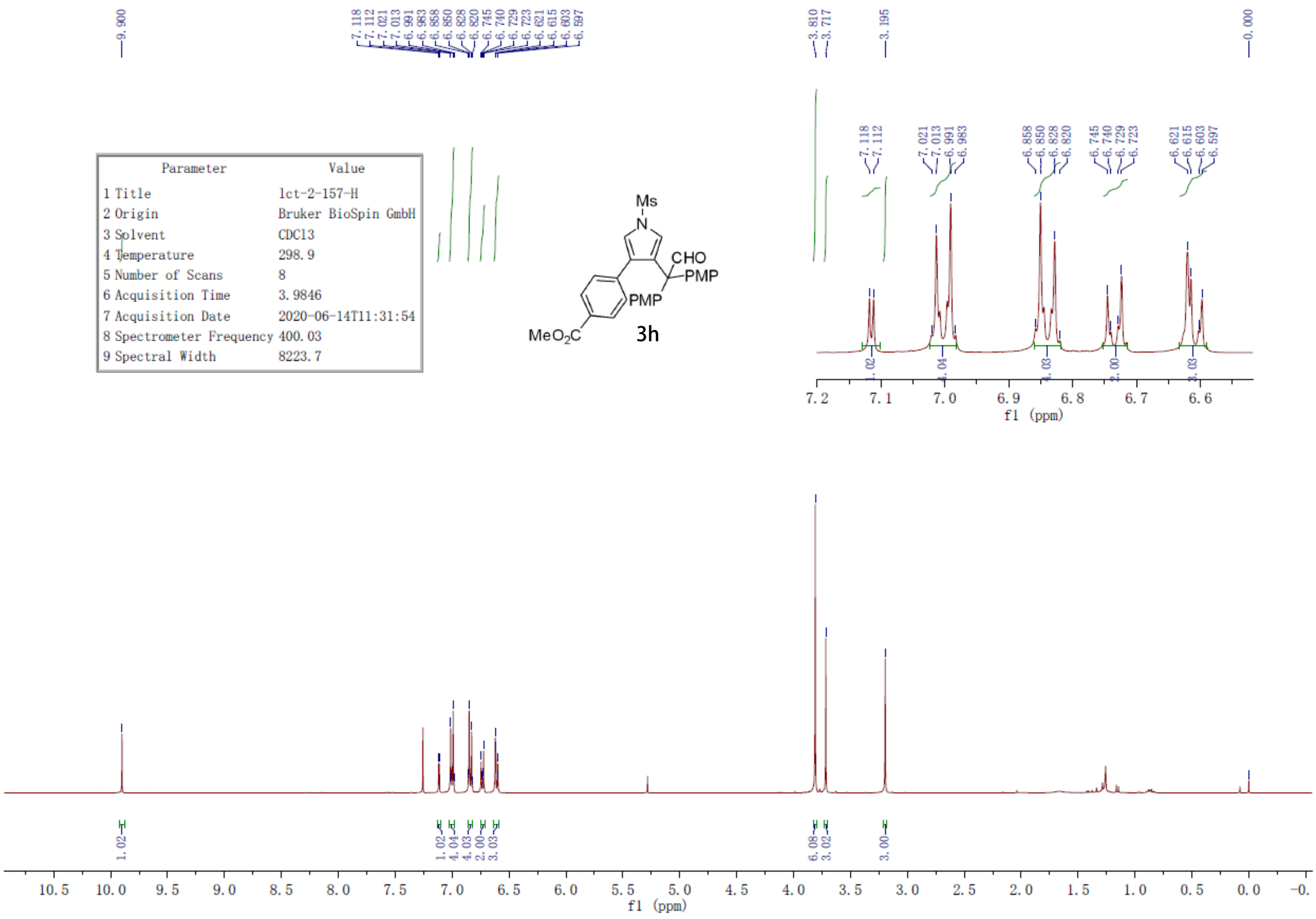

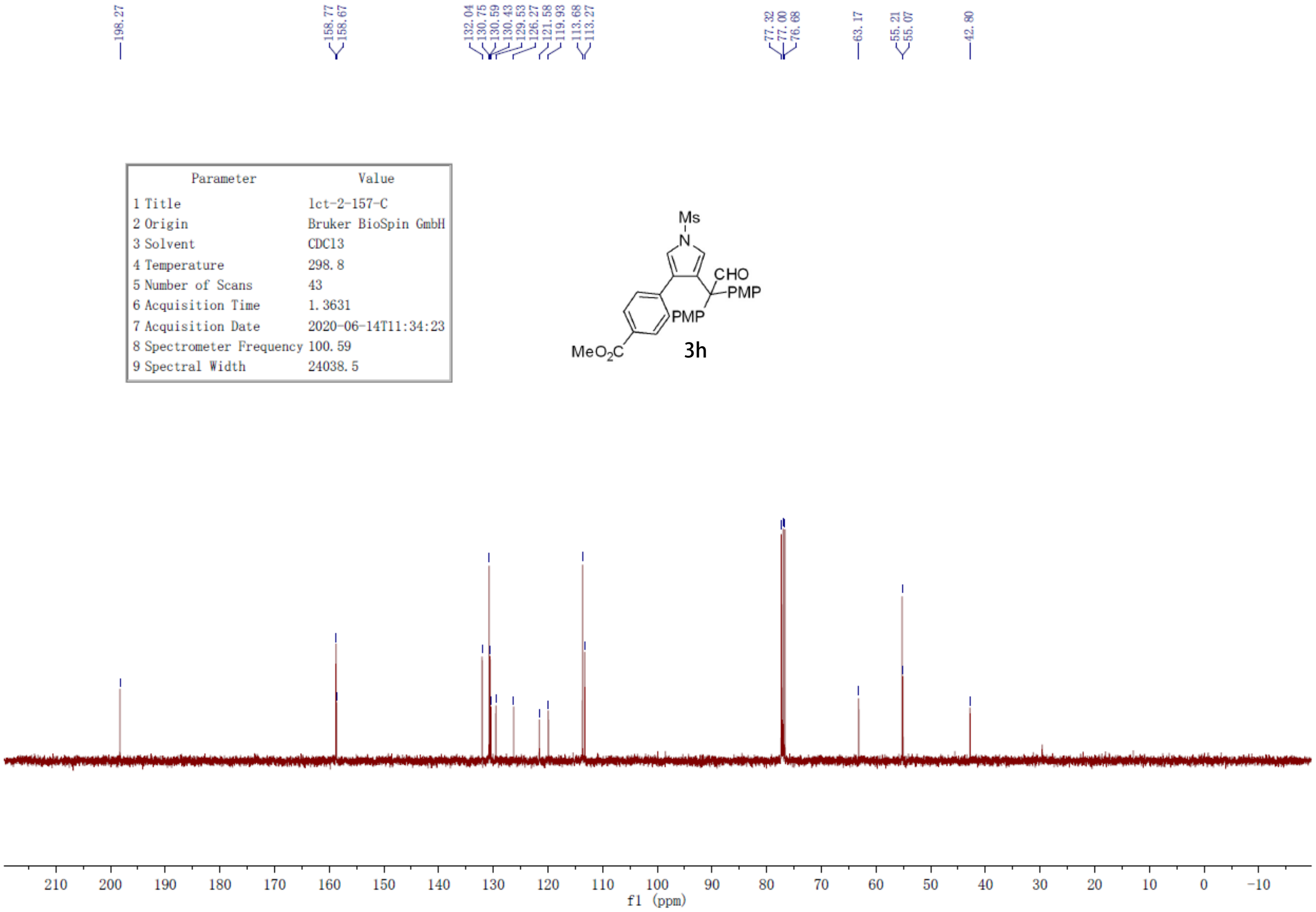

Supplementary Figure 48. ^{1}H and ^{13}C NMR spectra for **3h**

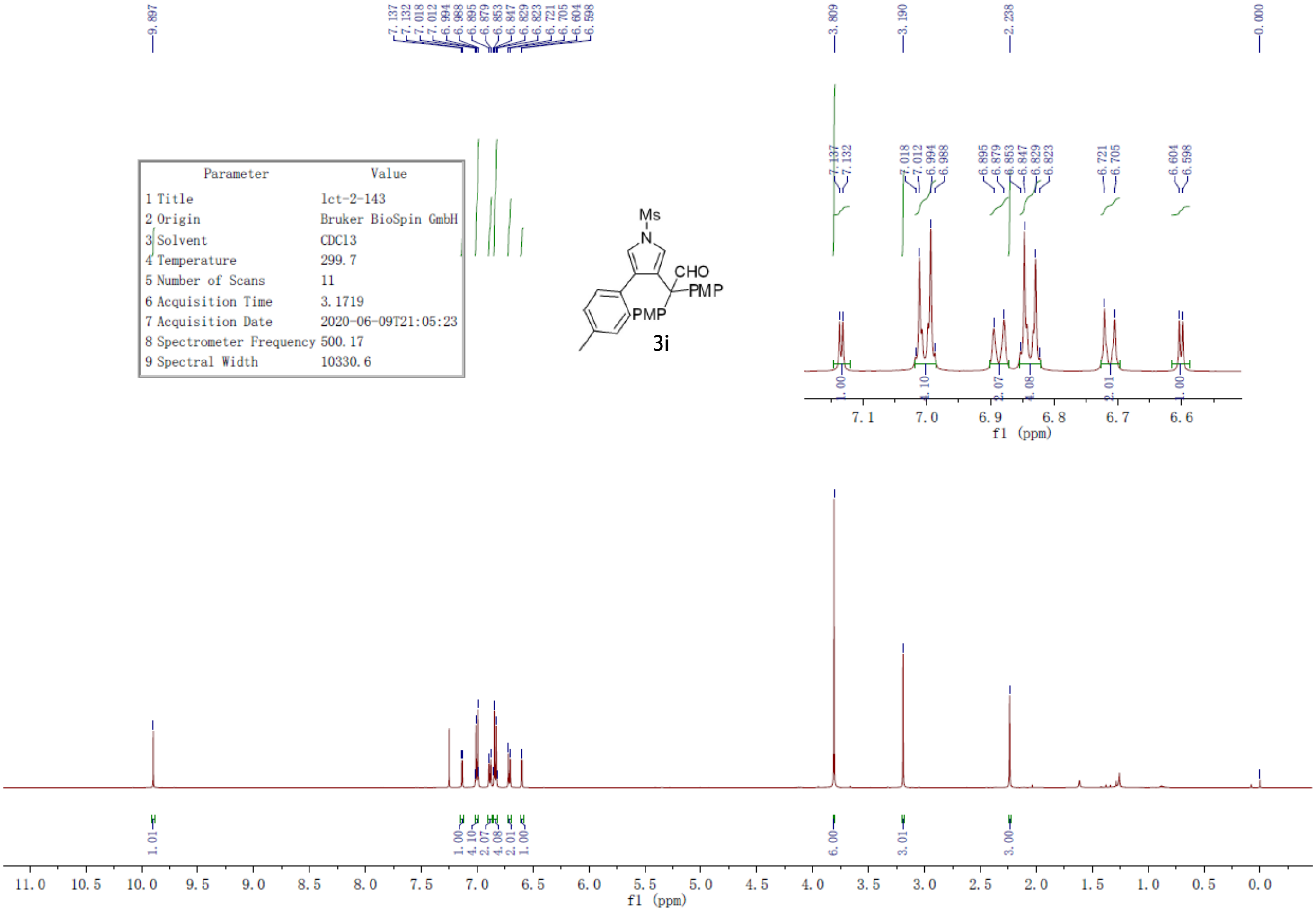

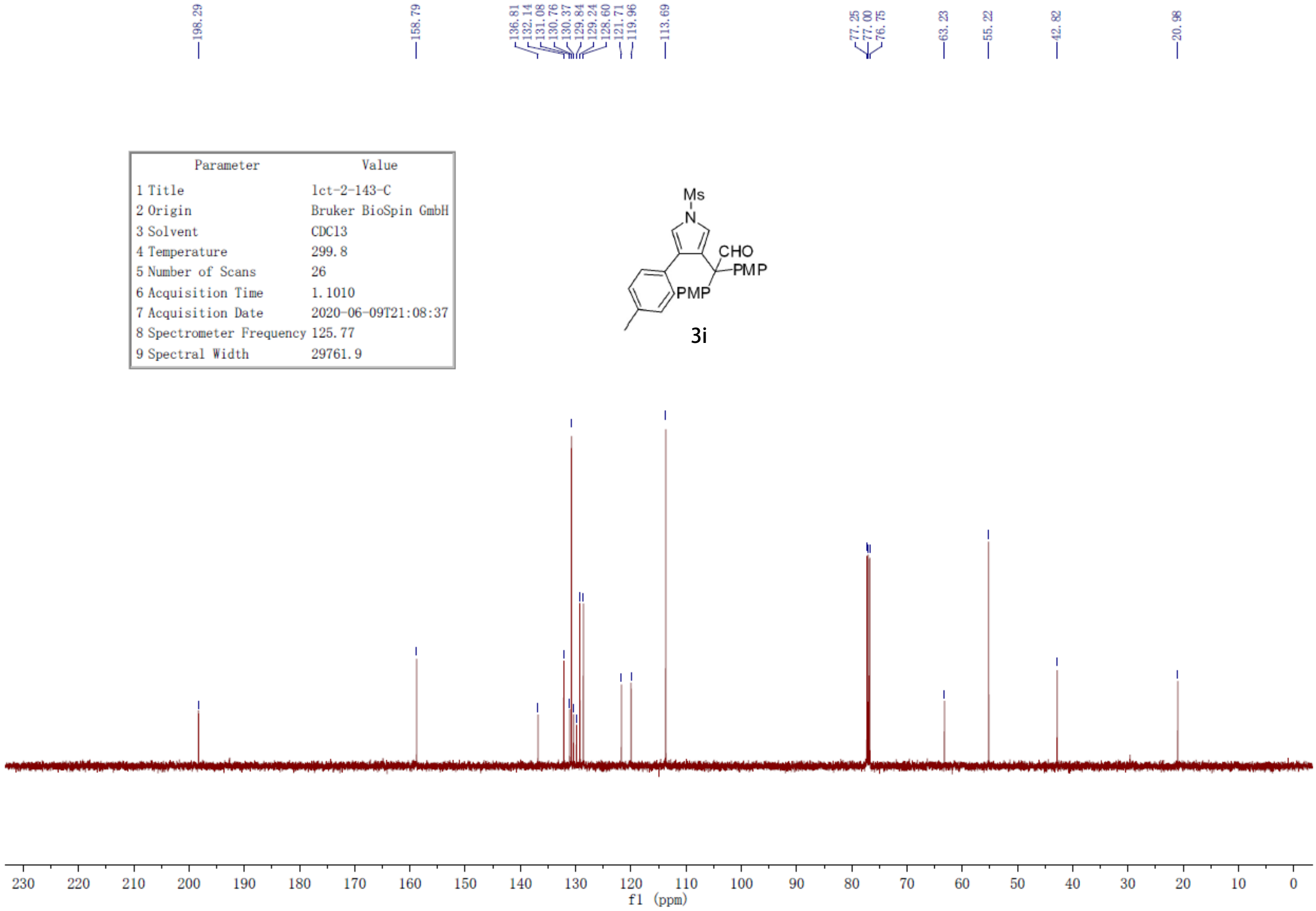

Supplementary Figure 49. ^{1}H and ^{13}C NMR spectra for **3i**

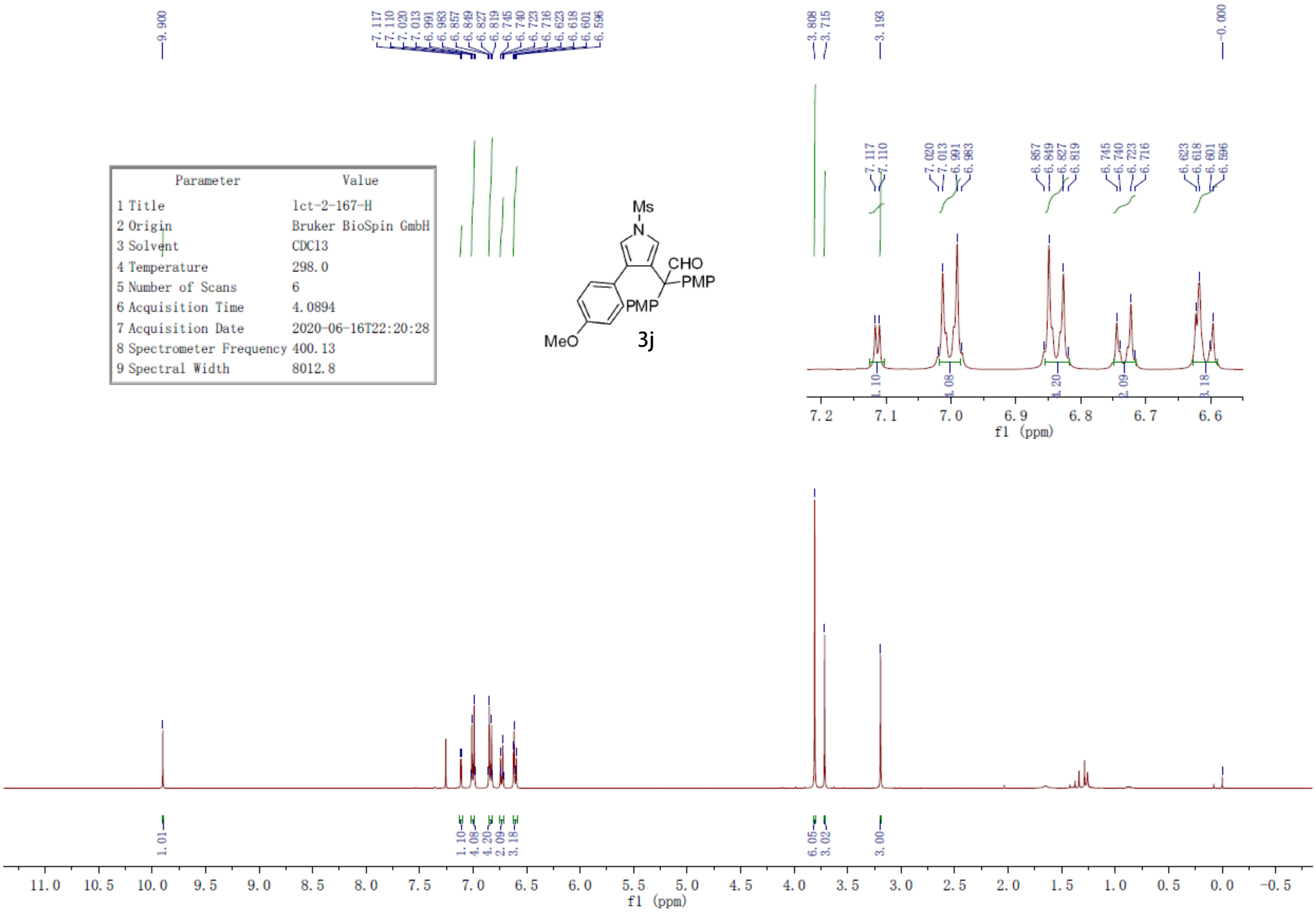

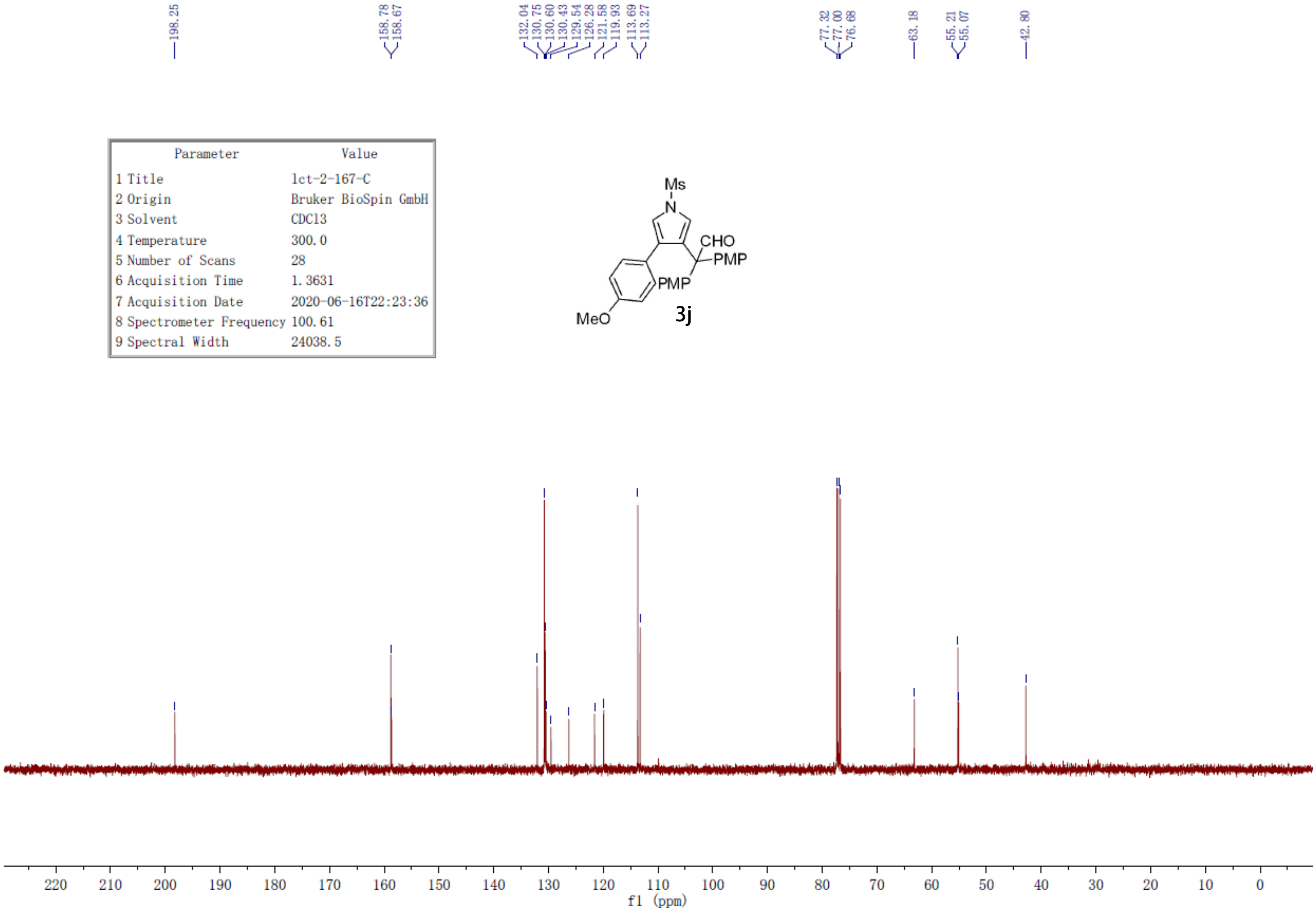

Supplementary Figure 50. ^{1}H and ^{13}C NMR spectra for **3j**

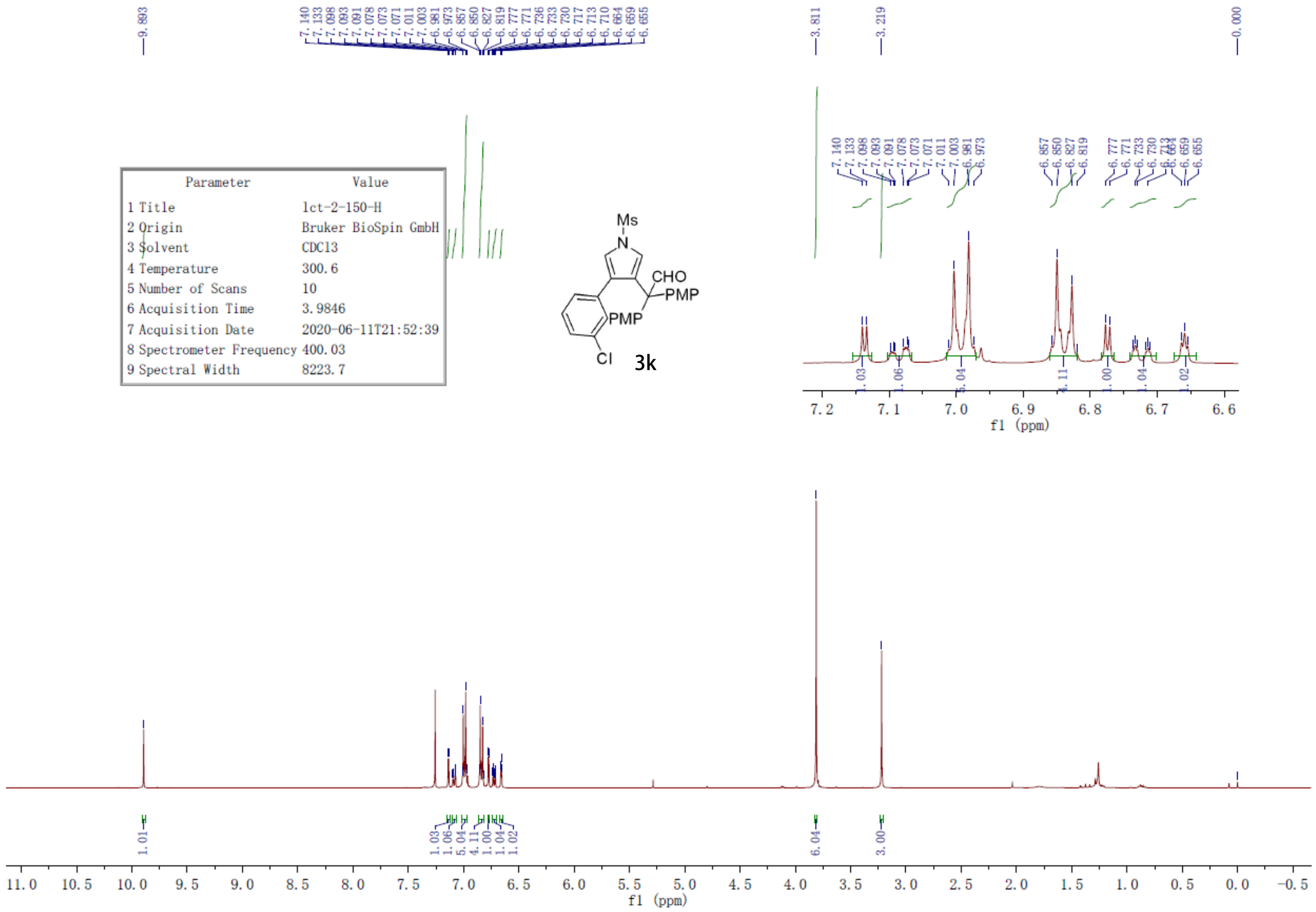

Parameter	Value
1 Title	lct-2-150-H
2 Origin	Bruker BioSpin GmbH
3 Solvent	CDCl3
4 Temperature	300.6
5 Number of Scans	10
6 Acquisition Time	3.9846
7 Acquisition Date	2020-06-11T21:52:39
8 Spectrometer Frequency	400.03
9 Spectral Width	8223.7

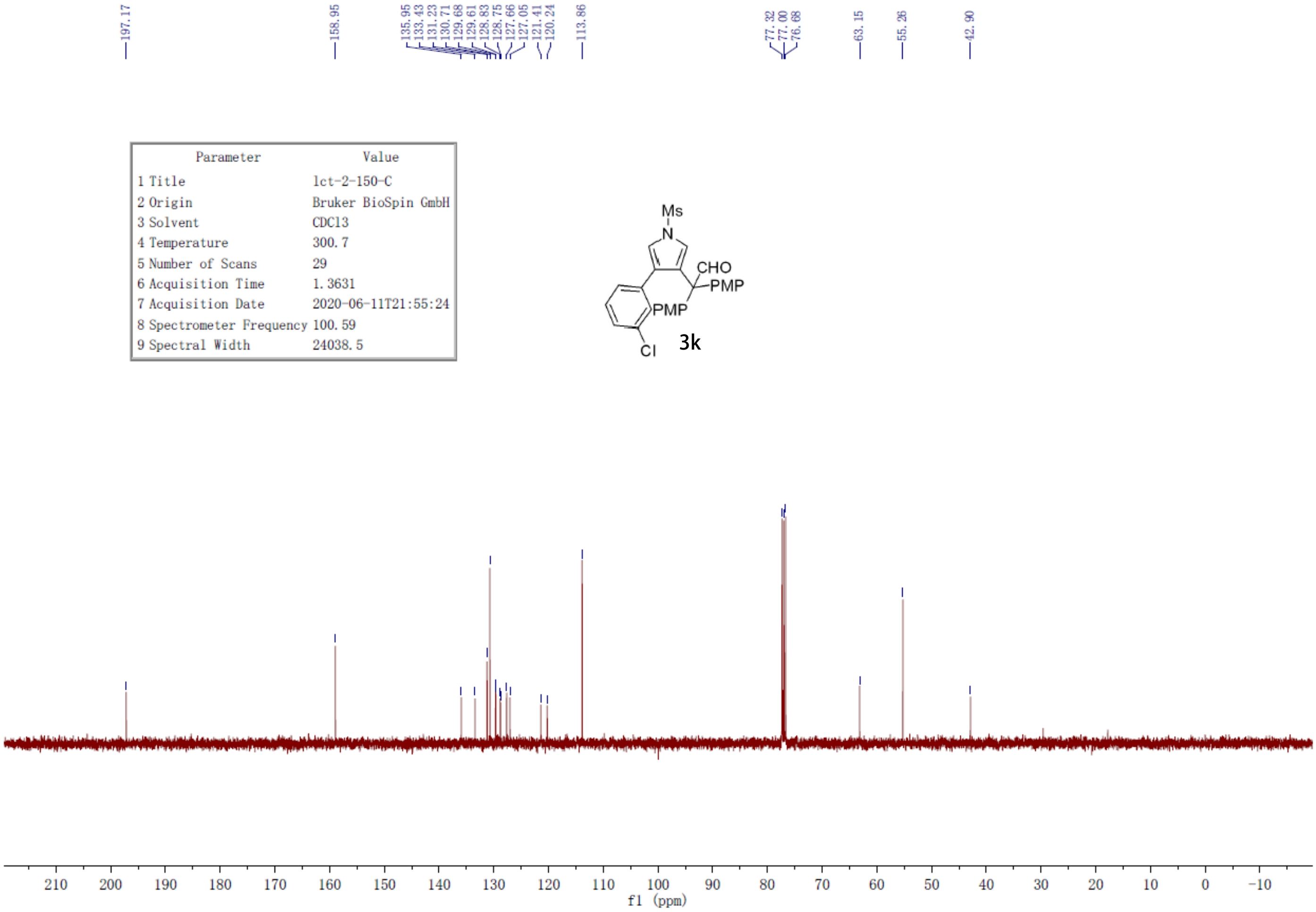

Parameter	Value
1 Title	lct-2-150-C
2 Origin	Bruker BioSpin GmbH
3 Solvent	CDCl3
4 Temperature	300.7
5 Number of Scans	29
6 Acquisition Time	1.3631
7 Acquisition Date	2020-06-11T21:55:24
8 Spectrometer Frequency	100.59
9 Spectral Width	24038.5

Supplementary Figure 51. ^{1}H and ^{13}C NMR spectra for **3k**

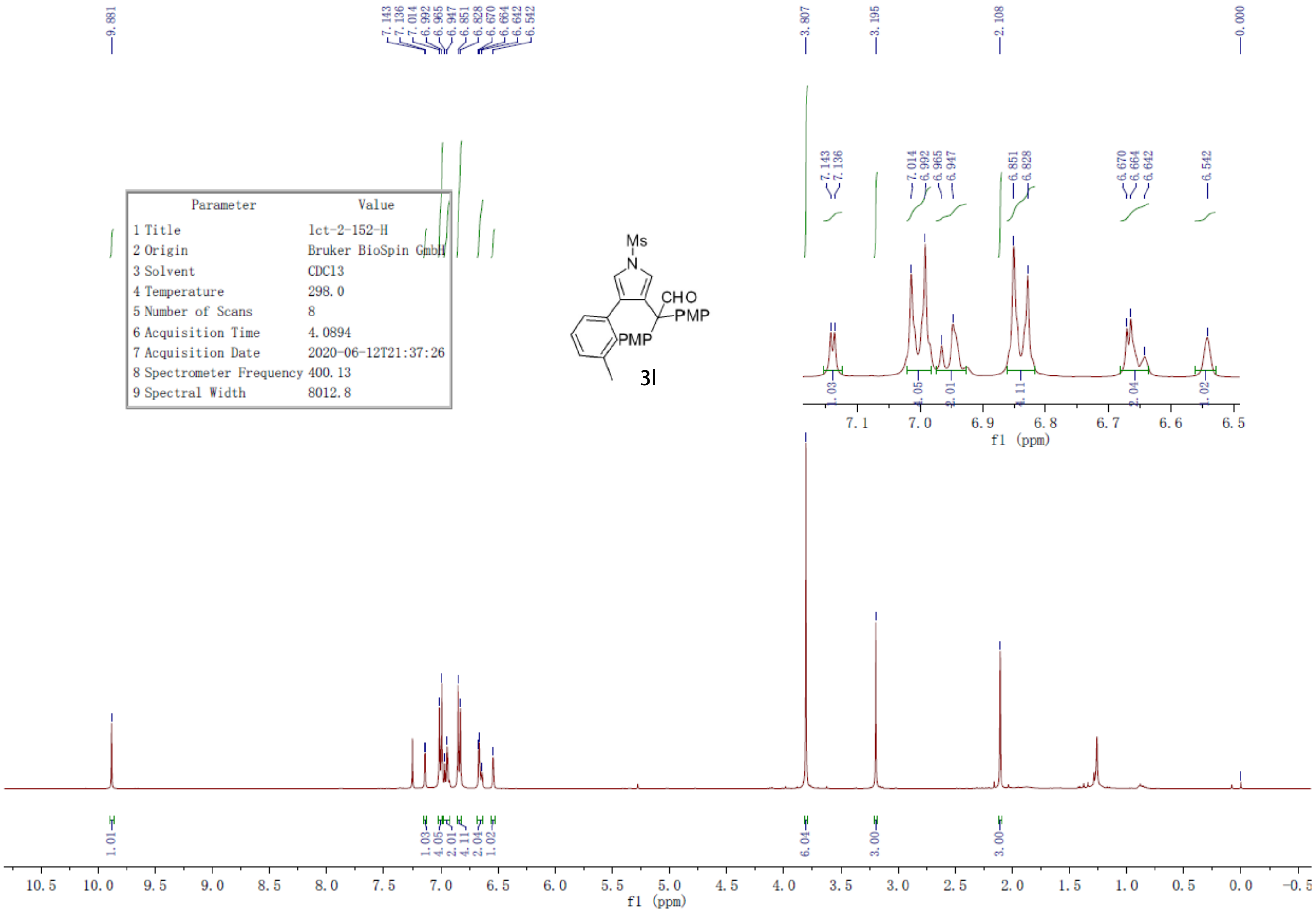

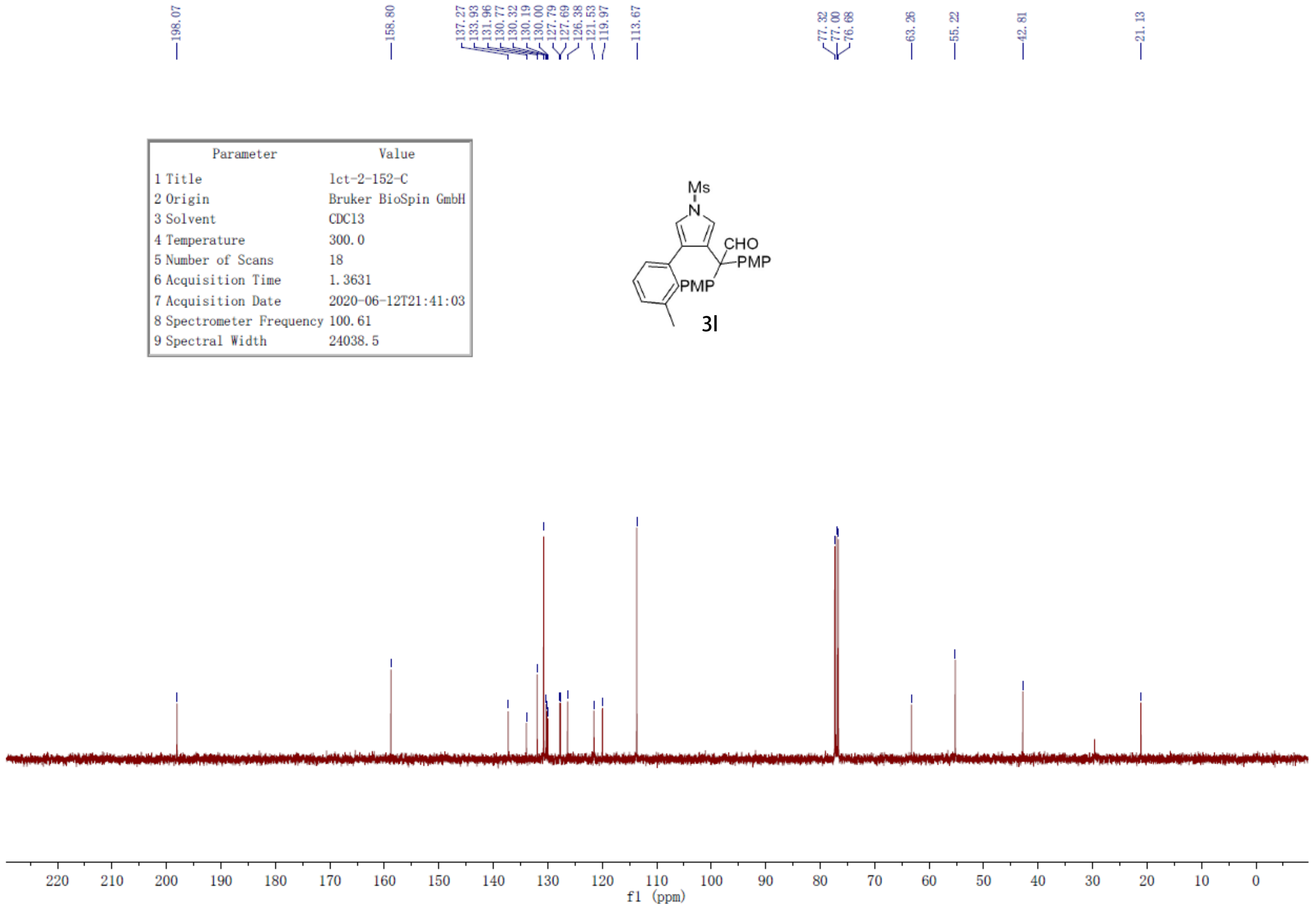

Supplementary Figure 52. ^{1}H and ^{13}C NMR spectra for **3l**

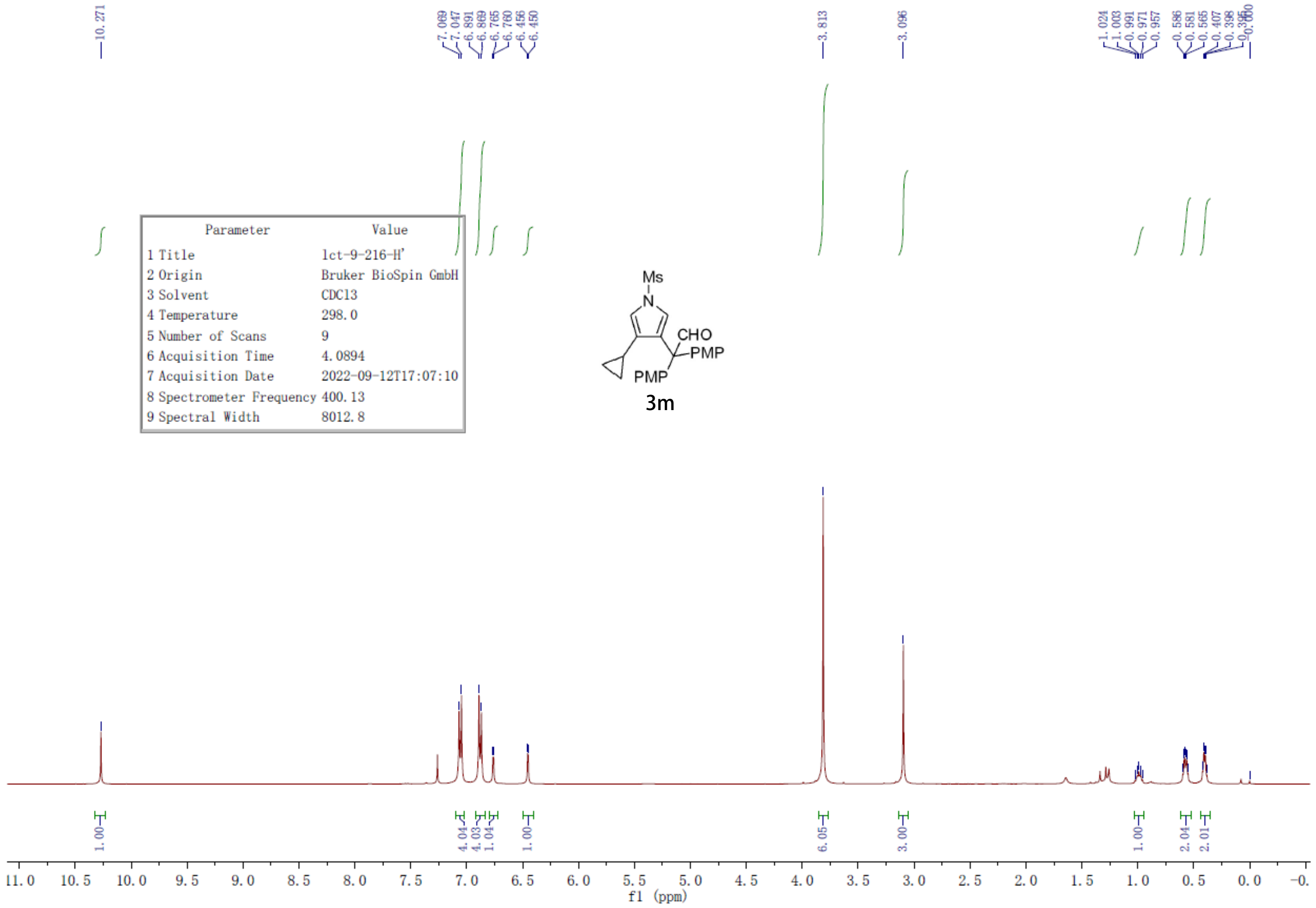

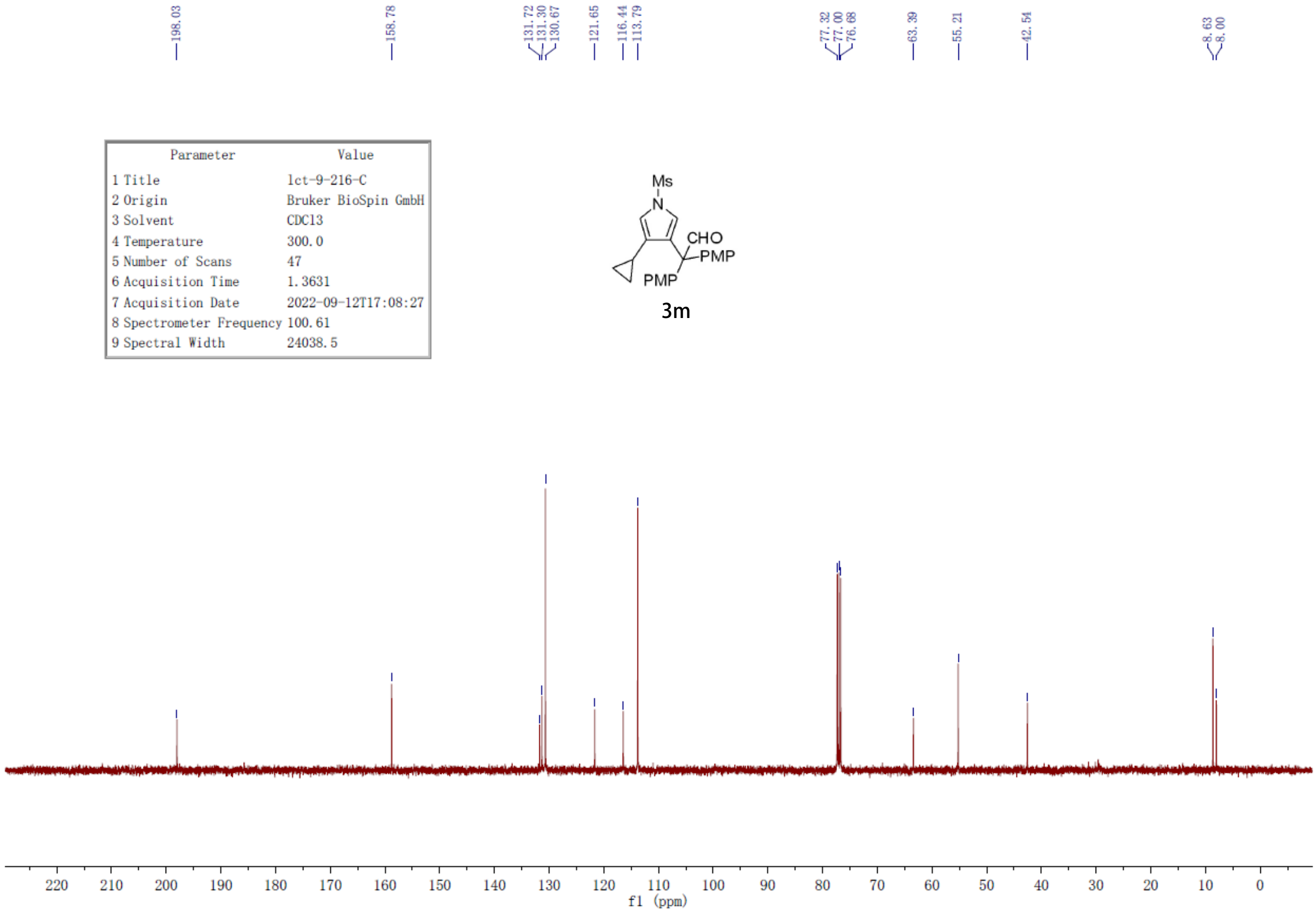

Supplementary Figure 53. ^{1}H and ^{13}C NMR spectra for **3m**

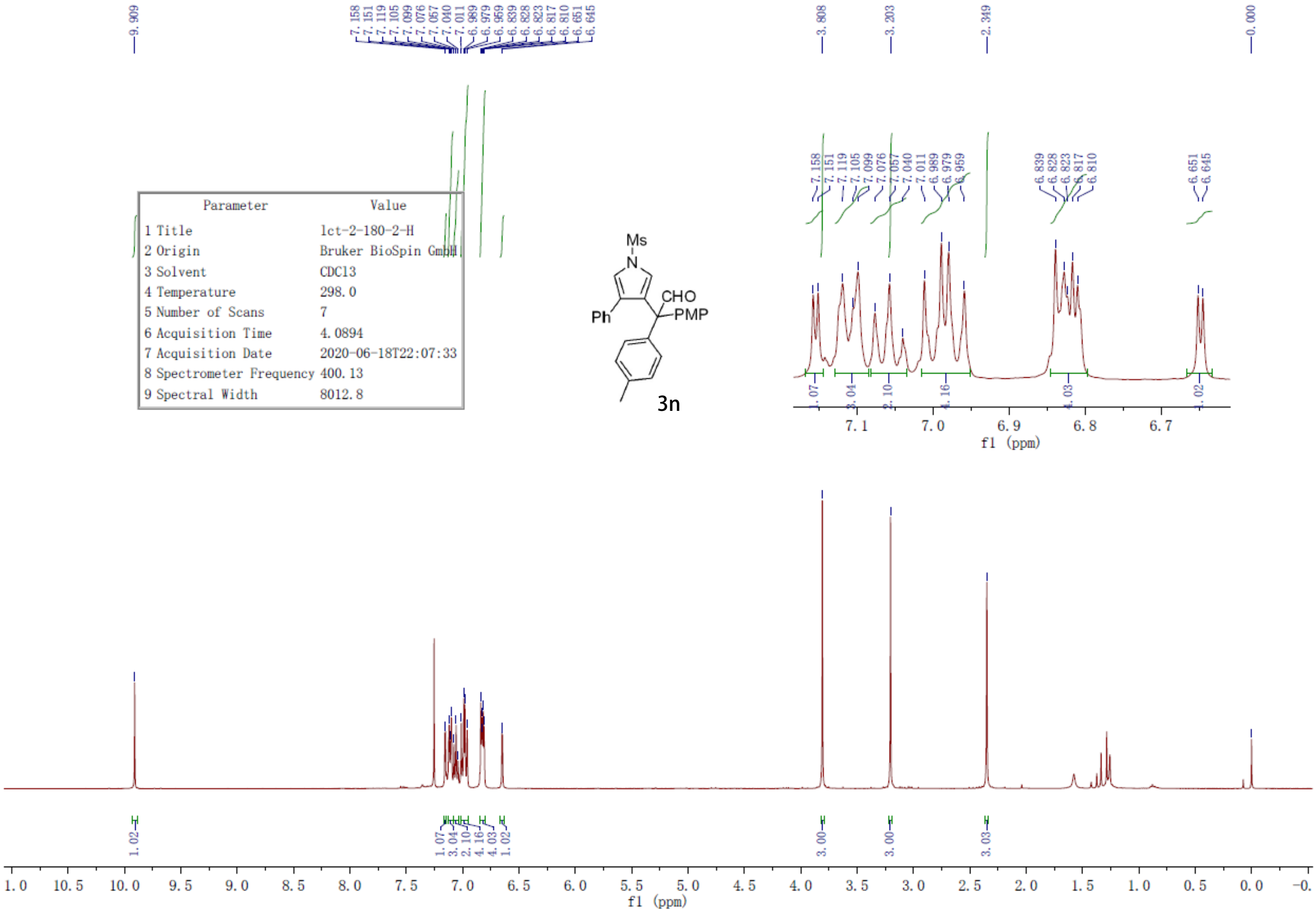

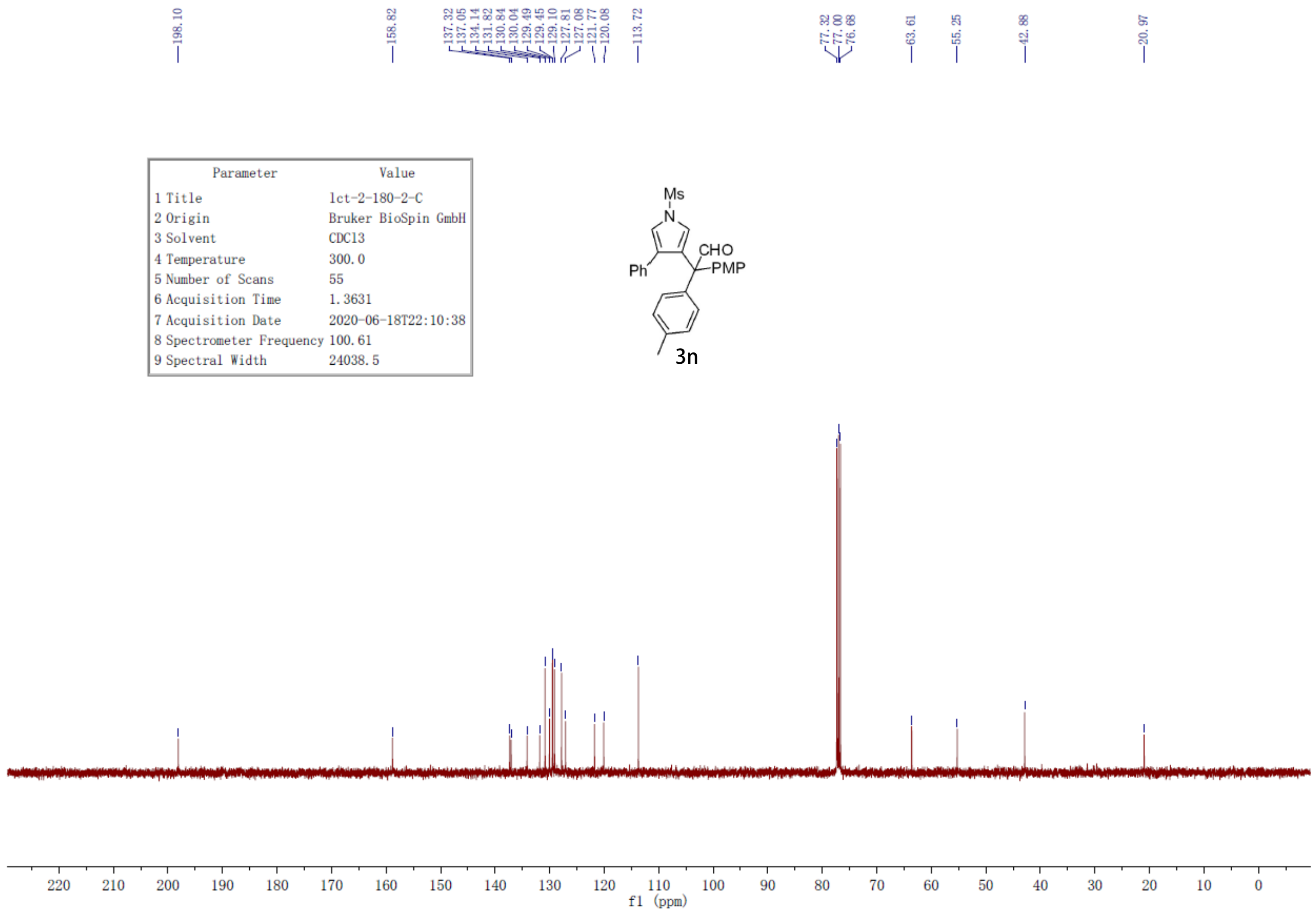

Supplementary Figure 54. ^{1}H and ^{13}C NMR spectra for **3n**

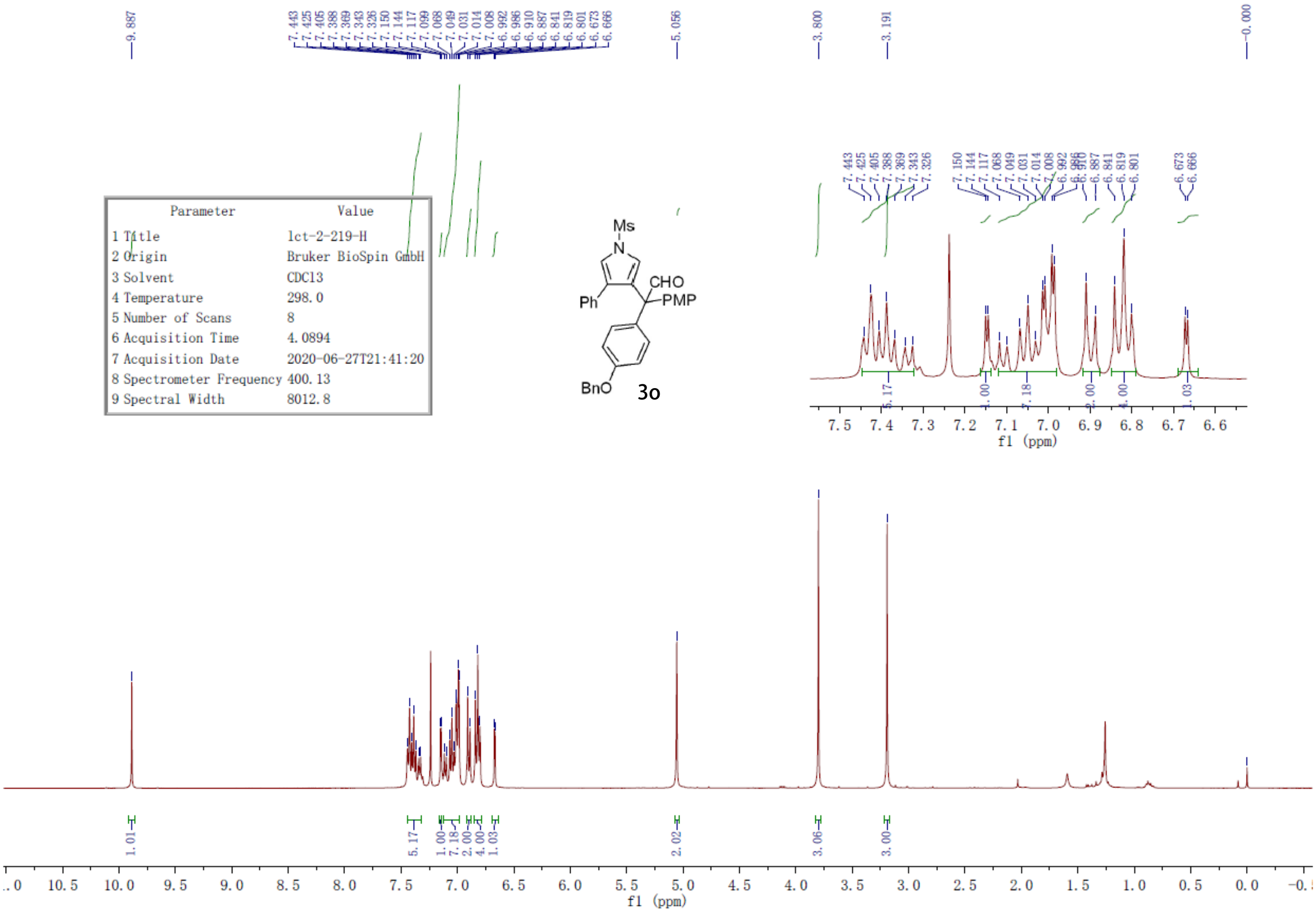

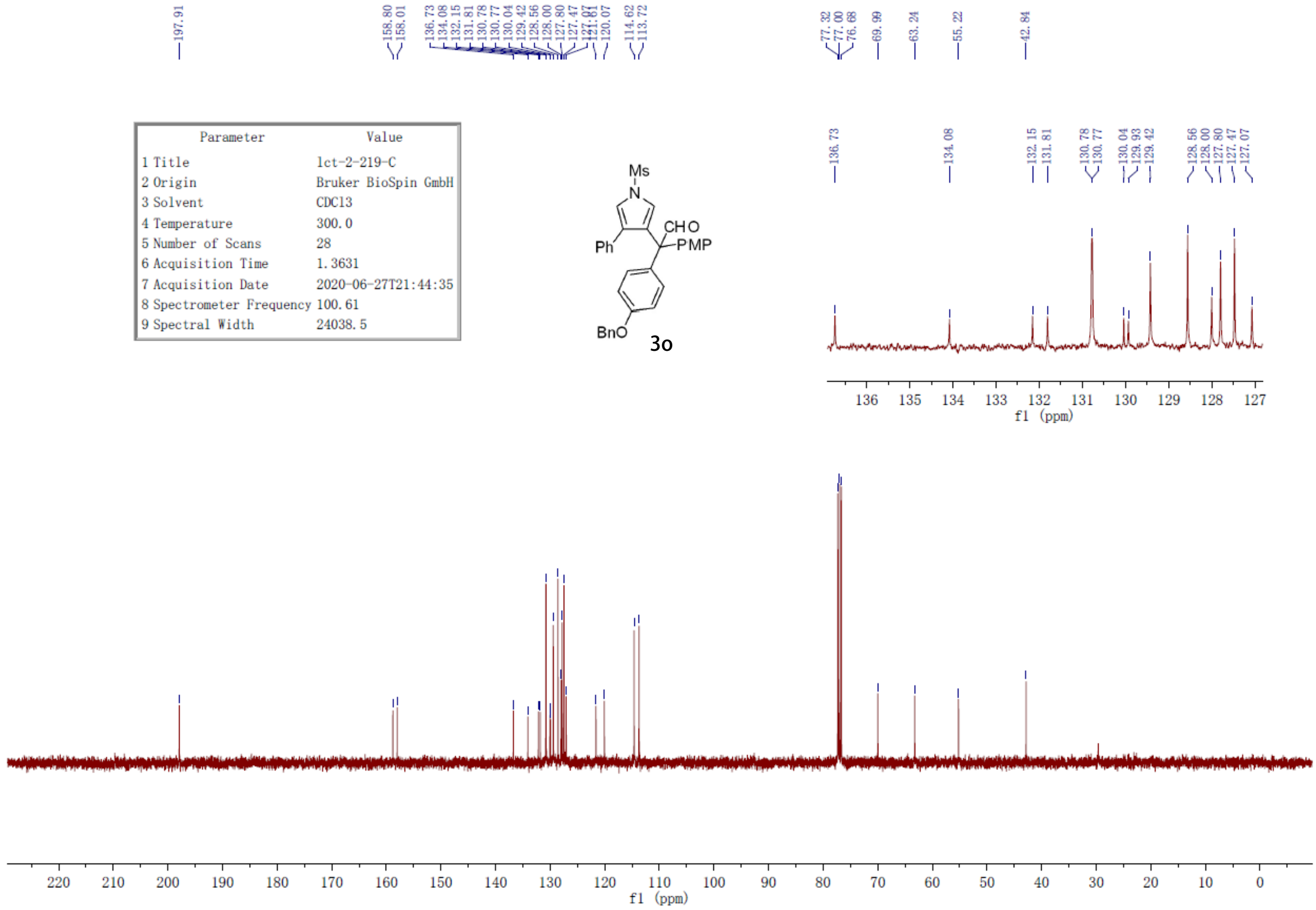

Supplementary Figure 55. ^{1}H and ^{13}C NMR spectra for **3o**

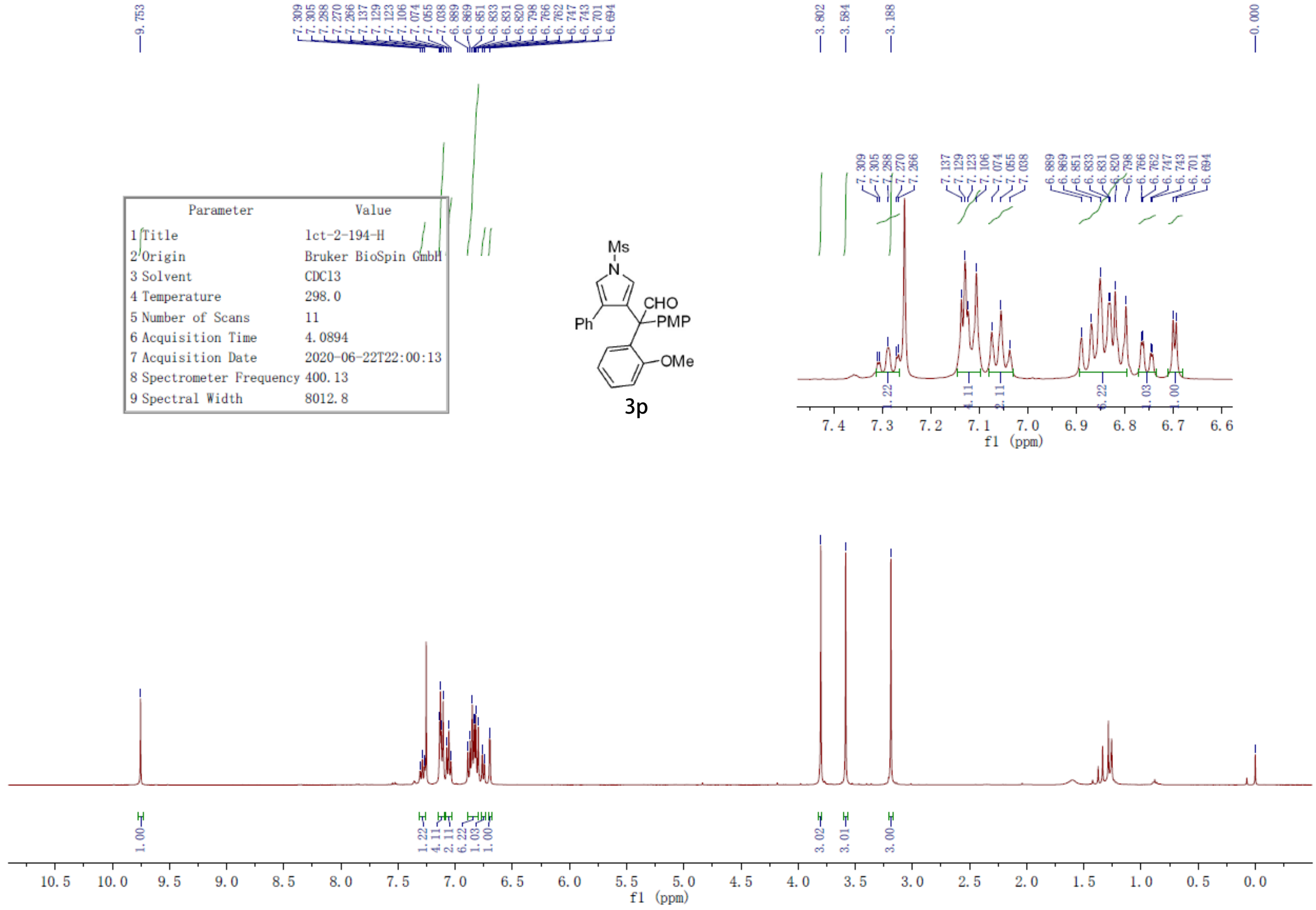

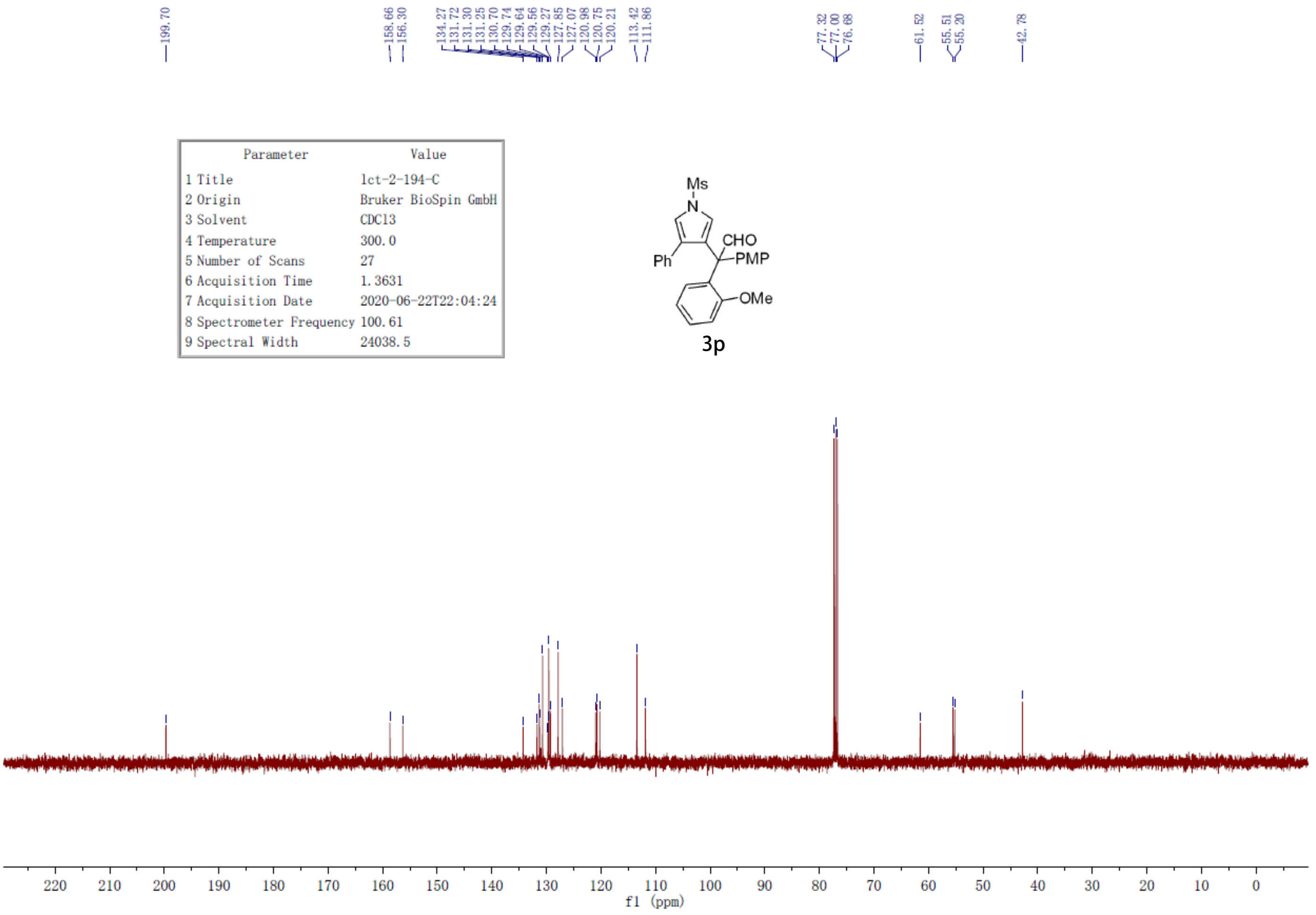

Supplementary Figure 56. ^{1}H and ^{13}C NMR spectra for **3p**

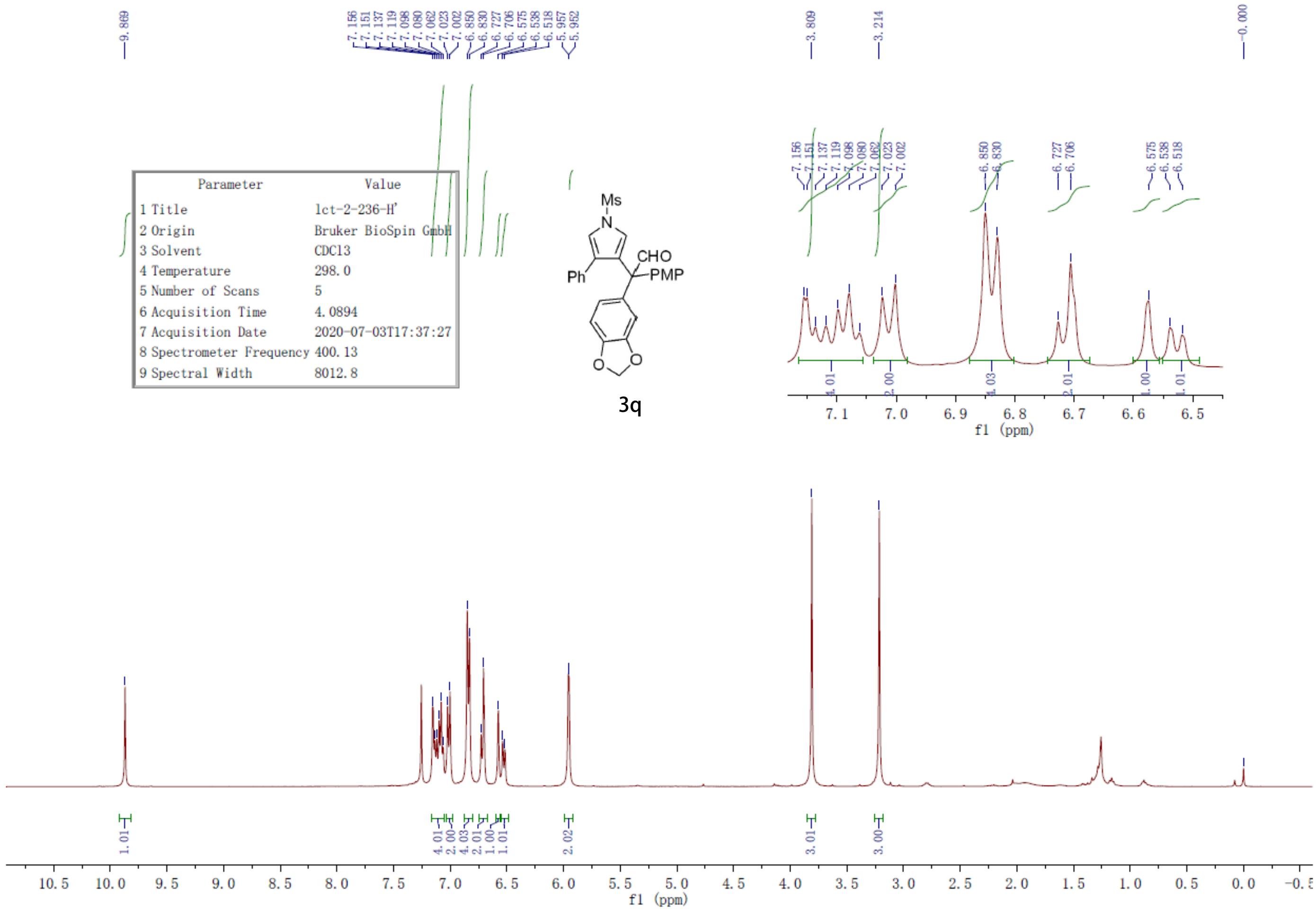

Parameter	Value
1 Title	lct-2-236-H'
2 Origin	Bruker BioSpin GmbH
3 Solvent	CDCl3
4 Temperature	298.0
5 Number of Scans	5
6 Acquisition Time	4.0894
7 Acquisition Date	2020-07-03T17:37:27
8 Spectrometer Frequency	400.13
9 Spectral Width	8012.8

3q

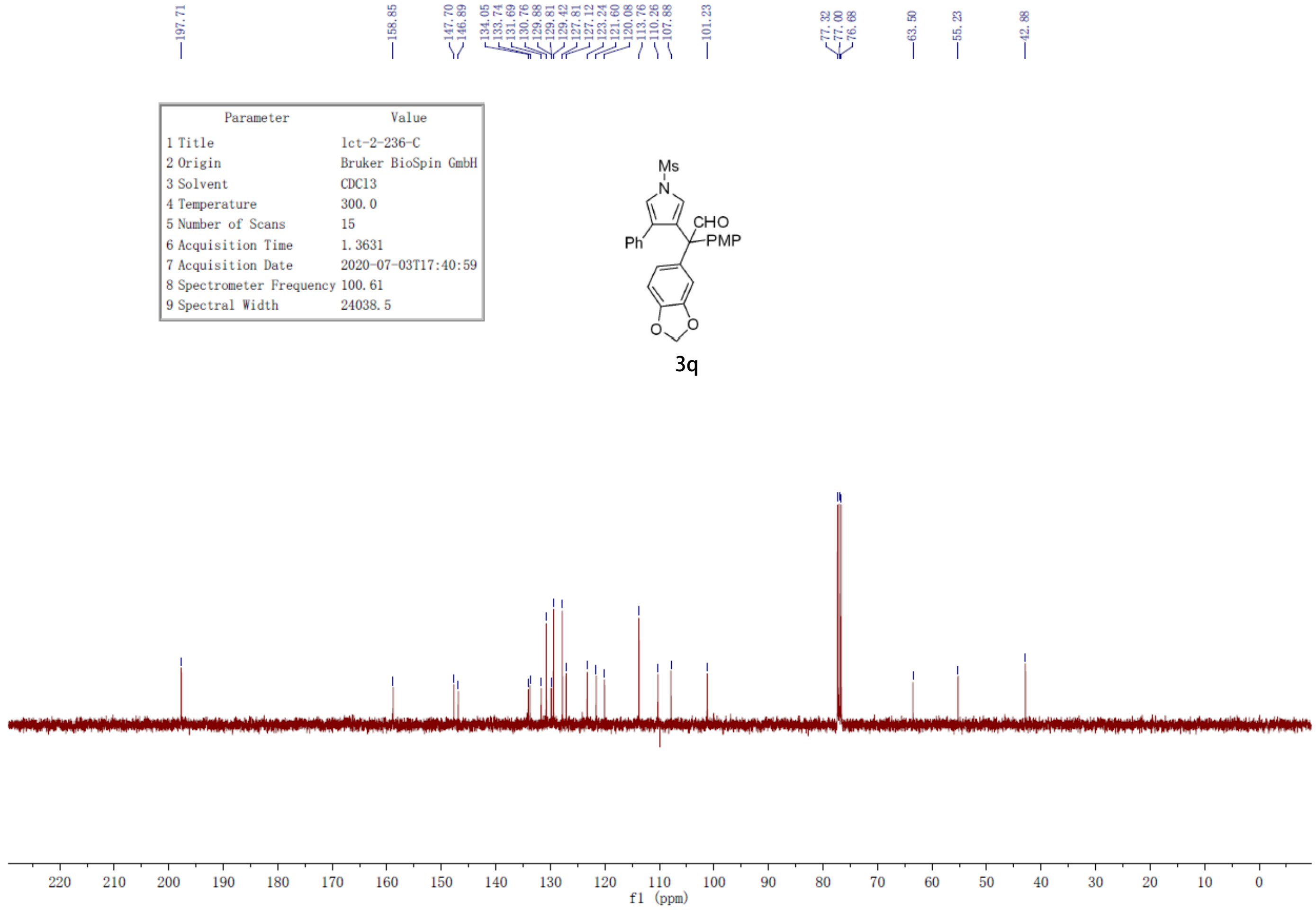

Parameter	Value
1 Title	lct-2-236-C
2 Origin	Bruker BioSpin GmbH
3 Solvent	CDCl3
4 Temperature	300.0
5 Number of Scans	15
6 Acquisition Time	1.3631
7 Acquisition Date	2020-07-03T17:40:59
8 Spectrometer Frequency	100.61
9 Spectral Width	24038.5

3q

Supplementary Figure 57. ^{1}H and ^{13}C NMR spectra for **3q**

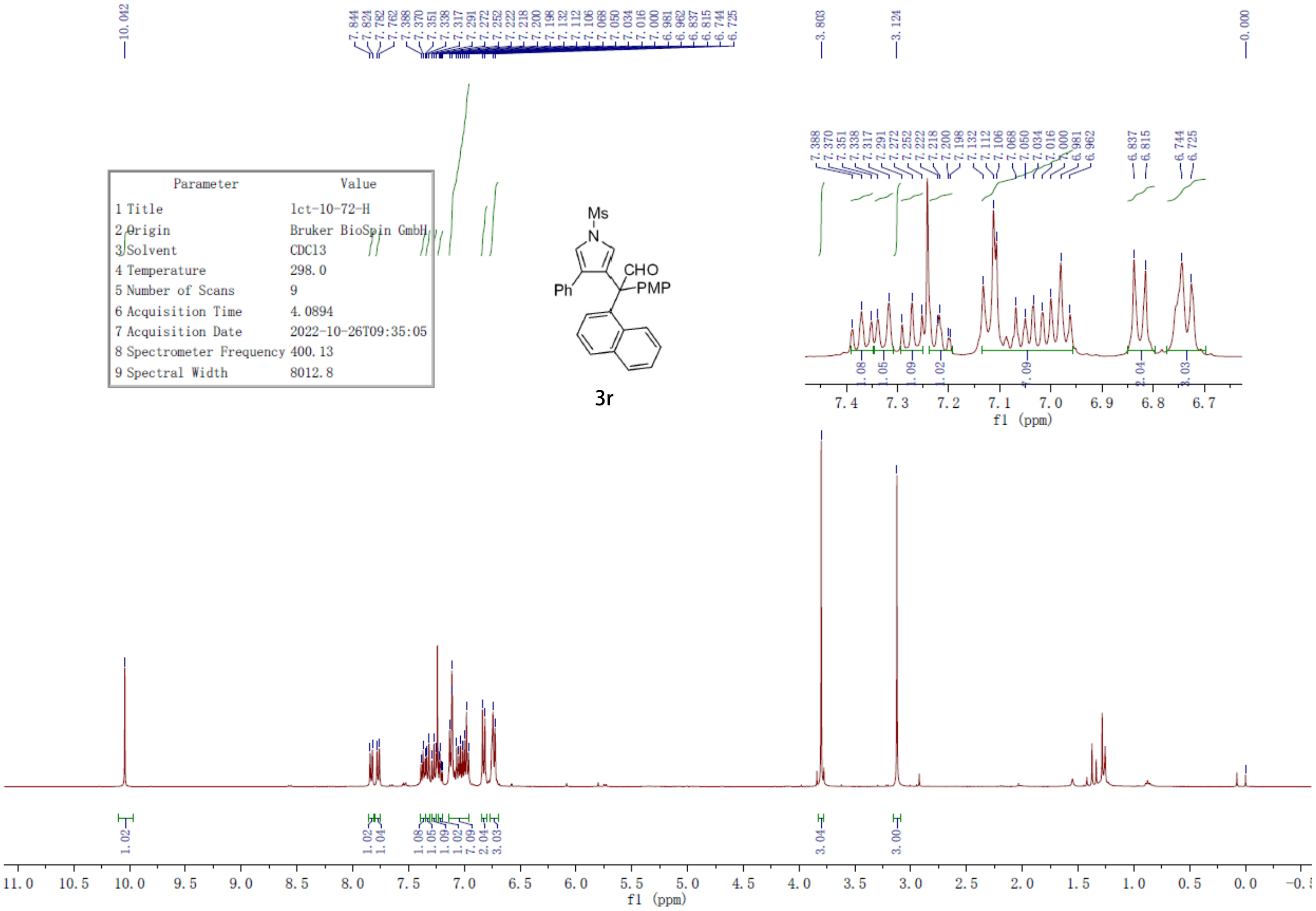

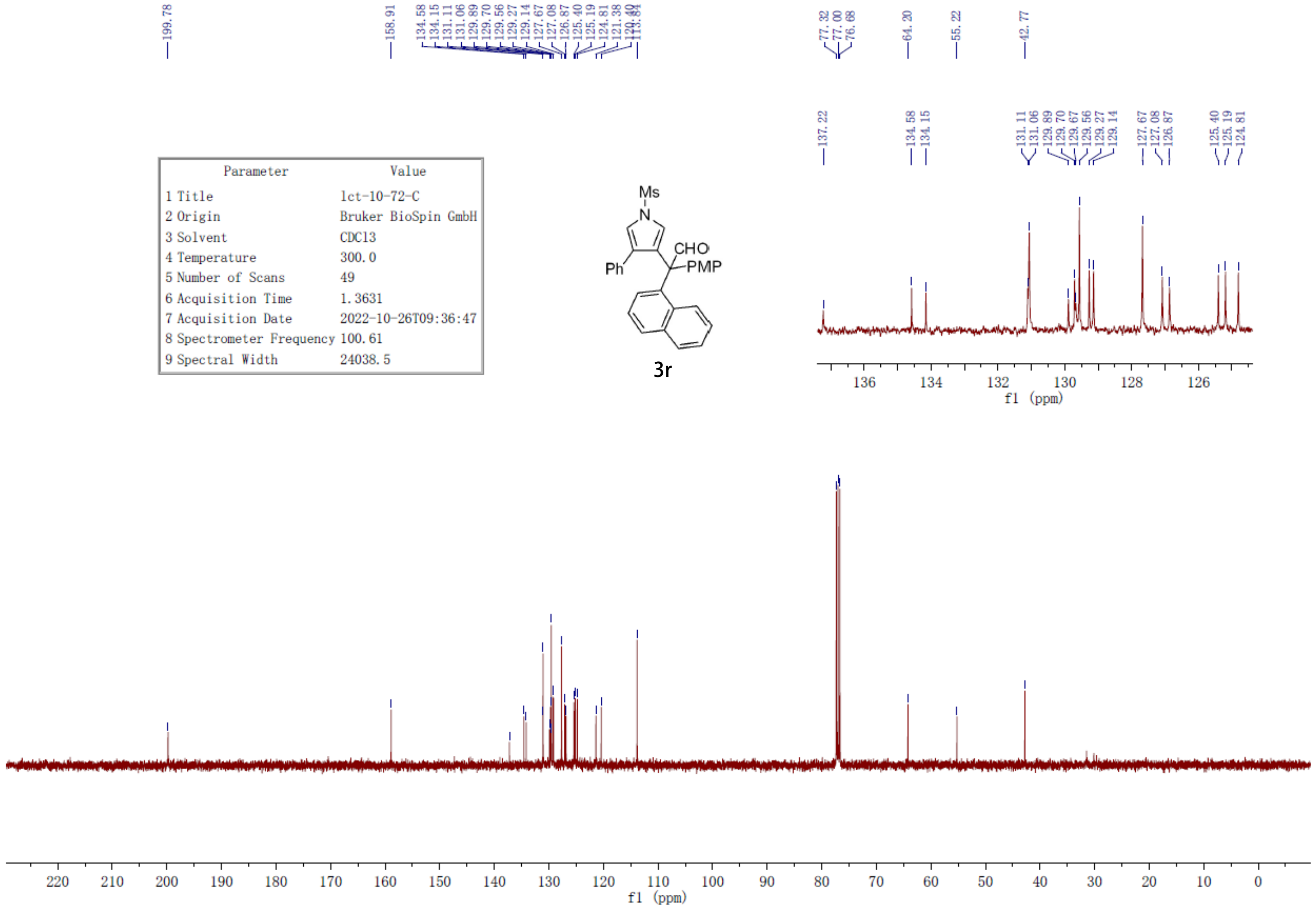

Supplementary Figure 58. ^{1}H and ^{13}C NMR spectra for **3r**

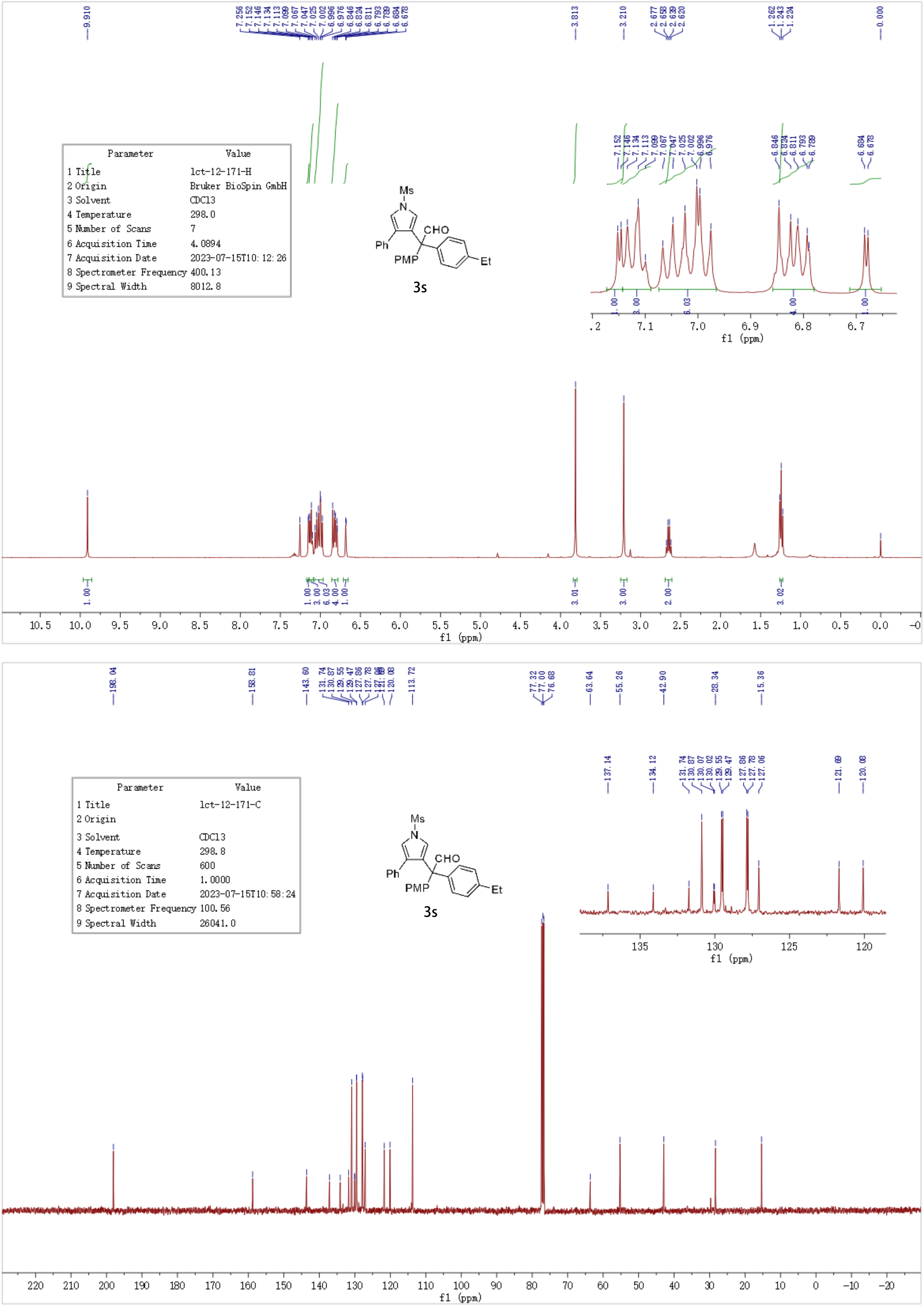

Supplementary Figure 59. ^{1}H and ^{13}C NMR spectra for **3s**

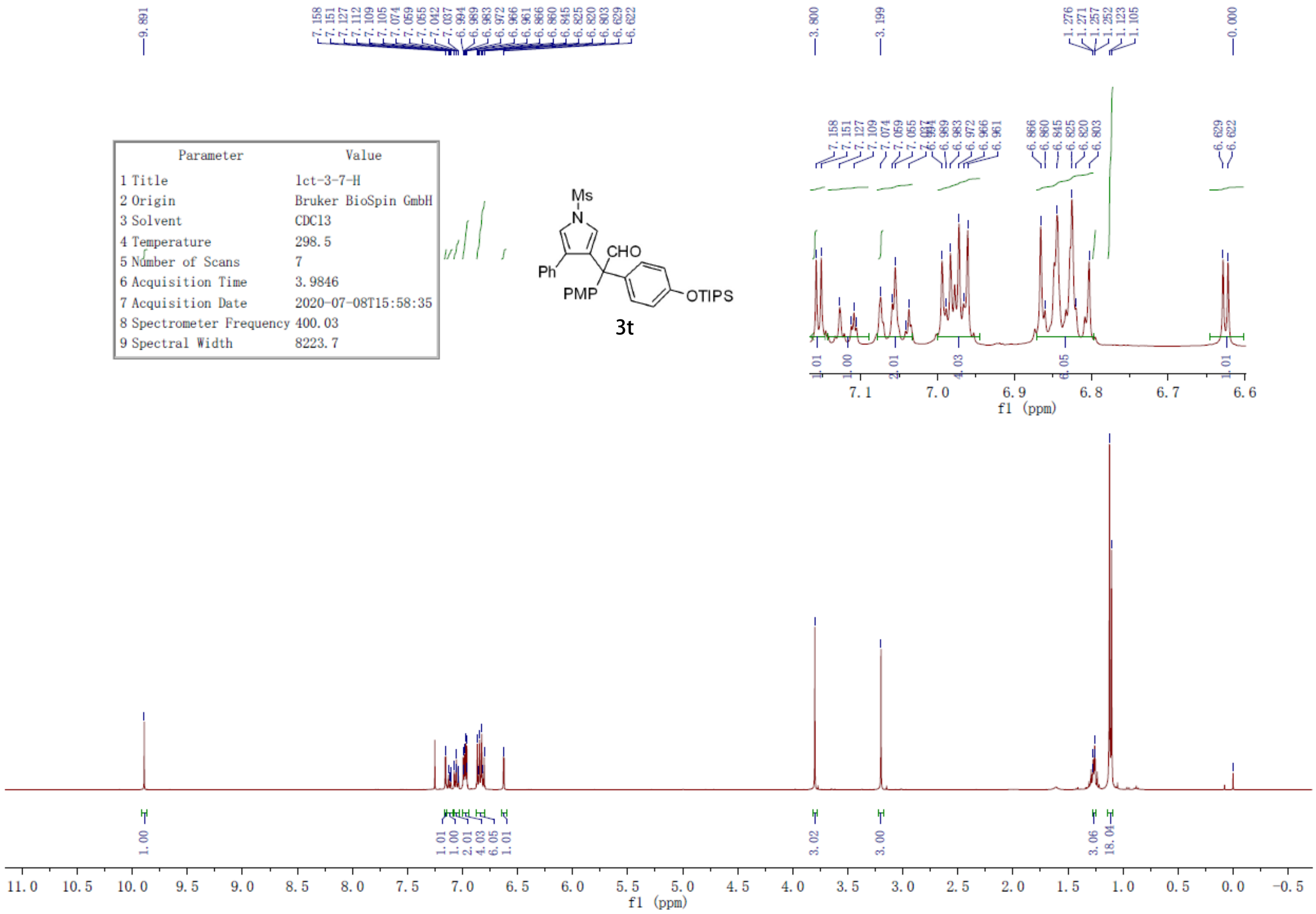

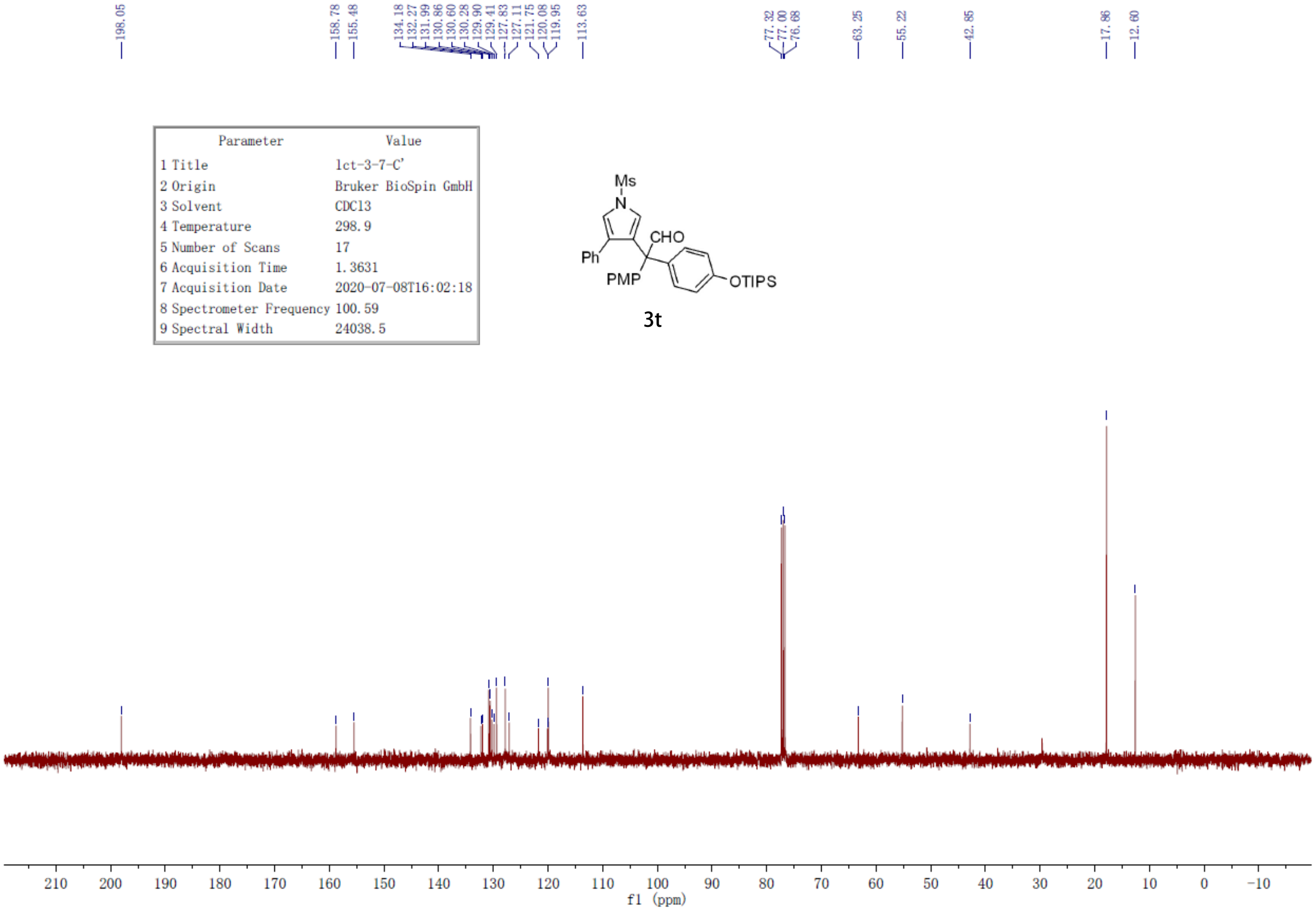

Supplementary Figure 60. ^{1}H and ^{13}C NMR spectra for **3t**

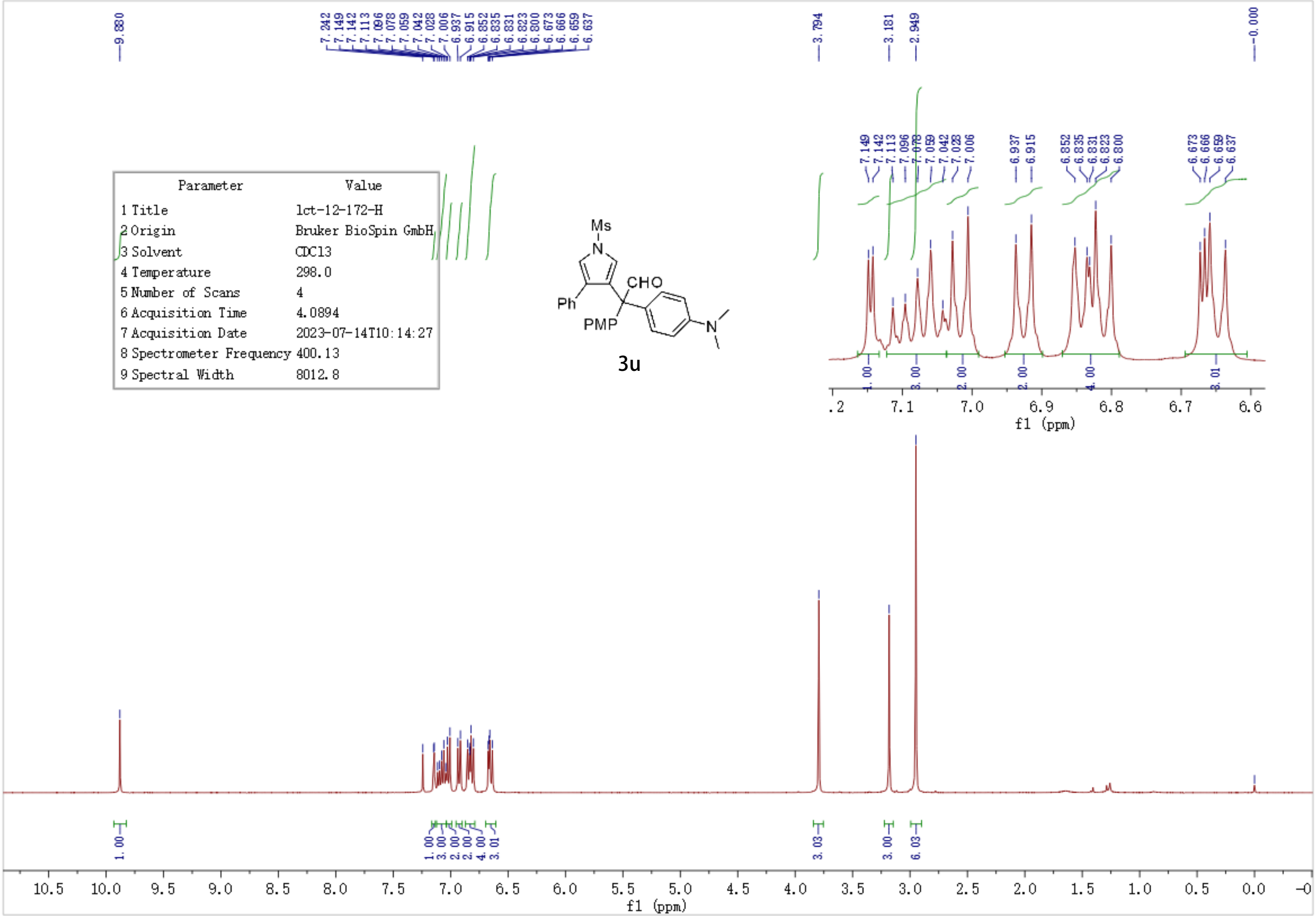

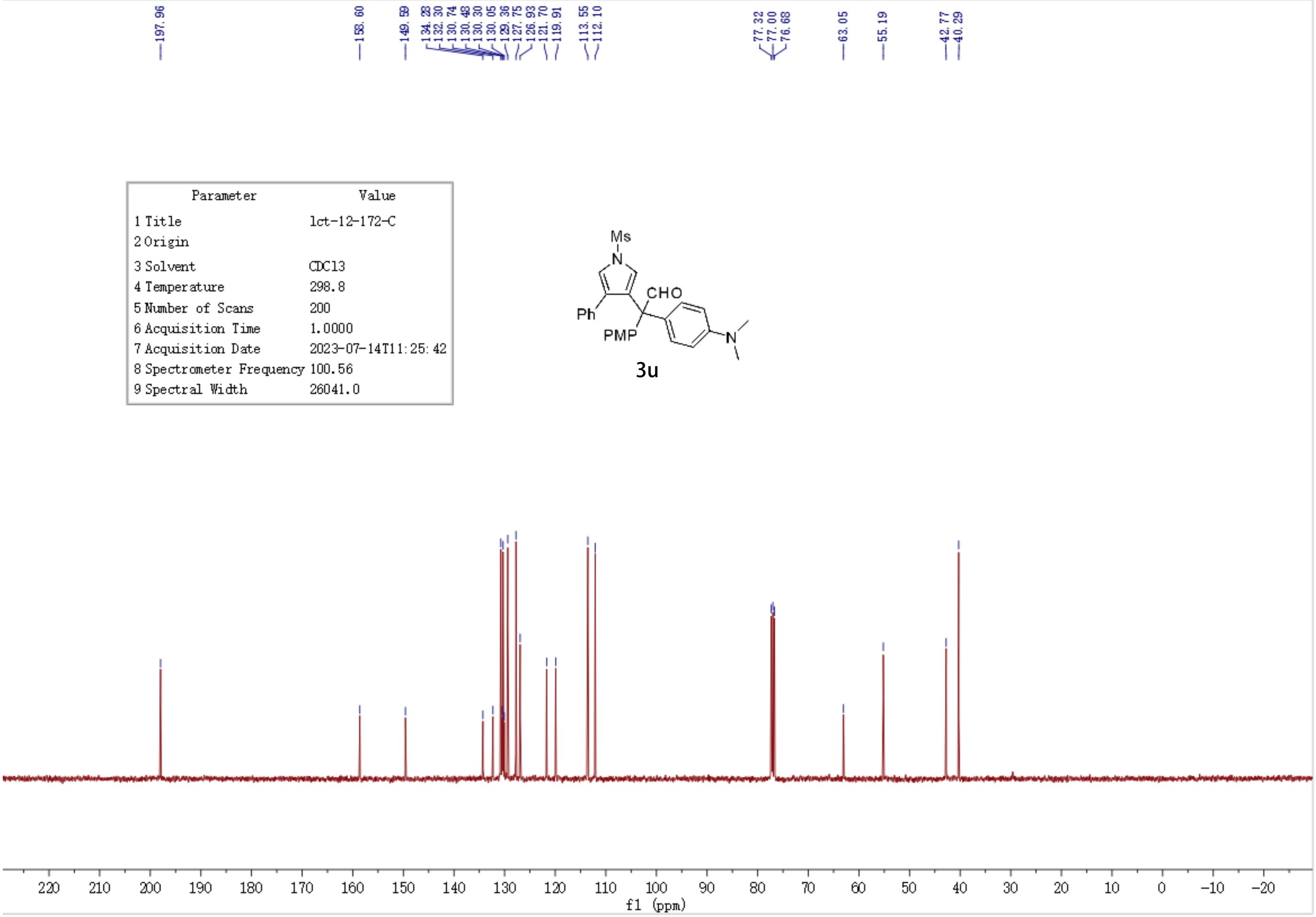

Supplementary Figure 61. ^{1}H and ^{13}C NMR spectra for **3u**

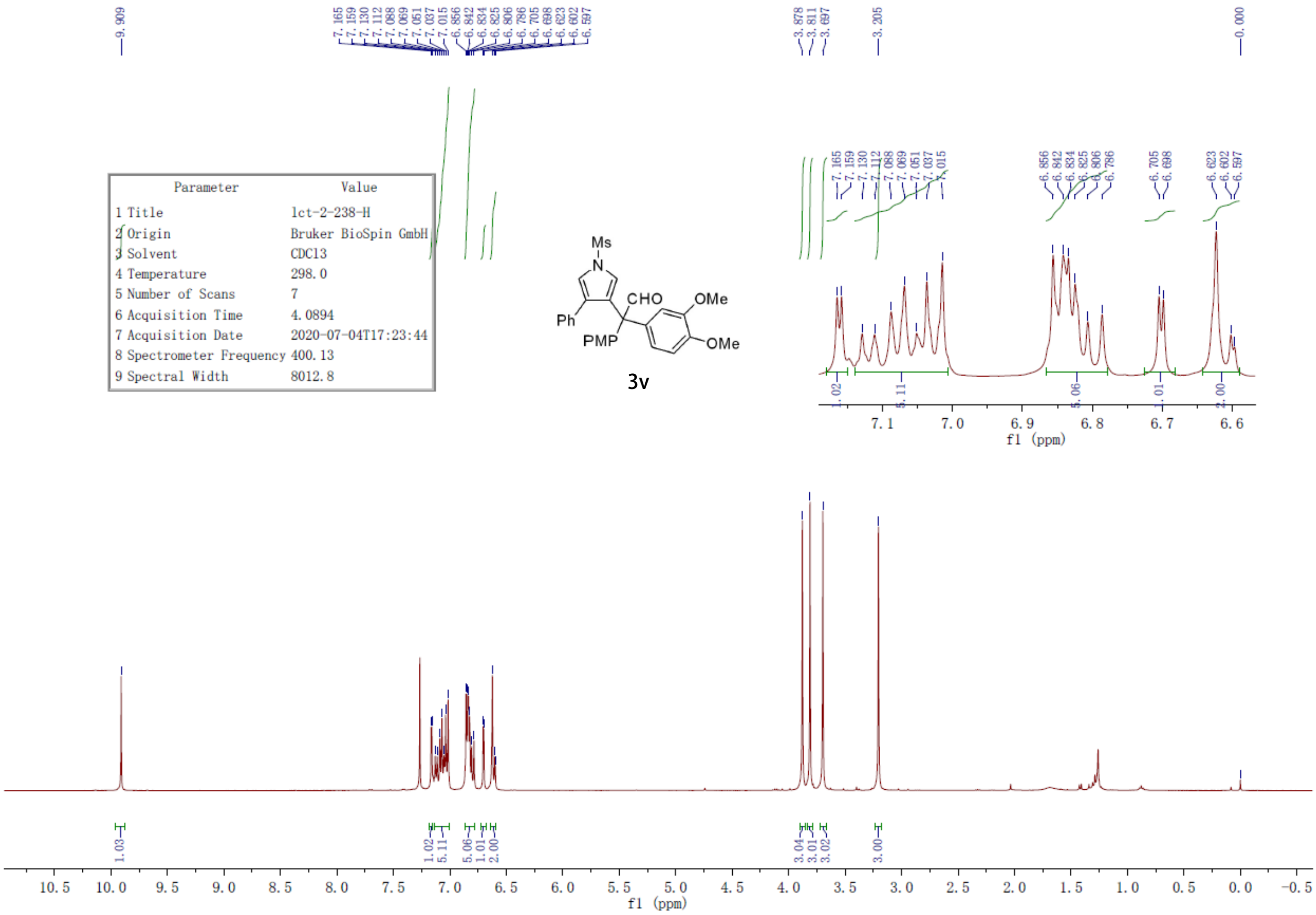

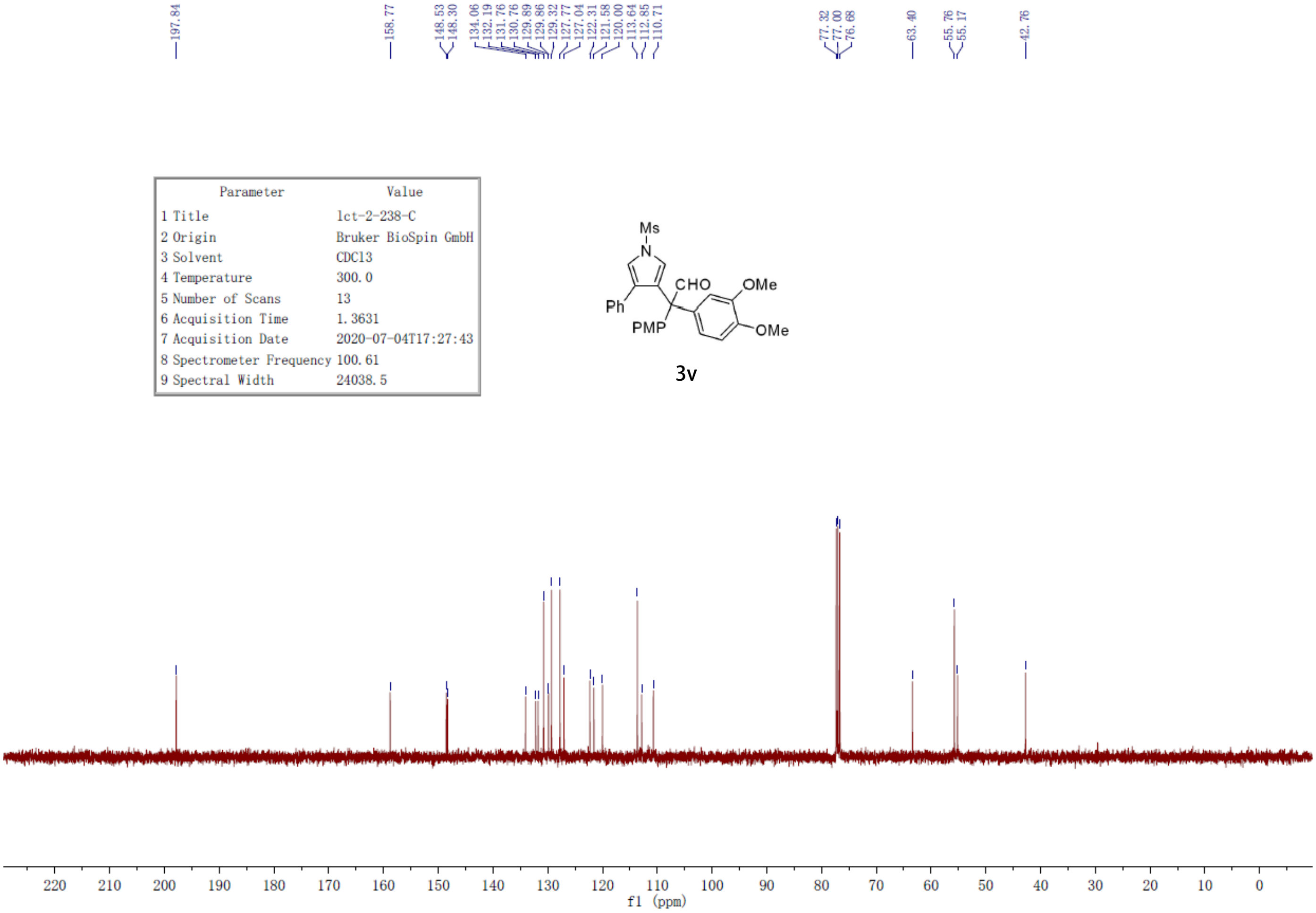

Supplementary Figure 62. ^{1}H and ^{13}C NMR spectra for **3v**

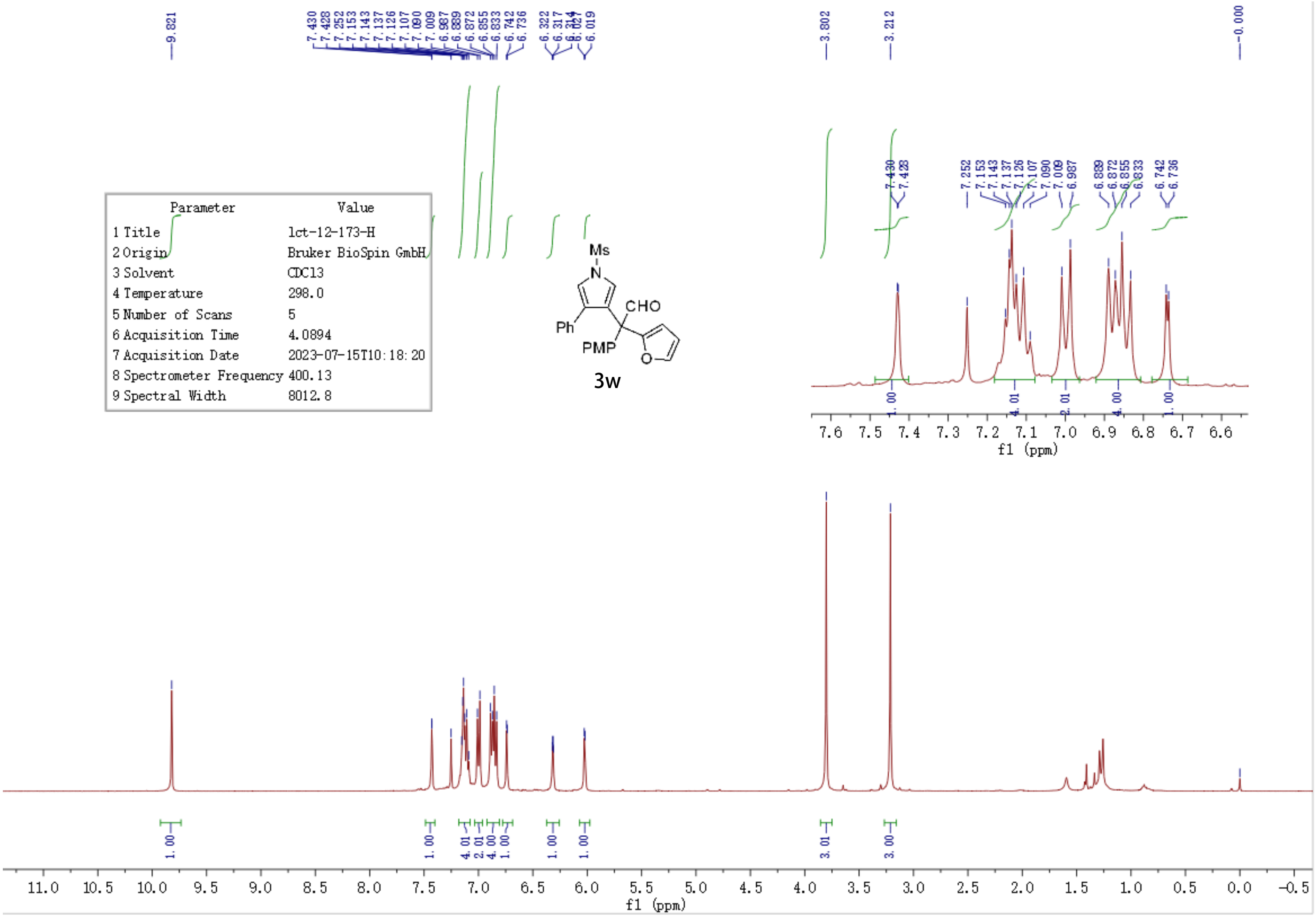

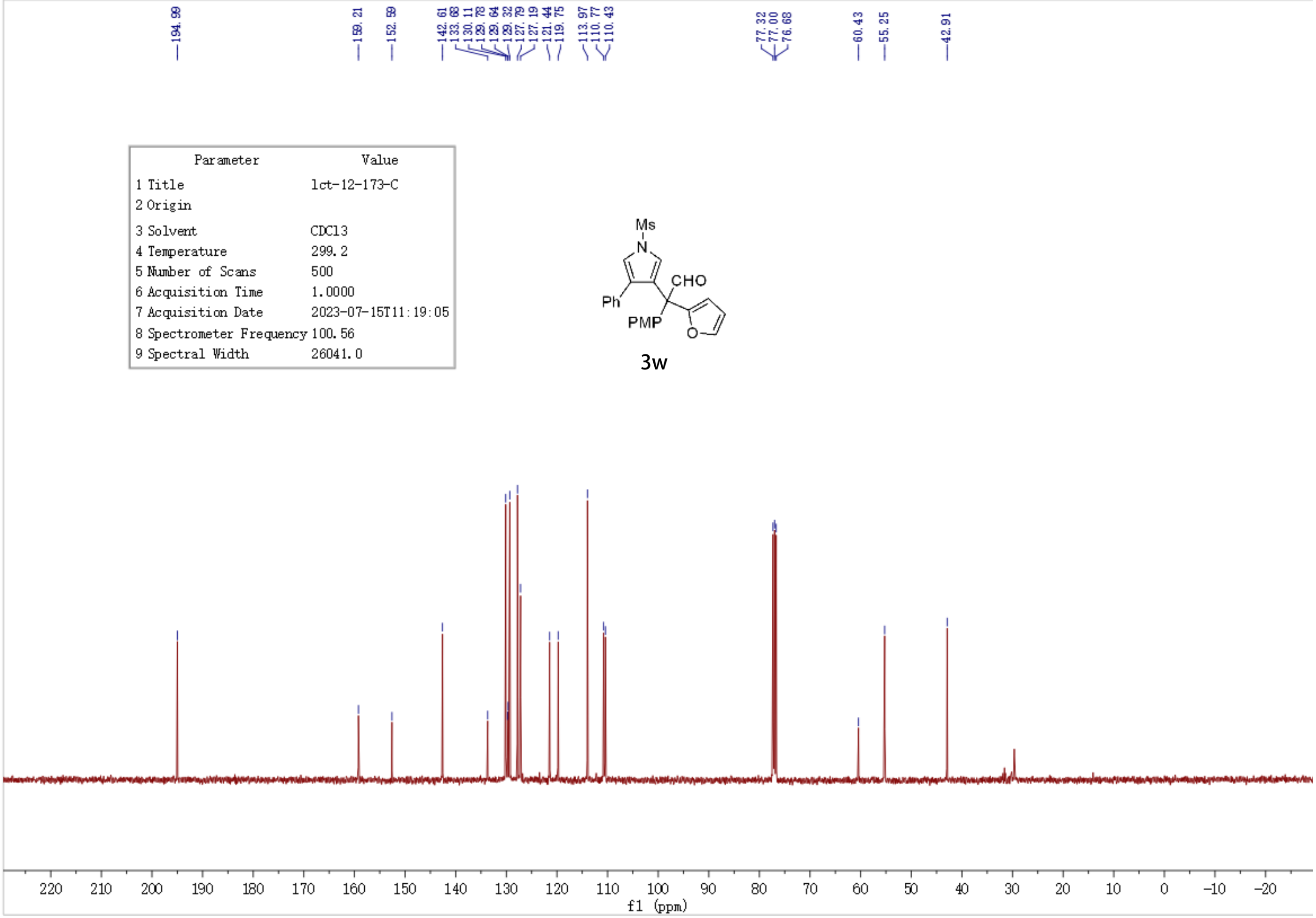

Supplementary Figure 63. ^{1}H and ^{13}C NMR spectra for **3w**

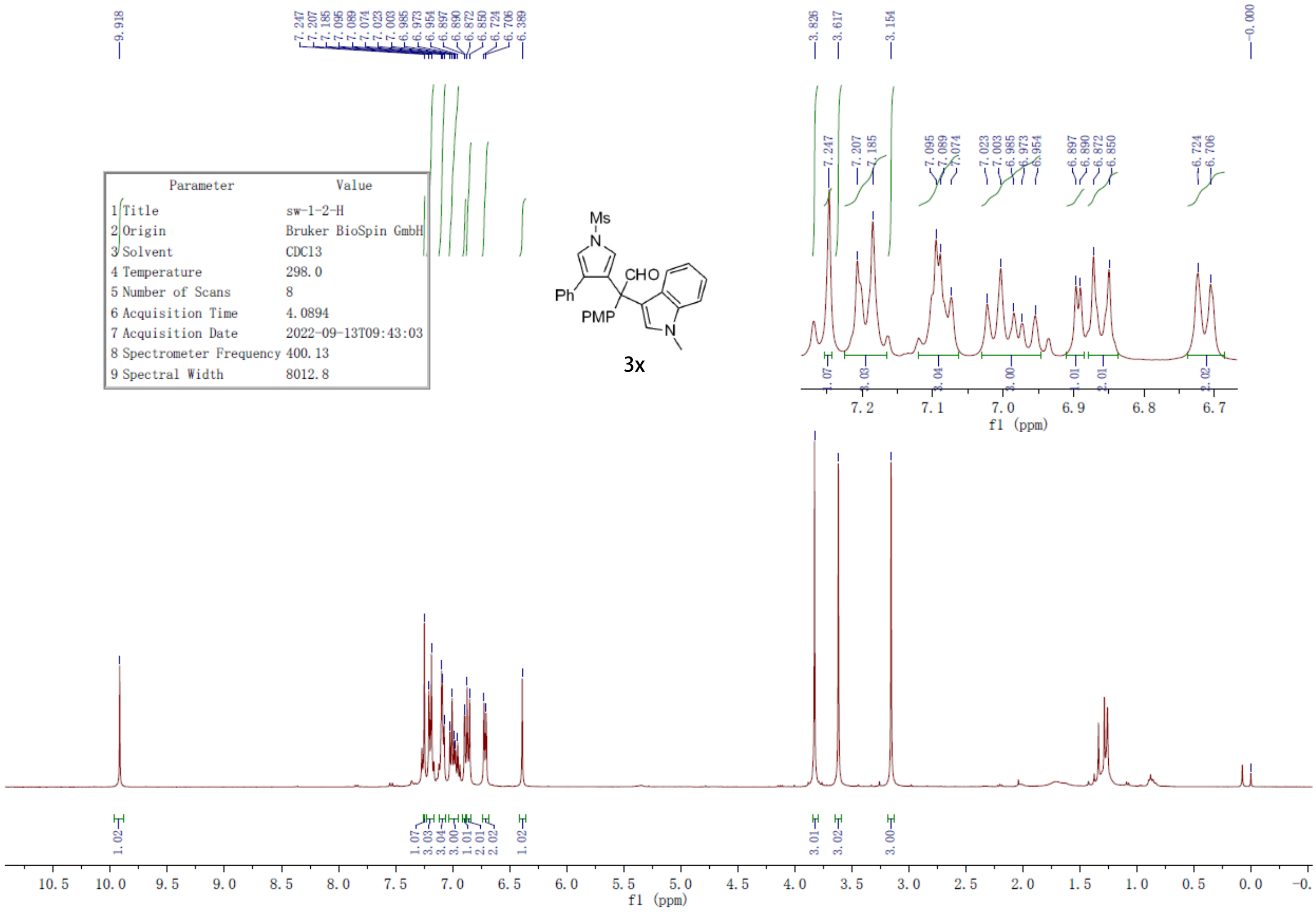

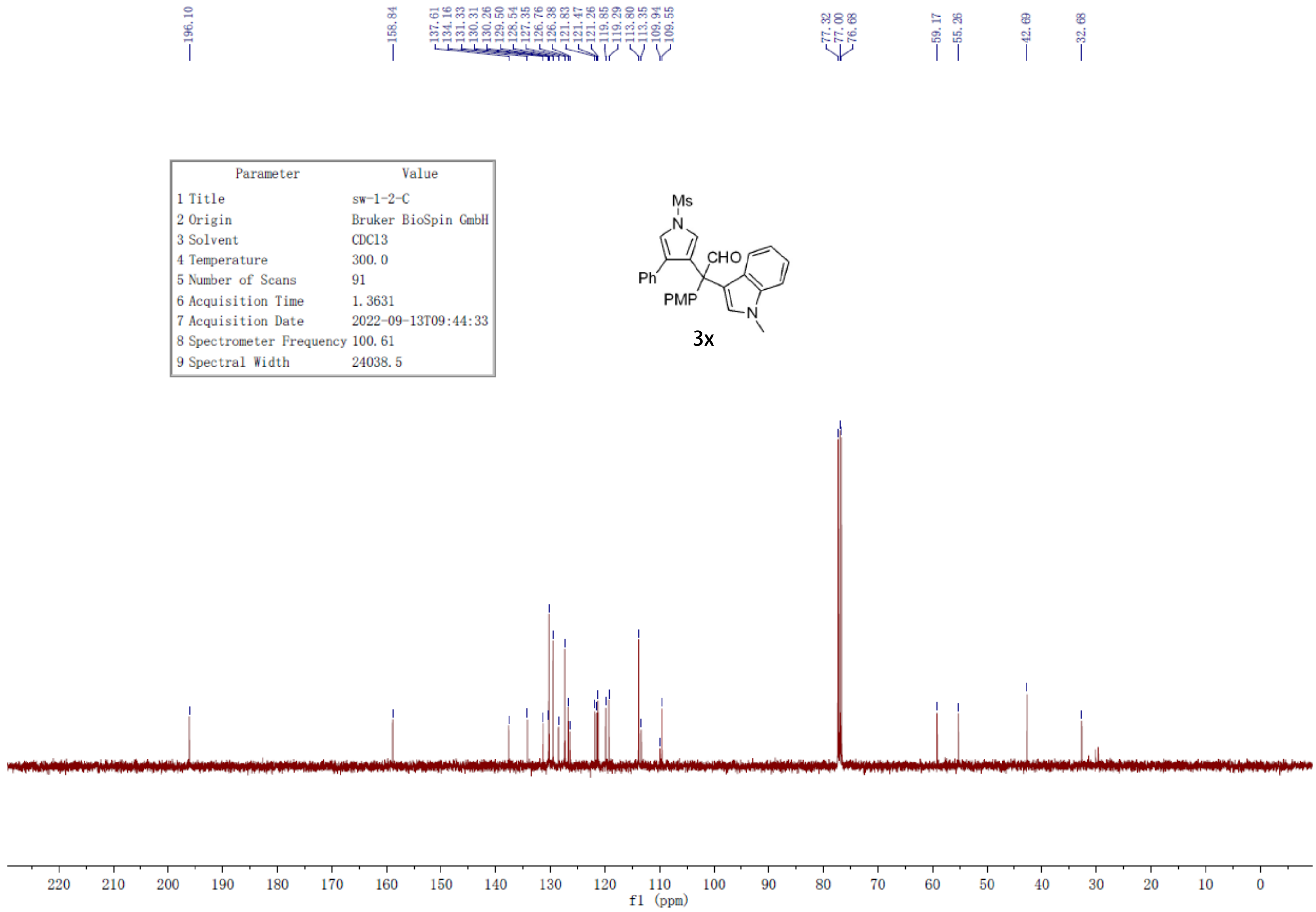

Supplementary Figure 64. ^{1}H and ^{13}C NMR spectra for **3x**

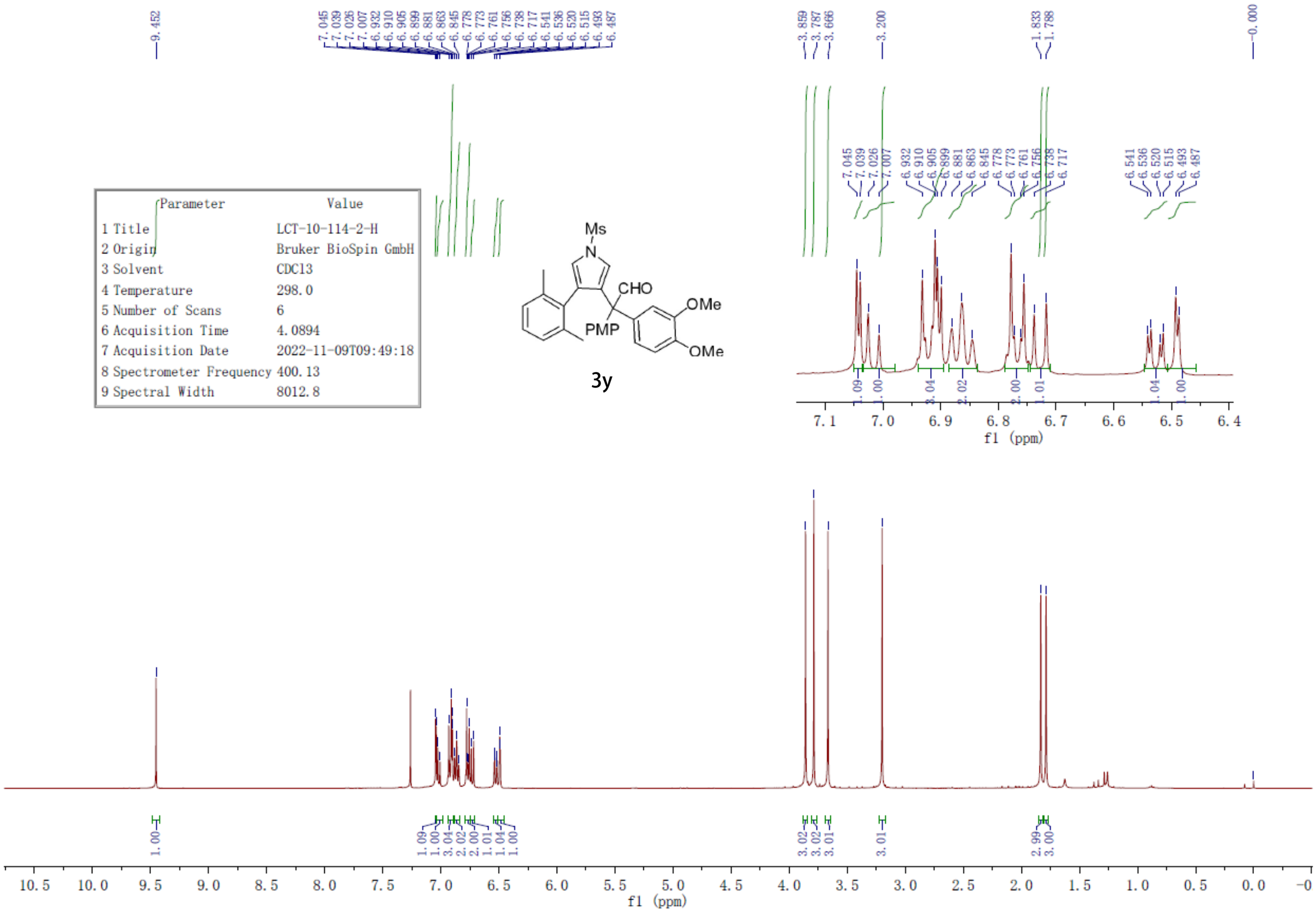

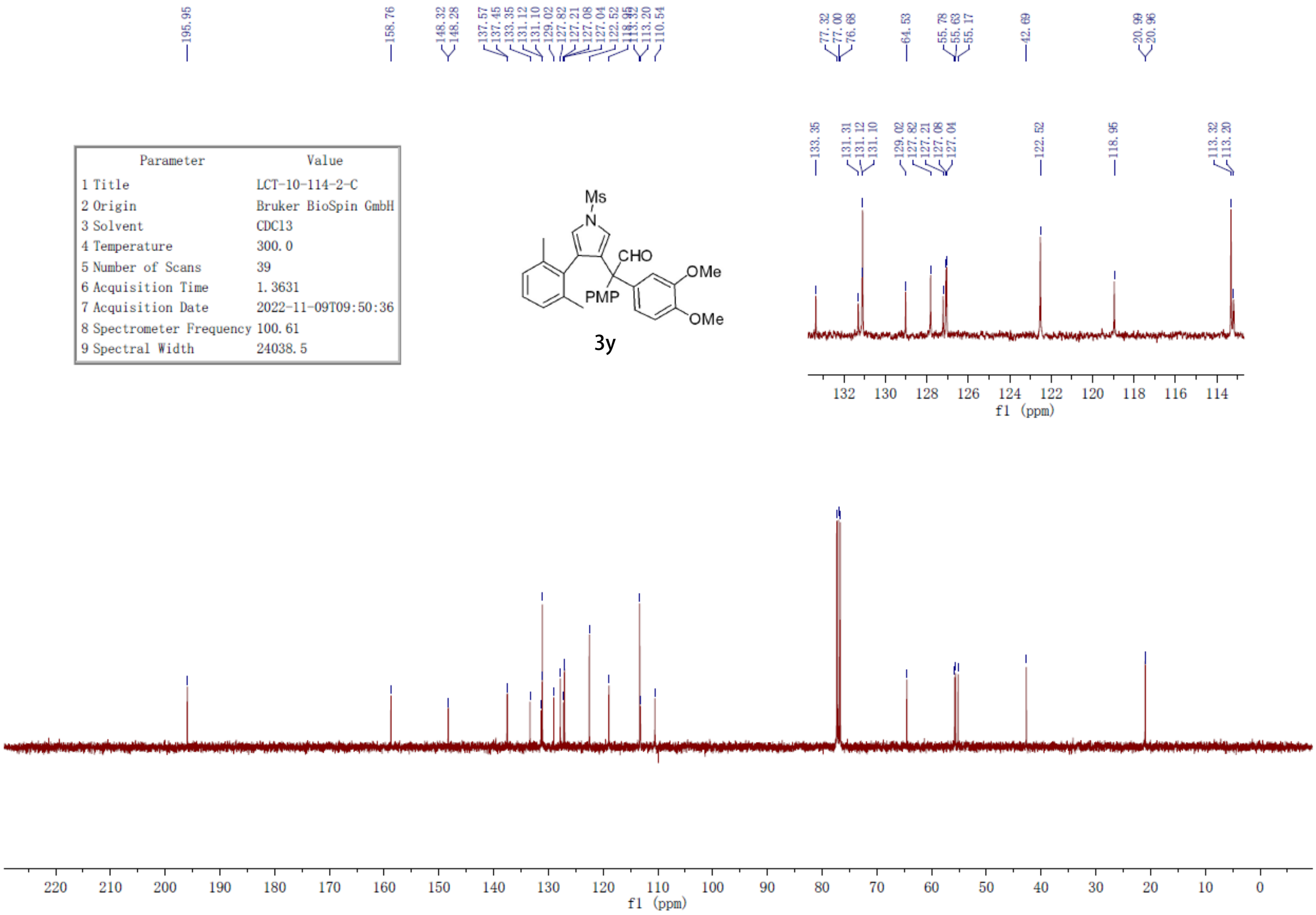

Supplementary Figure 65. ^{1}H and ^{13}C NMR spectra for **3y**

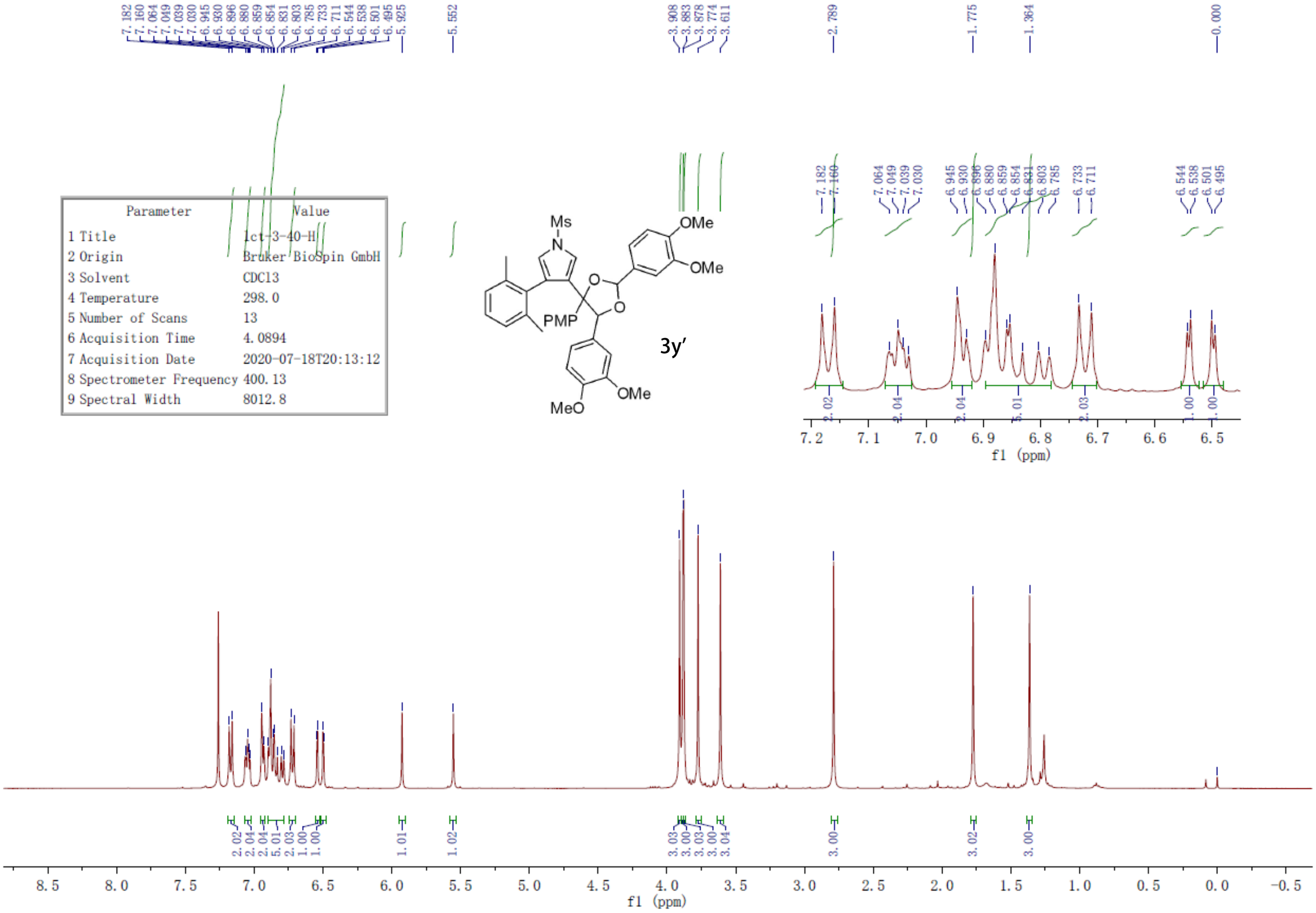

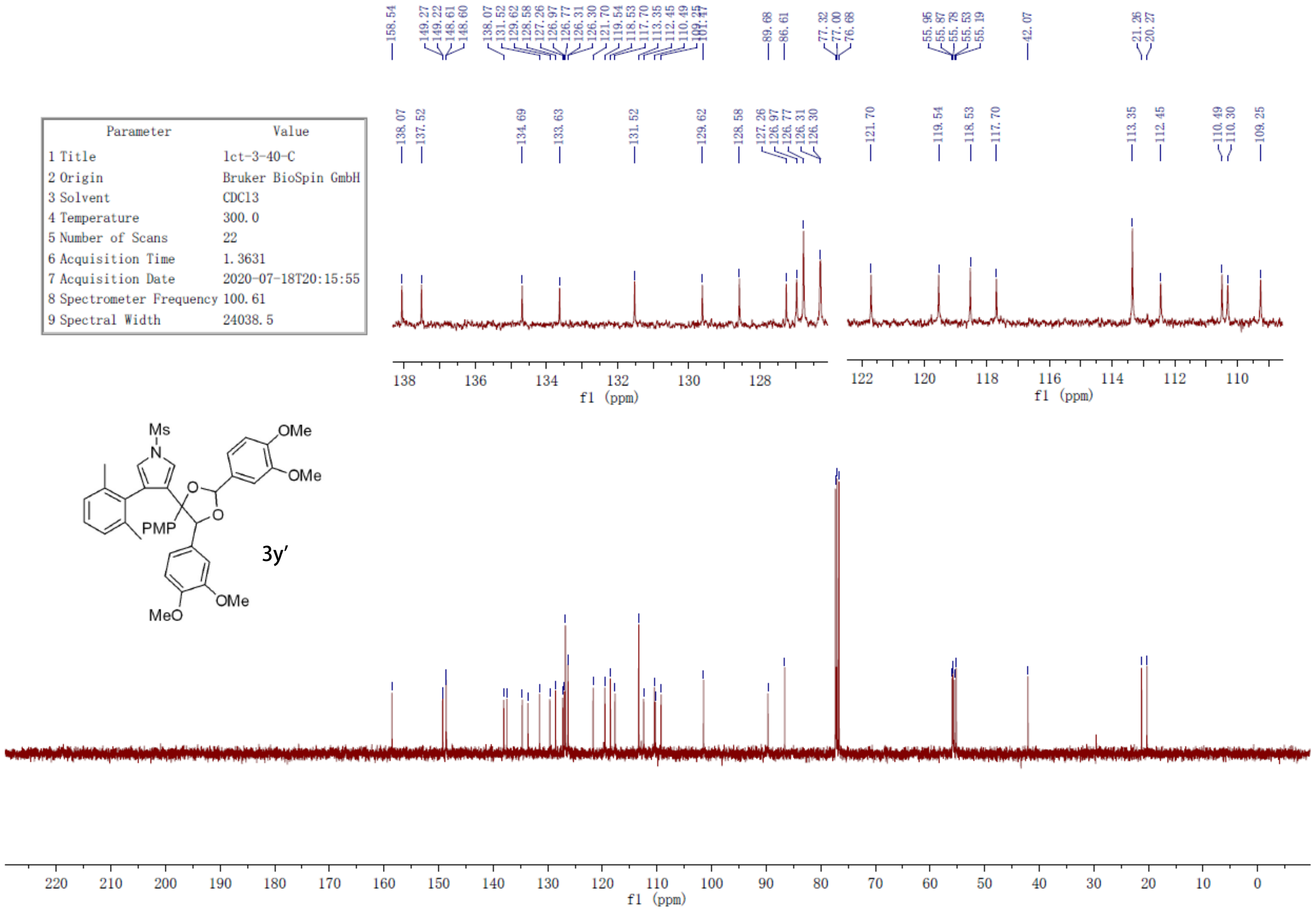

Supplementary Figure 66. ^{1}H and ^{13}C NMR spectra for **3y'**

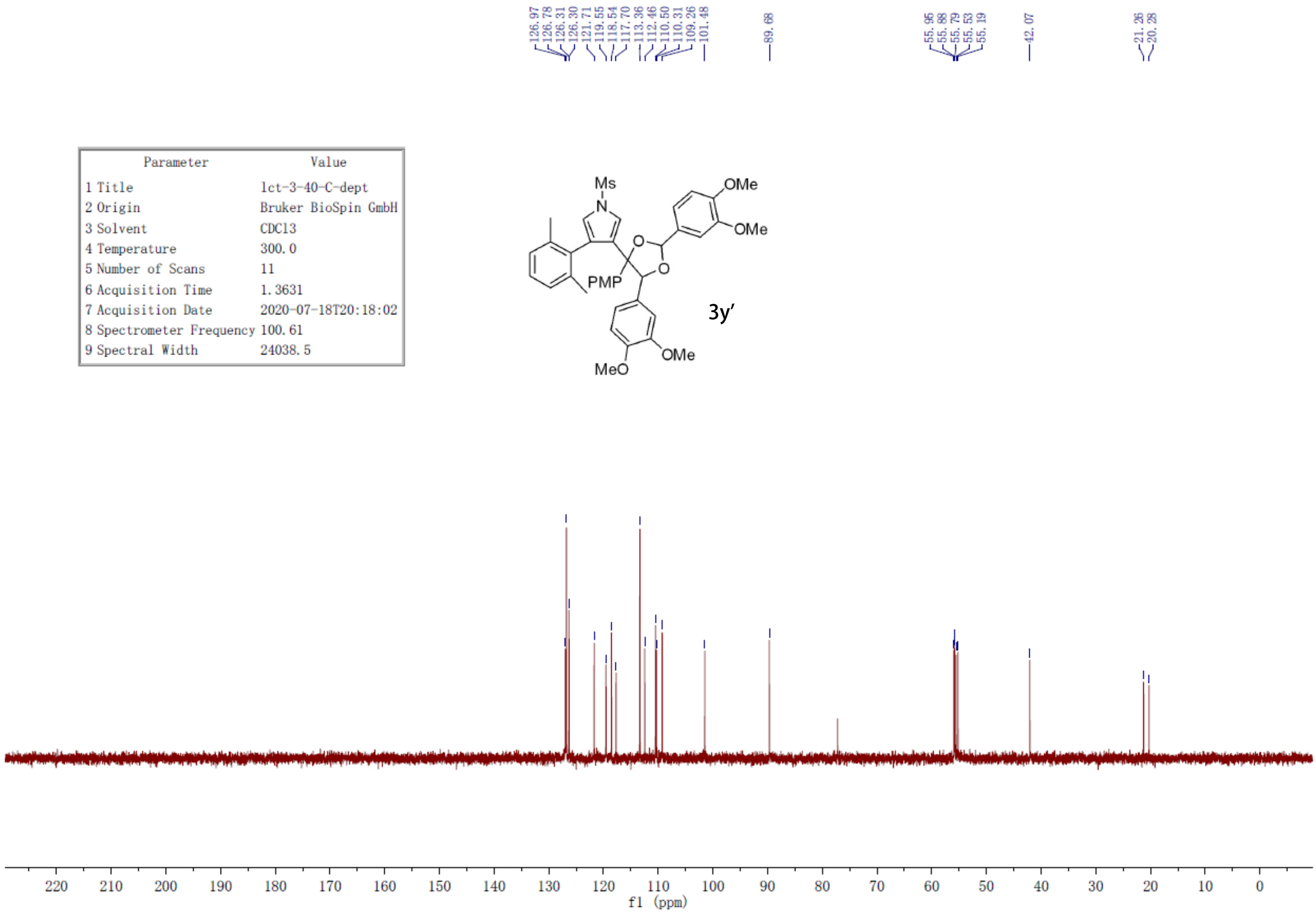

Supplementary Figure 67. DEPT 135 spectra for **3y'**

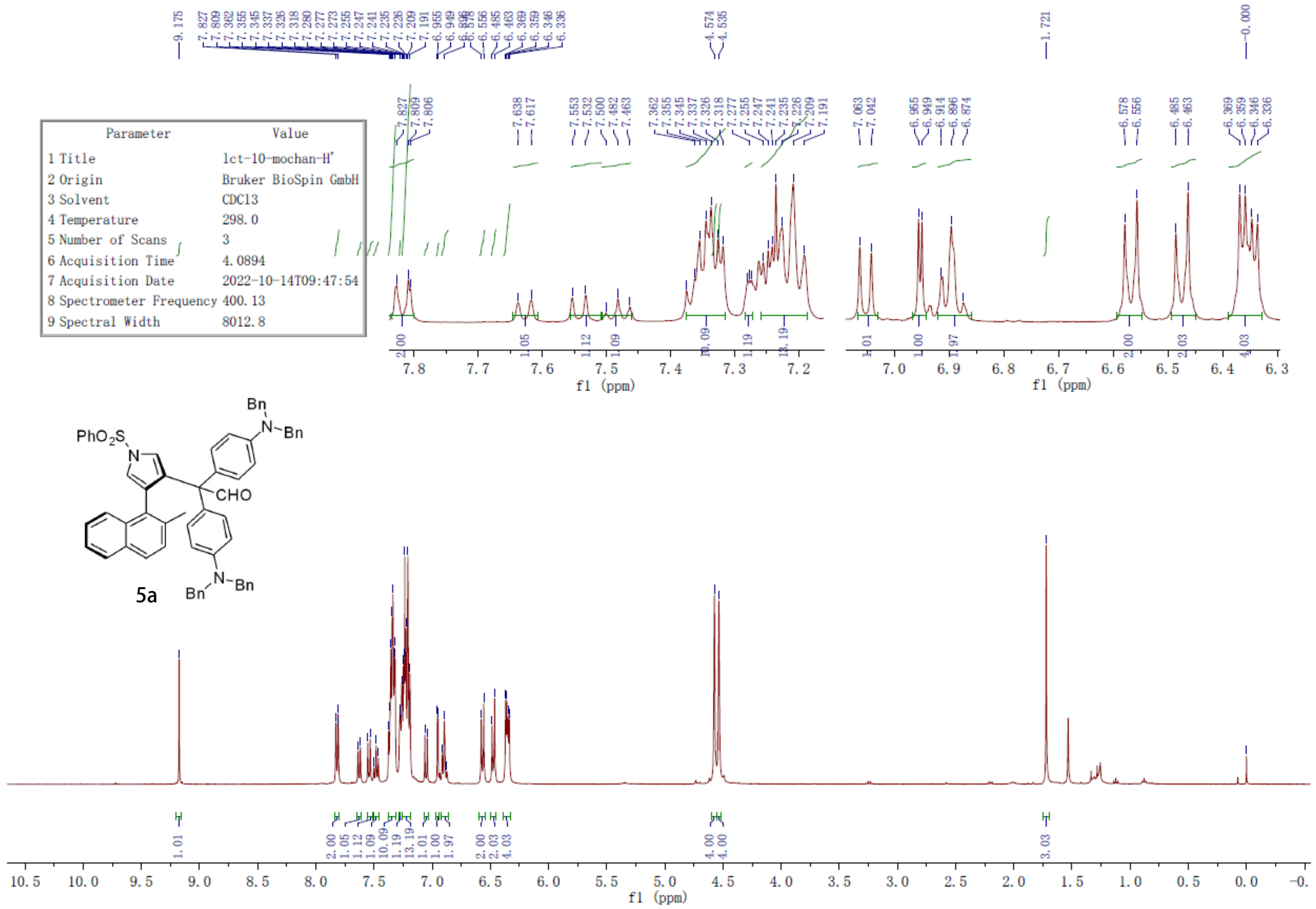

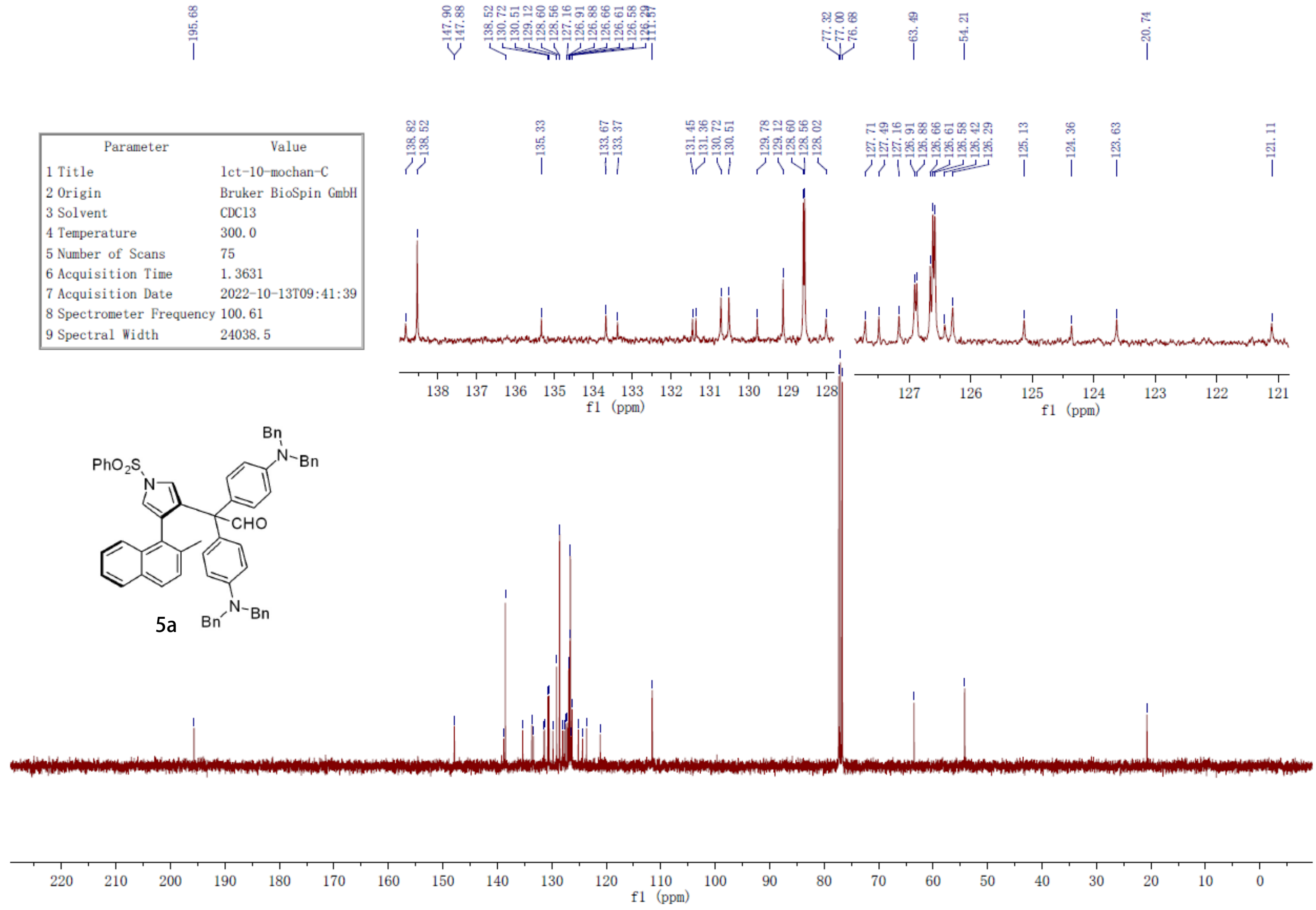

Supplementary Figure 68. ^{1}H and ^{13}C NMR spectra for **5a**

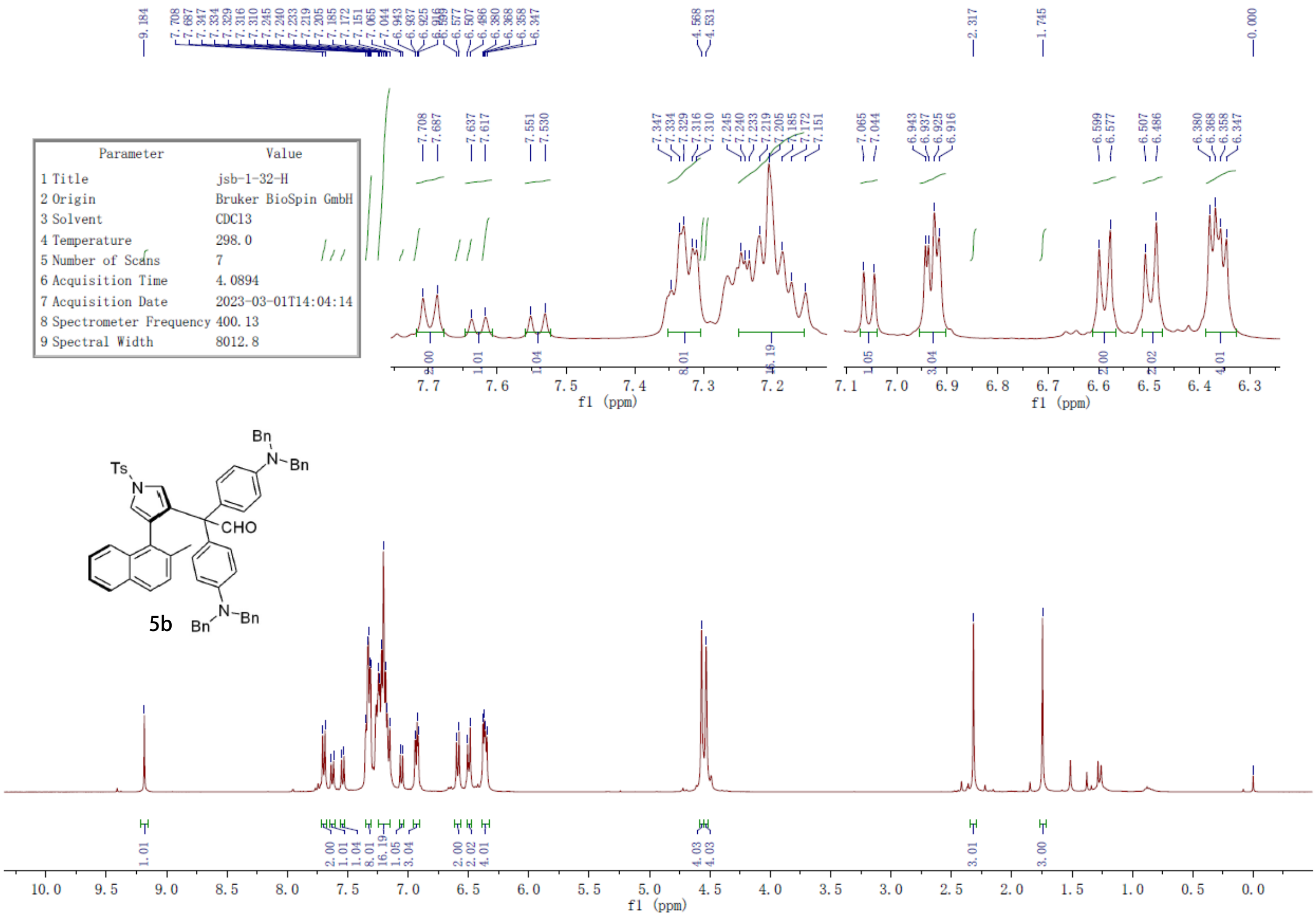

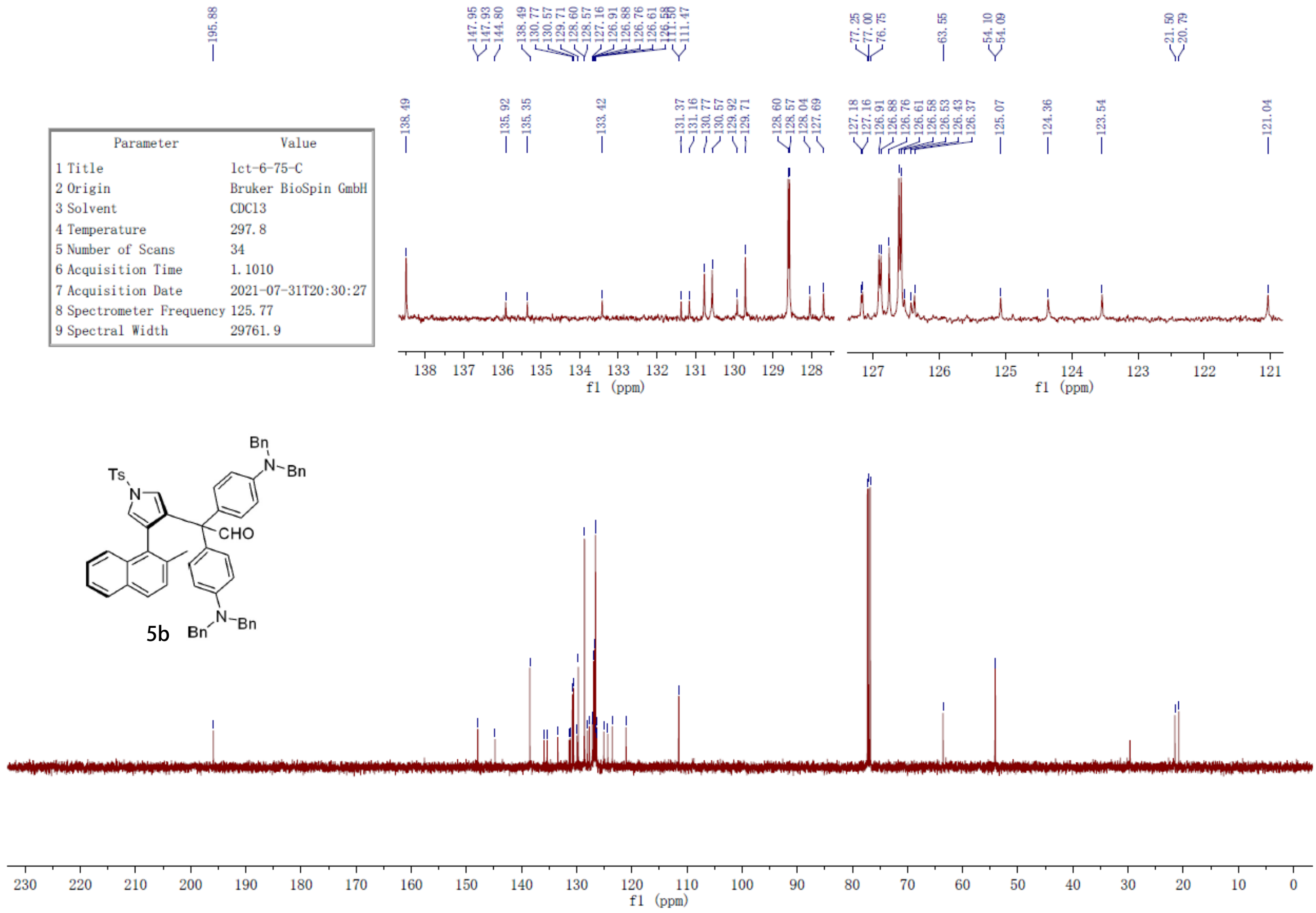

Supplementary Figure 69. ^{1}H and ^{13}C NMR spectra for **5b**

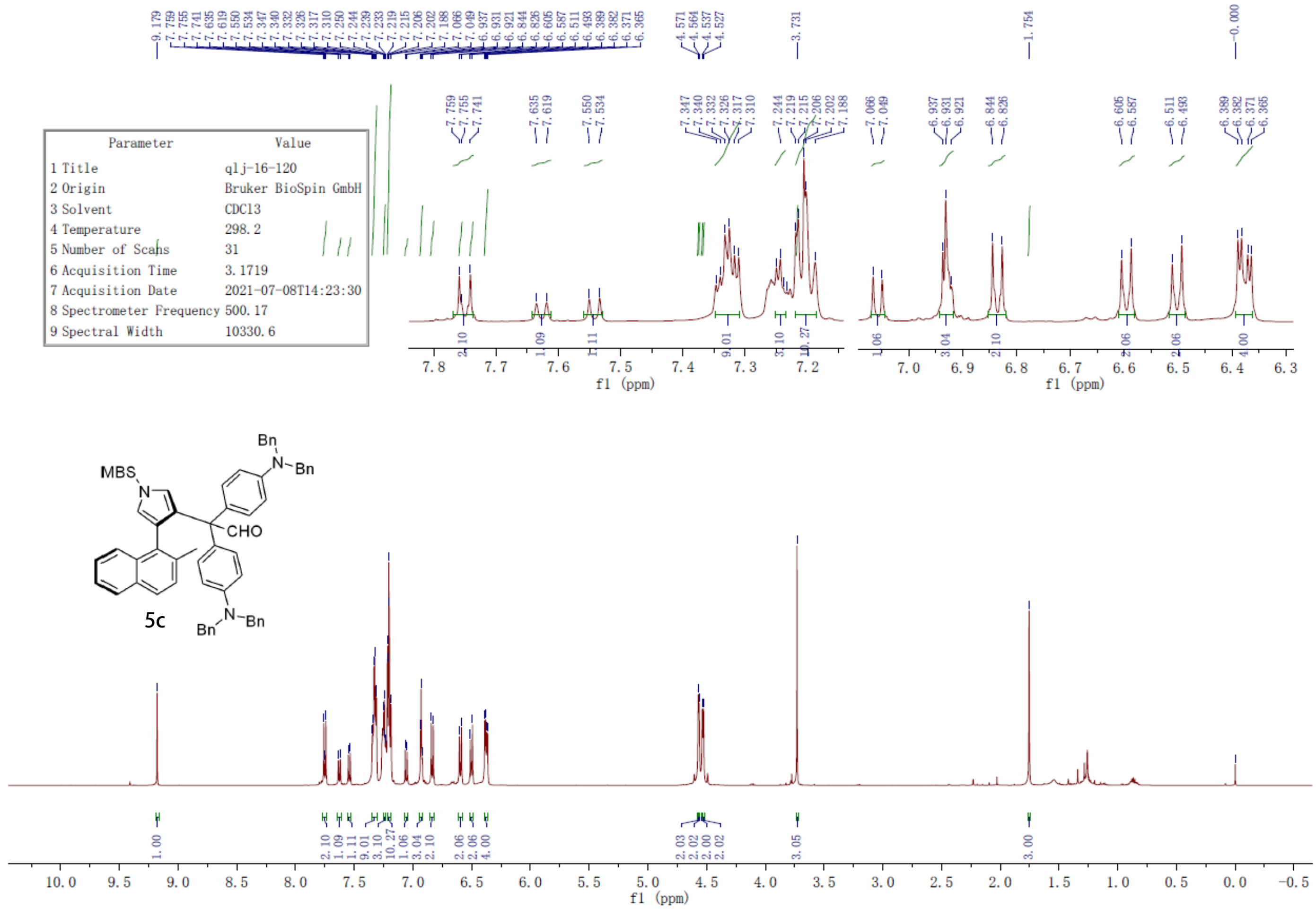

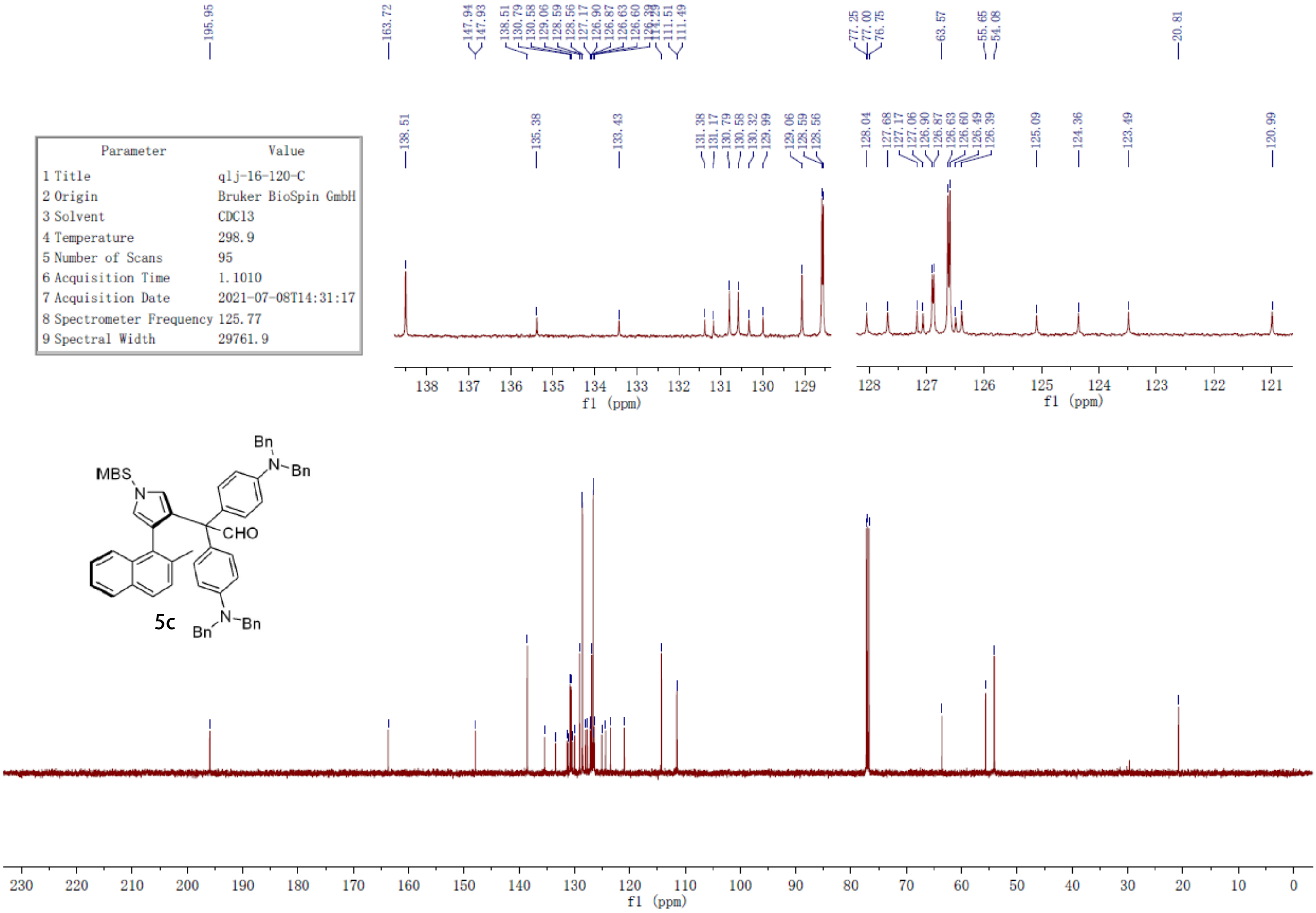

Supplementary Figure 70. ^{1}H and ^{13}C NMR spectra for **5c**

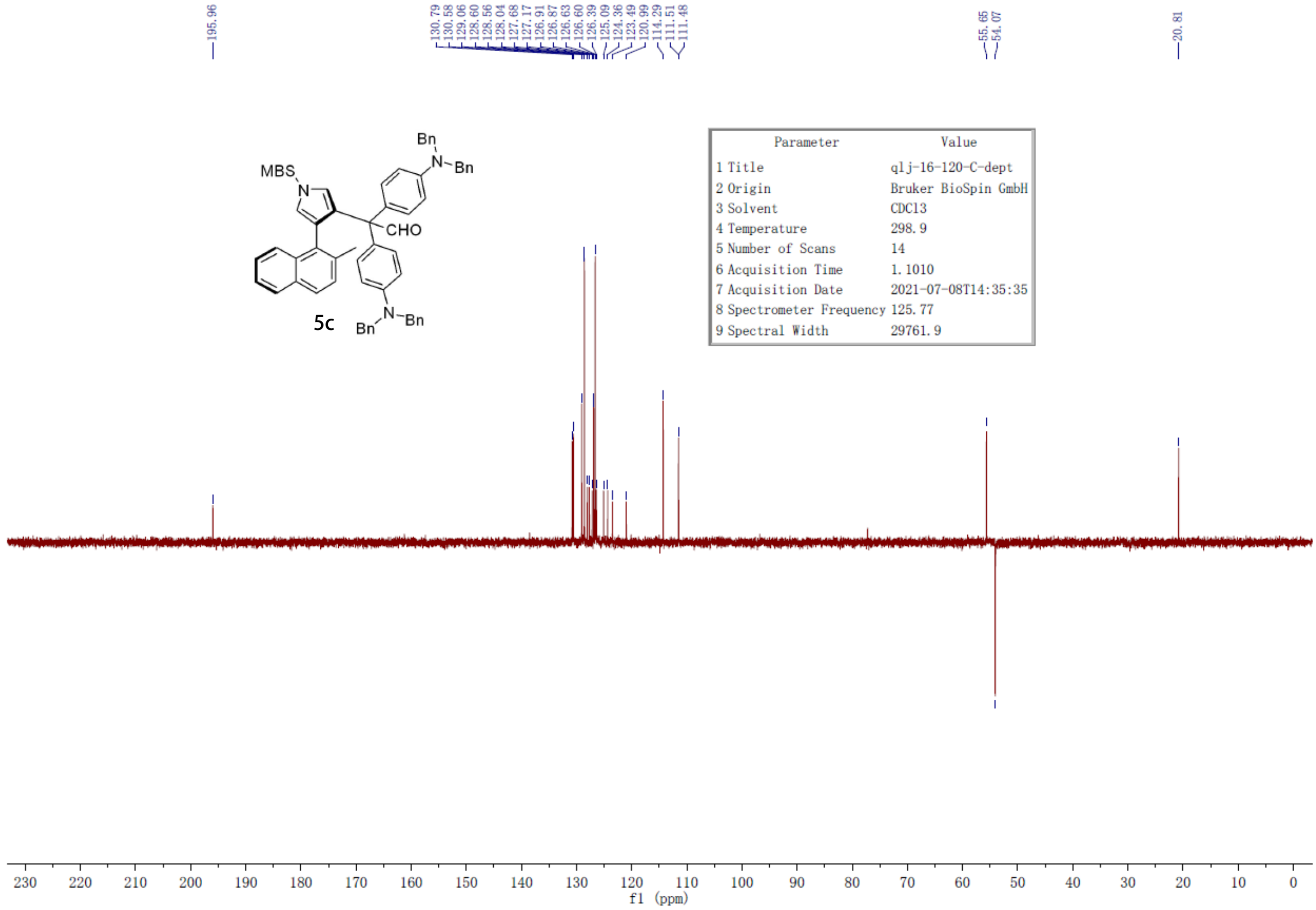

Supplementary Figure 71. DEPT 135 NMR spectra for **5c**

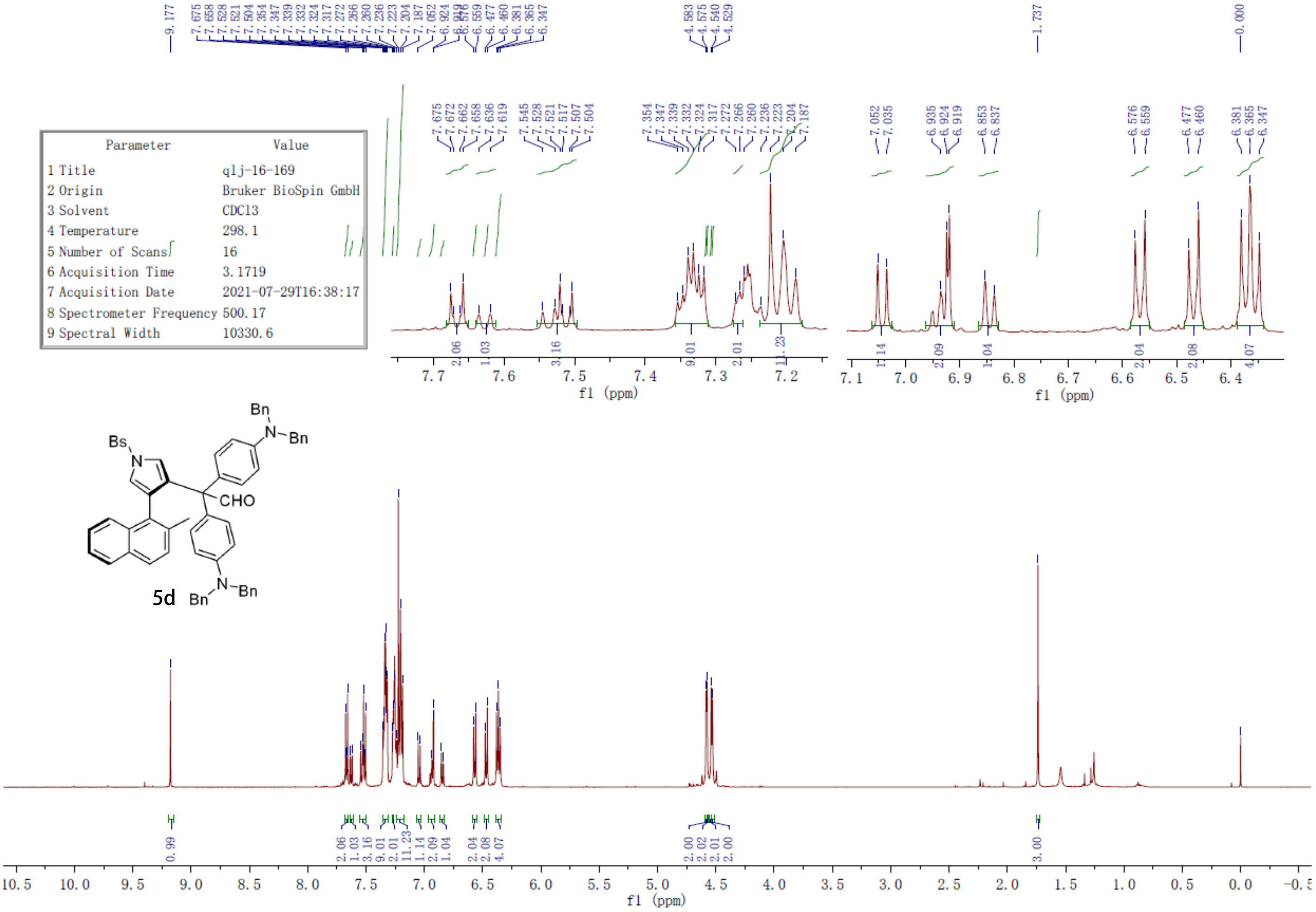

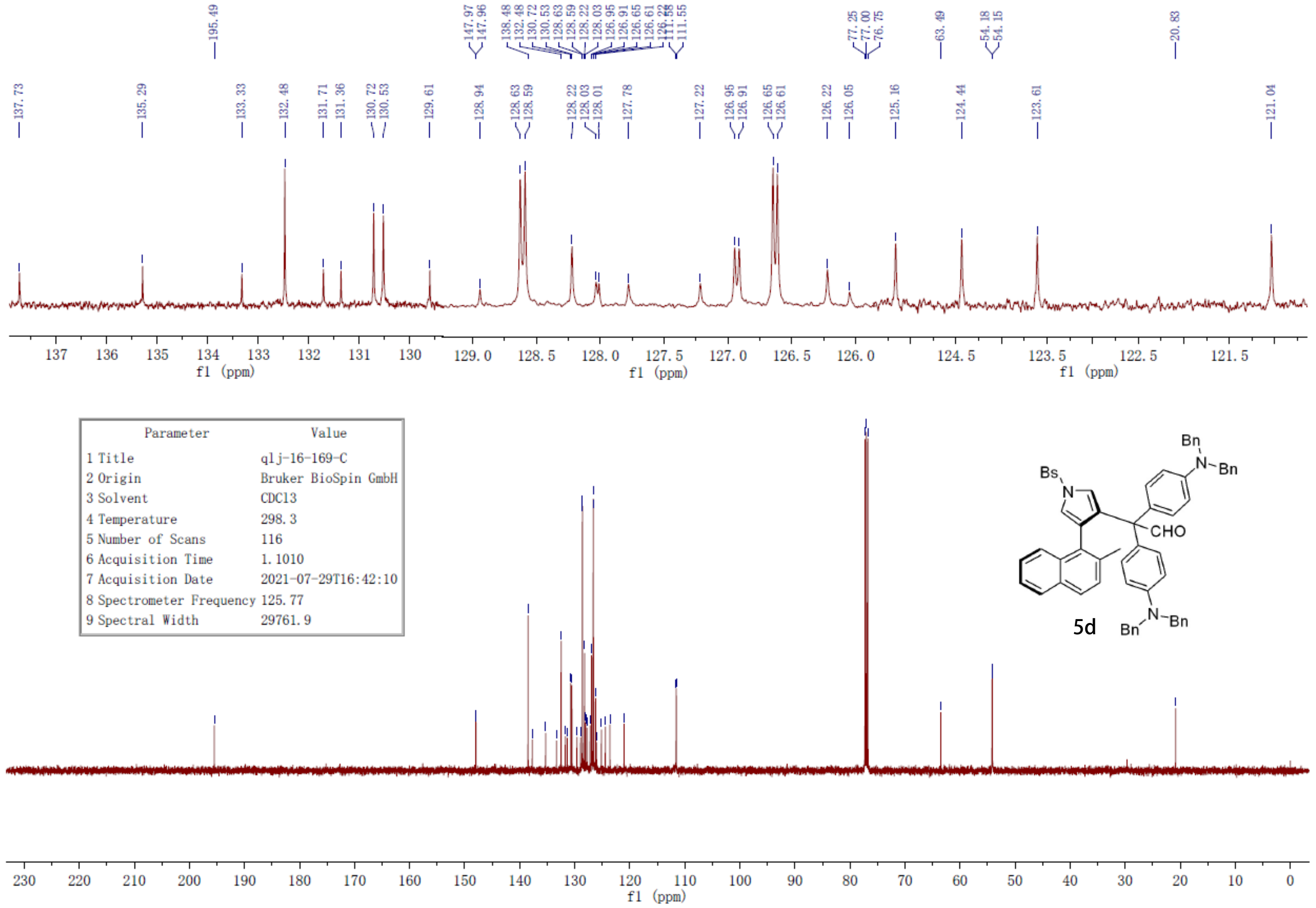

Supplementary Figure 72. ^{1}H and ^{13}C NMR spectra for **5d**

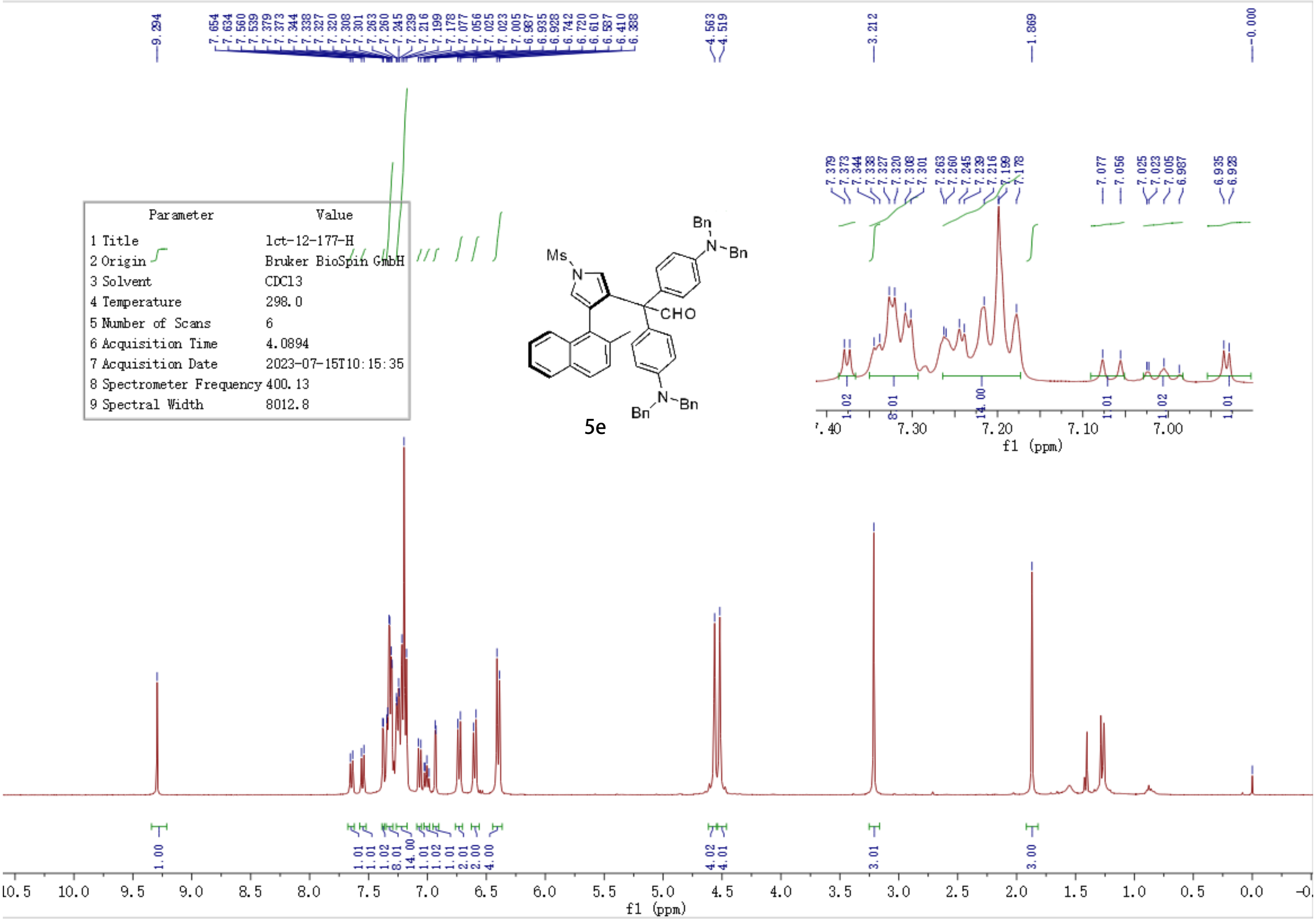

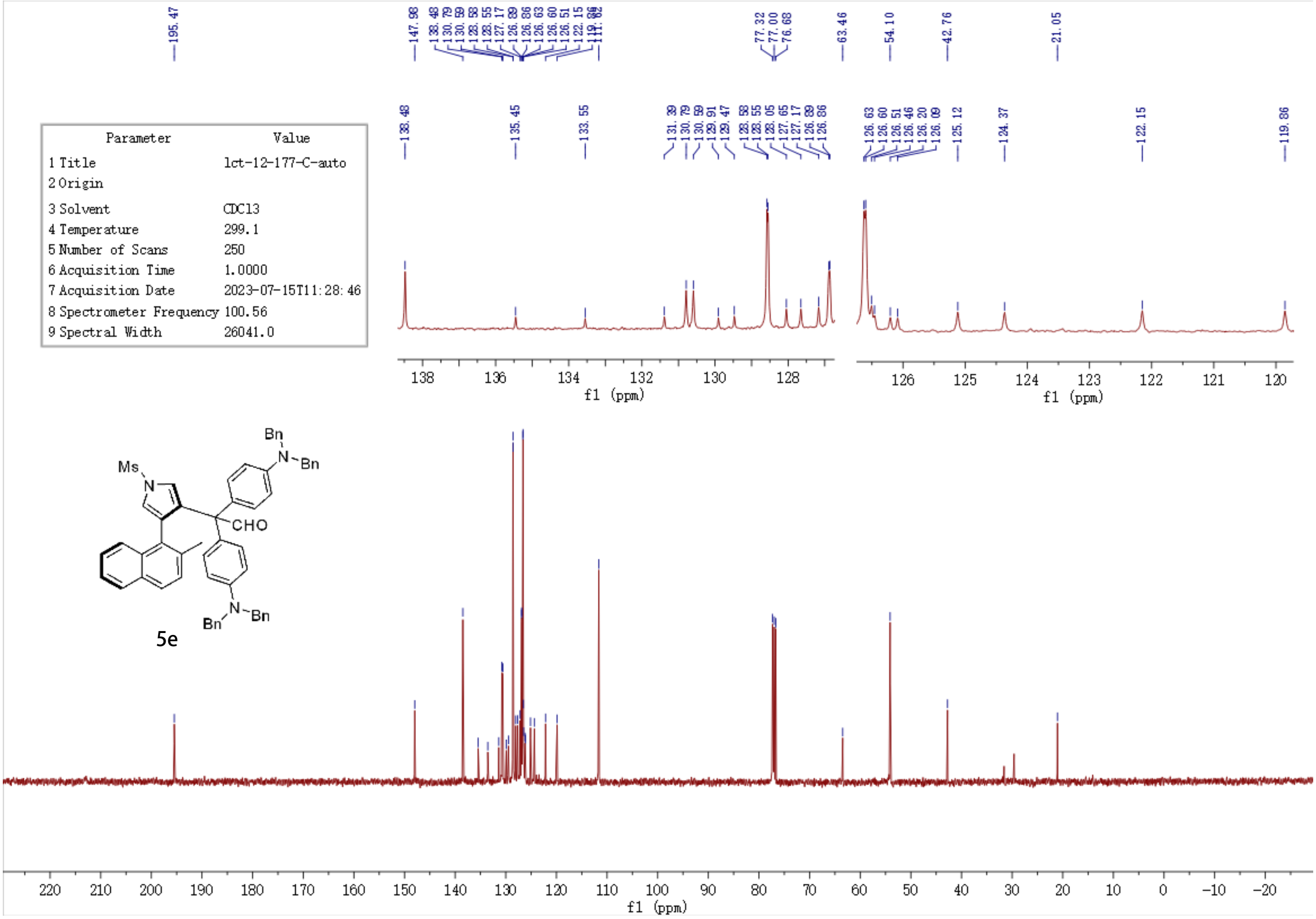

Supplementary Figure 73. ^{1}H and ^{13}C NMR spectra for **5e**

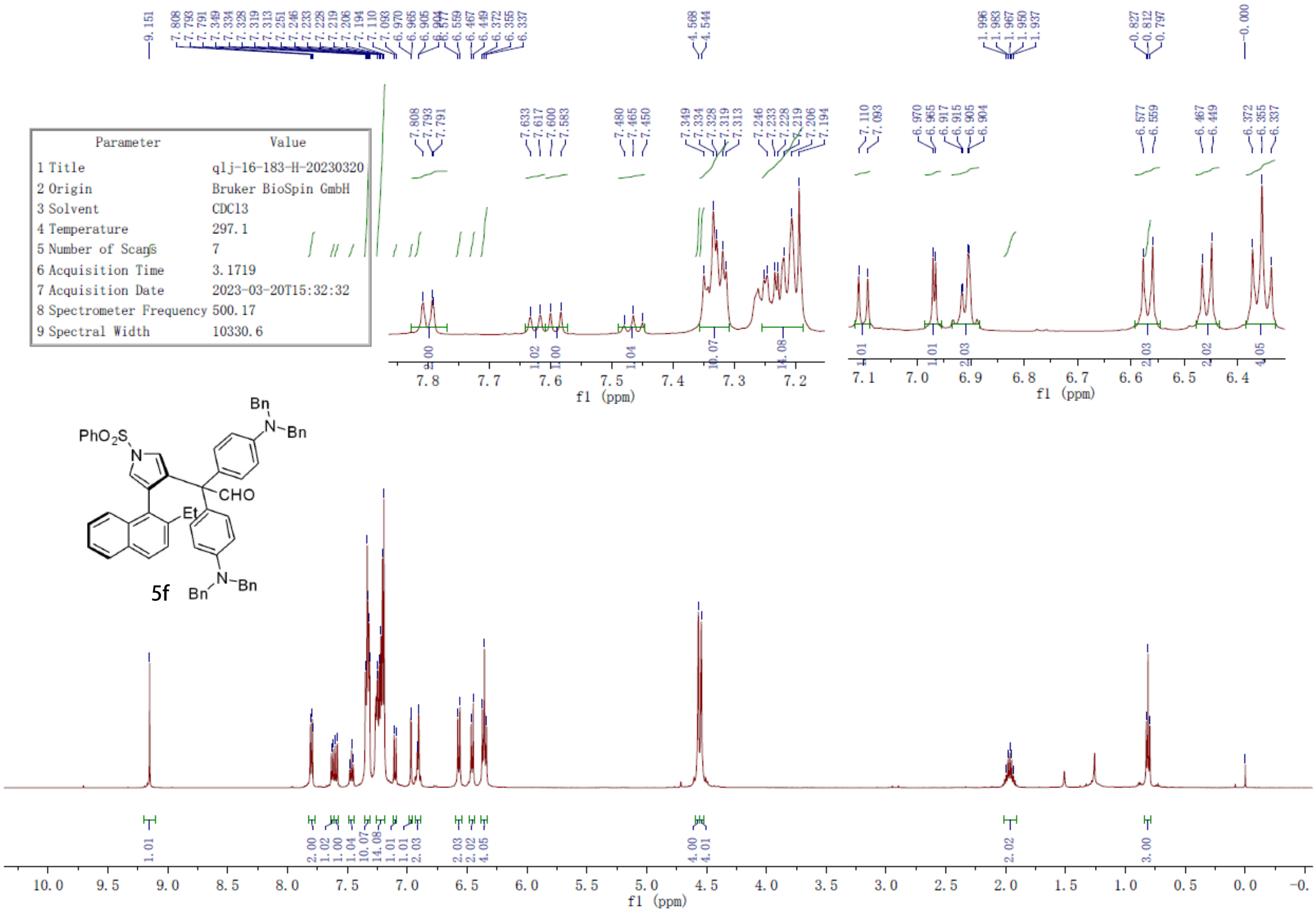

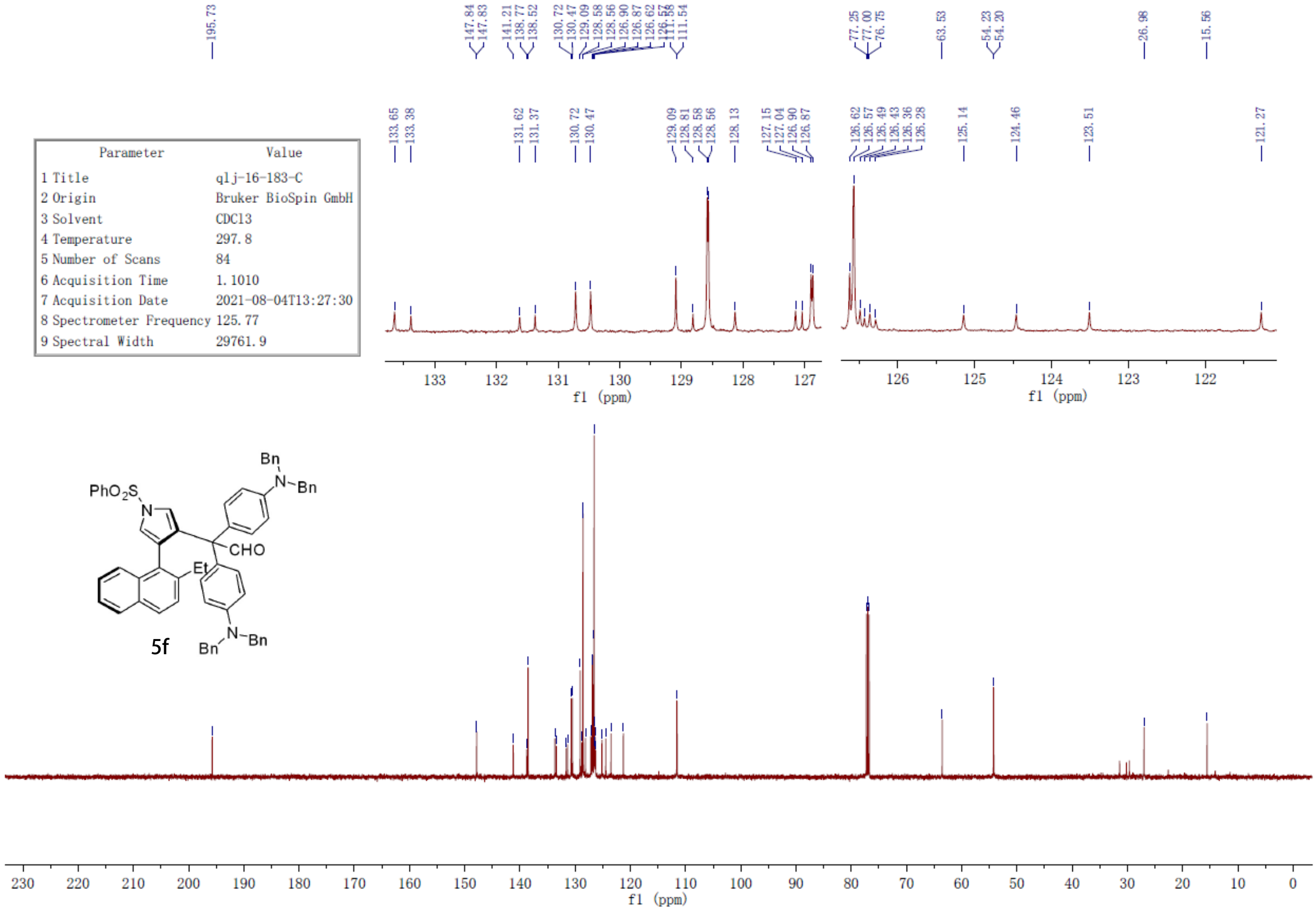

Supplementary Figure 74. ^{1}H and ^{13}C NMR spectra for **5f**

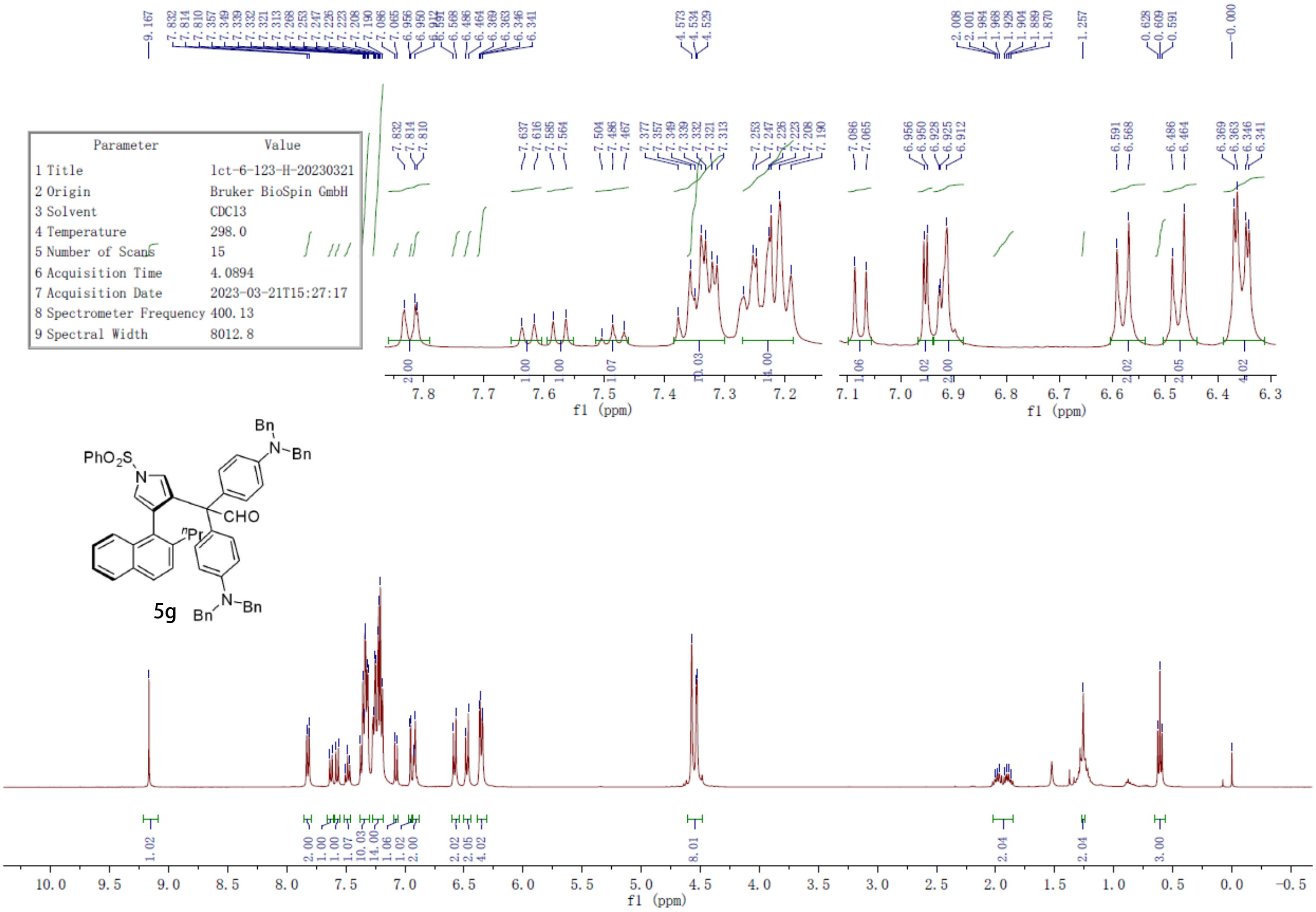

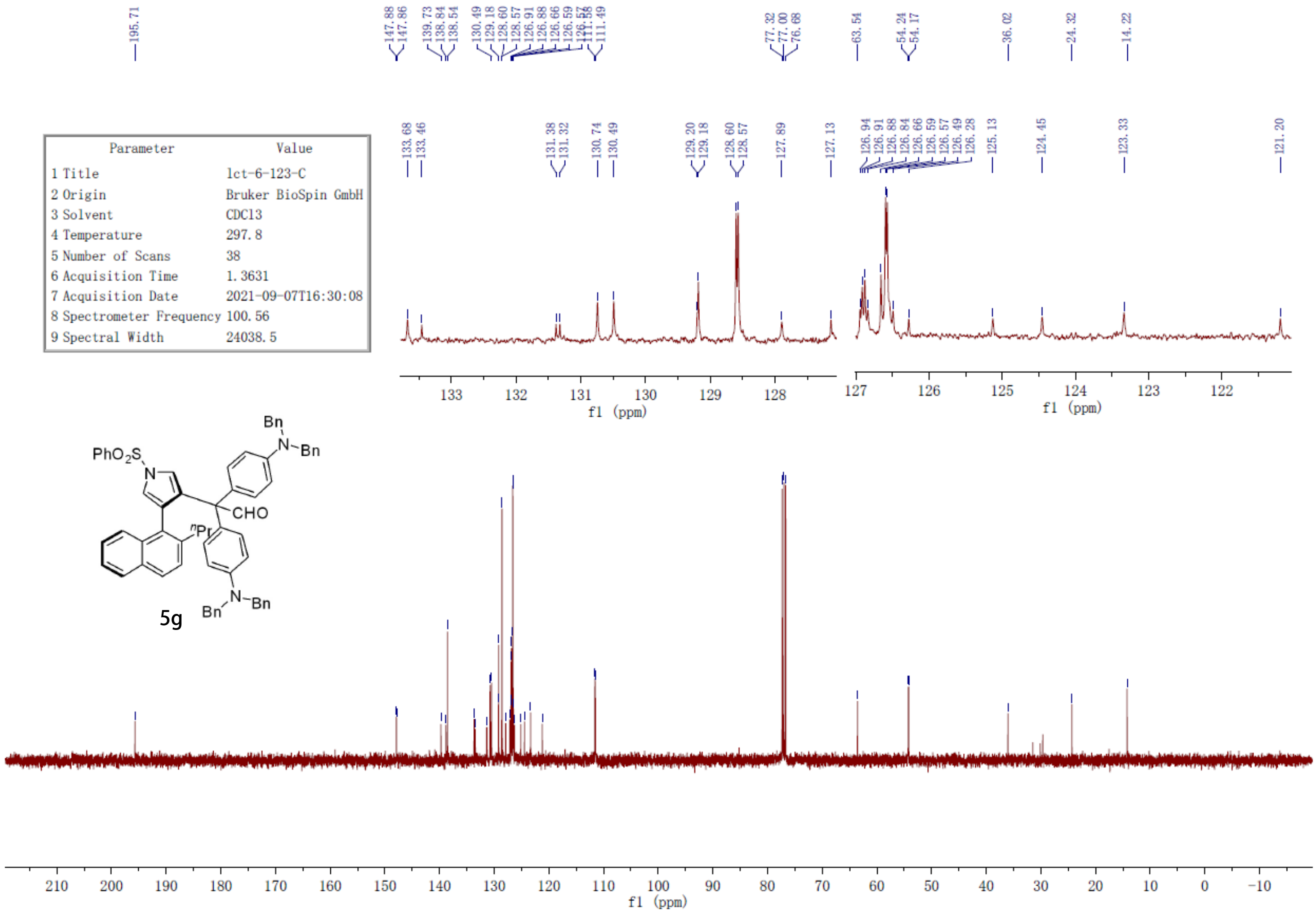

Supplementary Figure 75. ^{1}H and ^{13}C NMR spectra for **5g**

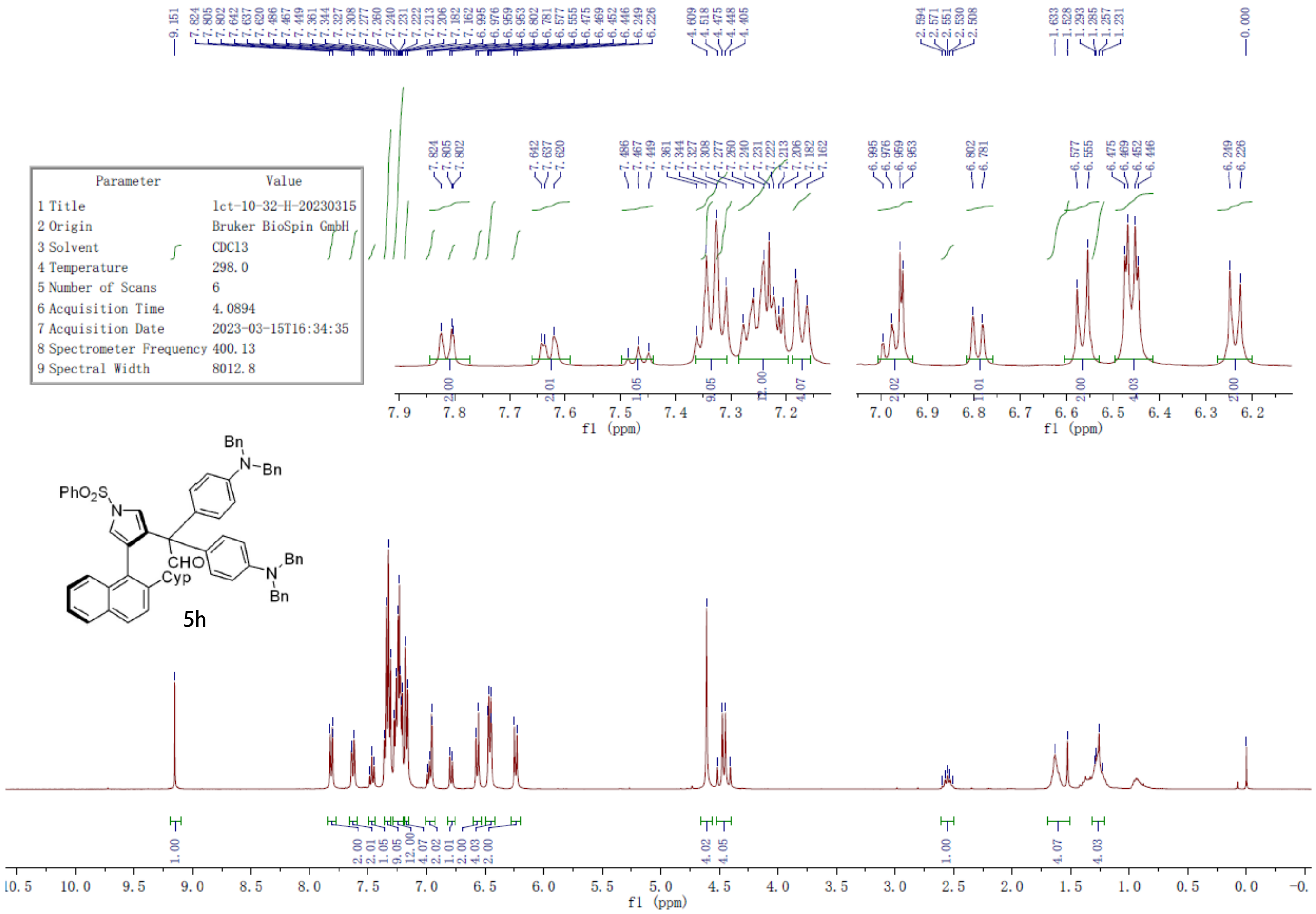

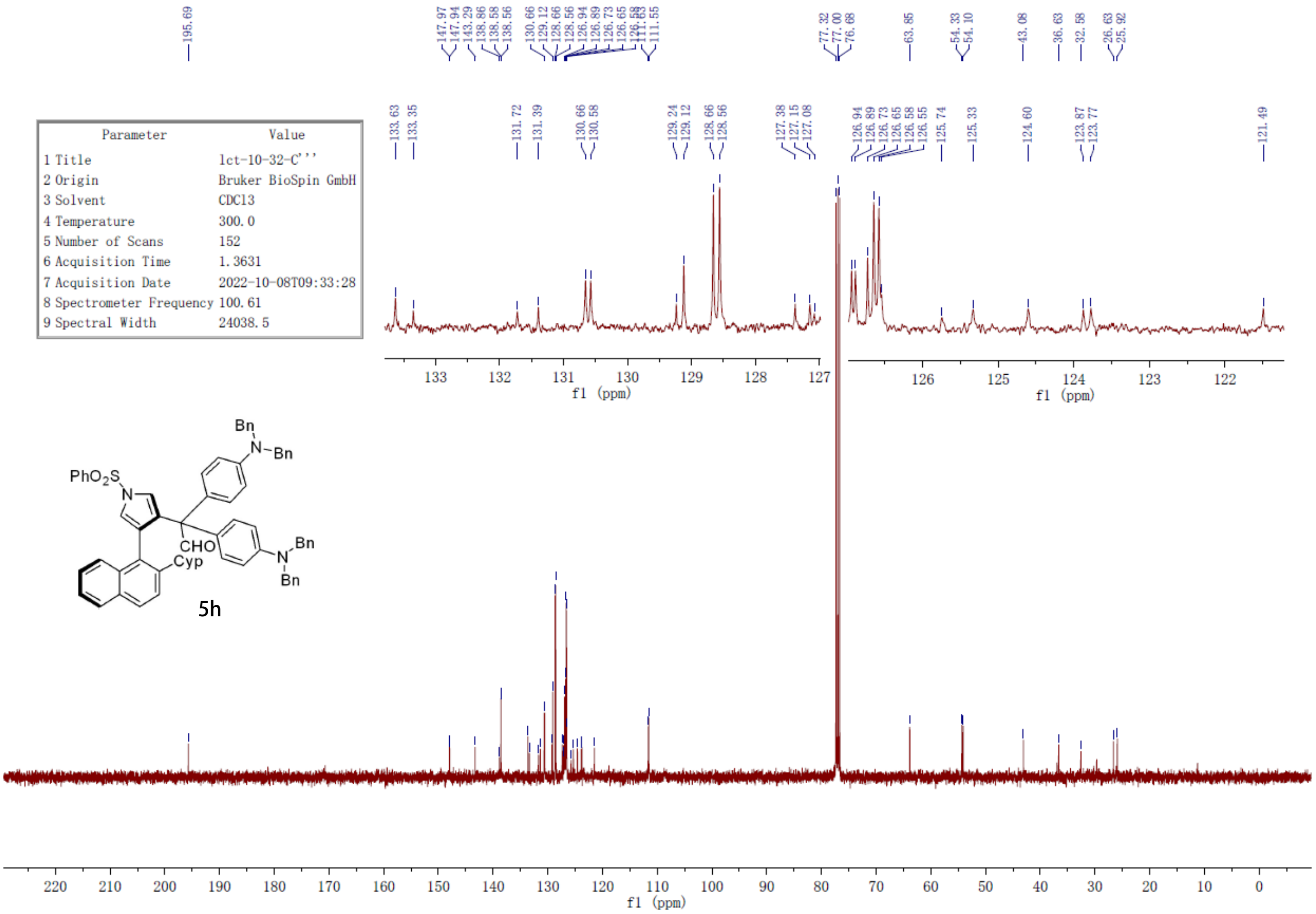

Supplementary Figure 76. ^{1}H and ^{13}C NMR spectra for **5h**

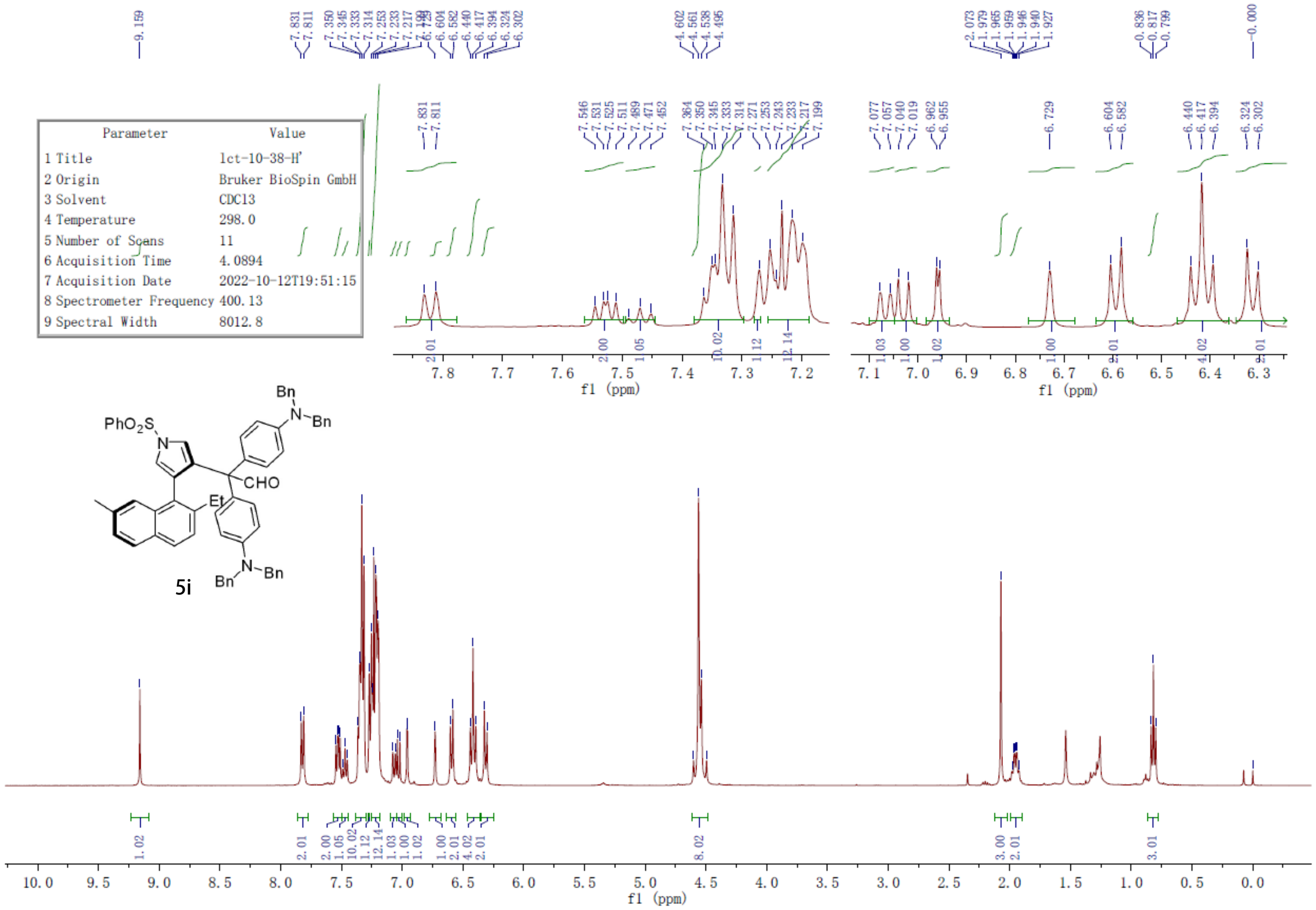

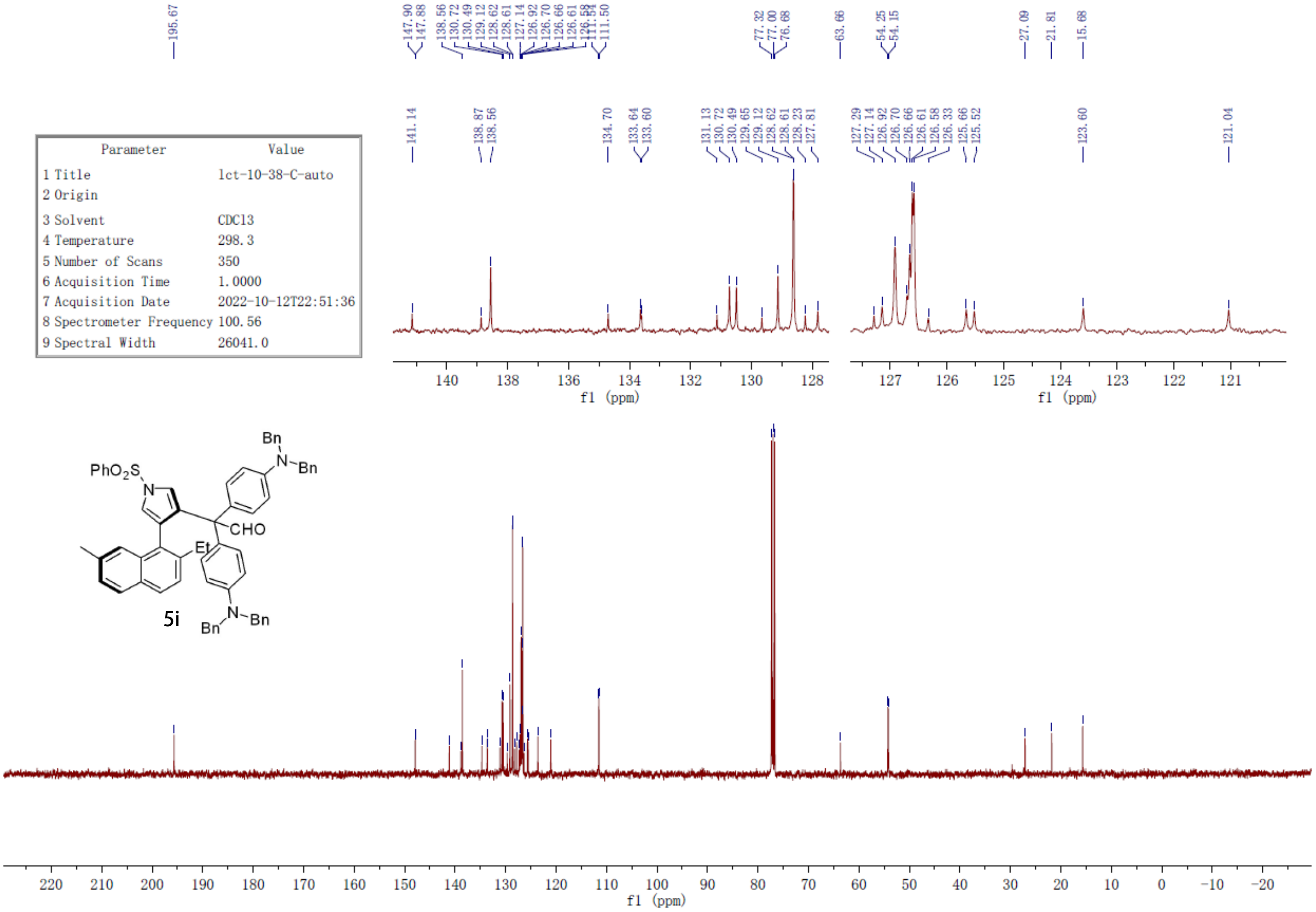

Supplementary Figure 77. ^{1}H and ^{13}C NMR spectra for **5i**

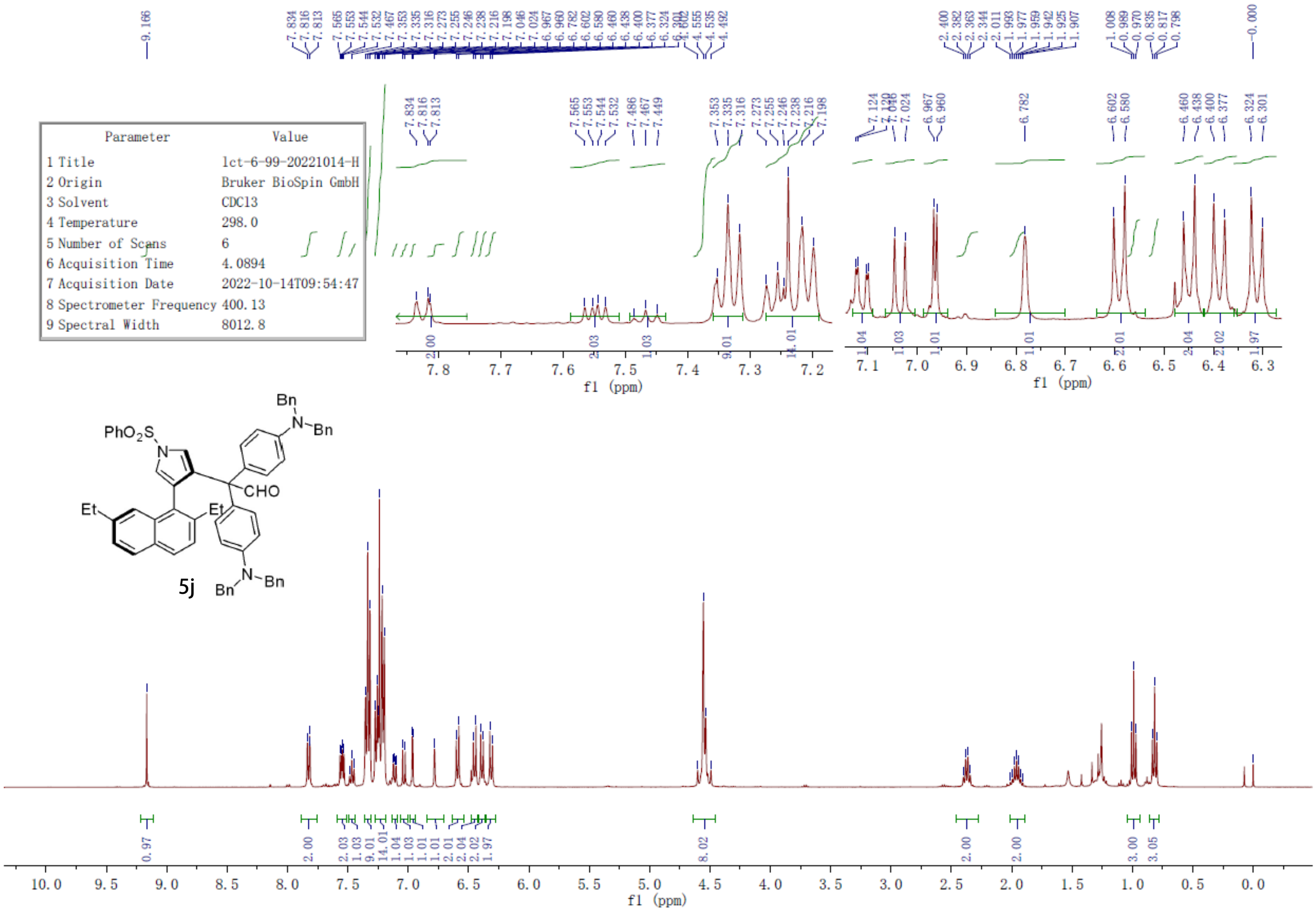

Parameter	Value
1 Title	lct-6-99-20221014-H
2 Origin	Bruker BioSpin GmbH
3 Solvent	CDCl3
4 Temperature	298.0
5 Number of Scans	6
6 Acquisition Time	4.0894
7 Acquisition Date	2022-10-14T09:54:47
8 Spectrometer Frequency	400.13
9 Spectral Width	8012.8

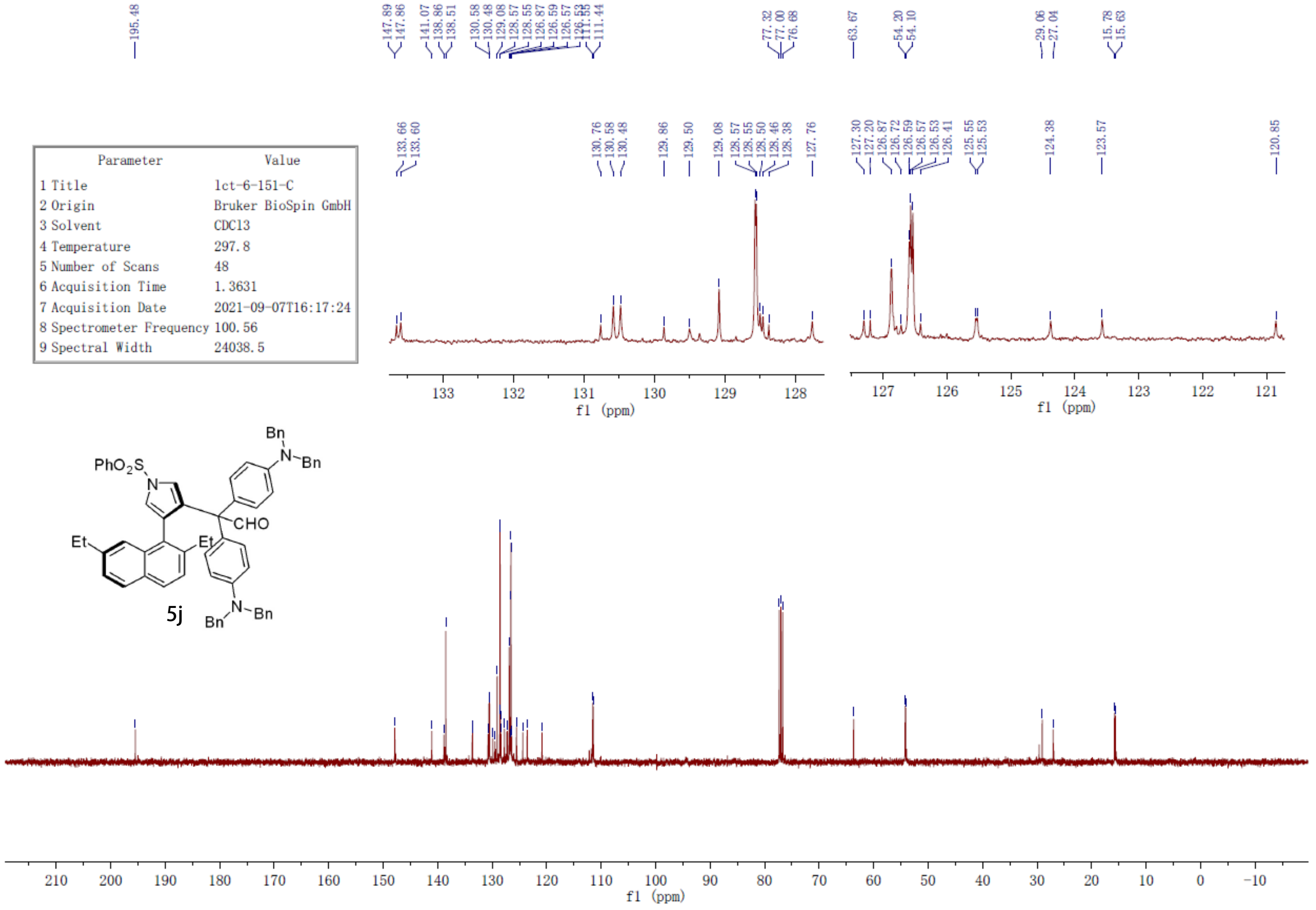

Parameter	Value
1 Title	lct-6-151-C
2 Origin	Bruker BioSpin GmbH
3 Solvent	CDCl3
4 Temperature	297.8
5 Number of Scans	48
6 Acquisition Time	1.3631
7 Acquisition Date	2021-09-07T16:17:24
8 Spectrometer Frequency	100.56
9 Spectral Width	24038.5

Supplementary Figure 78. ^{1}H and ^{13}C NMR spectra for **5j**

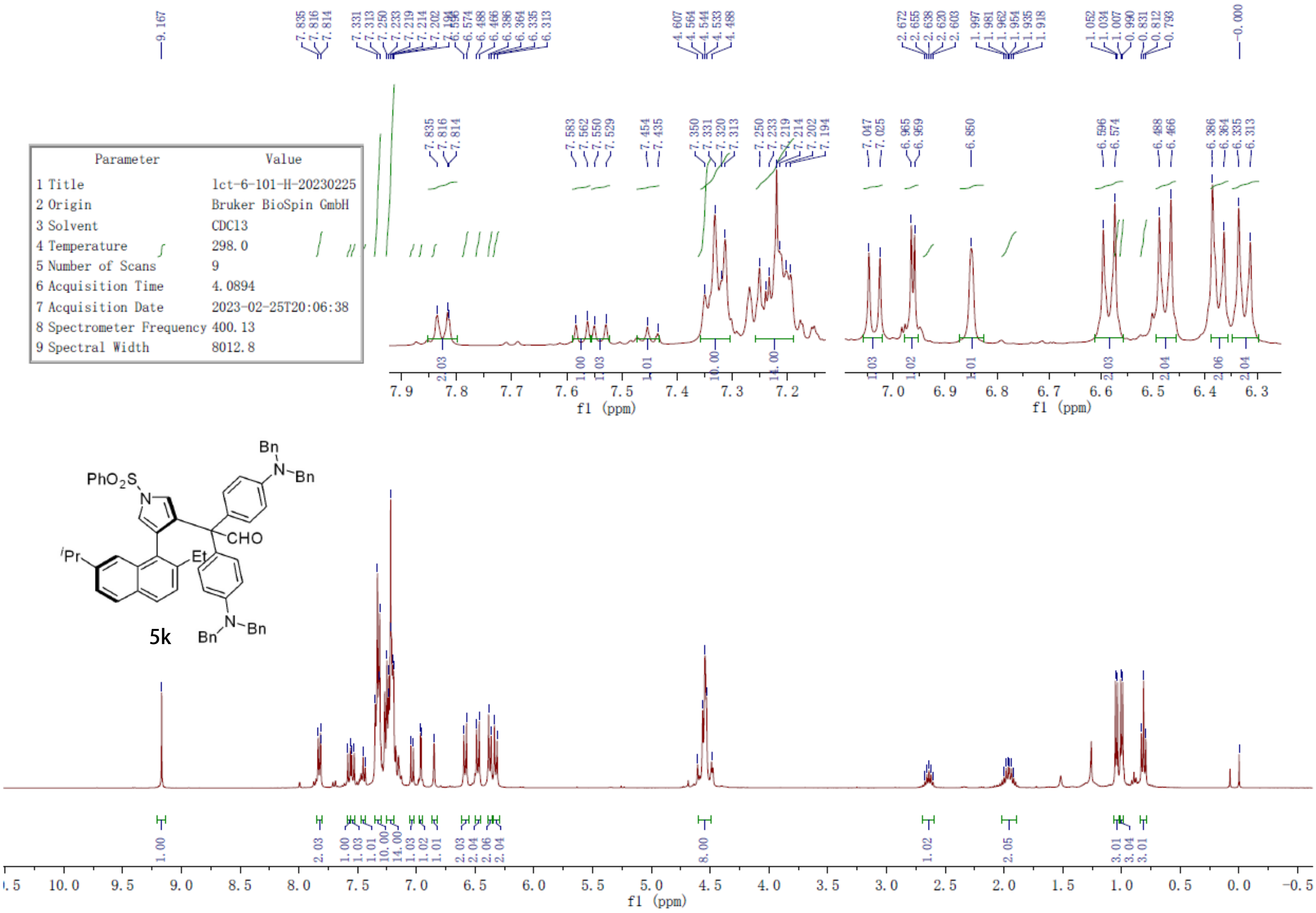

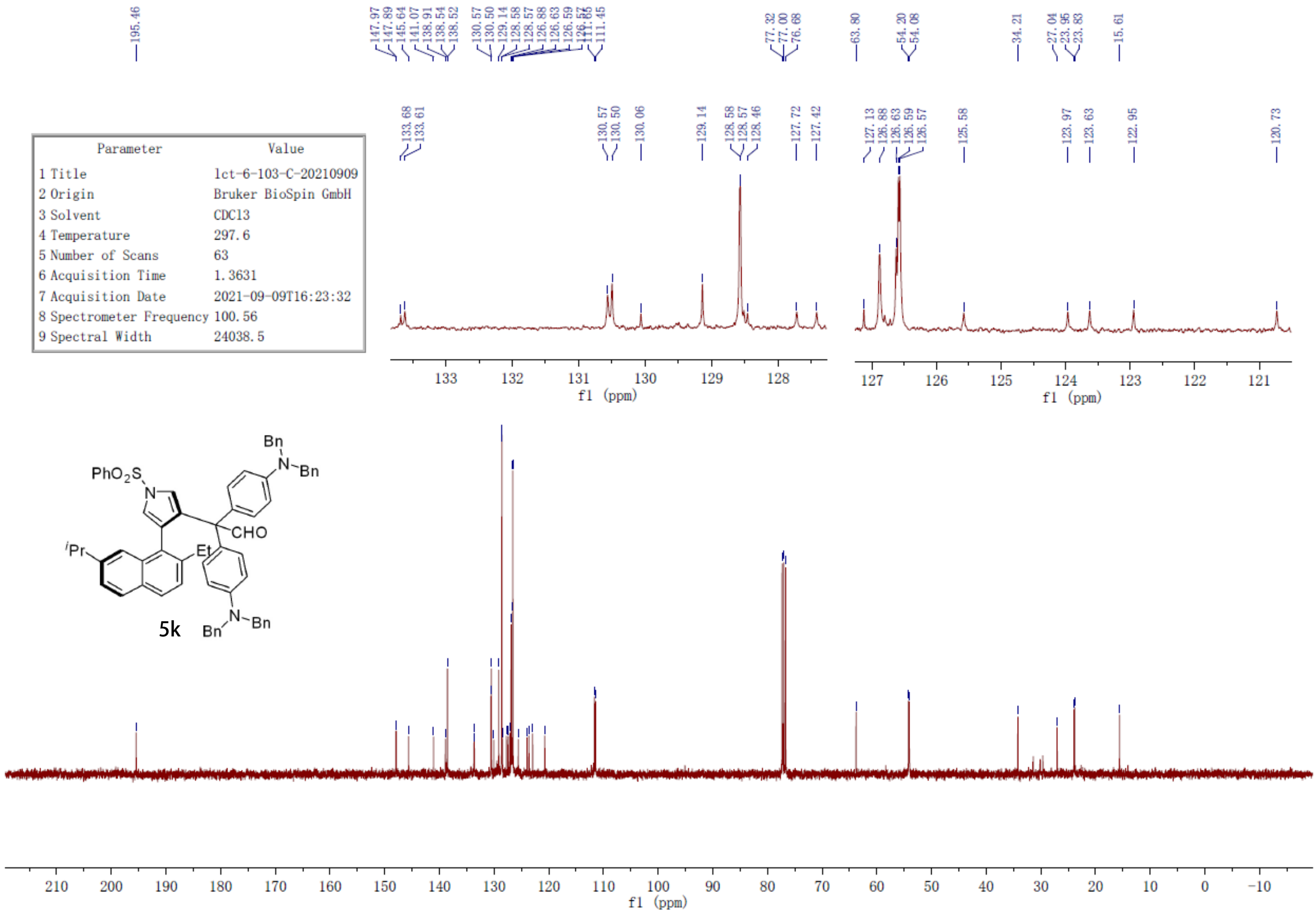

Supplementary Figure 79. ^{1}H and ^{13}C NMR spectra for **5k**

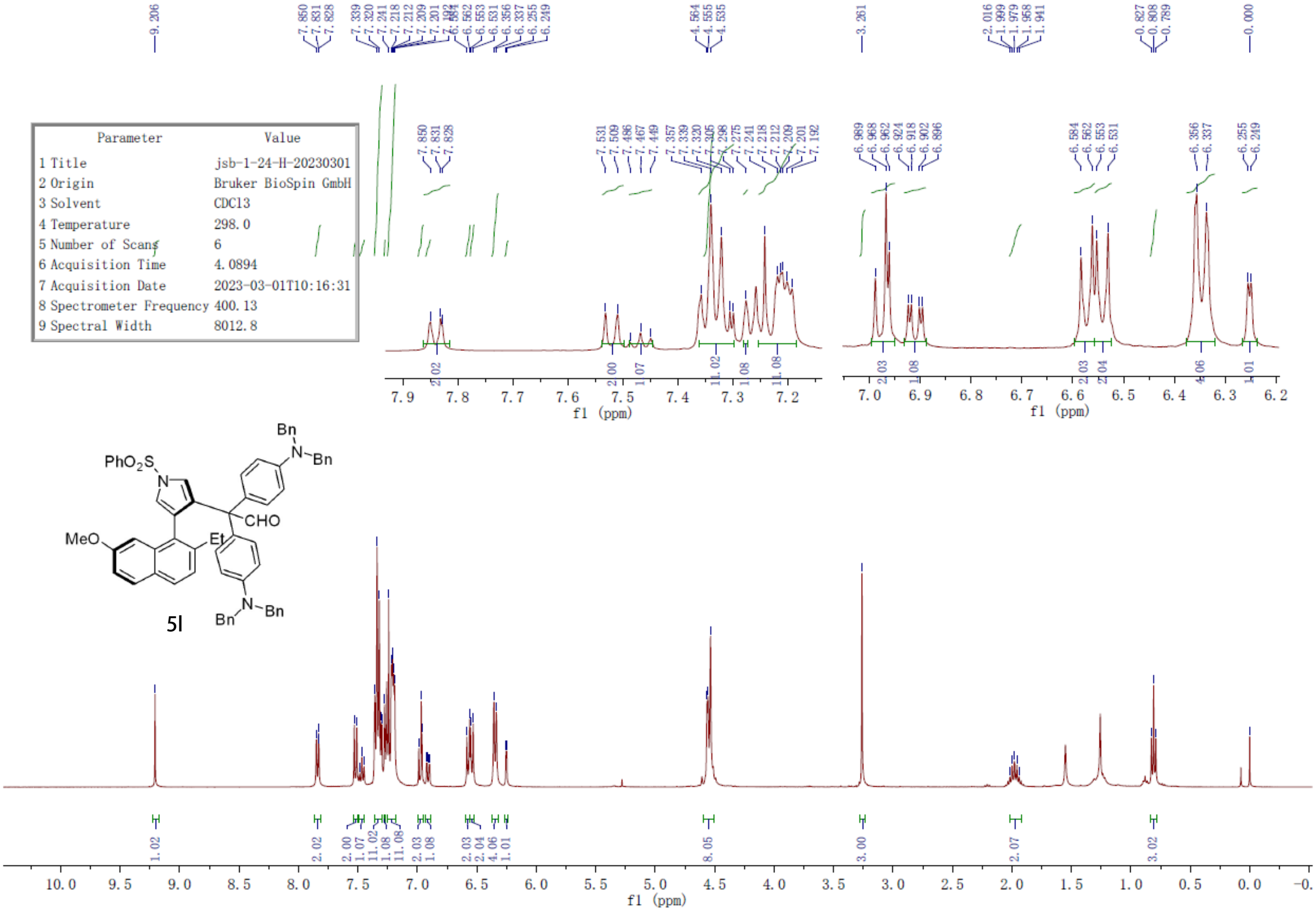

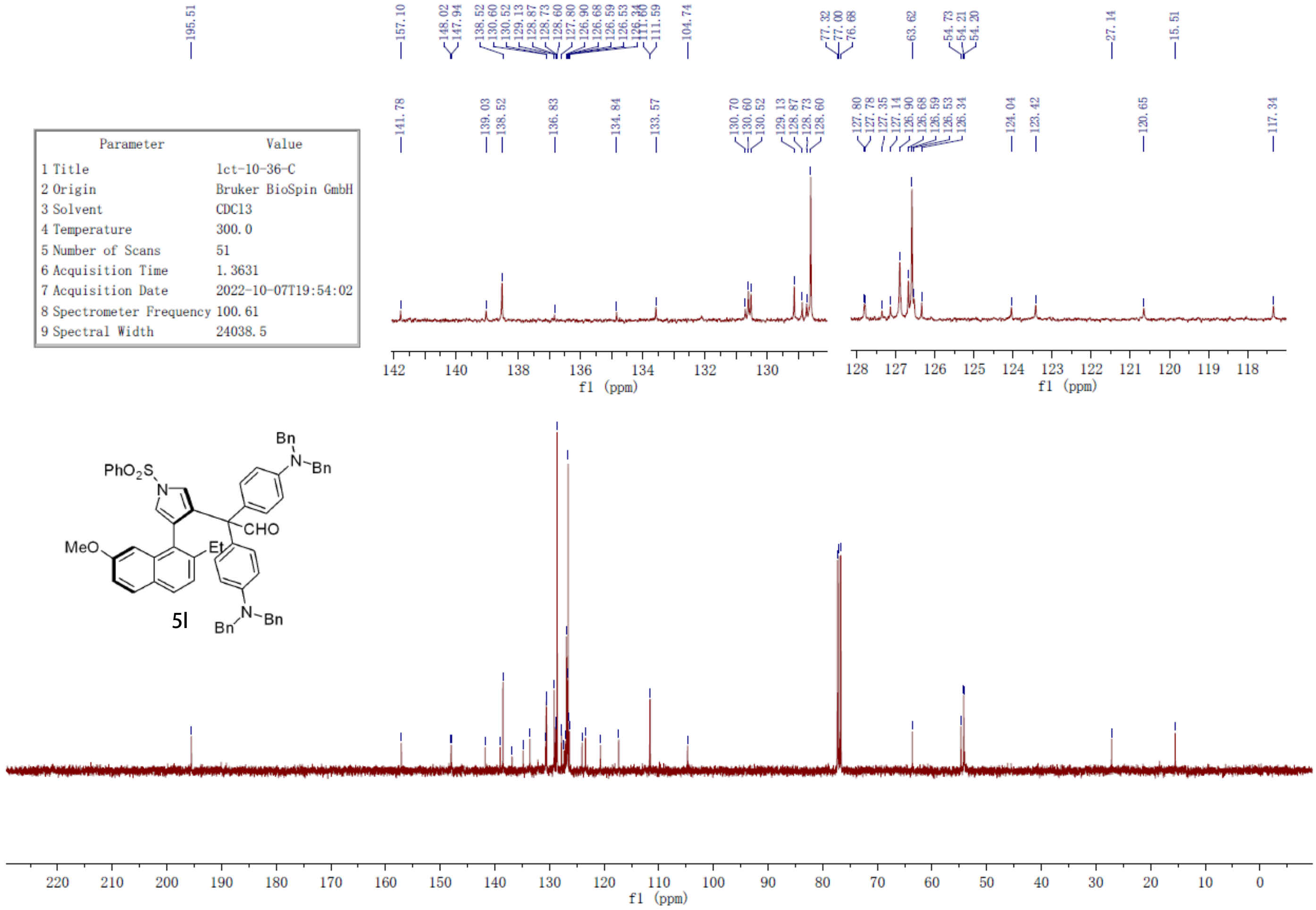

Supplementary Figure 80. ^{1}H and ^{13}C NMR spectra for **5l**

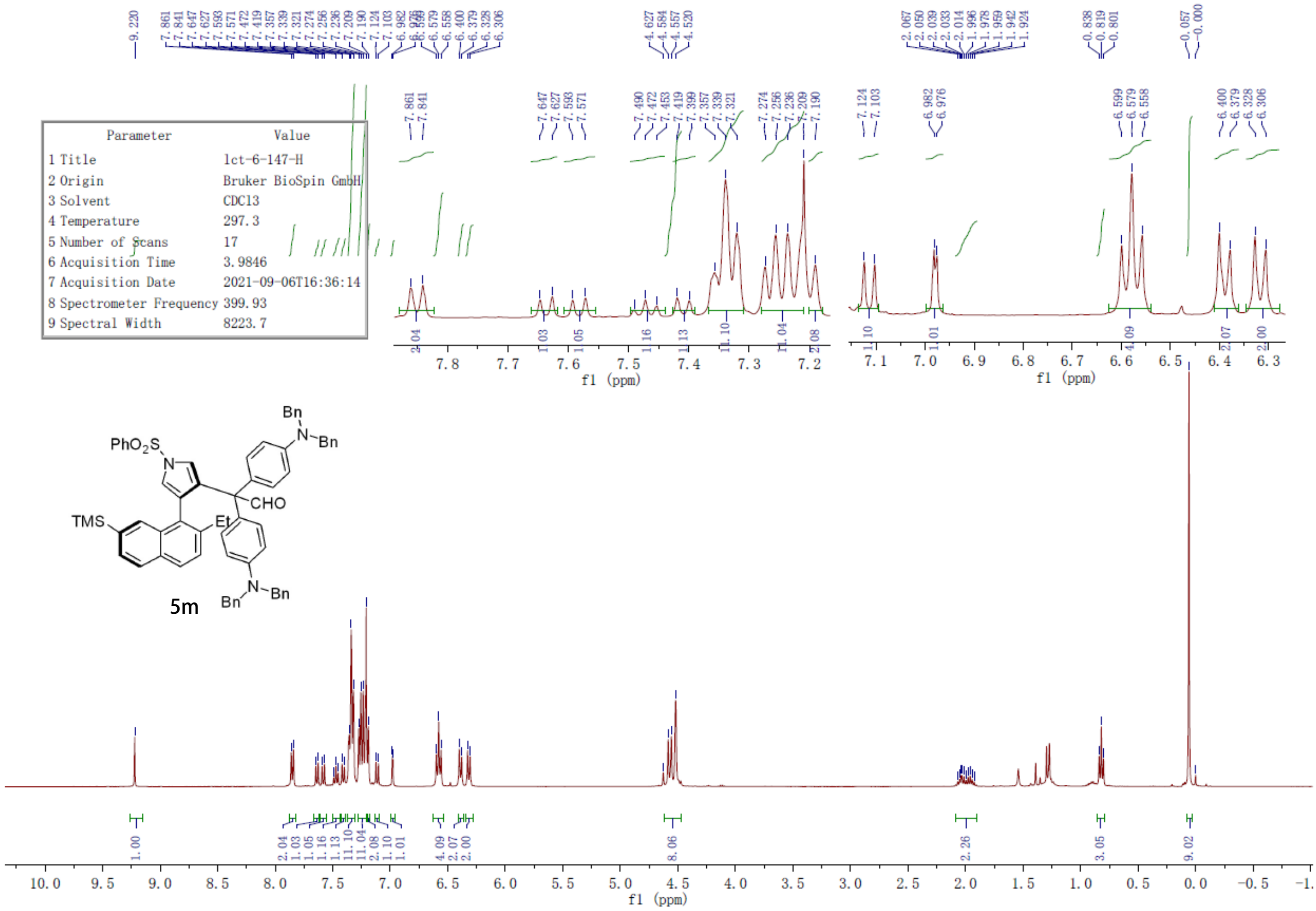

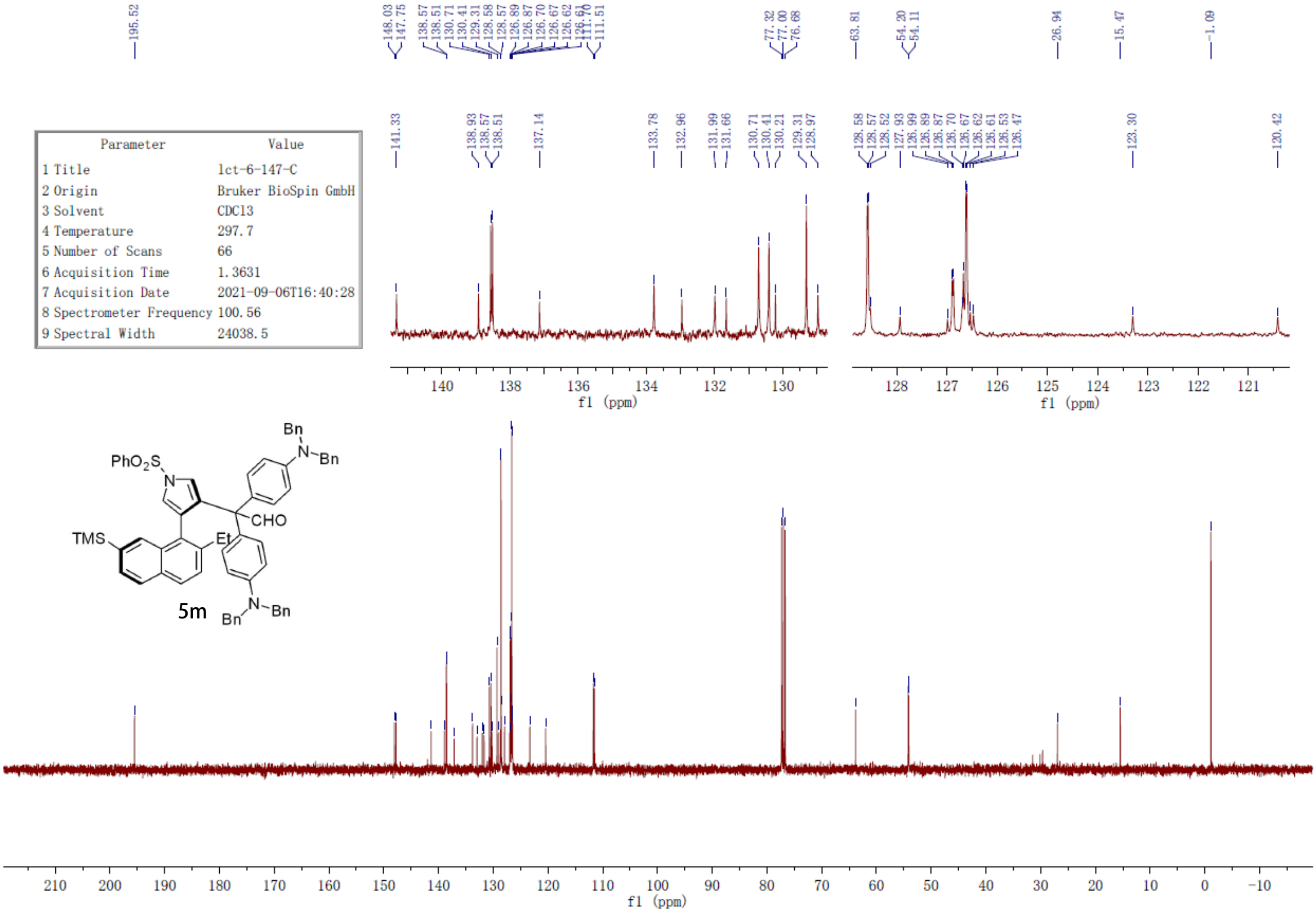

Supplementary Figure 81. ^{1}H and ^{13}C NMR spectra for **5m**

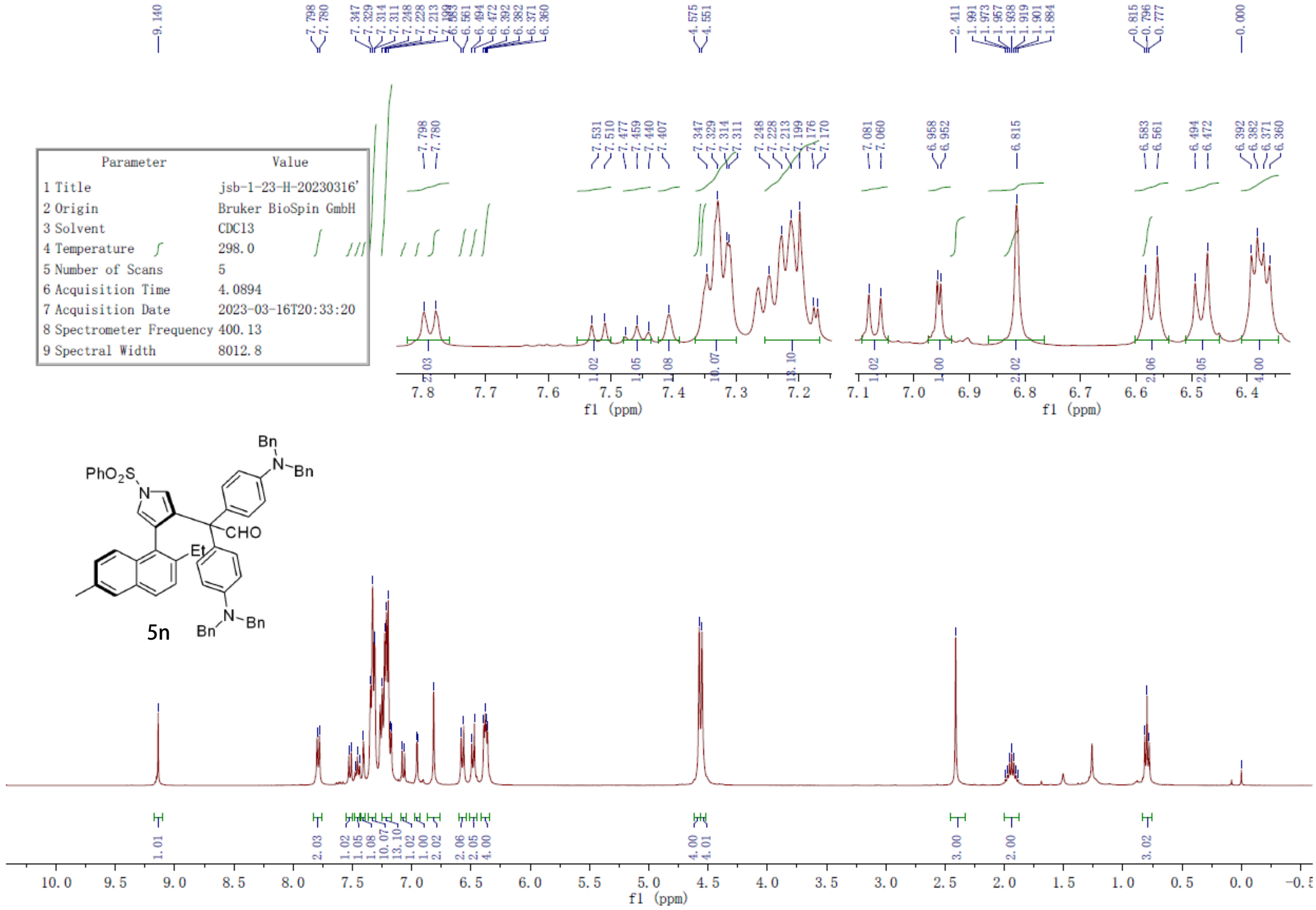

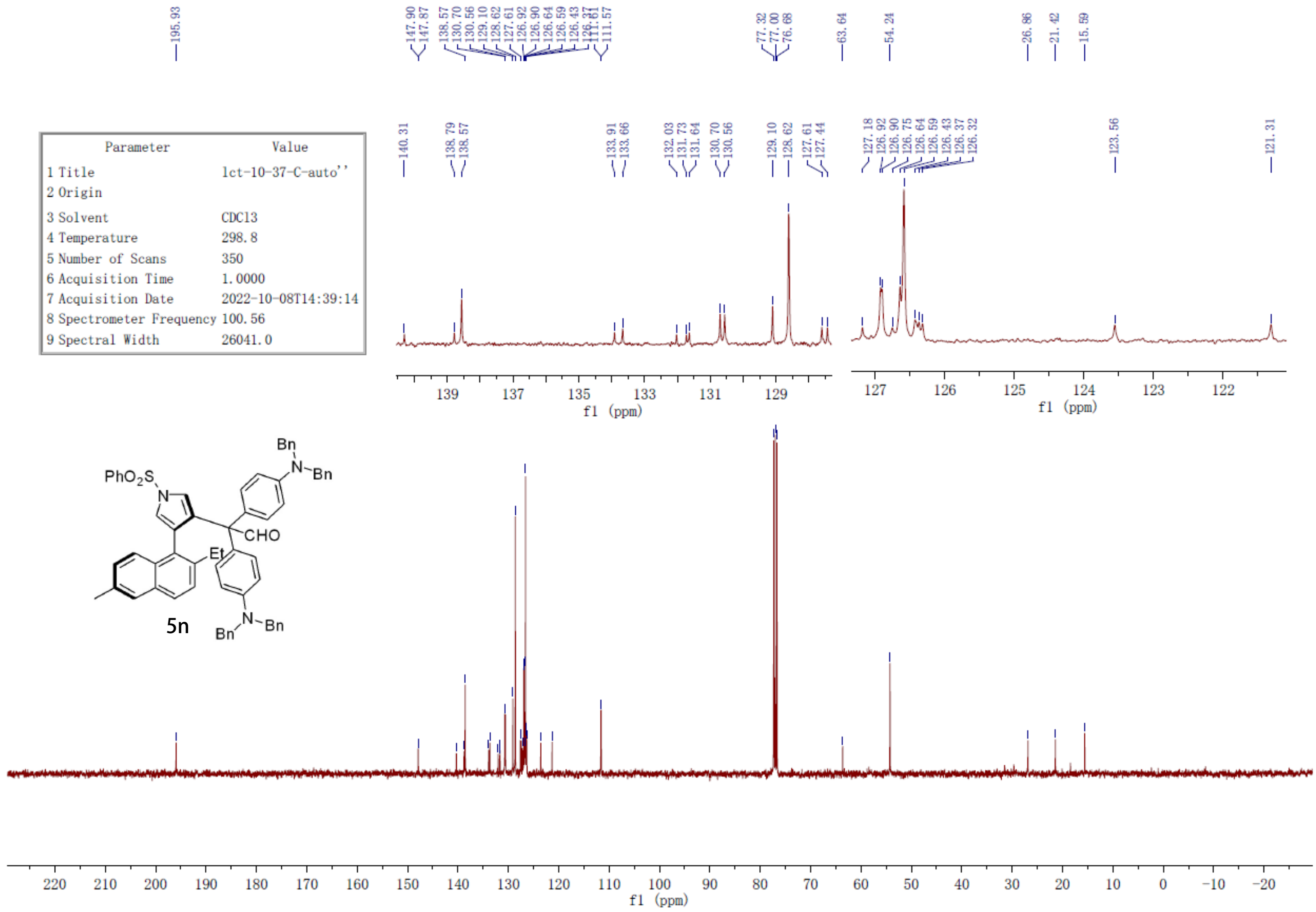

Supplementary Figure 82. ^{1}H and ^{13}C NMR spectra for **5n**

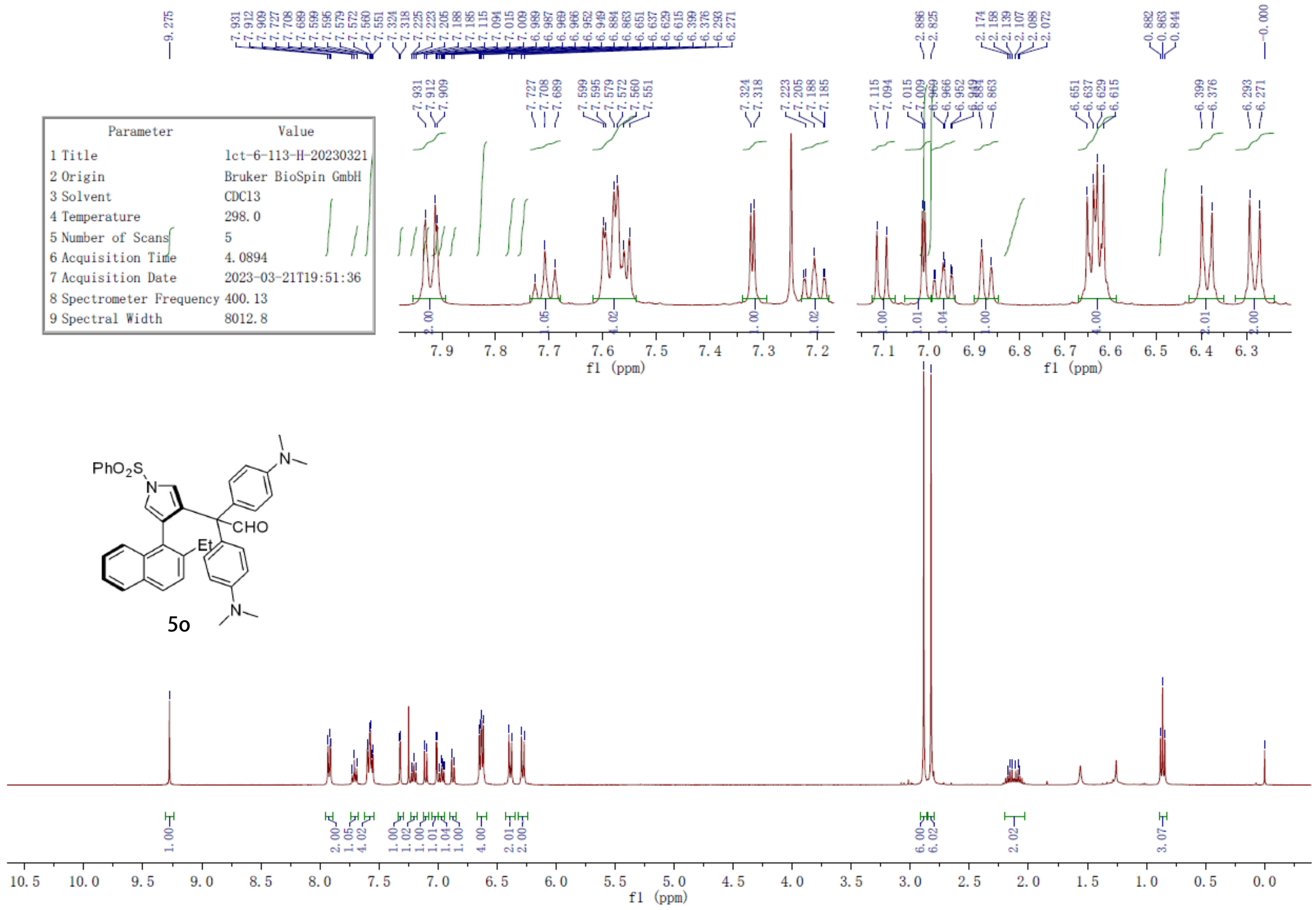

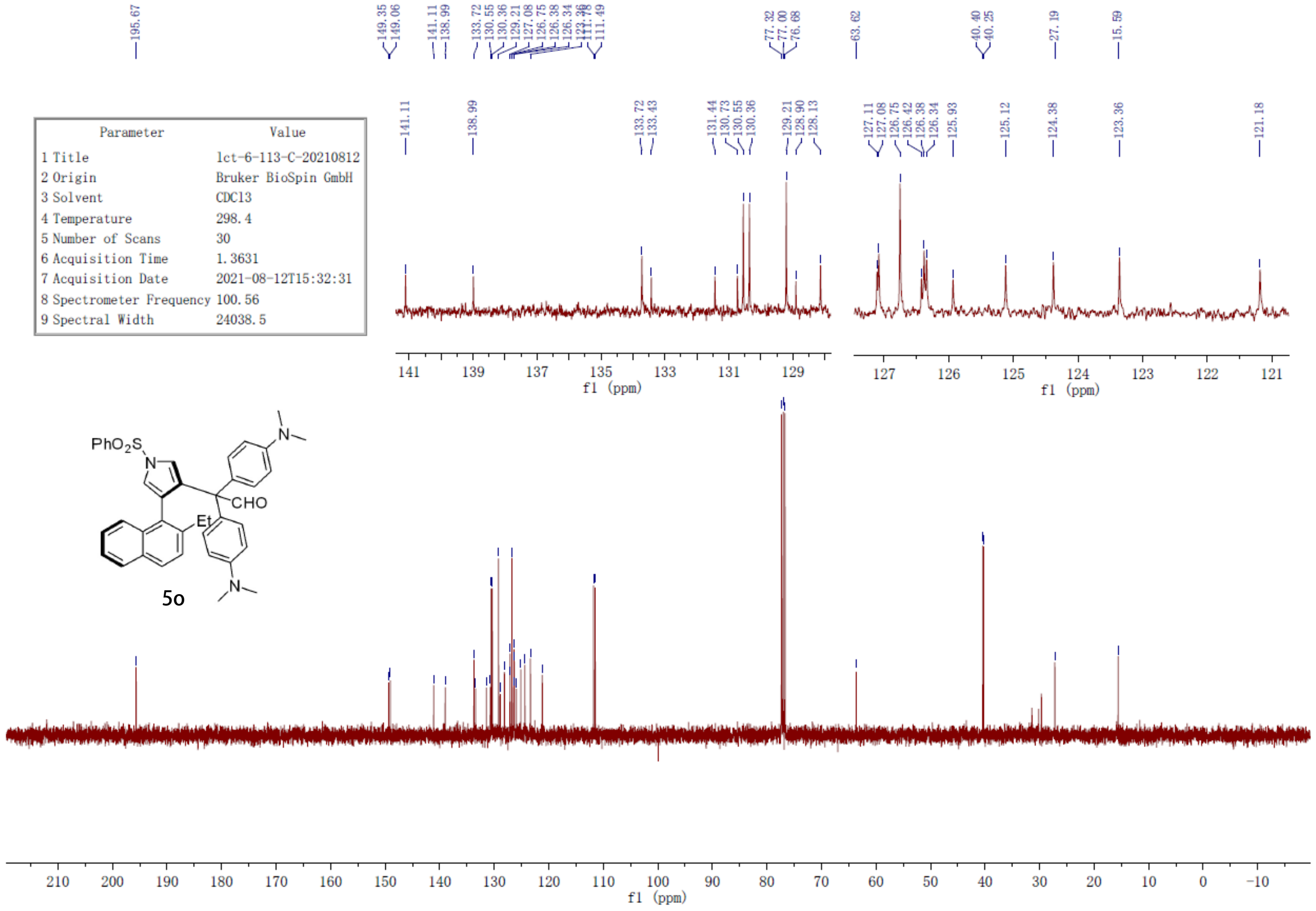

Supplementary Figure 83. ^{1}H and ^{13}C NMR spectra for **5o**

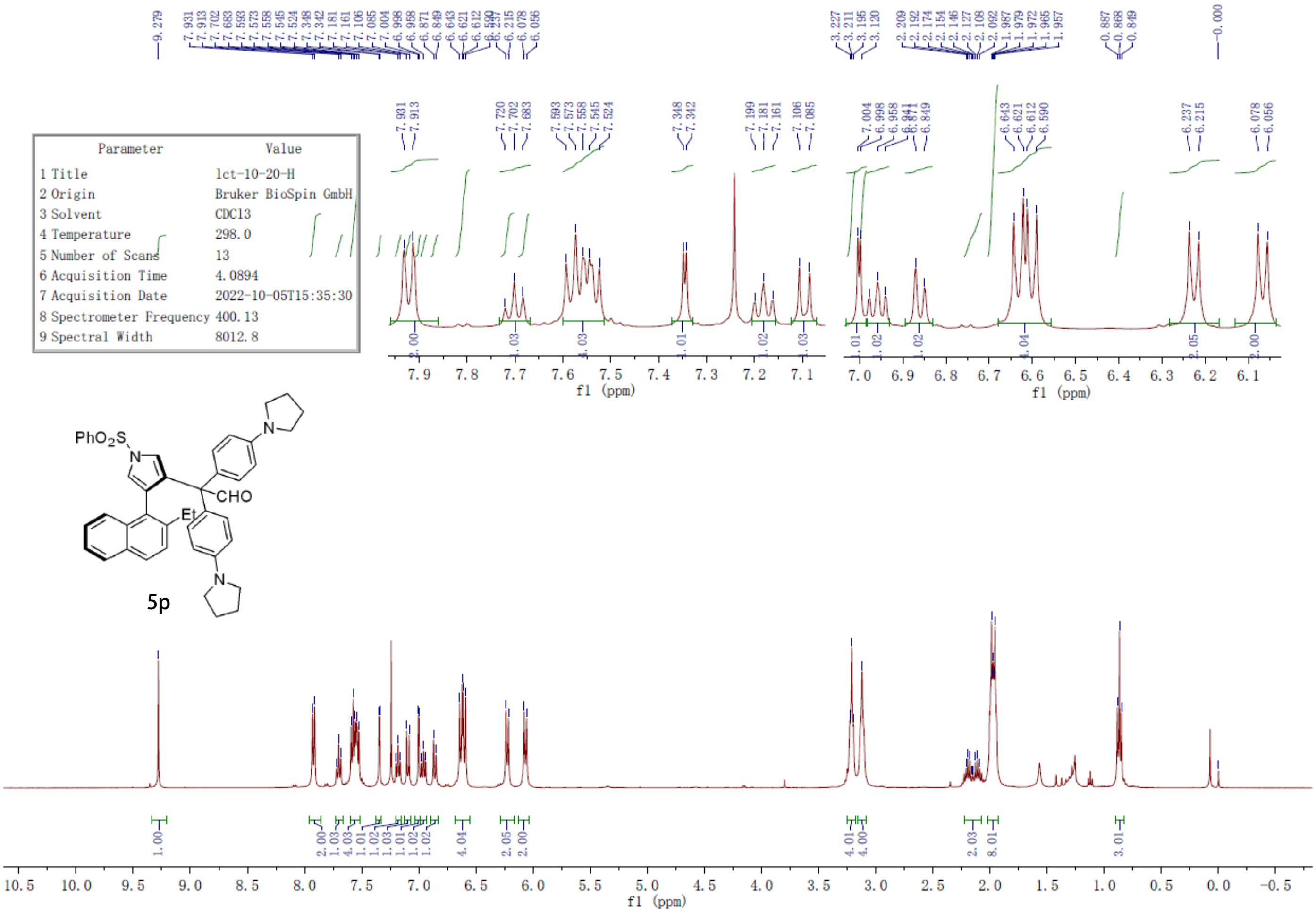

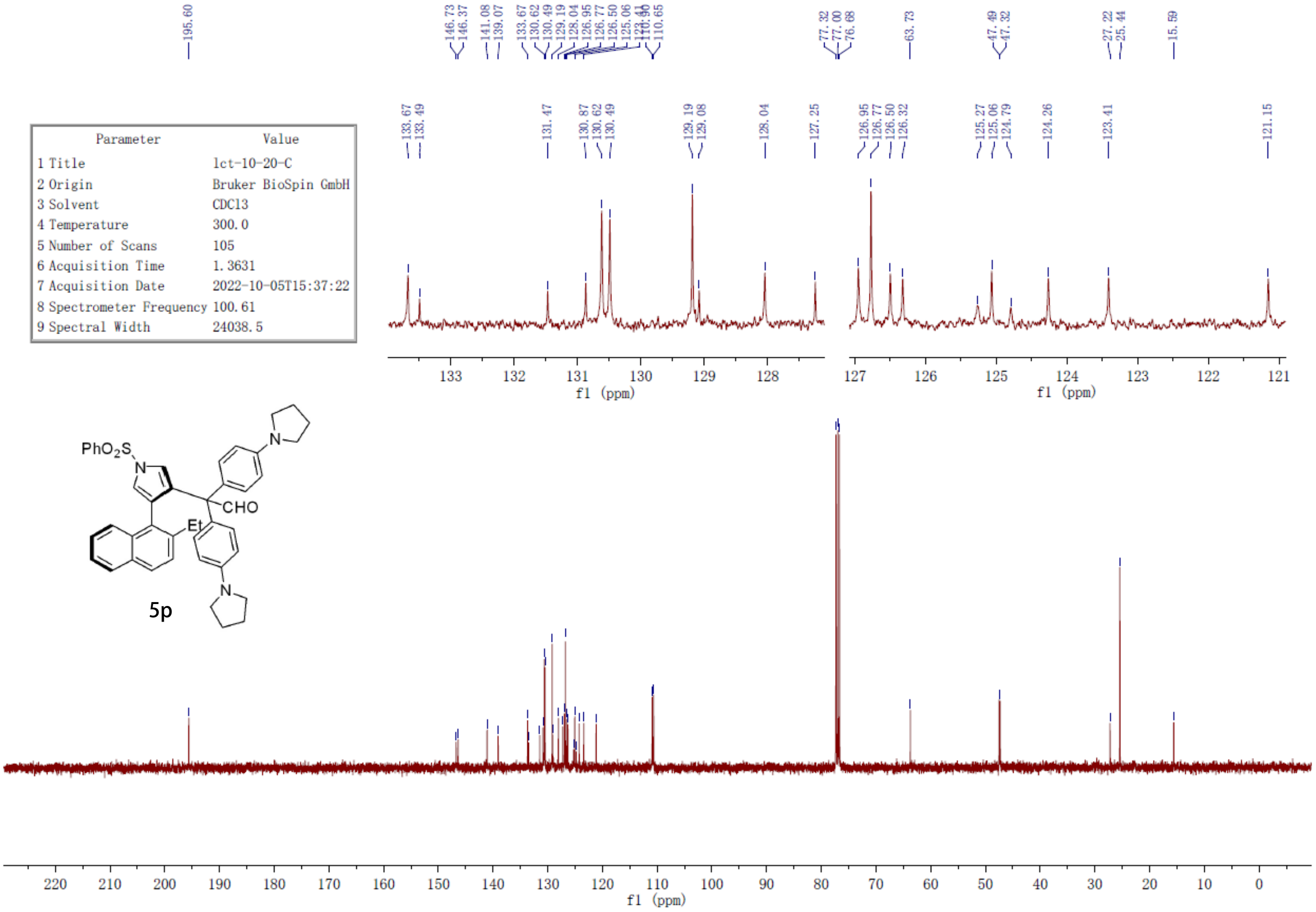

Supplementary Figure 84. ^{1}H and ^{13}C NMR spectra for **5p**

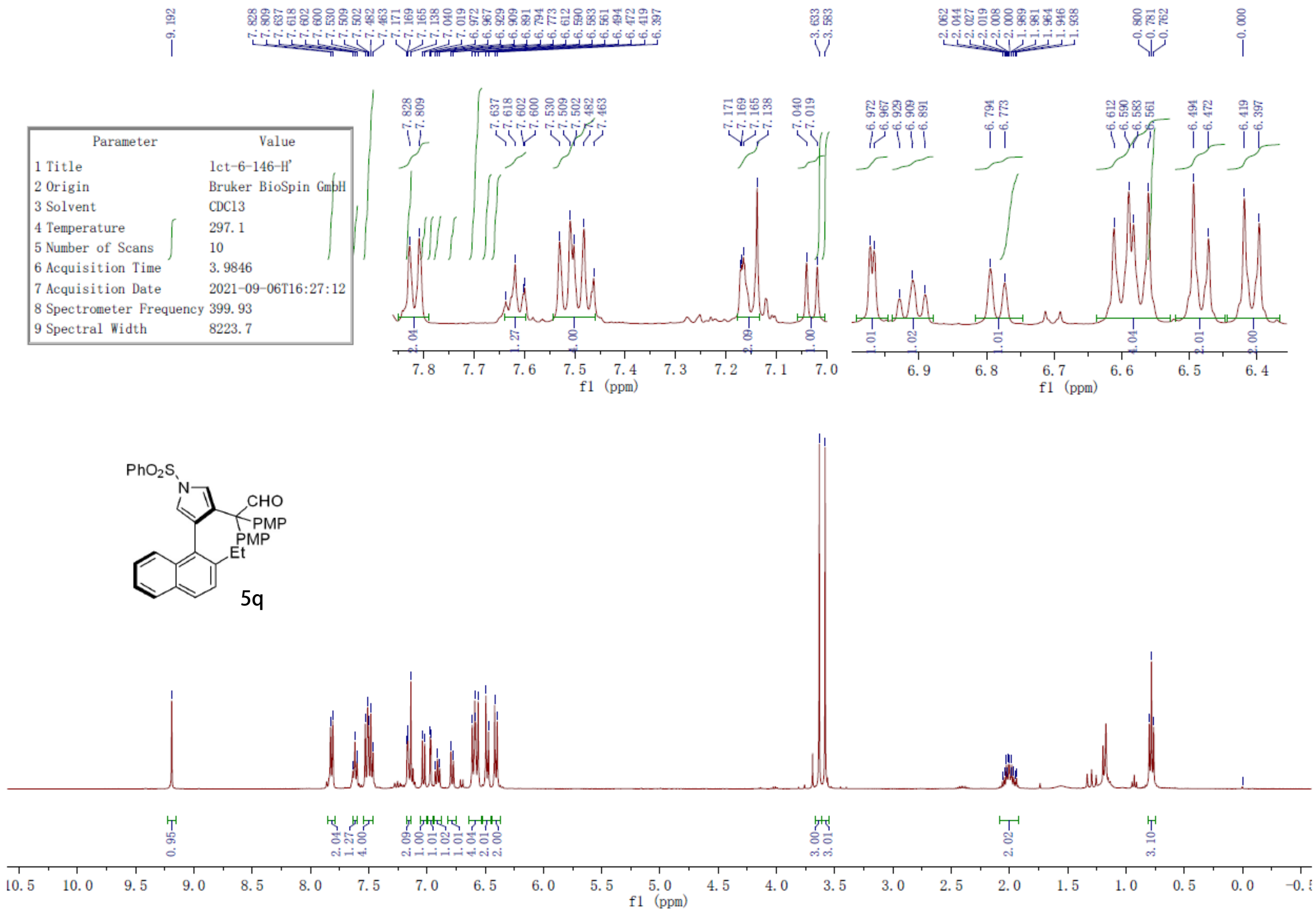

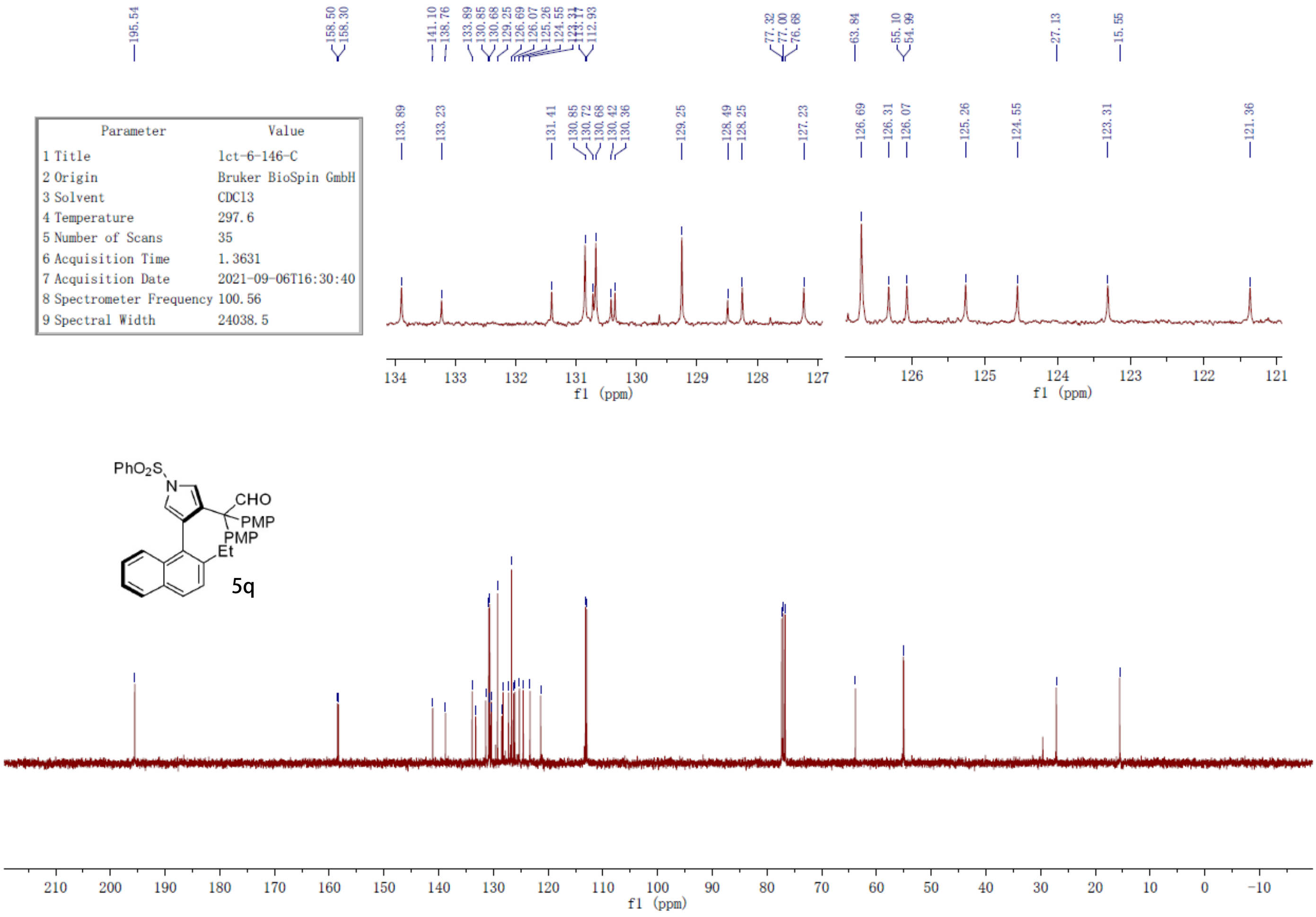

Supplementary Figure 85. ^{1}H and ^{13}C NMR spectra for **5q**

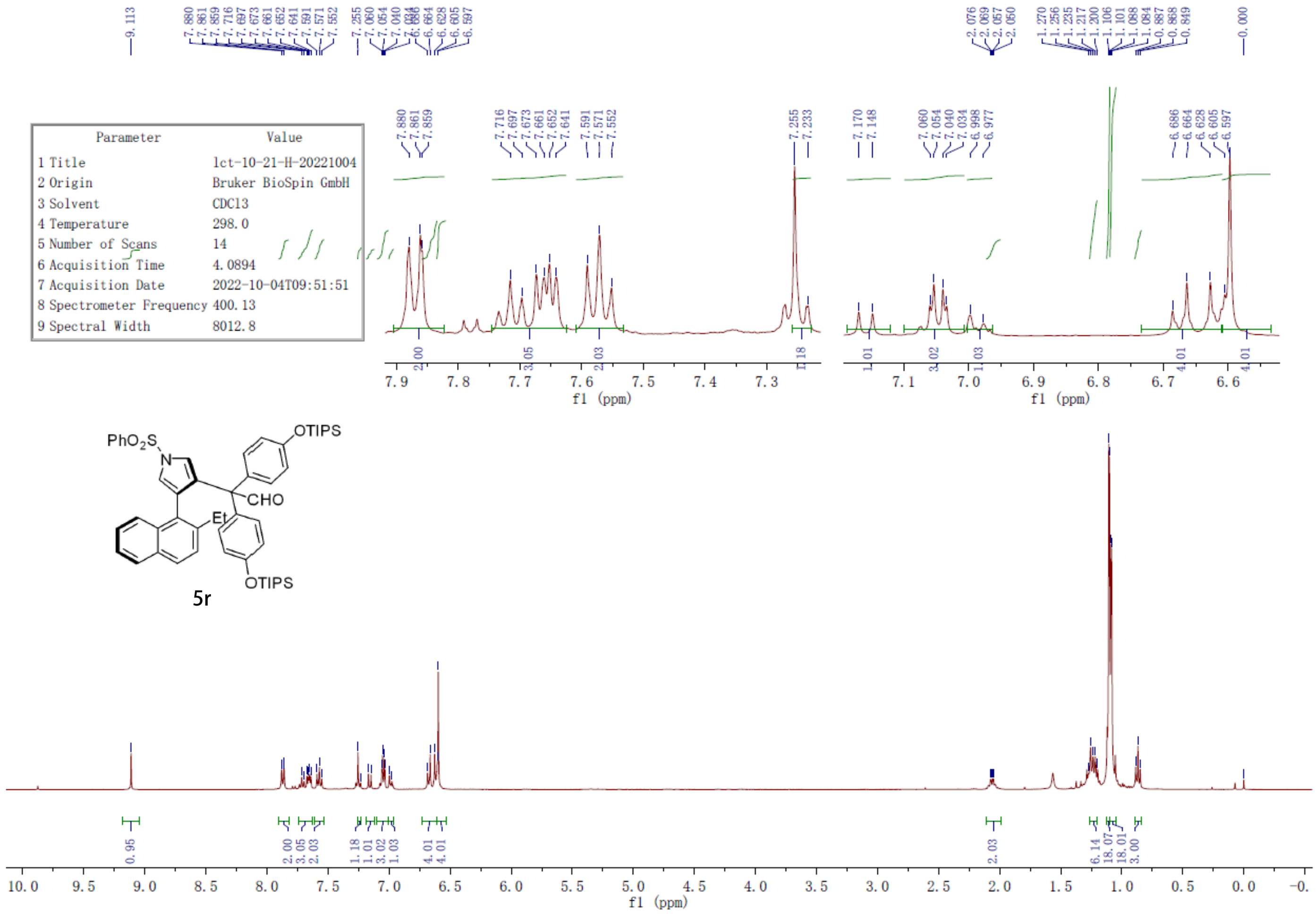

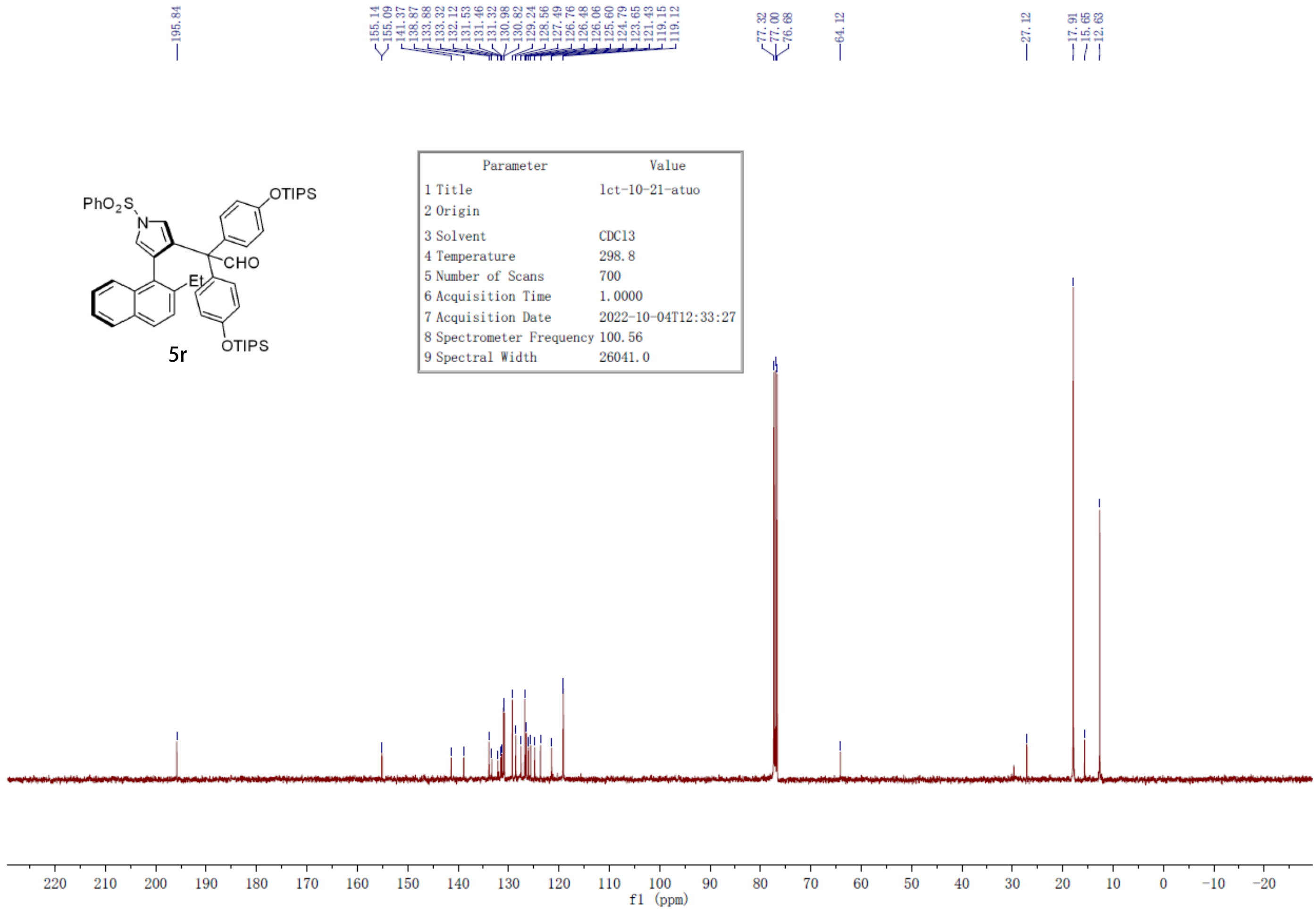

Supplementary Figure 86. ^{1}H and ^{13}C NMR spectra for **5r**

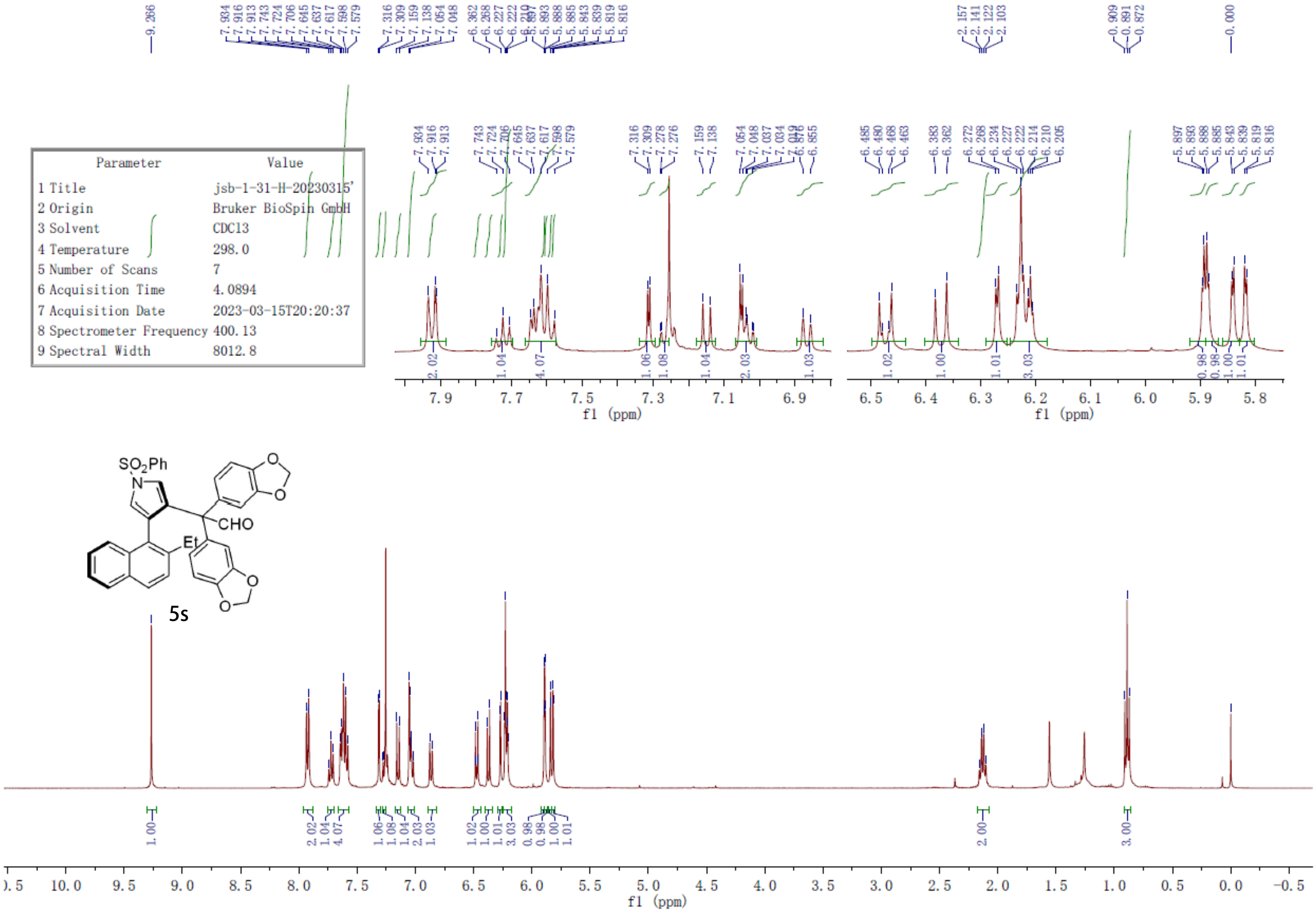

Parameter	Value
1 Title	jsb-1-31-H-20230315'
2 Origin	Bruker BioSpin GmbH
3 Solvent	CDCl3
4 Temperature	298.0
5 Number of Scans	7
6 Acquisition Time	4.0894
7 Acquisition Date	2023-03-15T20:20:37
8 Spectrometer Frequency	400.13
9 Spectral Width	8012.8

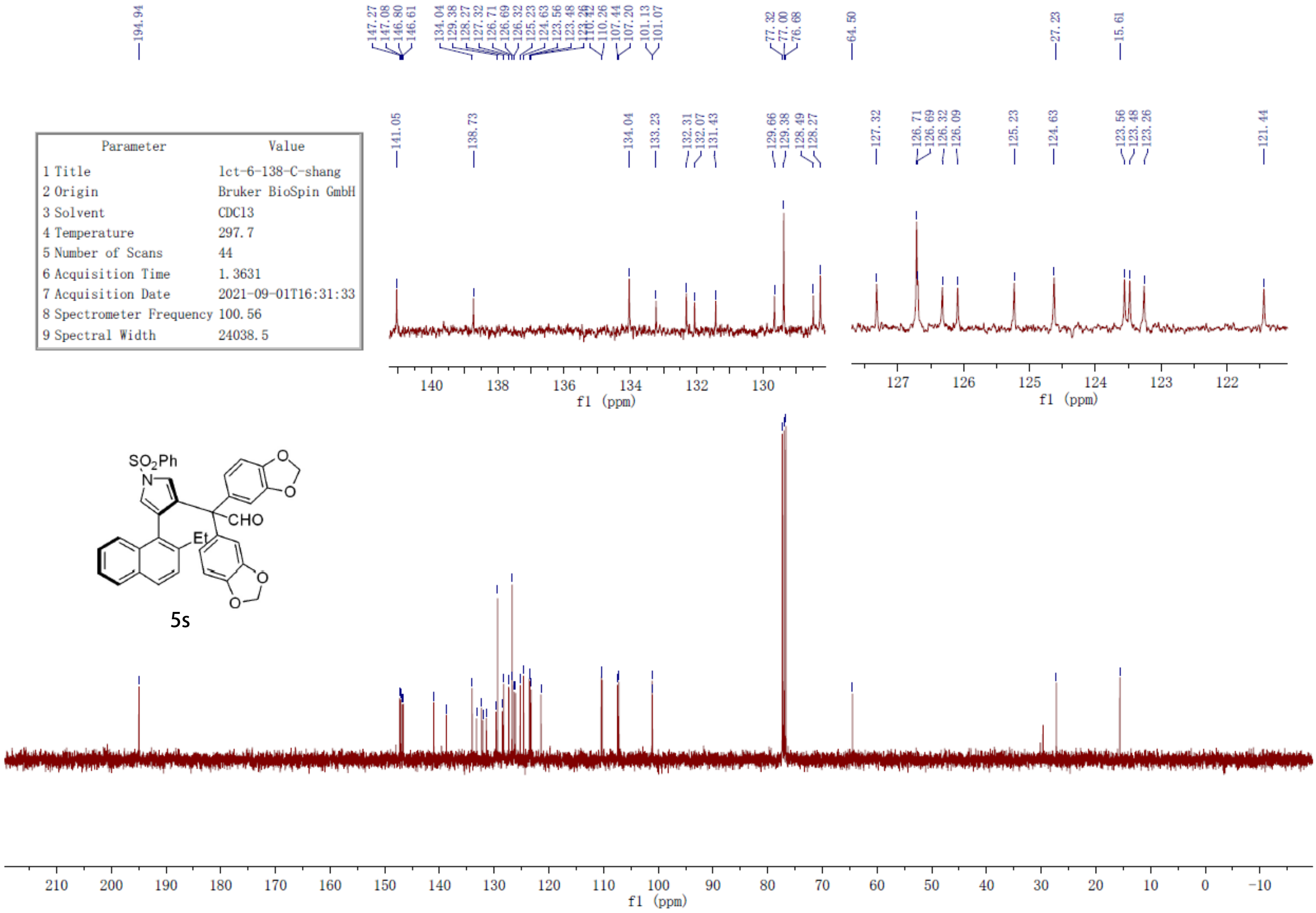

Parameter	Value
1 Title	lct-6-138-C-shang
2 Origin	Bruker BioSpin GmbH
3 Solvent	CDCl3
4 Temperature	297.7
5 Number of Scans	44
6 Acquisition Time	1.3631
7 Acquisition Date	2021-09-01T16:31:33
8 Spectrometer Frequency	100.56
9 Spectral Width	24038.5

Supplementary Figure 87. ^{1}H and ^{13}C NMR spectra for **5s**

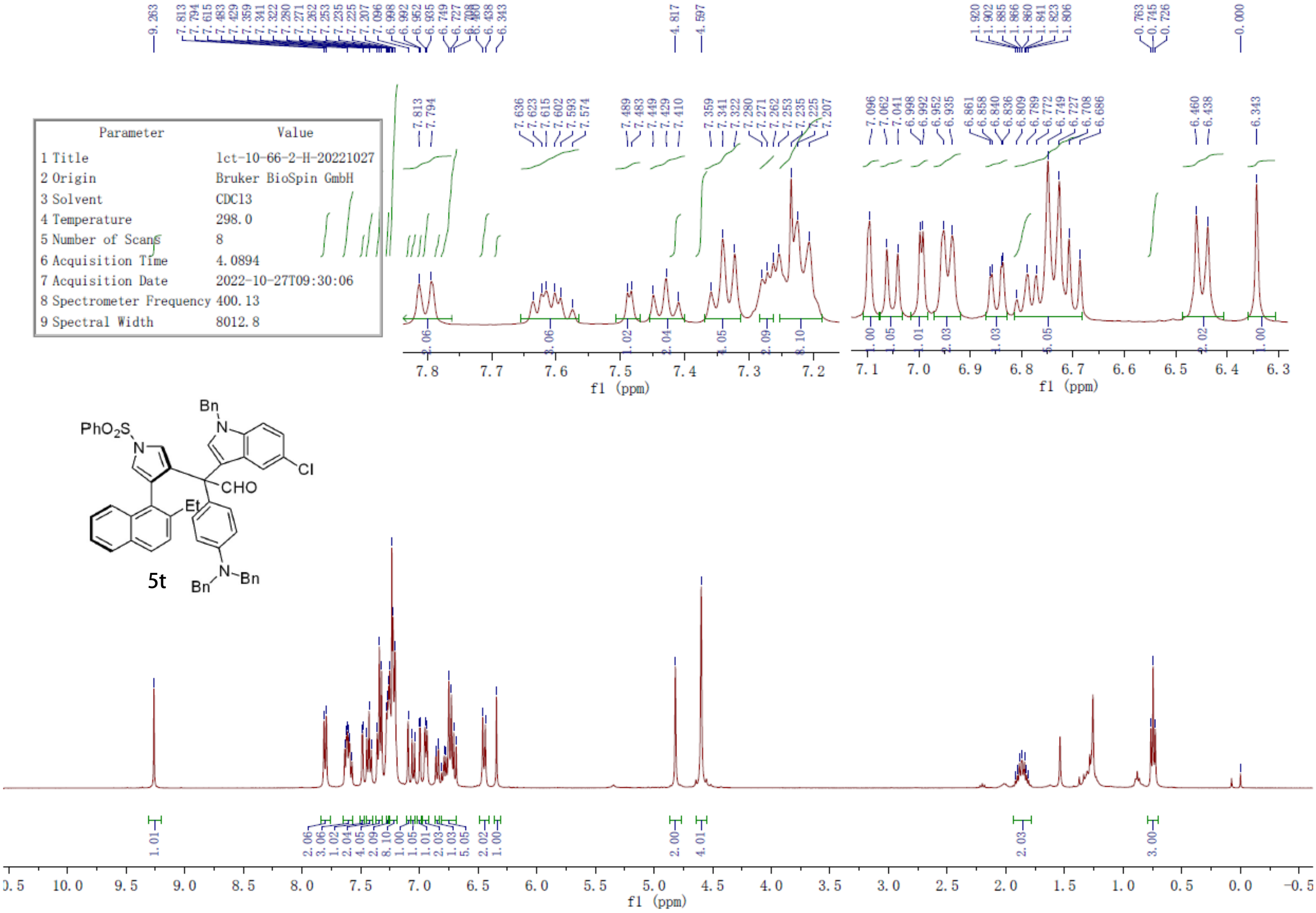

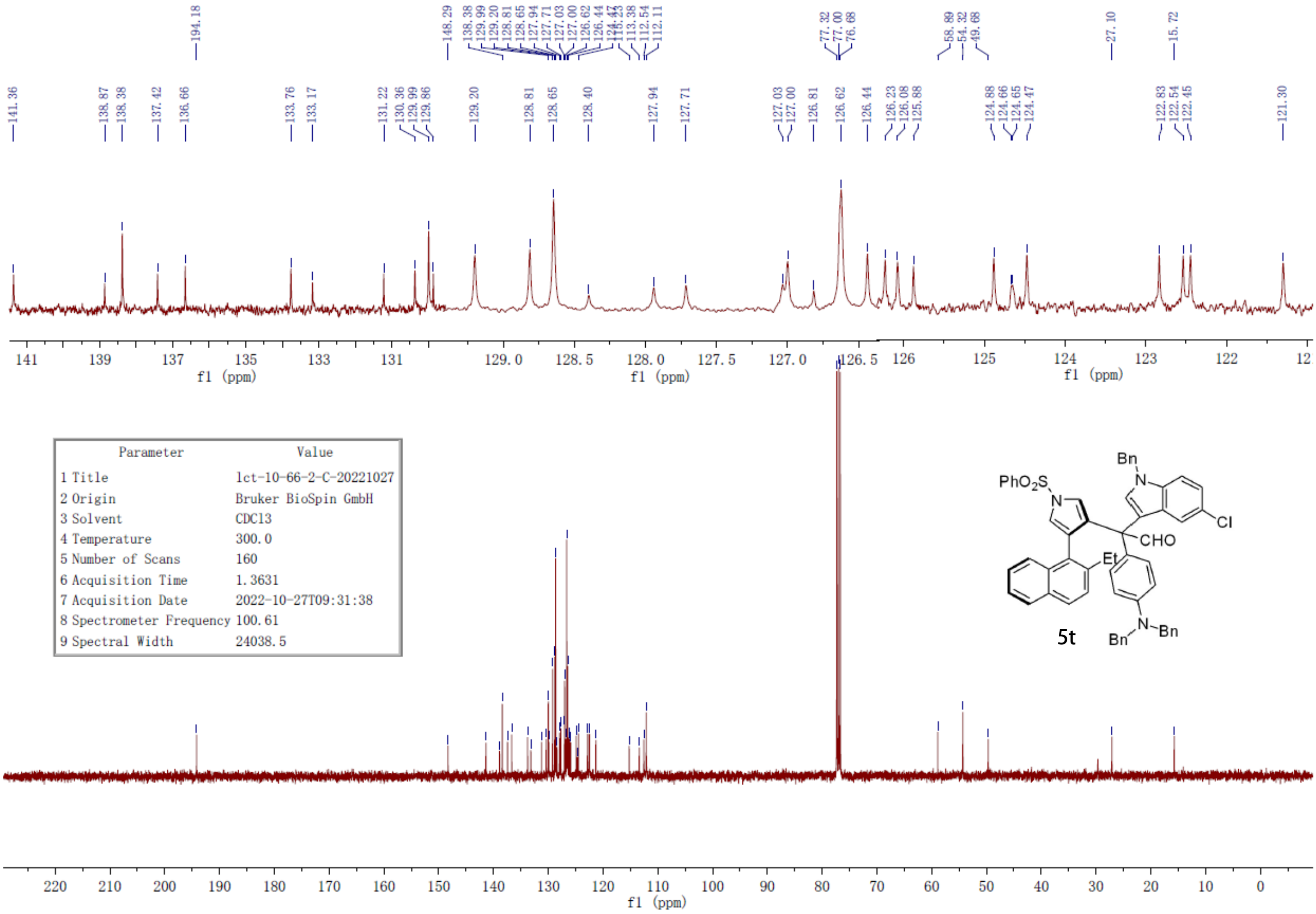

Supplementary Figure 88. ^{1}H and ^{13}C NMR spectra for **5t**

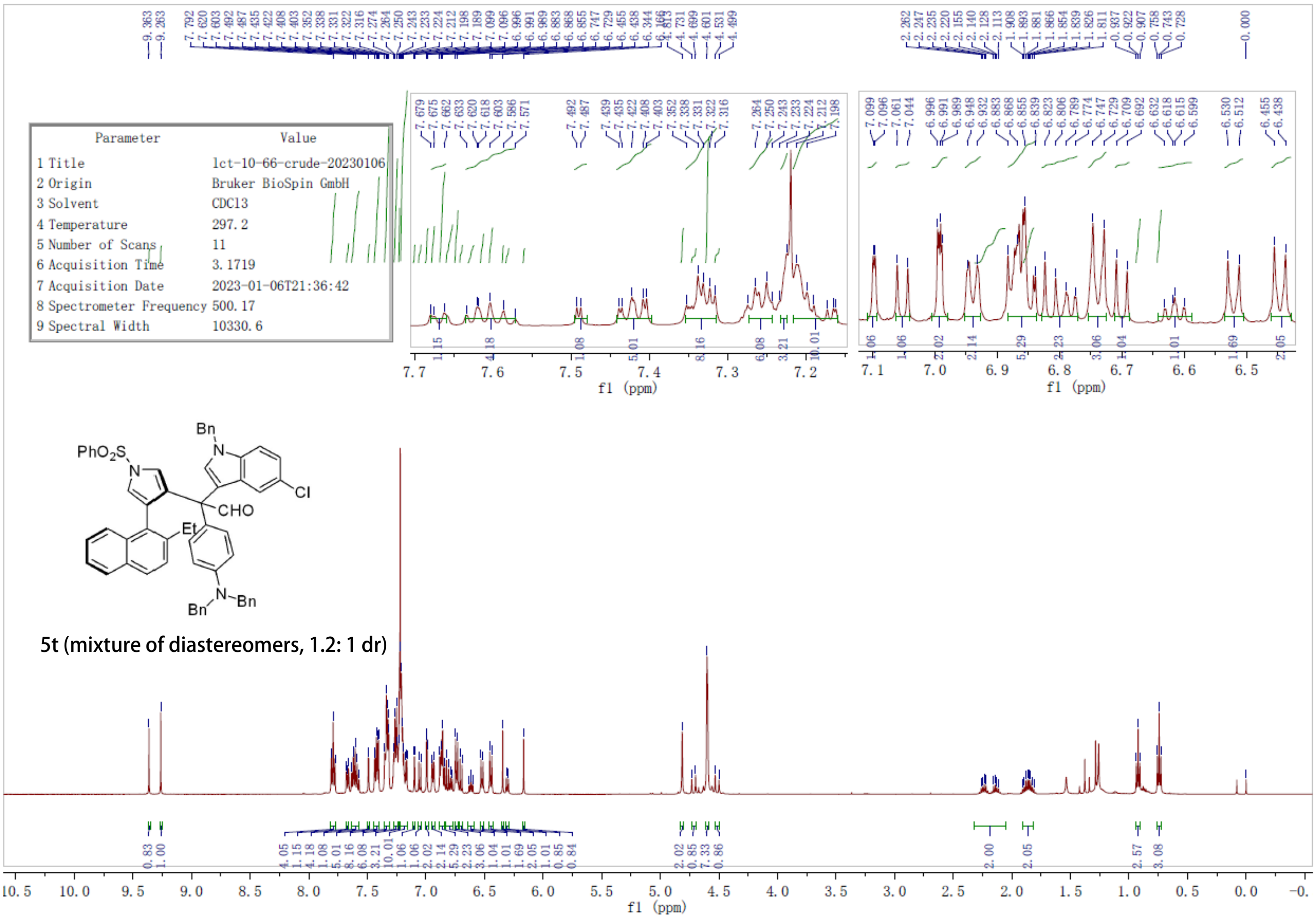

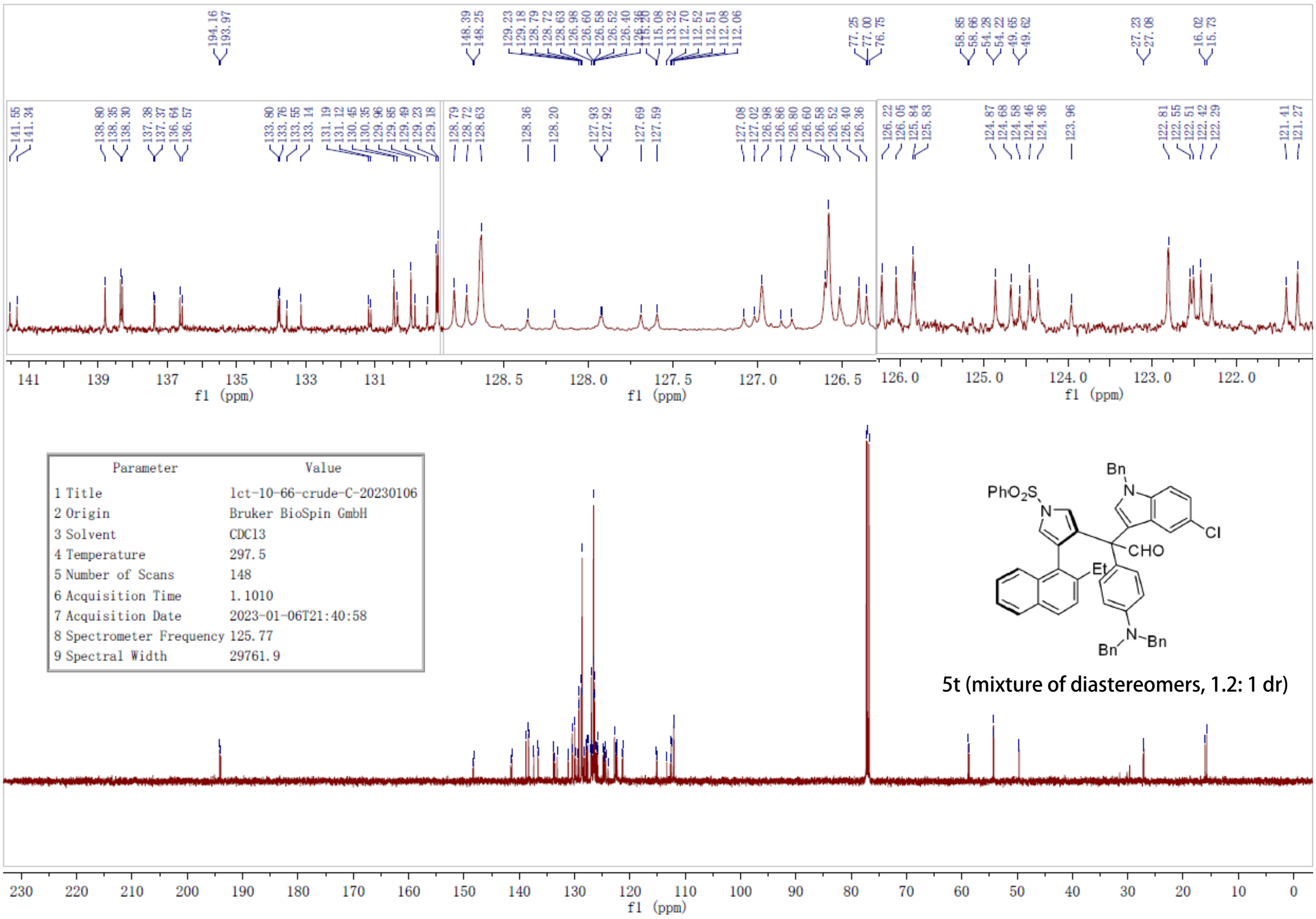

Supplementary Figure 89. ^{1}H and ^{13}C NMR spectra for **5t (mixture of diastereomers)**

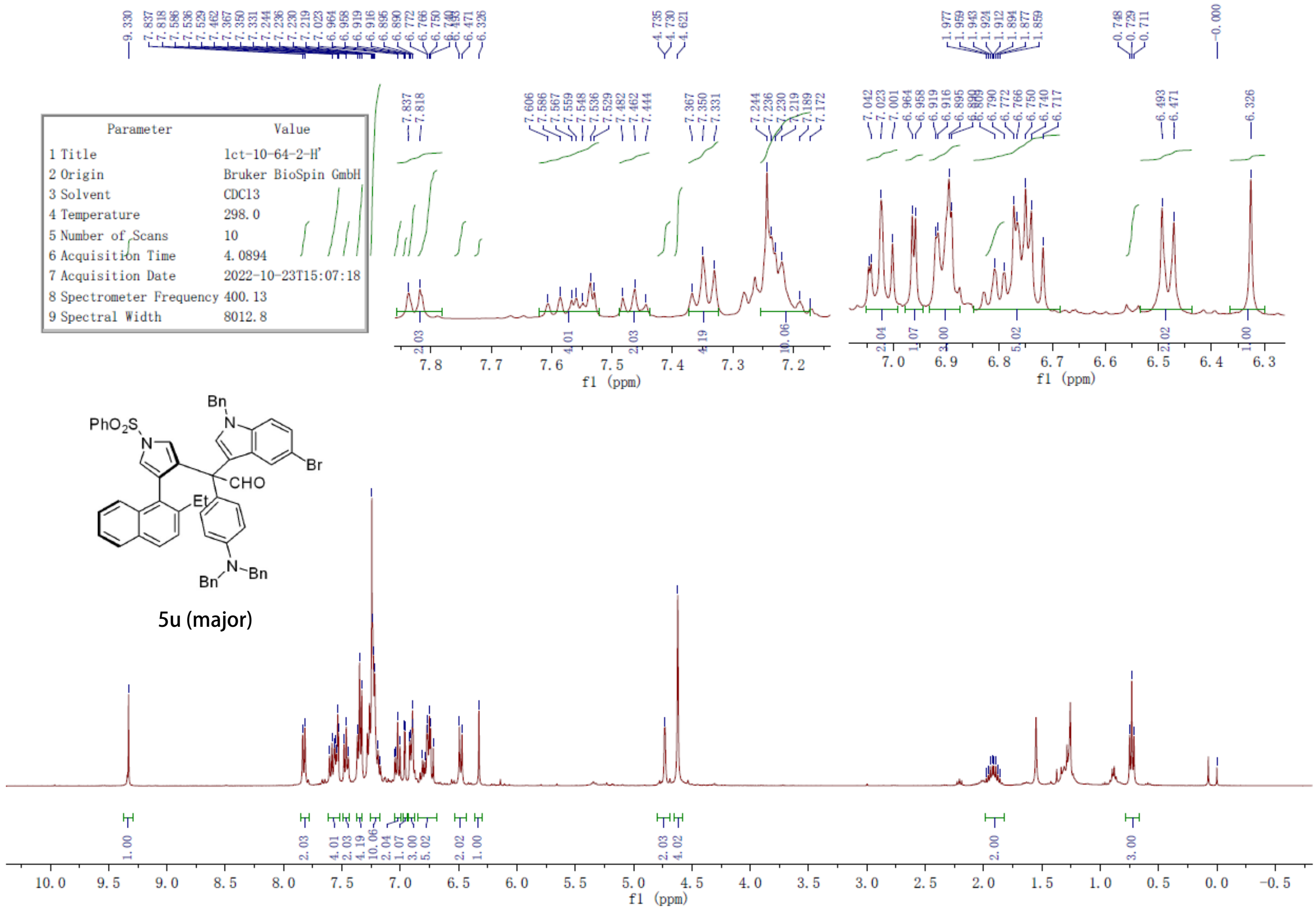

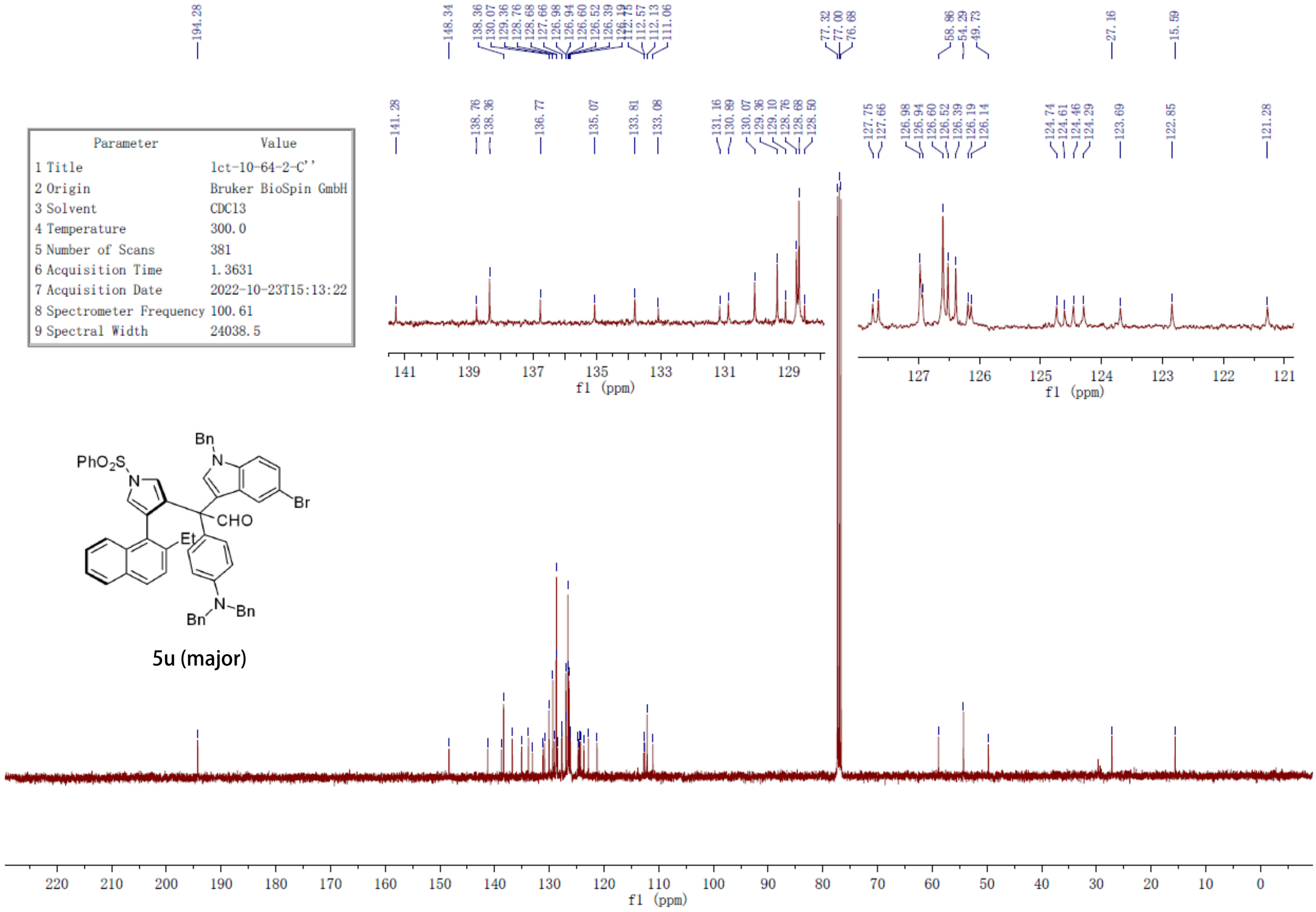

Supplementary Figure 90. ^{1}H and ^{13}C NMR spectra for **5u (major)**

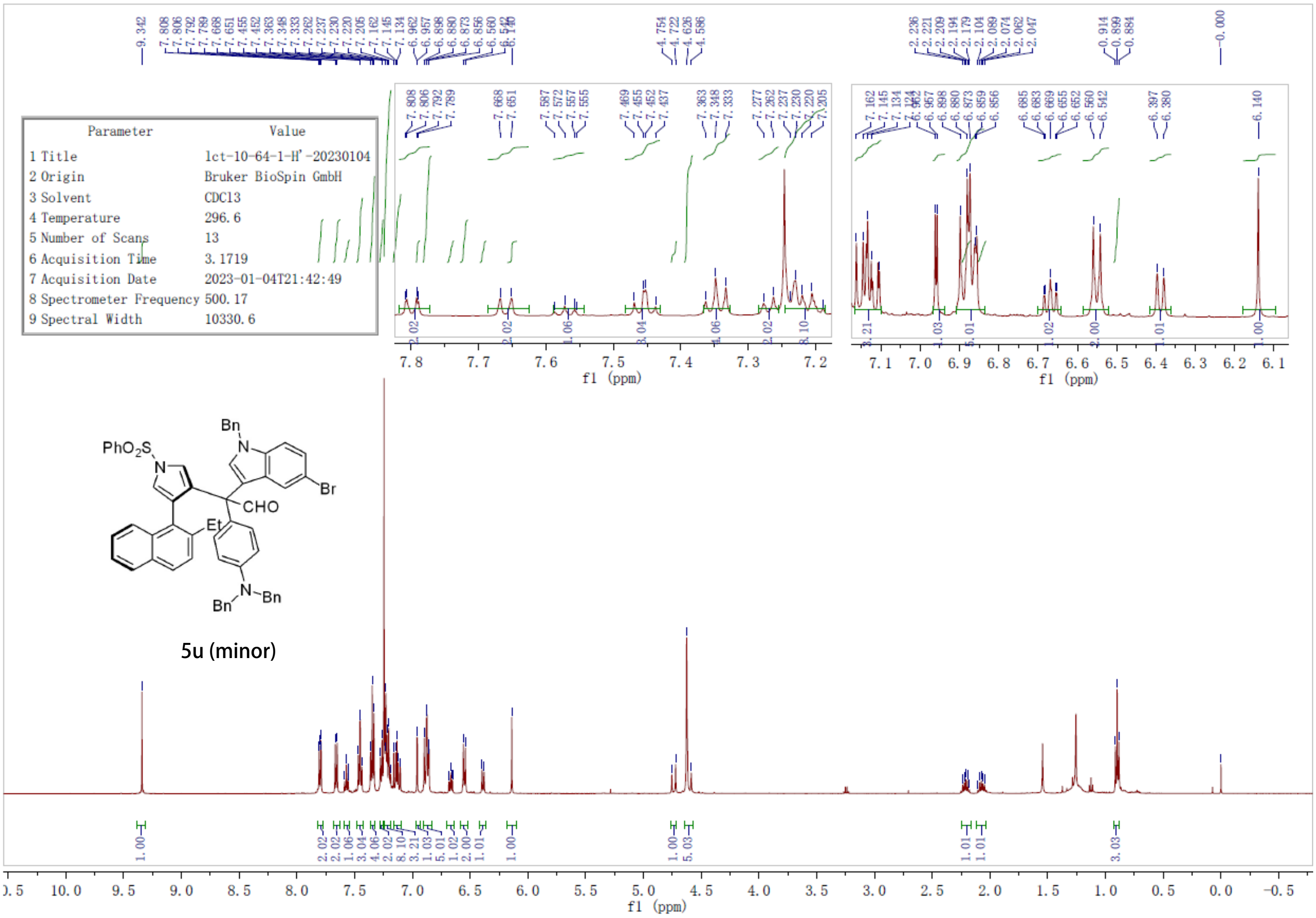

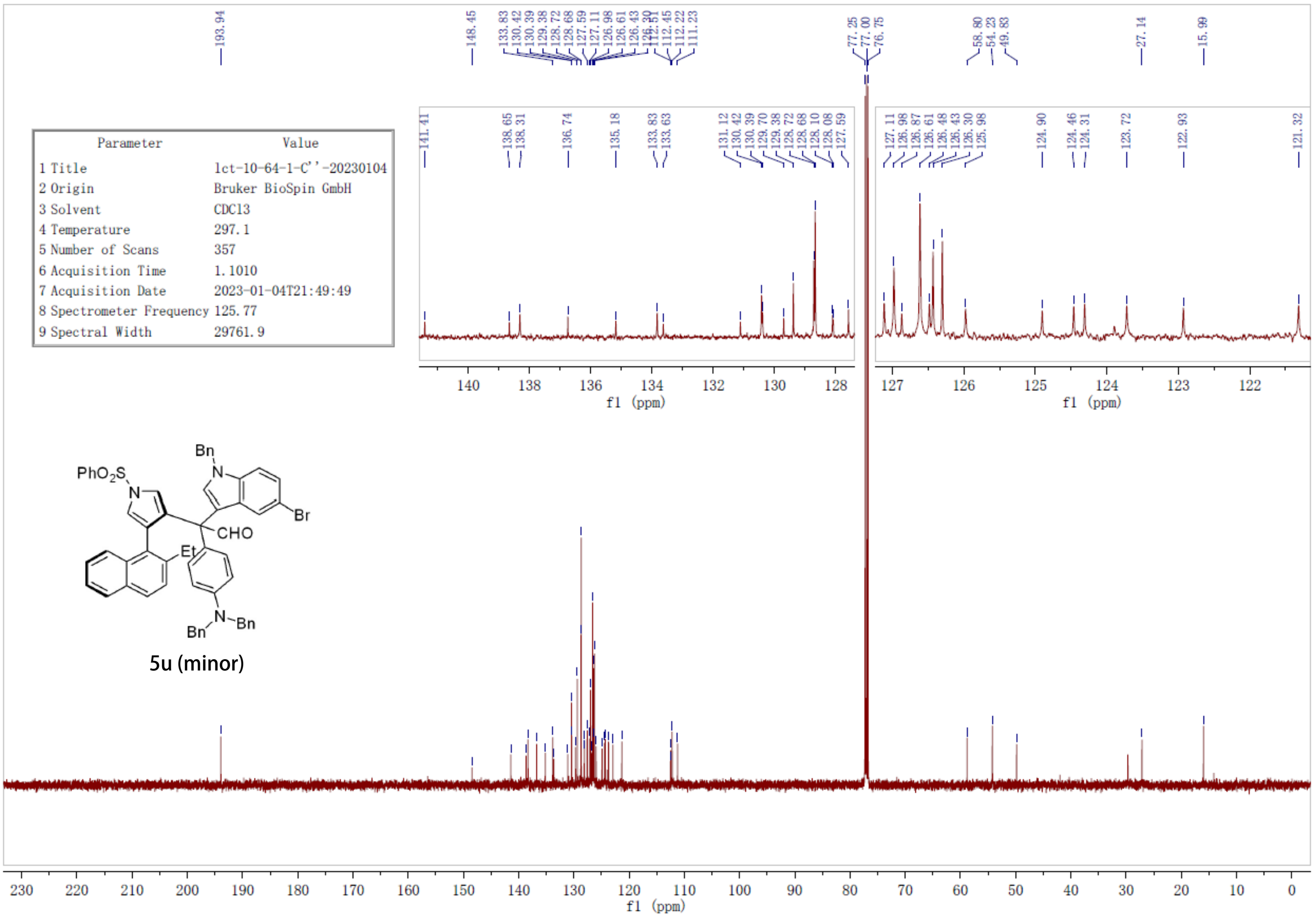

Supplementary Figure 91. ^{1}H and ^{13}C NMR spectra for **5u (minor)**

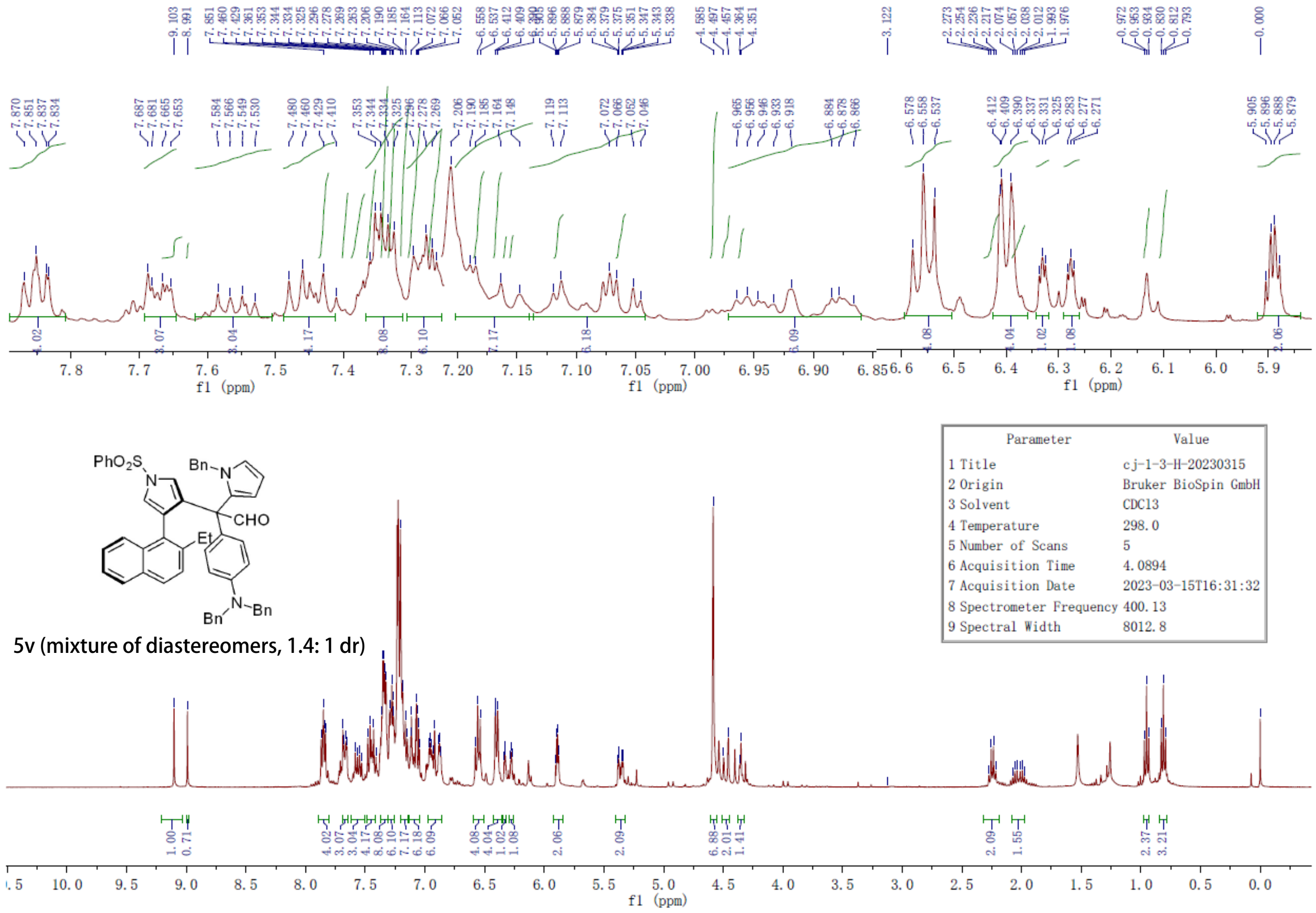

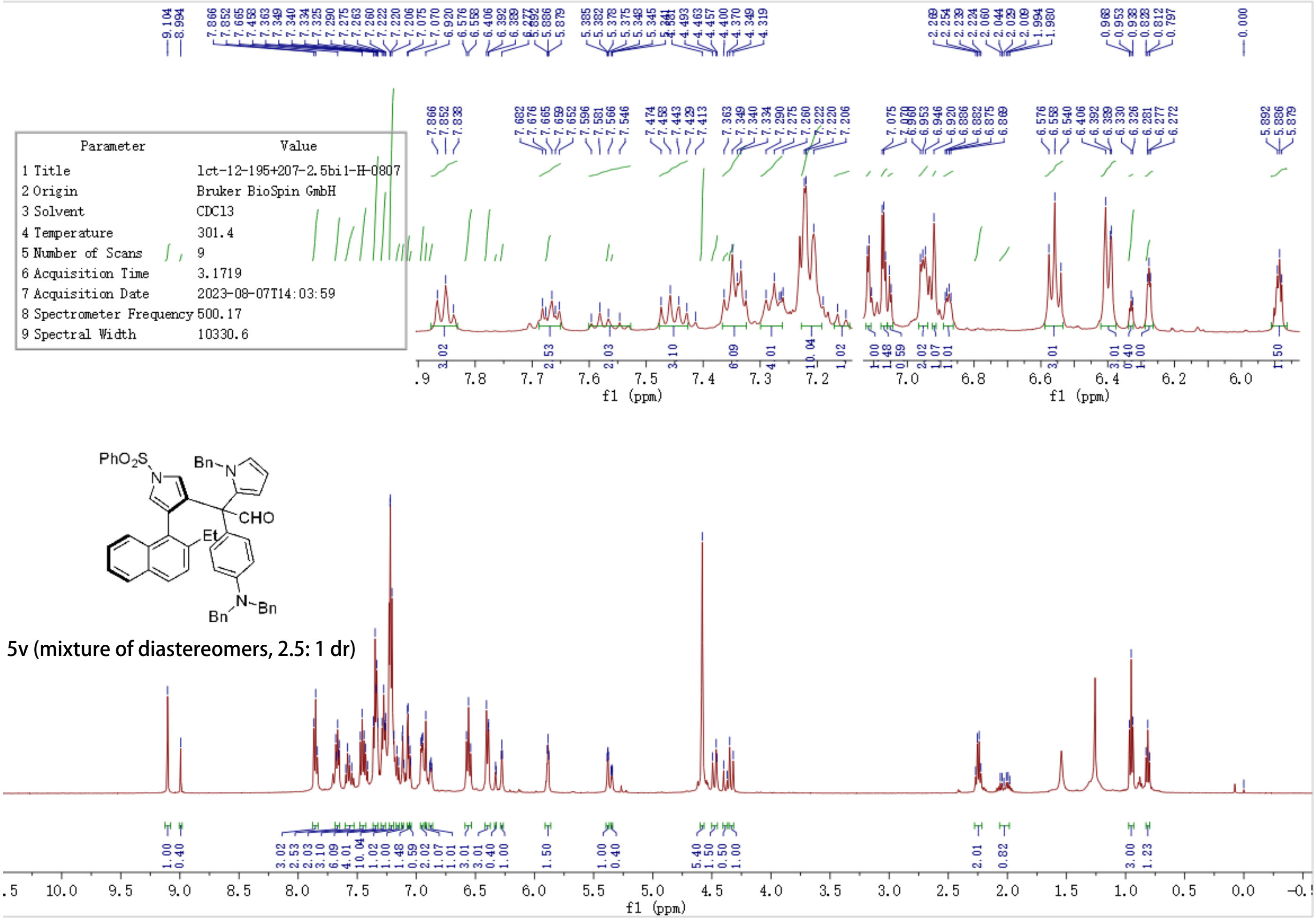

Supplementary Figure 92. ^{1}H NMR spectra for **5v (mixture of diastereomers)**

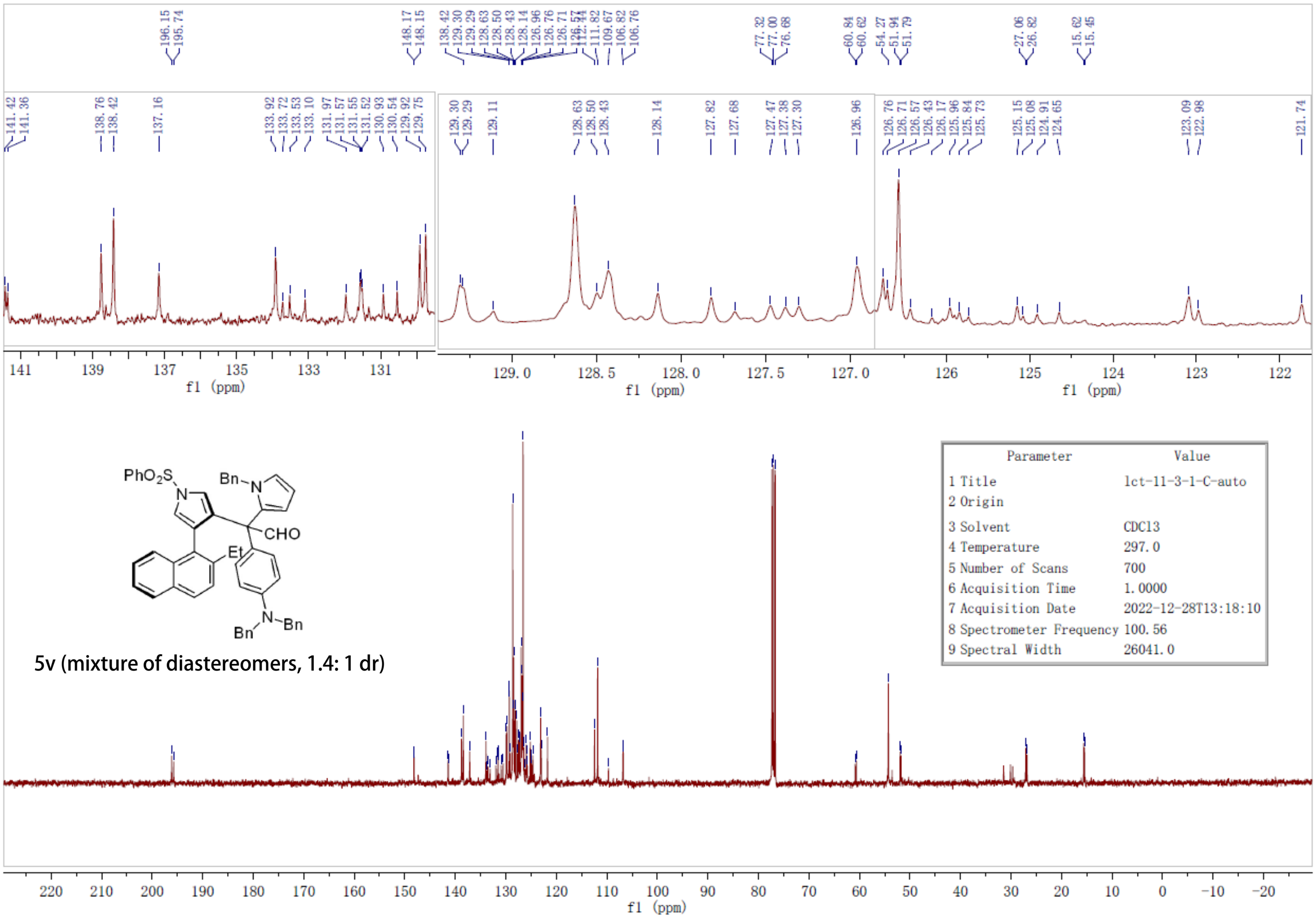

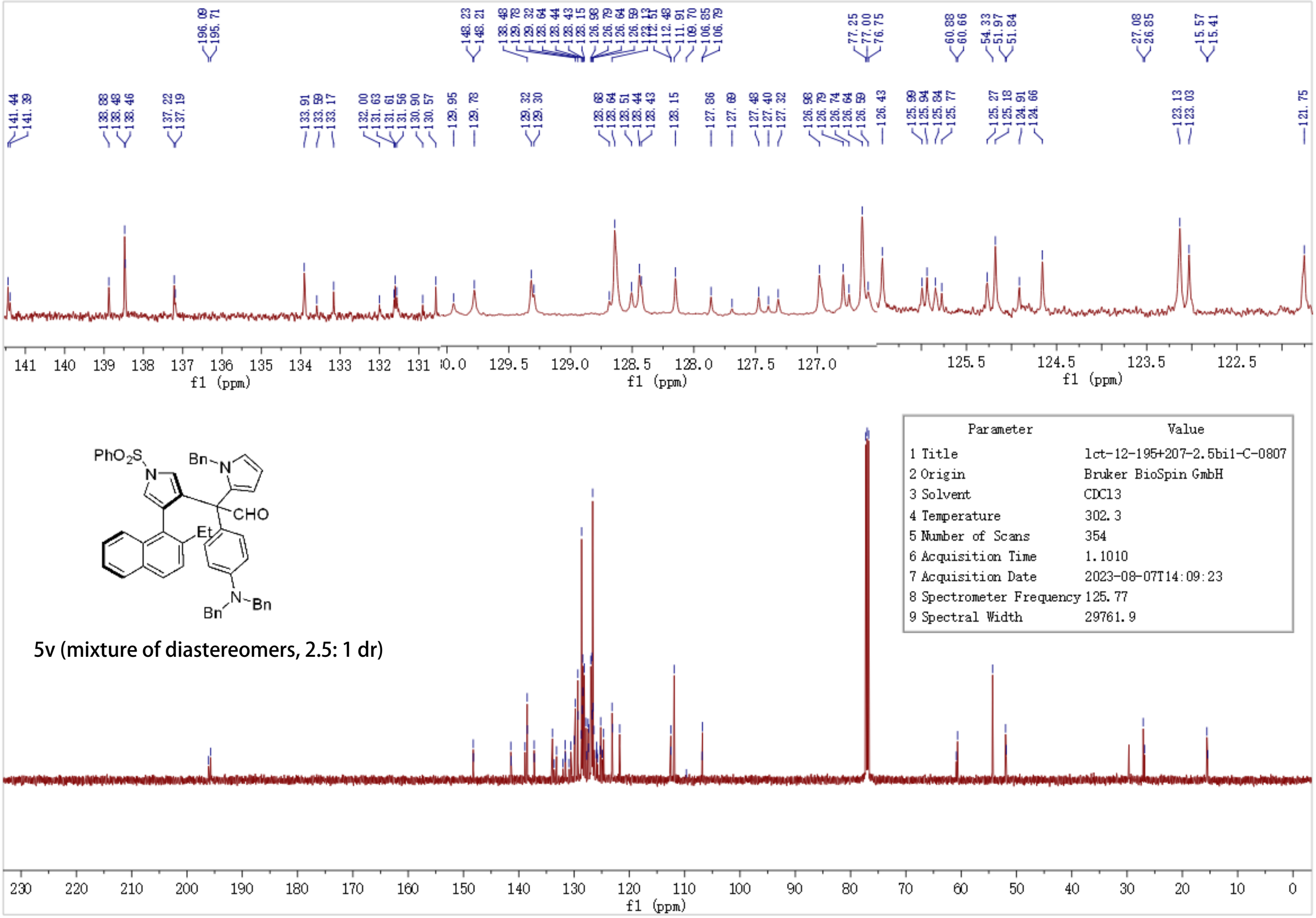

Supplementary Figure 93. ^{13}C NMR spectra for **5v (mixture of diastereomers)**

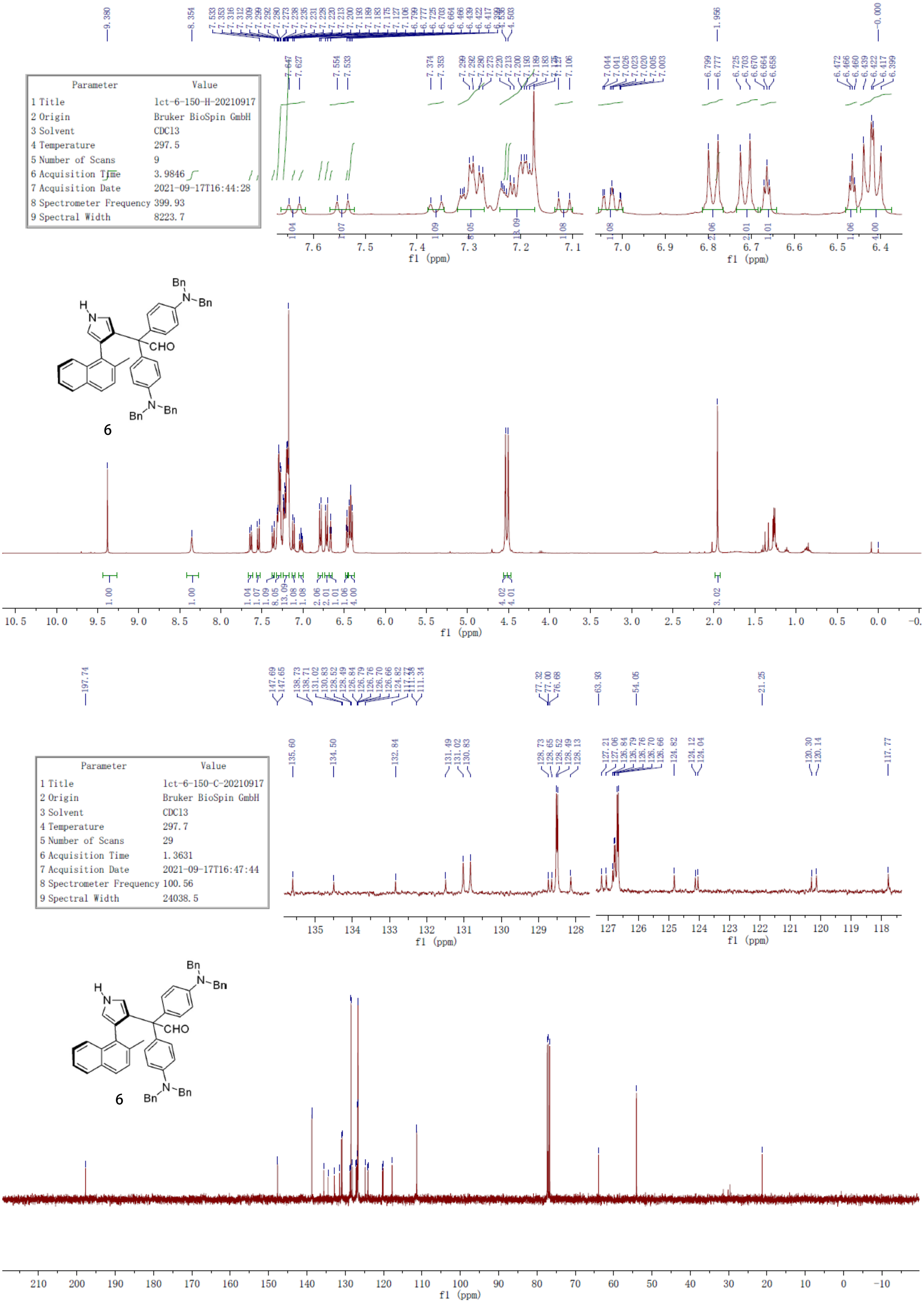

Supplementary Figure 94. ^{1}H and ^{13}C NMR spectra for **6**

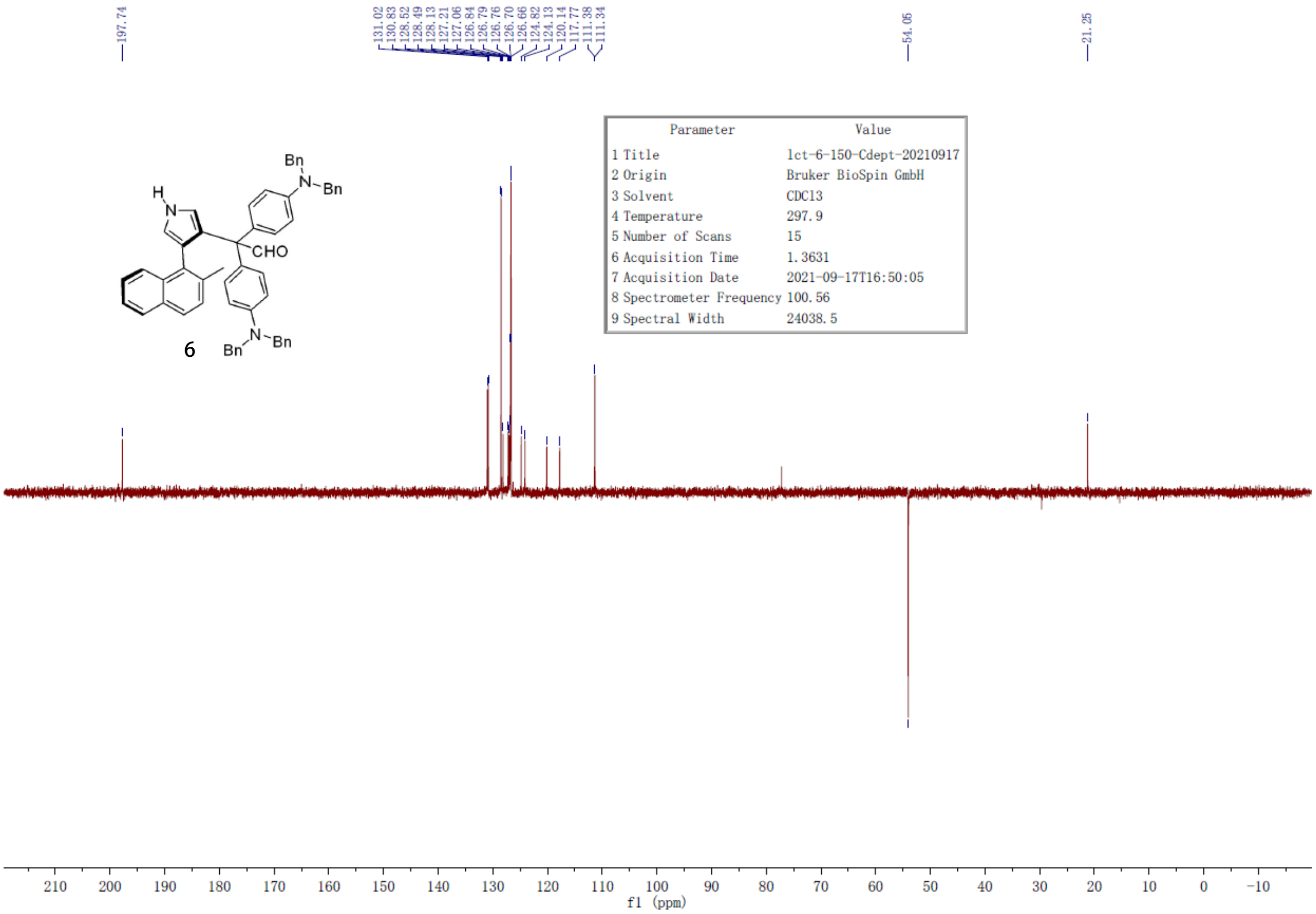

Supplementary Figure 95. DEPT 135 NMR spectra for **6**

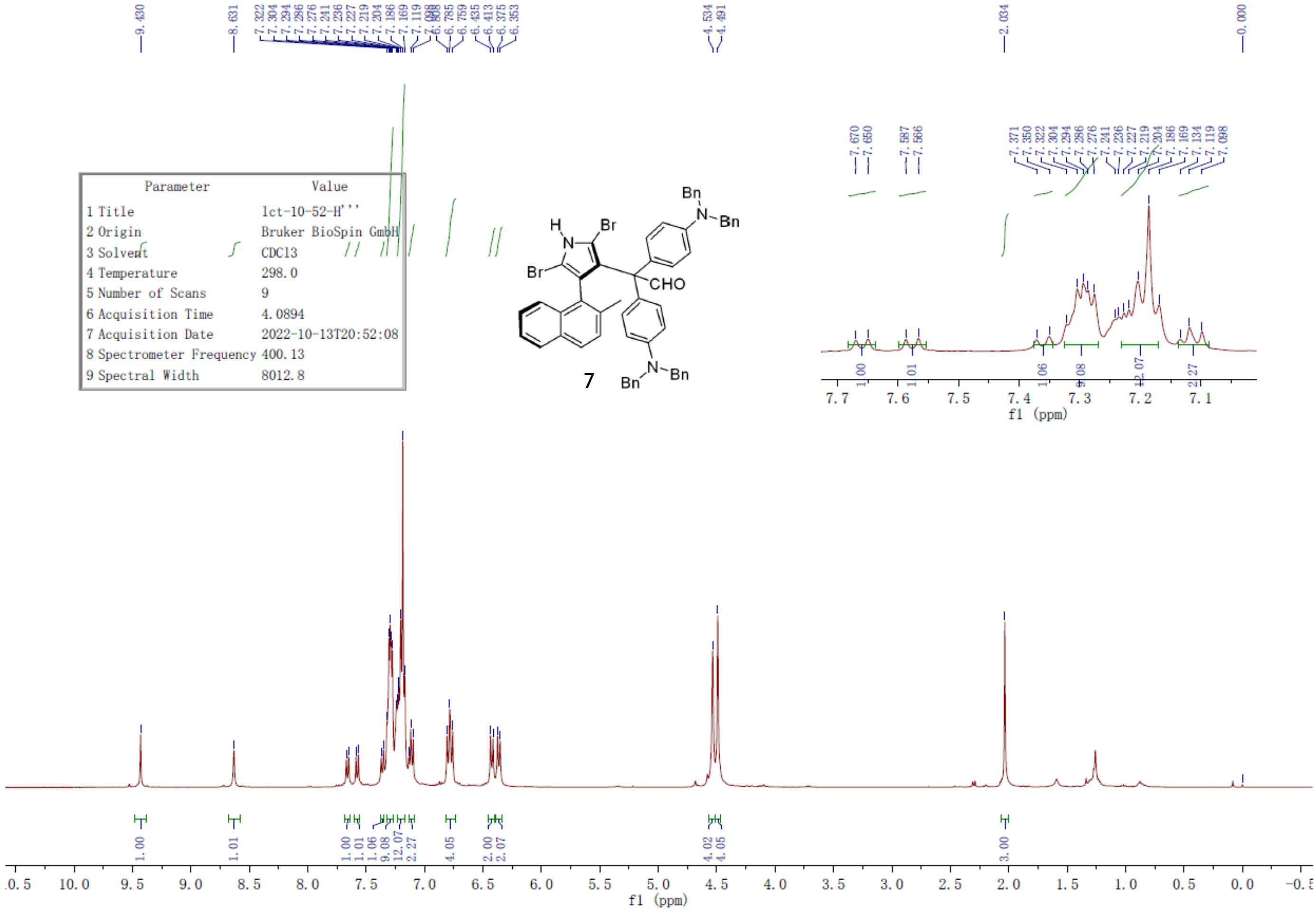

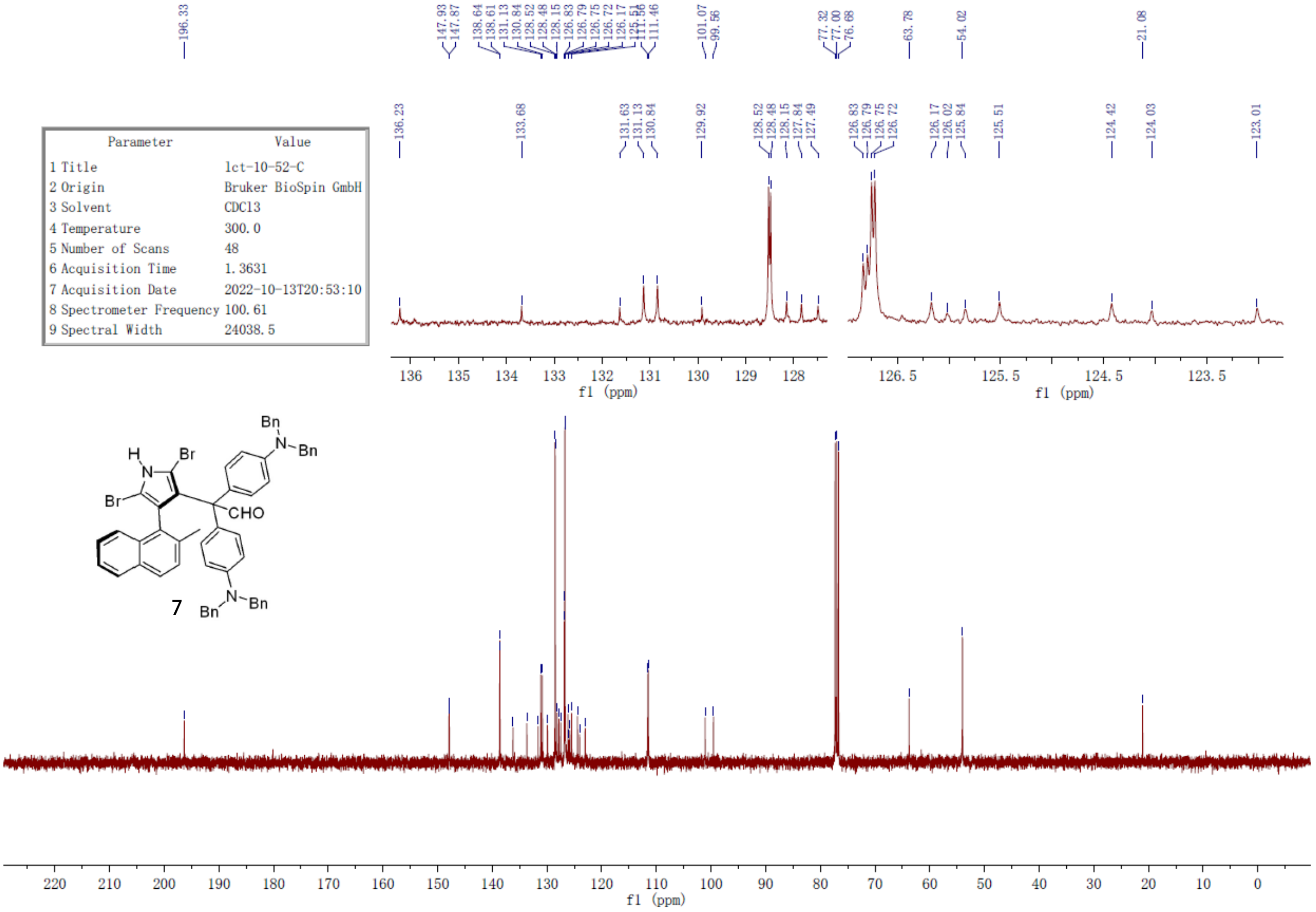

Supplementary Figure 96. ^{1}H and ^{13}C NMR spectra for **7**

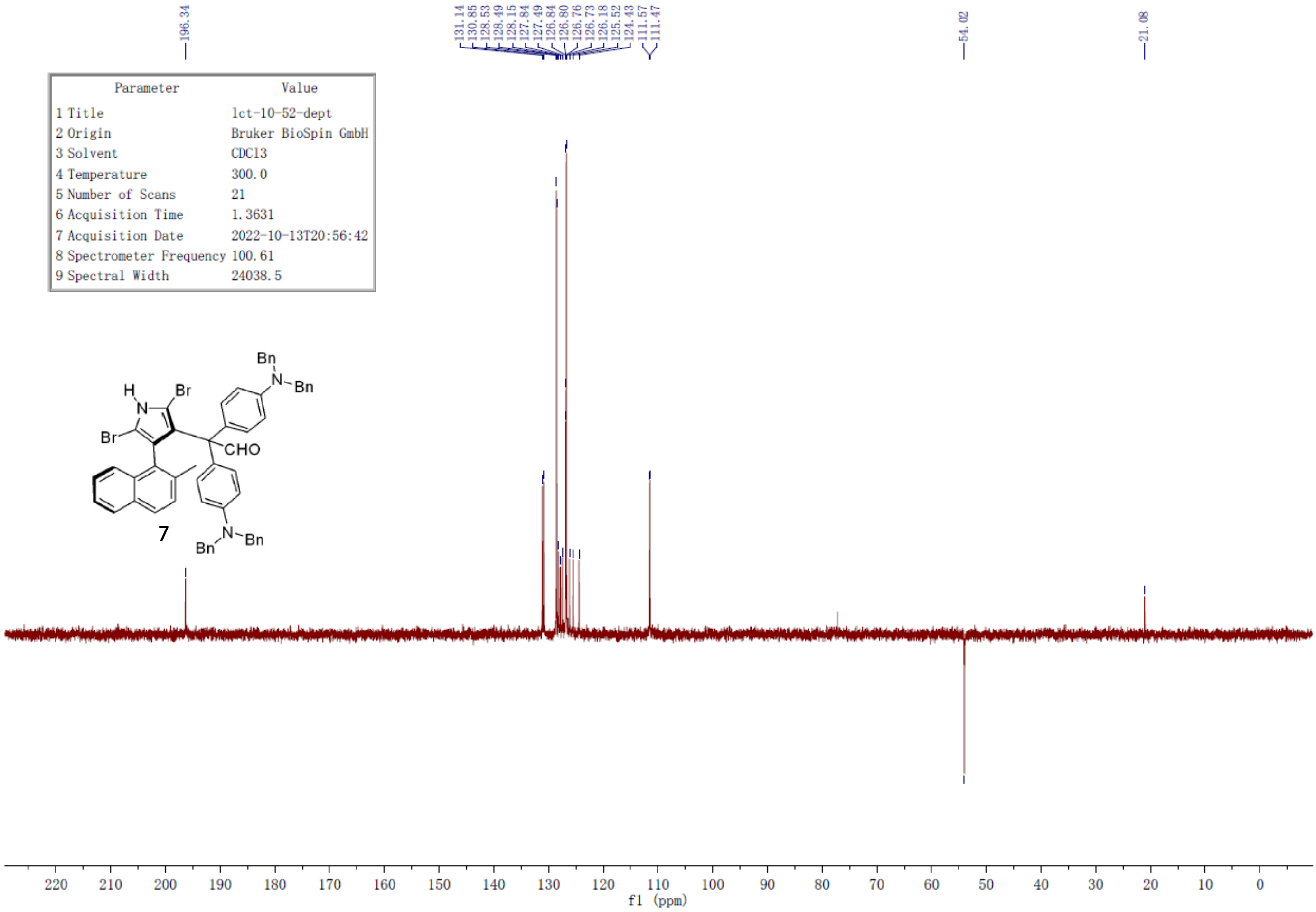

Supplementary Figure 97. DEPT 135 NMR spectra for **7**

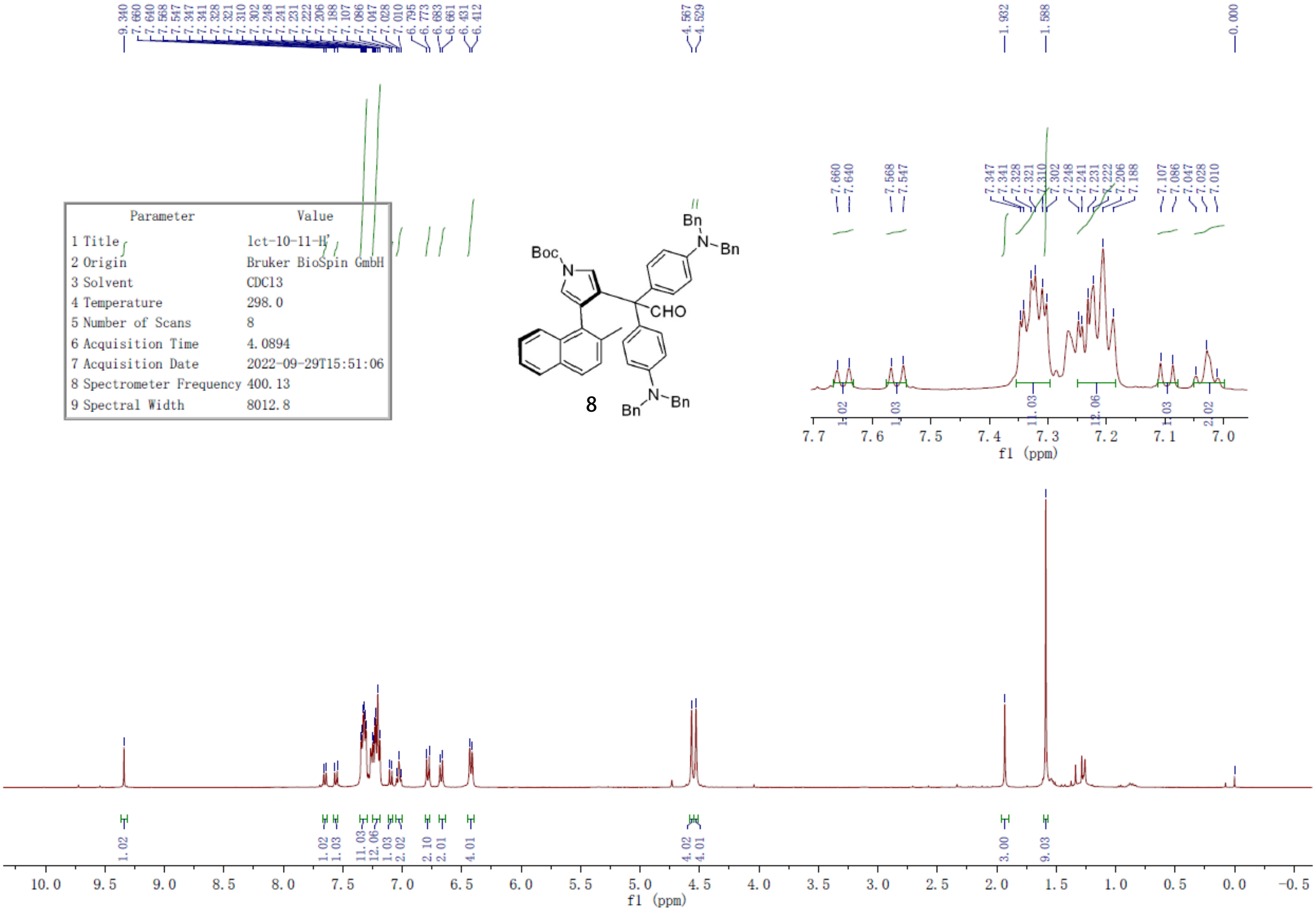

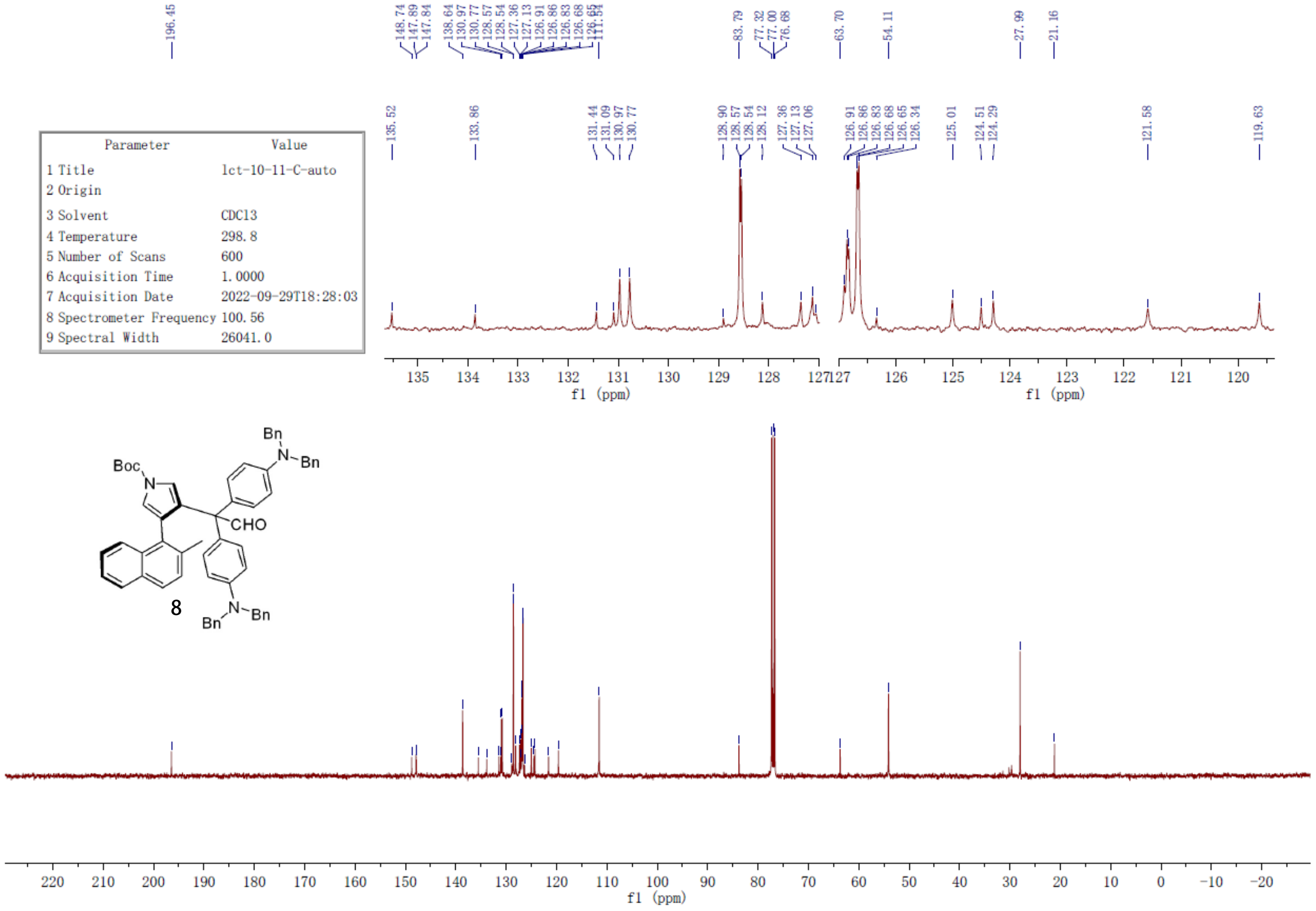

Supplementary Figure 98. ^{1}H and ^{13}C NMR spectra for **8**

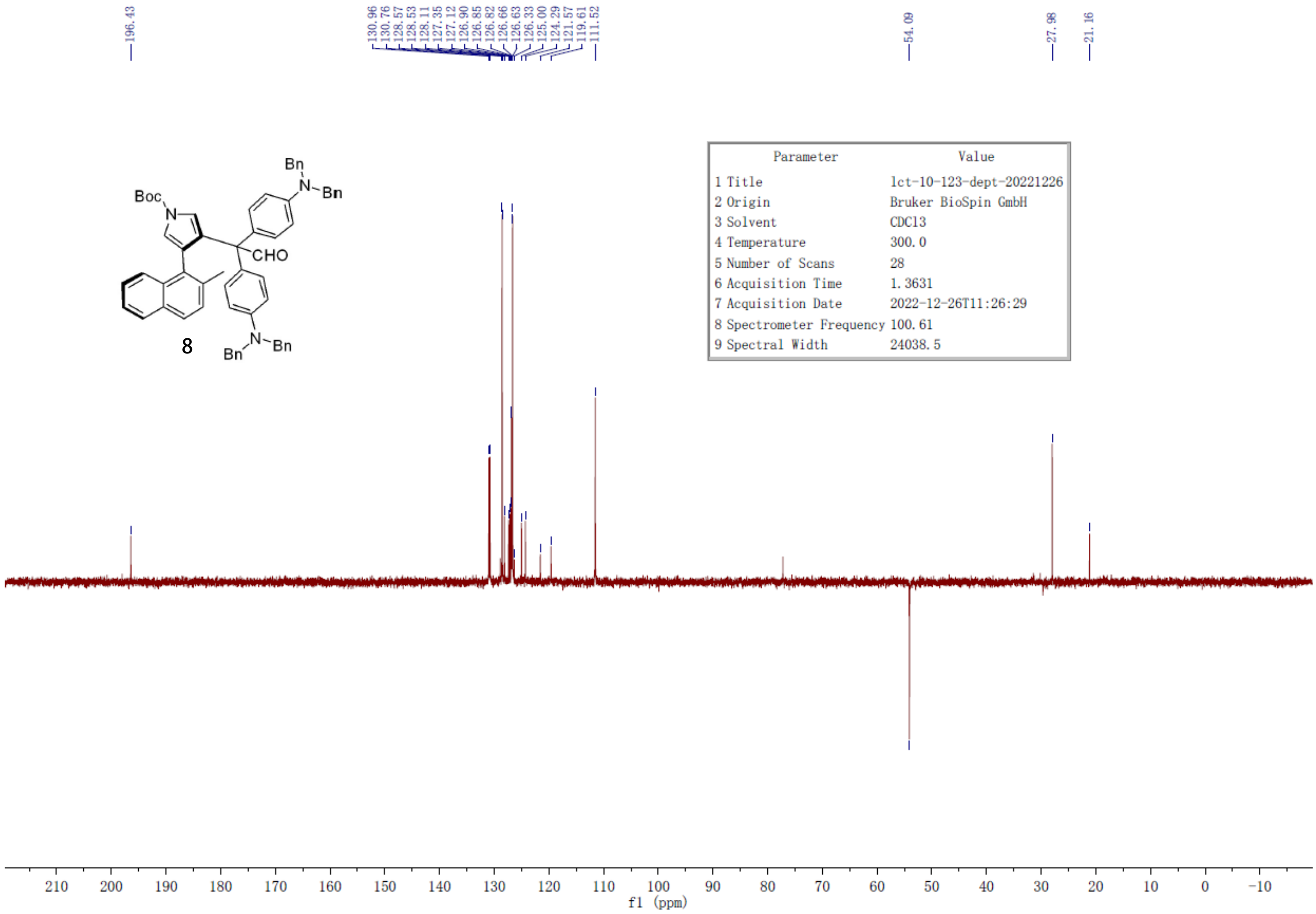

Supplementary Figure 99. DEPT 135 NMR spectra for **8**

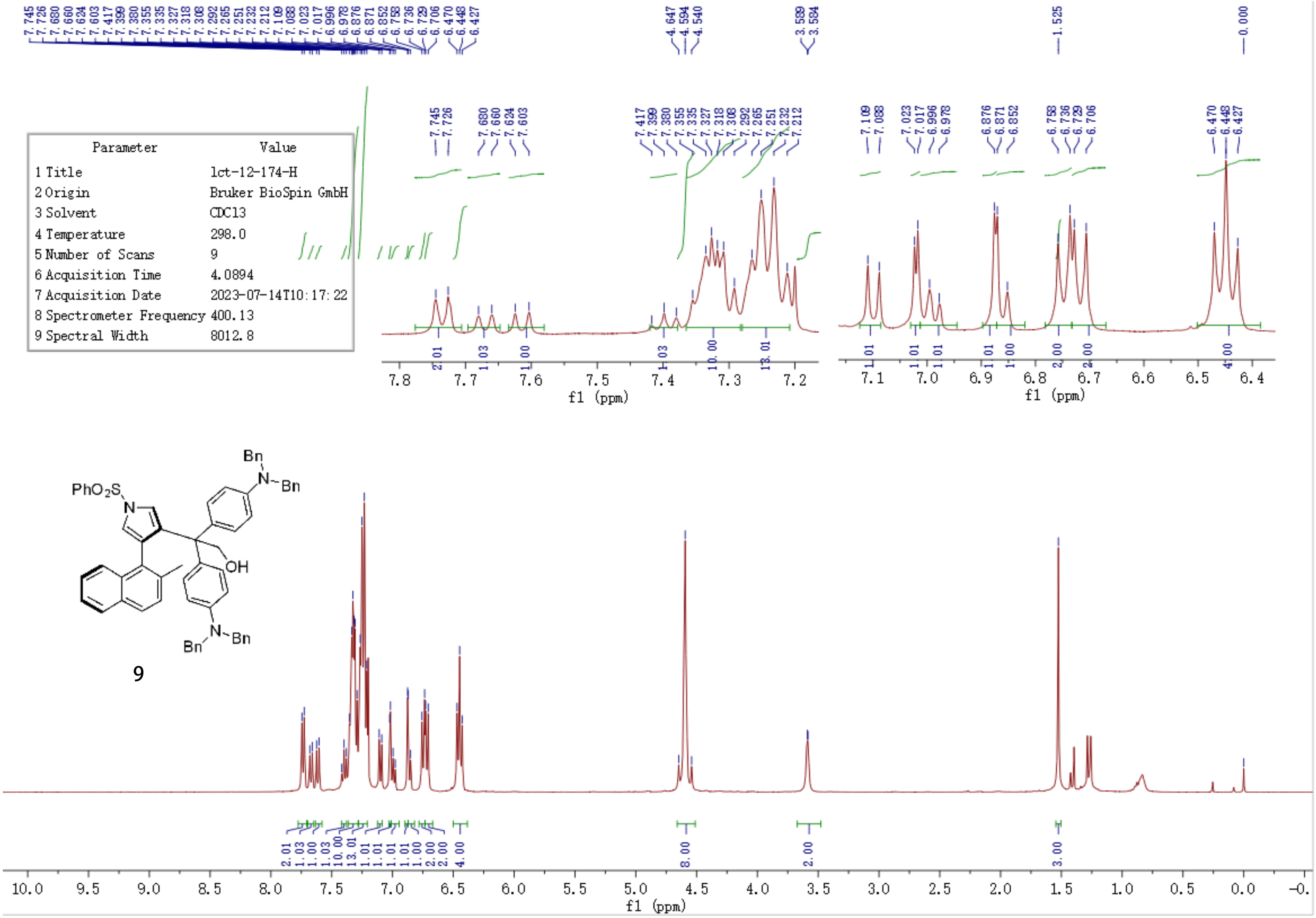

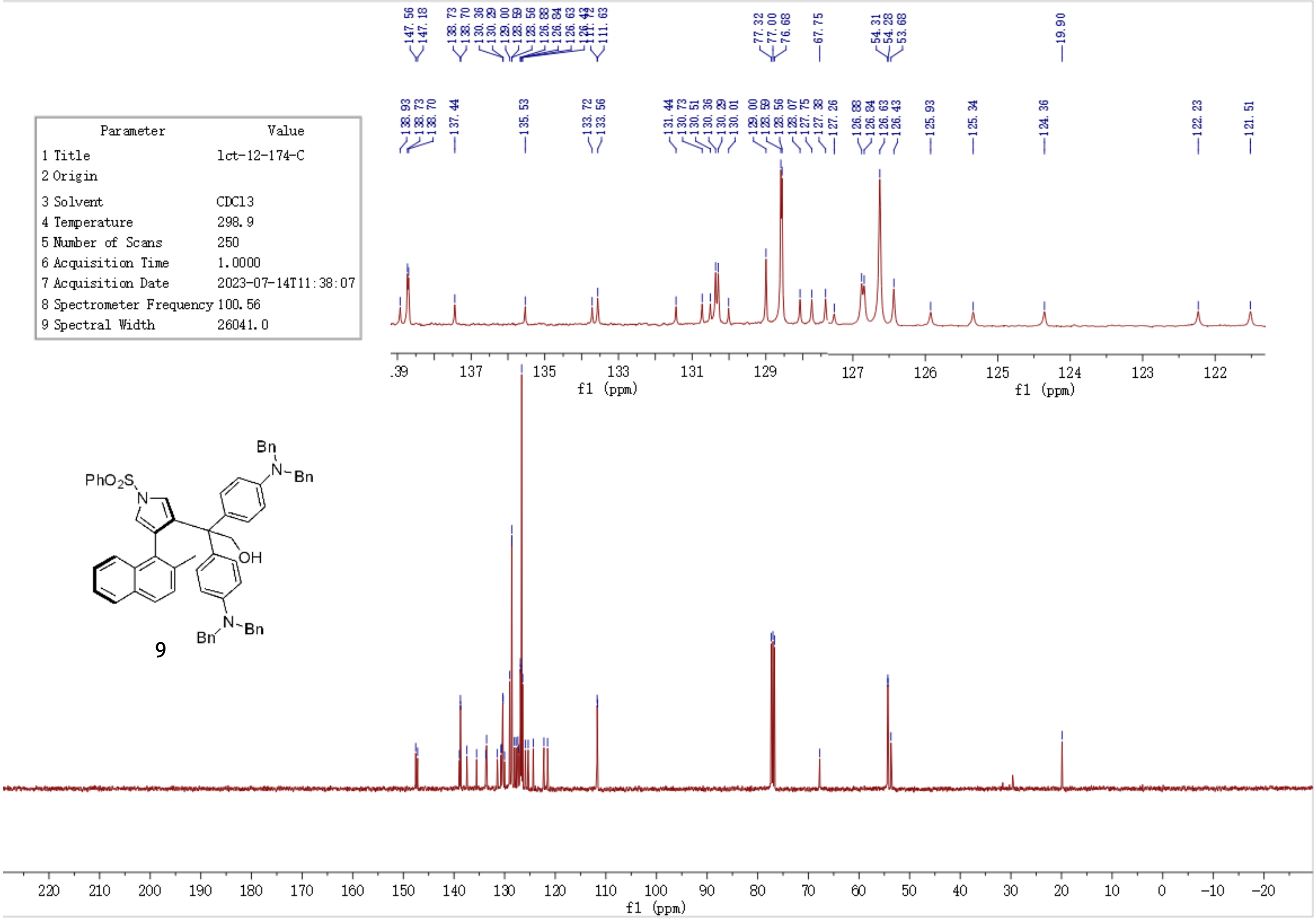

Supplementary Figure 100. ^{1}H and ^{13}C NMR spectra for **9**

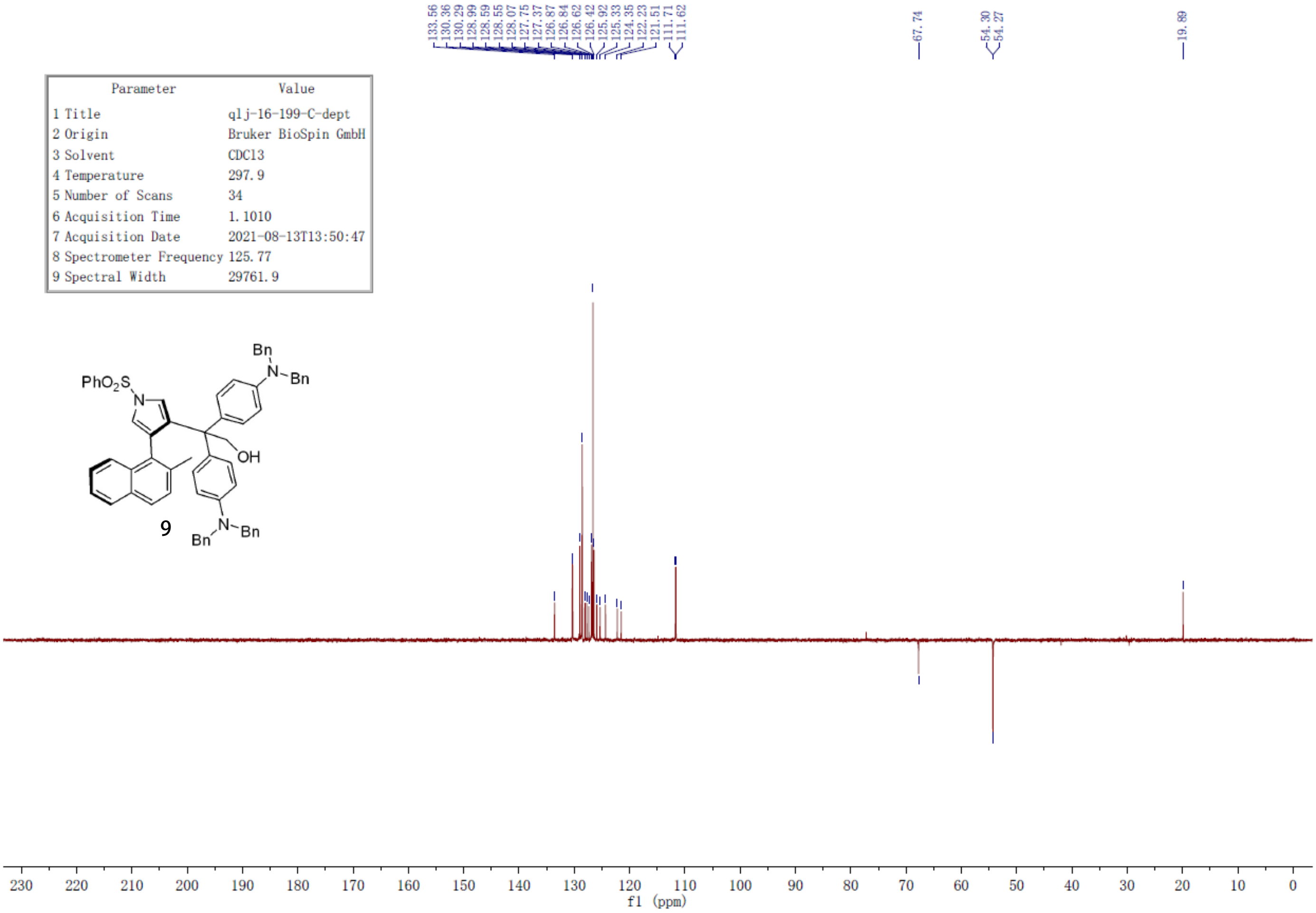

Parameter	Value
1 Title	qlj-16-199-C-dept
2 Origin	Bruker BioSpin GmbH
3 Solvent	CDCl3
4 Temperature	297.9
5 Number of Scans	34
6 Acquisition Time	1.1010
7 Acquisition Date	2021-08-13T13:50:47
8 Spectrometer Frequency	125.77
9 Spectral Width	29761.9

Supplementary Figure 101. DEPT 135 NMR spectra for **9**

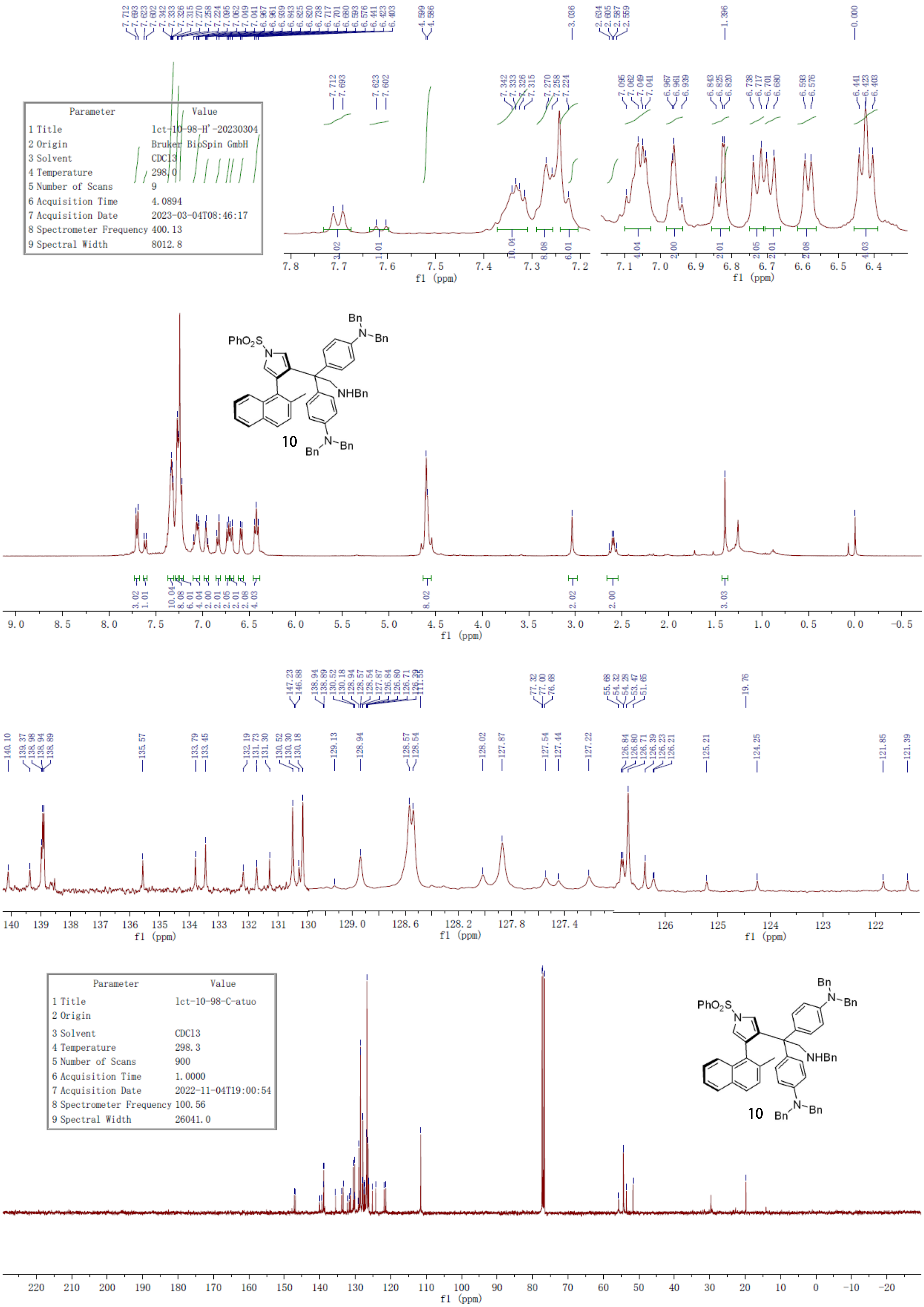

Supplementary Figure 102. ^{1}H and ^{13}C NMR spectra for **10**

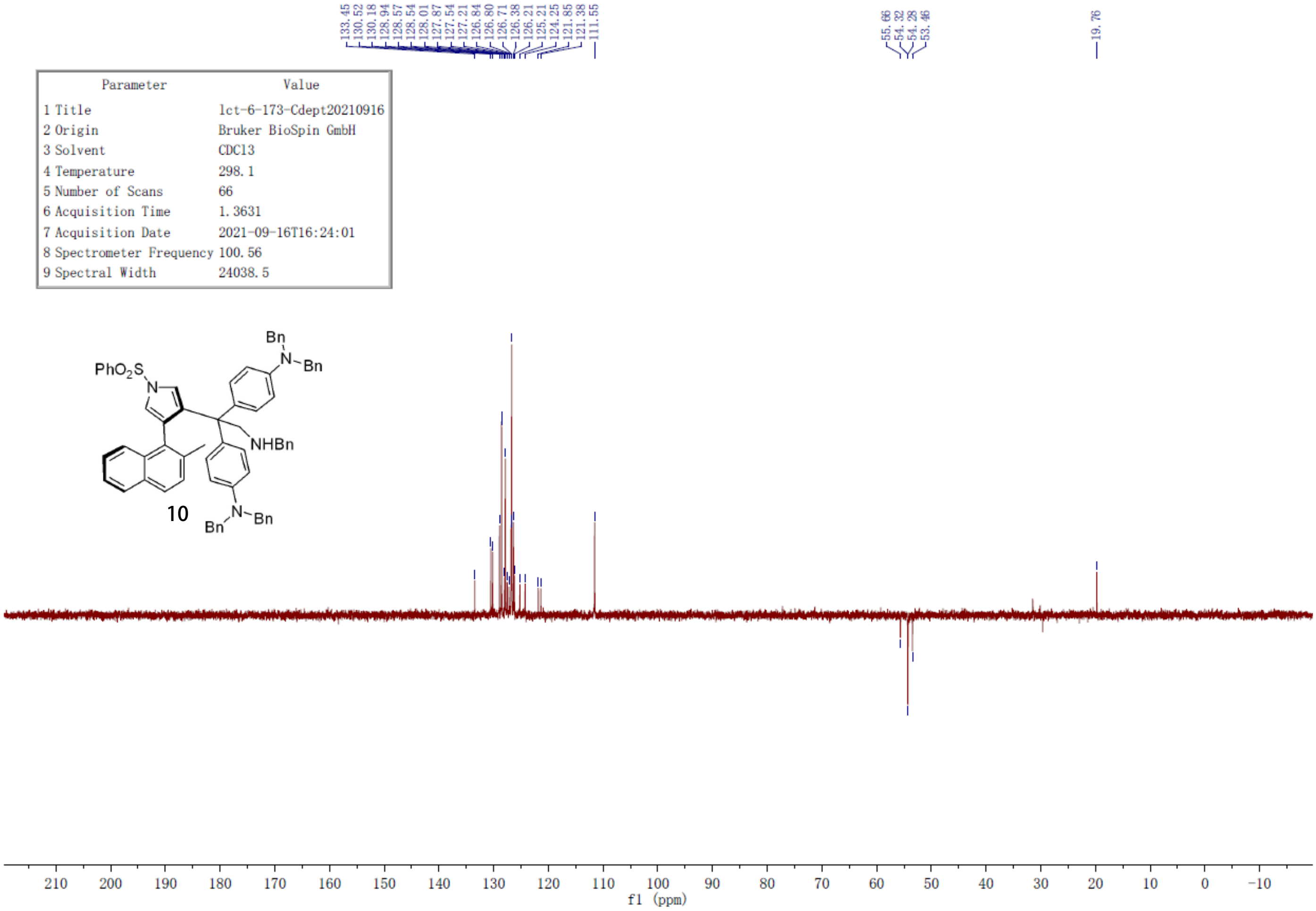

Supplementary Figure 103. DEPT 135 NMR spectra for **10**

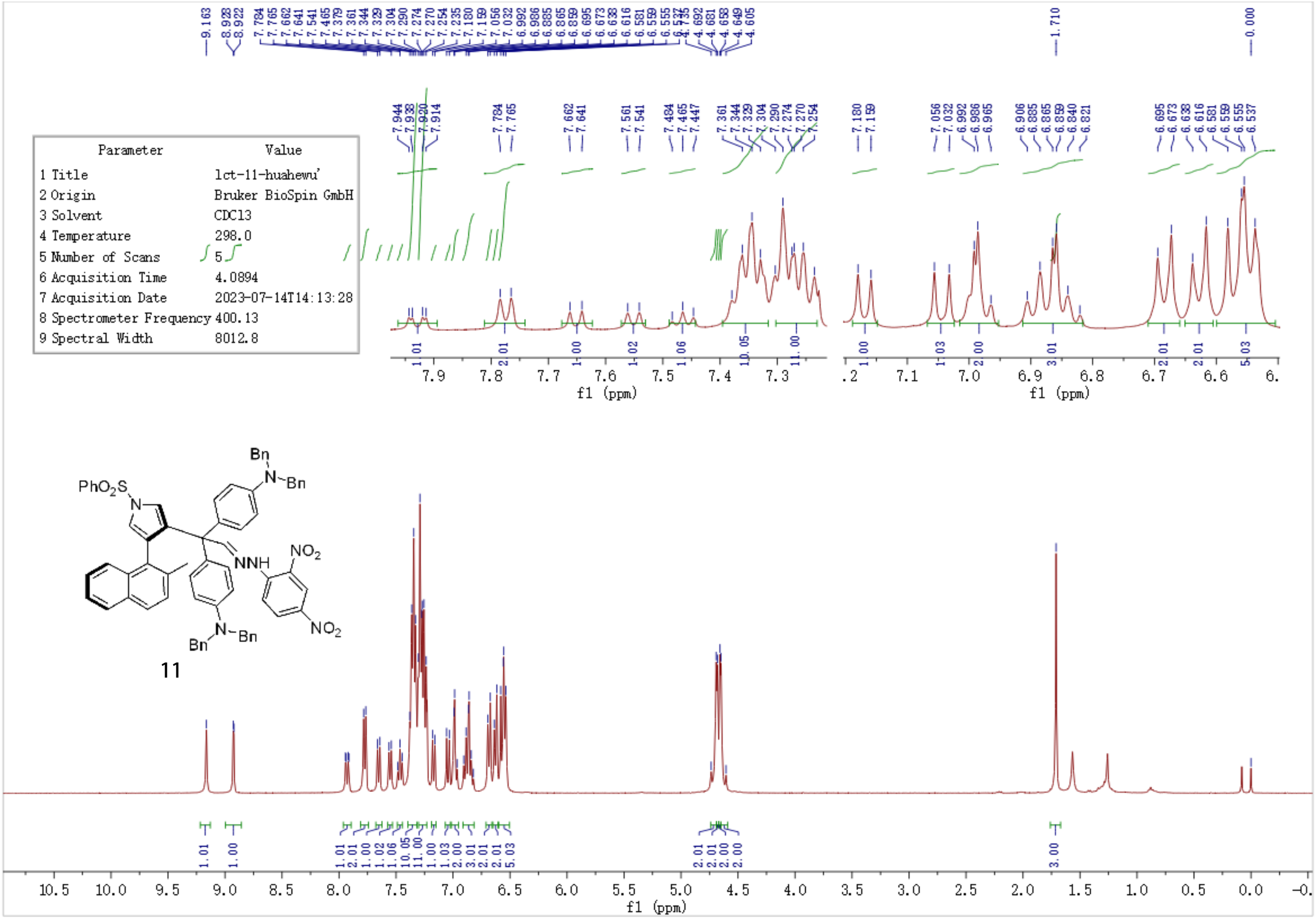

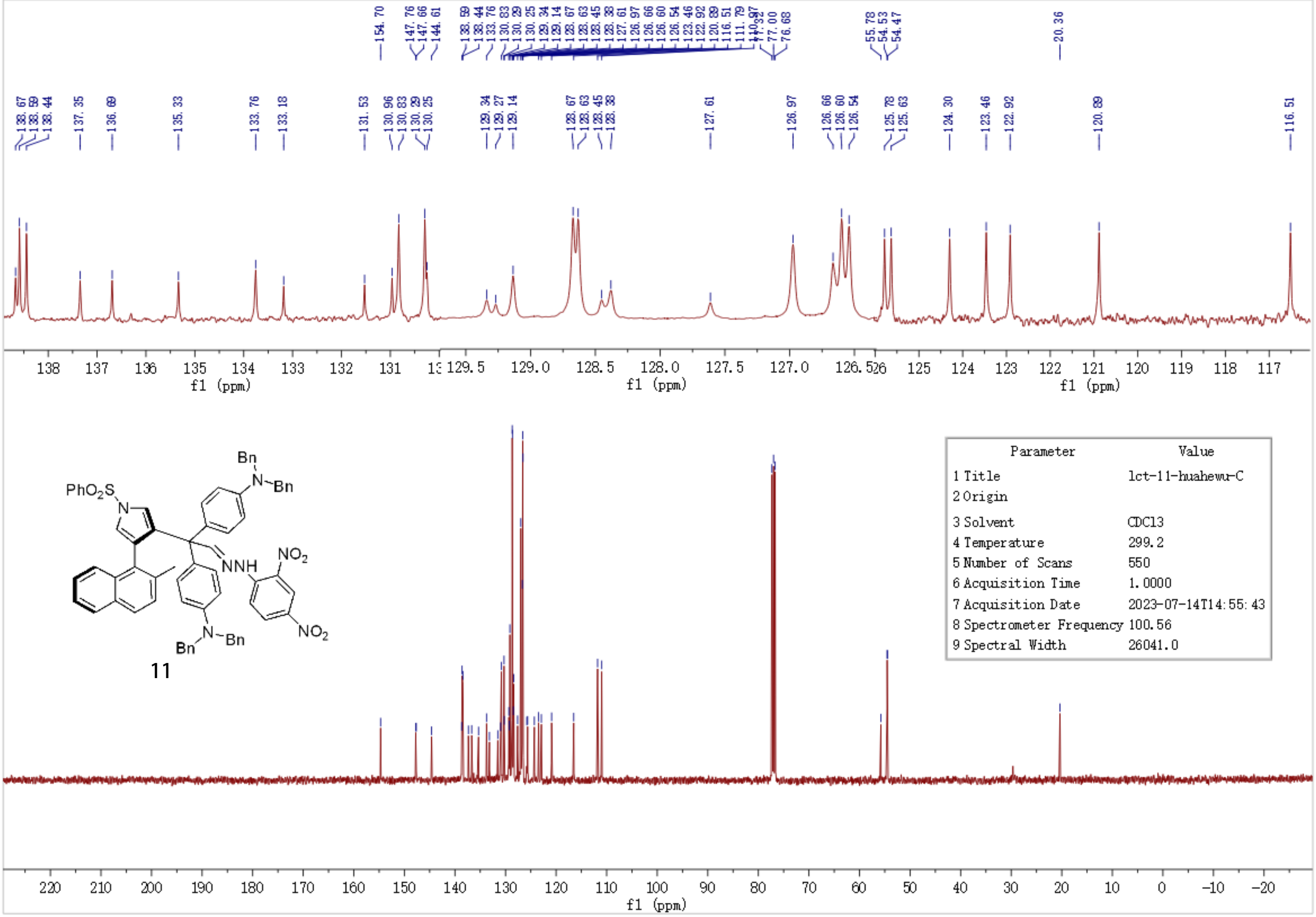

Supplementary Figure 104. ^{1}H and ^{13}C NMR spectra for **11**

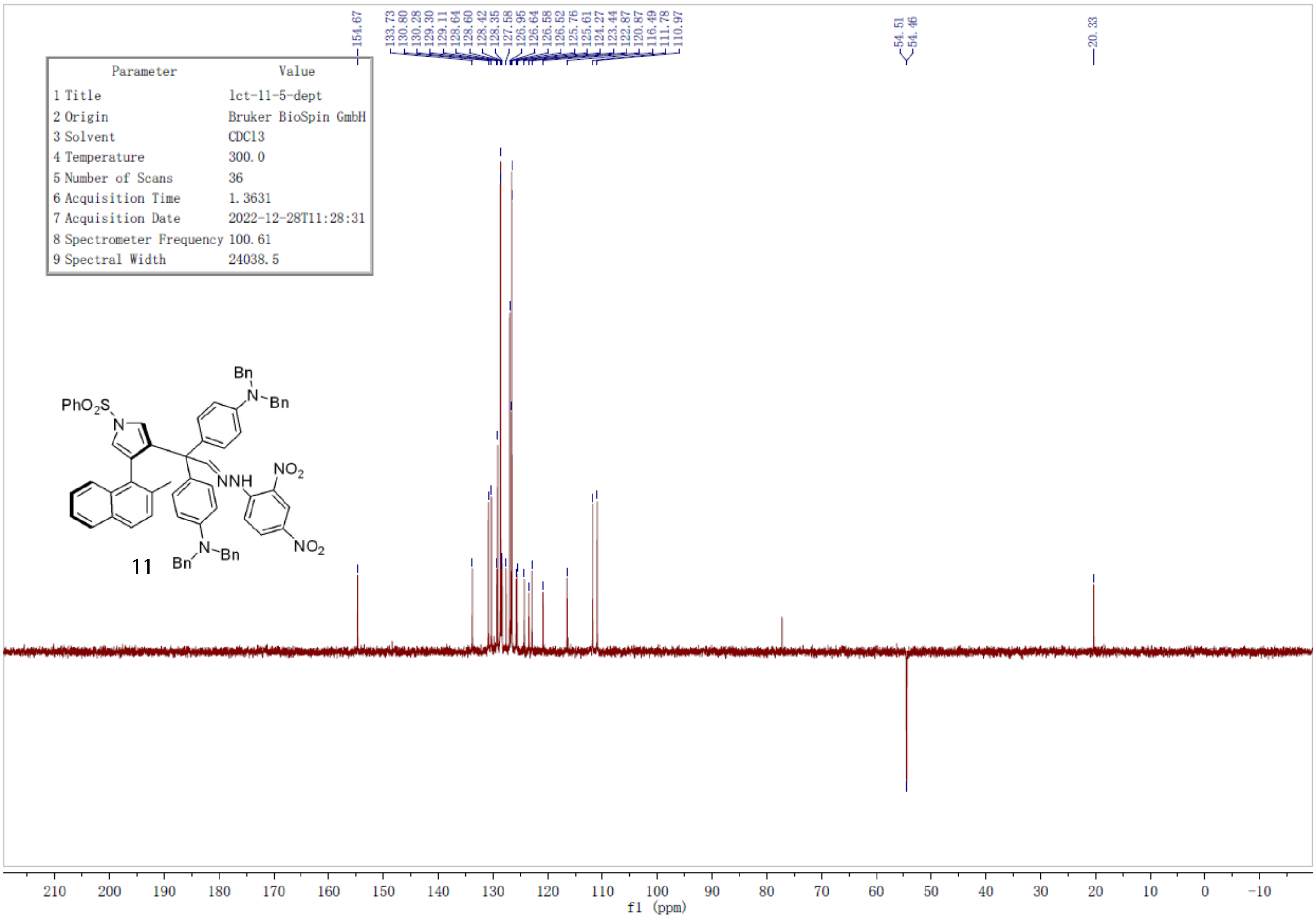

Supplementary Figure 105. DEPT 135 NMR spectra for **11**

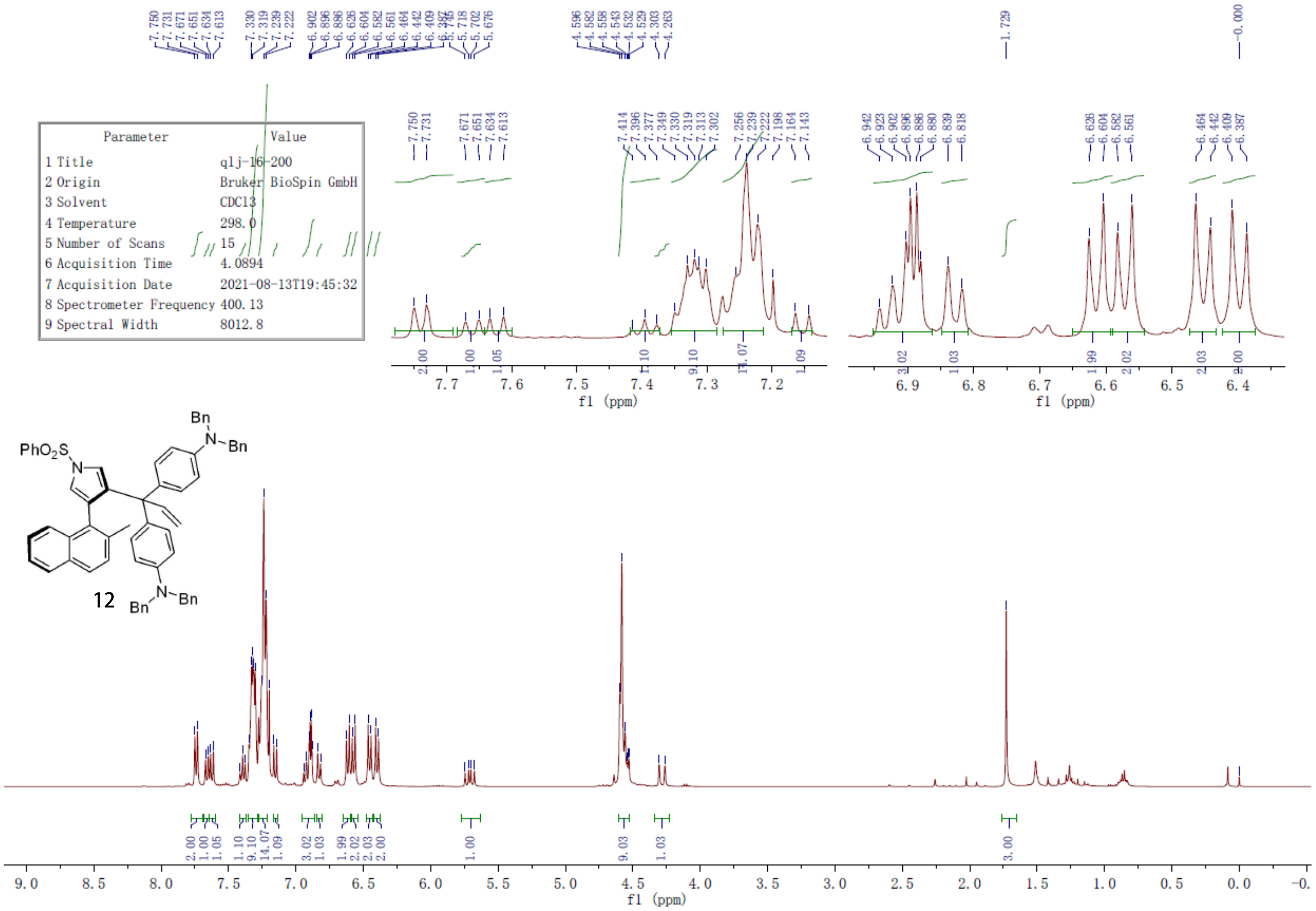

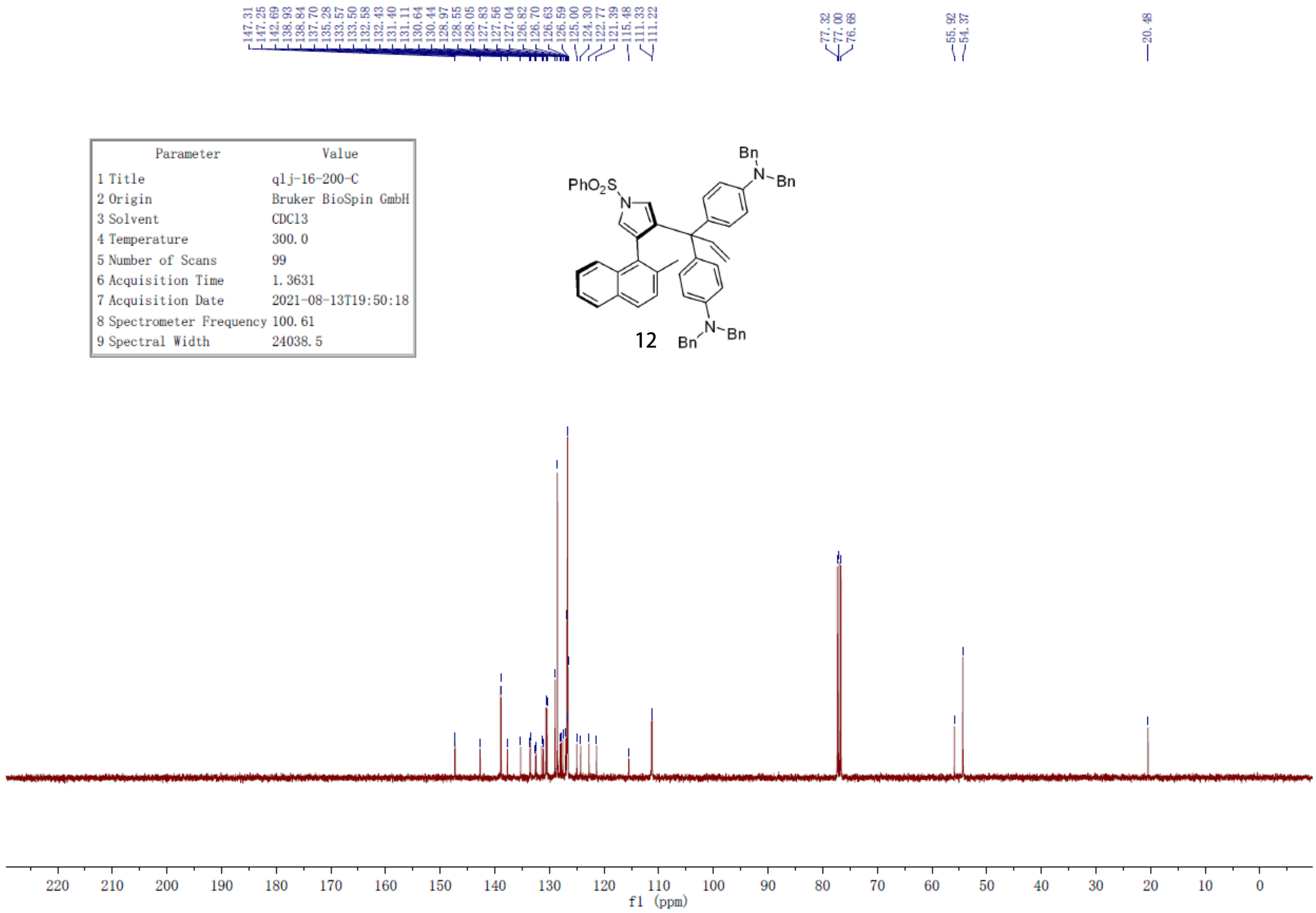

Supplementary Figure 106. ^{1}H and ^{13}C NMR spectra for **12**

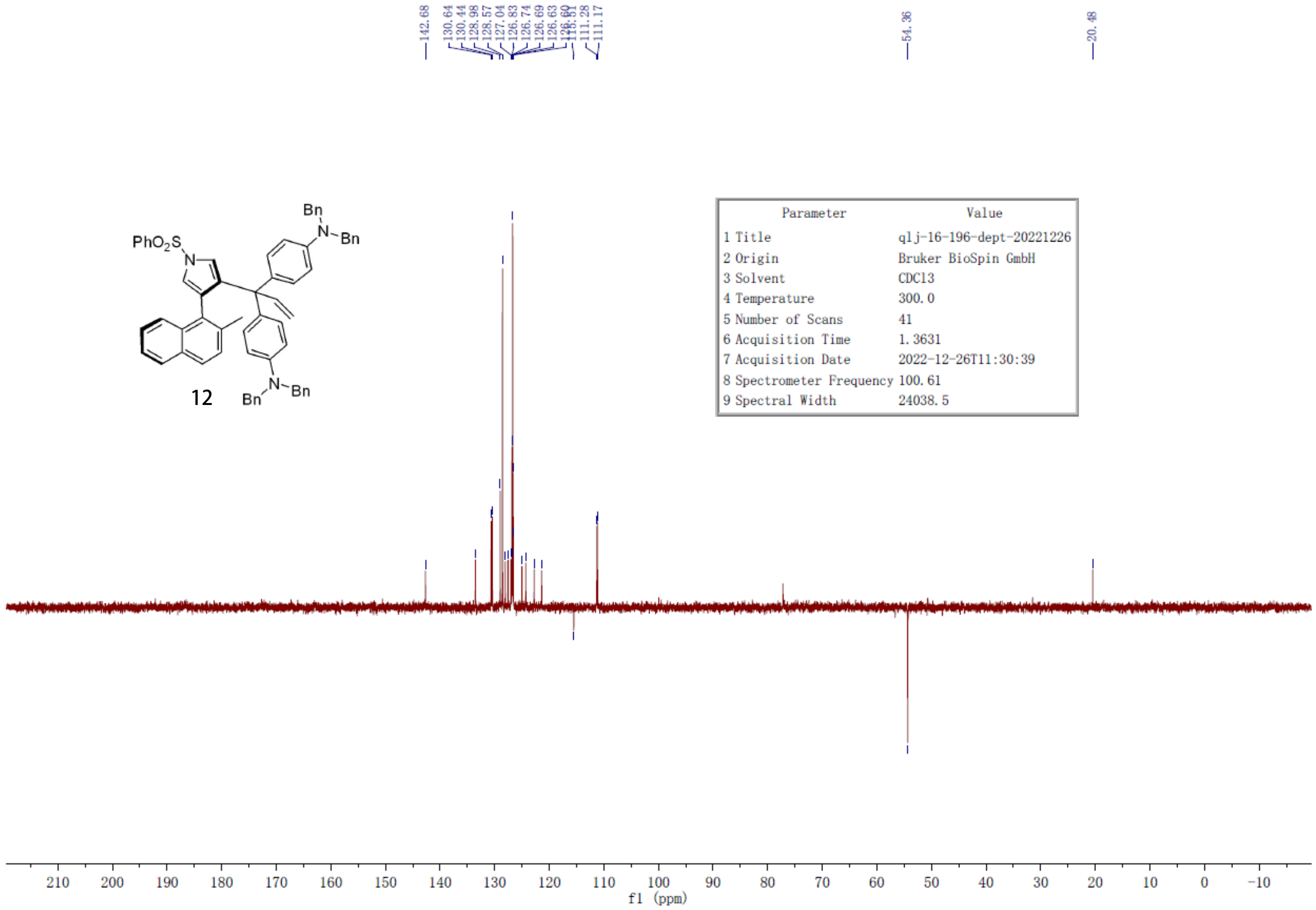

Parameter	Value
1 Title	qlj-16-196-dept-20221226
2 Origin	Bruker BioSpin GmbH
3 Solvent	CDCl3
4 Temperature	300.0
5 Number of Scans	41
6 Acquisition Time	1.3631
7 Acquisition Date	2022-12-26T11:30:39
8 Spectrometer Frequency	100.61
9 Spectral Width	24038.5

Supplementary Figure 107. DEPT 135 NMR spectra for **12**

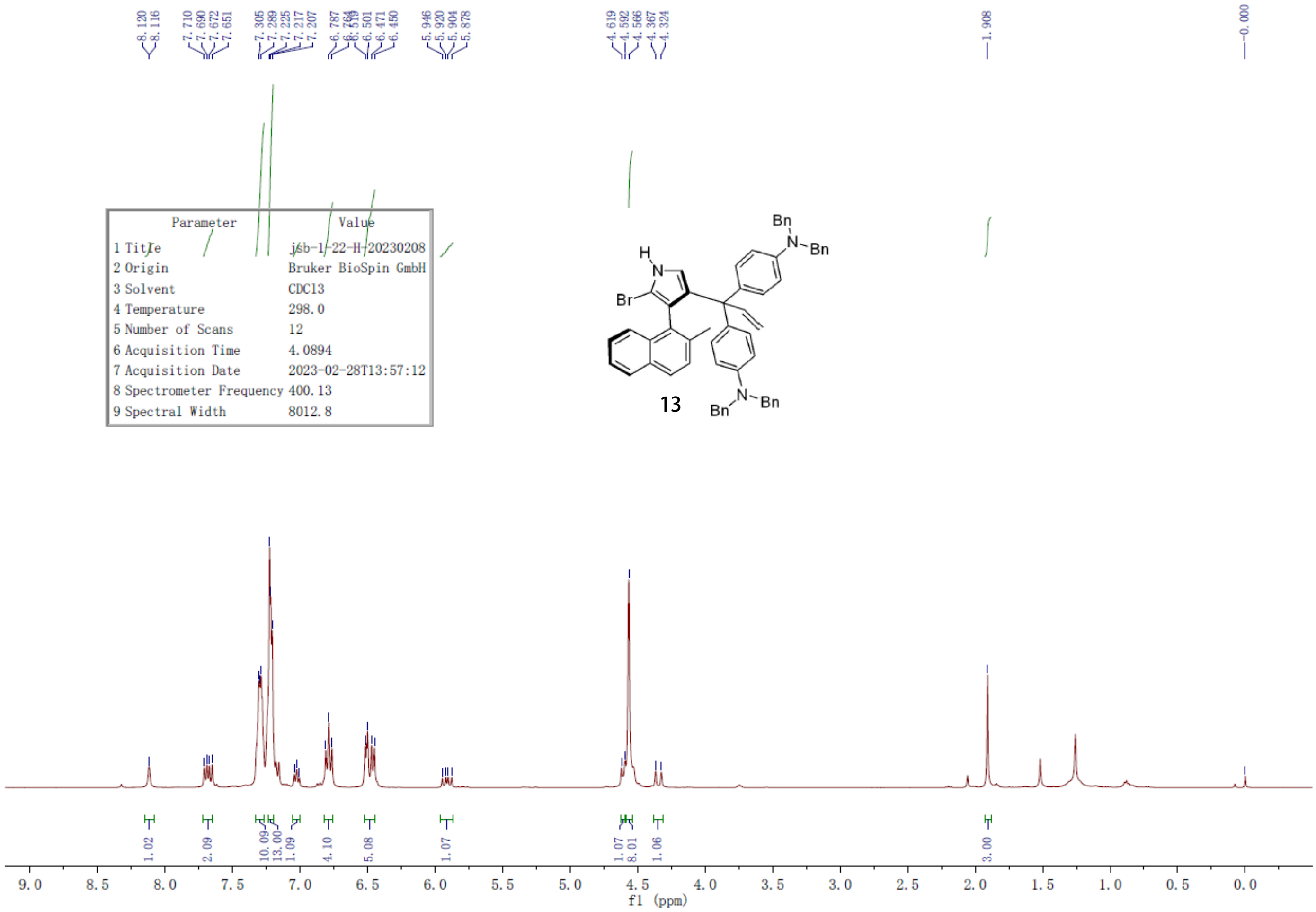

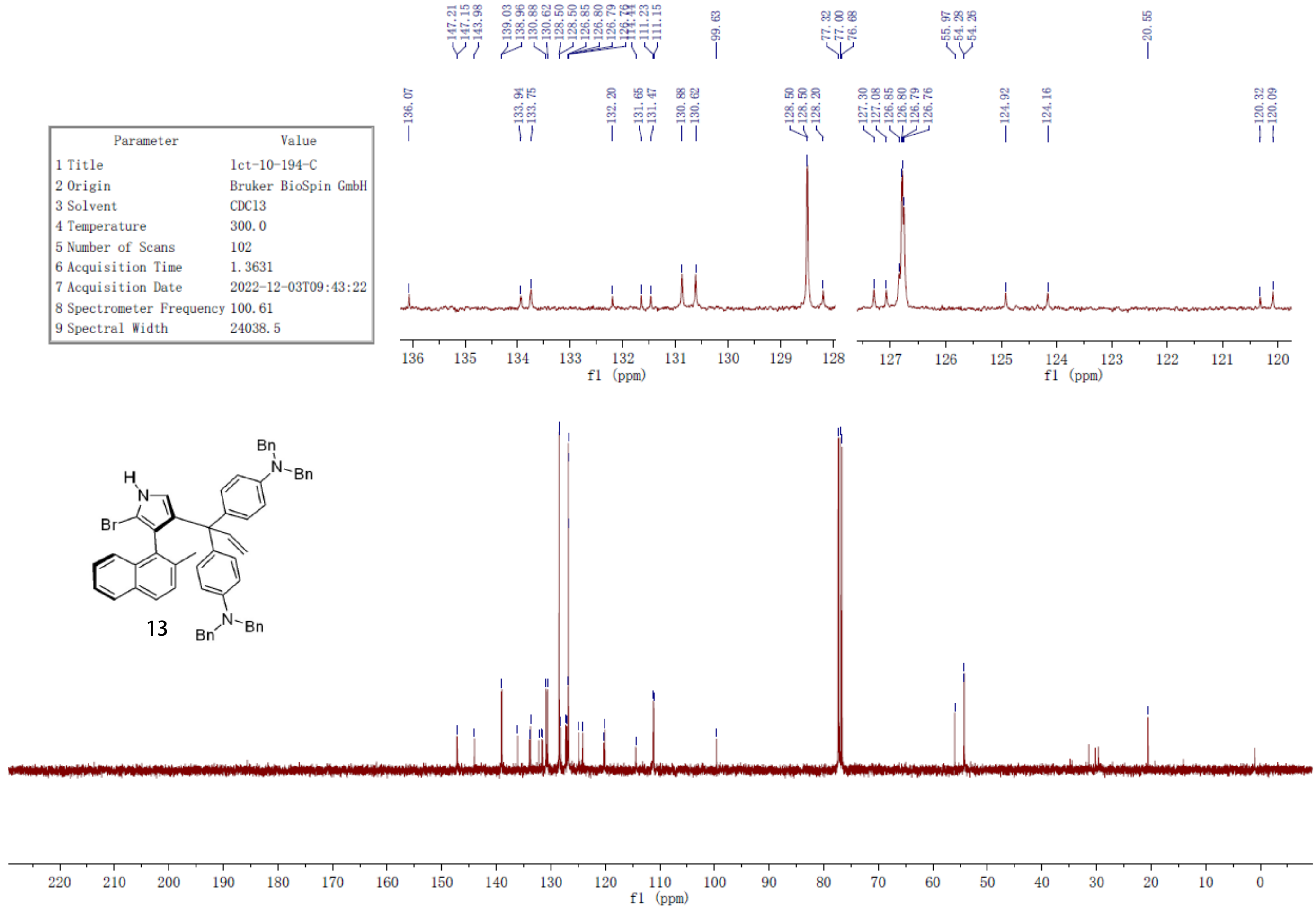

Supplementary Figure 108. ^{1}H and ^{13}C NMR spectra for **13**

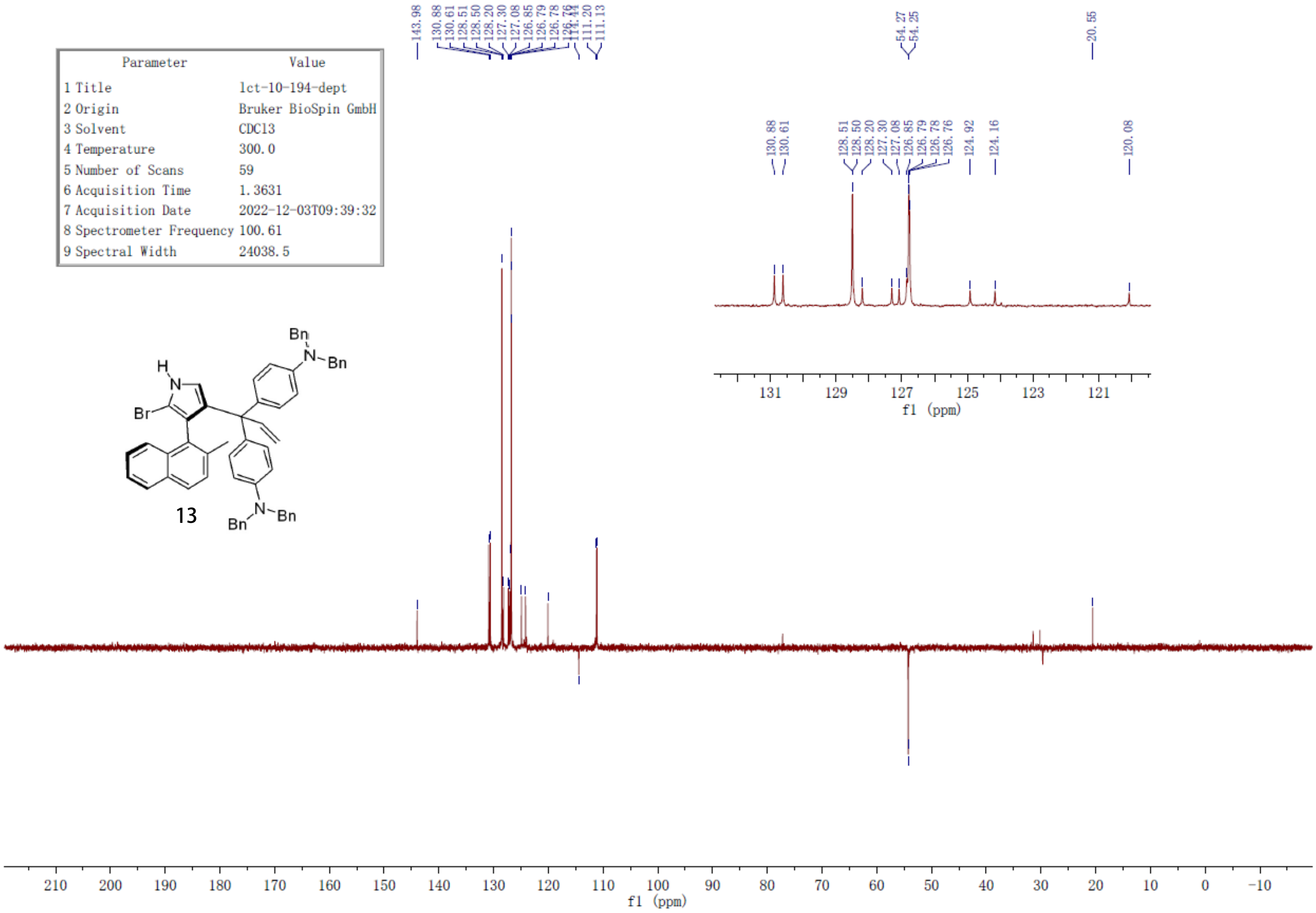

Supplementary Figure 109. DEPT 135 NMR spectra for **13**

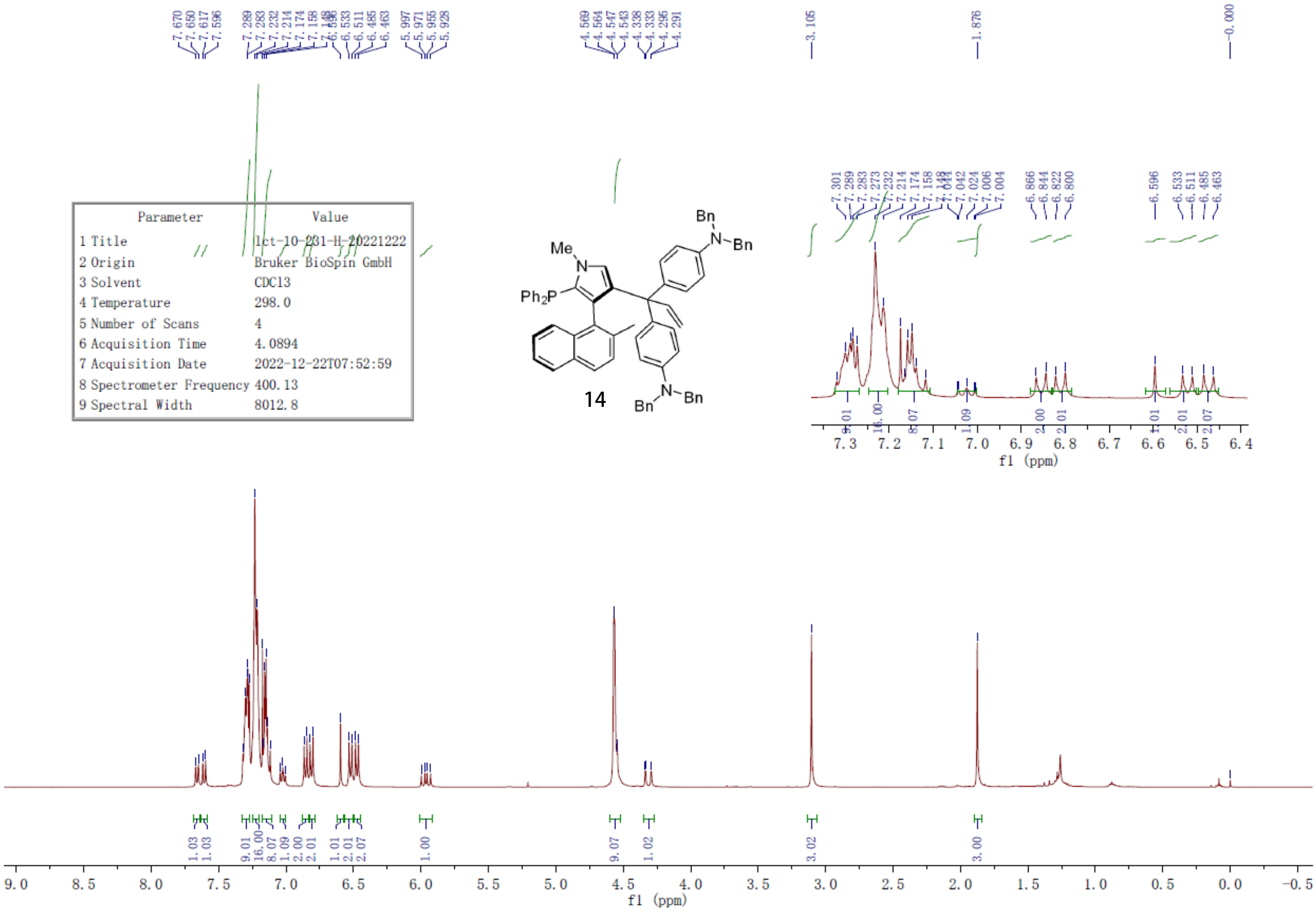

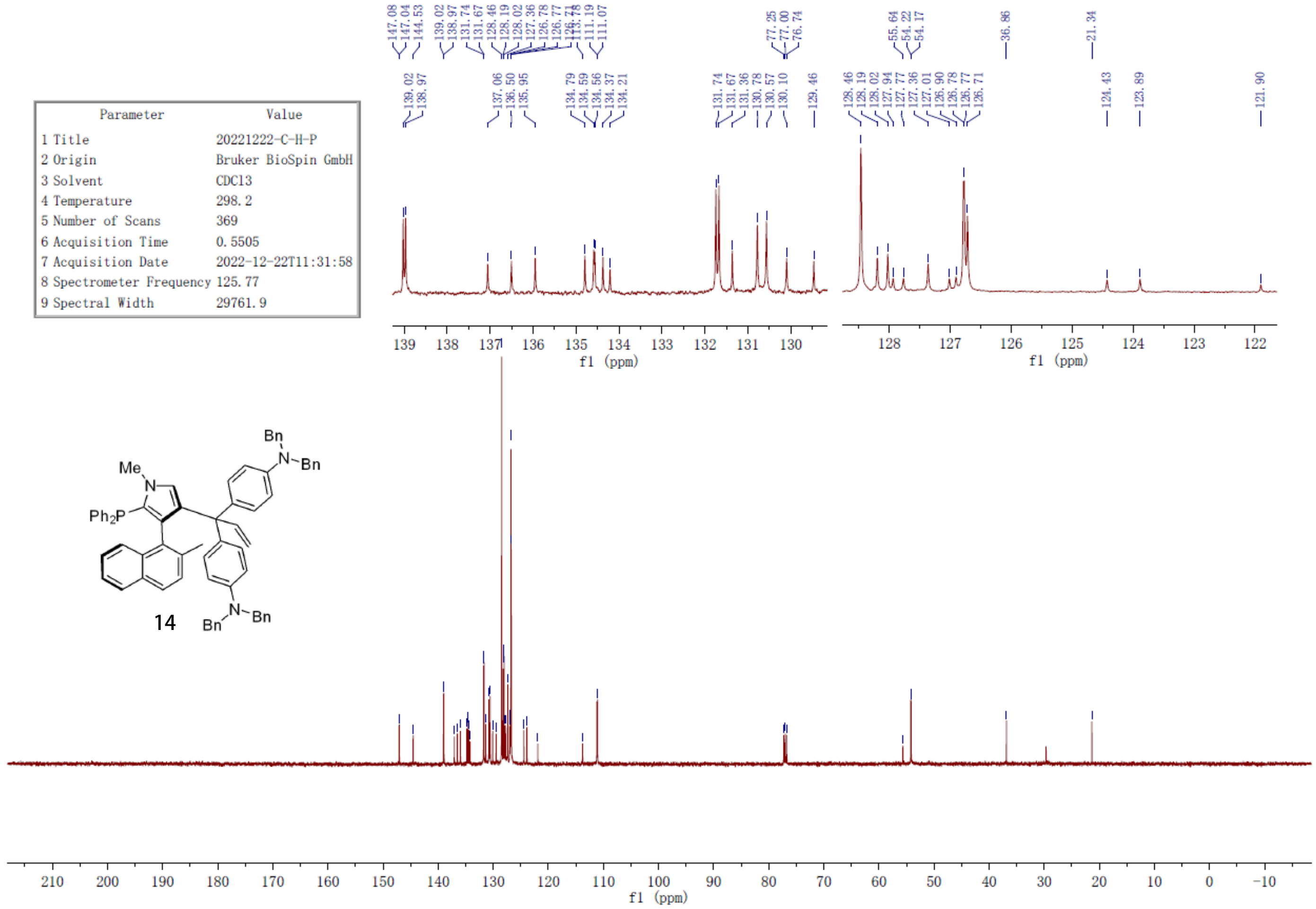

Supplementary Figure 110. ^{1}H and ^{13}C NMR spectra for **14**

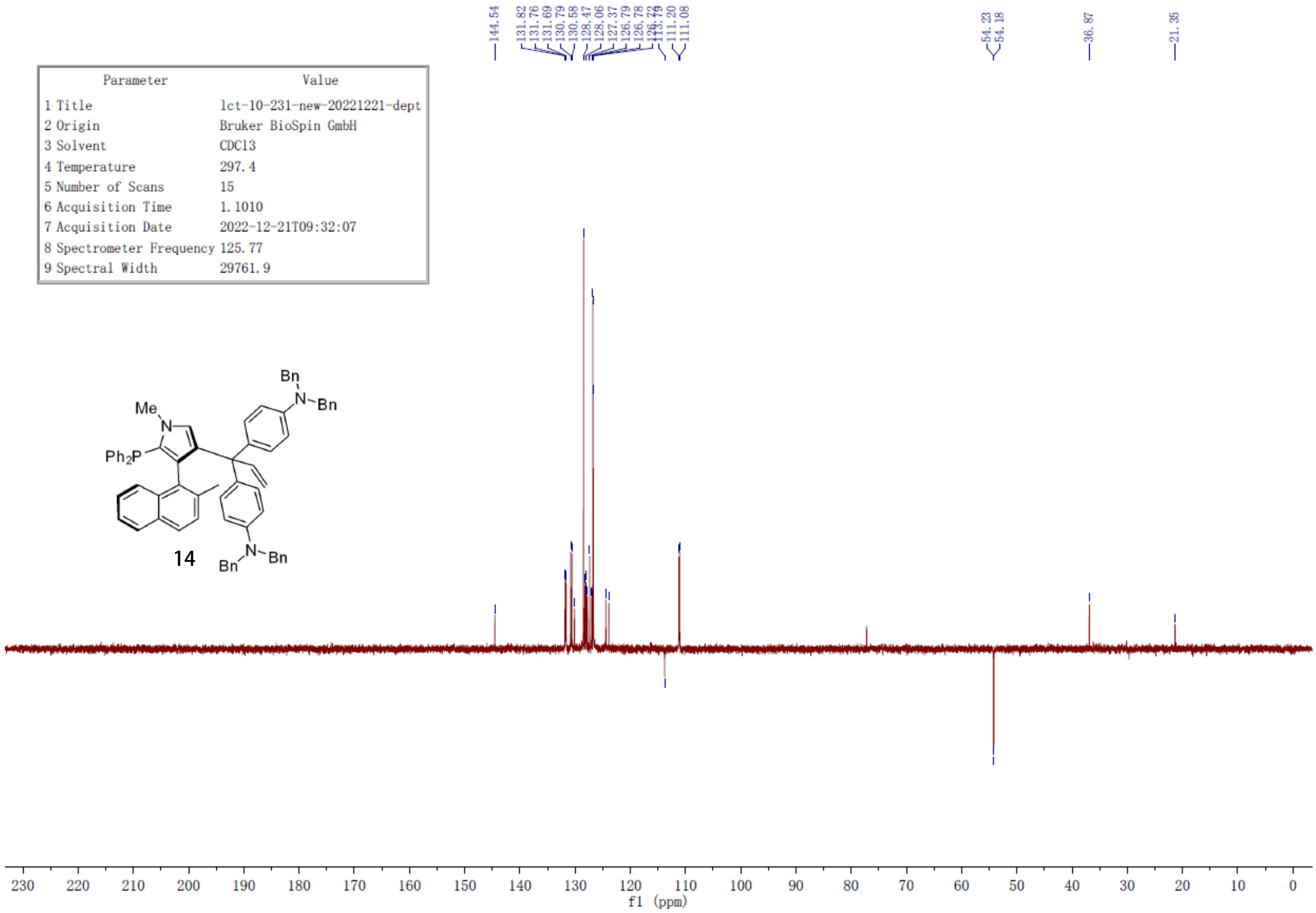

Parameter	Value
1 Title	lct-10-231-new-20221221-dept
2 Origin	Bruker BioSpin GmbH
3 Solvent	CDCl3
4 Temperature	297.4
5 Number of Scans	15
6 Acquisition Time	1.1010
7 Acquisition Date	2022-12-21T09:32:07
8 Spectrometer Frequency	125.77
9 Spectral Width	29761.9

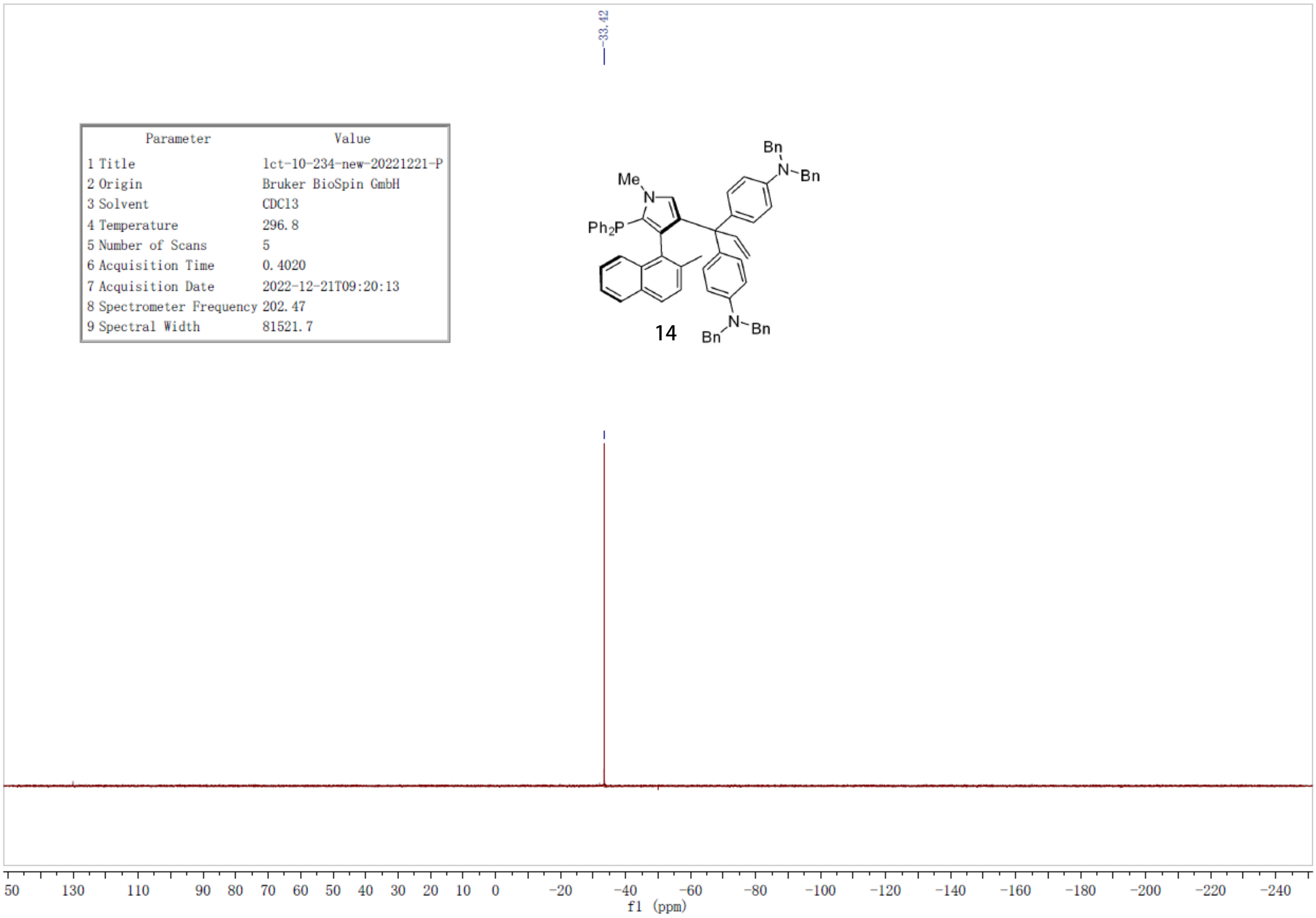

Parameter	Value
1 Title	lct-10-234-new-20221221-P
2 Origin	Bruker BioSpin GmbH
3 Solvent	CDCl3
4 Temperature	296.8
5 Number of Scans	5
6 Acquisition Time	0.4020
7 Acquisition Date	2022-12-21T09:20:13
8 Spectrometer Frequency	202.47
9 Spectral Width	81521.7

Supplementary Figure 111. DEPT 135 and ^{31}P NMR spectra for **14**

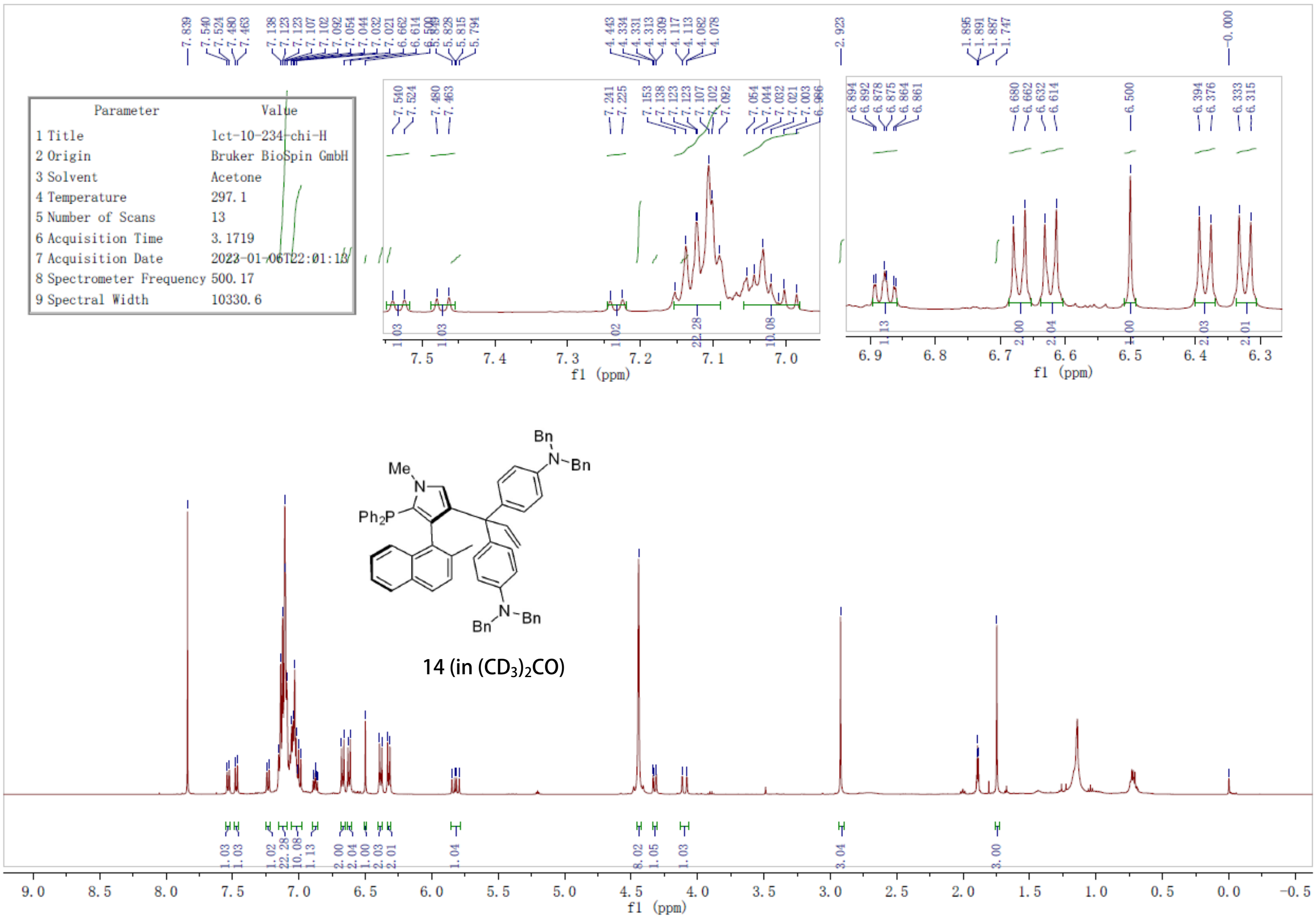

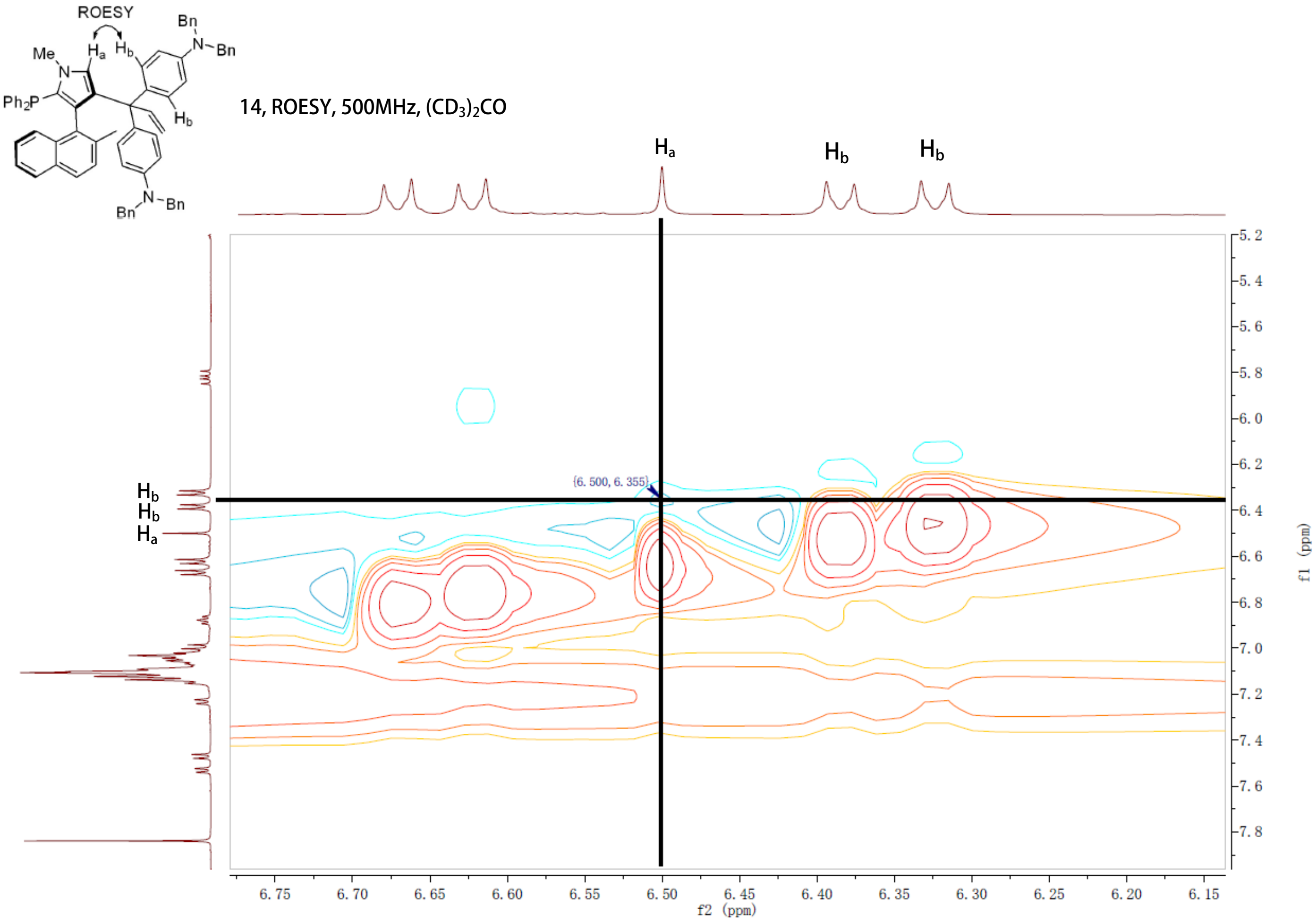

Supplementary Figure 112. ^{1}H and ROESY NMR (in $(CD_3)_2CO$) spectra for **14**

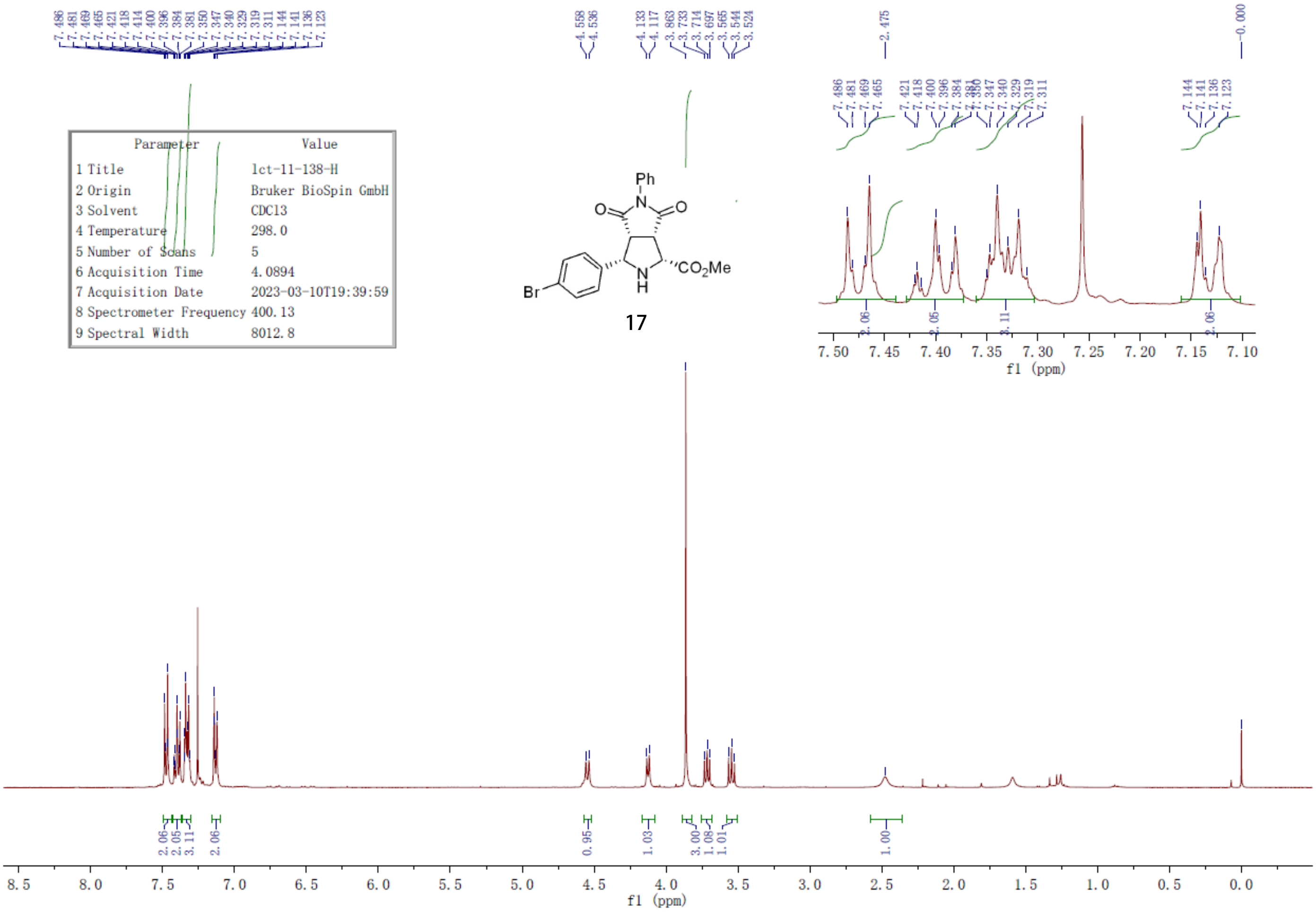

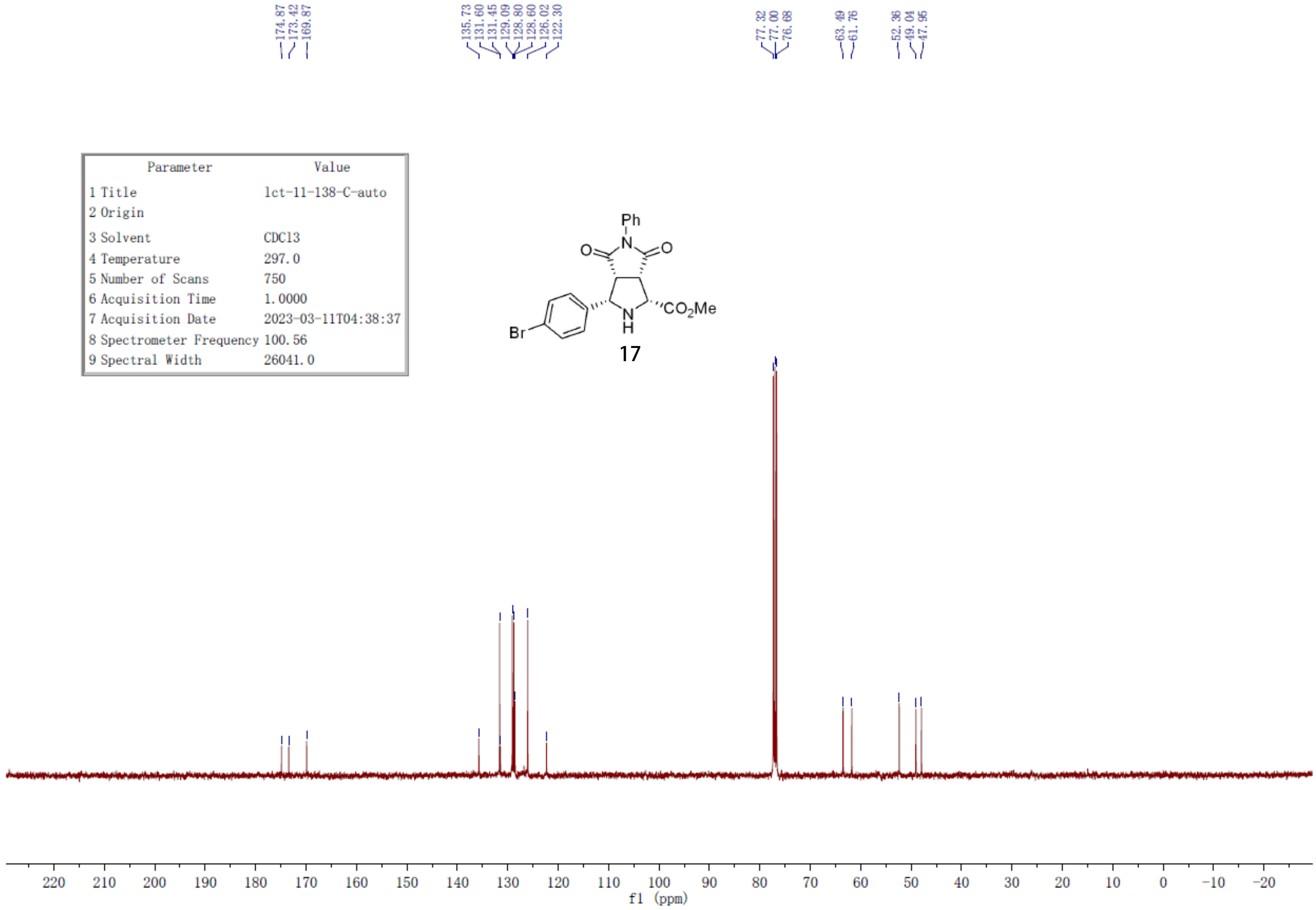

Supplementary Figure 113. ^{1}H and ^{13}C NMR spectra for **17**

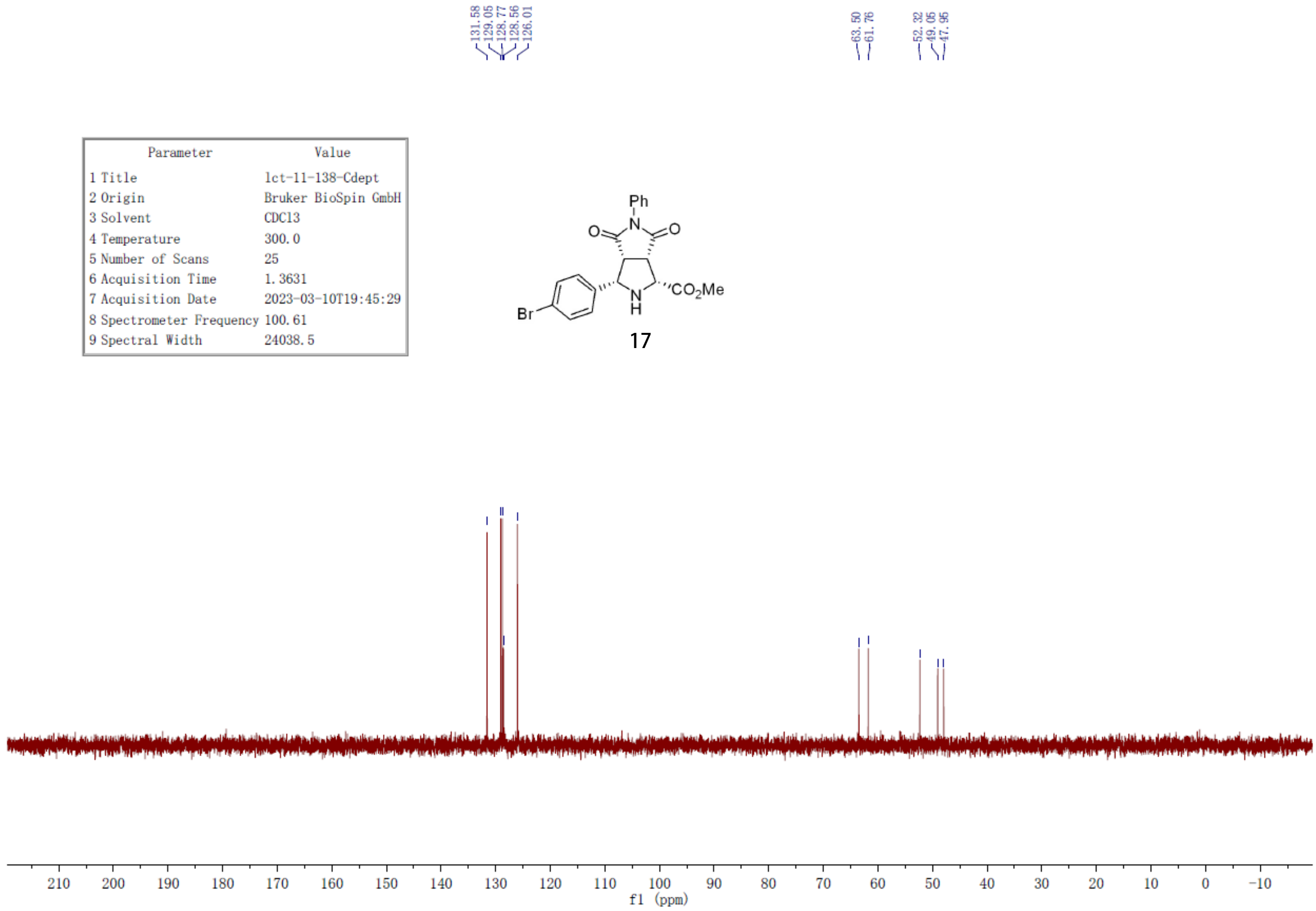

Supplementary Figure 114. DEPT 135 NMR spectra for **17**

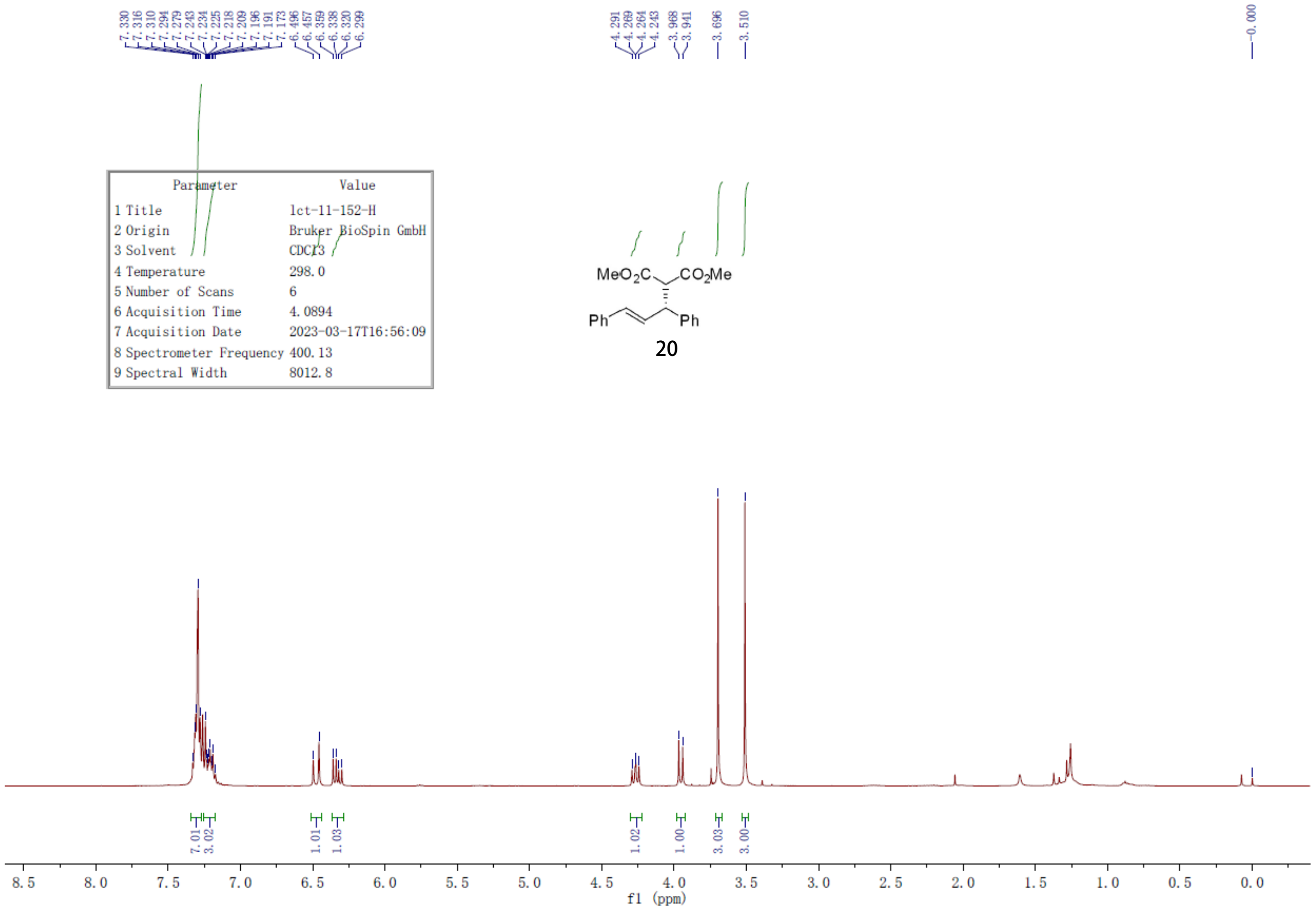

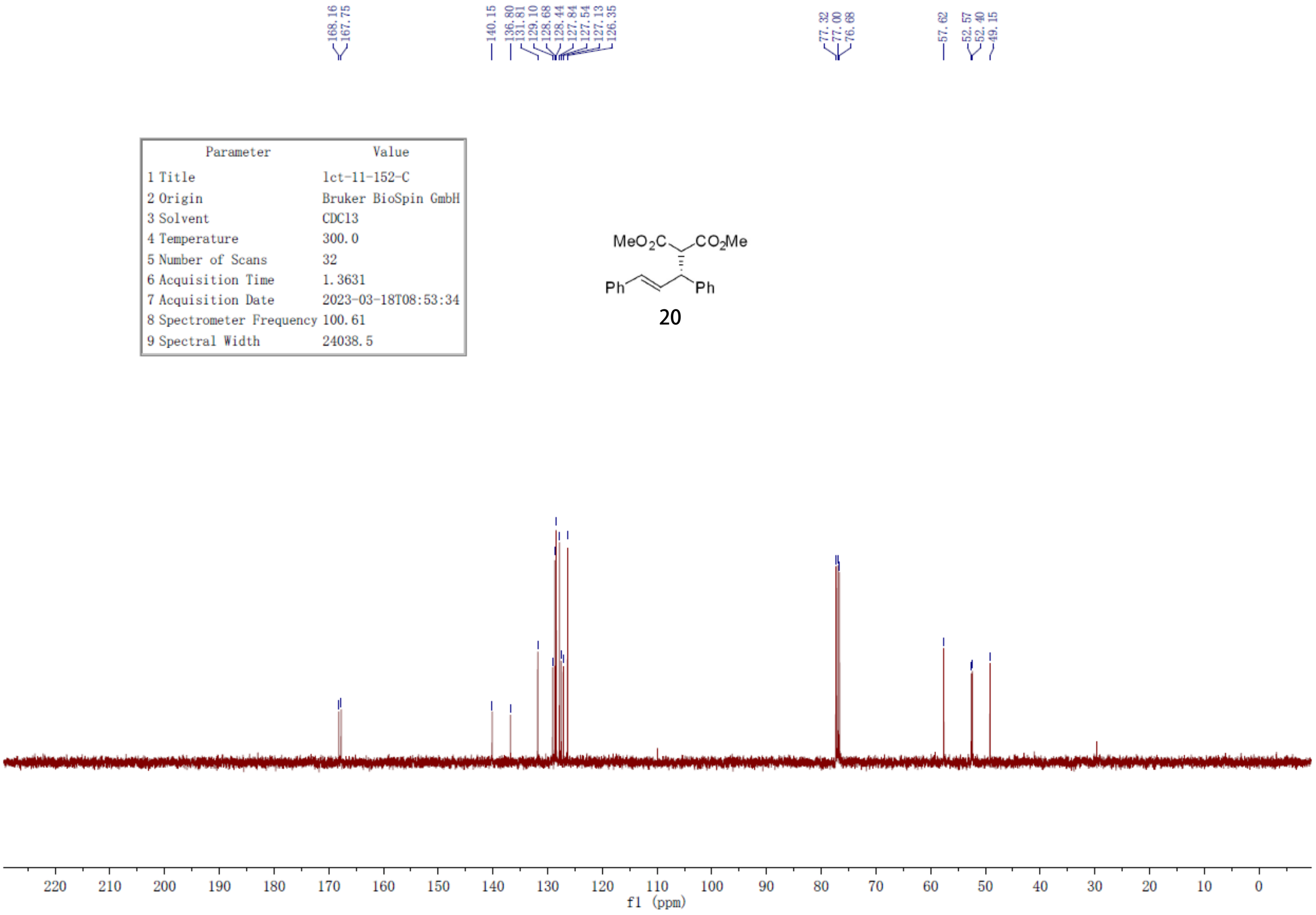

Supplementary Figure 115. ^{1}H and ^{13}C NMR spectra for **20**

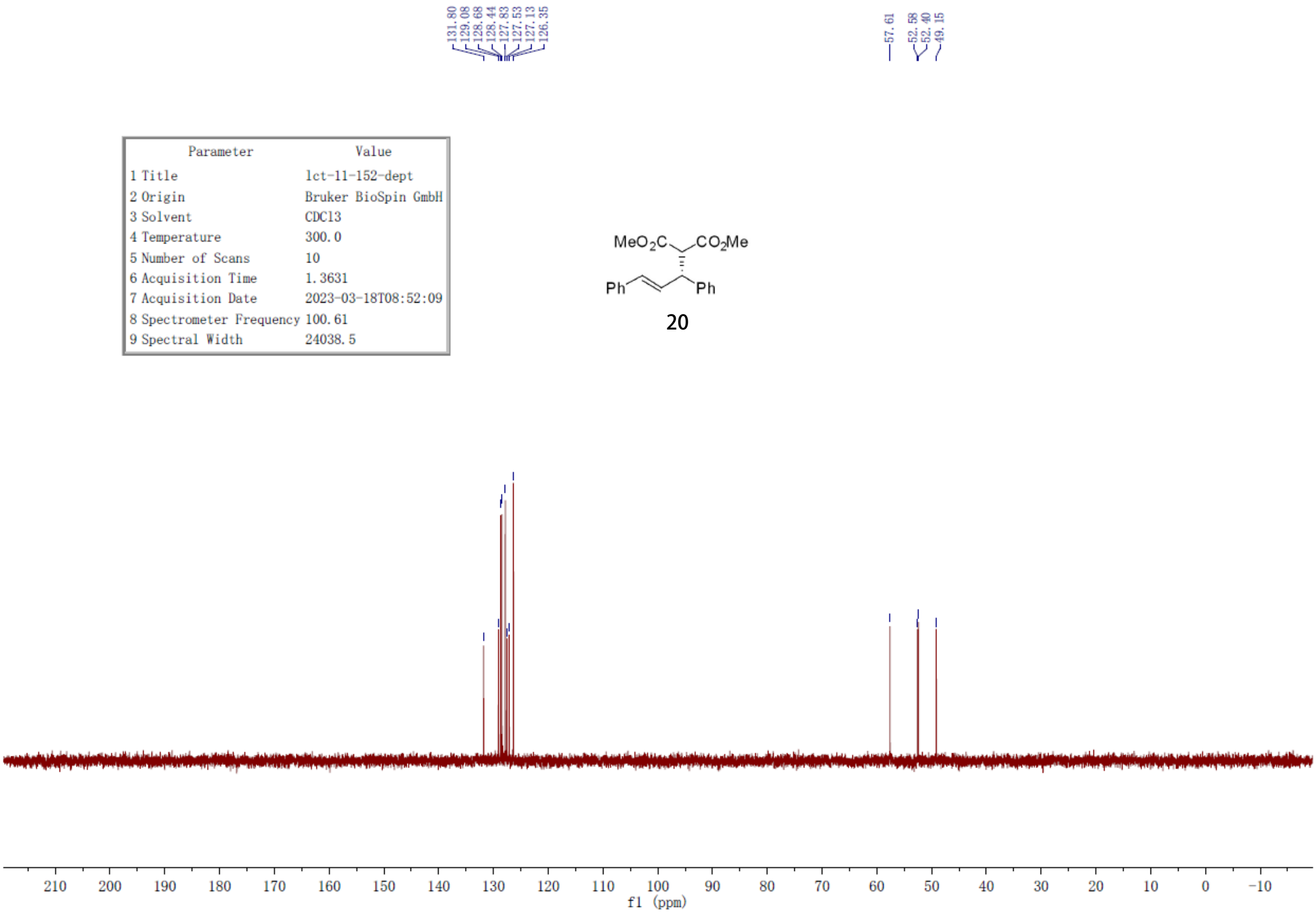

Supplementary Figure 116. DEPT 135 NMR spectra for **20**

1.9 HPLC Chromatograms

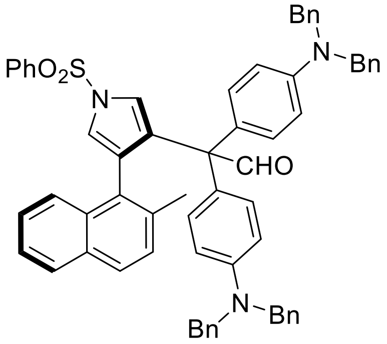

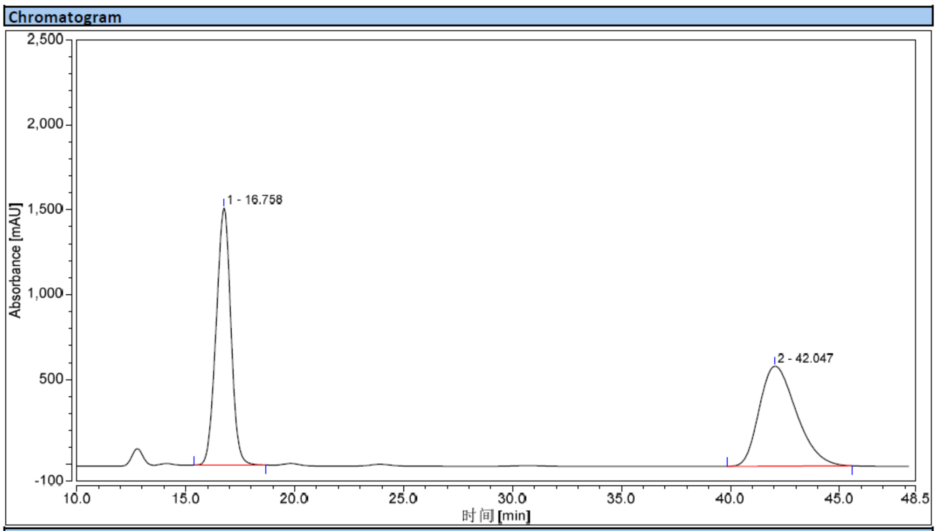

Integration Results

No.	Peak Name	Retention Time min	Area mAU*min	Height mAU	Relative Area %	Relative Height %	Amount n.a.
1		16.758	1184.231	1515.044	50.47	72.01	n.a.
2		42.047	1162.196	588.865	49.53	27.99	n.a.
**Total:**			**2346.427**	**2103.909**	**100.00**	**100.00**	

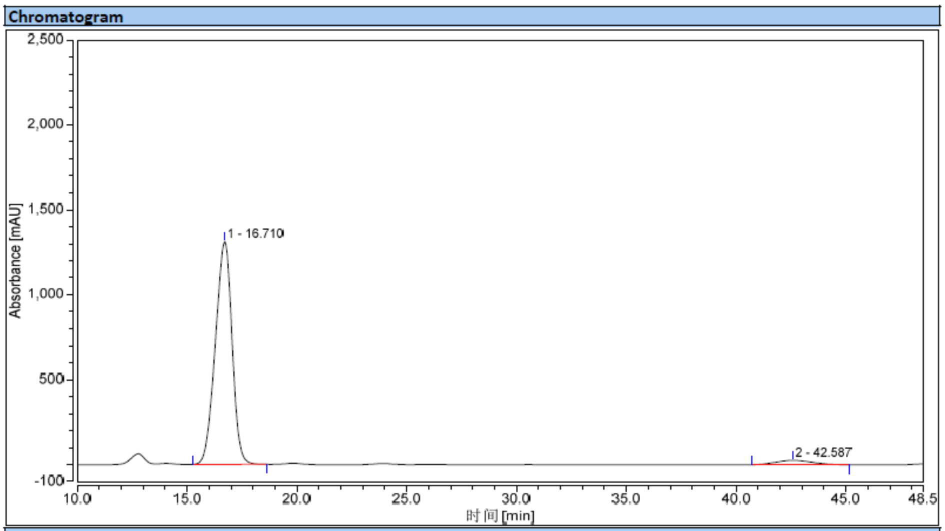

Integration Results

No.	Peak Name	Retention Time min	Area mAU*min	Height mAU	Relative Area %	Relative Height %	Amount n.a.
1		16.710	1157.324	1312.222	95.85	98.06	n.a.
2		42.587	50.082	25.966	4.15	1.94	n.a.
**Total:**			**1207.406**	**1338.188**	**100.00**	**100.00**	

Supplementary Figure 117. HPLC spectrum for compound **5a**

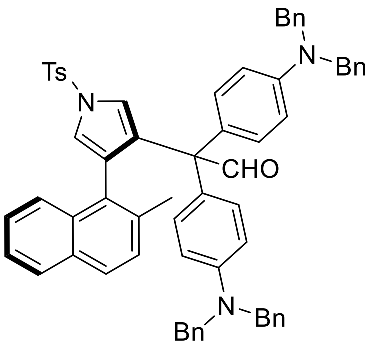

Chromatogram

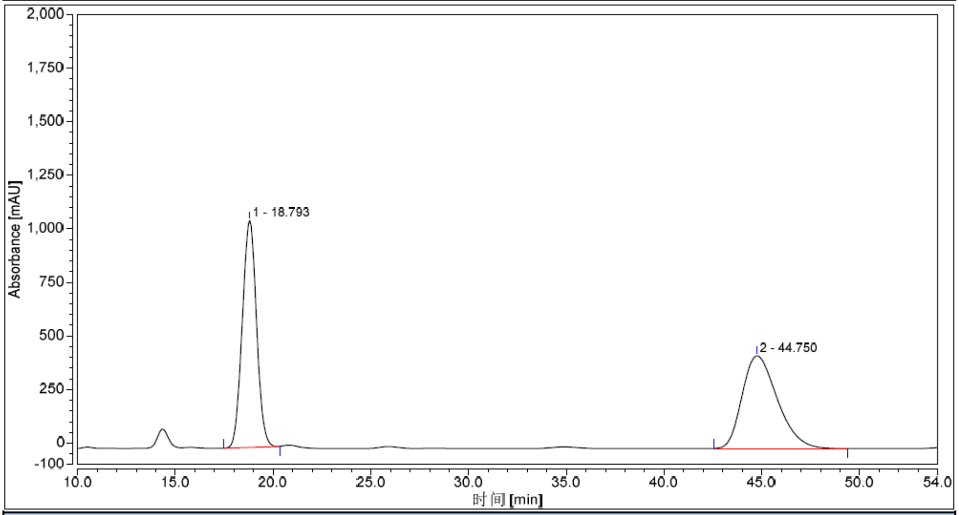

Integration Results

No.	Peak Name	Retention Time min	Area mAU*min	Height mAU	Relative Area %	Relative Height %	Amount n.a.
1		18.793	892.911	1058.595	49.78	71.00	n.a.
2		44.750	900.703	432.319	50.22	29.00	n.a.
**Total:**			**1793.614**	**1490.914**	**100.00**	**100.00**	

Chromatogram

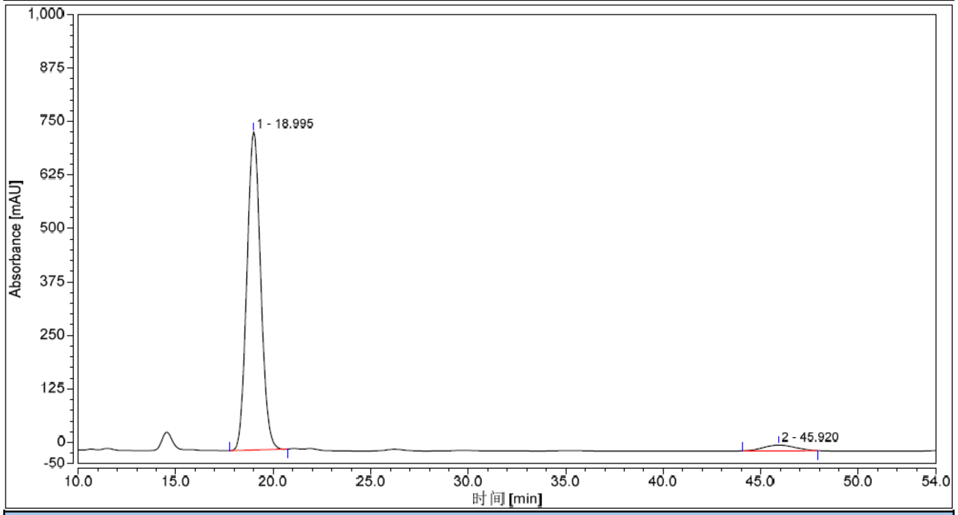

Integration Results

No.	Peak Name	Retention Time min	Area mAU*min	Height mAU	Relative Area %	Relative Height %	Amount n.a.
1		18.995	609.705	744.014	96.11	98.25	n.a.
2		45.920	24.679	13.262	3.89	1.75	n.a.
**Total:**			**634.384**	**757.276**	**100.00**	**100.00**	

Supplementary Figure 118. HPLC spectrum for compound **5b**

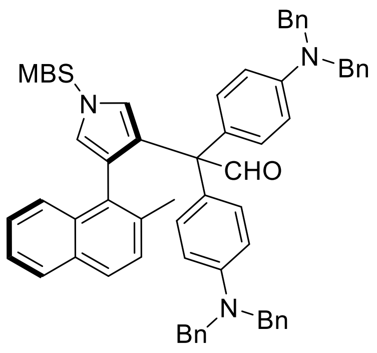

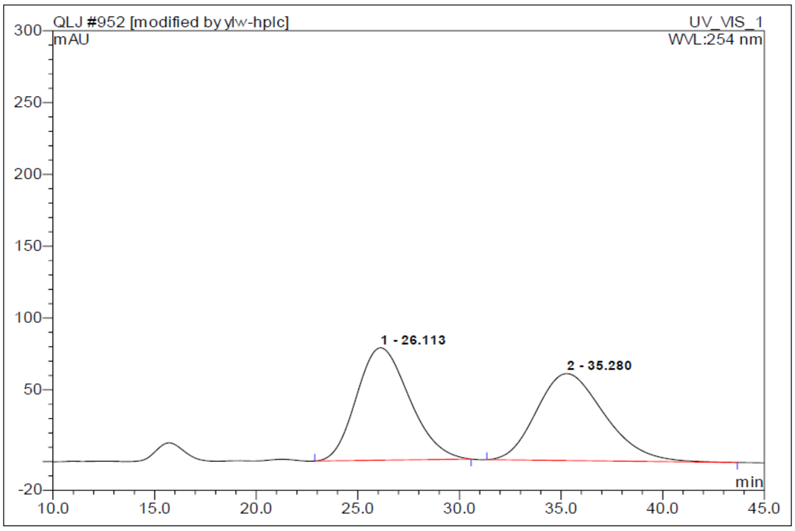

No.	Ret.Time min	Peak Name	Height mAU	Area mAU*min	Rel.Area %	Amount n.a.	Type
1	26.11	n.a.	78.274	236.431	50.21	n.a.	BMB*
2	35.28	n.a.	60.518	234.465	49.79	n.a.	BMB*
Total:			138.793	470.896	100.00	0.000	

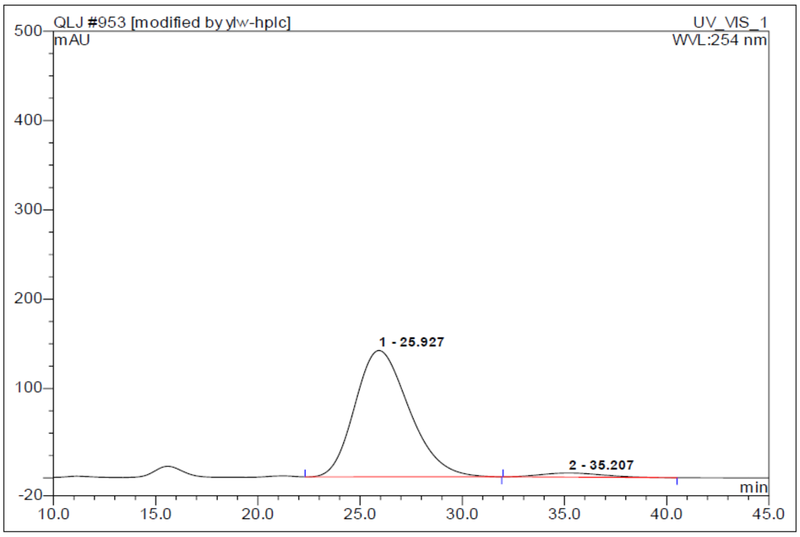

No.	Ret.Time min	Peak Name	Height mAU	Area mAU*min	Rel.Area %	Amount n.a.	Type
1	25.93	n.a.	141.458	435.829	96.42	n.a.	BMB*
2	35.21	n.a.	4.649	16.185	3.58	n.a.	BMB*
Total:			146.107	452.013	100.00	0.000	

Supplementary Figure 119. HPLC spectrum for compound **5c**

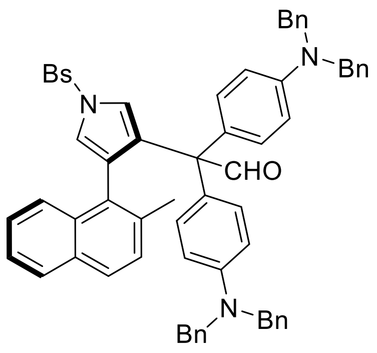

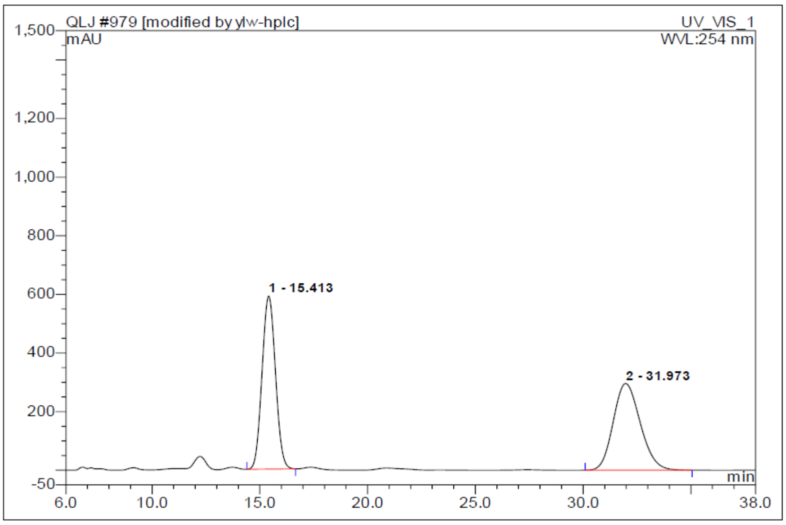

No.	Ret.Time min		Peak Name	Height mAU	Area mAU*min	Rel.Area %	Amount n.a.	Type
1	15.41	n.a.		589.987	425.357	49.77	n.a.	BMB*
2	31.97	n.a.		295.611	429.282	50.23	n.a.	BMB*
Total:				885.598	854.639	100.00	0.000	

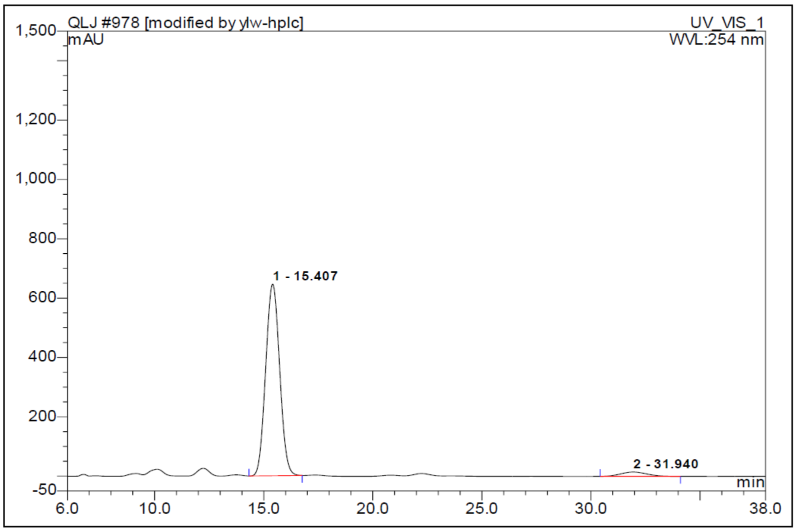

No.	Ret.Time min		Peak Name	Height mAU	Area mAU*min	Rel.Area %	Amount n.a.	Type
1	15.41	n.a.		645.816	478.826	95.90	n.a.	BMB*
2	31.94	n.a.		14.618	20.457	4.10	n.a.	BMB*
Total:				660.433	499.283	100.00	0.000	

Supplementary Figure 120. HPLC spectrum for compound **5d**

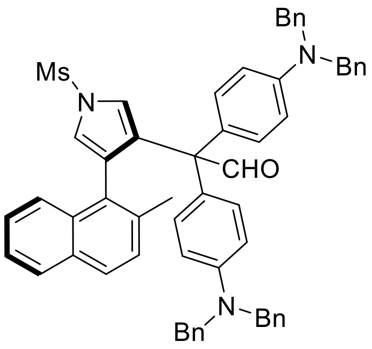

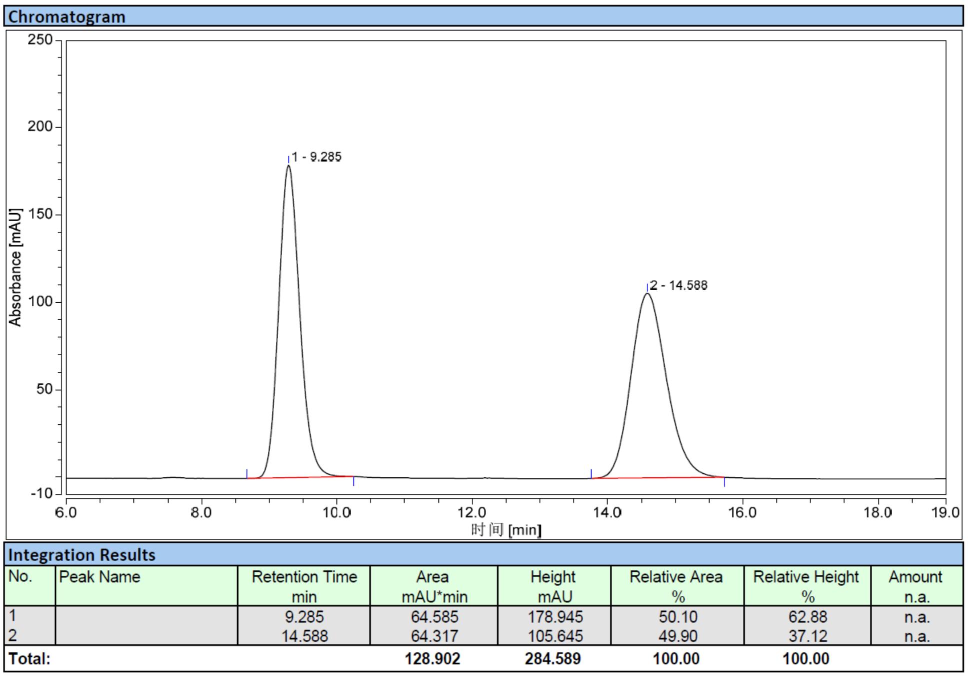

Integration Results

No.	Peak Name	Retention Time<br>min	Area<br>mAU*min	Height<br>mAU	Relative Area<br>%	Relative Height<br>%	Amount<br>n.a.
1		9.285	64.585	178.945	50.10	62.88	n.a.
2		14.588	64.317	105.645	49.90	37.12	n.a.
**Total:**			**128.902**	**284.589**	**100.00**	**100.00**	

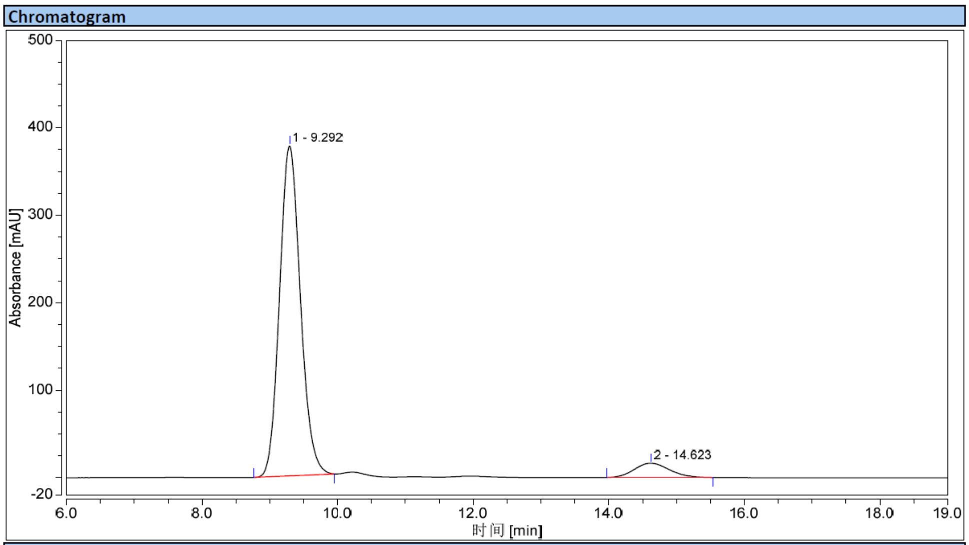

Integration Results

No.	Peak Name	Retention Time<br>min	Area<br>mAU*min	Height<br>mAU	Relative Area<br>%	Relative Height<br>%	Amount<br>n.a.
1		9.292	134.522	377.598	93.19	95.84	n.a.
2		14.623	9.829	16.397	6.81	4.16	n.a.
**Total:**			**144.351**	**393.995**	**100.00**	**100.00**	

Supplementary Figure 121. HPLC spectrum for compound **5e**

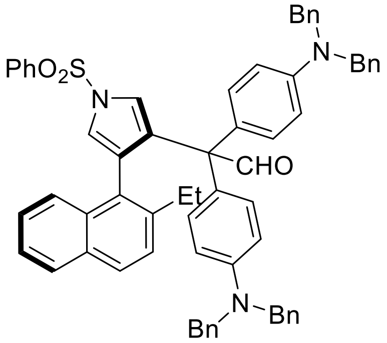

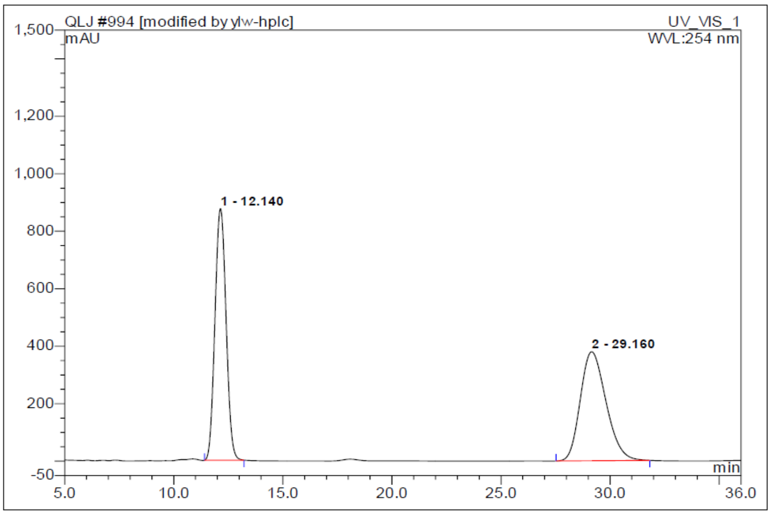

No.	Ret.Time min	Peak Name	Height mAU	Area mAU*min	Rel.Area %	Amount n.a.	Type
1	12.14	n.a.	875.450	516.359	49.95	n.a.	BMB*
2	29.16	n.a.	379.131	517.312	50.05	n.a.	BMB*
Total:			1254.581	1033.671	100.00	0.000	

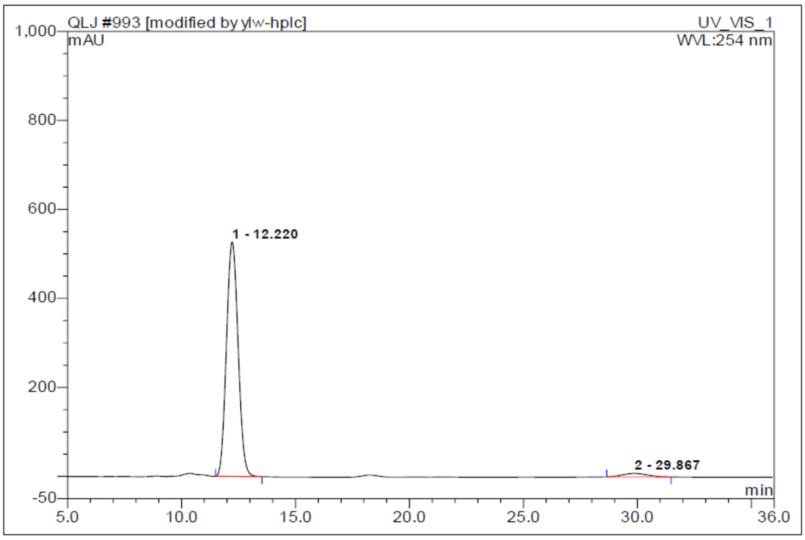

No.	Ret.Time min	Peak Name	Height mAU	Area mAU*min	Rel.Area %	Amount n.a.	Type
1	12.22	n.a.	526.898	311.970	96.88	n.a.	BMB*
2	29.87	n.a.	7.929	10.060	3.12	n.a.	BMB*
Total:			534.828	322.031	100.00	0.000	

Supplementary Figure 122. HPLC spectrum for compound **5f**

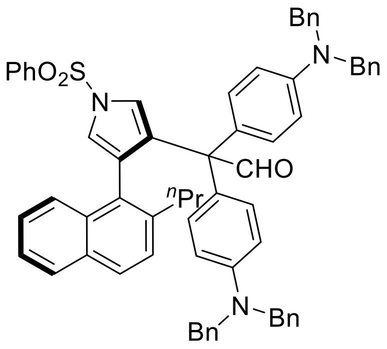

Chromatogram

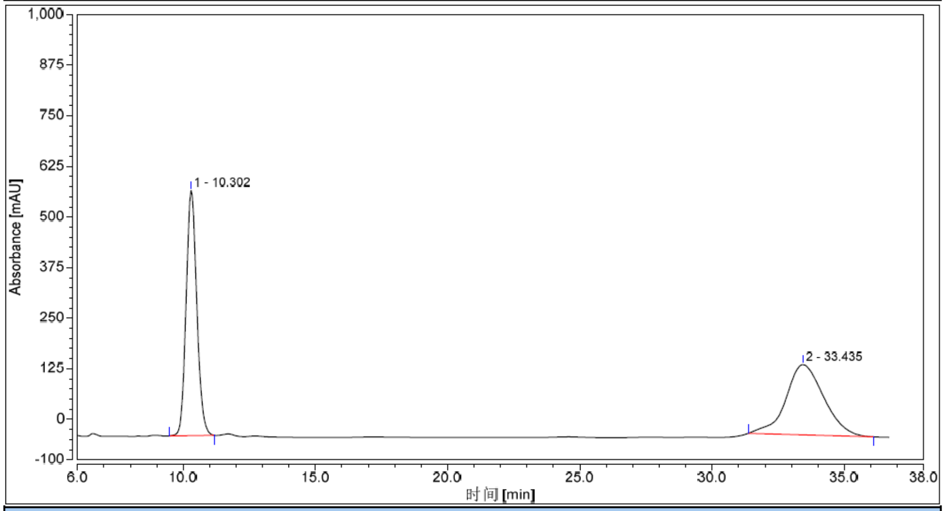

Integration Results

No.	Peak Name	Retention Time min	Area mAU*min	Height mAU	Relative Area %	Relative Height %	Amount n.a.
1		10.302	282.740	605.703	49.48	77.72	n.a.
2		33.435	288.648	173.629	50.52	22.28	n.a.
**Total:**			**571.388**	**779.332**	**100.00**	**100.00**	

Chromatogram

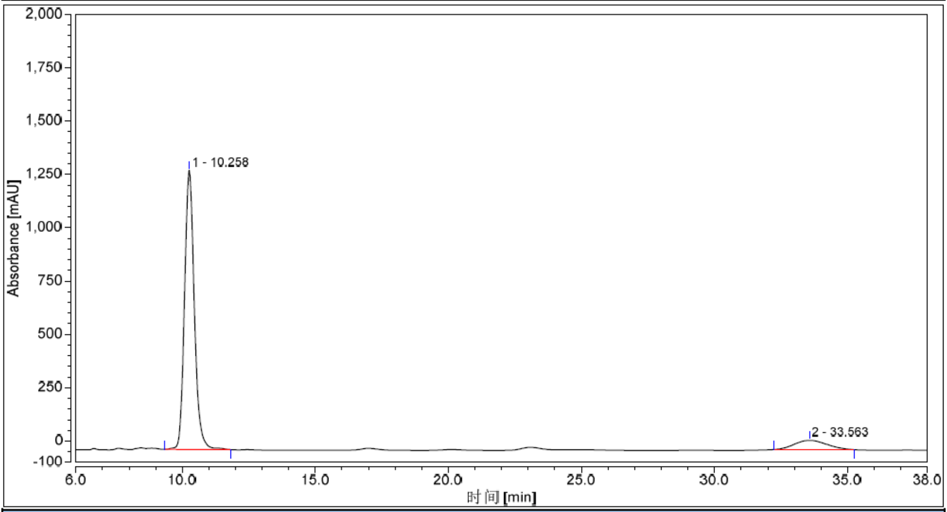

Integration Results

No.	Peak Name	Retention Time min	Area mAU*min	Height mAU	Relative Area %	Relative Height %	Amount n.a.
1		10.258	558.525	1310.020	90.08	96.78	n.a.
2		33.563	61.480	43.522	9.92	3.22	n.a.
**Total:**			**620.004**	**1353.542**	**100.00**	**100.00**	

Supplementary Figure 123. HPLC spectrum for compound **5g**

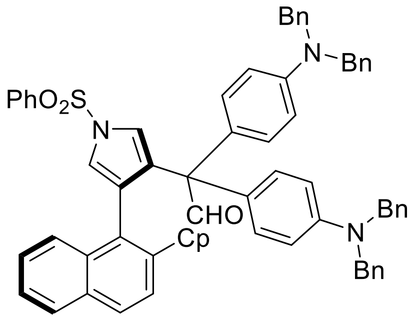

Chromatogram

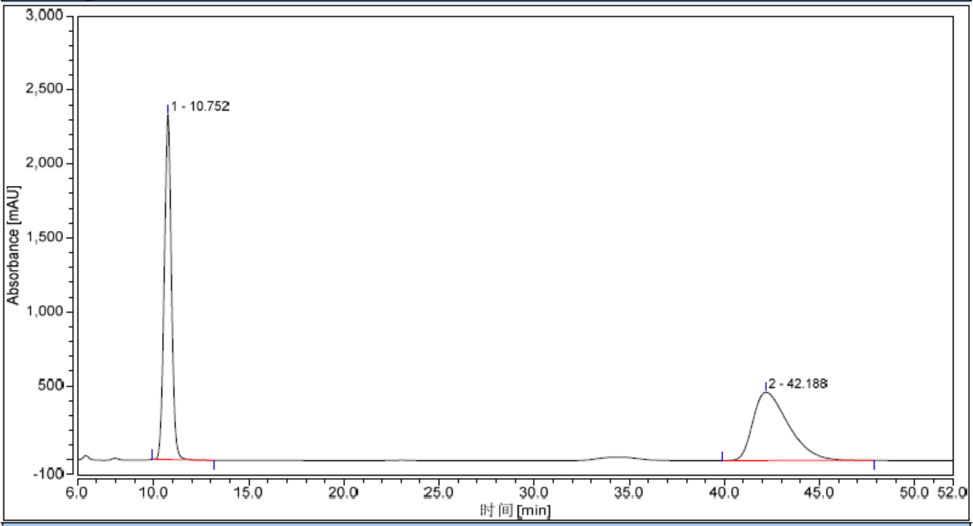

Integration Results

No.	Peak Name	Retention Time<br>min	Area<br>mAU*min	Height<br>mAU	Relative Area<br>%	Relative Height<br>%	Amount<br>n.a.
1		10.752	1016.645	2331.795	50.53	83.49	n.a.
2		42.188	995.459	461.176	49.47	16.51	n.a.
**Total:**			**2012.104**	**2792.971**	**100.00**	**100.00**	

Chromatogram

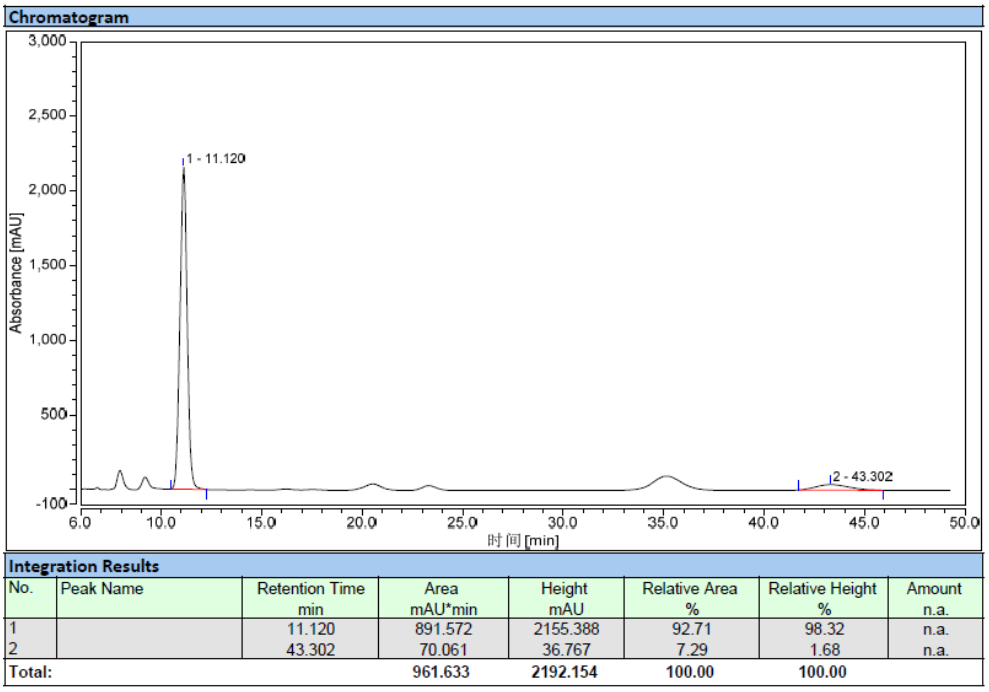

Integration Results

No.	Peak Name	Retention Time<br>min	Area<br>mAU*min	Height<br>mAU	Relative Area<br>%	Relative Height<br>%	Amount<br>n.a.
1		11.120	891.572	2155.388	92.71	98.32	n.a.
2		43.302	70.061	36.767	7.29	1.68	n.a.
**Total:**			**961.633**	**2192.154**	**100.00**	**100.00**	

Supplementary Figure 124. HPLC spectrum for compound **5h**

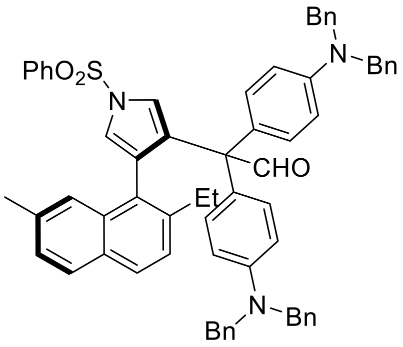

Chromatogram

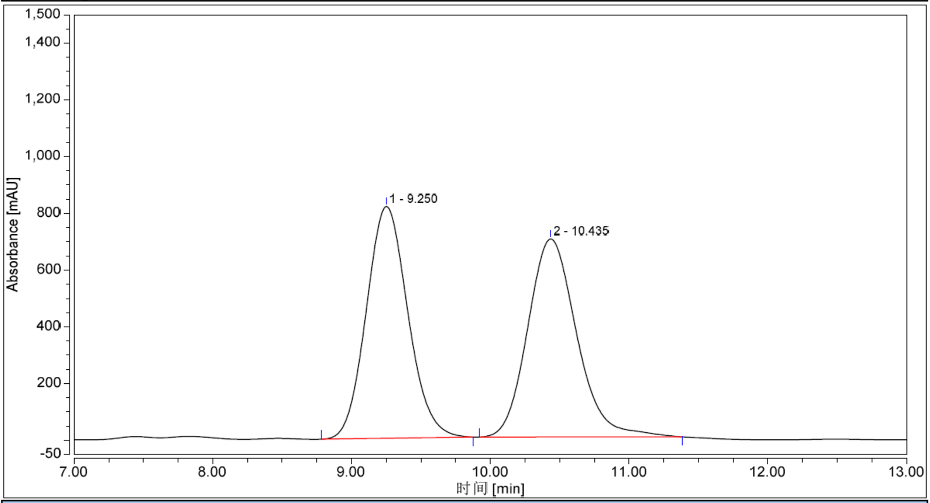

Integration Results

No.	Peak Name	Retention Time min	Area mAU*min	Height mAU	Relative Area %	Relative Height %	Amount n.a.
1		9.250	283.670	817.387	49.89	53.93	n.a.
2		10.435	284.973	698.181	50.11	46.07	n.a.
**Total:**			**568.643**	**1515.568**	**100.00**	**100.00**	

Chromatogram

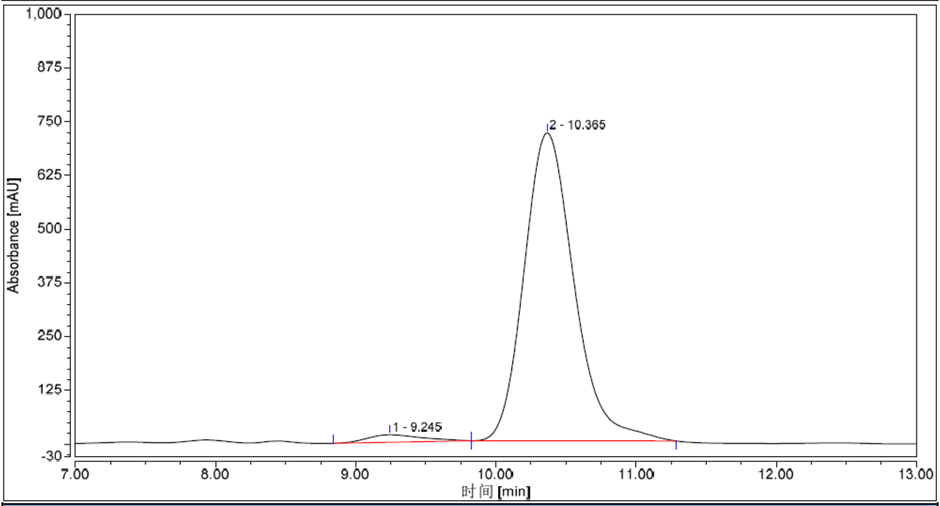

Integration Results

No.	Peak Name	Retention Time min	Area mAU*min	Height mAU	Relative Area %	Relative Height %	Amount n.a.
1		9.245	7.776	17.276	2.56	2.35	n.a.
2		10.365	295.618	716.815	97.44	97.65	n.a.
**Total:**			**303.394**	**734.091**	**100.00**	**100.00**	

Supplementary Figure 125. HPLC spectrum for compound **5i**

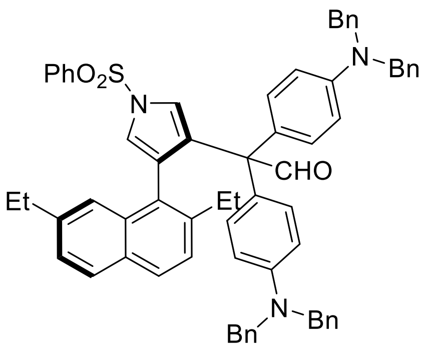

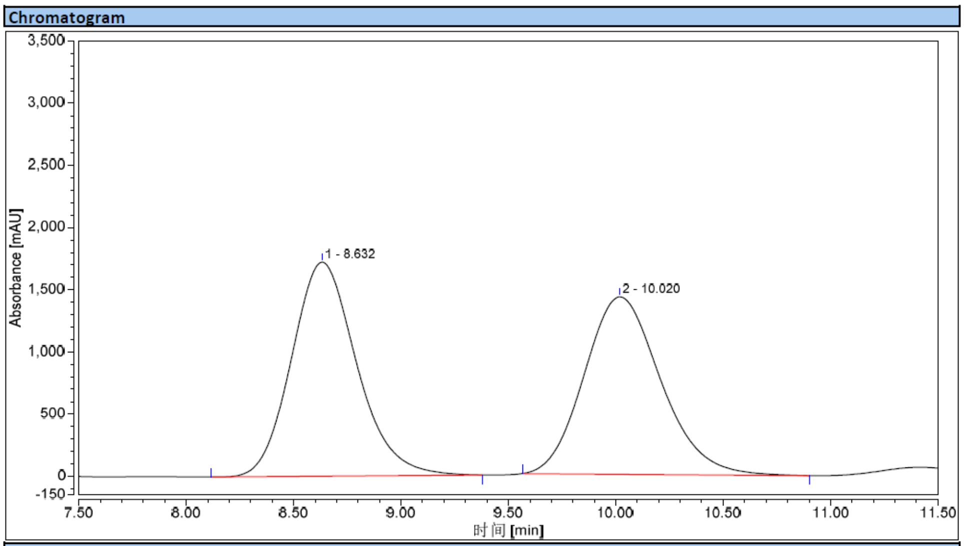

Integration Results

No.	Peak Name	Retention Time min	Area mAU*min	Height mAU	Relative Area %	Relative Height %	Amount n.a.
1		8.632	605.224	1721.274	50.68	54.69	n.a.
2		10.020	589.052	1426.338	49.32	45.31	n.a.
**Total:**			**1194.276**	**3147.612**	**100.00**	**100.00**	

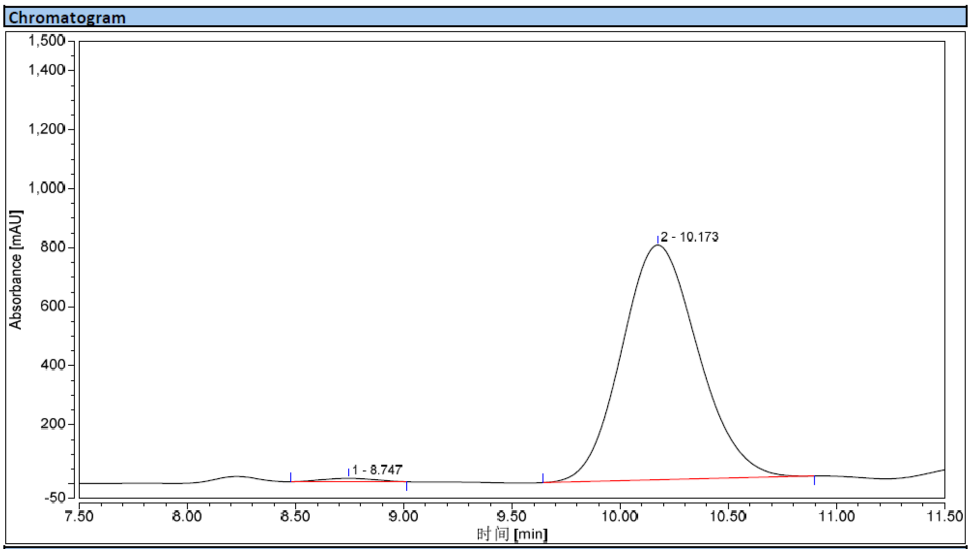

Integration Results

No.	Peak Name	Retention Time min	Area mAU*min	Height mAU	Relative Area %	Relative Height %	Amount n.a.
1		8.747	3.189	11.451	0.97	1.42	n.a.
2		10.173	326.126	795.982	99.03	98.58	n.a.
**Total:**			**329.314**	**807.432**	**100.00**	**100.00**	

Supplementary Figure 126. HPLC spectrum for compound **5j**

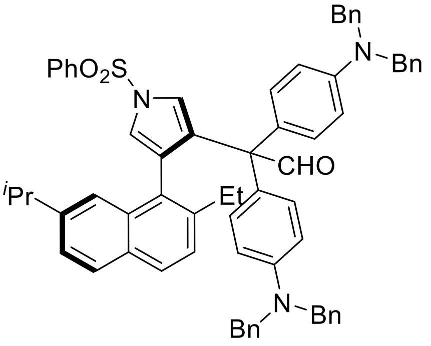

Chromatogram

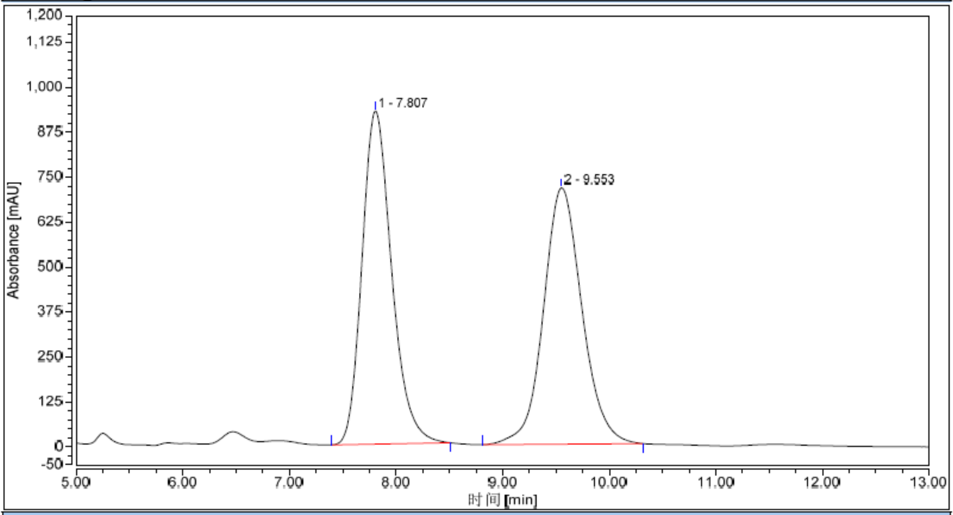

Integration Results

No.	Peak Name	Retention Time<br>min	Area<br>mAU*min	Height<br>mAU	Relative Area<br>%	Relative Height<br>%	Amount<br>n.a.
1		7.807	298.843	927.105	50.06	56.53	n.a.
2		9.553	298.142	713.017	49.94	43.47	n.a.
Total:			596.986	1640.123	100.00	100.00	

Chromatogram

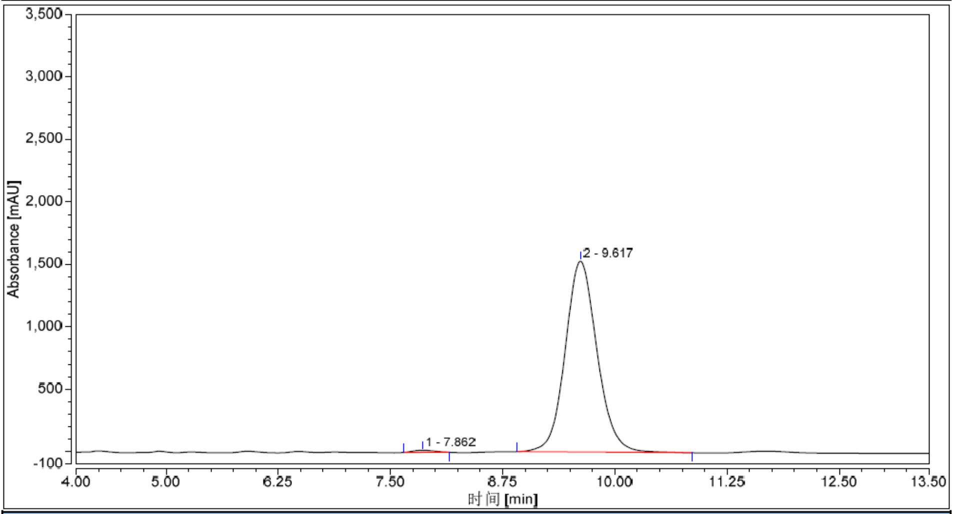

Integration Results

No.	Peak Name	Retention Time<br>min	Area<br>mAU*min	Height<br>mAU	Relative Area<br>%	Relative Height<br>%	Amount<br>n.a.
1		7.862	4.752	17.490	0.75	1.13	n.a.
2		9.617	629.185	1528.724	99.25	98.87	n.a.
Total:			633.936	1546.215	100.00	100.00	

Supplementary Figure 127. HPLC spectrum for compound **5k**

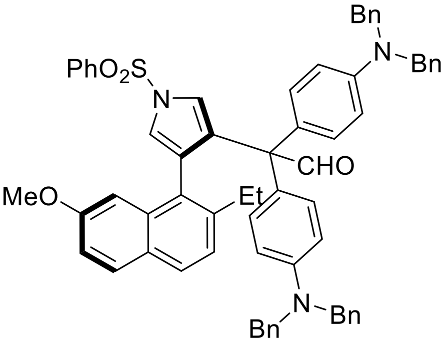

Chromatogram

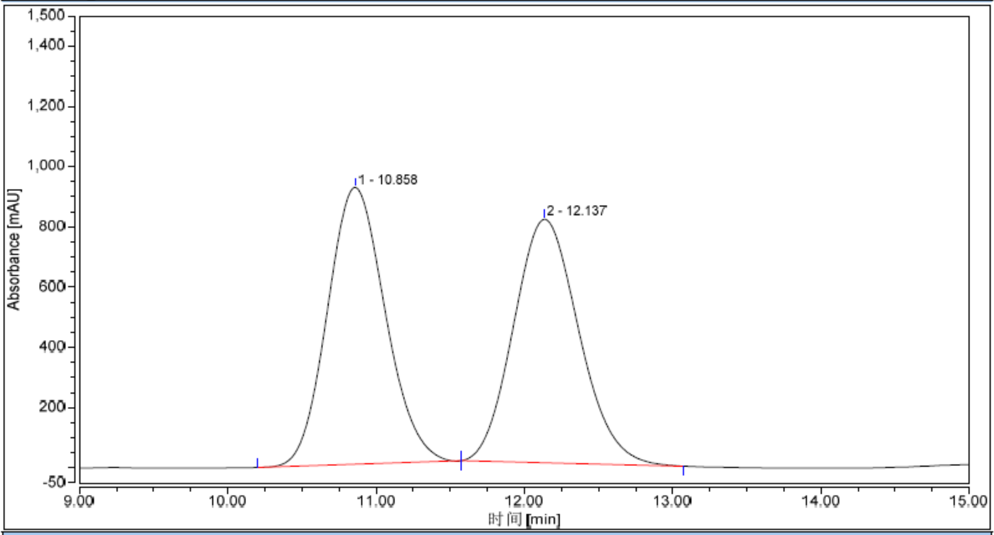

Integration Results

No.	Peak Name	Retention Time<br>min	Area<br>mAU*min	Height<br>mAU	Relative Area<br>%	Relative Height<br>%	Amount<br>n.a.
1		10.858	414.525	918.462	50.18	53.20	n.a.
2		12.137	411.510	808.076	49.82	46.80	n.a.
**Total:**			**826.035**	**1726.539**	**100.00**	**100.00**	

Chromatogram

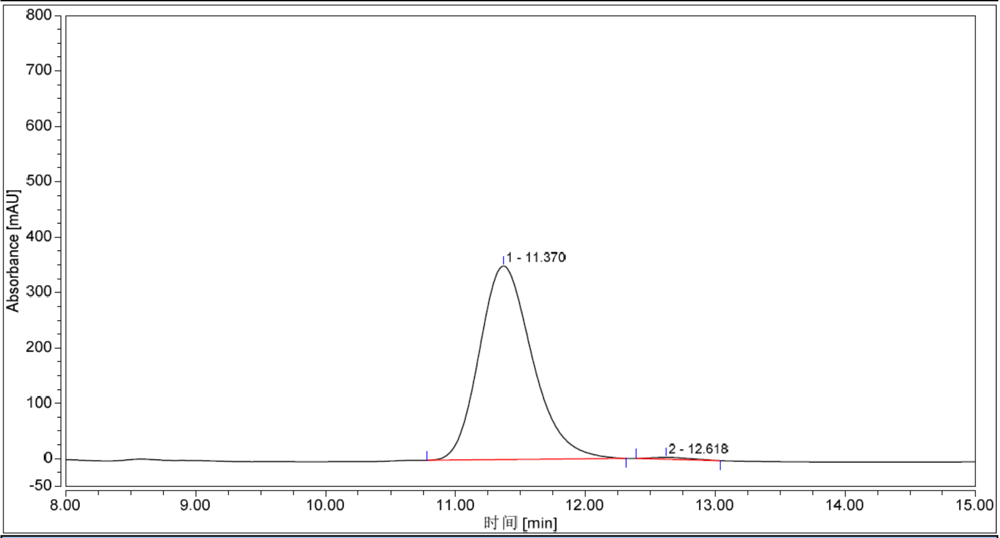

Integration Results

No.	Peak Name	Retention Time<br>min	Area<br>mAU*min	Height<br>mAU	Relative Area<br>%	Relative Height<br>%	Amount<br>n.a.
1		11.370	165.222	349.224	99.30	99.12	n.a.
2		12.618	1.163	3.101	0.70	0.88	n.a.
**Total:**			**166.385**	**352.325**	**100.00**	**100.00**	

Supplementary Figure 128. HPLC spectrum for compound **5l**

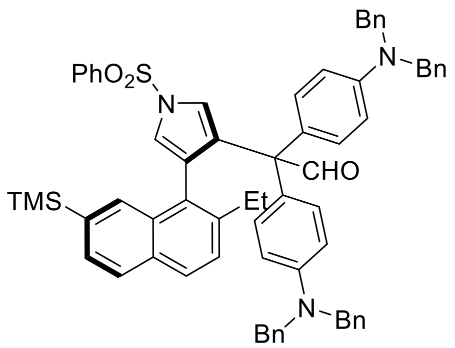

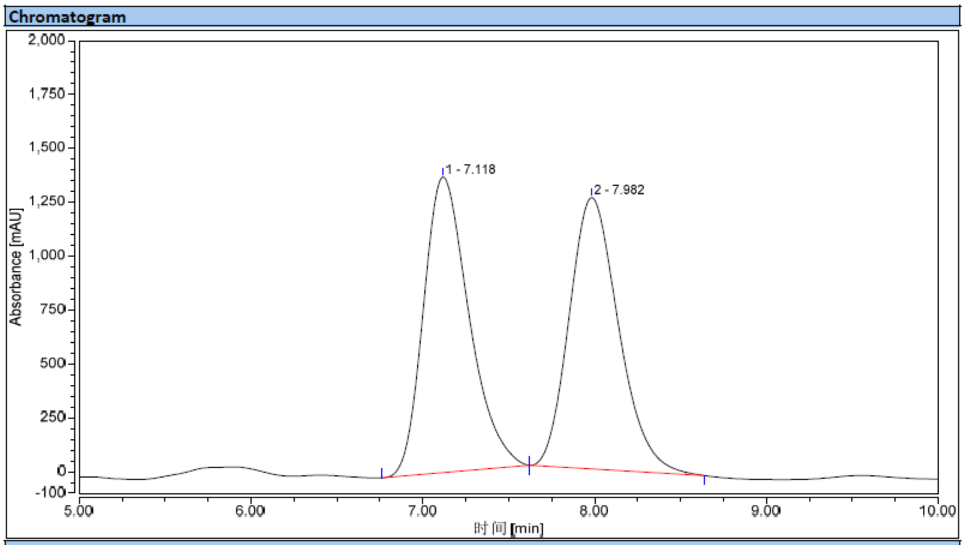

Integration Results

No.	Peak Name	Retention Time<br>min	Area<br>mAU*min	Height<br>mAU	Relative Area<br>%	Relative Height<br>%	Amount<br>n.a.
1		7.118	420.233	1371.536	50.11	52.14	n.a.
2		7.982	418.420	1259.045	49.89	47.86	n.a.
Total:			838.653	2630.581	100.00	100.00	

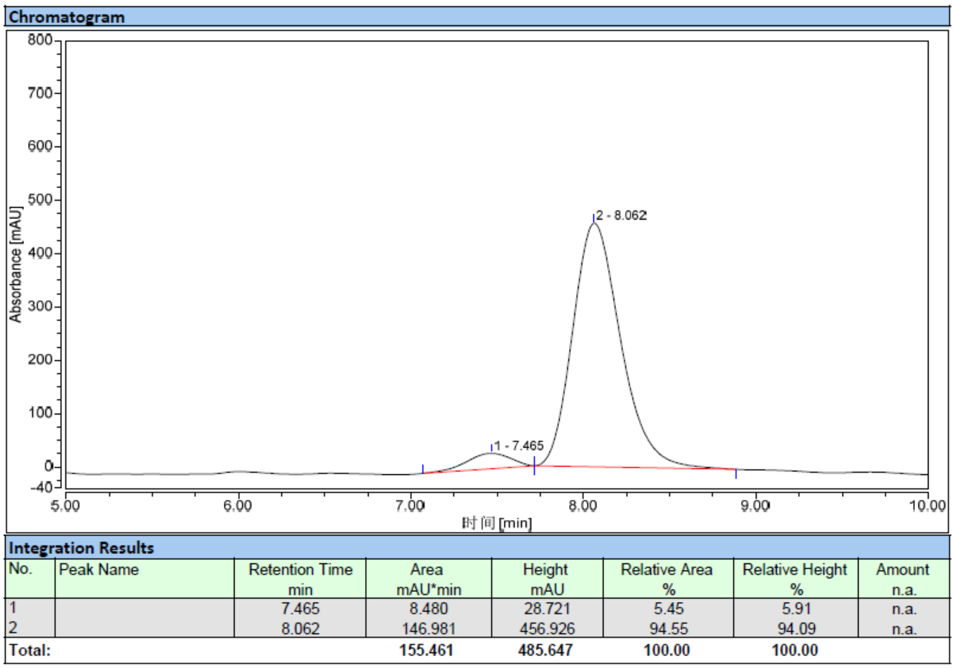

Integration Results

No.	Peak Name	Retention Time<br>min	Area<br>mAU*min	Height<br>mAU	Relative Area<br>%	Relative Height<br>%	Amount<br>n.a.
1		7.465	8.480	28.721	5.45	5.91	n.a.
2		8.062	146.981	456.926	94.55	94.09	n.a.
Total:			155.461	485.647	100.00	100.00	

Supplementary Figure 129. HPLC spectrum for compound **5m**

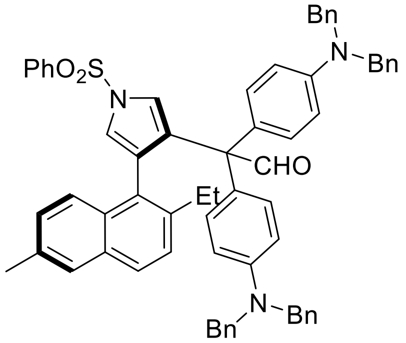

Chromatogram

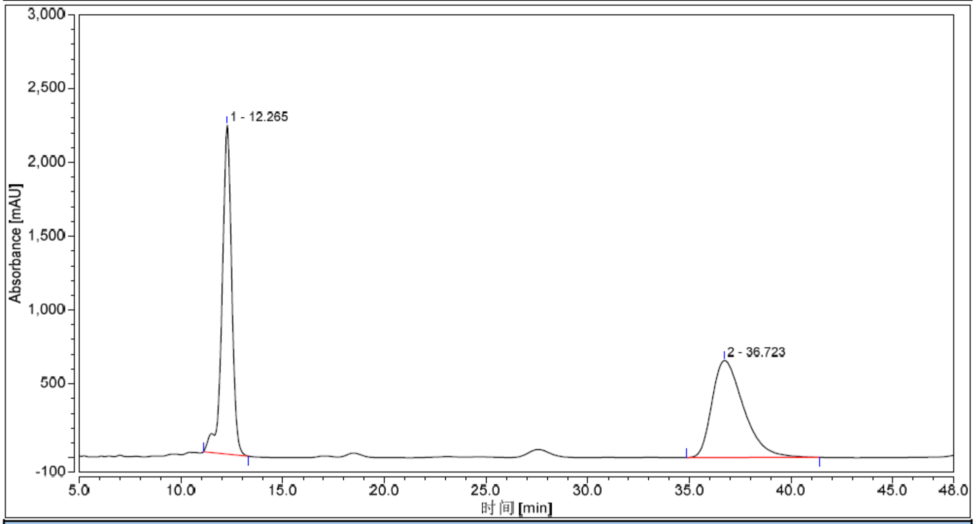

Integration Results

No.	Peak Name	Retention Time<br>min	Area<br>mAU*min	Height<br>mAU	Relative Area<br>%	Relative Height<br>%	Amount<br>n.a.
1		12.265	1217.845	2227.852	50.66	77.22	n.a.
2		36.723	1186.270	657.057	49.34	22.78	n.a.
**Total:**			**2404.115**	**2884.909**	**100.00**	**100.00**	

Chromatogram

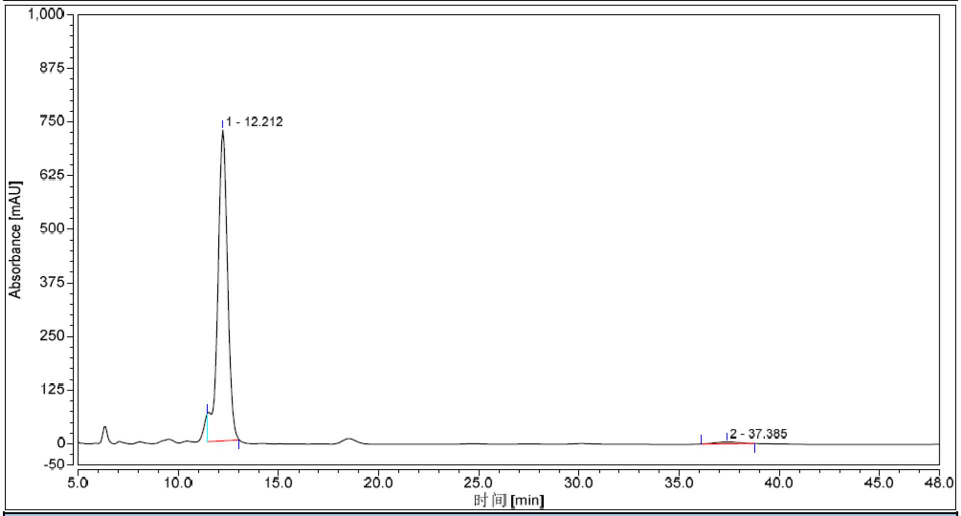

Integration Results

No.	Peak Name	Retention Time<br>min	Area<br>mAU*min	Height<br>mAU	Relative Area<br>%	Relative Height<br>%	Amount<br>n.a.
1		12.212	405.731	724.524	98.57	99.43	n.a.
2		37.385	5.875	4.137	1.43	0.57	n.a.
**Total:**			**411.606**	**728.661**	**100.00**	**100.00**	

Supplementary Figure 130. HPLC spectrum for compound **5n**

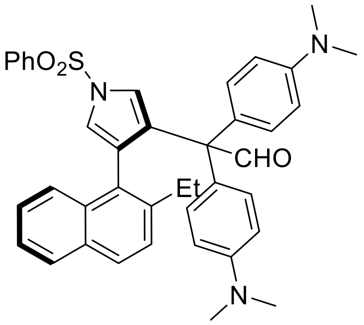

Chromatogram

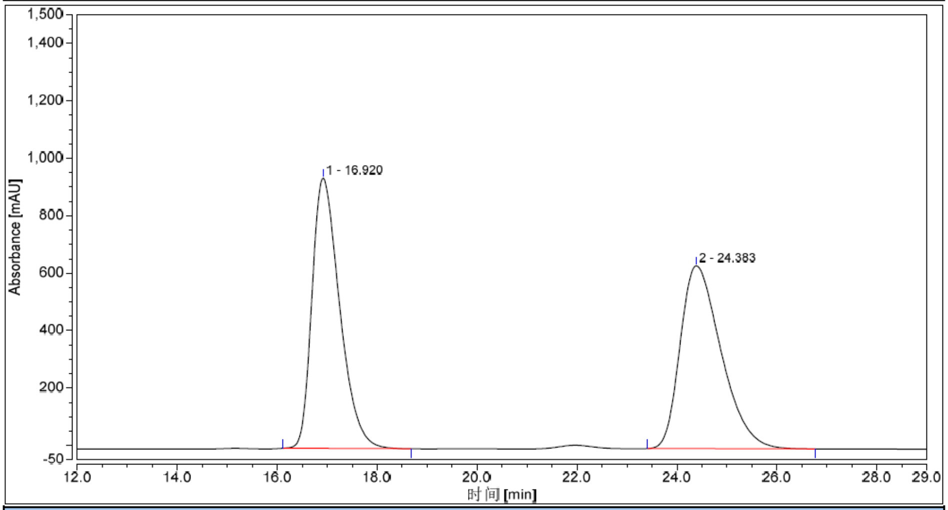

Integration Results

No.	Peak Name	Retention Time<br>min	Area<br>mAU*min	Height<br>mAU	Relative Area<br>%	Relative Height<br>%	Amount<br>n.a.
1		16.920	603.020	940.971	49.97	59.65	n.a.
2		24.383	603.737	636.600	50.03	40.35	n.a.
**Total:**			**1206.756**	**1577.571**	**100.00**	**100.00**	

Chromatogram

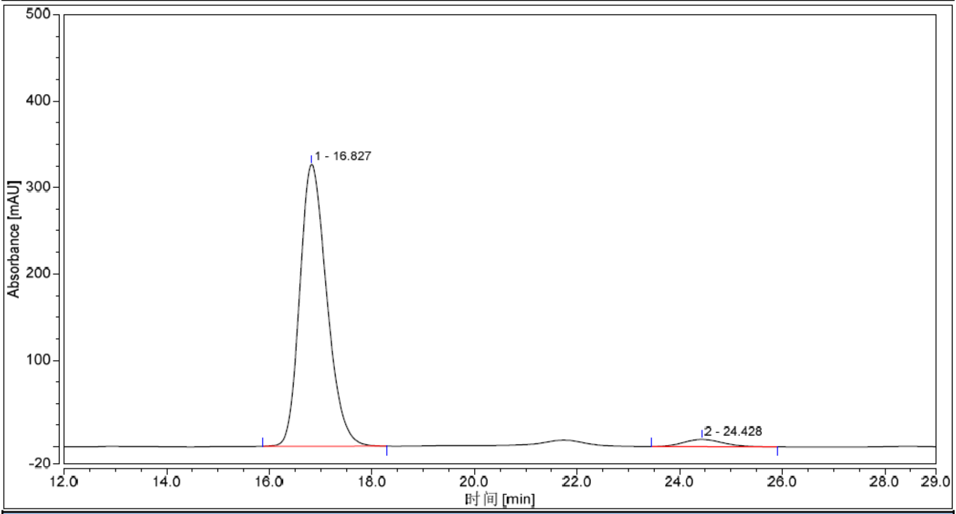

Integration Results

No.	Peak Name	Retention Time<br>min	Area<br>mAU*min	Height<br>mAU	Relative Area<br>%	Relative Height<br>%	Amount<br>n.a.
1		16.827	198.704	326.338	96.38	97.50	n.a.
2		24.428	7.453	8.380	3.62	2.50	n.a.
**Total:**			**206.156**	**334.718**	**100.00**	**100.00**	

Supplementary Figure 131. HPLC spectrum for compound **5o**

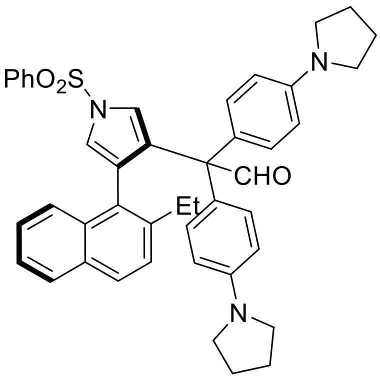

Chromatogram

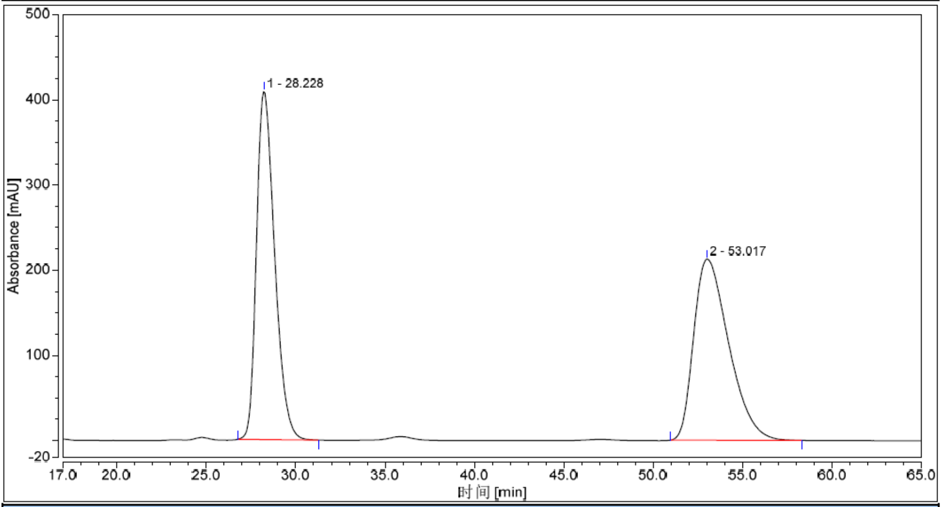

Integration Results

No.	Peak Name	Retention Time min	Area mAU*min	Height mAU	Relative Area %	Relative Height %	Amount n.a.
1		28.228	481.292	408.837	49.93	65.77	n.a.
2		53.017	482.632	212.749	50.07	34.23	n.a.
**Total:**			**963.924**	**621.586**	**100.00**	**100.00**	

Chromatogram

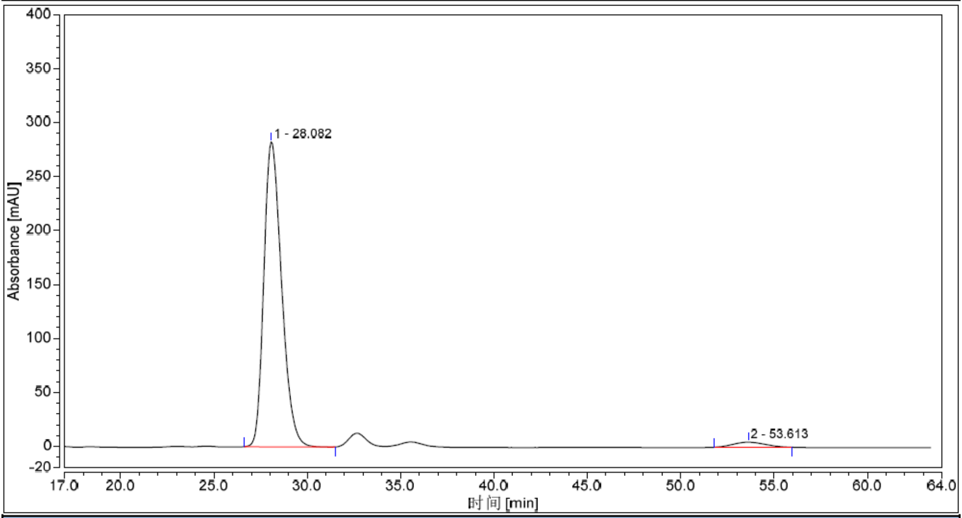

Integration Results

No.	Peak Name	Retention Time min	Area mAU*min	Height mAU	Relative Area %	Relative Height %	Amount n.a.
1		28.082	311.237	282.926	97.09	98.35	n.a.
2		53.613	9.313	4.748	2.91	1.65	n.a.
**Total:**			**320.550**	**287.674**	**100.00**	**100.00**	

Supplementary Figure 132. HPLC spectrum for compound **5p**

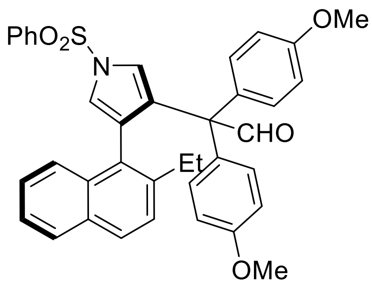

Chromatogram

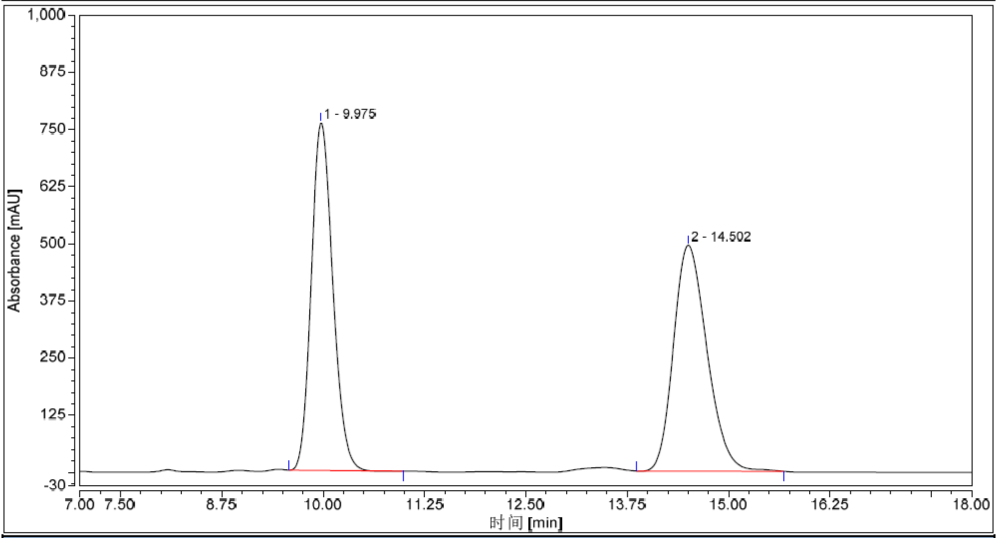

Integration Results

No.	Peak Name	Retention Time<br>min	Area<br>mAU*min	Height<br>mAU	Relative Area<br>%	Relative Height<br>%	Amount<br>n.a.
1		9.975	234.136	761.241	49.75	60.62	n.a.
2		14.502	236.476	494.514	50.25	39.38	n.a.
**Total:**			**470.612**	**1255.755**	**100.00**	**100.00**	

Chromatogram

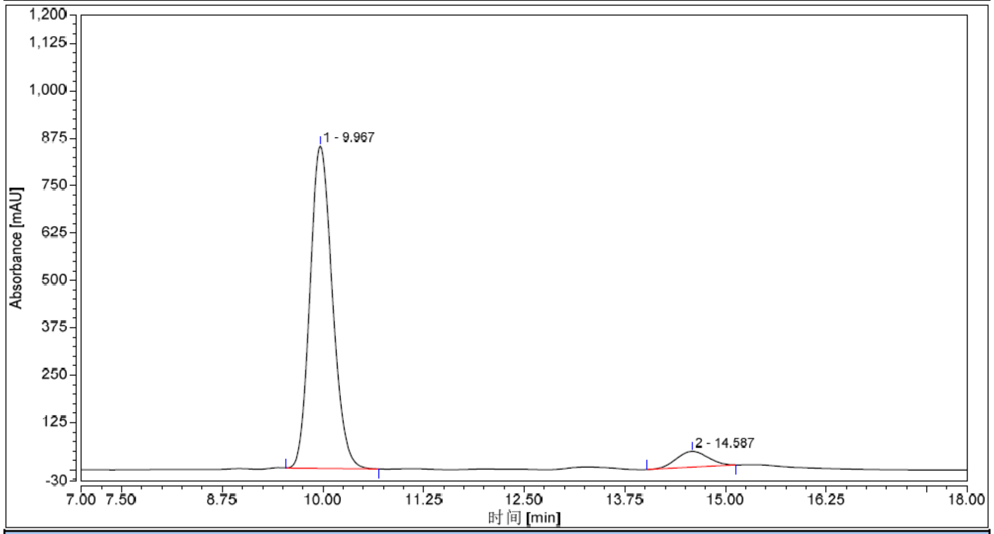

Integration Results

No.	Peak Name	Retention Time<br>min	Area<br>mAU*min	Height<br>mAU	Relative Area<br>%	Relative Height<br>%	Amount<br>n.a.
1		9.967	274.558	850.728	93.48	95.32	n.a.
2		14.587	19.147	41.739	6.52	4.68	n.a.
**Total:**			**293.705**	**892.467**	**100.00**	**100.00**	

Supplementary Figure 133. HPLC spectrum for compound **5q**

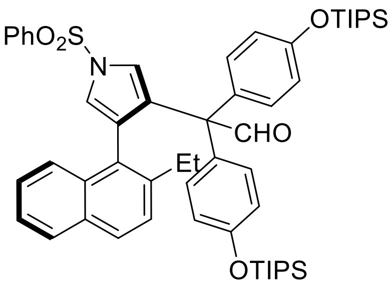

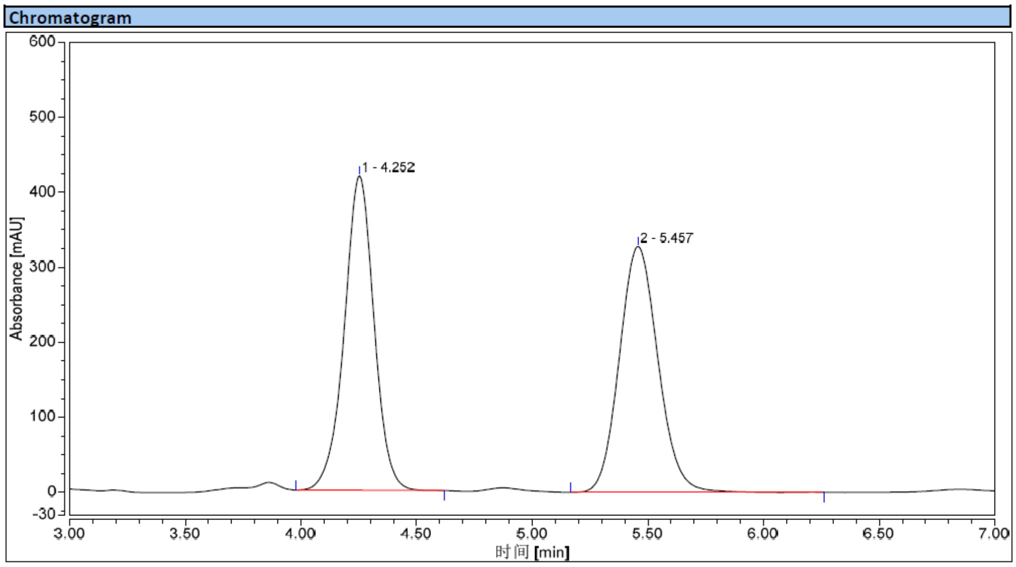

Integration Results

No.	Peak Name	Retention Time min	Area mAU*min	Height mAU	Relative Area %	Relative Height %	Amount n.a.
1		4.252	62.433	419.173	49.60	56.11	n.a.
2		5.457	63.432	327.858	50.40	43.89	n.a.
**Total:**			**125.865**	**747.031**	**100.00**	**100.00**	

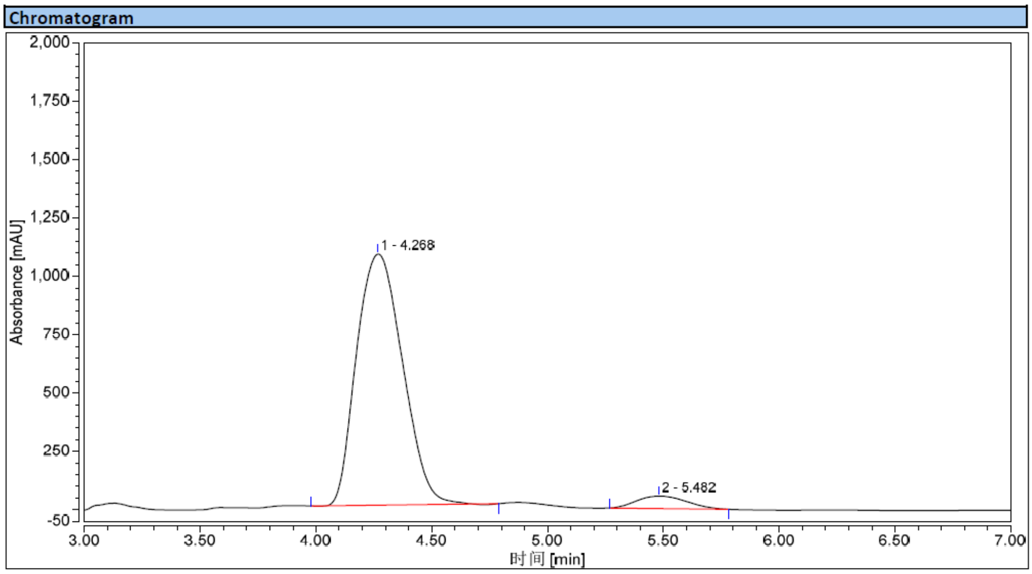

Integration Results

No.	Peak Name	Retention Time min	Area mAU*min	Height mAU	Relative Area %	Relative Height %	Amount n.a.
1		4.268	247.568	1076.676	94.90	95.28	n.a.
2		5.482	13.313	53.388	5.10	4.72	n.a.
**Total:**			**260.880**	**1130.065**	**100.00**	**100.00**	

Supplementary Figure 134. HPLC spectrum for compound **5r**

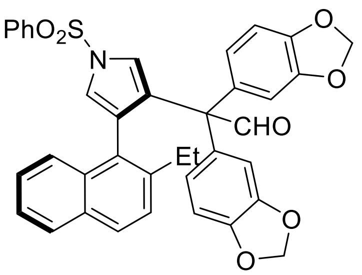

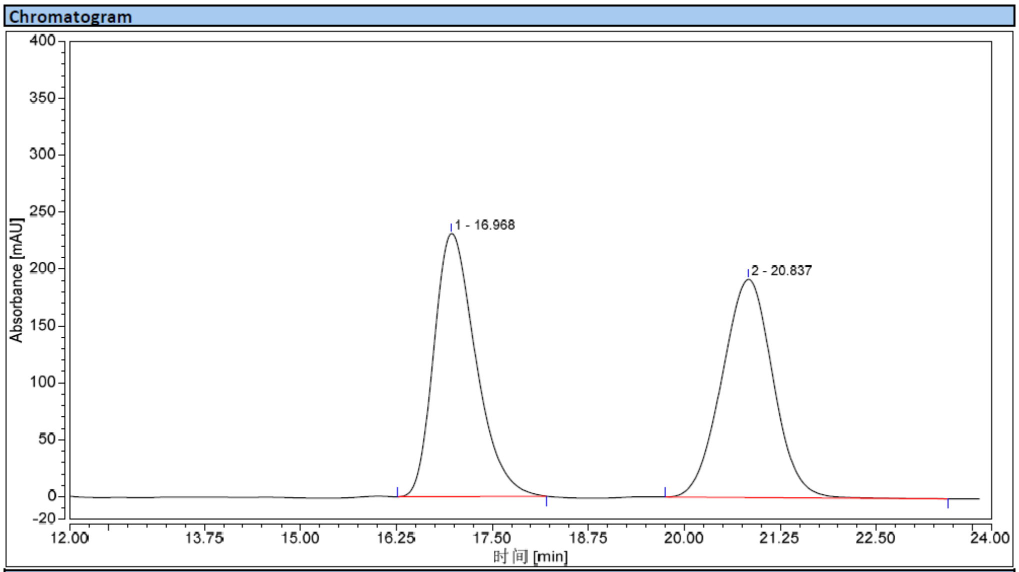

Integration Results

No.	Peak Name	Retention Time<br>min	Area<br>mAU*min	Height<br>mAU	Relative Area<br>%	Relative Height<br>%	Amount<br>n.a.
1		16.968	145.987	231.148	49.73	54.65	n.a.
2		20.837	147.544	191.841	50.27	45.35	n.a.
**Total:**			**293.531**	**422.990**	**100.00**	**100.00**	

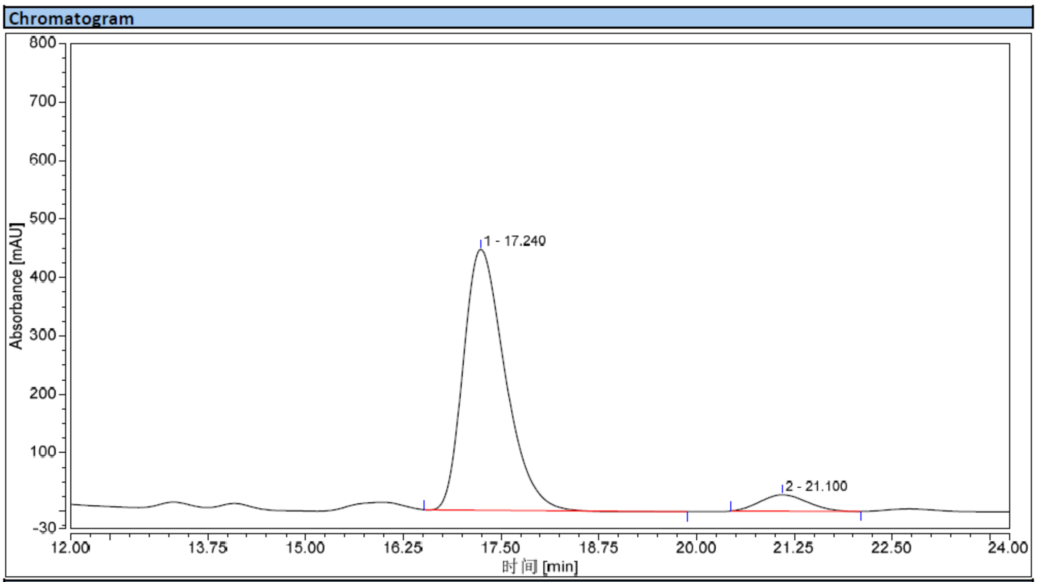

Integration Results

No.	Peak Name	Retention Time<br>min	Area<br>mAU*min	Height<br>mAU	Relative Area<br>%	Relative Height<br>%	Amount<br>n.a.
1		17.240	275.702	446.188	93.44	94.14	n.a.
2		21.100	19.342	27.791	6.56	5.86	n.a.
**Total:**			**295.044**	**473.978**	**100.00**	**100.00**	

Supplementary Figure 135. HPLC spectrum for compound **5s**

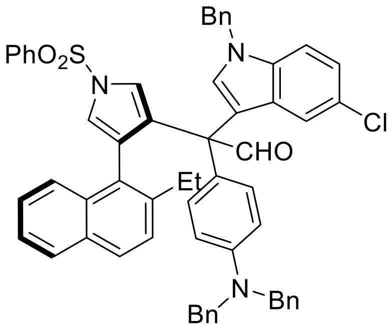

Chromatogram

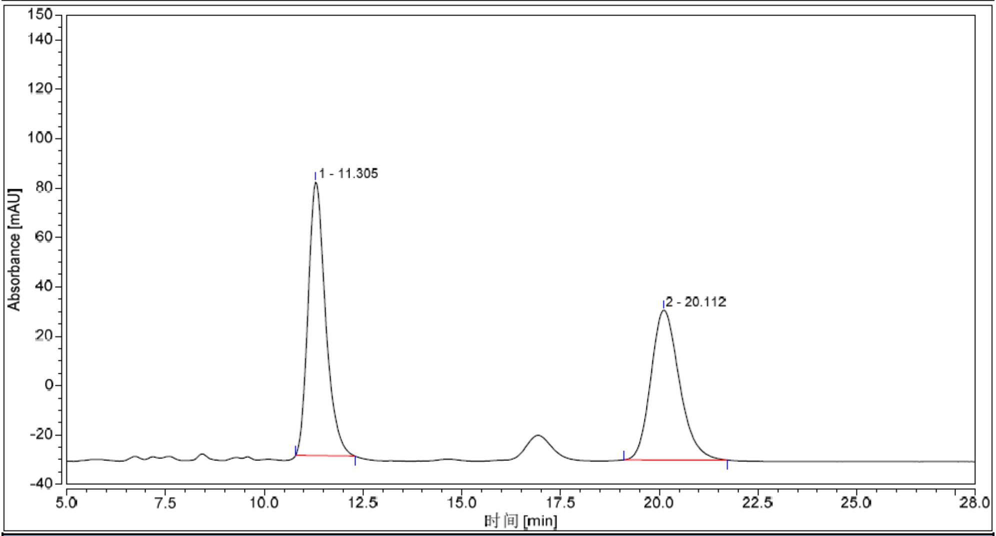

Integration Results

No.	Peak Name	Retention Time<br>min	Area<br>mAU*min	Height<br>mAU	Relative Area<br>%	Relative Height<br>%	Amount<br>n.a.
1		11.305	56.702	110.849	52.80	64.62	n.a.
2		20.112	50.682	60.696	47.20	35.38	n.a.
**Total:**			**107.384**	**171.545**	**100.00**	**100.00**	

Chromatogram

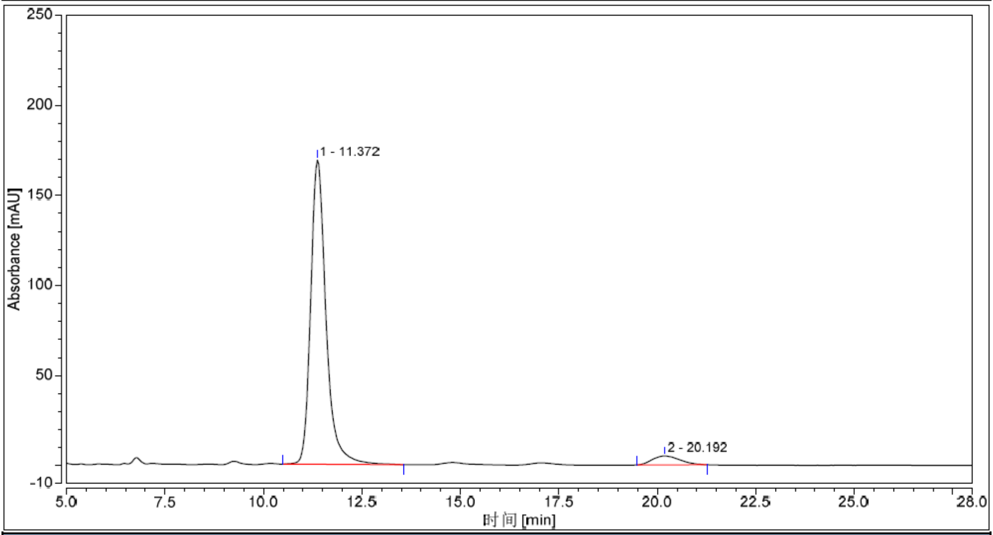

Integration Results

No.	Peak Name	Retention Time<br>min	Area<br>mAU*min	Height<br>mAU	Relative Area<br>%	Relative Height<br>%	Amount<br>n.a.
1		11.372	76.556	168.976	95.03	97.17	n.a.
2		20.192	4.000	4.917	4.97	2.83	n.a.
**Total:**			**80.557**	**173.892**	**100.00**	**100.00**	

Supplementary Figure 136. HPLC spectrum for compound **5t (major)**

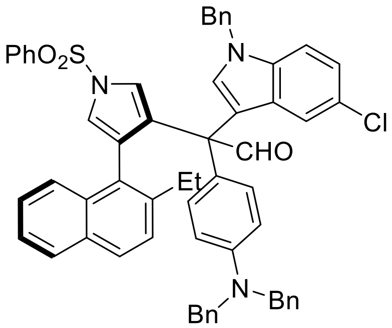

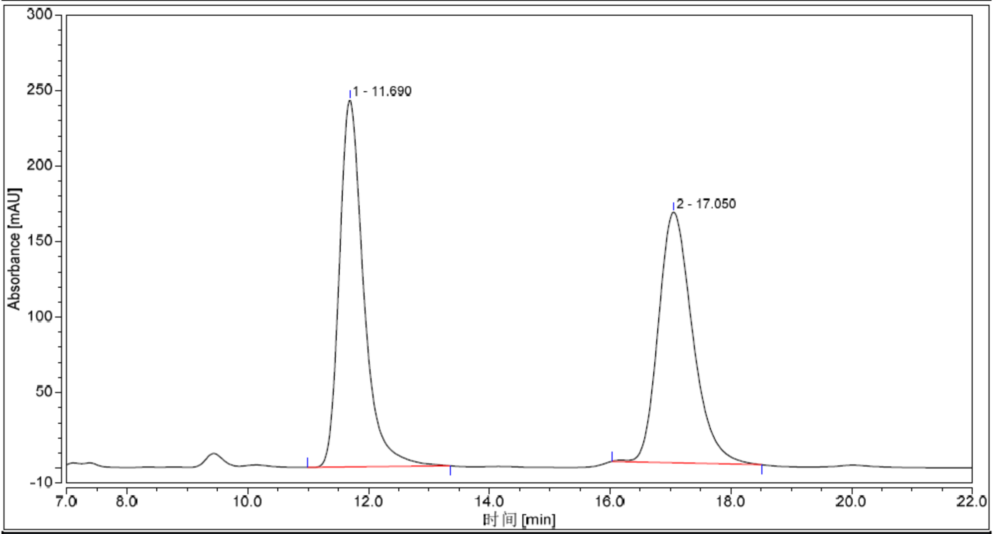

Integration Results

No.	Peak Name	Retention Time min	Area mAU*min	Height mAU	Relative Area %	Relative Height %	Amount n.a.
1		11.690	113.232	243.129	50.94	59.42	n.a.
2		17.050	109.039	166.014	49.06	40.58	n.a.
**Total:**			**222.270**	**409.143**	**100.00**	**100.00**	

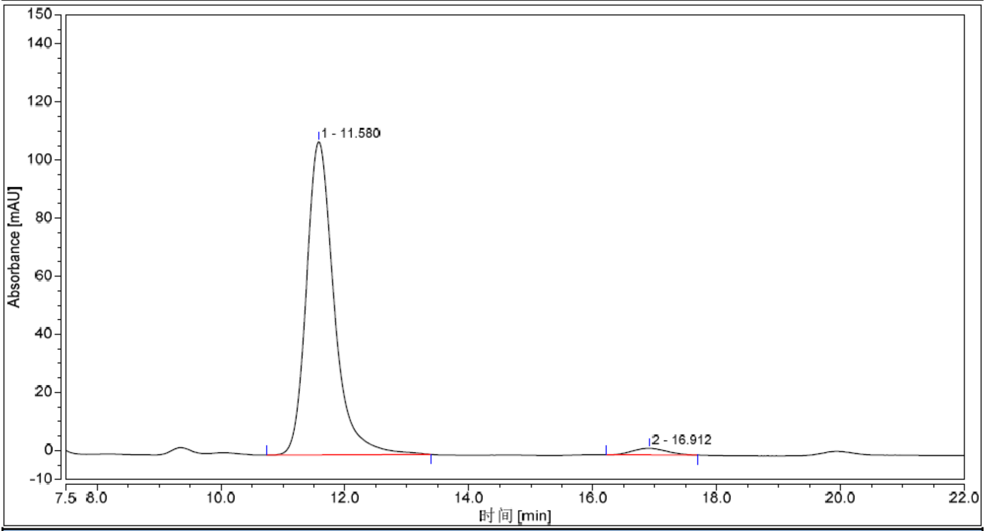

Integration Results

No.	Peak Name	Retention Time min	Area mAU*min	Height mAU	Relative Area %	Relative Height %	Amount n.a.
1		11.580	55.766	107.879	97.46	97.92	n.a.
2		16.912	1.455	2.287	2.54	2.08	n.a.
**Total:**			**57.222**	**110.166**	**100.00**	**100.00**	

Supplementary Figure 137. HPLC spectrum for compound **5t (minor)**

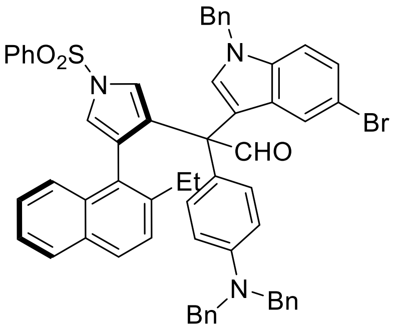

Chromatogram

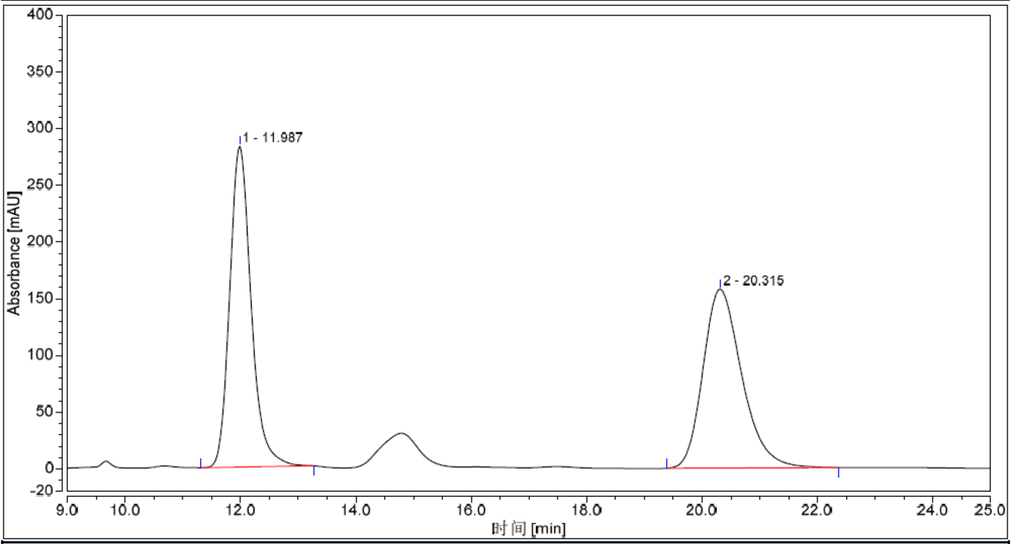

Integration Results

No.	Peak Name	Retention Time min	Area mAU*min	Height mAU	Relative Area %	Relative Height %	Amount n.a.
1		11.987	122.001	282.886	49.85	64.20	n.a.
2		20.315	122.746	157.762	50.15	35.80	n.a.
**Total:**			**244.747**	**440.648**	**100.00**	**100.00**	

Chromatogram

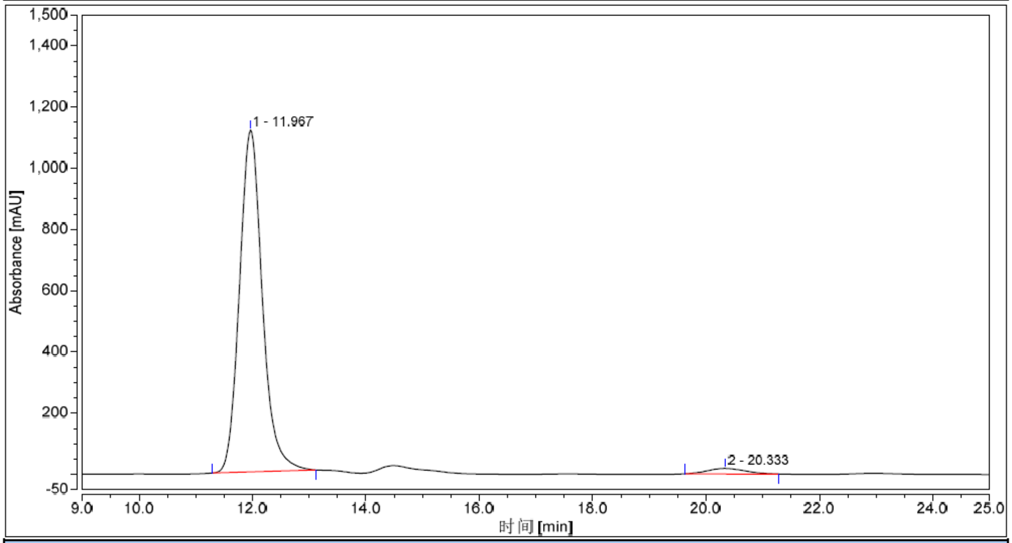

Integration Results

No.	Peak Name	Retention Time min	Area mAU*min	Height mAU	Relative Area %	Relative Height %	Amount n.a.
1		11.967	520.037	1116.887	97.47	98.43	n.a.
2		20.333	13.486	17.773	2.53	1.57	n.a.
**Total:**			**533.523**	**1134.660**	**100.00**	**100.00**	

Supplementary Figure 138. HPLC spectrum for compound **5u (major)**

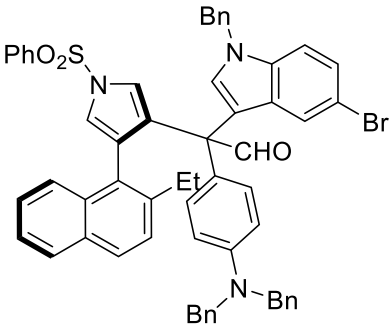

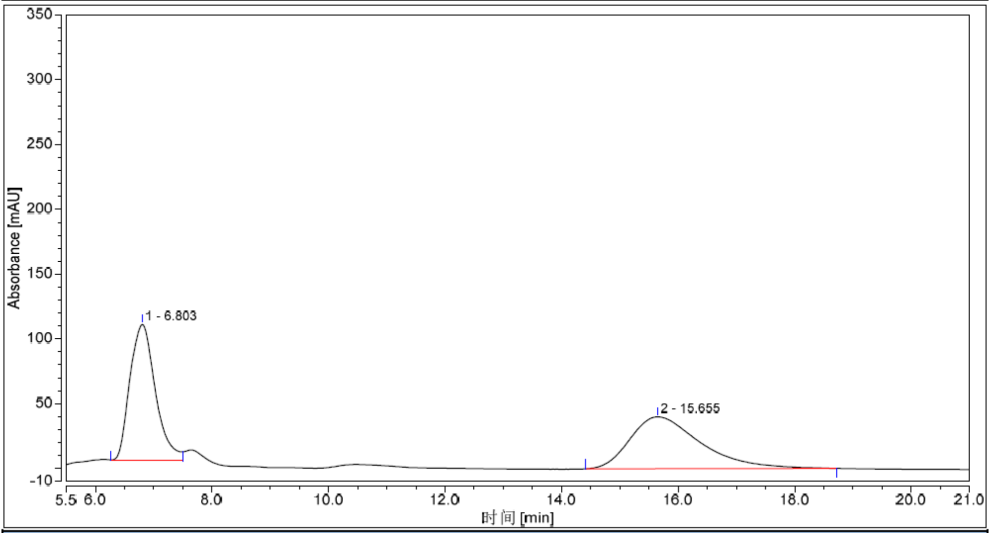

Integration Results

No.	Peak Name	Retention Time<br>min	Area<br>mAU*min	Height<br>mAU	Relative Area<br>%	Relative Height<br>%	Amount<br>n.a.
1		6.803	52.637	104.602	48.33	72.32	n.a.
2		15.655	56.274	40.027	51.67	27.68	n.a.
**Total:**			**108.911**	**144.629**	**100.00**	**100.00**	

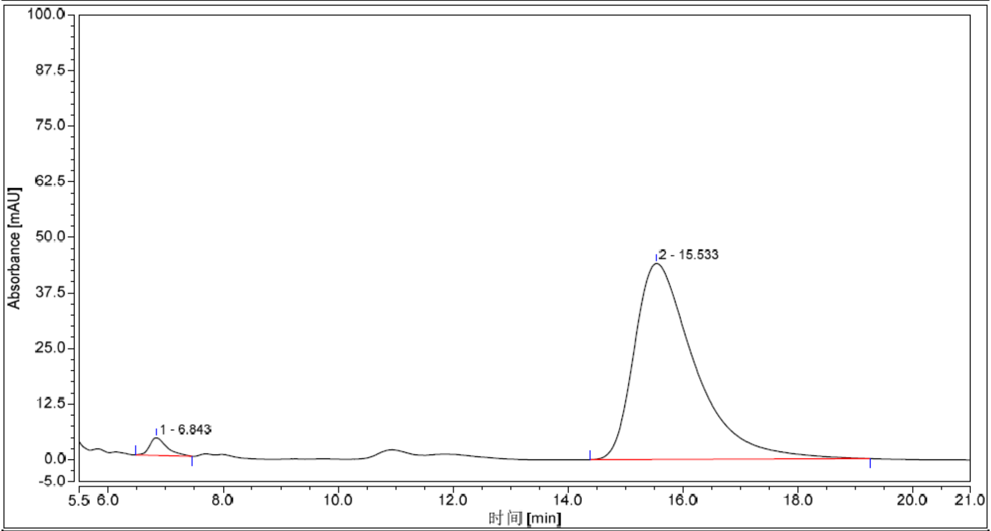

Integration Results

No.	Peak Name	Retention Time<br>min	Area<br>mAU*min	Height<br>mAU	Relative Area<br>%	Relative Height<br>%	Amount<br>n.a.
1		6.843	1.392	3.984	2.53	8.29	n.a.
2		15.533	53.737	44.079	97.47	91.71	n.a.
**Total:**			**55.129**	**48.063**	**100.00**	**100.00**	

Supplementary Figure 139. HPLC spectrum for compound **5u (minor)**

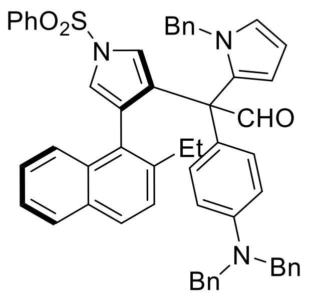

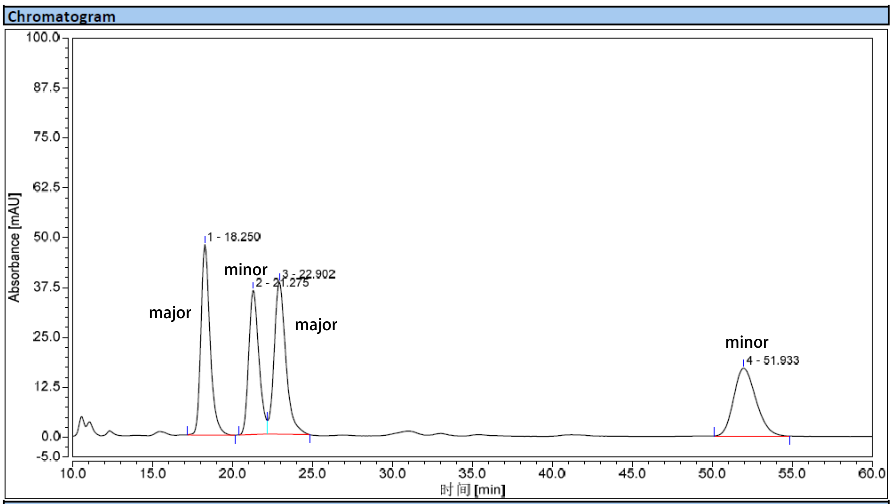

Integration Results

No.	Peak Name	Retention Time min	Area mAU*min	Height mAU	Relative Area %	Relative Height %	Amount n.a.
1		18.250	31.760	47.804	26.92	34.35	n.a.
2		21.275	26.549	36.107	22.51	25.95	n.a.
3		22.902	32.275	38.244	27.36	27.48	n.a.
4		51.933	27.381	16.997	23.21	12.21	n.a.
Total:			117.965	139.151	100.00	100.00	

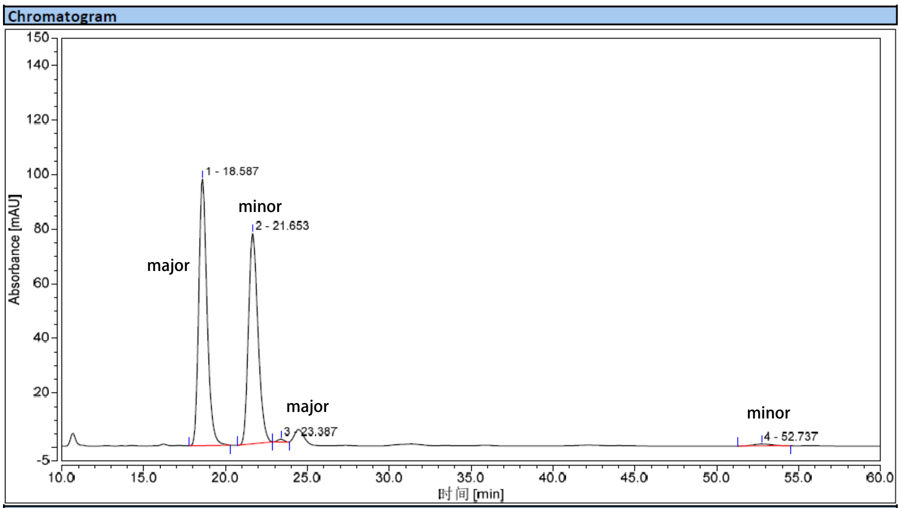

Integration Results

No.	Peak Name	Retention Time min	Area mAU*min	Height mAU	Relative Area %	Relative Height %	Amount n.a.
1		18.587	57.419	97.715	51.78	55.35	n.a.
2		21.653	52.048	77.120	46.93	43.68	n.a.
3		23.387	0.462	1.003	0.42	0.57	n.a.
4		52.737	0.970	0.702	0.87	0.40	n.a.
Total:			110.899	176.539	100.00	100.00	

Supplementary Figure 140. HPLC spectrum for compound **5v (mixture of diastereomers, 1.4:1 dr)**

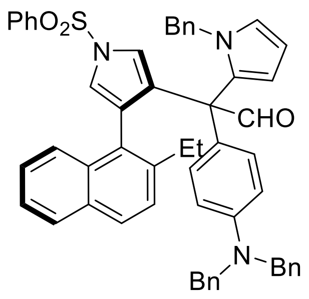

Chromatogram

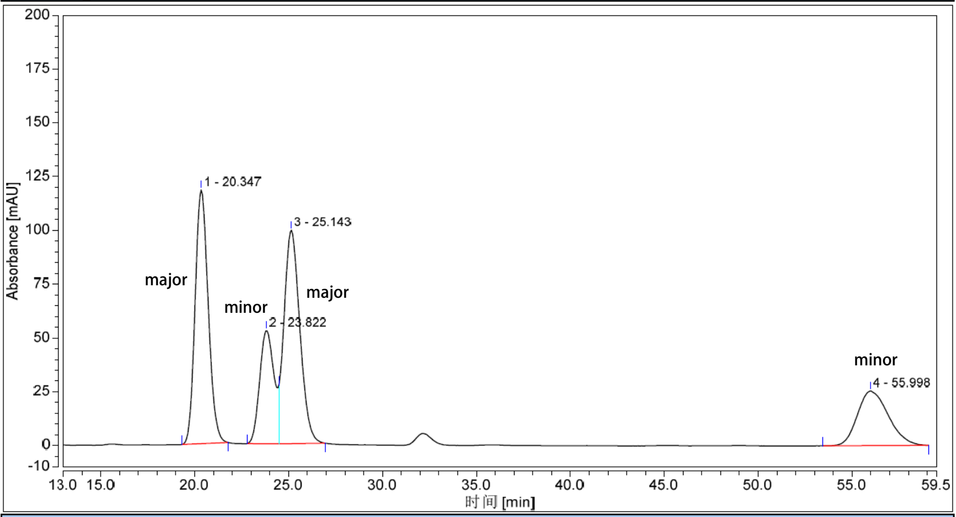

Integration Results

No.	Peak Name	Retention Time min	Area mAU*min	Height mAU	Relative Area %	Relative Height %	Amount n.a.
1		20.347	95.280	117.990	33.15	39.94	n.a.
2		23.822	46.817	52.663	16.29	17.83	n.a.
3		25.143	96.862	99.275	33.70	33.61	n.a.
4		55.998	48.493	25.482	16.87	8.63	n.a.
Total:			287.452	295.410	100.00	100.00	

Chromatogram

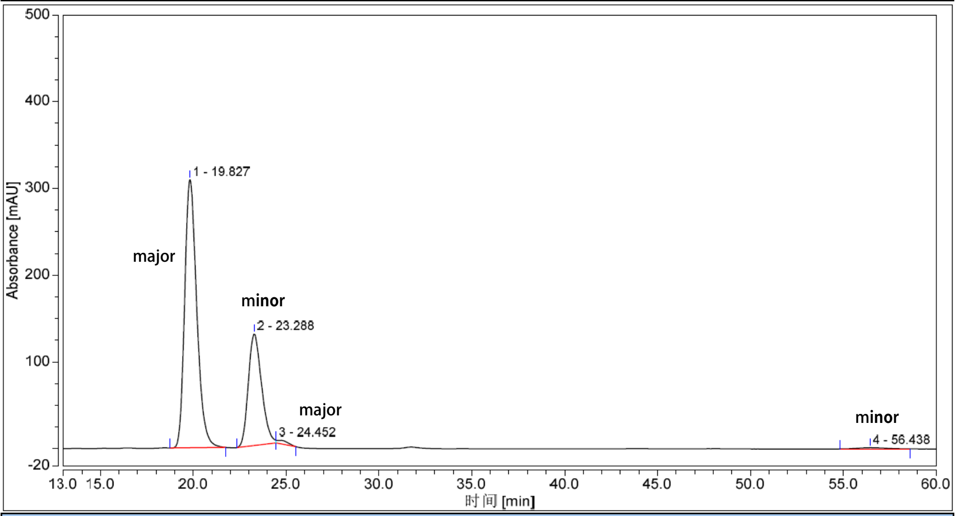

Integration Results

No.	Peak Name	Retention Time min	Area mAU*min	Height mAU	Relative Area %	Relative Height %	Amount n.a.
1		19.827	232.384	308.857	67.53	69.84	n.a.
2		23.288	106.318	128.777	30.90	29.12	n.a.
3		24.452	2.885	3.100	0.84	0.70	n.a.
4		56.438	2.508	1.493	0.73	0.34	n.a.
Total:			344.095	442.227	100.00	100.00	

Supplementary Figure 141. HPLC spectrum for compound **5v (mixture of diastereomers, 2.5:1 dr)**

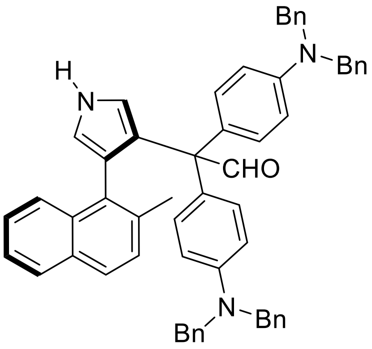

Chromatogram

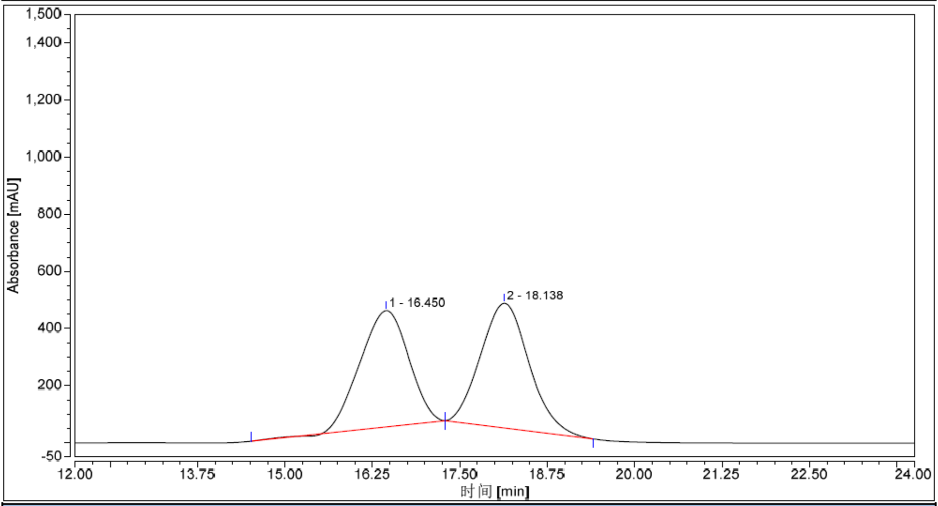

Integration Results

No.	Peak Name	Retention Time min	Area mAU*min	Height mAU	Relative Area %	Relative Height %	Amount n.a.
1		16.450	329.290	407.128	48.41	48.26	n.a.
2		18.138	350.984	436.448	51.59	51.74	n.a.
**Total:**			**680.273**	**843.576**	**100.00**	**100.00**	

Chromatogram

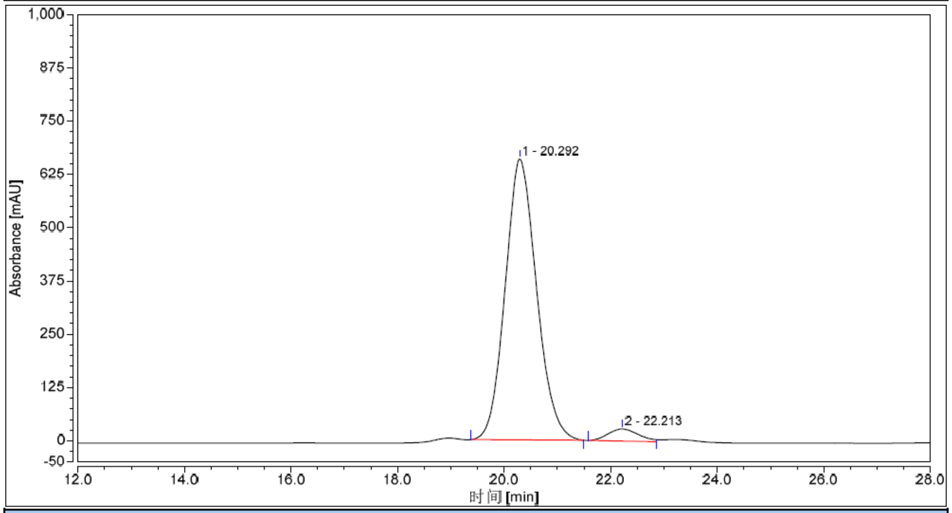

Integration Results

No.	Peak Name	Retention Time min	Area mAU*min	Height mAU	Relative Area %	Relative Height %	Amount n.a.
1		20.292	450.024	659.661	95.99	95.86	n.a.
2		22.213	18.782	28.499	4.01	4.14	n.a.
**Total:**			**468.806**	**688.160**	**100.00**	**100.00**	

Supplementary Figure 142. HPLC spectrum for compound **6**

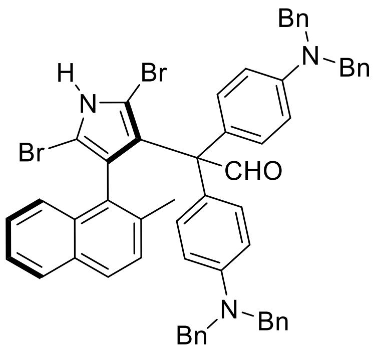

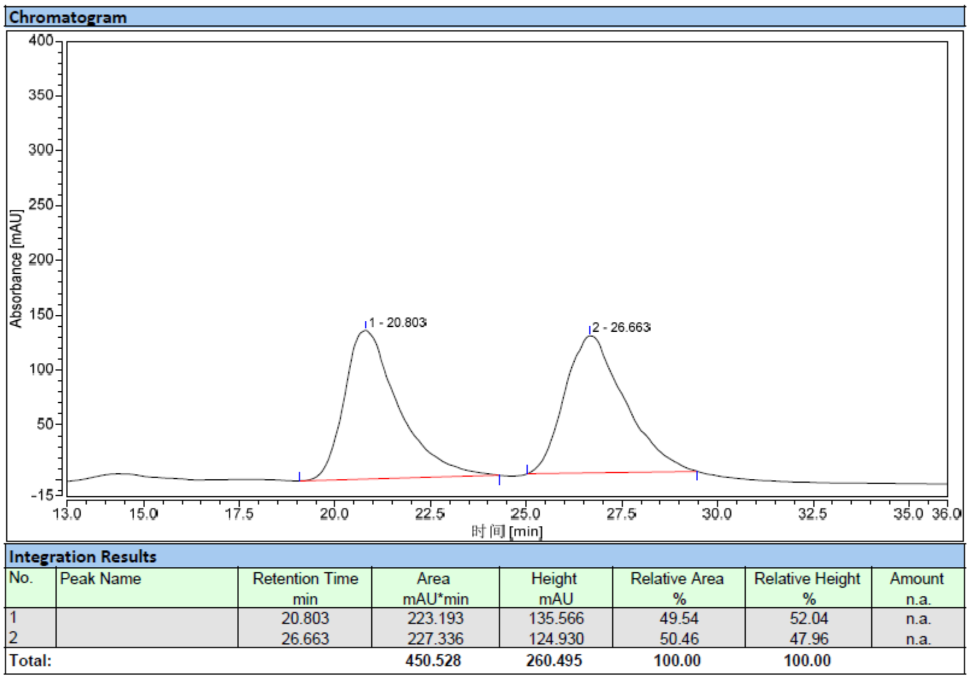

Integration Results

No.	Peak Name	Retention Time min	Area mAU*min	Height mAU	Relative Area %	Relative Height %	Amount n.a.
1		20.803	223.193	135.566	49.54	52.04	n.a.
2		26.663	227.336	124.930	50.46	47.96	n.a.
Total:			450.528	260.495	100.00	100.00	

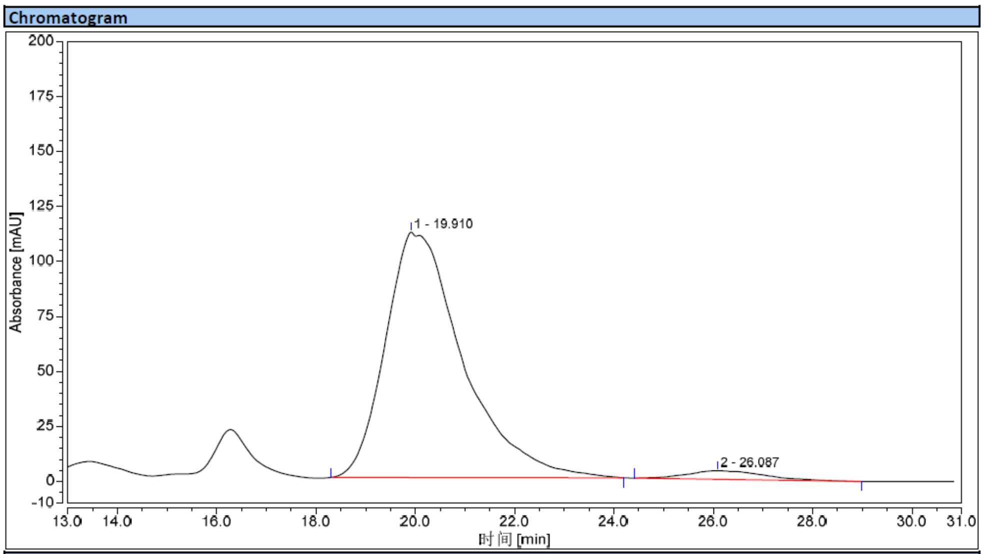

Integration Results

No.	Peak Name	Retention Time min	Area mAU*min	Height mAU	Relative Area %	Relative Height %	Amount n.a.
1		19.910	201.341	111.687	96.65	96.59	n.a.
2		26.087	6.972	3.942	3.35	3.41	n.a.
Total:			208.313	115.629	100.00	100.00	

Supplementary Figure 143. HPLC spectrum for compound **7**

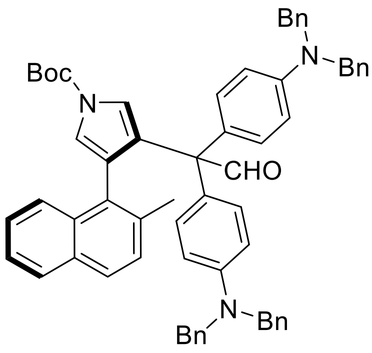

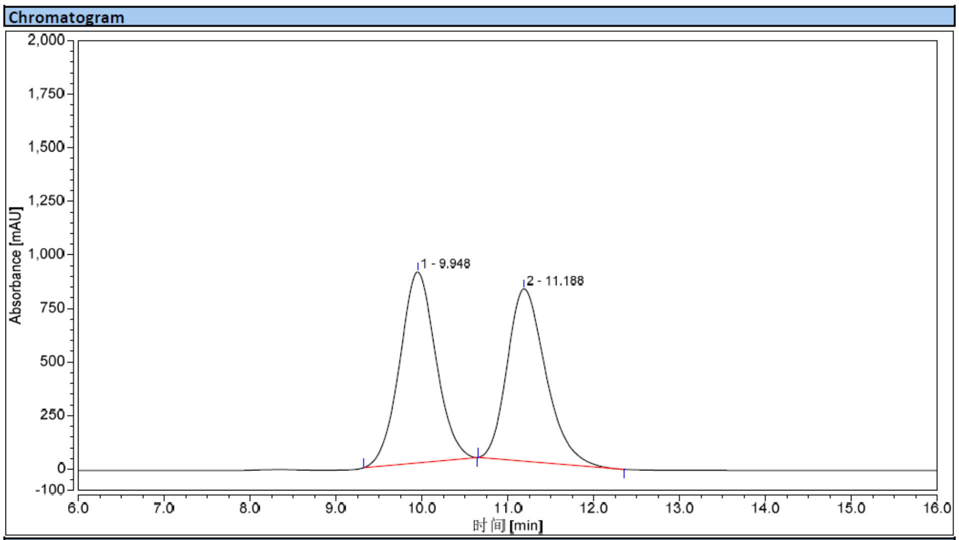

Integration Results

No.	Peak Name	Retention Time min	Area mAU*min	Height mAU	Relative Area %	Relative Height %	Amount n.a.
1		9.948	433.660	891.392	50.93	52.56	n.a.
2		11.188	417.741	804.477	49.07	47.44	n.a.
**Total:**			**851.401**	**1695.869**	**100.00**	**100.00**	

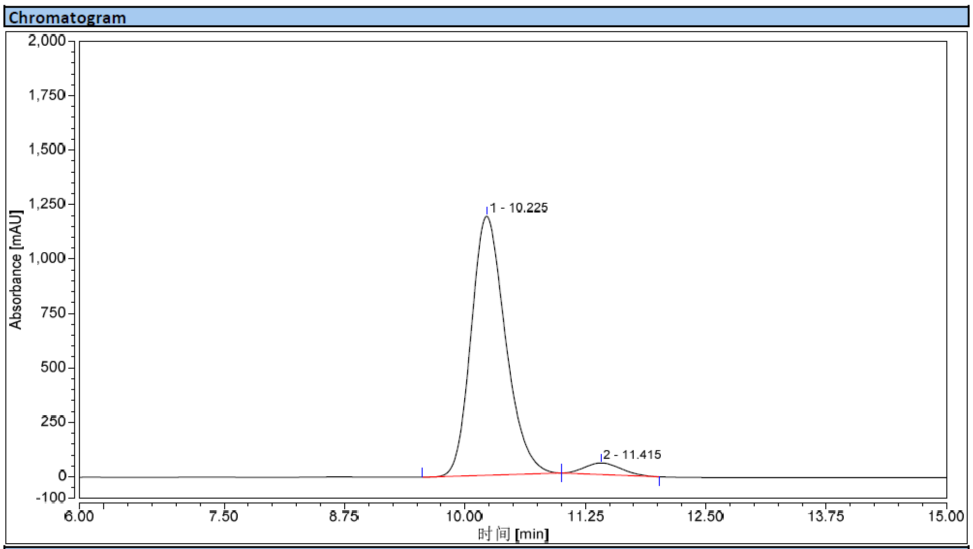

Integration Results

No.	Peak Name	Retention Time min	Area mAU*min	Height mAU	Relative Area %	Relative Height %	Amount n.a.
1		10.225	490.491	1190.674	95.45	95.71	n.a.
2		11.415	23.368	53.361	4.55	4.29	n.a.
**Total:**			**513.859**	**1244.035**	**100.00**	**100.00**	

Supplementary Figure 144. HPLC spectrum for compound **8**

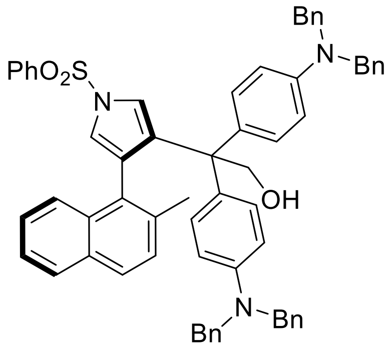

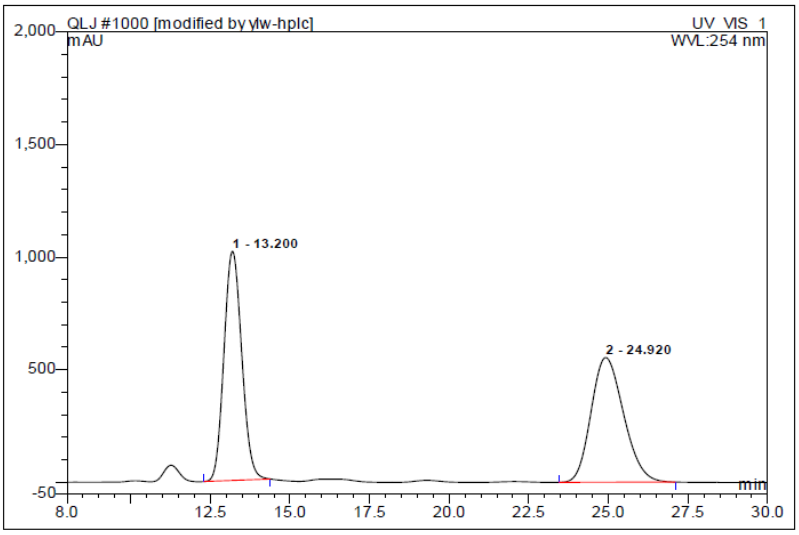

No.	Ret.Time min	Peak Name	Height mAU	Area mAU*min	Rel.Area %	Amount n.a.	Type
1	13.20	n.a.	1017.353	658.177	50.08	n.a.	BMB*
2	24.92	n.a.	553.495	655.954	49.92	n.a.	BMB*
Total:			1570.849	1314.131	100.00	0.000	

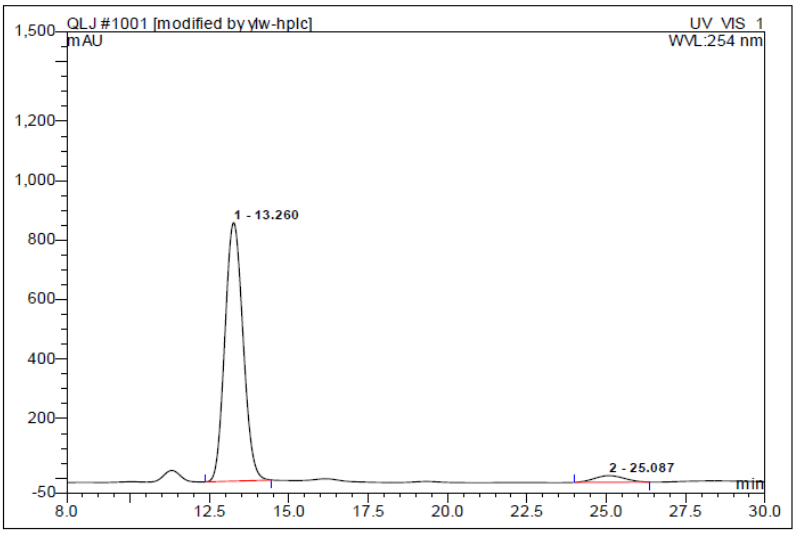

No.	Ret.Time min	Peak Name	Height mAU	Area mAU*min	Rel.Area %	Amount n.a.	Type
1	13.26	n.a.	869.139	576.632	95.98	n.a.	BMB*
2	25.09	n.a.	22.029	24.147	4.02	n.a.	BMB*
Total:			891.168	600.779	100.00	0.000	

Supplementary Figure 145. HPLC spectrum for compound **9**

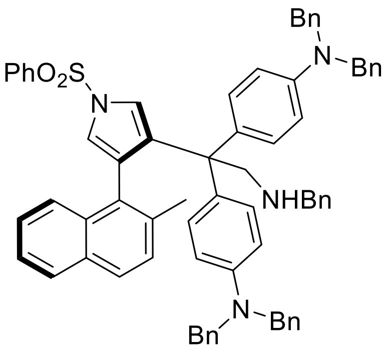

Chromatogram

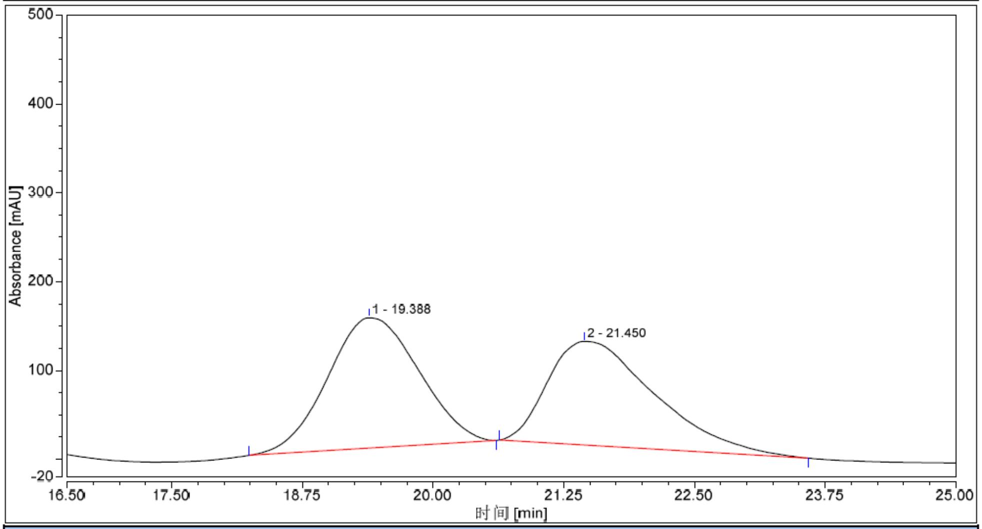

Integration Results

No.	Peak Name	Retention Time min	Area mAU*min	Height mAU	Relative Area %	Relative Height %	Amount n.a.
1		19.388	147.762	146.726	51.63	55.69	n.a.
2		21.450	138.432	116.720	48.37	44.31	n.a.
**Total:**			**286.194**	**263.445**	**100.00**	**100.00**	

Chromatogram

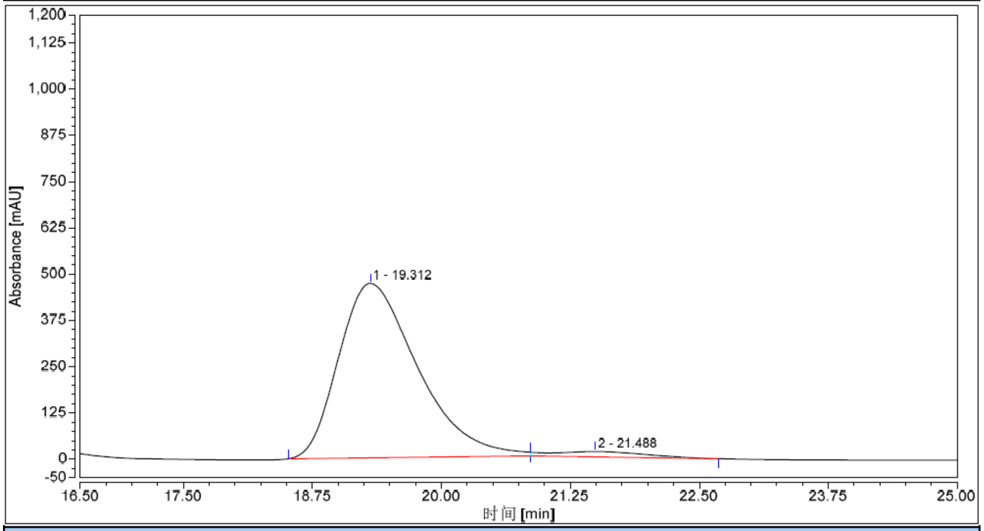

Integration Results

No.	Peak Name	Retention Time min	Area mAU*min	Height mAU	Relative Area %	Relative Height %	Amount n.a.
1		19.312	417.294	471.388	96.52	97.08	n.a.
2		21.488	15.056	14.177	3.48	2.92	n.a.
**Total:**			**432.349**	**485.565**	**100.00**	**100.00**	

Supplementary Figure 146. HPLC spectrum for compound **10**

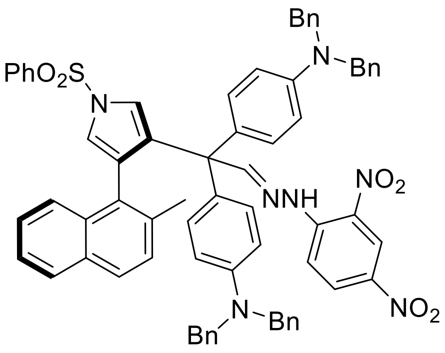

Chromatogram

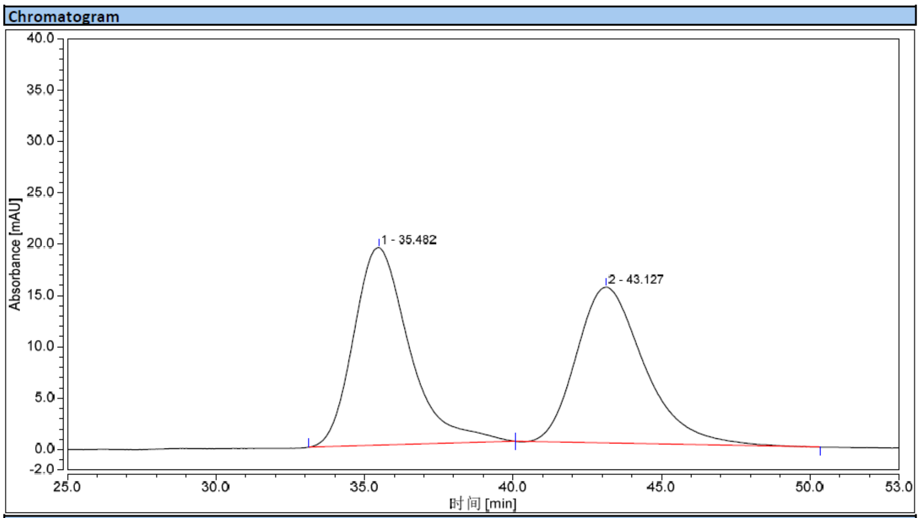

Integration Results

No.	Peak Name	Retention Time<br>min	Area<br>mAU*min	Height<br>mAU	Relative Area<br>%	Relative Height<br>%	Amount<br>n.a.
1		35.482	41.845	19.253	50.97	55.90	n.a.
2		43.127	40.244	15.189	49.03	44.10	n.a.
**Total:**			**82.089**	**34.442**	**100.00**	**100.00**	

Chromatogram

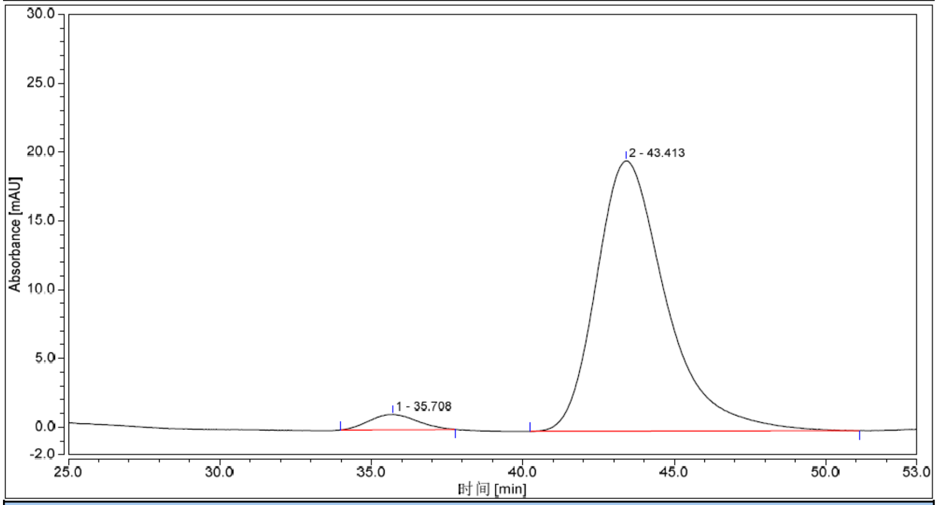

Integration Results

No.	Peak Name	Retention Time<br>min	Area<br>mAU*min	Height<br>mAU	Relative Area<br>%	Relative Height<br>%	Amount<br>n.a.
1		35.708	2.038	1.118	3.74	5.39	n.a.
2		43.413	52.392	19.648	96.26	94.61	n.a.
**Total:**			**54.430**	**20.766**	**100.00**	**100.00**	

Supplementary Figure 147. HPLC spectrum for compound **11**

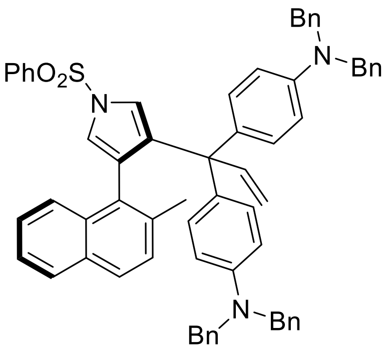

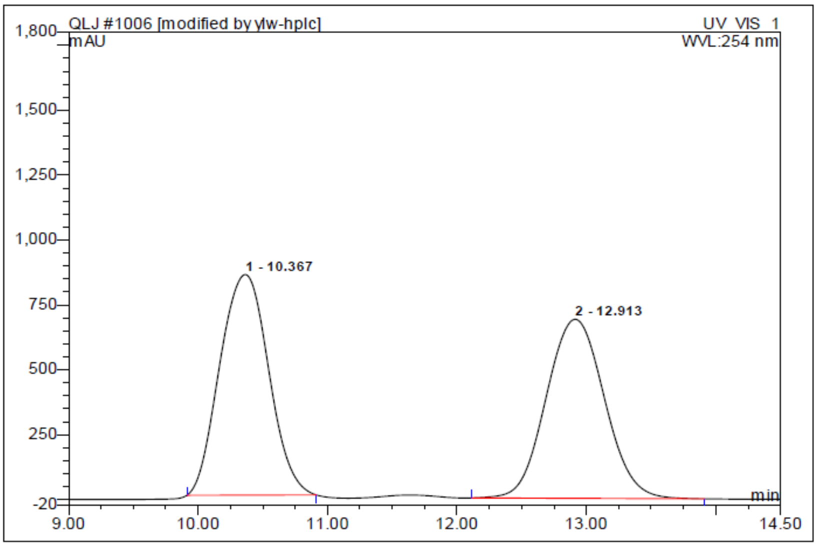

No.	Ret.Time min	Peak Name	Height mAU	Area mAU*min	Rel.Area %	Amount n.a.	Type
1	10.37	n.a.	849.536	371.272	50.81	n.a.	BMB*
2	12.91	n.a.	689.667	359.492	49.19	n.a.	BMB*
Total:			1539.203	730.764	100.00	0.000	

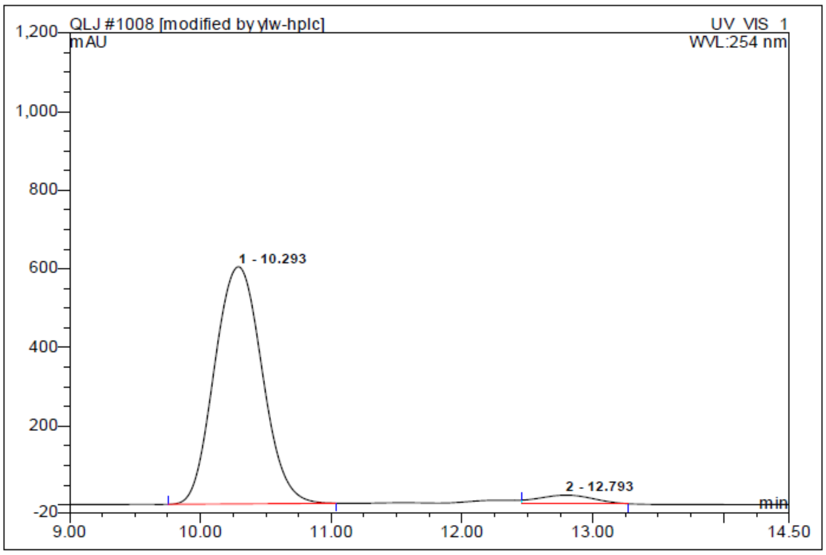

No.	Ret.Time min	Peak Name	Height mAU	Area mAU*min	Rel.Area %	Amount n.a.	Type
1	10.29	n.a.	603.014	254.260	96.00	n.a.	BMB*
2	12.79	n.a.	21.769	10.585	4.00	n.a.	MB*
Total:			624.783	264.844	100.00	0.000	

Supplementary Figure 148. HPLC spectrum for compound **12**

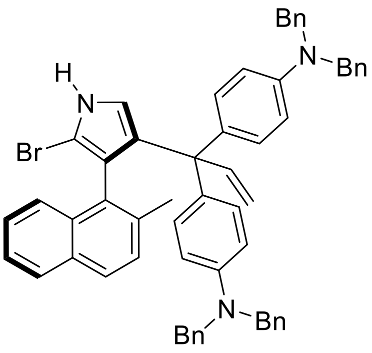

Chromatogram

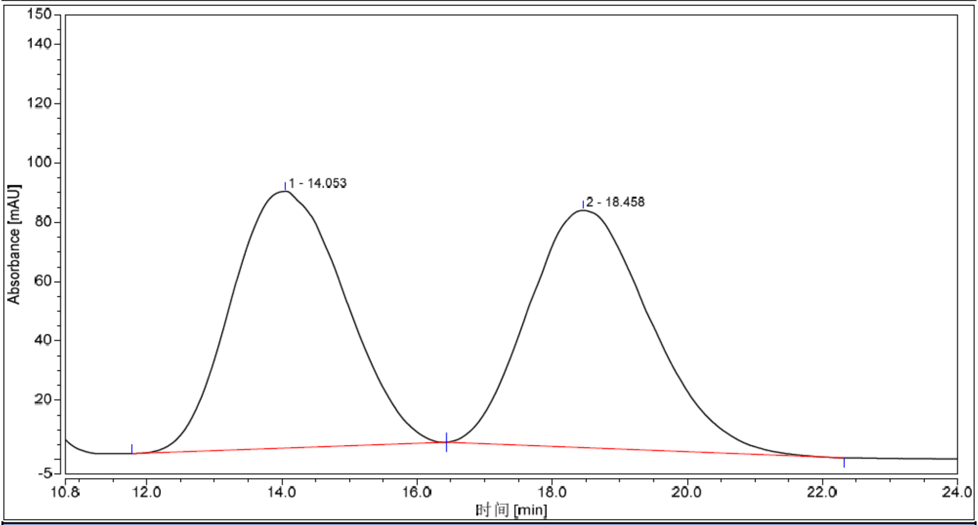

Integration Results

No.	Peak Name	Retention Time min	Area mAU*min	Height mAU	Relative Area %	Relative Height %	Amount n.a.
1		14.053	168.176	86.664	50.08	51.98	n.a.
2		18.458	167.632	80.077	49.92	48.02	n.a.
**Total:**			**335.808**	**166.741**	**100.00**	**100.00**	

Chromatogram

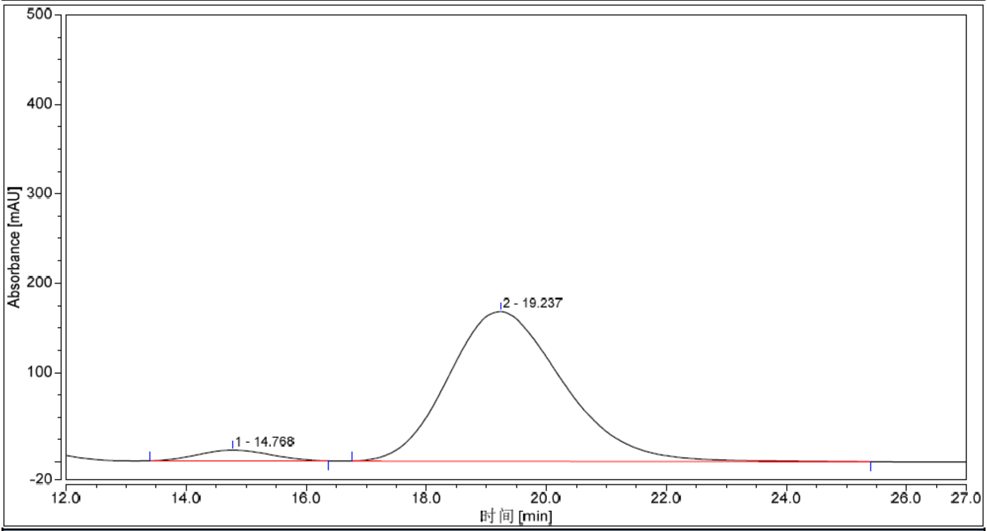

Integration Results

No.	Peak Name	Retention Time min	Area mAU*min	Height mAU	Relative Area %	Relative Height %	Amount n.a.
1		14.768	16.719	11.948	4.32	6.67	n.a.
2		19.237	370.322	167.295	95.68	93.33	n.a.
**Total:**			**387.041**	**179.243**	**100.00**	**100.00**	

Supplementary Figure 149. HPLC spectrum for compound **13**

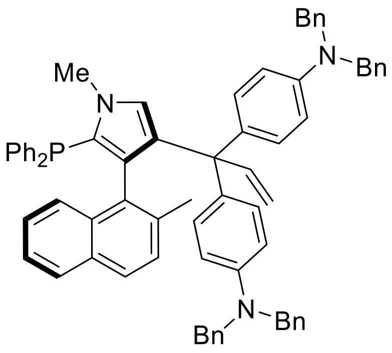

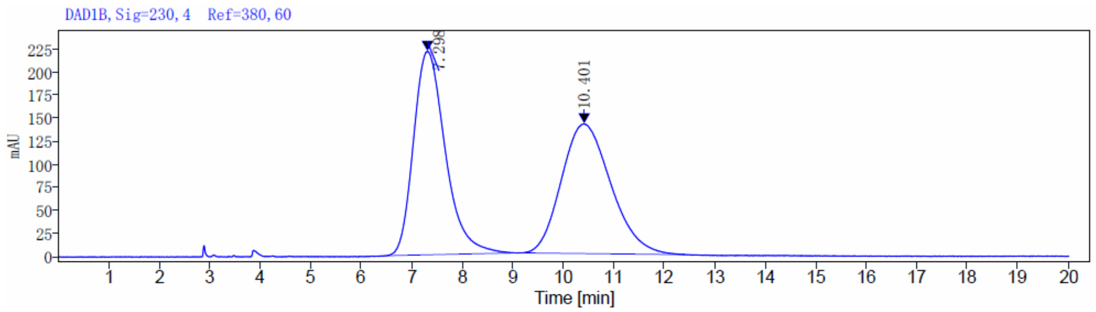

Signal: DAD1B, Sig=230, 4 Ref=380, 60

RetTime [min]	Type	Width [min]	Area [mAU*s]	Height [mAU]	Area %
7.298	MM m	2.57017	9655.77540	221.25471	50.2184
10.401	MM m	3.21272	9571.78153	141.27856	49.7816
		Totals	19227.55693		

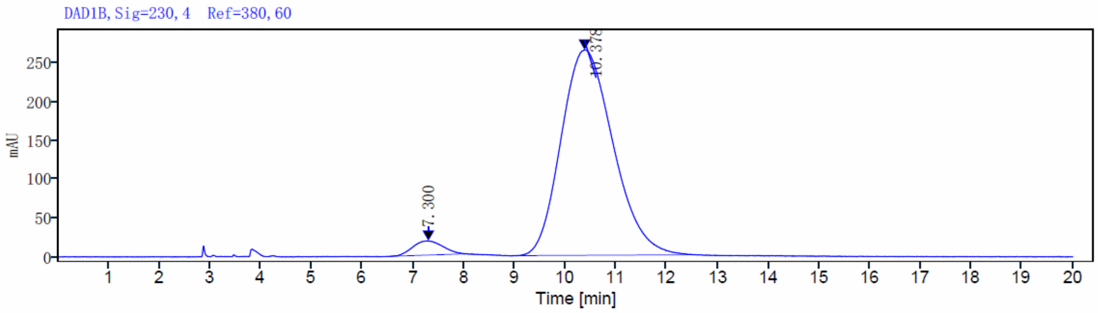

Signal: DAD1B, Sig=230, 4 Ref=380, 60

RetTime [min]	Type	Width [min]	Area [mAU*s]	Height [mAU]	Area %
7.300	MM m	1.50998	750.80771	18.34969	3.8219
10.378	MM m	3.48580	18894.17331	265.39107	96.1781
		Totals	19644.98102		

Supplementary Figure 150. HPLC spectrum for compound **14**

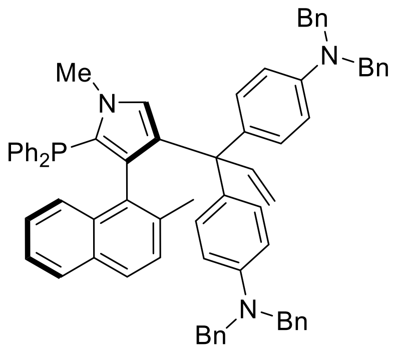

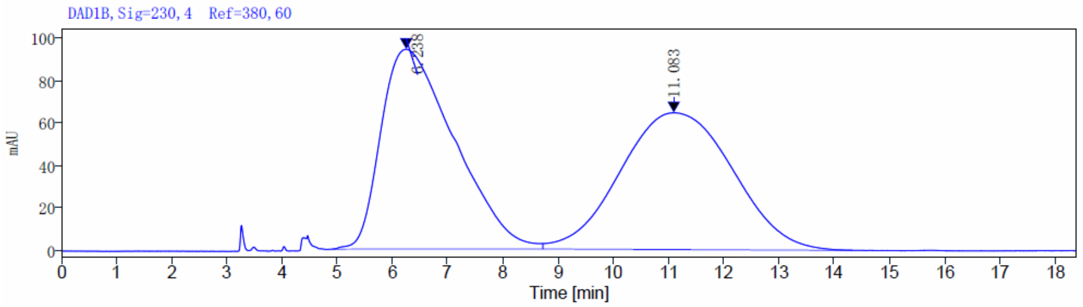

Signal: DAD1B, Sig=230, 4 Ref=380, 60

RetTime [min]	Type	Width [min]	Area [mAU*s]	Height [mAU]	Area %
6.238	MM m	3.80108	8718.62149	94.34176	48.7811
11.083	MM m	5.61390	9154.34453	64.64101	51.2189
		Totals	17872.96602		

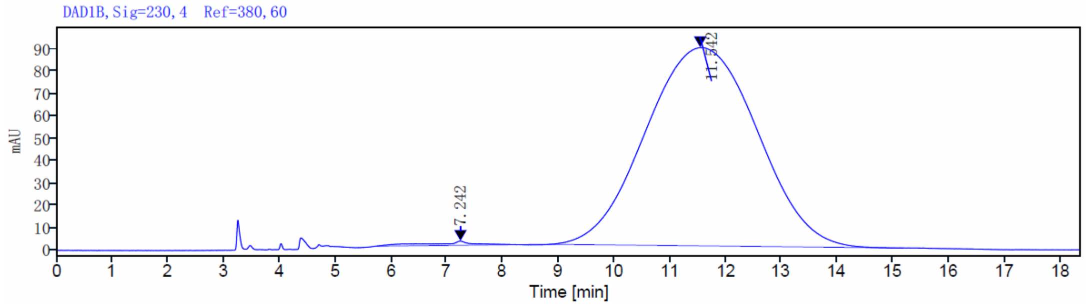

Signal: DAD1B, Sig=230, 4 Ref=380, 60

RetTime [min]	Type	Width [min]	Area [mAU*s]	Height [mAU]	Area %
7.242	MM m	3.12284	94.66677	1.85784	0.7618
11.542	MM m	6.33001	12331.89577	88.61922	99.2382
		Totals	12426.56254		

Supplementary Figure 151. HPLC spectrum for compound **14 (98% ee)**

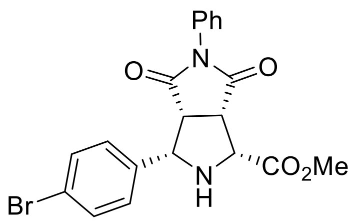

Chromatogram

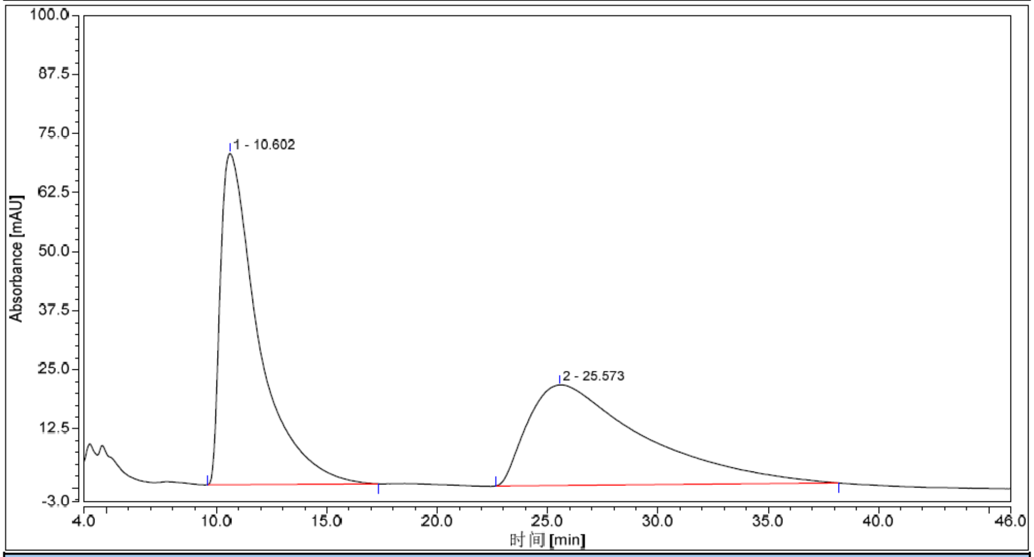

Integration Results

No.	Peak Name	Retention Time min	Area mAU*min	Height mAU	Relative Area %	Relative Height %	Amount n.a.
1		10.602	139.886	70.180	51.99	76.70	n.a.
2		25.573	129.192	21.316	48.01	23.30	n.a.
**Total:**			**269.079**	**91.496**	**100.00**	**100.00**	

Chromatogram

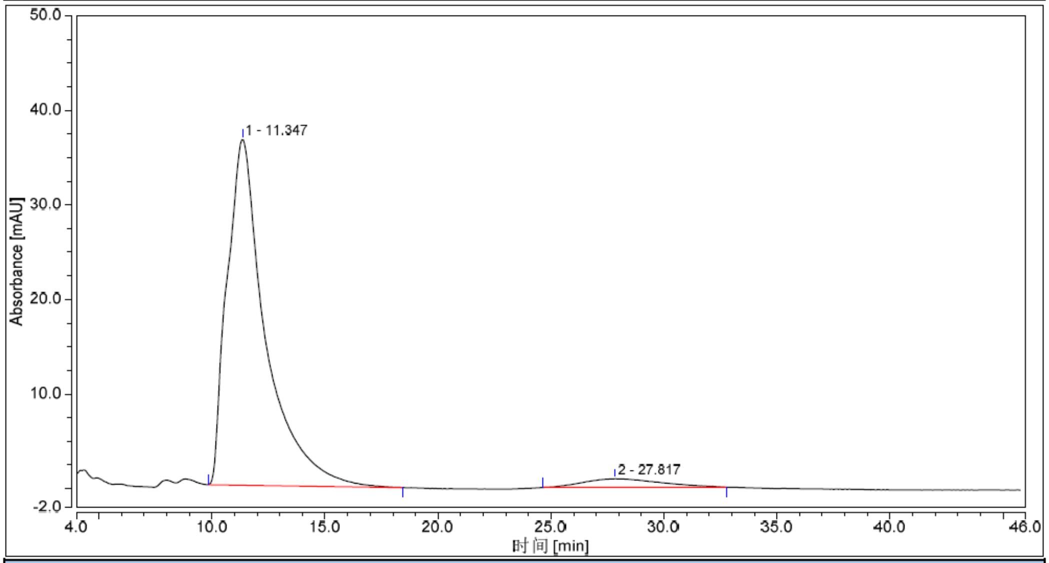

Integration Results

No.	Peak Name	Retention Time min	Area mAU*min	Height mAU	Relative Area %	Relative Height %	Amount n.a.
1		11.347	71.938	36.595	95.32	97.59	n.a.
2		27.817	3.528	0.903	4.68	2.41	n.a.
**Total:**			**75.466**	**37.498**	**100.00**	**100.00**	

Supplementary Figure 152. HPLC spectrum for compound **17**

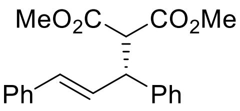

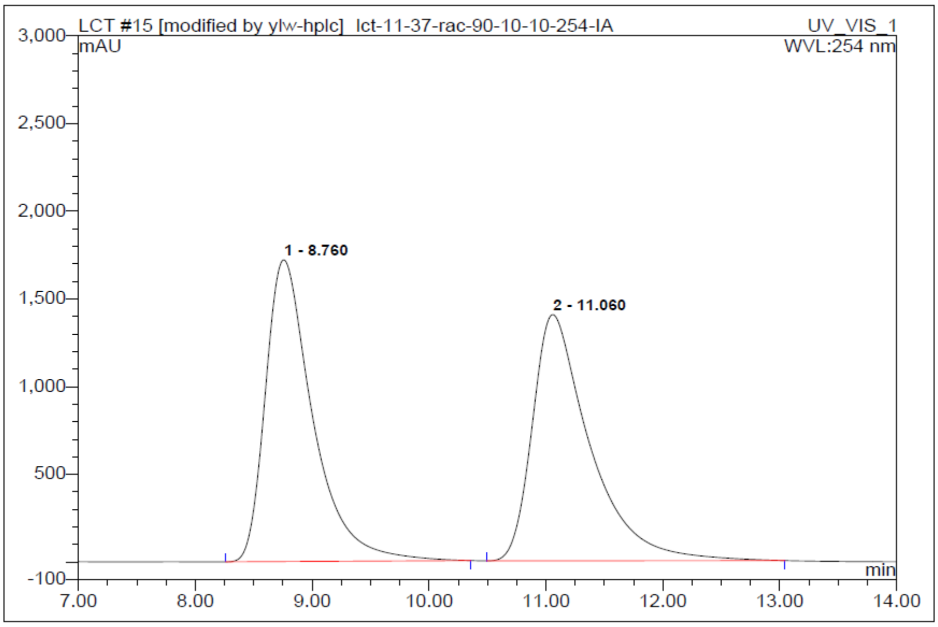

No.	Ret.Time min	Peak Name	Height mAU	Area mAU*min	Rel.Area %	Amount n.a.	Type
1	8.76	n.a.	1719.910	808.338	50.21	n.a.	BMB*
2	11.06	n.a.	1403.658	801.451	49.79	n.a.	BMB*
Total:			3123.568	1609.789	100.00	0.000	

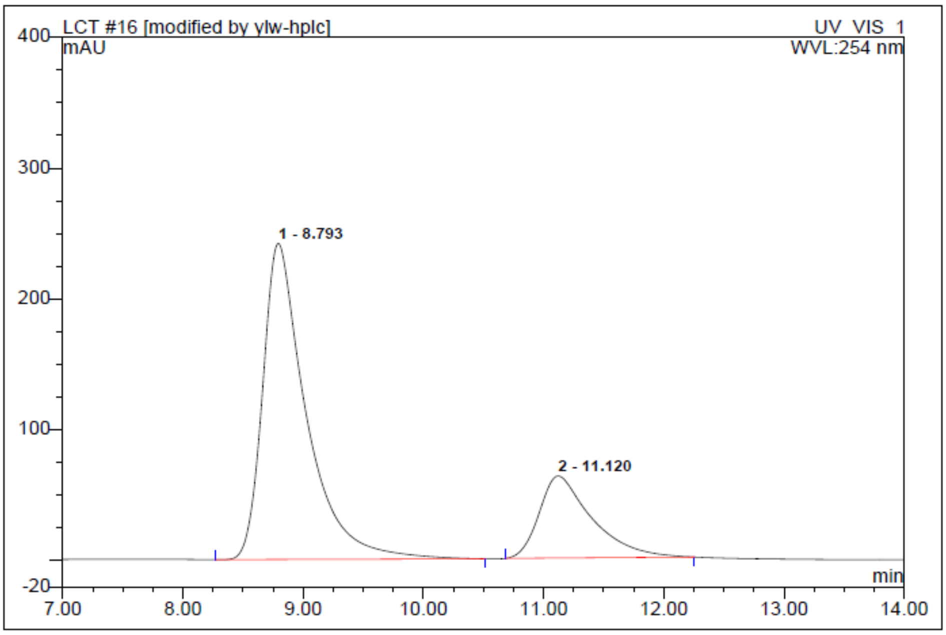

No.	Ret.Time min	Peak Name	Height mAU	Area mAU*min	Rel.Area %	Amount n.a.	Type
1	8.79	n.a.	241.500	101.303	76.18	n.a.	BMB*
2	11.12	n.a.	62.644	31.682	23.82	n.a.	BMB*
Total:			304.144	132.985	100.00	0.000	

Supplementary Figure 153. HPLC spectrum for compound **20**

II. Supplementary References

1. Lu, L., Siu, J. C., Lai, Y. & Lin, S. An electroreductive approach to radical silylation via the activation of strong Si–Cl bond. *J. Am. Chem. Soc.* **142**, 21272–21278 (2020).

2. Nelson, G. et al. Synthesis and evaluation of p-*N*,*N*-dialkyl substituted chalcones as anti-cancer agents. *Med. Chem. Res*. **22**, 4610–4614 (2013).

3. Reddy, Y. T., Reddy, P. N., Koduru, S., Damodaran, C. & Crooks, P. A. Aplysinopsin analogs: synthesis and anti-proliferative activity of substituted (*Z*)-5-(*N*-benzylindol-3-ylmethylene)imidazolidine-2,4-diones. *Bioorg. Med. Chem.* **18**, 3570–3574 (2010).

4. Sala, R. et al. Redox-neutral Ru(0)-catalyzed alkenylation of 2-carboxaldimine-heterocyclopentadienes. *J. Org. Chem*. **87**, 4640–4648 (2022).

5. Champagne, P. A., Benhassine, Y., Desroches, J. & Paquin, J.-F. Friedel–Crafts reaction of benzyl fluorides: selective activation of C–F bonds as enabled by hydrogen bonding. *Angew. Chem. Int. Ed*. **53**, 13835–13839 (2014).

6. Hong, F.-L. et al. Generation of donor/donor copper carbenes through copper-catalyzed diyne cyclization: enantioselective and divergent synthesis of chiral polycyclic pyrroles. *J. Am. Chem. Soc.* **141**, 16961–16970 (2019).

7. Xu, H.-J. et al. Copper-catalyzed formal [4 + 1] annulation of *N*-propargyl ynamides with diketones. *Org. Chem. Front.* **10**, 203–208 (2023).

8. Coste, A., Karthikeyan, G., Couty, F. & Evano, G. Copper-mediated coupling of 1,1-dibromo-1-alkenes with nitrogen nucleophiles: a general method for the synthesis of ynamides. *Angew. Chem. Int. Ed.* **48**, 4381–4385 (2009).

9. Li, C.-W. et al. Gold-catalyzed oxidative ring expansions and ring cleavages of alkynylcyclopropanes by intermolecular reactions oxidized by diphenylsulfoxide.

Angew. Chem. Int. Ed. **49**, 9891–9894 (2010).

10. Rajasekar, S. & Anbarasan, P. Rhodium-catalyzed transannulation of 1,2,3-triazoles to polysubstituted pyrroles. *J. Org. Chem*. **79**, 8428–8434 (2019).

11. Gilow, H. M. & Burton, D. E. Bromination and chlorination of pyrrole and some reactive 1-substituted pyrroles. *J. Org. Chem*. **46**, 2221–2225 (1981).

12. Kerr, W. J. et al. Site-selective deuteration of *N*-heterocycles via Iridium-catalyzed hydrogen isotope exchange. *ACS Catal*. **7**, 7182–7186 (2017).

13. Cee, V. J. et al. Discovery and optimization of macrocyclic quinoxaline-pyrrolo-dihydropiperidinones as potent pim-1/2 kinase inhibitors. *ACS Med. Chem. Lett.* **7**, 408–412 (2016).

14. Raina, G. et al. Programmed synthesis of triarylnitroimidazoles via sequential cross-coupling reactions. *Org. Biomol. Chem.* **17**, 2134–2147 (2019).

15. Johnson, K. R. D., Hannon, M. A., Ritch, J. S. & Hayes, P. G. Thermally stable rare earth dialkyl complexes supported by a novel bis(phosphinimine)pyrrole ligand. *Dalton Trans*. **41**, 7873–7875 (2012).

16. Wu, Y., Xu, B., Liu, B., Zhang, Z.-M. & Liu, Y. A new trifluoromethylated sulfonamide phosphine ligand for Ag(I)-catalyzed enantioselective [3 + 2] cycloaddition of azomethine ylides. *Org. Biomol. Chem.* **17**, 1395–1401 (2019).

17. Xia, W., An, Q.-J., Xiang, S.-H., Li, S., Wang, Y.-B. & Tan, B. *Angew. Chem. Int. Ed.* **59**, 6775–6779 (2020).

18. Frisch, M. J. et al. *Gaussian 16, Revision A.03, Gaussian, Inc., Wallingford CT*, (2016).

19. Becke, A. D. Density-functional thermochemistry. III. The role of exact exchange. *J. Chem. Phys.* **98**, 5648–5652 (1993).

20. Lee, C., Yang, W. & Parr, R. G. Development of the colle-salvetti correlation-energy formula into a functional of the electron density. *Phys. Rev. B* **37**, 785–789 (1988).

21. Wadt, W. R. & Hay, P. J. Ab initio effective core potentials for molecular calculations. Potentials for main group elements Na to Bi. *J. Chem. Phys*. **82**, 284–298 (1985).

22. Hay, P. J. & Wadt, W. R. Ab initio effective core potentials for molecular calculations. Potentials for the transition metal atoms Sc to Hg. *J. Chem. Phys*. **82**, 270–283 (1985).

23. Woon, D. E. & Dunning, T. H. Gaussian basis sets for use in correlated molecular calculations. V. Core-valence basis sets for boron through neon. *J. Chem. Phys.* **103**, 4572–4585 (1995).

24. Weigend, F. & Ahlrichs, R. Balanced basis sets of split valence, triple zeta valence and quadruple zeta valence quality for H to Rn: Design and assessment of accuracy. *Phy. Chem. Chem. Phy.* **7**, 3297–3305 (2005).

25. Weigend, F. Accurate Coulomb-fitting basis sets for H to Rn. *Phy. Chem. Chem. Phy.* **8**, 1057–1065 (2006).

26. Marenich, A. V., Cramer, C. J. & Truhlar, D. G. Universal solvation model based on solute electron density and on a continuum model of the solvent defined by the bulk dielectric constant and atomic surface tensions. *J. Phys. Chem. B* **113**, 6378–6396 (2009).

27. CYLview20; Legault, C. Y. Université de Sherbrooke, **2020**, (http://www.cylview.org).
